# Supplementary material for: Equivalent user experience and improved community augmented meta-analyses knowledge for a new version of a Plain Language Summary guideline
Source: PLoS One. 2024 May 9;19(5):e0300675. doi: 10.1371/journal.pone.0300675 (PMC11081257; doi:10.1371/journal.pone.0300675)
Supplement: S1 File — https://doi.org/10.23668/psycharchives.14217. (PDF) [file pone.0300675.s001.pdf]

S1. Original German PLSs and English translations

The following pages contain the German PLS texts originally presented to lay readers in the context of our study, as well as newly generated English translations. As stated, the original PLSs summarized published, peer-reviewed psychological meta-analyses conducted by Barth et al. (2013) and Färber and Rosendahl (2018). For Barth et al., we created two different versions: One based on the old guideline, and one based on the new guideline. For Färber and Rosendahl, we similarly created a version based on the old guideline, a version based on the new guideline, and a version based on the new guideline with additional CAMA-elements. Experimental manipulations were only included in the “new guideline”-versions and were introduced in the sections “Was sagen die Ergebnisse aus” (causality statement) and “Hinweis der KLARtext-Autor:innen” (disclaimer on the extent of evaluation). The English versions were created via DeepL and lightly edited by the primary author. Please note that we did not use the English versions in our study. They should therefore be closely examined and validated before using them for a direct replication of our findings in an English language context.

Barth et. al, Old Guideline

Welche Psychotherapien helfen Erwachsenen mit einer Depression am besten?

Eine Übersichtsarbeit

Diese Zusammenfassung bezieht sich auf die Übersichtsarbeit mit dem Titel „Comparative Efficacy of Seven Psychotherapeutic Interventions for Patients with Depression: A Network Meta-Analysis“ von Jürgen Barth und anderen aus dem Jahr 2013. Die Forschenden arbeiten an der Universität Bern und zwei weiteren Instituten.

Gut zu wissen

Die Übersichtsarbeit, zu der Sie heute eine Zusammenfassung lesen, ist eine Metaanalyse. Forschende, die eine Metaanalyse machen, suchen zuerst die Ergebnisse aller Studien zu einer bestimmten Frage (zum Beispiel “Wie gut hilft eine bestimmte Psychotherapie?”). Dann fassen sie die Ergebnisse dieser Studien zusammen.

Warum macht man das? Eine Metaanalyse hat zwei Ziele:

1. Die Metaanalyse gibt einen Überblick über alle Studien, die diese Frage schon untersucht haben. Sie beschreibt, wer schon zu einer Frage geforscht hat (zum Beispiel, wer schon alles diese Psychotherapie untersucht hat). Außerdem steht dort, was in den einzelnen Studien herauskam (zum Beispiel, ob und wie gut die Psychotherapie geholfen hat).

2. Die Metaanalyse liefert außerdem einen Wert, der die Ergebnisse aller Studien zusammenfasst. Dafür nehmen die Forschenden die Einzelergebnisse aller gefundenen Studien und berechnen daraus ein Gesamtergebnis. Es ist dabei wichtig, dass die gefundenen Studien zu einem Thema ziemlich ähnlich sind. Wenn sich die Studien sehr unterscheiden, wird das berechnete Gesamtergebnis der Metaanalyse ungenau. Das berechnete Gesamtergebnis liefert den Forschenden die Antwort auf die anfangs gestellte Frage (zum Beispiel “Über alle Studien hinweg hilft die Psychotherapie sehr gut.”). Dieses Gesamtergebnis hat eine viel höhere Aussagekraft als die Ergebnisse der einzelnen Studien.

Was war das Ziel der Übersichtsarbeit?

Hintergrund: Psychotherapien können Menschen mit psychischen Erkrankungen helfen. Das hat die Forschung bereits herausgefunden. Es gibt verschiedene Arten von Psychotherapien. Bei Erwachsenen mit einer leichten bis mittelschweren Depression helfen diese womöglich unterschiedlich gut.

Forschungsfrage: In dieser Übersichtsarbeit wollten die Forschenden herausfinden: Welche Arten von Psychotherapien helfen Erwachsenen mit leichten bis mittelschweren Depressionen?

Kernaussage der Übersichtsarbeit

Alle untersuchten Arten von Psychotherapien helfen Erwachsenen mit Depressionen ähnlich gut. Sie helfen alle besser als gar keine Psychotherapie.

Wie sind die Forschenden vorgegangen?

Welche Studien haben die Forschenden gesucht?
Die Forschenden suchten nach Studien, die Arten von Psychotherapien miteinander oder gegenüber keiner Psychotherapie verglichen. Die Studien sollten untersuchen, wie gut die jeweilige Psychotherapie Erwachsenen mit Depression hilft.

Welche Studien haben die Forschenden gefunden?
Die Forschenden fanden insgesamt 198 Studien aus den Jahren 1975 bis 2012, deren Ergebnisse sie zu einer Metaanalyse zusammenfassen konnten. Insgesamt sind das Studienergebnisse von 15 118 Erwachsenen mit Depressionen.

Was haben die Forschenden gemacht?
In den 198 Studien schauten die Forschenden jeweils welche Art der Psychotherapie den Erwachsenen mit einer Depression wie gut half. Sie untersuchten auch, ob der Behandlungserfolg der jeweiligen Art der Psychotherapie mit anderen Merkmalen der Betroffenen zusammenhing.

Was haben die Forschenden untersucht?

» Verschiedene Arten von Psychotherapie:
- Kognitive Verhaltenstherapie
- Nicht-direktive supportive Therapie
- Verhaltensaktivierung
- Psychodynamische Psychotherapie
- Problemlösetherapie
- Interpersonelle Psychotherapie
- Training sozialer Fertigkeiten

» Merkmale der untersuchten Erwachsenen mit Depressionen (zum Beispiel Alter)

» Behandlungserfolg: Schwere der Depression nach einer Psychotherapie

Was sind die wichtigsten Ergebnisse?

» Jede Art der Psychotherapie half besser als keine Psychotherapie zu machen. Die Effektstärke Cohen's d betrug über die 198 Studien hinweg d = - 0.62 bis d = - 0.92. Das sind mittelgroße bis große Unterschiede zwischen Erwachsenen mit Depressionen, die keine Psychotherapie machten und denen, die eine Psychotherapie machten.

» Beim Vergleich von je zwei Arten der Psychotherapie miteinander, half eine Psychotherapieart wesentlich besser als eine andere. Cohen's d betrug hier d = - 0.01 bis d = - 0.29. Das sind praktisch nicht bedeutsame Unterschiede zwischen den Arten der Psychotherapie. Lediglich die Interpersonelle Psychotherapie half etwas besser als die Nicht-direktive supportive Therapie. Cohen's d betrug hier d = - 0.30. Das ist ein kleiner Unterschied zwischen diesen beiden Therapiearten.

» Die Forschenden fanden heraus, dass sich die Merkmale der untersuchten Erwachsenen nicht darauf ausgewirkt haben, wie gut eine Psychotherapie half. Somer's d betrug hier d = - 0.08. Das ist ein praktisch nicht bedeutsamer Einfluss solcher Merkmale auf den Behandlungserfolg.

Was bedeuten die Ergebnisse im Alltag?

Jede in dieser Übersichtsarbeit untersuchte Art der Psychotherapie kann zur Behandlung von Depressionen bei Erwachsenen empfohlen werden. Im Vergleich zu keiner Behandlung trägt eine Psychotherapie eher dazu bei, die Schwere der Depression zu mindern.

Was ist noch zu beachten?

Verzerrung der Ergebnisse durch eingeschränktes Veröffentlichen von Studien

» *Worum geht es?* Eindeutige Forschungsergebnisse lassen sich leichter veröffentlichen als uneindeutige Ergebnisse. Das ist für Übersichtsarbeiten problematisch, weil sie diese unveröffentlichten, uneindeutigen Ergebnisse nicht mitberücksichtigen können.

» *Was bedeutet das für die vorliegende Übersichtsarbeit?* Die Forschenden dieser Übersichtsarbeit fanden Hinweise auf solche unveröffentlichten Studien mit uneindeutigen Ergebnissen. Sie nehmen deshalb an, dass der Behandlungserfolg einzelner Psychotherapien tatsächlich kleiner ist als in ihrer Übersichtsarbeit berechnet.

Wer hat die Studie finanziert?
Die Forschung wurde durch den Schweizerischen Nationalfonds finanziert. Eine Stiftung, die Forschung fördert.

Gab es Interessenkonflikte?
Die Forschenden berichten in ihrer Übersichtsarbeit, dass folgende Interessenkonflikte bei ihnen vorliegen: Ein Forschender ist Mitglied einer Steuerungsgruppe für Studien der Pharmaindustrie. Einige der Forschenden gehören einer universitären Forschungseinrichtung (CTU Bern) an. Diese unterstützt Studien der Pharmaindustrie.

Which psychotherapies best help adults with depression?

A review

This summary refers to the 2013 review paper titled "Comparative Efficacy of Seven Psychotherapeutic Interventions for Patients with Depression: A Network Meta-Analysis" by Jürgen Barth and others. The researchers work at the University of Bern and two other institutes.

Good to know

The review presented to you as a summary today is a meta-analysis. Researchers who do a meta-analysis first look for the results of all the studies on a particular question (for example, "How well does a particular psychotherapy help?"). Then they summarize the results of those studies.

Why do this? A meta-analysis has two goals:

1. The meta-analysis provides an overview of all the studies that have already examined that question. It describes who has already done research on a question (for example, who has already studied a particular type of psychotherapy). It also states the results of each study (for example, whether psychotherapy helped and how well it helped).

2. A Meta-analysis also provides a value that summarizes the results of all the studies. To do this, researchers take the individual results of all the studies they found and calculate an overall result. It is important here that the studies found on a topic are fairly similar. If the studies differ greatly, the calculated overall result of the meta-analysis becomes inaccurate. The calculated overall result provides the researchers with the answer to the question posed at the beginning (for example, "Across all studies, psychotherapy helps very well."). This overall result is much more meaningful than the results of the individual studies.

What was the goal of the review?

Background: Psychotherapy can help people with mental illness. Research has already found this out. There are different types of psychotherapies. For adults with mild to moderate depression, their helpfulness may vary.

Research question: In this review, the researchers wanted to find out: What types of psychotherapy help adults with mild to moderate depression?

Key message of the review

All examined types of psychotherapy help adults with depression similarly well. They all help better than no psychotherapy at all.

How did the researchers proceed?

What studies did the researchers look for?
The researchers looked for studies that compared types of psychotherapies with each other or with no psychotherapy. The studies should examine how well each psychotherapy helps adults with depression.

What studies did the researchers find?
The researchers found a total of 198 studies from 1975 to 2012, the results of which they were able to combine into a meta-analysis. In total, these add up to results from 15,118 adults with depression.

What did the researchers do?
In each of the 198 studies, the researchers examined the effectiveness of various psychotherapies for adults with depression. They also looked at whether the treatment success of each type of psychotherapy was related to other characteristics of the people with depression.

What did the researchers examine?
» Different types of psychotherapy:
- Cognitive behavioral therapy
- Non-directive supportive therapy
- Behavioral activation
- Psychodynamic psychotherapy
- Problem solving therapy
- Interpersonal psychotherapy
- Social skills training

» Characteristics of the examined adults with depression (for example, age).

» Treatment outcome: severity of depression after psychotherapy

What are the main findings?

» Any type of psychotherapy helped better than no psychotherapy. The effect size Cohen's d across the 198 studies was d = - 0.62 to d = - 0.92, which are moderate to large differences between adults with depression not receiving psychotherapy and those who received psychotherapy.

» When comparing each of two types of psychotherapy with each other, one type of psychotherapy helped significantly better than another. Cohen's d here was

d = - 0.01 to d = - 0.29. These are practically non-significant differences between types of psychotherapy. Only interpersonal psychotherapy helped slightly better than nondirective supportive therapy. Cohen's d here was d = - 0.30. This is a small difference between these two types of therapy.

»The researchers found that the characteristics of the adults studied did not affect how well psychotherapy helped. Somer's d here was d = - 0.08, which is a practically insignificant impact of such characteristics on treatment success.

What do the results mean in everyday life?

Every type of psychotherapy studied in this review can be recommended for the treatment of depression in adults. Compared with no treatment, psychotherapy is more likely to help reduce the severity of depression.

What else needs to be considered?

Bias in results due to limited publication of studies

»*What is the issue?* Unambiguous research results are easier to publish than ambiguous results. This is problematic for reviews because they cannot include these unpublished, inconclusive results.

»*What does this mean for this review?* The researchers of this review found evidence of such unpublished studies with inconclusive results. They therefore assume that the treatment success of individual psychotherapies is actually smaller than calculated in their review.

Who funded the study?

The research was funded by the Swiss National Science Foundation. This is a foundation that funds research.

Were there any conflicts of interest?

The researchers report in their review paper that they have the following conflicts of interest: One researcher is a member of a steering group for pharmaceutical industry studies. Some of the researchers belong to a university research institution (CTU Bern). This institution supports studies of the pharmaceutical industry.

Barth et. al, New Guideline

Welche Psychotherapien helfen Erwachsenen mit einer Depression am besten?

Eine Übersichtsarbeit

.

KLARtexte bereiten Forschungsergebnisse aus der Psychologie für die Öffentlichkeit auf. Dieser KLARtext wurde von Mitarbeitenden des Leibniz-Institut für Psychologie verfasst. Der KLARtext fasst die Übersichtsarbeit mit dem Titel „Comparative Efficacy of Seven Psychotherapeutic Interventions for Patients with Depression: A Network Meta-Analysis“ zusammen. Die Übersichtsarbeit wurde 2013 veröffentlicht. Sie stammt von Jürgen Barth und sieben weiteren Forschenden von der Universität Bern und zwei weiteren Instituten.

Gut zu wissen

Dieser KLARtext fasst eine Übersichtsarbeit, eine sogenannte Metaanalyse, zusammen. Forschende, die eine Metaanalyse machen, suchen zuerst die Ergebnisse aller Studien zu einer bestimmten Frage (zum Beispiel. “Wie gut hilft eine bestimmte Psychotherapie?”). Dann fassen sie die Ergebnisse dieser Studien zusammen.

Warum macht man das? Eine Metaanalyse hat zwei Ziele:

1. Die Metaanalyse gibt einen Überblick über alle Studien, die diese Frage schon untersucht haben. Sie beschreibt, wer schon zu einer Frage geforscht hat (zum Beispiel, wer schon alles diese Psychotherapie untersucht hat). Außerdem steht dort, was in den einzelnen Studien herauskam (zum Beispiel, ob und wie gut die Psychotherapie geholfen hat).

2. Die Metaanalyse liefert außerdem einen Wert, der die Ergebnisse aller Studien zusammenfasst. Dafür nehmen die Forschenden die Einzelergebnisse aller gefundenen Studien und berechnen daraus ein Gesamtergebnis. Es ist dabei wichtig, dass die gefundenen Studien zu einem Thema ziemlich ähnlich sind. Wenn sich die Studien sehr unterscheiden, wird das berechnete Gesamtergebnis

der Metaanalyse ungenau. Das berechnete Gesamtergebnis liefert den Forschenden die Antwort auf die anfangs gestellte Frage (zum Beispiel “Über alle Studien hinweg hilft die Psychotherapie sehr gut.”). Dieses Gesamtergebnis hat eine viel höhere Aussagekraft als die Ergebnisse der einzelnen Studien.

Was war das Ziel der Übersichtsarbeit?

Hintergrund: Psychotherapien können Menschen mit psychischen Erkrankungen helfen. Das hat die Forschung bereits herausgefunden. Es gibt verschiedene Arten von Psychotherapien. Bei Erwachsenen mit einer leichten bis mittelschweren Depression helfen diese womöglich unterschiedlich gut.

Forschungsfrage: Mit ihrer Übersichtsarbeit wollten die Forschenden herausfinden: Welche Arten von Psychotherapien helfen Erwachsenen mit leichten bis mittelschweren Depressionen?

Kernaussage der Übersichtsarbeit

Alle untersuchten Arten von Psychotherapien helfen Erwachsenen mit Depressionen ähnlich gut. Sie helfen alle besser als gar keine Psychotherapie zu machen.

Wie sind die Forschenden in der Übersichtsarbeit vorgegangen?

Welche Studien haben die Forschenden für die Übersichtsarbeit gesucht?
Die Forschenden suchten nach Studien, die Arten von Psychotherapien miteinander oder gegenüber keiner Psychotherapie verglichen. Die Studien sollten untersuchen, wie gut die jeweilige Psychotherapie Erwachsenen mit Depression hilft.

Welche Studien haben die Forschenden für die Übersichtsarbeit gefunden?
Die Forschenden fanden insgesamt 198 Studien aus den Jahren 1975 bis 2012, deren Ergebnisse sie zu einer Metaanalyse zusammenfassen konnten. Insgesamt sind das Studienergebnisse von 15 118 Erwachsenen mit Depressionen.

Was haben die Forschenden in der Übersichtsarbeit gemacht?
In den 198 Studien schauten die Forschenden jeweils, welche Art der Psychotherapie den Erwachsenen mit Depressionen wie gut half. Sie untersuchten auch, ob der Behandlungserfolg der jeweiligen Art der Psychotherapie mit anderen Merkmalen der Betroffenen zusammenhing.

Was haben die Forschenden in der Übersichtsarbeit untersucht?

» Verschiedene Arten von Psychotherapie:
- Kognitive Verhaltenstherapie
- Nicht-direktive supportive Therapie
- Verhaltensaktivierung

- Psychodynamische Psychotherapie
- Problemlösetherapie
- Interpersonelle Psychotherapie
- Training sozialer Fertigkeiten

» Merkmale der untersuchten Erwachsenen mit Depressionen (zum Beispiel Alter)

» Behandlungserfolg: Schwere der Depression nach einer Psychotherapie

Was sind die wichtigsten Ergebnisse?

» Jede Art der Psychotherapie half besser als keine Psychotherapie. Die Effektstärke Cohen's d betrug über die 198 Studien hinweg - 0.62 bis - 0.92. Das sind mittelgroße bis große Unterschiede zwischen Erwachsenen mit Depressionen, die keine Psychotherapie machten und denen, die eine Psychotherapie machten. Umgerechnet auf 100 Personen bedeutet dies: Zwischen 73 und 82 von 100 Erwachsenen waren nach einer Psychotherapie weniger depressiv als der Durchschnitt derjenigen ohne Psychotherapie.

» Beim Vergleich von je zwei Arten der Psychotherapie miteinander, half eine Psychotherapieart wesentlich besser als eine andere. Die restlichen Unterschiede im Behandlungserfolg zwischen den Arten der Psychotherapie waren nicht bedeutsam. Lediglich die Interpersonelle Psychotherapie half etwas besser als die Nicht-direktive supportive Therapie. Cohen's d betrug hier - 0.30. Das ist ein kleiner Unterschied zwischen diesen beiden Therapiearten. Umgerechnet auf 100 Personen bedeutet dies: 61 von 100 Erwachsenen mit Depressionen in einer Interpersonellen Psychotherapie hatten einen höheren Behandlungserfolg als der Durchschnitt in Nicht-direktiver supportiver Therapie.

» Die Forschenden fanden heraus, dass sich die Merkmale der untersuchten Erwachsenen nicht darauf ausgewirkt haben, wie gut eine Psychotherapie half. Der Einfluss solcher Merkmale auf den Behandlungserfolg war nicht bedeutsam.

Wie lassen sich die Ergebnisse bewerten?

Was sagen die Ergebnisse aus?

In der Übersichtsarbeit wurden Unterschiede im Behandlungserfolg zwischen zwei Arten der Psychotherapie sowie zwischen einer und keiner Psychotherapie beobachtet. Wegen der Art der Studien, die berücksichtigt wurden, kann man mit hoher Sicherheit sagen, dass die Art der Psychotherapie auch die Ursache für den Behandlungserfolg von Erwachsenen mit Depressionen ist.

Sind die Ergebnisse durch eingeschränktes Veröffentlichen von Studien verzerrt?

» **Worum geht es?** Eindeutige Forschungsergebnisse lassen sich leichter veröffentlichen als uneindeutige Ergebnisse. Das ist für Übersichtsarbeiten problematisch. Sie können unveröffentlichte Ergebnisse nämlich nicht berücksichtigen.

»**Was bedeutet das für die vorliegende Übersichtsarbeit?** Die Forschenden fanden Hinweise auf solche Verzerrungen. Sie haben sich bemüht, diese Verzerrungen zu berücksichtigen. Sie nehmen deshalb an, dass der Behandlungserfolg der Psychotherapien tatsächlich kleiner ist als in ihrer Übersichtsarbeit berechnet.

Wie zuverlässig sind die Ergebnisse?
Die Forschenden geben zu bedenken: Zum Behandlungserfolg mancher Therapiearten wurden wenige Studien gefunden. Die zugehörigen Aussagen sind daher mit Vorsicht zu betrachten. Außerdem weisen sie darauf hin, dass das Ausmaß des Behandlungserfolges von Merkmalen der eingeschlossenen Studien abhängig war. Dazu gehören zum Beispiel, wie der Behandlungserfolg gemessen wurde und wie die Studien durchgeführt wurden.

Welchen Alltagsbezug sehen die Forschenden in der Übersichtsarbeit?
Jede in dieser Übersichtsarbeit untersuchte Art der Psychotherapie kann zur Behandlung von Depressionen bei Erwachsenen empfohlen werden. Falls Sie oder jemand in Ihrem Umfeld von einer Depression betroffen ist, sollte die Entscheidung für eine bestimmte Psychotherapie erst nach Rücksprache mit Fachpersonal erfolgen.

Was ist noch zu beachten?

Wer hat die Übersichtsarbeit finanziert?
Die Erstellung der Übersichtsarbeit wurde durch den Schweizerische Nationalfonds finanziert. Das ist eine Stiftung, die Forschung fördert.

Berichten die Forschenden in der Übersichtsarbeit eigene Interessenkonflikte?
Die Forschenden berichten, dass folgende Interessenkonflikte bei ihnen vorliegen: Ein Forschender ist Mitglied einer Steuerungsgruppe für Studien der Pharmaindustrie. Einige der Forschenden gehören einer universitären Forschungseinrichtung (CTU Bern) an. Diese unterstützt Studien der Pharmaindustrie.

Hinweis der KLARtext-Autor:innen
Als KLARtext-Autor:innen fassen wir eine bereits vorhandene Übersichtsarbeit zusammen. Wir haben diese Übersichtsarbeit nicht selbst durchgeführt. Die Aussagen der Autor:innen der Übersichtsarbeit haben wir in allgemeinverständliche Sprache übersetzt. Wir überprüfen nicht, ob diese Aussagen wissenschaftlich korrekt sind. Wir überprüfen nicht, wie gut die Übersichtsarbeit durchgeführt wurde. Wir prüfen auch nicht, ob die Ergebnisse der Übersichtsarbeit bereits veraltet sind.

Link zur Übersichtsarbeit: [Link wurde zu Studienzwecken entfernt]

Which psychotherapies best help adults with depression?

A review

KLARtexts prepare research results from psychology for the public. This KLARtext was written by staff of the Leibniz Institute of Psychology. The KLARtext summarizes the review paper titled "Comparative Efficacy of Seven Psychotherapeutic Interventions for Patients with Depression: A Network Meta-Analysis." The review paper was published in 2013. It was written by Jürgen Barth and seven other researchers from the University of Bern and two other institutes.

Good to know

This KLARtext summarizes a review paper, called a meta-analysis. Researchers who do a meta-analysis first look for the results of all the studies on a particular question (for example. "How well does a particular psychotherapy help?"). Then they summarize the results of those studies.

Why do this? A meta-analysis has two goals:

1. The meta-analysis gives an overview of all the studies that have already investigated that question. It describes who has already done research on a question (for example, who has already studied a particular type of psychotherapy). It also states the results of each study (for example, whether psychotherapy helped and how well it helped).

2. Meta-analysis also provides a value that summarizes the results of all the studies. To do this, researchers take the individual results of all the studies they found and calculate an overall result. It is important here that the studies found on a topic are fairly similar. If the studies differ greatly, the calculated overall result of the meta-analysis becomes inaccurate. The calculated overall result provides the researchers with the answer to the question posed at the beginning (for example, "Across all studies, psychotherapy helps very well."). This overall result has much more power than the results of the individual studies.

What was the goal of the review?

Background: Psychotherapy can help people with mental illness. Research has already found this out. There are different types of psychotherapies. For adults with mild to moderate depression, they may help differently.

Research question: With their review, the researchers wanted to find out: What types of psychotherapies help adults with mild to moderate depression?

Key message of the review

All of the types of psychotherapies studied help adults with depression similarly well. They all help better than not doing psychotherapy at all.

How did the researchers proceed in the review?

What studies did the researchers look for in the review?

The researchers looked for studies that compared types of psychotherapy with each other or with no psychotherapy. The studies should look at how well each psychotherapy helps adults with depression.

What studies did the researchers find for the review?

The researchers found a total of 198 studies from 1975 to 2012 whose results they combined into a meta-analysis. In total, these add up to results from 15,118 adults with depression.

What did the researchers do in the review?

In each of the 198 studies, the researchers examined the effectiveness of various psychotherapies for adults with depression. They also looked at whether the treatment success of each type of psychotherapy was related to other characteristics of the people with depression.

What did the researchers examine in the review?

»Different types of psychotherapy:

- Cognitive behavioral therapy
- Non-directive supportive therapy
- Behavioral activation
- Psychodynamic psychotherapy
- Problem solving therapy
- Interpersonal psychotherapy
- Social skills training

»Characteristics of the adults with depression studied (for example, age)

»Treatment outcome: severity of depression after psychotherapy.

What are the main findings?

»Any type of psychotherapy helped better than no psychotherapy. The effect size Cohen's d across the 198 studies was - 0.62 to - 0.92, which are moderate to large differences between adults with depression not receiving psychotherapy and those who received psychotherapy. Converted to 100 people, this means: Between 73 and 82 of 100 adults were less depressed after psychotherapy than the average of those without psychotherapy.

» When comparing each of two types of psychotherapy with each other, one type of psychotherapy helped significantly better than another. The remaining differences in treatment success between types of psychotherapy were not significant. Only interpersonal psychotherapy helped slightly better than nondirective supportive therapy. Cohen's d here was - 0.30. This is a small difference between these two types of therapy. Converted to 100 people, this means 61 out of 100 adults with depression in Interpersonal Psychotherapy had higher treatment success than the average in Non-Directive Supportive Therapy.

» The researchers found that the characteristics of the adults studied did not affect how well psychotherapy helped. The impact of such characteristics on treatment success was not significant.

How can the results be evaluated?

What do the results tell us?

In the review, differences in treatment success were observed between two types of psychotherapy and between psychotherapy and no psychotherapy. Because of the types of studies that were included, it can be said with a high degree of certainty that the type of psychotherapy
is also the cause of treatment success for adults with depression.

Are the results biased by limited publication of studies?

» What is the issue? Clear research results are easier to publish than ambiguous results. This is problematic for review papers. Namely, they cannot take unpublished results into account.

» What does this mean for this review? The researchers found evidence of such bias. They made an effort to account for these biases. They therefore assume that the treatment success of psychotherapies is actually smaller than calculated in their review.

How reliable are the results?

The researchers caution: few studies were found on the treatment success of some types of therapy. The associated statements should therefore be viewed with caution. They also point out that the extent of treatment success depended on characteristics of the included studies. These include, for example, how treatment success was measured and how the studies were conducted.

What everyday relevance do the researchers see in the review?

Each type of psychotherapy studied in this review can be recommended for treating depression in adults. If you or someone close to you is affected by depression, the decision to use a particular psychotherapy should be made only after consulting with professionals.

What else should be noted?

Who funded the review?

The preparation of the review was funded by the Swiss National Science Foundation. This is a

foundation that funds research.

Do the researchers report their own conflicts of interest in the review?

The researchers report that they have the following conflicts of interest: One researcher is a member of a steering group for studies of the pharmaceutical industry. Some of the researchers belong to a university research institution (CTU Bern). This institution supports studies of the pharmaceutical industry.

Disclaimer of the KLARtext authors

As KLARtext authors, we summarize an existing review. We did not conduct this review ourselves. We have translated the statements of the authors of the review into generally understandable language. We do not check whether these statements are scientifically correct. We do not check how well the review was conducted. We also do not check whether the results of the review are already outdated.

Link to the review: [link has been removed for study purposes].

Färber and Rosendahl, Old Guideline

Wie hängen die Anpassung an schwierige Umstände und das seelische Wohlbefinden bei Menschen mit körperlichen Erkrankungen zusammen?

Eine Übersichtsarbeit

Diese Zusammenfassung bezieht sich auf die Übersichtsarbeit mit dem Titel „The Association Between Resilience and Mental Health in the Somatically Ill“ von Francesca Färber und Jenny Rosendahl aus dem Jahr 2018. Die Forschenden arbeiten an der Universität Jena.

Gut zu wissen

Die Übersichtsarbeit, zu der Sie heute eine Zusammenfassung lesen, ist eine Metaanalyse. Forschende, die eine Metaanalyse machen, suchen zuerst die Ergebnisse aller Studien zu einer bestimmten Frage (zum Beispiel “Wie gut hilft eine bestimmte Psychotherapie?”). Dann fassen sie die Ergebnisse dieser Studien zusammen.

Warum macht man das? Eine Metaanalyse hat zwei Ziele:

1. Die Metaanalyse gibt einen Überblick über alle Studien, die diese Frage schon untersucht haben. Sie beschreibt, wer schon zu einer Frage geforscht hat (z. B., wer schon alles diese Psychotherapie untersucht hat). Außerdem steht dort, was in den einzelnen Studien herauskam (zum Beispiel, ob und wie gut die Psychotherapie geholfen hat).

2. Die Metaanalyse liefert außerdem einen Wert, der die Ergebnisse aller Studien zusammenfasst. Dafür nehmen die Forschenden die Einzelergebnisse aller gefundenen Studien und berechnen daraus ein Gesamtergebnis. Es ist dabei wichtig, dass die gefundenen Studien zu einem Thema ziemlich ähnlich sind. Wenn sich die Studien sehr unterscheiden, wird das berechnete Gesamtergebnis der Metaanalyse ungenau. Das berechnete Gesamtergebnis liefert den Forschenden die Antwort auf die anfangs gestellte Frage (zum Beispiel “Über alle Studien hinweg hilft die Psychotherapie sehr gut.”). Dieses Gesamtergebnis hat eine viel höhere Aussagekraft als die Ergebnisse der einzelnen Studien.

Was war das Ziel der Übersichtsarbeit?

Hintergrund: Eine körperliche Erkrankung wird häufig als belastend erlebt. Wie belastend eine solche Erkrankung für das seelische Wohlbefinden ist, hängt auch von den Eigenschaften einer Person ab. Dazu gehört die Eigenschaft, sich gut an schwierige Umstände anzupassen.

Forschungsfrage: Mit ihrer Übersichtsarbeit wollten die Forschenden herausfinden: Wie hängen die individuelle Anpassung an schwierige Umstände und das seelische Wohlbefinden bei Menschen mit körperlichen Erkrankungen zusammen?

Kernaussage der Übersichtsarbeit

Bei Menschen mit körperlichen Erkrankungen hängen die individuelle Anpassung an schwierige Umstände und das seelische Wohlbefinden zusammen: Diejenigen, die sich besser anpassen, haben ein höheres Wohlbefinden.

Wie sind die Forschenden vorgegangen?

Welche Studien haben die Forschenden gesucht?
Die Forschenden suchten Studien zum Zusammenhang zwischen der individuellen Anpassung an schwierige Umstände und seelischem Wohlbefinden. Teilnehmende der Studien mussten Menschen mit körperlichen Erkrankungen sein.

Welche Studien haben die Forschenden gefunden?
Die Forschenden fanden insgesamt 55 Studien aus den Jahren 2006 bis 2018, deren Ergebnisse sie zu einer Metaanalyse zusammenfassen konnten. Insgesamt sind das Studienergebnisse von 15 003 Teilnehmenden.

Was haben die Forschenden gemacht?
In den 55 Studien schauten die Forschenden, wie die Anpassung an schwierige Umstände und das seelische Wohlbefinden zusammenhingen. Dabei interessierte sie die Größe und Richtung dieses Zusammenhangs: Geht eine bessere Anpassung mit höherem oder niedrigerem Wohlbefinden einher? Sie untersuchten auch den Einfluss von Eigenschaften der Studien auf diesen Zusammenhang.

Was haben die Forschenden untersucht?

- Individuelle Anpassung an schwierige Umstände
- Eigenschaften der Studien: zum Beispiel die Anzahl der Teilnehmenden
- Seelisches Wohlbefinden

Was sind die wichtigsten Ergebnisse?

- Die Korrelation r betrug $r = 0.43$. Das ist ein mittelgroßer Zusammenhang zwischen der Anpassung an schwierige Umstände und dem seelischen Wohlbefinden bei Menschen mit körperlicher Erkrankung.
- Der Einfluss Beta der Anzahl der Teilnehmenden einer Studie auf den Zusammenhang betrug Beta = -0.003. Dies ist ein sehr kleiner Einfluss. Der Zusammenhang zwischen der Anpassung und dem Wohlbefinden wurde kleiner, je mehr Teilnehmende eine Studie

hatte.

Was bedeuten die Ergebnisse im Alltag?

Für Menschen mit körperlicher Erkrankung, die sich schlechter an schwierige Umstände anpassen können, ist möglicherweise äußere Unterstützung besonders wichtig für ihr seelisches Wohlbefinden. Die individuelle Anpassung an schwierige Umstände kann mit Hilfe von Fachpersonen eingeschätzt und trainiert werden. So kann das seelische Wohlbefinden bei einer körperlichen Erkrankung gegebenenfalls weniger beeinträchtigt sein.

Was ist noch zu beachten?

Verzerrung der Ergebnisse durch eingeschränktes Veröffentlichen von Studien

»**Worum geht es?** Eindeutige Forschungsergebnisse lassen sich leichter veröffentlichen als uneindeutige Ergebnisse. Das ist für Übersichtsarbeiten problematisch, weil sie diese unveröffentlichten, uneindeutigen Ergebnisse nicht mitberücksichtigen können.

»**Was bedeutet das für die vorliegende Übersichtsarbeit?** Die Forschenden fanden Hinweise auf solche unveröffentlichte Studien mit uneindeutigen Ergebnissen und haben sich bemüht, diese zu berücksichtigen. Sie nehmen deshalb an, dass der Zusammenhang zwischen der Anpassung an schwierige Umstände und dem seelischen Wohlbefinden tatsächlich ähnlich groß ist wie in ihrer Übersichtsarbeit berechnet.

Wer hat die Übersichtsarbeit finanziert?
In der Veröffentlichung der Übersichtsarbeit können keine Angaben dazu gefunden werden, wie die Forschung finanziert wurde.

Gab es Interessenkonflikte?
In der Veröffentlichung der Übersichtsarbeit geben die Forschenden an, dass keine Interessenkonflikte vorliegen.

How are adjustment to difficult circumstances and psychological well-being related in people with physical illness?

A review

This summary refers to the 2018 review paper titled "The Association Between Resilience and Mental Health in the Somatically Ill" by Francesca Färber and Jenny Rosendahl, researchers at the University of Jena, Germany.

Good to know

The review presented to you as a summary today is a meta-analysis. Researchers who do a meta-analysis first look for the results of all the studies on a particular question (for example, "How well does a particular psychotherapy help?"). Then they summarize the results of those studies.

Why do this? A meta-analysis has two goals:

1. The meta-analysis gives an overview of all the studies that have already investigated that question. It describes who has already done research on a question (for example, who has already studied a particular type of psychotherapy). It also states the results of each study (for example, whether psychotherapy helped and how well it helped).

2. A Meta-analysis also provides a value that summarizes the results of all the studies. To do this, researchers take the individual results of all the studies they found and calculate an overall result. It is important here that the studies found on a topic are fairly similar. If the studies differ greatly, the calculated overall result of the meta-analysis becomes inaccurate. The calculated overall result provides the researchers with the answer to the question posed at the beginning (for example, "Across all studies, psychotherapy helps very well."). This overall result has much more power than the results of the individual studies.

What was the goal of the review?

Background: A physical illness is often experienced as stressful. How burdensome such an illness is for mental well-being also depends on a person's characteristics. These traits encompass the ability to adapt effectively in challenging situations.

Research question: With their review, the researchers wanted to find out: How are individual adaptation to difficult circumstances and psychological well-being related in people with physical illness?

Key message of the review

In people with physical illnesses, individual adjustment to difficult circumstances and psychological well-being are related: Those who adapt better have higher well-being.

How did the researchers proceed?

What studies did the researchers look for?
The researchers sought studies on the relationship between individual adaptation to difficult circumstances and psychological well-being. Participants in the studies had to be people with physical illnesses.

What studies did the researchers find?
The researchers identified a total of 55 studies conducted between 2006 and 2018, and combined their results into a meta-analysis. In total, these add up to results from 15,003 participants.

What did the researchers do?
In the 55 studies, the researchers looked at how adaptation to difficult circumstances and psychological well-being were related. They were interested in the magnitude and direction of this relationship: is better adjustment associated with higher or lower well-being? They also examined the influence of study characteristics on this relationship.

What did the researchers examine?

- » Individual adaptation to difficult circumstances
- » Characteristics of the studies: for example, the number of participants
- » Mental well-being

What are the main results?

- » The correlation r was r = 0.43, which is a moderately large correlation between adaptation to difficult circumstances and psychological well-being in people with physical illness.

- » The influence beta of the number of participants in a study on the correlation was beta = -0.003. This is a very small influence. The correlation between adjustment and well-being decreased as the number of participants in a study increased.

What do the results mean in everyday life?

For people with physical illness who are less able to adapt to difficult circumstances, external support may be particularly important for their psychological well-being. Individual adaptation to difficult circumstances can be assessed and trained with the help of professionals. In this way, mental well-being may be less impaired in the event of a physical illness.

What else should be considered?

Bias in results due to limited publication of studies.

- » What is the issue? Clear research results are easier to publish than ambiguous results. This is problematic for reviews because they cannot include these unpublished, inconclusive results.

- » What does that mean for this review? The researchers found evidence of such unpublished studies with inconclusive results and made an effort to include them. They therefore hypothesize that the association between adjustment to difficult circumstances and psychological well-being is indeed similar in magnitude to that calculated in their review.

Who funded the review paper?
No information can be found in the review publication about how the research was funded.

Were there any conflicts of interest?
In the publication of the review paper, the researchers state that there were no conflicts of interest.

Färber and Rosendahl, New Guideline without CAMA Elements

Wie hängen die Anpassung an schwierige Umstände und das seelische Wohlbefinden bei Menschen mit körperlichen Erkrankungen zusammen?

Eine Übersichtsarbeit

KLARtexte bereiten Forschungsergebnisse aus der Psychologie für die Öffentlichkeit auf. Dieser KLARtext wurde von Mitarbeitenden des Leibniz-Instituts für Psychologie verfasst. Der KLARtext fasst die Übersichtsarbeit mit dem Titel „The Association Between Resilience and Mental Health in the Somatically Ill“ zusammen. Die Übersichtsarbeit wurde 2018 veröffentlicht. Sie stammt von den Forschenden Francesca Färber und Jenny Rosendahl von der Universität Jena.

Gut zu wissen

Dieser KLARtext fasst eine Übersichtsarbeit, eine sogenannte Metaanalyse, zusammen. Forschende, die eine Metaanalyse machen, suchen zuerst die Ergebnisse aller Studien zu einer bestimmten Frage (zum Beispiel “Wie gut hilft eine bestimmte Psychotherapie?”). Dann fassen sie die Ergebnisse dieser Studien zusammen.

Warum macht man das? Eine Metaanalyse hat zwei Ziele:

1. Die Metaanalyse gibt einen Überblick über alle Studien, die diese Frage schon untersucht haben. Sie beschreibt, wer schon zu einer Frage geforscht hat (zum Beispiel, wer schon alles diese Psychotherapie untersucht hat). Außerdem steht dort, was in den einzelnen Studien herauskam (zum Beispiel, ob und wie gut die Psychotherapie geholfen hat).

2. Die Metaanalyse liefert außerdem einen Wert, der die Ergebnisse aller Studien zusammenfasst. Dafür nehmen die Forschenden die Einzelergebnisse aller gefundenen Studien und berechnen daraus ein Gesamtergebnis. Es ist dabei wichtig, dass die gefundenen Studien zu einem Thema ziemlich ähnlich sind. Wenn sich die Studien sehr unterscheiden, wird das berechnete Gesamtergebnis der Metaanalyse ungenau. Das berechnete Gesamtergebnis liefert den Forschenden die Antwort auf die anfangs gestellte Frage (zum Beispiel “Über alle Studien hinweg hilft die Psychotherapie sehr gut.”). Dieses Gesamtergebnis hat eine viel höhere Aussagekraft als die Ergebnisse der einzelnen Studien.

Was war das Ziel der Übersichtsarbeit?

Hintergrund: Eine körperliche Erkrankung wird häufig als belastend erlebt. Wie belastend eine solche Erkrankung für das seelische Wohlbefinden ist, hängt auch von den Eigenschaften einer Person ab. Dazu gehört die Eigenschaft, sich gut an schwierige Umstände anzupassen.

Forschungsfrage: Mit ihrer Übersichtsarbeit wollten die Forschenden herausfinden: Wie hängen die individuelle Anpassung an schwierige Umstände und das seelische Wohlbefinden bei Menschen mit körperlichen Erkrankungen zusammen?

Kernaussage der Übersichtsarbeit

Bei Menschen mit körperlichen Erkrankungen hängen die individuelle Anpassung an schwierige Umstände und das seelische Wohlbefinden zusammen: Diejenigen, die sich besser anpassen, haben ein höheres Wohlbefinden.

Wie sind die Forschenden in der Übersichtsarbeit vorgegangen?

Welche Studien haben die Forschenden für die Übersichtsarbeit gesucht?
Die Forschenden suchten Studien zum Zusammenhang zwischen der individuellen Anpassung an schwierige Umstände und seelischem Wohlbefinden. Teilnehmende der Studien mussten Menschen mit körperlichen Erkrankungen sein.

Welche Studien haben die Forschenden für die Übersichtsarbeit gefunden?
Die Forschenden fanden insgesamt 55 Studien aus den Jahren 2006 bis 2018, deren Ergebnisse sie zu einer Metaanalyse zusammenfassen konnten. Insgesamt sind das Studienergebnisse von 15 003 Teilnehmenden.

Was haben die Forschenden in der Übersichtsarbeit gemacht?
In den 55 Studien schauten die Forschenden, wie die Anpassung an schwierige Umstände und das seelische Wohlbefinden zusammenhingen. Dabei interessierte sie die Größe und Richtung dieses Zusammenhangs: Geht eine bessere Anpassung mit höherem oder niedrigerem Wohlbefinden einher? Sie untersuchten auch den Einfluss von Eigenschaften der Studien auf diesen Zusammenhang.

Was haben die Forschenden in der Übersichtsarbeit untersucht?

- Individuelle Anpassung an schwierige Umstände
- Eigenschaften der Studien: zum Beispiel die Anzahl der Teilnehmenden
- Seelisches Wohlbefinden

Was sind die wichtigsten Ergebnisse?

- Die Korrelation r betrug 0.43. Das ist ein mittelgroßer Zusammenhang zwischen der Anpassung an schwierige Umstände und dem seelischen Wohlbefinden bei Menschen mit

körperlicher Erkrankung.

- Der Einfluss Beta der Anzahl der Teilnehmenden einer Studie auf den Zusammenhang betrug -0.003. Dies ist ein sehr kleiner Einfluss. Der Zusammenhang zwischen der Anpassung und dem Wohlbefinden wurde kleiner, je mehr Teilnehmende eine Studie hatte.

Wie lassen sich die Ergebnisse bewerten?

Was sagen die Ergebnisse aus?

In der Übersichtsarbeit wurde ein Zusammenhang zwischen der Anpassung an schwierige Umstände und seelischem Wohlbefinden beobachtet. Wegen der Art der Studien, die berücksichtigt wurden, weiß man nur, dass es diesen Zusammenhang bei Menschen mit körperlichen Erkrankungen gibt. Man kann aber nicht sicher sagen, dass die Anpassung auch die Ursache des Wohlbefindens ist.

Sind die Ergebnisse durch eingeschränktes Veröffentlichen von Studien verzerrt?

»**Worum geht es?** Eindeutige Forschungsergebnisse lassen sich leichter veröffentlichen als uneindeutige Ergebnisse. Das ist für Übersichtsarbeiten problematisch. Sie können unveröffentlichte Ergebnisse nämlich nicht berücksichtigen.

»**Was bedeutet das für die vorliegende Übersichtsarbeit?** Die Forschenden fanden Hinweise auf solche Verzerrungen. Sie haben sich bemüht, diese Verzerrungen zu berücksichtigen. Sie nehmen deshalb an, dass der Zusammenhang zwischen der Anpassung an schwierige Umstände und dem seelischen Wohlbefinden tatsächlich ähnlich groß ist wie in ihrer Übersichtsarbeit berechnet.

Wie zuverlässig sind die Ergebnisse?

Die Forschenden geben zu bedenken: In der Forschung ist man sich nicht einig, ob die Anpassung an schwierige Umstände eine angeborene Eigenschaft ist. Es könnte auch eine erlernbare und damit trainierbare Fähigkeit sein. Außerdem schwankte die Größe des Zusammenhangs zwischen den einzelnen gefundenen Studien stark. Manche Studien fanden einen größeren andere einen kleineren Zusammenhang.

Welchen Alltagsbezug sehen die Forschenden in der Übersichtsarbeit?

Körperlich erkrankte Menschen könnten während ihrer Behandlung möglicherweise besser unterstützt werden. Fachpersonal könnte frühzeitig die individuelle Anpassung an schwierige Umstände einschätzen. Wer sich schlecht an schwierige Umstände anpassen kann, könnte dann besondere Hilfe bekommen.

Was ist noch zur Übersichtsarbeit zu beachten?

Wer hat die Übersichtsarbeit finanziert?

In der Übersichtsarbeit können keine Angaben dazu gefunden werden, wie diese finanziert wurde.

Berichten die Forschenden in der Übersichtsarbeit eigene Interessenkonflikte?

Die Forschenden berichten, dass keine Interessenkonflikte bei ihnen vorliegen.

Hinweis der KLARtext-Autor:innen

Als KLARtext-Autor:innen fassen wir eine bereits vorhandene Übersichtsarbeit zusammen. Wir haben diese Übersichtsarbeit nicht selbst durchgeführt. Die Aussagen der Autor:innen der Übersichtsarbeit haben wir in allgemeinverständliche Sprache übersetzt. Wir überprüfen nicht, ob diese Aussagen wissenschaftlich korrekt sind. Wir überprüfen nicht, wie gut die Übersichtsarbeit durchgeführt wurde. Wir überprüfen auch nicht, ob die Ergebnisse der Übersichtsarbeit bereits veraltet sind.

Link zur Übersichtsarbeit: [Link wurde zu Studienzwecken entfernt]

How are adjustment to difficult circumstances and psychological well-being related in people with physical illness?

A review

KLARtexts prepare research results from psychology for the public. This KLARtext was written by staff of the Leibniz Institute of Psychology. The KLARtext summarizes the review paper titled "The Association Between Resilience and Mental Health in the Somatically Ill." The review paper was published in 2018. It was written by researchers Francesca Färber and Jenny Rosendahl of the University of Jena.

Good to know

This KLARtext summarizes a review, called a meta-analysis. Researchers who do a meta-analysis first look for the results of all the studies on a particular question (for example, "How well does a particular psychotherapy help?"). Then they summarize the results of those studies.

Why do this? A meta-analysis has two goals:

1. The meta-analysis gives an overview of all the studies that have already investigated that question. It describes who has already done research on a question (for example, who has already studied a particular type of psychotherapy).It also states the results of each study (for example, whether psychotherapy helped and how well it helped).

2. The meta-analysis also provides a value that summarizes the results of all studies. To do this, the researchers take the individual results of all the studies found and calculate an overall result from them. It is important here that the studies found on a topic are fairly similar. If the studies differ greatly, the calculated overall result of the meta-analysis becomes inaccurate. The calculated overall result provides the researchers with the answer to the question posed at the beginning (for example, "Across all studies, psychotherapy helps very well."). This overall result has much more power than the results of the individual studies.

What was the aim of the review?

Background: A physical illness is often experienced as stressful. How stressful such an illness is for mental well-being also depends on a person's characteristics. These traits encompass the ability to adapt effectively in challenging situations.

Research question: With their review, the researchers wanted to find out: How are individual adaptation to difficult circumstances and psychological well-being related in people with physical

illness?

Key message of the review

In people with physical illnesses, individual adjustment to difficult circumstances and psychological well-being are related: Those who adapt better have higher well-being.

How did the researchers proceed in the review?

What studies did the researchers look for in the review?

The researchers sought studies on the relationship between individual adaptation to difficult circumstances and psychological well-being. Participants in the studies had to be people with physical illnesses.

What studies did the researchers find for the review?

The researchers identified a total of 55 studies conducted between 2006 and 2018, and combined their results into a meta-analysis. In total, these add up to results from 15,003 participants.

What did the researchers do in the review?

In the 55 studies, the researchers looked at how adaptation to difficult circumstances and psychological well-being were related. They were interested in the magnitude and direction of this relationship: is better adjustment associated with higher or lower well-being? They also examined the influence of study characteristics on this relationship.

What did the researchers study in the review?

- Individual adaptation to difficult circumstances
- Characteristics of the studies: for example, the number of participants
- Mental well-being

What are the main findings?

- The correlation r was 0.43, which is a moderately large correlation between adjustment to difficult circumstances and psychological well-being in people with physical illness.
- The influence beta of the number of participants in a study on the correlation was -0.003. This is a very small influence. The correlation between adjustment and well-being decreased as the number of participants in a study increased.

How can the results be evaluated?

What do the results tell us?

In the review, a relationship was observed between adaptation to difficult circumstances and psychological well-being. Because of the nature of the studies that were considered, we only know that this association exists in people with physical illnesses. However, one cannot say for certain that adaptation is also the cause of well-being.

Are the results biased by limited publishing of studies?

» **What is the issue?** Unambiguous research results are easier to publish than ambiguous results. This is problematic for reviews. Namely, they cannot take unpublished results into account.

» **What does this mean for this review?** The researchers found evidence of such bias. They made an effort to account for these biases. Therefore, they assume that the association between adjustment to difficult circumstances and psychological well-being is indeed similar in magnitude to that calculated in their review.

How reliable are the results?

The researchers give pause for thought: There is no consensus in research as to whether adaptation to difficult circumstances is an innate trait. It could also be a learnable and thus trainable skill. In addition, the magnitude of the association varied widely among the studies that found it. Some studies found a larger, others a smaller correlation.

What everyday relevance do the researchers see in the review?

People with physical illness could potentially be better supported during their treatment. Professionals could assess individual adaptation to difficult circumstances early on. Those who adapt poorly to difficult circumstances could then receive special help.

What else should be noted about the review?

Who funded the review?

No information can be found in the review paper about how it was funded.

Do the researchers report their own conflicts of interest in the review?

The researchers report that they have no conflicts of interest.

Disclaimer of the KLARtext authors

As KLARtext authors, we summarize an existing review. We did not conduct this review ourselves. We have translated the statements of the authors of the review into generally understandable language. We do not check whether these statements are scientifically correct. We do not check how well the review was conducted. We also do not check whether the results of the review are already outdated.

Link to the review: [link has been removed for study purposes].

Färber and Rosendahl, New Guideline with CAMA Elements

Wie hängen die Anpassung an schwierige Umstände und das seelische Wohlbefinden bei Menschen mit körperlichen Erkrankungen zusammen?

Lebendige Evidenz in PsychOpen CAMA

KLARtexte bereiten Forschungsergebnisse aus der Psychologie für die Öffentlichkeit auf. Dieser KLARtext wurde von Mitarbeitenden des Leibniz-Instituts für Psychologie verfasst. Der KLARtext beruht auf lebendiger Evidenz aus der PsychOpen CAMA Datenbank des Leibniz-Instituts für Psychologie vom 07.06.2022. “Lebendige Evidenz” bedeutet hier, dass fortlaufend neue Forschungsergebnisse in die Metaanalyse (siehe “Gut zu wissen” weiter unten) aufgenommen werden können. Das Gesamtergebnis der Metaanalyse kann damit immer wieder an den neuesten Forschungsstand angepasst werden. Die Grundlage für die hier vorgestellte lebendige Evidenz bildet die Übersichtsarbeit mit dem Titel „The Association Between Resilience and Mental Health in the Somatically Ill“. Diese Übersichtsarbeit wurde 2018 veröffentlicht. Sie stammt von den Forschenden Francesca Färber und Jenny Rosendahl der Universität Jena.

Gut zu wissen

Die Übersichtsarbeit, auf der die lebendige Evidenz in PsychOpen CAMA beruht, ist eine Metaanalyse. Forschende, die eine Metaanalyse machen, suchen zuerst die Ergebnisse aller Studien zu einer bestimmten Frage (zum Beispiel “Wie gut hilft eine bestimmte Psychotherapie?”). Dann fassen sie die Ergebnisse dieser Studien zusammen.

Warum macht man das? Eine Metaanalyse hat zwei Ziele:

1. Die Metaanalyse gibt einen Überblick über alle Studien, die diese Frage schon untersucht haben. Sie beschreibt, wer schon zu einer Frage geforscht hat (zum Beispiel, wer schon alles diese Psychotherapie untersucht hat). Außerdem steht dort, was in den einzelnen Studien herauskam (zum Beispiel, ob und wie gut die Psychotherapie geholfen hat).

2. Die Metaanalyse liefert außerdem einen Wert, der die Ergebnisse aller Studien zusammenfasst. Dafür nehmen die Forschenden die Einzelergebnisse aller gefundenen Studien und berechnen ein Gesamtergebnis. Es ist dabei wichtig, dass die gefundenen Studien zu einem Thema ziemlich ähnlich sind. Wenn sich die Studien sehr

unterscheiden, wird das berechnete Gesamtergebnis der Metaanalyse ungenau. Das berechnete Gesamtergebnis liefert den Forschenden die Antwort auf die anfangs gestellte Frage (zum Beispiel “Über alle Studien hinweg hilft die Psychotherapie sehr gut.”). Dieses Gesamtergebnis hat eine viel höhere Aussagekraft als die Ergebnisse der einzelnen Studien.

Was war das Ziel der Übersichtsarbeit, die in PsychOpen CAMA aufgenommen wurde?

Hintergrund: Eine körperliche Erkrankung wird häufig als belastend erlebt. Wie belastend eine solche Erkrankung für das seelische Wohlbefinden ist, hängt auch von den Eigenschaften einer Person ab. Dazu gehört die Eigenschaft, sich gut an schwierige Umstände anzupassen.

Forschungsfrage: Mit ihrer Übersichtsarbeit wollten die Forschenden herausfinden: Wie hängen die individuelle Anpassung an schwierige Umstände und das seelische Wohlbefinden bei Menschen mit körperlichen Erkrankungen zusammen?

Aktuelle Kernaussage in PsychOpen CAMA

Bei Menschen mit körperlichen Erkrankungen hängen die individuelle Anpassung an schwierige Umstände und das seelische Wohlbefinden zusammen: Diejenigen, die sich besser anpassen, haben ein höheres Wohlbefinden.

Wie sind die Forschenden in der Übersichtsarbeit vorgegangen?

Welche Studien haben die Forschenden für die Übersichtsarbeit gesucht?
Die Forschenden suchten Studien zum Zusammenhang zwischen der individuellen Anpassung an schwierige Umstände und seelischem Wohlbefinden. Teilnehmende der Studien mussten Menschen mit körperlichen Erkrankungen sein.

Was haben die Forschenden in der Übersichtsarbeit gemacht?
In den in PsychOpen CAMA vorliegenden Studien schauten die Forschenden, wie die Anpassung an schwierige Umstände und das seelische Wohlbefinden zusammenhingen. Dabei interessierte sie die Größe und Richtung dieses Zusammenhangs: Geht eine bessere Anpassung mit höherem oder niedrigerem Wohlbefinden einher? Sie untersuchten auch den Einfluss von Eigenschaften der Studien auf diesen Zusammenhang.

Welche lebendige Evidenz findet sich in PsychOpen CAMA?

Welche Studien zur Forschungsfrage sind vorhanden?
Die lebendige Evidenz in PsychOpen CAMA umfasst aktuell insgesamt 55 Studien aus den Jahren 2006 bis 2018. Aus diesen Studien können derzeit insgesamt 95 Ergebnisse zu einer Metaanalyse zusammengefasst werden.

Was kann mit PsychOpen CAMA untersucht werden?
Für diesen KLARtext relevant:

- Individuelle Anpassung an schwierige Umstände
- Eigenschaften der Studien: die Anzahl der Teilnehmenden
- Seelisches Wohlbefinden

Zusätzlich können in PsychOpen CAMA weitere Vorgehensweisen zur Erfassung seelischen Wohlbefindens sowie weitere Eigenschaften der Studien (zum Beispiel, wo die Studie stattfand) verglichen werden.

Was sind die aktuellen Ergebnisse in PsychOpen CAMA?

- Die Korrelation r betrug 0.43. Das ist ein mittelgroßer Zusammenhang zwischen der Anpassung an schwierige Umstände und dem seelischen Wohlbefinden bei Menschen mit körperlicher Erkrankung.
- Der Einfluss Beta der Anzahl der Teilnehmenden einer Studie auf den Zusammenhang betrug -0.003. Dies ist ein sehr kleiner Einfluss. Der Zusammenhang zwischen der Anpassung und dem Wohlbefinden wurde kleiner, je mehr Teilnehmende eine Studie hatte.

Wie lassen sich die Ergebnisse bewerten?

Was sagen die Ergebnisse aus?

In der Übersichtsarbeit wurde ein Zusammenhang zwischen der Anpassung an schwierige Umstände und seelischem Wohlbefinden beobachtet. Wegen der Art der Studien, die berücksichtigt wurden, weiß man nur, dass es diesen Zusammenhang bei Menschen mit körperlichen Erkrankungen gibt. Man kann aber nicht sicher sagen, dass die Anpassung auch die Ursache des Wohlbefindens ist.

Sind die Ergebnisse durch eingeschränktes Veröffentlichen von Studien verzerrt?

»**Worum geht es?** Eindeutige Forschungsergebnisse lassen sich leichter veröffentlichen als uneindeutige Ergebnisse. Das ist für Übersichtsarbeiten problematisch. Sie können unveröffentlichte Ergebnisse nämlich nicht berücksichtigen.

»**Was bedeutet das für die lebendige Evidenz in PsychOpen CAMA?** In PsychOpen CAMA fanden sich keine Hinweise auf solche Verzerrungen. Es ist deshalb anzunehmen, dass der Zusammenhang zwischen der Anpassung an schwierige Umstände und dem seelischen Wohlbefinden tatsächlich ähnlich groß ist wie berechnet.

Wie zuverlässig sind die Ergebnisse?

PsychOpen CAMA testet automatisch mit einem speziellen Verfahren, ob der Zusammenhang zwischen der individuellen Anpassung an schwierige Umstände und seelischem Wohlbefinden bei Menschen mit körperlichen Erkrankungen aussagekräftig ist oder die Aussagekraft durch fragwürdige Methoden eingeschränkt ist. Dieser Test deutet darauf hin, dass der gefundene Zusammenhang aussagekräftig ist. Man kann davon ausgehen, dass dieser Zusammenhang nicht durch fragwürdige Methoden in den gefundenen Studien zustande gekommen ist.

Welchen Alltagsbezug sehen die Forschenden in der Übersichtsarbeit?

.

In ihrer Übersichtsarbeit machten die Forschenden folgende Aussage: Körperlich erkrankte Menschen könnten während ihrer Behandlung möglicherweise besser unterstützt werden. Fachpersonal könnte frühzeitig die individuelle Anpassung an schwierige Umstände einschätzen. Wer sich schlecht an schwierige Umstände anpassen kann, könnte dann besondere Hilfe bekommen. Beachten Sie, dass die lebendige Evidenz seitdem möglicherweise erweitert wurde. Diese Aussage wurde aber nicht aktualisiert.

Was ist noch zur Übersichtsarbeit zu beachten?

Wer hat die Übersichtsarbeit finanziert?
In der Übersichtsarbeit, auf der die lebendige Evidenz beruht, können keine Angaben dazu gefunden werden, wie diese finanziert wurde.

Berichten die Forschenden in der Übersichtsarbeit eigene Interessenkonflikte?
Die Forschenden berichten in der Übersichtsarbeit, auf der die lebendige Evidenz beruht, dass keine Interessenkonflikte bei ihnen vorliegen.

Hinweis der KLARtext-Autor:innen

Als KLARtext-Autor:innen fassen wir eine bereits vorhandene Übersichtsarbeit und die dazu gehörende lebendige Evidenz zusammen. Wir haben diese Übersichtsarbeit nicht selbst durchgeführt. Die Aussagen der Autor:innen der Übersichtsarbeit haben wir in allgemeinverständliche Sprache übersetzt. Außerdem geben wir den Ergebnisstand aus PsychOpen CAMA wieder. Wir überprüfen nicht, ob diese Aussagen und die Ergebnisse wissenschaftlich korrekt sind. Wir überprüfen nicht, wie gut die Übersichtsarbeit durchgeführt wurde.

Link zur lebendigen Evidenz in PsychOpen CAMA: [Link wurde zu Studienzwecken entfernt]

Link zur Übersichtsarbeit, auf der die lebendige Evidenz beruht: [Link wurde zu Studienzwecken entfernt]

How are adaptation to challenging circumstances and psychological well-being related in people with physical illness?

Living Evidence in PsychOpen CAMA

KLARtexts prepare research findings in psychology for the public. This KLARtext was written by staff of the Leibniz Institute of Psychology. The KLARtext is based on living evidence from the PsychOpen CAMA database of the Leibniz Institute for Psychology as of 07.06.2022. "Living evidence" here means that new research results can be continuously added to the meta-analysis (see "Good to know" below). The overall result of the meta-analysis can thus always be adapted to the latest state of research. The basis for the living evidence presented here is the review paper titled "The Association Between Resilience and Mental Health in the Somatically Ill." This review paper was published in 2018. It was written by researchers Francesca Färber and Jenny Rosendahl of the University of Jena.

Good to know

The review on which the living evidence in PsychOpen CAMA is based is a meta-analysis. Researchers who do a meta-analysis first look for the results of all the studies on a particular question (for example, "How well does a particular psychotherapy help?"). Then they summarize the results of those studies.

Why do this? A meta-analysis has two goals:

1. Meta-analysis provides an overview of all the studies that have already investigated this question. It describes who has already done research on a question (for example, who has already studied a particular type of psychotherapy).It also states the results of each study(for example, whether psychotherapy helped and how well it helped).

2. Meta-analysis also provides a value that summarizes the results of all the studies. To do this, researchers take the individual results of all the studies they found and calculate an overall result. It is important here that the studies found on a topic are fairly similar. If the studies are very different, the calculated overall result of the meta-analysis will be inaccurate. The calculated overall result provides the researchers with the answer to the question posed at the beginning (for example, "Across all studies, psychotherapy helps very well."). This overall result has much more power than the results of the individual studies.

What was the goal of the review that was included in PsychOpen CAMA?

Background: A physical illness is often experienced as burdensome. How burdensome such an illness is for mental well-being also depends on a person's characteristics. These traits encompass the ability to adapt effectively in challenging situations.

Research question: With their review, the researchers wanted to find out: How are individual adjustment to difficult circumstances and psychological well-being related in people with physical illness?

Current key message in PsychOpen CAMA

Individual adjustment to difficult circumstances and psychological well-being are related in people with physical illnesses: Those who adapt better have higher well-being.

How did the researchers proceed in the review?

What studies did the researchers look for in the review?

The researchers sought studies on the relationship between individual adaptation to difficult circumstances and psychological well-being. Participants in the studies had to be people with physical illnesses.

What did the researchers do in the review?

In the studies available in PsychOpen CAMA, the researchers looked at how adaptation to difficult circumstances and psychological well-being were related. They were interested in the magnitude and direction of this relationship: is better adjustment associated with higher or lower well-being? They also examined the influence of study characteristics on this relationship.

What living evidence can be found in PsychOpen CAMA?

Which studies on the research question are available?

The living evidence in PsychOpen CAMA currently includes a total of 55 studies from 2006 to 2018. From these studies, a total of 95 results can currently be combined into a meta-analysis.

What can be studied with PsychOpen CAMA?

Relevant to this KLARtext:

- Individual adjustment to difficult circumstances
- Characteristics of the studies: the number of participants
- Mental well-being

In addition, PsychOpen CAMA can compare other approaches to assessing psychological well-being and other characteristics of the studies (for example, where the study took place).

What are the current results in PsychOpen CAMA?

- The correlation r was 0.43, which is a moderately large association between adjustment to difficult circumstances and psychological well-being in people with physical illness.
- The influence beta of the number of participants in a study on the correlation was -0.003. This is a very small influence. The correlation between adjustment and well-being decreased as the number of participants in a study increased.

How can the results be evaluated?

What do the results tell us?

In the review, a relationship was observed between adaptation to difficult circumstances and psychological well-being. Because of the nature of the studies that were considered, we only know that this association exists in people with physical illnesses. However, one cannot say for certain that adaptation is also the cause of well-being.

Are the results biased by limited publishing of studies?

- **What is the issue?** Unambiguous research results are easier to publish than ambiguous results. This is problematic for reviews. Namely, they cannot take unpublished results into account.

- **What does this mean for the living evidence in PsychOpen CAMA?** No evidence of such bias was found in PsychOpen CAMA. It is therefore reasonable to assume that the association between adjustment to difficult circumstances and psychological well-being is indeed similar in magnitude to that calculated.

How reliable are the results?

PsychOpen CAMA automatically uses a special procedure to test whether the correlation between individual adaptation to difficult circumstances and psychological well-being in people with physical illnesses is meaningful or whether the meaningfulness is limited by questionable methods. This test suggests that the relationship found is meaningful. It can be assumed that this correlation did not result from questionable methods in the studies found.

What everyday relevance do the researchers see in the review paper?

In their review, the researchers made the following statement: physically ill people could possibly be better supported during their treatment. Professionals could assess individual adaptation to difficult circumstances early on. Those who adapt poorly to difficult circumstances could then receive special help. Note that the living evidence may have been expanded since then. However, this statement has not been updated.

What else should be noted about the review?

Who funded the review?

No information can be found in the review on which the living evidence is based about how it was funded.

Do the researchers report their own conflicts of interest in the review?

The researchers report in the review on which the living evidence is based that they have no conflicts of interest.

Disclaimer of the KLARtext authors

As KLARtext authors, we summarize an existing review and the associated living evidence. We did not conduct this review ourselves. We have translated the statements of the authors of the review into generally understandable language. We also report the results from PsychOpen CAMA. We do not verify whether these statements and the results are scientifically correct. We do not review how well the review was conducted.

Link to living evidence in PsychOpen CAMA: [link has been removed for study purposes].

Link to the review on which the living evidence is based: [link has been removed for study purposes]
