# Supplementary material for: Equivalent user experience and improved community augmented meta-analyses knowledge for a new version of a Plain Language Summary guideline
Source: PLoS One. 2024 May 9;19(5):e0300675. doi: 10.1371/journal.pone.0300675 (PMC11081257; doi:10.1371/journal.pone.0300675)
Supplement: S4 File — https://doi.org/10.23668/psycharchives.14219. (ZIP) [file pone.0300675.s004.zip › S4_File.html]

S4. Complete R Markdown


# S4. Complete R Markdown

#### 2024-01-23

# Preparation

## Load Dataframe

```
data <- read.csv2("data_project_917982_2022_11_15.csv",
                  na.strings = c("-66","-77","-99"),
                  encoding = "UFT-8")
View(data)
names(data)
```

```
##   [1] "lfdn"                "external_lfdn"       "tester"             
##   [4] "dispcode"            "lastpage"            "quality"            
##   [7] "duration"            "c_0001"              "p_0001"             
##  [10] "c_0002"              "c_0003"              "c_0004"             
##  [13] "v_1"                 "v_2"                 "v_3"                
##  [16] "v_4"                 "v_5"                 "v_7"                
##  [19] "v_8"                 "v_9"                 "v_10"               
##  [22] "v_11"                "v_47"                "v_48"               
##  [25] "v_49"                "v_12"                "v_14"               
##  [28] "v_16"                "v_71"                "v_17"               
##  [31] "v_18"                "v_19"                "v_20"               
##  [34] "v_21"                "v_115"               "v_116"              
##  [37] "v_117"               "v_22"                "v_23"               
##  [40] "v_24"                "v_25"                "v_26"               
##  [43] "v_120"               "v_27"                "v_28"               
##  [46] "v_29"                "v_30"                "v_31"               
##  [49] "v_121"               "v_32"                "v_33"               
##  [52] "v_34"                "v_35"                "v_36"               
##  [55] "v_122"               "v_37"                "v_38"               
##  [58] "v_39"                "v_40"                "v_41"               
##  [61] "v_123"               "v_124"               "v_42"               
##  [64] "v_43"                "v_44"                "v_45"               
##  [67] "v_46"                "v_125"               "v_72"               
##  [70] "v_73"                "v_74"                "v_75"               
##  [73] "v_76"                "v_77"                "v_79"               
##  [76] "v_81"                "v_83"                "v_126"              
##  [79] "v_127"               "v_128"               "v_129"              
##  [82] "v_130"               "v_131"               "v_132"              
##  [85] "v_133"               "v_134"               "v_135"              
##  [88] "v_136"               "v_137"               "v_138"              
##  [91] "v_139"               "v_140"               "v_141"              
##  [94] "v_142"               "v_143"               "v_144"              
##  [97] "v_145"               "v_146"               "v_147"              
## [100] "v_148"               "v_149"               "v_150"              
## [103] "v_151"               "v_152"               "v_153"              
## [106] "v_154"               "v_155"               "v_156"              
## [109] "v_157"               "v_158"               "v_159"              
## [112] "v_160"               "v_161"               "v_162"              
## [115] "v_163"               "v_164"               "v_50"               
## [118] "v_51"                "v_52"                "v_53"               
## [121] "v_54"                "v_165"               "v_166"              
## [124] "v_167"               "v_55"                "v_56"               
## [127] "v_57"                "v_58"                "v_401"              
## [130] "v_91"                "v_92"                "v_93"               
## [133] "v_94"                "v_95"                "v_96"               
## [136] "v_98"                "v_100"               "v_102"              
## [139] "v_235"               "v_236"               "v_237"              
## [142] "v_238"               "v_239"               "v_240"              
## [145] "v_241"               "v_242"               "v_243"              
## [148] "v_244"               "v_245"               "v_246"              
## [151] "v_247"               "v_248"               "v_249"              
## [154] "v_250"               "v_251"               "v_252"              
## [157] "v_253"               "v_254"               "v_255"              
## [160] "v_256"               "v_257"               "v_258"              
## [163] "v_259"               "v_313"               "v_314"              
## [166] "v_315"               "v_316"               "v_317"              
## [169] "v_323"               "v_324"               "v_325"              
## [172] "v_326"               "v_327"               "v_328"              
## [175] "v_329"               "v_330"               "v_331"              
## [178] "v_332"               "v_333"               "v_334"              
## [181] "v_335"               "v_336"               "v_337"              
## [184] "v_338"               "v_339"               "v_340"              
## [187] "v_341"               "v_342"               "v_343"              
## [190] "v_344"               "v_345"               "v_103"              
## [193] "v_104"               "v_105"               "v_106"              
## [196] "v_107"               "v_108"               "v_110"              
## [199] "v_112"               "v_114"               "v_274"              
## [202] "v_275"               "v_276"               "v_277"              
## [205] "v_278"               "v_279"               "v_280"              
## [208] "v_281"               "v_282"               "v_283"              
## [211] "v_284"               "v_285"               "v_286"              
## [214] "v_287"               "v_288"               "v_289"              
## [217] "v_290"               "v_291"               "v_292"              
## [220] "v_293"               "v_294"               "v_295"              
## [223] "v_296"               "v_297"               "v_298"              
## [226] "v_299"               "v_300"               "v_301"              
## [229] "v_302"               "v_303"               "v_304"              
## [232] "v_305"               "v_306"               "v_307"              
## [235] "v_308"               "v_309"               "v_310"              
## [238] "v_402"               "v_360"               "v_361"              
## [241] "v_362"               "v_363"               "v_364"              
## [244] "v_365"               "v_366"               "v_367"              
## [247] "v_368"               "v_369"               "v_370"              
## [250] "v_371"               "v_372"               "v_373"              
## [253] "v_374"               "v_375"               "v_376"              
## [256] "v_377"               "v_378"               "v_379"              
## [259] "v_380"               "v_381"               "v_382"              
## [262] "v_383"               "v_384"               "v_385"              
## [265] "v_386"               "v_387"               "v_388"              
## [268] "browser"             "referer"             "device_type"        
## [271] "quota"               "quota_assignment"    "quota_rejected_id"  
## [274] "page_history"        "hflip"               "vflip"              
## [277] "output_mode"         "javascript"          "flash"              
## [280] "session_id"          "language"            "cleaned"            
## [283] "ats"                 "datetime"            "date_of_last_access"
## [286] "date_of_first_mail"  "rts6018385"          "rts6018739"         
## [289] "rts6018818"          "rts6019080"          "rts6019089"         
## [292] "rts6021451"          "rts6021455"          "rts6023513"         
## [295] "rts6023515"          "rts6023627"          "rts6023655"         
## [298] "rts6023657"          "rts6023660"          "rts6023667"         
## [301] "rts6023676"          "rts6023679"          "rts6033975"
```

```
str(data)
```

```
## 'data.frame':    6706 obs. of  303 variables:
##  $ lfdn               : int  94 95 98 99 100 101 107 109 93 103 ...
##  $ external_lfdn      : int  0 0 0 0 0 0 0 0 0 0 ...
##  $ tester             : int  0 0 0 0 0 0 0 0 0 0 ...
##  $ dispcode           : int  37 37 37 37 37 37 37 37 31 22 ...
##  $ lastpage           : int  6018729 6018729 6018729 6018729 6018729 6018729 6018729 6018729 6018381 6023627 ...
##  $ quality            : logi  NA NA NA NA NA NA ...
##  $ duration           : int  23 57 19 25 23 37 30 38 779 68 ...
##  $ c_0001             : int  NA NA NA NA NA NA NA NA 3 2 ...
##  $ p_0001             : num  2.26e+14 2.26e+14 2.26e+14 2.26e+14 2.26e+14 ...
##  $ c_0002             : int  NA NA NA NA NA NA NA NA 3 2 ...
##  $ c_0003             : int  NA NA NA NA NA NA NA NA 1 2 ...
##  $ c_0004             : int  NA NA NA NA NA NA NA NA 2 2 ...
##  $ v_1                : int  2 1 2 1 2 1 1 2 2 2 ...
##  $ v_2                : int  48 19 55 55 33 44 64 43 31 42 ...
##  $ v_3                : int  1 1 2 2 1 1 3 3 2 2 ...
##  $ v_4                : int  1 1 1 1 1 1 1 1 1 1 ...
##  $ v_5                : int  2 2 2 2 2 2 2 2 2 2 ...
##  $ v_7                : int  1 3 1 2 1 1 3 2 5 6 ...
##  $ v_8                : int  NA NA NA NA NA NA NA NA 1 1 ...
##  $ v_9                : chr  NA NA NA NA ...
##  $ v_10               : int  NA NA NA NA NA NA NA NA 4 NA ...
##  $ v_11               : int  NA NA NA NA NA NA NA NA 6 NA ...
##  $ v_47               : int  NA NA NA NA NA NA NA NA 3 NA ...
##  $ v_48               : int  NA NA NA NA NA NA NA NA 6 NA ...
##  $ v_49               : int  NA NA NA NA NA NA NA NA 6 NA ...
##  $ v_12               : int  NA NA NA NA NA NA NA NA 2 NA ...
##  $ v_14               : int  NA NA NA NA NA NA NA NA 1 NA ...
##  $ v_16               : int  NA NA NA NA NA NA NA NA 3 NA ...
##  $ v_71               : int  NA NA NA NA NA NA NA NA 3 NA ...
##  $ v_17               : int  NA NA NA NA NA NA NA NA 1 NA ...
##  $ v_18               : int  NA NA NA NA NA NA NA NA 1 NA ...
##  $ v_19               : int  NA NA NA NA NA NA NA NA 2 NA ...
##  $ v_20               : int  NA NA NA NA NA NA NA NA 2 NA ...
##  $ v_21               : int  NA NA NA NA NA NA NA NA 1 NA ...
##  $ v_115              : int  NA NA NA NA NA NA NA NA 1 NA ...
##  $ v_116              : int  NA NA NA NA NA NA NA NA 1 NA ...
##  $ v_117              : int  NA NA NA NA NA NA NA NA 1 NA ...
##  $ v_22               : int  NA NA NA NA NA NA NA NA 3 NA ...
##  $ v_23               : int  NA NA NA NA NA NA NA NA 1 NA ...
##  $ v_24               : int  NA NA NA NA NA NA NA NA 1 NA ...
##  $ v_25               : int  NA NA NA NA NA NA NA NA 1 NA ...
##  $ v_26               : int  NA NA NA NA NA NA NA NA 1 NA ...
##  $ v_120              : int  NA NA NA NA NA NA NA NA 1 NA ...
##  $ v_27               : int  NA NA NA NA NA NA NA NA 2 NA ...
##  $ v_28               : int  NA NA NA NA NA NA NA NA 2 NA ...
##  $ v_29               : int  NA NA NA NA NA NA NA NA 2 NA ...
##  $ v_30               : int  NA NA NA NA NA NA NA NA 2 NA ...
##  $ v_31               : int  NA NA NA NA NA NA NA NA 1 NA ...
##  $ v_121              : int  NA NA NA NA NA NA NA NA 1 NA ...
##  $ v_32               : int  NA NA NA NA NA NA NA NA 2 NA ...
##  $ v_33               : int  NA NA NA NA NA NA NA NA 2 NA ...
##  $ v_34               : int  NA NA NA NA NA NA NA NA 2 NA ...
##  $ v_35               : int  NA NA NA NA NA NA NA NA 2 NA ...
##  $ v_36               : int  NA NA NA NA NA NA NA NA 2 NA ...
##  $ v_122              : int  NA NA NA NA NA NA NA NA 1 NA ...
##  $ v_37               : int  NA NA NA NA NA NA NA NA 2 NA ...
##  $ v_38               : int  NA NA NA NA NA NA NA NA 0 NA ...
##  $ v_39               : int  NA NA NA NA NA NA NA NA 2 NA ...
##  $ v_40               : int  NA NA NA NA NA NA NA NA 2 NA ...
##  $ v_41               : int  NA NA NA NA NA NA NA NA 1 NA ...
##  $ v_123              : int  NA NA NA NA NA NA NA NA 1 NA ...
##  $ v_124              : int  NA NA NA NA NA NA NA NA 2 NA ...
##  $ v_42               : int  NA NA NA NA NA NA NA NA 3 NA ...
##  $ v_43               : int  NA NA NA NA NA NA NA NA 1 NA ...
##  $ v_44               : int  NA NA NA NA NA NA NA NA 3 NA ...
##  $ v_45               : int  NA NA NA NA NA NA NA NA 1 NA ...
##  $ v_46               : int  NA NA NA NA NA NA NA NA 3 NA ...
##  $ v_125              : int  NA NA NA NA NA NA NA NA 3 NA ...
##  $ v_72               : int  NA NA NA NA NA NA NA NA NA 6 ...
##  $ v_73               : int  NA NA NA NA NA NA NA NA NA 7 ...
##  $ v_74               : int  NA NA NA NA NA NA NA NA NA 7 ...
##  $ v_75               : int  NA NA NA NA NA NA NA NA NA 7 ...
##  $ v_76               : int  NA NA NA NA NA NA NA NA NA 7 ...
##  $ v_77               : int  NA NA NA NA NA NA NA NA NA 4 ...
##  $ v_79               : int  NA NA NA NA NA NA NA NA NA 1 ...
##  $ v_81               : int  NA NA NA NA NA NA NA NA NA 1 ...
##  $ v_83               : int  NA NA NA NA NA NA NA NA NA 1 ...
##  $ v_126              : int  NA NA NA NA NA NA NA NA NA NA ...
##  $ v_127              : int  NA NA NA NA NA NA NA NA NA NA ...
##  $ v_128              : int  NA NA NA NA NA NA NA NA NA NA ...
##  $ v_129              : int  NA NA NA NA NA NA NA NA NA NA ...
##  $ v_130              : int  NA NA NA NA NA NA NA NA NA NA ...
##  $ v_131              : int  NA NA NA NA NA NA NA NA NA NA ...
##  $ v_132              : int  NA NA NA NA NA NA NA NA NA NA ...
##  $ v_133              : int  NA NA NA NA NA NA NA NA NA NA ...
##  $ v_134              : int  NA NA NA NA NA NA NA NA NA NA ...
##  $ v_135              : int  NA NA NA NA NA NA NA NA NA NA ...
##  $ v_136              : int  NA NA NA NA NA NA NA NA NA NA ...
##  $ v_137              : int  NA NA NA NA NA NA NA NA NA NA ...
##  $ v_138              : int  NA NA NA NA NA NA NA NA NA NA ...
##  $ v_139              : int  NA NA NA NA NA NA NA NA NA NA ...
##  $ v_140              : int  NA NA NA NA NA NA NA NA NA NA ...
##  $ v_141              : int  NA NA NA NA NA NA NA NA NA NA ...
##  $ v_142              : int  NA NA NA NA NA NA NA NA NA NA ...
##  $ v_143              : int  NA NA NA NA NA NA NA NA NA NA ...
##  $ v_144              : int  NA NA NA NA NA NA NA NA NA NA ...
##  $ v_145              : int  NA NA NA NA NA NA NA NA NA NA ...
##  $ v_146              : int  NA NA NA NA NA NA NA NA NA NA ...
##  $ v_147              : int  NA NA NA NA NA NA NA NA NA NA ...
##   [list output truncated]
```

```
nrow(data)
```

```
## [1] 6706
```

## Check Dispcodes

```
table(data$dispcode)
```

```
## 
##   20   22   31   32   36   37 
##   45 1039 1754  288 1474 2106
```

```
# 20 = Not started yet --> 45
# 22 = Interrupted --> 1039
# 37,38,39,40 = Screenout --> 2106
# 35,36,41 = Quota full --> 1474
# 31,32,33,34 = Finished --> 2042

# Check for multiple participations
x <- table(data$p_0001[data$dispcode==31|
                         data$dispcode==32])

code <- dimnames(x)[[1]]
code <- code[x>1]
code
```

```
## [1] "225951288721641"
```

```
#Case 225951288721641 participated twice. Exclude second participation.

data$lfdn[data$p_0001 == "225951288721641"]
```

```
## [1]   NA 1732 3192
```

```
data$datetime[data$p_0001 == "225951288721641"]
```

```
## [1] NA                    "2022-10-26 07:45:35" "2022-10-27 17:38:04"
```

```
data <- data[!data$lfdn == 3192,]
```

## Recode Data

```
data$lfdn <- rank(rnorm(nrow(data)))
names(data)[1] <- "id"

data$id <- factor(data$id,levels = c(1:6705))

str(data)
```

```
## 'data.frame':    6705 obs. of  303 variables:
##  $ id                 : Factor w/ 6705 levels "1","2","3","4",..: 759 52 4690 3401 6194 1195 5513 1286 4692 193 ...
##  $ external_lfdn      : int  0 0 0 0 0 0 0 0 0 0 ...
##  $ tester             : int  0 0 0 0 0 0 0 0 0 0 ...
##  $ dispcode           : int  37 37 37 37 37 37 37 37 31 22 ...
##  $ lastpage           : int  6018729 6018729 6018729 6018729 6018729 6018729 6018729 6018729 6018381 6023627 ...
##  $ quality            : logi  NA NA NA NA NA NA ...
##  $ duration           : int  23 57 19 25 23 37 30 38 779 68 ...
##  $ c_0001             : int  NA NA NA NA NA NA NA NA 3 2 ...
##  $ p_0001             : num  2.26e+14 2.26e+14 2.26e+14 2.26e+14 2.26e+14 ...
##  $ c_0002             : int  NA NA NA NA NA NA NA NA 3 2 ...
##  $ c_0003             : int  NA NA NA NA NA NA NA NA 1 2 ...
##  $ c_0004             : int  NA NA NA NA NA NA NA NA 2 2 ...
##  $ v_1                : int  2 1 2 1 2 1 1 2 2 2 ...
##  $ v_2                : int  48 19 55 55 33 44 64 43 31 42 ...
##  $ v_3                : int  1 1 2 2 1 1 3 3 2 2 ...
##  $ v_4                : int  1 1 1 1 1 1 1 1 1 1 ...
##  $ v_5                : int  2 2 2 2 2 2 2 2 2 2 ...
##  $ v_7                : int  1 3 1 2 1 1 3 2 5 6 ...
##  $ v_8                : int  NA NA NA NA NA NA NA NA 1 1 ...
##  $ v_9                : chr  NA NA NA NA ...
##  $ v_10               : int  NA NA NA NA NA NA NA NA 4 NA ...
##  $ v_11               : int  NA NA NA NA NA NA NA NA 6 NA ...
##  $ v_47               : int  NA NA NA NA NA NA NA NA 3 NA ...
##  $ v_48               : int  NA NA NA NA NA NA NA NA 6 NA ...
##  $ v_49               : int  NA NA NA NA NA NA NA NA 6 NA ...
##  $ v_12               : int  NA NA NA NA NA NA NA NA 2 NA ...
##  $ v_14               : int  NA NA NA NA NA NA NA NA 1 NA ...
##  $ v_16               : int  NA NA NA NA NA NA NA NA 3 NA ...
##  $ v_71               : int  NA NA NA NA NA NA NA NA 3 NA ...
##  $ v_17               : int  NA NA NA NA NA NA NA NA 1 NA ...
##  $ v_18               : int  NA NA NA NA NA NA NA NA 1 NA ...
##  $ v_19               : int  NA NA NA NA NA NA NA NA 2 NA ...
##  $ v_20               : int  NA NA NA NA NA NA NA NA 2 NA ...
##  $ v_21               : int  NA NA NA NA NA NA NA NA 1 NA ...
##  $ v_115              : int  NA NA NA NA NA NA NA NA 1 NA ...
##  $ v_116              : int  NA NA NA NA NA NA NA NA 1 NA ...
##  $ v_117              : int  NA NA NA NA NA NA NA NA 1 NA ...
##  $ v_22               : int  NA NA NA NA NA NA NA NA 3 NA ...
##  $ v_23               : int  NA NA NA NA NA NA NA NA 1 NA ...
##  $ v_24               : int  NA NA NA NA NA NA NA NA 1 NA ...
##  $ v_25               : int  NA NA NA NA NA NA NA NA 1 NA ...
##  $ v_26               : int  NA NA NA NA NA NA NA NA 1 NA ...
##  $ v_120              : int  NA NA NA NA NA NA NA NA 1 NA ...
##  $ v_27               : int  NA NA NA NA NA NA NA NA 2 NA ...
##  $ v_28               : int  NA NA NA NA NA NA NA NA 2 NA ...
##  $ v_29               : int  NA NA NA NA NA NA NA NA 2 NA ...
##  $ v_30               : int  NA NA NA NA NA NA NA NA 2 NA ...
##  $ v_31               : int  NA NA NA NA NA NA NA NA 1 NA ...
##  $ v_121              : int  NA NA NA NA NA NA NA NA 1 NA ...
##  $ v_32               : int  NA NA NA NA NA NA NA NA 2 NA ...
##  $ v_33               : int  NA NA NA NA NA NA NA NA 2 NA ...
##  $ v_34               : int  NA NA NA NA NA NA NA NA 2 NA ...
##  $ v_35               : int  NA NA NA NA NA NA NA NA 2 NA ...
##  $ v_36               : int  NA NA NA NA NA NA NA NA 2 NA ...
##  $ v_122              : int  NA NA NA NA NA NA NA NA 1 NA ...
##  $ v_37               : int  NA NA NA NA NA NA NA NA 2 NA ...
##  $ v_38               : int  NA NA NA NA NA NA NA NA 0 NA ...
##  $ v_39               : int  NA NA NA NA NA NA NA NA 2 NA ...
##  $ v_40               : int  NA NA NA NA NA NA NA NA 2 NA ...
##  $ v_41               : int  NA NA NA NA NA NA NA NA 1 NA ...
##  $ v_123              : int  NA NA NA NA NA NA NA NA 1 NA ...
##  $ v_124              : int  NA NA NA NA NA NA NA NA 2 NA ...
##  $ v_42               : int  NA NA NA NA NA NA NA NA 3 NA ...
##  $ v_43               : int  NA NA NA NA NA NA NA NA 1 NA ...
##  $ v_44               : int  NA NA NA NA NA NA NA NA 3 NA ...
##  $ v_45               : int  NA NA NA NA NA NA NA NA 1 NA ...
##  $ v_46               : int  NA NA NA NA NA NA NA NA 3 NA ...
##  $ v_125              : int  NA NA NA NA NA NA NA NA 3 NA ...
##  $ v_72               : int  NA NA NA NA NA NA NA NA NA 6 ...
##  $ v_73               : int  NA NA NA NA NA NA NA NA NA 7 ...
##  $ v_74               : int  NA NA NA NA NA NA NA NA NA 7 ...
##  $ v_75               : int  NA NA NA NA NA NA NA NA NA 7 ...
##  $ v_76               : int  NA NA NA NA NA NA NA NA NA 7 ...
##  $ v_77               : int  NA NA NA NA NA NA NA NA NA 4 ...
##  $ v_79               : int  NA NA NA NA NA NA NA NA NA 1 ...
##  $ v_81               : int  NA NA NA NA NA NA NA NA NA 1 ...
##  $ v_83               : int  NA NA NA NA NA NA NA NA NA 1 ...
##  $ v_126              : int  NA NA NA NA NA NA NA NA NA NA ...
##  $ v_127              : int  NA NA NA NA NA NA NA NA NA NA ...
##  $ v_128              : int  NA NA NA NA NA NA NA NA NA NA ...
##  $ v_129              : int  NA NA NA NA NA NA NA NA NA NA ...
##  $ v_130              : int  NA NA NA NA NA NA NA NA NA NA ...
##  $ v_131              : int  NA NA NA NA NA NA NA NA NA NA ...
##  $ v_132              : int  NA NA NA NA NA NA NA NA NA NA ...
##  $ v_133              : int  NA NA NA NA NA NA NA NA NA NA ...
##  $ v_134              : int  NA NA NA NA NA NA NA NA NA NA ...
##  $ v_135              : int  NA NA NA NA NA NA NA NA NA NA ...
##  $ v_136              : int  NA NA NA NA NA NA NA NA NA NA ...
##  $ v_137              : int  NA NA NA NA NA NA NA NA NA NA ...
##  $ v_138              : int  NA NA NA NA NA NA NA NA NA NA ...
##  $ v_139              : int  NA NA NA NA NA NA NA NA NA NA ...
##  $ v_140              : int  NA NA NA NA NA NA NA NA NA NA ...
##  $ v_141              : int  NA NA NA NA NA NA NA NA NA NA ...
##  $ v_142              : int  NA NA NA NA NA NA NA NA NA NA ...
##  $ v_143              : int  NA NA NA NA NA NA NA NA NA NA ...
##  $ v_144              : int  NA NA NA NA NA NA NA NA NA NA ...
##  $ v_145              : int  NA NA NA NA NA NA NA NA NA NA ...
##  $ v_146              : int  NA NA NA NA NA NA NA NA NA NA ...
##  $ v_147              : int  NA NA NA NA NA NA NA NA NA NA ...
##   [list output truncated]
```

```
data <- rename(data, condition = c_0001, text_order = c_0003,
               METI_target = c_0004)

data$condition <- factor(data$condition)

# Text presented first, 1 = Barth et al., 2 = Faerber et al.
data$text_order <- factor(data$text_order, levels = c(1,2), labels = 
                            c("Barth", "Faerber"))

data$METI_text <- ifelse(data$text_order == "Barth","Faerber","Barth")
data$METI_text <- factor(data$METI_text, levels = c("Barth","Faerber"))

data$summary1 <- data$text_order
data$summary2 <- data$METI_text

data$METI_target <- factor (data$METI_target, levels = c(1,2), labels =
                              c("Study Authors","Summary Authors"))

data <- rename(data, s_sex = v_1, s_age = v_2,
               s_school = v_3, s_german = v_4,
               s_psychology = v_5, s_interest = v_7,
               s_contact = v_8, s_field = v_9)

data$s_sex <- factor(data$s_sex, levels = c (1,2), labels = c("female","male"))
data$s_school <- factor(data$s_school, levels = c(1,2,3), 
                        labels = c("Haupt","Real","Abi"))

data$quota[data$quota == 0] <- NA
data$quota <- factor(data$quota)
```

### Create Factor for Experimental Condition

```
data$version <- case_when(data$condition == 1 ~1,
                          data$condition == 2 ~1,
                          data$condition == 3 ~1,
                          data$condition == 4 ~1,
                          data$condition == 5 ~1,
                          data$condition == 6 ~0)

data$version <- factor(data$version, levels = c(0,1),
                       labels = c("old guideline","new guideline"))
summary(data$version)
```

```
## old guideline new guideline          NA's 
##           498          2492          3715
```

```
data$causality <- case_when(data$condition == 1 ~0,
                            data$condition == 2 ~0,
                            data$condition == 3 ~1,
                            data$condition == 4 ~1,
                            data$condition == 5 ~1,
                            data$condition == 6 ~0)
data$causality <- factor(data$causality, levels = c(0,1),
                         labels = c("no causality statement",
                                    "causality statement"))
summary(data$causality)
```

```
## no causality statement    causality statement                   NA's 
##                   1496                   1494                   3715
```

```
data$disclaimer <- case_when(data$condition == 1 ~0,
                             data$condition == 2 ~1,
                             data$condition == 3 ~0,
                             data$condition == 4 ~1,
                             data$condition == 5 ~1,
                             data$condition == 6 ~0)
data$disclaimer <- factor(data$disclaimer, levels = c(0,1),
                         labels = c("no disclaimer",
                                    "disclaimer"))
summary(data$disclaimer)
```

```
## no disclaimer    disclaimer          NA's 
##          1495          1495          3715
```

```
data$CAMA <- case_when(data$condition == 1 ~0,
                             data$condition == 2 ~0,
                             data$condition == 3 ~0,
                             data$condition == 4 ~0,
                             data$condition == 5 ~1,
                             data$condition == 6 ~0)
data$CAMA <- factor(data$CAMA, levels = c(0,1),
                          labels = c("no CAMA PLS",
                                     "CAMA PLS"))
summary(data$disclaimer)
```

```
## no disclaimer    disclaimer          NA's 
##          1495          1495          3715
```

### Drop Unneeded Dispcodes

```
data <- data[data$dispcode == 22|data$dispcode == 31|data$dispcode == 32,]
length(unique(data$p_0001
              [data$dispcode==22|data$dispcode==31|data$dispcode==32]))
```

```
## [1] 3001
```

## Dropout Analyses

### By Condition

```
data$dropout <- data$dispcode == 22
data$dropout <- factor(data$dropout, c("FALSE","TRUE"),
                       labels = c("No Dropout", "Dropout"))

table(data$dropout, data$condition)
```

```
##             
##                1   2   3   4   5   6
##   No Dropout 334 345 336 341 328 357
##   Dropout    165 154 162 156 170 141
```

```
table(data$dropout,data$condition)[1,]/colSums(table(
  data$dropout,data$condition))*100
```

```
##        1        2        3        4        5        6 
## 66.93387 69.13828 67.46988 68.61167 65.86345 71.68675
```

```
chisq.test(data$dropout, data$condition)
```

```
## 
##  Pearson's Chi-squared test
## 
## data:  data$dropout and data$condition
## X-squared = 4.775, df = 5, p-value = 0.444
```

### By Quota

```
table(data$dropout, data$quota)
```

```
##             
##                1   2   3   4   5   6   7   8   9  10  11  12
##   No Dropout 169 171 174 168 171 172 168 167 164 170 172 175
##   Dropout     88 112 131  93 124 143  33  44  36  63  90  74
```

```
table(data$dropout, data$quota)[1,]/colSums(table(
  data$dropout, data$quota))*100
```

```
##        1        2        3        4        5        6        7        8 
## 65.75875 60.42403 57.04918 64.36782 57.96610 54.60317 83.58209 79.14692 
##        9       10       11       12 
## 82.00000 72.96137 65.64885 70.28112
```

```
chisq.test(data$dropout,data$quota)
```

```
## 
##  Pearson's Chi-squared test
## 
## data:  data$dropout and data$quota
## X-squared = 116.16, df = 11, p-value < 2.2e-16
```

### By Participants’ Gender

```
table(data$dropout, data$s_sex)
```

```
##             
##              female male
##   No Dropout   1028 1013
##   Dropout       587  446
```

```
table(data$dropout, data$s_sex)[1,]/colSums(table(data$dropout, data$s_sex))*100
```

```
##   female     male 
## 63.65325 69.43112
```

```
chisq.test(data$dropout,data$s_sex)
```

```
## 
##  Pearson's Chi-squared test with Yates' continuity correction
## 
## data:  data$dropout and data$s_sex
## X-squared = 11.211, df = 1, p-value = 0.0008129
```

### By Participants’ Age

```
# Set single age value of 744 as NA
data$s_age[data$s_age == 744] <- NA

dropout_age <- glm(data$dropout ~ data$s_age, data = data, family = "binomial",
                   na.action = na.omit)
summary(dropout_age)
```

```
## 
## Call:
## glm(formula = data$dropout ~ data$s_age, family = "binomial", 
##     data = data, na.action = na.omit)
## 
## Coefficients:
##              Estimate Std. Error z value Pr(>|z|)    
## (Intercept) -1.992992   0.130678  -15.25   <2e-16 ***
## data$s_age   0.027108   0.002514   10.78   <2e-16 ***
## ---
## Signif. codes:  0 '***' 0.001 '**' 0.01 '*' 0.05 '.' 0.1 ' ' 1
## 
## (Dispersion parameter for binomial family taken to be 1)
## 
##     Null deviance: 3932.6  on 3076  degrees of freedom
## Residual deviance: 3810.8  on 3075  degrees of freedom
##   (3 Beobachtungen als fehlend gelöscht)
## AIC: 3814.8
## 
## Number of Fisher Scoring iterations: 4
```

```
exp(dropout_age$coefficients)
```

```
## (Intercept)  data$s_age 
##    0.136287    1.027478
```

### By Participants’ Educational Background

```
table(data$dropout, data$s_school)
```

```
##             
##              Haupt Real Abi
##   No Dropout   685  681 675
##   Dropout      387  373 277
```

```
table(data$dropout, data$s_school)[1,]/colSums(
  table(data$dropout, data$s_school))*100
```

```
##    Haupt     Real      Abi 
## 63.89925 64.61101 70.90336
```

```
chisq.test(data$dropout,data$s_school)
```

```
## 
##  Pearson's Chi-squared test
## 
## data:  data$dropout and data$s_school
## X-squared = 13.142, df = 2, p-value = 0.001401
```

```
dropout_edu <- glm(data$dropout ~ data$s_school, data = data, family = "binomial",
                   na.action = na.omit)
summary(dropout_edu)
```

```
## 
## Call:
## glm(formula = data$dropout ~ data$s_school, family = "binomial", 
##     data = data, na.action = na.omit)
## 
## Coefficients:
##                   Estimate Std. Error z value Pr(>|z|)    
## (Intercept)       -0.57099    0.06359  -8.979  < 2e-16 ***
## data$s_schoolReal -0.03099    0.09052  -0.342 0.732076    
## data$s_schoolAbi  -0.31970    0.09558  -3.345 0.000823 ***
## ---
## Signif. codes:  0 '***' 0.001 '**' 0.01 '*' 0.05 '.' 0.1 ' ' 1
## 
## (Dispersion parameter for binomial family taken to be 1)
## 
##     Null deviance: 3933.5  on 3077  degrees of freedom
## Residual deviance: 3920.1  on 3075  degrees of freedom
##   (2 Beobachtungen als fehlend gelöscht)
## AIC: 3926.1
## 
## Number of Fisher Scoring iterations: 4
```

```
exp(dropout_edu$coefficients)
```

```
##       (Intercept) data$s_schoolReal  data$s_schoolAbi 
##         0.5649635         0.9694855         0.7263662
```

### Dropout Regression Analyses

```
dropout_logistic_1 <- glm(formula =  dropout ~ condition + quota, data = data, 
                        family = "binomial", na.action = na.omit)
summary(dropout_logistic_1)
```

```
## 
## Call:
## glm(formula = dropout ~ condition + quota, family = "binomial", 
##     data = data, na.action = na.omit)
## 
## Coefficients:
##              Estimate Std. Error z value Pr(>|z|)    
## (Intercept) -0.653151   0.163055  -4.006 6.18e-05 ***
## condition2  -0.140337   0.138830  -1.011 0.312085    
## condition3  -0.048597   0.138044  -0.352 0.724807    
## condition4  -0.122653   0.138728  -0.884 0.376629    
## condition5   0.007591   0.137135   0.055 0.955854    
## condition6  -0.257050   0.140745  -1.826 0.067798 .  
## quota2       0.220186   0.185468   1.187 0.235151    
## quota3       0.352223   0.180902   1.947 0.051530 .  
## quota4       0.136894   0.188245   0.727 0.467096    
## quota5       0.342825   0.182117   1.882 0.059776 .  
## quota6       0.487074   0.178462   2.729 0.006347 ** 
## quota7      -0.947738   0.238435  -3.975 7.04e-05 ***
## quota8      -0.825807   0.230417  -3.584 0.000338 ***
## quota9      -0.882218   0.236521  -3.730 0.000191 ***
## quota10     -0.334860   0.204088  -1.641 0.100846    
## quota11      0.035250   0.190172   0.185 0.852947    
## quota12     -0.162174   0.195749  -0.828 0.407400    
## ---
## Signif. codes:  0 '***' 0.001 '**' 0.01 '*' 0.05 '.' 0.1 ' ' 1
## 
## (Dispersion parameter for binomial family taken to be 1)
## 
##     Null deviance: 3734.5  on 2988  degrees of freedom
## Residual deviance: 3609.0  on 2972  degrees of freedom
##   (91 Beobachtungen als fehlend gelöscht)
## AIC: 3643
## 
## Number of Fisher Scoring iterations: 4
```

```
exp(dropout_logistic_1$coefficients)
```

```
## (Intercept)  condition2  condition3  condition4  condition5  condition6 
##   0.5204033   0.8690651   0.9525646   0.8845708   1.0076203   0.7733299 
##      quota2      quota3      quota4      quota5      quota6      quota7 
##   1.2463090   1.4222263   1.1467070   1.4089220   1.6275464   0.3876166 
##      quota8      quota9     quota10     quota11     quota12 
##   0.4378814   0.4138641   0.7154382   1.0358787   0.8502934
```

```
dropout_logistic_2 <- glm(formula =  dropout ~ s_sex + s_school + s_age,
                          data = data, family = "binomial", na.action = na.omit)
summary(dropout_logistic_2)
```

```
## 
## Call:
## glm(formula = dropout ~ s_sex + s_school + s_age, family = "binomial", 
##     data = data, na.action = na.omit)
## 
## Coefficients:
##               Estimate Std. Error z value Pr(>|z|)    
## (Intercept)  -1.828571   0.147292 -12.415  < 2e-16 ***
## s_sexmale    -0.346953   0.079185  -4.382 1.18e-05 ***
## s_schoolReal  0.031970   0.092998   0.344   0.7310    
## s_schoolAbi  -0.222797   0.098232  -2.268   0.0233 *  
## s_age         0.028037   0.002556  10.971  < 2e-16 ***
## ---
## Signif. codes:  0 '***' 0.001 '**' 0.01 '*' 0.05 '.' 0.1 ' ' 1
## 
## (Dispersion parameter for binomial family taken to be 1)
## 
##     Null deviance: 3919.6  on 3070  degrees of freedom
## Residual deviance: 3769.0  on 3066  degrees of freedom
##   (9 Beobachtungen als fehlend gelöscht)
## AIC: 3779
## 
## Number of Fisher Scoring iterations: 4
```

```
exp(dropout_logistic_2$coefficients)
```

```
##  (Intercept)    s_sexmale s_schoolReal  s_schoolAbi        s_age 
##    0.1606429    0.7068385    1.0324869    0.8002773    1.0284335
```

## Merging Variables

### Check for NAs in Demographic Variables

```
sum(is.na(data$s_sex))
```

```
## [1] 6
```

```
sum(is.na(data$s_age))
```

```
## [1] 3
```

```
sum(is.na(data$s_school))
```

```
## [1] 2
```

```
sum(is.na(data$s_german))
```

```
## [1] 0
```

```
sum(is.na(data$s_psychology))
```

```
## [1] 0
```

```
sum(is.na(data$s_interest))
```

```
## [1] 0
```

```
sum(is.na(data$s_contact))
```

```
## [1] 20
```

```
sum(is.na(data$s_field))
```

```
## [1] 2466
```

### Merge T1 and T2 Variables

```
data$v_10[data$v_10 == 0] <- NA
data$v_72[data$v_72 == 0] <- NA
data$v_91[data$v_91 == 0] <- NA
data$v_103[data$v_103 == 0] <- NA
data$v_11[data$v_11 == 0] <- NA
data$v_73[data$v_73 == 0] <- NA
data$v_92[data$v_92 == 0] <- NA
data$v_104[data$v_104 == 0] <- NA
data$v_47[data$v_47 == 0] <- NA
data$v_74[data$v_74 == 0] <- NA
data$v_93[data$v_93 == 0] <- NA
data$v_105[data$v_105 == 0] <- NA
data$v_48[data$v_48 == 0] <- NA
data$v_75[data$v_75 == 0] <- NA
data$v_94[data$v_94 == 0] <- NA
data$v_106[data$v_106 == 0] <- NA
data$v_49[data$v_49 == 0] <- NA
data$v_76[data$v_76 == 0] <- NA
data$v_95[data$v_95 == 0] <- NA
data$v_107[data$v_107 == 0] <- NA
data$v_12[data$v_12 == 0] <- NA
data$v_77[data$v_77 == 0] <- NA
data$v_96[data$v_96 == 0] <- NA
data$v_108[data$v_108 == 0] <- NA
data$v_14[data$v_14 == 0] <- NA
data$v_79[data$v_79 == 0] <- NA
data$v_98[data$v_98 == 0] <- NA
data$v_110[data$v_110 == 0] <- NA
data$v_16[data$v_16 == 0] <- NA
data$v_81[data$v_81 == 0] <- NA
data$v_100[data$v_100 == 0] <- NA
data$v_112[data$v_112 == 0] <- NA
data$v_71[data$v_71 == 0] <- NA
data$v_83[data$v_83 == 0] <- NA
data$v_102[data$v_102 == 0] <- NA
data$v_114[data$v_114 == 0] <- NA

data$accessibility_1 <- coalesce(data$v_10, data$v_72)
table(data$accessibility_1)
```

```
## 
##   1   2   3   4   5   6   7   8 
##  52  82 192 353 425 527 409 471
```

```
data$accessibility_2 <- coalesce(data$v_91, data$v_103)
table(data$accessibility_2)
```

```
## 
##   1   2   3   4   5   6   7   8 
##  67  74 177 321 353 393 321 331
```

```
data$understanding_1 <- coalesce(data$v_11, data$v_73)
table(data$understanding_1)
```

```
## 
##   1   2   3   4   5   6   7   8 
##  30  55 151 342 446 576 458 445
```

```
data$understanding_2 <- coalesce(data$v_92, data$v_104)
table(data$understanding_2)
```

```
## 
##   1   2   3   4   5   6   7   8 
##  51  76 160 304 410 459 327 251
```

```
data$empowerment_1 <- coalesce(data$v_47, data$v_74)
table(data$empowerment_1)
```

```
## 
##   1   2   3   4   5   6   7   8 
## 125 137 315 468 566 469 227 190
```

```
data$empowerment_2 <- coalesce(data$v_93, data$v_105)
table(data$empowerment_2)
```

```
## 
##   1   2   3   4   5   6   7   8 
## 131 127 232 410 449 367 182 144
```

```
data$credibility_1 <- coalesce(data$v_48, data$v_75)
table(data$credibility_1)
```

```
## 
##   1   2   3   4   5   6   7   8 
##  12  29  87 344 469 601 487 470
```

```
data$credibility_2 <- coalesce(data$v_94, data$v_106)
table(data$credibility_2)
```

```
## 
##   1   2   3   4   5   6   7   8 
##  26  25  91 296 423 465 372 341
```

```
data$relevance_1 <- coalesce(data$v_49, data$v_76)
table(data$relevance_1)
```

```
## 
##   1   2   3   4   5   6   7   8 
##  17  30  62 231 327 559 466 808
```

```
data$relevance_2 <- coalesce(data$v_95, data$v_107)
table(data$relevance_2)
```

```
## 
##   1   2   3   4   5   6   7   8 
##  32  26  77 204 298 411 397 594
```

```
data$curiosity_1 <- coalesce(data$v_12, data$v_77)
table(data$curiosity_1)
```

```
## 
##   1   2   3   4   5 
## 162 414 773 816 345
```

```
data$curiosity_2 <- coalesce(data$v_96, data$v_108)
table(data$curiosity_2)
```

```
## 
##   1   2   3   4   5 
## 168 420 642 542 273
```

```
data$boredom_1 <- coalesce(data$v_14, data$v_79)
table(data$boredom_1)
```

```
## 
##    1    2    3    4    5 
## 1045  671  542  168   76
```

```
data$boredom_2 <- coalesce(data$v_98, data$v_110)
table(data$boredom_2)
```

```
## 
##   1   2   3   4   5 
## 881 507 424 150  81
```

```
data$confusion_1 <- coalesce(data$v_16, data$v_81)
table(data$confusion_1)
```

```
## 
##   1   2   3   4   5 
## 939 751 598 162  54
```

```
data$confusion_2 <- coalesce(data$v_100, data$v_112)
table(data$confusion_2)
```

```
## 
##   1   2   3   4   5 
## 733 582 493 169  68
```

```
data$frustration_1 <- coalesce(data$v_71, data$v_83)
table(data$frustration_1)
```

```
## 
##    1    2    3    4    5 
## 1486  451  419  107   36
```

```
data$frustration_2 <- coalesce(data$v_102, data$v_114)
table(data$frustration_2)
```

```
## 
##    1    2    3    4    5 
## 1136  392  355  104   58
```

### Merge and Recode Relationship-Item

```
data$v_17[data$v_17 == 0] <- NA
data$v_126[data$v_126 == 0] <- NA
data$v_18[data$v_18 == 0] <- NA
data$v_127[data$v_127 == 0] <- NA
data$v_19[data$v_19 == 0] <- NA
data$v_128[data$v_128 == 0] <- NA
data$v_20[data$v_20 == 0] <- NA
data$v_129[data$v_129 == 0] <- NA
data$v_21[data$v_21 == 0] <- NA
data$v_130[data$v_130 == 0] <- NA
data$v_115[data$v_115 == 0] <- NA
data$v_131[data$v_131 == 0] <- NA
data$v_116[data$v_116 == 0] <- NA
data$v_132[data$v_132 == 0] <- NA
data$v_117[data$v_117 == 0] <- NA
data$v_133[data$v_133 == 0] <- NA

data$s_relationship_1 <- coalesce(data$v_17, data$v_126)
data$s_relationship_2 <- coalesce(data$v_18, data$v_127)
data$s_relationship_3 <- coalesce(data$v_19, data$v_128)
data$s_relationship_4 <- coalesce(data$v_20, data$v_129)
data$s_relationship_5 <- coalesce(data$v_21, data$v_130)
data$s_relationship_6 <- coalesce(data$v_115, data$v_131)
data$s_relationship_7 <- coalesce(data$v_116, data$v_132)
data$s_relationship_8 <- coalesce(data$v_117, data$v_133)

data$s_relationship_1 <- mapvalues(data$s_relationship_1, c(1,2,3), c(1,-1,0))
table(data$s_relationship_1)
```

```
## 
##   -1    0    1 
##  233  345 1690
```

```
data$s_relationship_2 <- mapvalues(data$s_relationship_2, c(1,2,3), c(-1,1,0))
table(data$s_relationship_2)
```

```
## 
##   -1    0    1 
## 1175  406  683
```

```
data$s_relationship_3 <- mapvalues(data$s_relationship_3, c(1,2,3), c(-1,1,0))
table(data$s_relationship_3)
```

```
## 
##  -1   0   1 
## 671 772 822
```

```
data$s_relationship_4 <- mapvalues(data$s_relationship_4, c(1,2,3), c(-1,1,0))
table(data$s_relationship_4)
```

```
## 
##  -1   0   1 
## 730 736 801
```

```
data$s_relationship_5 <- mapvalues(data$s_relationship_5, c(1,2,3), c(1,-1,0))
table(data$s_relationship_5)
```

```
## 
##   -1    0    1 
##  256  375 1638
```

```
data$s_relationship_6 <- mapvalues(data$s_relationship_6, c(1,2,3), c(-1,1,0))
table(data$s_relationship_6)
```

```
## 
##   -1    0    1 
## 1321  426  518
```

```
data$s_relationship_7 <- mapvalues(data$s_relationship_7, c(1,2,3), c(-1,1,0))
table(data$s_relationship_7)
```

```
## 
##   -1    0    1 
## 1349  448  467
```

```
data$s_relationship_8 <- mapvalues(data$s_relationship_8, c(1,2,3), c(-1,1,0))
table(data$s_relationship_8)
```

```
## 
##   -1    0    1 
## 1156  464  642
```

### Merge and Recode Extent of Evaluation-Item

```
data$v_22[data$v_22 == 0] <- NA
data$v_134[data$v_134 == 0] <- NA
data$v_23[data$v_23 == 0] <- NA
data$v_135[data$v_135 == 0] <- NA
data$v_24[data$v_24 == 0] <- NA
data$v_136[data$v_136 == 0] <- NA
data$v_25[data$v_25 == 0] <- NA
data$v_137[data$v_137 == 0] <- NA
data$v_26[data$v_26 == 0] <- NA
data$v_138[data$v_138 == 0] <- NA
data$v_120[data$v_120 == 0] <- NA
data$v_139[data$v_139 == 0] <- NA

data$s_extent_1 <- coalesce(data$v_22, data$v_134)
data$s_extent_2 <- coalesce(data$v_23, data$v_135)
data$s_extent_3 <- coalesce(data$v_24, data$v_136)
data$s_extent_4 <- coalesce(data$v_25, data$v_137)
data$s_extent_5 <- coalesce(data$v_26, data$v_138)
data$s_extent_6 <- coalesce(data$v_120, data$v_139)

data$s_extent_1 <- mapvalues(data$s_extent_1, c(1,2,3), c(-1,1,0))
table(data$s_extent_1)
```

```
## 
##  -1   0   1 
## 663 773 832
```

```
data$s_extent_2 <- mapvalues(data$s_extent_2, c(1,2,3), c(-1,1,0))
table(data$s_extent_2)
```

```
## 
##  -1   0   1 
## 931 585 750
```

```
data$s_extent_3 <- mapvalues(data$s_extent_3, c(1,2,3), c(-1,1,0))
table(data$s_extent_3)
```

```
## 
##   -1    0    1 
## 1035  740  488
```

```
data$s_extent_4 <- mapvalues(data$s_extent_4, c(1,2,3), c(-1,1,0))
table(data$s_extent_4)
```

```
## 
##  -1   0   1 
## 968 763 533
```

```
data$s_extent_5 <- mapvalues(data$s_extent_5, c(1,2,3), c(1,-1,0))
table(data$s_extent_5)
```

```
## 
##   -1    0    1 
##  317  535 1411
```

```
data$s_extent_6 <- mapvalues(data$s_extent_6, c(1,2,3), c(1,-1,0))
table(data$s_extent_6)
```

```
## 
##   -1    0    1 
##  380  497 1393
```

### Merge and Recode Differentiation-Item

```
# Caution: Due to an error (wrong answers provided during experiment), all values for Faerber et al. are NA. Only answers for Barth et al. can be considered for analysis

data$v_27[data$v_27 == 0] <- NA
data$v_28[data$v_28 == 0] <- NA
data$v_29[data$v_29 == 0] <- NA
data$v_30[data$v_30 == 0] <- NA
data$v_31[data$v_31 == 0] <- NA
data$v_121[data$v_121 == 0] <- NA

data$v_140 <- NA
data$v_141 <- NA
data$v_142 <- NA
data$v_143 <- NA
data$v_144 <- NA
data$v_145 <- NA

data$v_235[data$v_235 == 0] <- NA
data$v_236[data$v_236 == 0] <- NA
data$v_237[data$v_237 == 0] <- NA
data$v_238[data$v_238 == 0] <- NA
data$v_239[data$v_239 == 0] <- NA
data$v_240[data$v_240 == 0] <- NA

data$v_274 <- NA
data$v_275 <- NA
data$v_276 <- NA
data$v_277 <- NA
data$v_278 <- NA
data$v_279 <- NA

#Values only need to be mapped for the items from Barth et al.
data$v_27 <- mapvalues(data$v_27, c(1,2,3),c(-1,1,0))
table(data$v_27)
```

```
## 
##  -1   0   1 
## 529 305 305
```

```
data$v_28 <- mapvalues(data$v_28, c(1,2,3),c(-1,1,0))
table(data$v_28)
```

```
## 
##  -1   0   1 
## 561 297 284
```

```
data$v_29 <- mapvalues(data$v_29, c(1,2,3),c(1,-1,0))
table(data$v_29)
```

```
## 
##  -1   0   1 
## 371 321 447
```

```
data$v_30 <- mapvalues(data$v_30, c(1,2,3),c(-1,1,0))
table(data$v_30)
```

```
## 
##  -1   0   1 
## 373 298 467
```

```
data$v_31 <- mapvalues(data$v_31, c(1,2,3),c(1,-1,0))
table(data$v_31)
```

```
## 
##  -1   0   1 
## 262 404 474
```

```
data$v_121 <- mapvalues(data$v_121, c(1,2,3),c(1,-1,0))
table(data$v_121)
```

```
## 
##  -1   0   1 
## 317 343 483
```

```
data$v_235 <- mapvalues(data$v_235, c(1,2,3),c(-1,1,0))
table(data$v_235)
```

```
## 
##  -1   0   1 
## 386 229 397
```

```
data$v_236 <- mapvalues(data$v_236, c(1,2,3),c(-1,1,0))
table(data$v_236)
```

```
## 
##  -1   0   1 
## 415 255 344
```

```
data$v_237 <- mapvalues(data$v_237, c(1,2,3),c(1,-1,0))
table(data$v_237)
```

```
## 
##  -1   0   1 
## 376 255 377
```

```
data$v_238 <- mapvalues(data$v_238, c(1,2,3),c(-1,1,0))
table(data$v_238)
```

```
## 
##  -1   0   1 
## 282 252 481
```

```
data$v_239 <- mapvalues(data$v_239, c(1,2,3),c(1,-1,0))
table(data$v_239)
```

```
## 
##  -1   0   1 
## 259 250 500
```

```
data$v_240 <- mapvalues(data$v_240, c(1,2,3),c(1,-1,0))
table(data$v_240)
```

```
## 
##  -1   0   1 
## 311 255 446
```

```
#Merge for T1
data$s_diff_1_1 <- coalesce(data$v_27, data$v_140)
table(data$s_diff_1_1)
```

```
## 
##  -1   0   1 
## 529 305 305
```

```
data$s_diff_1_2 <- coalesce(data$v_28, data$v_141)
table(data$s_diff_1_2)
```

```
## 
##  -1   0   1 
## 561 297 284
```

```
data$s_diff_1_3 <- coalesce(data$v_29, data$v_142)
table(data$s_diff_1_3)
```

```
## 
##  -1   0   1 
## 371 321 447
```

```
data$s_diff_1_4 <- coalesce(data$v_30, data$v_143)
table(data$s_diff_1_4)
```

```
## 
##  -1   0   1 
## 373 298 467
```

```
data$s_diff_1_5 <- coalesce(data$v_31, data$v_144)
table(data$s_diff_1_5)
```

```
## 
##  -1   0   1 
## 262 404 474
```

```
data$s_diff_1_6 <- coalesce(data$v_121, data$v_145)
table(data$s_diff_1_6)
```

```
## 
##  -1   0   1 
## 317 343 483
```

```
#Merge for T2
data$s_diff_2_1 <- coalesce(data$v_235, data$v_274)
table(data$s_diff_2_1)
```

```
## 
##  -1   0   1 
## 386 229 397
```

```
data$s_diff_2_2 <- coalesce(data$v_236, data$v_275)
table(data$s_diff_2_2)
```

```
## 
##  -1   0   1 
## 415 255 344
```

```
data$s_diff_2_3 <- coalesce(data$v_237, data$v_276)
table(data$s_diff_2_3)
```

```
## 
##  -1   0   1 
## 376 255 377
```

```
data$s_diff_2_4 <- coalesce(data$v_238, data$v_277)
table(data$s_diff_2_4)
```

```
## 
##  -1   0   1 
## 282 252 481
```

```
data$s_diff_2_5 <- coalesce(data$v_239, data$v_278)
table(data$s_diff_2_5)
```

```
## 
##  -1   0   1 
## 259 250 500
```

```
data$s_diff_2_6 <- coalesce(data$v_240, data$v_279)
table(data$s_diff_2_6)
```

```
## 
##  -1   0   1 
## 311 255 446
```

### Merge and Recode Funding-Item

```
data$v_32[data$v_32 == 0] <- NA
data$v_33[data$v_33 == 0] <- NA
data$v_34[data$v_34 == 0] <- NA
data$v_35[data$v_35 == 0] <- NA
data$v_36[data$v_36 == 0] <- NA
data$v_122[data$v_122 == 0] <- NA

data$v_146[data$v_146 == 0] <- NA
data$v_147[data$v_147 == 0] <- NA
data$v_148[data$v_148 == 0] <- NA
data$v_149[data$v_149 == 0] <- NA
data$v_150[data$v_150 == 0] <- NA
data$v_151[data$v_151 == 0] <- NA

data$v_241[data$v_241 == 0] <- NA
data$v_242[data$v_242 == 0] <- NA
data$v_243[data$v_243 == 0] <- NA
data$v_244[data$v_244 == 0] <- NA
data$v_245[data$v_245 == 0] <- NA
data$v_246[data$v_246 == 0] <- NA

data$v_280[data$v_280 == 0] <- NA
data$v_281[data$v_281 == 0] <- NA
data$v_282[data$v_282 == 0] <- NA
data$v_283[data$v_283 == 0] <- NA
data$v_284[data$v_284 == 0] <- NA
data$v_285[data$v_285 == 0] <- NA

data$v_32 <- mapvalues(data$v_32, c(1,2,3), c(-1,1,0))
table(data$v_32)
```

```
## 
##  -1   0   1 
## 363 418 357
```

```
data$v_33 <- mapvalues(data$v_33, c(1,2,3), c(-1,1,0))
table(data$v_33)
```

```
## 
##  -1   0   1 
## 335 406 401
```

```
data$v_34 <- mapvalues(data$v_34, c(1,2,3), c(-1,1,0))
table(data$v_34)
```

```
## 
##  -1   0   1 
## 283 333 524
```

```
data$v_35 <- mapvalues(data$v_35, c(1,2,3), c(-1,1,0))
table(data$v_35)
```

```
## 
##  -1   0   1 
## 316 452 374
```

```
data$v_36 <- mapvalues(data$v_36, c(1,2,3), c(-1,1,0))
table(data$v_36)
```

```
## 
##  -1   0   1 
## 341 434 367
```

```
data$v_122 <- mapvalues(data$v_122, c(1,2,3), c(1,-1,0))
table(data$v_122)
```

```
## 
##  -1   0   1 
## 240 400 501
```

```
data$v_146 <- mapvalues(data$v_146, c(1,2,3), c(-1,1,0))
table(data$v_122)
```

```
## 
##  -1   0   1 
## 240 400 501
```

```
data$v_147 <- mapvalues(data$v_147, c(1,2,3), c(-1,1,0))
table(data$v_147)
```

```
## 
##  -1   0   1 
## 311 396 420
```

```
data$v_148 <- mapvalues(data$v_148, c(1,2,3), c(-1,1,0))
table(data$v_148)
```

```
## 
##  -1   0   1 
## 200 443 480
```

```
data$v_149 <- mapvalues(data$v_149, c(1,2,3), c(1,-1,0))
table(data$v_149)
```

```
## 
##  -1   0   1 
## 241 324 559
```

```
data$v_150 <- mapvalues(data$v_150, c(1,2,3), c(-1,1,0))
table(data$v_150)
```

```
## 
##  -1   0   1 
## 226 437 460
```

```
data$v_151 <- mapvalues(data$v_151, c(1,2,3), c(-1,1,0))
table(data$v_151)
```

```
## 
##  -1   0   1 
## 249 405 465
```

```
data$v_241 <- mapvalues(data$v_241, c(1,2,3), c(-1,1,0))
table(data$v_241)
```

```
## 
##  -1   0   1 
## 223 257 533
```

```
data$v_242 <- mapvalues(data$v_242, c(1,2,3), c(-1,1,0))
table(data$v_242)
```

```
## 
##  -1   0   1 
## 227 277 508
```

```
data$v_243 <- mapvalues(data$v_243, c(1,2,3), c(-1,1,0))
table(data$v_243)
```

```
## 
##  -1   0   1 
## 193 234 590
```

```
data$v_244 <- mapvalues(data$v_244, c(1,2,3), c(-1,1,0))
table(data$v_244)
```

```
## 
##  -1   0   1 
## 310 274 431
```

```
data$v_245 <- mapvalues(data$v_245, c(1,2,3), c(-1,1,0))
table(data$v_245)
```

```
## 
##  -1   0   1 
## 307 257 450
```

```
data$v_246 <- mapvalues(data$v_246, c(1,2,3), c(1,-1,0))
table(data$v_246)
```

```
## 
##  -1   0   1 
## 192 240 585
```

```
data$v_280 <- mapvalues(data$v_280, c(1,2,3), c(-1,1,0))
table(data$v_280)
```

```
## 
##  -1   0   1 
## 263 276 487
```

```
data$v_281 <- mapvalues(data$v_281, c(1,2,3), c(-1,1,0))
table(data$v_281)
```

```
## 
##  -1   0   1 
## 239 279 507
```

```
data$v_282 <- mapvalues(data$v_282, c(1,2,3), c(-1,1,0))
table(data$v_282)
```

```
## 
##  -1   0   1 
## 212 307 506
```

```
data$v_283 <- mapvalues(data$v_283, c(1,2,3), c(1,-1,0))
table(data$v_283)
```

```
## 
##  -1   0   1 
## 197 259 572
```

```
data$v_284 <- mapvalues(data$v_284, c(1,2,3), c(-1,1,0))
table(data$v_284)
```

```
## 
##  -1   0   1 
## 230 296 501
```

```
data$v_285 <- mapvalues(data$v_285, c(1,2,3), c(-1,1,0))
table(data$v_285)
```

```
## 
##  -1   0   1 
## 185 311 527
```

```
# Merge for T1
data$s_funding_1_1 <- coalesce(data$v_32, data$v_146)
table(data$s_funding_1_1)
```

```
## 
##  -1   0   1 
## 716 797 749
```

```
data$s_funding_1_2 <- coalesce(data$v_33, data$v_147)
table(data$s_funding_1_2)
```

```
## 
##  -1   0   1 
## 646 802 821
```

```
data$s_funding_1_3 <- coalesce(data$v_34, data$v_148)
table(data$s_funding_1_3)
```

```
## 
##   -1    0    1 
##  483  776 1004
```

```
data$s_funding_1_4 <- coalesce(data$v_35, data$v_149)
table(data$s_funding_1_4)
```

```
## 
##  -1   0   1 
## 557 776 933
```

```
data$s_funding_1_5 <- coalesce(data$v_36, data$v_150)
table(data$s_funding_1_5)
```

```
## 
##  -1   0   1 
## 567 871 827
```

```
data$s_funding_1_6 <- coalesce(data$v_122, data$v_151)
table(data$s_funding_1_6)
```

```
## 
##  -1   0   1 
## 489 805 966
```

```
# Merge for T2
data$s_funding_2_1 <- coalesce(data$v_241, data$v_280)
table(data$s_funding_2_1)
```

```
## 
##   -1    0    1 
##  486  533 1020
```

```
data$s_funding_2_2 <- coalesce(data$v_242, data$v_281)
table(data$s_funding_2_2)
```

```
## 
##   -1    0    1 
##  466  556 1015
```

```
data$s_funding_2_3 <- coalesce(data$v_243, data$v_282)
table(data$s_funding_2_3)
```

```
## 
##   -1    0    1 
##  405  541 1096
```

```
data$s_funding_2_4 <- coalesce(data$v_244, data$v_283)
table(data$s_funding_2_4)
```

```
## 
##   -1    0    1 
##  507  533 1003
```

```
data$s_funding_2_5 <- coalesce(data$v_245, data$v_284)
table(data$s_funding_2_5)
```

```
## 
##  -1   0   1 
## 537 553 951
```

```
data$s_funding_2_6 <- coalesce(data$v_246, data$v_285)
table(data$s_funding_2_6)
```

```
## 
##   -1    0    1 
##  377  551 1112
```

### Merge and Recode COI-Item

```
data$v_37[data$v_37 == 0] <- NA
data$v_38[data$v_38 == 0] <- NA
data$v_39[data$v_39 == 0] <- NA
data$v_40[data$v_40 == 0] <- NA
data$v_41[data$v_41 == 0] <- NA
data$v_123[data$v_123 == 0] <- NA
data$v_124[data$v_124 == 0] <- NA

data$v_152[data$v_152 == 0] <- NA
data$v_153[data$v_153 == 0] <- NA
data$v_154[data$v_154 == 0] <- NA
data$v_155[data$v_155 == 0] <- NA
data$v_156[data$v_156 == 0] <- NA
data$v_157[data$v_157 == 0] <- NA
data$v_158[data$v_158 == 0] <- NA

data$v_247[data$v_247 == 0] <- NA
data$v_248[data$v_248 == 0] <- NA
data$v_249[data$v_249 == 0] <- NA
data$v_250[data$v_250 == 0] <- NA
data$v_251[data$v_251 == 0] <- NA
data$v_252[data$v_252 == 0] <- NA
data$v_253[data$v_253 == 0] <- NA

data$v_286[data$v_286 == 0] <- NA
data$v_287[data$v_287 == 0] <- NA
data$v_288[data$v_288 == 0] <- NA
data$v_289[data$v_289 == 0] <- NA
data$v_290[data$v_290 == 0] <- NA
data$v_291[data$v_291 == 0] <- NA
data$v_292[data$v_292 == 0] <- NA

data$v_37 <- mapvalues(data$v_37,c(1,2,3),c(-1,1,0))
table(data$v_37)
```

```
## 
##  -1   0   1 
## 360 388 393
```

```
data$v_38 <- mapvalues(data$v_38,c(1,2,3),c(-1,1,0))
table(data$v_38)
```

```
## 
##  -1   0   1 
## 356 360 417
```

```
data$v_39 <- mapvalues(data$v_39,c(1,2,3),c(-1,1,0))
table(data$v_39)
```

```
## 
##  -1   0   1 
## 328 386 414
```

```
data$v_40 <- mapvalues(data$v_40,c(1,2,3),c(-1,1,0))
table(data$v_40)
```

```
## 
##  -1   0   1 
## 385 389 367
```

```
data$v_41 <- mapvalues(data$v_41,c(1,2,3),c(-1,1,0))
table(data$v_41)
```

```
## 
##  -1   0   1 
## 309 388 437
```

```
data$v_123 <- mapvalues(data$v_123,c(1,2,3),c(1,-1,0))
table(data$v_42)
```

```
## 
##   0   1   2   3 
##   9 542 339 257
```

```
data$v_124 <- mapvalues(data$v_124,c(1,2,3),c(-1,1,0))
table(data$v_124)
```

```
## 
##  -1   0   1 
## 345 395 397
```

```
data$v_152 <- mapvalues(data$v_152,c(1,2,3),c(-1,1,0))
table(data$v_152)
```

```
## 
##  -1   0   1 
## 306 371 446
```

```
data$v_153 <- mapvalues(data$v_153,c(1,2,3),c(-1,1,0))
table(data$v_153)
```

```
## 
##  -1   0   1 
## 270 380 469
```

```
data$v_154 <- mapvalues(data$v_154,c(1,2,3),c(-1,1,0))
table(data$v_154)
```

```
## 
##  -1   0   1 
## 302 366 452
```

```
data$v_155 <- mapvalues(data$v_155,c(1,2,3),c(-1,1,0))
table(data$v_155)
```

```
## 
##  -1   0   1 
## 317 379 428
```

```
data$v_156 <- mapvalues(data$v_156,c(1,2,3),c(-1,1,0))
table(data$v_156)
```

```
## 
##  -1   0   1 
## 265 375 482
```

```
data$v_157 <- mapvalues(data$v_157,c(1,2,3),c(-1,1,0))
table(data$v_157)
```

```
## 
##  -1   0   1 
## 276 378 467
```

```
data$v_158 <- mapvalues(data$v_158,c(1,2,3),c(1,-1,0))
table(data$v_158)
```

```
## 
##  -1   0   1 
## 293 377 453
```

```
data$v_247 <- mapvalues(data$v_247,c(1,2,3),c(-1,1,0))
table(data$v_247)
```

```
## 
##  -1   0   1 
## 316 265 436
```

```
data$v_248 <- mapvalues(data$v_248,c(1,2,3),c(-1,1,0))
table(data$v_248)
```

```
## 
##  -1   0   1 
## 276 287 451
```

```
data$v_249 <- mapvalues(data$v_249,c(1,2,3),c(-1,1,0))
table(data$v_249)
```

```
## 
##  -1   0   1 
## 269 293 452
```

```
data$v_250 <- mapvalues(data$v_250,c(1,2,3),c(-1,1,0))
table(data$v_250)
```

```
## 
##  -1   0   1 
## 312 301 403
```

```
data$v_251 <- mapvalues(data$v_251,c(1,2,3),c(-1,1,0))
table(data$v_251)
```

```
## 
##  -1   0   1 
## 261 300 455
```

```
data$v_252 <- mapvalues(data$v_252,c(1,2,3),c(1,-1,0))
table(data$v_252)
```

```
## 
##  -1   0   1 
## 375 301 336
```

```
data$v_253 <- mapvalues(data$v_253,c(1,2,3),c(-1,1,0))
table(data$v_253)
```

```
## 
##  -1   0   1 
## 255 307 453
```

```
data$v_286 <- mapvalues(data$v_286,c(1,2,3),c(-1,1,0))
table(data$v_286)
```

```
## 
##  -1   0   1 
## 246 270 511
```

```
data$v_287 <- mapvalues(data$v_287,c(1,2,3),c(-1,1,0))
table(data$v_287)
```

```
## 
##  -1   0   1 
## 260 284 482
```

```
data$v_288 <- mapvalues(data$v_288,c(1,2,3),c(-1,1,0))
table(data$v_288)
```

```
## 
##  -1   0   1 
## 247 260 518
```

```
data$v_289 <- mapvalues(data$v_289,c(1,2,3),c(-1,1,0))
table(data$v_289)
```

```
## 
##  -1   0   1 
## 238 283 505
```

```
data$v_290 <- mapvalues(data$v_290,c(1,2,3),c(-1,1,0))
table(data$v_290)
```

```
## 
##  -1   0   1 
## 241 272 513
```

```
data$v_291 <- mapvalues(data$v_291,c(1,2,3),c(-1,1,0))
table(data$v_291)
```

```
## 
##  -1   0   1 
## 231 282 513
```

```
data$v_292 <- mapvalues(data$v_292,c(1,2,3),c(1,-1,0))
table(data$v_292)
```

```
## 
##  -1   0   1 
## 316 266 443
```

```
# Merge for T1
data$s_coi_1_1 <- coalesce(data$v_37, data$v_152)
table(data$s_coi_1_1)
```

```
## 
##  -1   0   1 
## 666 759 839
```

```
data$s_coi_1_2 <- coalesce(data$v_38, data$v_153)
table(data$s_coi_1_2)
```

```
## 
##  -1   0   1 
## 626 740 886
```

```
data$s_coi_1_3 <- coalesce(data$v_39, data$v_154)
table(data$s_coi_1_3)
```

```
## 
##  -1   0   1 
## 630 752 866
```

```
data$s_coi_1_4 <- coalesce(data$v_40, data$v_155)
table(data$s_coi_1_4)
```

```
## 
##  -1   0   1 
## 702 768 795
```

```
data$s_coi_1_5 <- coalesce(data$v_41, data$v_156)
table(data$s_coi_1_5)
```

```
## 
##  -1   0   1 
## 574 763 919
```

```
data$s_coi_1_6 <- coalesce(data$v_123, data$v_157)
table(data$s_coi_1_6)
```

```
## 
##  -1   0   1 
## 644 765 854
```

```
data$s_coi_1_7 <- coalesce(data$v_124, data$v_158)
table(data$s_coi_1_7)
```

```
## 
##  -1   0   1 
## 638 772 850
```

```
# Merge for T2
data$s_coi_2_1 <- coalesce(data$v_247, data$v_286)
table(data$s_coi_2_1)
```

```
## 
##  -1   0   1 
## 562 535 947
```

```
data$s_coi_2_2 <- coalesce(data$v_248, data$v_287)
table(data$s_coi_2_2)
```

```
## 
##  -1   0   1 
## 536 571 933
```

```
data$s_coi_2_3 <- coalesce(data$v_249, data$v_288)
table(data$s_coi_2_3)
```

```
## 
##  -1   0   1 
## 516 553 970
```

```
data$s_coi_2_4 <- coalesce(data$v_250, data$v_289)
table(data$s_coi_2_4)
```

```
## 
##  -1   0   1 
## 550 584 908
```

```
data$s_coi_2_5 <- coalesce(data$v_251, data$v_290)
table(data$s_coi_2_5)
```

```
## 
##  -1   0   1 
## 502 572 968
```

```
data$s_coi_2_6 <- coalesce(data$v_252, data$v_291)
table(data$s_coi_2_6)
```

```
## 
##  -1   0   1 
## 606 583 849
```

```
data$s_coi_2_7 <- coalesce(data$v_253, data$v_292)
table(data$s_coi_2_7)
```

```
## 
##  -1   0   1 
## 571 573 896
```

### Merge and Recode Causality-Item

```
data$v_42[data$v_42 == 0] <- NA
data$v_43[data$v_43 == 0] <- NA
data$v_44[data$v_44 == 0] <- NA
data$v_45[data$v_45 == 0] <- NA
data$v_46[data$v_46 == 0] <- NA
data$v_125[data$v_125 == 0] <- NA

data$v_159[data$v_159 == 0] <- NA
data$v_160[data$v_160 == 0] <- NA
data$v_161[data$v_161 == 0] <- NA
data$v_162[data$v_162 == 0] <- NA
data$v_163[data$v_163 == 0] <- NA
data$v_164[data$v_164 == 0] <- NA

data$v_254[data$v_254 == 0] <- NA
data$v_255[data$v_255 == 0] <- NA
data$v_256[data$v_256 == 0] <- NA
data$v_257[data$v_257 == 0] <- NA
data$v_258[data$v_258 == 0] <- NA
data$v_259[data$v_259 == 0] <- NA

data$v_293[data$v_293 == 0] <- NA
data$v_294[data$v_294 == 0] <- NA
data$v_295[data$v_295 == 0] <- NA
data$v_296[data$v_296 == 0] <- NA
data$v_297[data$v_297 == 0] <- NA
data$v_298[data$v_298 == 0] <- NA

data$v_42 <- mapvalues(data$v_42, c(1,2,3), c(1,-1,0))
table(data$v_42)
```

```
## 
##  -1   0   1 
## 339 257 542
```

```
data$v_43 <- mapvalues(data$v_43, c(1,2,3), c(-1,1,0))
table(data$v_43)
```

```
## 
##  -1   0   1 
## 512 273 358
```

```
data$v_44 <- mapvalues(data$v_44, c(1,2,3), c(-1,1,0))
table(data$v_44)
```

```
## 
##  -1   0   1 
## 406 373 361
```

```
data$v_45 <- mapvalues(data$v_45, c(1,2,3), c(-1,1,0))
table(data$v_45)
```

```
## 
##  -1   0   1 
## 423 382 337
```

```
data$v_46 <- mapvalues(data$v_46, c(1,2,3), c(-1,1,0))
table(data$v_46)
```

```
## 
##  -1   0   1 
## 476 313 353
```

```
data$v_125 <- mapvalues(data$v_125, c(1,2,3), c(-1,1,0))
table(data$v_125)
```

```
## 
##  -1   0   1 
## 505 342 294
```

```
data$v_159 <- mapvalues(data$v_159, c(1,2,3), c(1,-1,0))
table(data$v_159)
```

```
## 
##  -1   0   1 
## 115 186 824
```

```
data$v_160 <- mapvalues(data$v_160, c(1,2,3), c(-1,1,0))
table(data$v_160)
```

```
## 
##  -1   0   1 
## 555 266 301
```

```
data$v_161 <- mapvalues(data$v_161, c(1,2,3), c(-1,1,0))
table(data$v_161)
```

```
## 
##  -1   0   1 
## 642 224 257
```

```
data$v_162 <- mapvalues(data$v_162, c(1,2,3), c(-1,1,0))
table(data$v_162)
```

```
## 
##  -1   0   1 
## 686 217 219
```

```
data$v_163 <- mapvalues(data$v_163, c(1,2,3), c(-1,1,0))
table(data$v_163)
```

```
## 
##  -1   0   1 
## 455 314 352
```

```
data$v_164 <- mapvalues(data$v_164, c(1,2,3), c(-1,1,0))
table(data$v_164)
```

```
## 
##  -1   0   1 
## 438 314 371
```

```
data$v_254 <- mapvalues(data$v_254, c(1,2,3), c(1,-1,0))
table(data$v_254)
```

```
## 
##  -1   0   1 
## 305 234 475
```

```
data$v_255 <- mapvalues(data$v_255, c(1,2,3), c(-1,1,0))
table(data$v_255)
```

```
## 
##  -1   0   1 
## 416 255 341
```

```
data$v_256 <- mapvalues(data$v_256, c(1,2,3), c(-1,1,0))
table(data$v_256)
```

```
## 
##  -1   0   1 
## 347 330 339
```

```
data$v_257 <- mapvalues(data$v_257, c(1,2,3), c(-1,1,0))
table(data$v_257)
```

```
## 
##  -1   0   1 
## 341 344 323
```

```
data$v_258 <- mapvalues(data$v_258, c(1,2,3), c(-1,1,0))
table(data$v_258)
```

```
## 
##  -1   0   1 
## 392 295 328
```

```
data$v_259 <- mapvalues(data$v_259, c(1,2,3), c(-1,1,0))
table(data$v_259)
```

```
## 
##  -1   0   1 
## 392 293 330
```

```
data$v_293 <- mapvalues(data$v_293, c(1,2,3), c(1,-1,0))
table(data$v_293)
```

```
## 
##  -1   0   1 
## 124 204 698
```

```
data$v_294 <- mapvalues(data$v_294, c(1,2,3), c(-1,1,0))
table(data$v_294)
```

```
## 
##  -1   0   1 
## 436 295 292
```

```
data$v_295 <- mapvalues(data$v_295, c(1,2,3), c(-1,1,0))
table(data$v_295)
```

```
## 
##  -1   0   1 
## 535 231 261
```

```
data$v_296 <- mapvalues(data$v_296, c(1,2,3), c(-1,1,0))
table(data$v_296)
```

```
## 
##  -1   0   1 
## 597 240 185
```

```
data$v_297 <- mapvalues(data$v_297, c(1,2,3), c(-1,1,0))
table(data$v_297)
```

```
## 
##  -1   0   1 
## 384 311 331
```

```
data$v_298 <- mapvalues(data$v_298, c(1,2,3), c(-1,1,0))
table(data$v_298)
```

```
## 
##  -1   0   1 
## 383 312 328
```

```
# Merge for T1
data$s_causality_1_1 <- coalesce(data$v_42, data$v_159)
table(data$s_causality_1_1)
```

```
## 
##   -1    0    1 
##  454  443 1366
```

```
data$s_causality_1_2 <- coalesce(data$v_43, data$v_160)
table(data$s_causality_1_2)
```

```
## 
##   -1    0    1 
## 1067  539  659
```

```
data$s_causality_1_3 <- coalesce(data$v_44, data$v_161)
table(data$s_causality_1_3)
```

```
## 
##   -1    0    1 
## 1048  597  618
```

```
data$s_causality_1_4 <- coalesce(data$v_45, data$v_162)
table(data$s_causality_1_4)
```

```
## 
##   -1    0    1 
## 1109  599  556
```

```
data$s_causality_1_5 <- coalesce(data$v_46, data$v_163)
table(data$s_causality_1_5)
```

```
## 
##  -1   0   1 
## 931 627 705
```

```
data$s_causality_1_6 <- coalesce(data$v_125, data$v_164)
table(data$s_causality_1_6)
```

```
## 
##  -1   0   1 
## 943 656 665
```

```
# Merge for T2
data$s_causality_2_1 <- coalesce(data$v_254, data$v_293)
table(data$s_causality_2_1)
```

```
## 
##   -1    0    1 
##  429  438 1173
```

```
data$s_causality_2_2 <- coalesce(data$v_255, data$v_294)
table(data$s_causality_2_2)
```

```
## 
##  -1   0   1 
## 852 550 633
```

```
data$s_causality_2_3 <- coalesce(data$v_256, data$v_295)
table(data$s_causality_2_3)
```

```
## 
##  -1   0   1 
## 882 561 600
```

```
data$s_causality_2_4 <- coalesce(data$v_257, data$v_296)
table(data$s_causality_2_4)
```

```
## 
##  -1   0   1 
## 938 584 508
```

```
data$s_causality_2_5 <- coalesce(data$v_258, data$v_297)
table(data$s_causality_2_5)
```

```
## 
##  -1   0   1 
## 776 606 659
```

```
data$s_causality_2_6 <- coalesce(data$v_259, data$v_298)
table(data$s_causality_2_6)
```

```
## 
##  -1   0   1 
## 775 605 658
```

### Merge and Recode CAMA-Items

```
data$v_50[data$v_50 == 0] <- NA
data$v_51[data$v_51 == 0] <- NA
data$v_52[data$v_52 == 0] <- NA
data$v_53[data$v_53 == 0] <- NA
data$v_54[data$v_54 == 0] <- NA
data$v_165[data$v_165 == 0] <- NA
data$v_166[data$v_166 == 0] <- NA
data$v_167[data$v_167 == 0] <- NA

data$v_55[data$v_55 == 0] <- NA
data$v_56[data$v_56 == 0] <- NA
data$v_57[data$v_57 == 0] <- NA
data$v_58[data$v_58 == 0] <- NA

data$v_401[data$v_401 == 0] <- NA

data$v_299[data$v_299 == 0] <- NA
data$v_300[data$v_300 == 0] <- NA
data$v_301[data$v_301 == 0] <- NA
data$v_302[data$v_302 == 0] <- NA
data$v_303[data$v_303 == 0] <- NA
data$v_304[data$v_304 == 0] <- NA
data$v_305[data$v_305 == 0] <- NA
data$v_306[data$v_306 == 0] <- NA

data$v_307[data$v_307 == 0] <- NA
data$v_308[data$v_308 == 0] <- NA
data$v_309[data$v_309 == 0] <- NA
data$v_310[data$v_310 == 0] <- NA

data$v_402[data$v_402 == 0] <- NA

# Caution: For v_401 and v_402, coding is dependent on condition. Items is correct in condition 5, incorrect in conditions 4 and 6.

data$v_50 <- mapvalues(data$v_50, c(1,2,3), c(1,-1,0))
table(data$v_50)
```

```
## 
##  -1   0   1 
##  65 179 284
```

```
data$v_51 <- mapvalues(data$v_51, c(1,2,3), c(-1,1,0))
table(data$v_51)
```

```
## 
##  -1   0   1 
## 129 188 211
```

```
data$v_52 <- mapvalues(data$v_52, c(1,2,3), c(-1,1,0))
table(data$v_52)
```

```
## 
##  -1   0   1 
## 203 217 111
```

```
data$v_53 <- mapvalues(data$v_53, c(1,2,3), c(-1,1,0))
table(data$v_53)
```

```
## 
##  -1   0   1 
## 221 204 100
```

```
data$v_54 <- mapvalues(data$v_54, c(1,2,3), c(1,-1,0))
table(data$v_54)
```

```
## 
##  -1   0   1 
##  81 246 202
```

```
data$v_165 <- mapvalues(data$v_165, c(1,2,3), c(-1,1,0))
table(data$v_165)
```

```
## 
##  -1   0   1 
## 130 297 102
```

```
data$v_166 <- mapvalues(data$v_166, c(1,2,3), c(-1,1,0))
table(data$v_166)
```

```
## 
##  -1   0   1 
##  99 225 206
```

```
data$v_167 <- mapvalues(data$v_167, c(1,2,3), c(-1,1,0))
table(data$v_167)
```

```
## 
##  -1   0   1 
##  98 282 150
```

```
data$v_55 <- mapvalues(data$v_55, c(1,2,3), c(-1,1,0))
table(data$v_55)
```

```
## 
##  -1   0   1 
## 256 191  83
```

```
data$v_56 <- mapvalues(data$v_56, c(1,2,3), c(1,-1,0))
table(data$v_56)
```

```
## 
##  -1   0   1 
## 120 243 166
```

```
data$v_57 <- mapvalues(data$v_57, c(1,2,3), c(-1,1,0))
table(data$v_57)
```

```
## 
##  -1   0   1 
## 155 238 138
```

```
data$v_58 <- mapvalues(data$v_58, c(1,2,3), c(-1,1,0))
table(data$v_58)
```

```
## 
##  -1   0   1 
## 171 226 133
```

```
data$v_401_n <- NA
data$v_401_n <- ifelse(data$condition == 4 & data$v_401 == 1, -1, data$v_401_n)
data$v_401_n <- ifelse(data$condition == 4 & data$v_401 == 2, 1, data$v_401_n)
data$v_401_n <- ifelse(data$condition == 4 & data$v_401 == 3, 0, data$v_401_n)
data$v_401_n <- ifelse(data$condition == 5 & data$v_401 == 1, 1, data$v_401_n)
data$v_401_n <- ifelse(data$condition == 5 & data$v_401 == 2, -1, data$v_401_n)
data$v_401_n <- ifelse(data$condition == 5 & data$v_401 == 3, 0, data$v_401_n)
data$v_401_n <- ifelse(data$condition == 6 & data$v_401 == 1, -1, data$v_401_n)
data$v_401_n <- ifelse(data$condition == 6 & data$v_401 == 2, 1, data$v_401_n)
data$v_401_n <- ifelse(data$condition == 6 & data$v_401 == 3, 0, data$v_401_n)
table(data$v_401_n)
```

```
## 
##  -1   0   1 
## 176 193 163
```

```
data$v_299 <- mapvalues(data$v_299, c(1,2,3), c(1,-1,0))
table(data$v_299)
```

```
## 
##  -1   0   1 
##  88 177 263
```

```
data$v_300 <- mapvalues(data$v_300, c(1,2,3), c(-1,1,0))
table(data$v_300)
```

```
## 
##  -1   0   1 
## 116 209 202
```

```
data$v_301 <- mapvalues(data$v_301, c(1,2,3), c(-1,1,0))
table(data$v_301)
```

```
## 
##  -1   0   1 
## 181 229 118
```

```
data$v_302 <- mapvalues(data$v_302, c(1,2,3), c(-1,1,0))
table(data$v_302)
```

```
## 
##  -1   0   1 
## 185 217 126
```

```
data$v_303 <- mapvalues(data$v_303, c(1,2,3), c(1,-1,0))
table(data$v_303)
```

```
## 
##  -1   0   1 
##  85 238 203
```

```
data$v_304 <- mapvalues(data$v_304, c(1,2,3), c(-1,1,0))
table(data$v_304)
```

```
## 
##  -1   0   1 
## 134 266 122
```

```
data$v_305 <- mapvalues(data$v_305, c(1,2,3), c(-1,1,0))
table(data$v_305)
```

```
## 
##  -1   0   1 
## 108 208 210
```

```
data$v_306 <- mapvalues(data$v_306, c(1,2,3), c(-1,1,0))
table(data$v_306)
```

```
## 
##  -1   0   1 
## 113 256 159
```

```
data$v_307 <- mapvalues(data$v_307, c(1,2,3), c(-1,1,0))
table(data$v_307)
```

```
## 
##  -1   0   1 
## 211 170 147
```

```
data$v_308 <- mapvalues(data$v_308, c(1,2,3), c(1,-1,0))
table(data$v_308)
```

```
## 
##  -1   0   1 
## 143 219 166
```

```
data$v_309 <- mapvalues(data$v_309, c(1,2,3), c(-1,1,0))
table(data$v_309)
```

```
## 
##  -1   0   1 
## 170 213 145
```

```
data$v_310 <- mapvalues(data$v_310, c(1,2,3), c(-1,1,0))
table(data$v_310)
```

```
## 
##  -1   0   1 
## 193 201 135
```

```
data$v_402_n <- NA
data$v_402_n <- ifelse(data$condition == 4 & data$v_402 == 1, -1, data$v_402_n)
data$v_402_n <- ifelse(data$condition == 4 & data$v_402 == 2, 1, data$v_402_n)
data$v_402_n <- ifelse(data$condition == 4 & data$v_402 == 3, 0, data$v_402_n)
data$v_402_n <- ifelse(data$condition == 5 & data$v_402 == 1, 1, data$v_402_n)
data$v_402_n <- ifelse(data$condition == 5 & data$v_402 == 2, -1, data$v_402_n)
data$v_402_n <- ifelse(data$condition == 5 & data$v_402 == 3, 0, data$v_402_n)
data$v_402_n <- ifelse(data$condition == 6 & data$v_402 == 1, -1, data$v_402_n)
data$v_402_n <- ifelse(data$condition == 6 & data$v_402 == 2, 1, data$v_402_n)
data$v_402_n <- ifelse(data$condition == 6 & data$v_402 == 3, 0, data$v_402_n)
table(data$v_402_n)
```

```
## 
##  -1   0   1 
## 141 217 172
```

```
data <- rename(data, s_CAMA_1_1_1 = v_50, s_CAMA_1_1_2 = v_51, s_CAMA_1_1_3 =
                 v_52, s_CAMA_1_1_4 = v_53, s_CAMA_1_1_5 = v_54, s_CAMA_1_1_6 =
                 v_165, s_CAMA_1_1_7 = v_166, s_CAMA_1_1_8 = v_167, 
               s_CAMA_1_2_1 = v_55, s_CAMA_1_2_2 = v_56, s_CAMA_1_2_3 = v_57,
               s_CAMA_1_2_4 = v_58, s_CAMA_1_3 = v_401_n, s_CAMA_2_1_1 = v_299,
               s_CAMA_2_1_2 = v_300, s_CAMA_2_1_3 = v_301, s_CAMA_2_1_4 = v_302,
               s_CAMA_2_1_5 = v_303, s_CAMA_2_1_6 = v_304, s_CAMA_2_1_7 = v_305,
               s_CAMA_2_1_8 = v_306, s_CAMA_2_2_1 = v_307, s_CAMA_2_2_2 = v_308,
               s_CAMA_2_2_3 = v_309, s_CAMA_2_2_4 = v_310, s_CAMA_2_3 = v_402_n)

data$s_CAMA_1_1 <- coalesce(data$s_CAMA_1_1_1, data$s_CAMA_2_1_1)
table(data$s_CAMA_1_1)
```

```
## 
##  -1   0   1 
## 153 356 547
```

```
data$s_CAMA_1_2 <- coalesce(data$s_CAMA_1_1_2, data$s_CAMA_2_1_2)
table(data$s_CAMA_1_2)
```

```
## 
##  -1   0   1 
## 245 397 413
```

```
data$s_CAMA_1_3 <- coalesce(data$s_CAMA_1_1_3, data$s_CAMA_2_1_3)
table(data$s_CAMA_1_3)
```

```
## 
##  -1   0   1 
## 384 446 229
```

```
data$s_CAMA_1_4 <- coalesce(data$s_CAMA_1_1_4, data$s_CAMA_2_1_4)
table(data$s_CAMA_1_4)
```

```
## 
##  -1   0   1 
## 406 421 226
```

```
data$s_CAMA_1_5 <- coalesce(data$s_CAMA_1_1_5, data$s_CAMA_2_1_5)
table(data$s_CAMA_1_5)
```

```
## 
##  -1   0   1 
## 166 484 405
```

```
data$s_CAMA_1_6 <- coalesce(data$s_CAMA_1_1_6, data$s_CAMA_2_1_6)
table(data$s_CAMA_1_6)
```

```
## 
##  -1   0   1 
## 264 563 224
```

```
data$s_CAMA_1_7 <- coalesce(data$s_CAMA_1_1_7, data$s_CAMA_2_1_7)
table(data$s_CAMA_1_7)
```

```
## 
##  -1   0   1 
## 207 433 416
```

```
data$s_CAMA_1_8 <- coalesce(data$s_CAMA_1_1_8, data$s_CAMA_2_1_8)
table(data$s_CAMA_1_8)
```

```
## 
##  -1   0   1 
## 211 538 309
```

```
data$s_CAMA_2_1 <- coalesce(data$s_CAMA_1_2_1,data$s_CAMA_2_2_1)
table(data$s_CAMA_2_1)
```

```
## 
##  -1   0   1 
## 467 361 230
```

```
data$s_CAMA_2_2 <- coalesce(data$s_CAMA_1_2_2,data$s_CAMA_2_2_2)
table(data$s_CAMA_2_2)
```

```
## 
##  -1   0   1 
## 263 462 332
```

```
data$s_CAMA_2_3 <- coalesce(data$s_CAMA_1_2_3,data$s_CAMA_2_2_3)
table(data$s_CAMA_2_3)
```

```
## 
##  -1   0   1 
## 325 451 283
```

```
data$s_CAMA_2_4 <- coalesce(data$s_CAMA_1_2_1,data$s_CAMA_2_2_4)
table(data$s_CAMA_2_4)
```

```
## 
##  -1   0   1 
## 449 392 218
```

```
data$s_CAMA_3 <- coalesce(data$s_CAMA_1_3, data$s_CAMA_2_3)
table(data$s_CAMA_3)
```

```
## 
##  -1   0   1 
## 385 446 231
```

### Merge and Recode METI

```
data$v_313[data$v_313 == 0] <- NA
data$v_314[data$v_314 == 0] <- NA
data$v_315[data$v_315 == 0] <- NA
data$v_316[data$v_316 == 0] <- NA
data$v_317[data$v_317 == 0] <- NA
data$v_323[data$v_323 == 0] <- NA
data$v_324[data$v_324 == 0] <- NA
data$v_325[data$v_325 == 0] <- NA
data$v_326[data$v_326 == 0] <- NA
data$v_327[data$v_327 == 0] <- NA
data$v_328[data$v_328 == 0] <- NA
data$v_329[data$v_329 == 0] <- NA
data$v_330[data$v_330 == 0] <- NA
data$v_331[data$v_331 == 0] <- NA

data$v_360[data$v_360 == 0] <- NA
data$v_361[data$v_361 == 0] <- NA
data$v_362[data$v_362 == 0] <- NA
data$v_363[data$v_363 == 0] <- NA
data$v_364[data$v_364 == 0] <- NA
data$v_365[data$v_365 == 0] <- NA
data$v_366[data$v_366 == 0] <- NA
data$v_367[data$v_367 == 0] <- NA
data$v_368[data$v_368 == 0] <- NA
data$v_369[data$v_369 == 0] <- NA
data$v_370[data$v_370 == 0] <- NA
data$v_371[data$v_371 == 0] <- NA
data$v_372[data$v_372 == 0] <- NA
data$v_373[data$v_373 == 0] <- NA

data$v_332[data$v_332 == 0] <- NA
data$v_333[data$v_333 == 0] <- NA
data$v_334[data$v_334 == 0] <- NA
data$v_335[data$v_335 == 0] <- NA
data$v_336[data$v_336 == 0] <- NA
data$v_337[data$v_337 == 0] <- NA
data$v_338[data$v_338 == 0] <- NA
data$v_339[data$v_339 == 0] <- NA
data$v_340[data$v_340 == 0] <- NA
data$v_341[data$v_341 == 0] <- NA
data$v_342[data$v_342 == 0] <- NA
data$v_343[data$v_343 == 0] <- NA
data$v_344[data$v_344 == 0] <- NA
data$v_345[data$v_345 == 0] <- NA

data$v_374[data$v_374 == 0] <- NA
data$v_375[data$v_375 == 0] <- NA
data$v_376[data$v_376 == 0] <- NA
data$v_377[data$v_377 == 0] <- NA
data$v_378[data$v_378 == 0] <- NA
data$v_379[data$v_379 == 0] <- NA
data$v_380[data$v_380 == 0] <- NA
data$v_381[data$v_381 == 0] <- NA
data$v_382[data$v_382 == 0] <- NA
data$v_383[data$v_383 == 0] <- NA
data$v_384[data$v_384 == 0] <- NA
data$v_385[data$v_385 == 0] <- NA
data$v_386[data$v_386 == 0] <- NA
data$v_387[data$v_387 == 0] <- NA

data <- rename(data, s_METI_1_Res_exp_1 = v_313, s_METI_1_Res_int_1 = v_314,
               s_METI_1_Res_ben_1 = v_315, s_METI_1_Res_ben_2 = v_316,
               s_METI_1_Res_ben_3 = v_317, s_METI_1_Res_int_2 = v_323, 
               s_METI_1_Res_exp_2 = v_324, s_METI_1_Res_exp_3 = v_325,
               s_METI_1_Res_exp_4 = v_326, s_METI_1_Res_exp_5 = v_327,
               s_METI_1_Res_ben_4 = v_328, s_METI_1_Res_int_3 = v_329,
               s_METI_1_Res_exp_6 = v_330, s_METI_1_Res_int_4 = v_331)

data <- rename(data, s_METI_2_Res_exp_1 = v_360, s_METI_2_Res_int_1 = v_361,
               s_METI_2_Res_ben_1 = v_362, s_METI_2_Res_ben_2 = v_363,
               s_METI_2_Res_ben_3 = v_364, s_METI_2_Res_int_2 = v_365, 
               s_METI_2_Res_exp_2 = v_366, s_METI_2_Res_exp_3 = v_367,
               s_METI_2_Res_exp_4 = v_368, s_METI_2_Res_exp_5 = v_369,
               s_METI_2_Res_ben_4 = v_370, s_METI_2_Res_int_3 = v_371,
               s_METI_2_Res_exp_6 = v_372, s_METI_2_Res_int_4 = v_373)

data <- rename(data, s_METI_1_Auth_exp_1 = v_332, s_METI_1_Auth_int_1 = v_333,
               s_METI_1_Auth_ben_1 = v_334, s_METI_1_Auth_ben_2 = v_335,
               s_METI_1_Auth_ben_3 = v_336, s_METI_1_Auth_int_2 = v_337, 
               s_METI_1_Auth_exp_2 = v_338, s_METI_1_Auth_exp_3 = v_339,
               s_METI_1_Auth_exp_4 = v_340, s_METI_1_Auth_exp_5 = v_341,
               s_METI_1_Auth_ben_4 = v_342, s_METI_1_Auth_int_3 = v_343,
               s_METI_1_Auth_exp_6 = v_344, s_METI_1_Auth_int_4 = v_345)

data <- rename(data, s_METI_2_Auth_exp_1 = v_374, s_METI_2_Auth_int_1 = v_375,
               s_METI_2_Auth_ben_1 = v_376, s_METI_2_Auth_ben_2 = v_377,
               s_METI_2_Auth_ben_3 = v_378, s_METI_2_Auth_int_2 = v_379, 
               s_METI_2_Auth_exp_2 = v_380, s_METI_2_Auth_exp_3 = v_381,
               s_METI_2_Auth_exp_4 = v_382, s_METI_2_Auth_exp_5 = v_383,
               s_METI_2_Auth_ben_4 = v_384, s_METI_2_Auth_int_3 = v_385,
               s_METI_2_Auth_exp_6 = v_386, s_METI_2_Auth_int_4 = v_387)

data$s_METI_1_exp_1 <- coalesce(data$s_METI_1_Res_exp_1,
                                data$s_METI_1_Auth_exp_1)
data$s_METI_1_int_1 <- coalesce(data$s_METI_1_Res_int_1,
                                data$s_METI_1_Auth_int_1)
data$s_METI_1_ben_1 <- coalesce(data$s_METI_1_Res_ben_1,
                                data$s_METI_1_Auth_ben_1)
data$s_METI_1_ben_2 <- coalesce(data$s_METI_1_Res_ben_2,
                                data$s_METI_1_Auth_ben_2)
data$s_METI_1_ben_3 <- coalesce(data$s_METI_1_Res_ben_3,
                                data$s_METI_1_Auth_ben_3)
data$s_METI_1_int_2 <- coalesce(data$s_METI_1_Res_int_2,
                                data$s_METI_1_Auth_int_2)
data$s_METI_1_exp_2 <- coalesce(data$s_METI_1_Res_exp_2,
                                data$s_METI_1_Auth_exp_2)
data$s_METI_1_exp_3 <- coalesce(data$s_METI_1_Res_exp_3,
                                data$s_METI_1_Auth_exp_3)
data$s_METI_1_exp_4 <- coalesce(data$s_METI_1_Res_exp_4,
                                data$s_METI_1_Auth_exp_4)
data$s_METI_1_exp_5 <- coalesce(data$s_METI_1_Res_exp_5,
                                data$s_METI_1_Auth_exp_5)
data$s_METI_1_ben_4 <- coalesce(data$s_METI_1_Res_ben_4,
                                data$s_METI_1_Auth_ben_4)
data$s_METI_1_int_3 <- coalesce(data$s_METI_1_Res_int_3,
                                data$s_METI_1_Auth_int_3)
data$s_METI_1_exp_6 <- coalesce(data$s_METI_1_Res_exp_6,
                                data$s_METI_1_Auth_exp_6)
data$s_METI_1_int_4 <- coalesce(data$s_METI_1_Res_int_4,
                                data$s_METI_1_Auth_int_4)

data$s_METI_2_exp_1 <- coalesce(data$s_METI_2_Res_exp_1,
                                data$s_METI_2_Auth_exp_1)
data$s_METI_2_int_1 <- coalesce(data$s_METI_2_Res_int_1,
                                data$s_METI_2_Auth_int_1)
data$s_METI_2_ben_1 <- coalesce(data$s_METI_2_Res_ben_1,
                                data$s_METI_2_Auth_ben_1)
data$s_METI_2_ben_2 <- coalesce(data$s_METI_2_Res_ben_2,
                                data$s_METI_2_Auth_ben_2)
data$s_METI_2_ben_3 <- coalesce(data$s_METI_2_Res_ben_3,
                                data$s_METI_2_Auth_ben_3)
data$s_METI_2_int_2 <- coalesce(data$s_METI_2_Res_int_2,
                                data$s_METI_2_Auth_int_2)
data$s_METI_2_exp_2 <- coalesce(data$s_METI_2_Res_exp_2,
                                data$s_METI_2_Auth_exp_2)
data$s_METI_2_exp_3 <- coalesce(data$s_METI_2_Res_exp_3,
                                data$s_METI_2_Auth_exp_3)
data$s_METI_2_exp_4 <- coalesce(data$s_METI_2_Res_exp_4,
                                data$s_METI_2_Auth_exp_4)
data$s_METI_2_exp_5 <- coalesce(data$s_METI_2_Res_exp_5,
                                data$s_METI_2_Auth_exp_5)
data$s_METI_2_ben_4 <- coalesce(data$s_METI_2_Res_ben_4,
                                data$s_METI_2_Auth_ben_4)
data$s_METI_2_int_3 <- coalesce(data$s_METI_2_Res_int_3,
                                data$s_METI_2_Auth_int_3)
data$s_METI_2_exp_6 <- coalesce(data$s_METI_2_Res_exp_6,
                                data$s_METI_2_Auth_exp_6)
data$s_METI_2_int_4 <- coalesce(data$s_METI_2_Res_int_4,
                                data$s_METI_2_Auth_int_4)

data$s_METI_exp_1 <- coalesce(data$s_METI_1_exp_1,data$s_METI_2_exp_1)
table(data$s_METI_exp_1)
```

```
## 
##   1   2   3   4   5   6   7 
##  33  42  68 366 362 561 598
```

```
data$s_METI_int_1 <- coalesce(data$s_METI_1_int_1,data$s_METI_2_int_1)
table(data$s_METI_int_1)
```

```
## 
##   1   2   3   4   5   6   7 
##  27  42  62 464 400 548 490
```

```
data$s_METI_ben_1 <- coalesce(data$s_METI_1_ben_1,data$s_METI_2_ben_1)
table(data$s_METI_ben_1)
```

```
## 
##   1   2   3   4   5   6   7 
##  25  34  89 468 399 521 491
```

```
data$s_METI_ben_2 <- coalesce(data$s_METI_1_ben_2,data$s_METI_2_ben_2)
table(data$s_METI_ben_2)
```

```
## 
##   1   2   3   4   5   6   7 
##  37  31  88 444 397 530 504
```

```
data$s_METI_ben_3 <- coalesce(data$s_METI_1_ben_3,data$s_METI_2_ben_3)
table(data$s_METI_ben_3)
```

```
## 
##   1   2   3   4   5   6   7 
##  35  33  76 377 386 558 567
```

```
data$s_METI_int_2 <- coalesce(data$s_METI_1_int_2,data$s_METI_2_int_2)
table(data$s_METI_int_2)
```

```
## 
##   1   2   3   4   5   6   7 
##  37  36  84 428 375 545 523
```

```
data$s_METI_exp_2 <- coalesce(data$s_METI_1_exp_2,data$s_METI_2_exp_2)
table(data$s_METI_exp_2)
```

```
## 
##   1   2   3   4   5   6   7 
##  33  33  72 356 364 587 589
```

```
data$s_METI_exp_3 <- coalesce(data$s_METI_1_exp_3,data$s_METI_2_exp_3)
table(data$s_METI_exp_3)
```

```
## 
##   1   2   3   4   5   6   7 
##  24  51 102 427 398 525 507
```

```
data$s_METI_exp_4 <- coalesce(data$s_METI_1_exp_4,data$s_METI_2_exp_4)
table(data$s_METI_exp_4)
```

```
## 
##   1   2   3   4   5   6   7 
##  27  45  78 385 375 565 560
```

```
data$s_METI_exp_5 <- coalesce(data$s_METI_1_exp_5,data$s_METI_2_exp_5)
table(data$s_METI_exp_5)
```

```
## 
##   1   2   3   4   5   6   7 
##  28  46  72 375 359 593 556
```

```
data$s_METI_ben_4 <- coalesce(data$s_METI_1_ben_4,data$s_METI_2_ben_4)
table(data$s_METI_ben_4)
```

```
## 
##   1   2   3   4   5   6   7 
##  33  36  83 462 402 528 479
```

```
data$s_METI_int_3 <- coalesce(data$s_METI_1_int_3,data$s_METI_2_int_3)
table(data$s_METI_int_3)
```

```
## 
##   1   2   3   4   5   6   7 
##  25  44  83 385 343 581 568
```

```
data$s_METI_exp_6 <- coalesce(data$s_METI_1_exp_6,data$s_METI_2_exp_6)
table(data$s_METI_exp_6)
```

```
## 
##   1   2   3   4   5   6   7 
##  24  33  74 364 370 583 586
```

```
data$s_METI_int_4 <- coalesce(data$s_METI_1_int_4,data$s_METI_2_int_4)
table(data$s_METI_int_4)
```

```
## 
##   1   2   3   4   5   6   7 
##  30  36  86 382 386 561 543
```

### Recode Awareness Check

```
data <- plyr::rename(data, c("v_388" = "s_awareness"))
data$s_awareness <- mapvalues(data$s_awareness, c(0,1,2,3,4,5,6,7,8,9),
                              c(1,0,0,0,0,0,0,0,0,0))
data$s_awareness <- factor(data$s_awareness, c(0,1),
                           labels = c("fail","pass"))
table(data$s_awareness)
```

```
## 
## fail pass 
##  658 1383
```

```
prop.table(table(data$s_awareness))
```

```
## 
##     fail     pass 
## 0.322391 0.677609
```

## Study Duration Analyses

```
data2 <- data[!data$dispcode == 22,]
length(unique(data$p_0001[data$dispcode == 31| data$dispcode == 32]))
```

```
## [1] 2041
```

```
View(data2)

data2$duration_minutes <- data2$duration/60
data2$duration_minutes[data2$duration_minutes <= 0] <- NA

psych::describe(data2$duration_minutes)
```

```
##    vars    n mean    sd median trimmed  mad  min    max range skew kurtosis
## X1    1 1753 21.9 11.91  18.45   19.93 8.06 8.02 104.83 96.82 2.05     6.21
##      se
## X1 0.28
```

```
hist.duration <- ggplot (data2, aes(duration_minutes)) + 
  theme(legend.position = "none") + geom_histogram(aes(y = after_stat(density)),
                                                   colour = "black",
                                                   fill = "white") +
  labs(x = "Duration in Minutes", y = "Density")

hist.duration + stat_function(fun = dnorm,
                              args = list(mean = mean(data2$duration_minutes,
                                                      na.rm = TRUE),
                                          sd = sd(data2$duration_minutes,
                                                  na.rm = TRUE)),
                              colour = "blue", size = 1)
```

```
## Warning: Using `size` aesthetic for lines was deprecated in ggplot2 3.4.0.
## ℹ Please use `linewidth` instead.
## This warning is displayed once every 8 hours.
## Call `lifecycle::last_lifecycle_warnings()` to see where this warning was
## generated.
```

```
## `stat_bin()` using `bins = 30`. Pick better value with `binwidth`.
```

```
## Warning: Removed 288 rows containing non-finite values (`stat_bin()`).
```

### Duration by Condition (Boxplot)

```
conditionBox <- ggplot(data2, aes(condition, duration_minutes)) +
  geom_boxplot() + labs (x = "Condtion", y = "Duration in Minutes")
conditionBox
```

```
## Warning: Removed 288 rows containing non-finite values (`stat_boxplot()`).
```

```
conditionModel <- lm(duration_minutes ~ condition, data = data2)
summary(conditionModel)
```

```
## 
## Call:
## lm(formula = duration_minutes ~ condition, data = data2)
## 
## Residuals:
##     Min      1Q  Median      3Q     Max 
## -15.304  -7.995  -3.432   4.294  81.029 
## 
## Coefficients:
##             Estimate Std. Error t value Pr(>|t|)    
## (Intercept)  20.3892     0.7146  28.534  < 2e-16 ***
## condition2    0.8452     0.9840   0.859 0.390484    
## condition3    0.7425     0.9958   0.746 0.455988    
## condition4    2.5062     0.9933   2.523 0.011722 *  
## condition5    3.4150     1.0143   3.367 0.000776 ***
## condition6    1.6277     0.9840   1.654 0.098248 .  
## ---
## Signif. codes:  0 '***' 0.001 '**' 0.01 '*' 0.05 '.' 0.1 ' ' 1
## 
## Residual standard error: 11.87 on 1747 degrees of freedom
##   (288 Beobachtungen als fehlend gelöscht)
## Multiple R-squared:  0.008949,   Adjusted R-squared:  0.006113 
## F-statistic: 3.155 on 5 and 1747 DF,  p-value: 0.007696
```

### Duration by Quota (Boxplot)

```
quotaBox <- ggplot(data2, aes(quota, duration_minutes)) +
  geom_boxplot() + labs (x = "Quota", y = "Duration in Minutes")
quotaBox
```

```
## Warning: Removed 288 rows containing non-finite values (`stat_boxplot()`).
```

```
quotaModel <- lm(duration_minutes ~ quota, data = data2)
summary(quotaModel)
```

```
## 
## Call:
## lm(formula = duration_minutes ~ quota, data = data2)
## 
## Residuals:
##     Min      1Q  Median      3Q     Max 
## -15.507  -7.772  -3.212   4.205  79.393 
## 
## Coefficients:
##             Estimate Std. Error t value Pr(>|t|)    
## (Intercept)  24.5913     0.9541  25.773  < 2e-16 ***
## quota2       -3.3109     1.3342  -2.481 0.013178 *  
## quota3       -1.5023     1.3586  -1.106 0.268974    
## quota4        0.8492     1.3810   0.615 0.538703    
## quota5        0.2674     1.3610   0.196 0.844256    
## quota6       -2.2524     1.3384  -1.683 0.092582 .  
## quota7       -5.8959     1.3610  -4.332 1.56e-05 ***
## quota8       -4.7178     1.3494  -3.496 0.000484 ***
## quota9       -6.6560     1.3733  -4.847 1.37e-06 ***
## quota10      -1.6808     1.3758  -1.222 0.221997    
## quota11      -1.3459     1.3562  -0.992 0.321162    
## quota12      -6.0378     1.3682  -4.413 1.08e-05 ***
## ---
## Signif. codes:  0 '***' 0.001 '**' 0.01 '*' 0.05 '.' 0.1 ' ' 1
## 
## Residual standard error: 11.69 on 1741 degrees of freedom
##   (288 Beobachtungen als fehlend gelöscht)
## Multiple R-squared:  0.04297,    Adjusted R-squared:  0.03692 
## F-statistic: 7.106 on 11 and 1741 DF,  p-value: 6.521e-12
```

### Duration by Awareness Check (Boxplot)

```
summary(data2$s_awareness)
```

```
## fail pass 
##  658 1383
```

```
awarenessBox <- ggplot(data = data2, aes(s_awareness, duration_minutes)) +
  geom_boxplot() + labs(x = "Awarenes Check", y = "Duration in Minutes")
awarenessBox
```

```
## Warning: Removed 288 rows containing non-finite values (`stat_boxplot()`).
```

```
awarenessModel <- lm(duration_minutes ~ s_awareness, data = data2)
summary(awarenessModel)
```

```
## 
## Call:
## lm(formula = duration_minutes ~ s_awareness, data = data2)
## 
## Residuals:
##     Min      1Q  Median      3Q     Max 
## -14.837  -7.821  -3.371   3.996  81.213 
## 
## Coefficients:
##                 Estimate Std. Error t value Pr(>|t|)    
## (Intercept)      18.3032     0.4892  37.416   <2e-16 ***
## s_awarenesspass   5.3176     0.5947   8.941   <2e-16 ***
## ---
## Signif. codes:  0 '***' 0.001 '**' 0.01 '*' 0.05 '.' 0.1 ' ' 1
## 
## Residual standard error: 11.65 on 1751 degrees of freedom
##   (288 Beobachtungen als fehlend gelöscht)
## Multiple R-squared:  0.04366,    Adjusted R-squared:  0.04312 
## F-statistic: 79.95 on 1 and 1751 DF,  p-value: < 2.2e-16
```

### Duration by Age (Scatterplot)

```
describe(data$s_age)
```

```
##    vars    n  mean    sd median trimmed   mad min max range  skew kurtosis   se
## X1    1 3077 47.46 15.89     48   47.47 19.27  18  90    72 -0.01    -0.99 0.29
```

```
describe(data2$s_age)
```

```
##    vars    n  mean    sd median trimmed   mad min max range skew kurtosis   se
## X1    1 2040 45.22 15.23     45   45.01 17.79  18  90    72 0.12    -0.96 0.34
```

```
scatter.age <- ggplot(data2, aes(s_age,duration_minutes)) +
  geom_point() + geom_smooth(method = "lm", se = F) + 
  labs(x = "Age", y = "Duration in minutes")
scatter.age
```

```
## `geom_smooth()` using formula = 'y ~ x'
```

```
## Warning: Removed 289 rows containing non-finite values (`stat_smooth()`).
```

```
## Warning: Removed 289 rows containing missing values (`geom_point()`).
```

```
cor.test(data2$s_age, data2$duration_minutes)
```

```
## 
##  Pearson's product-moment correlation
## 
## data:  data2$s_age and data2$duration_minutes
## t = 6.5843, df = 1750, p-value = 6.025e-11
## alternative hypothesis: true correlation is not equal to 0
## 95 percent confidence interval:
##  0.1094461 0.2008492
## sample estimates:
##       cor 
## 0.1554804
```

### Duration by Gender (Boxplot)

```
genderBox <- ggplot(data = data2, aes(s_sex, duration_minutes)) +
  geom_boxplot() + labs(x = "Subject Gender", y = "Duration in Minutes")
genderBox
```

```
## Warning: Removed 288 rows containing non-finite values (`stat_boxplot()`).
```

```
genderModel <- lm(duration_minutes ~ s_sex, data = data2)
summary(genderModel)
```

```
## 
## Call:
## lm(formula = duration_minutes ~ s_sex, data = data2)
## 
## Residuals:
##     Min      1Q  Median      3Q     Max 
## -14.377  -7.994  -3.410   4.273  81.956 
## 
## Coefficients:
##             Estimate Std. Error t value Pr(>|t|)    
## (Intercept)  22.8771     0.4037  56.674  < 2e-16 ***
## s_sexmale    -1.9271     0.5672  -3.398 0.000694 ***
## ---
## Signif. codes:  0 '***' 0.001 '**' 0.01 '*' 0.05 '.' 0.1 ' ' 1
## 
## Residual standard error: 11.87 on 1751 degrees of freedom
##   (288 Beobachtungen als fehlend gelöscht)
## Multiple R-squared:  0.00655,    Adjusted R-squared:  0.005983 
## F-statistic: 11.55 on 1 and 1751 DF,  p-value: 0.0006944
```

### Duration by Educational Background (Boxplot)

```
schoolBox <- ggplot(data = data2, aes(s_school, duration_minutes)) +
  geom_boxplot() +labs(x = "Education Level", y = "Duration in Minutes")
schoolBox
```

```
## Warning: Removed 288 rows containing non-finite values (`stat_boxplot()`).
```

```
schoolModel <- lm(duration_minutes ~ s_school, data = data2)
summary(schoolModel)
```

```
## 
## Call:
## lm(formula = duration_minutes ~ s_school, data = data2)
## 
## Residuals:
##     Min      1Q  Median      3Q     Max 
## -14.872  -8.014  -3.464   4.473  81.945 
## 
## Coefficients:
##              Estimate Std. Error t value Pr(>|t|)    
## (Intercept)   20.5473     0.4917  41.784  < 2e-16 ***
## s_schoolReal   1.7293     0.6908   2.503 0.012393 *  
## s_schoolAbi    2.3414     0.6991   3.349 0.000828 ***
## ---
## Signif. codes:  0 '***' 0.001 '**' 0.01 '*' 0.05 '.' 0.1 ' ' 1
## 
## Residual standard error: 11.87 on 1750 degrees of freedom
##   (288 Beobachtungen als fehlend gelöscht)
## Multiple R-squared:  0.006883,   Adjusted R-squared:  0.005748 
## F-statistic: 6.064 on 2 and 1750 DF,  p-value: 0.002374
```

## Clean Wide-Format Dataset

```
names(data)
```

```
##   [1] "id"                  "external_lfdn"       "tester"             
##   [4] "dispcode"            "lastpage"            "quality"            
##   [7] "duration"            "condition"           "p_0001"             
##  [10] "c_0002"              "text_order"          "METI_target"        
##  [13] "s_sex"               "s_age"               "s_school"           
##  [16] "s_german"            "s_psychology"        "s_interest"         
##  [19] "s_contact"           "s_field"             "v_10"               
##  [22] "v_11"                "v_47"                "v_48"               
##  [25] "v_49"                "v_12"                "v_14"               
##  [28] "v_16"                "v_71"                "v_17"               
##  [31] "v_18"                "v_19"                "v_20"               
##  [34] "v_21"                "v_115"               "v_116"              
##  [37] "v_117"               "v_22"                "v_23"               
##  [40] "v_24"                "v_25"                "v_26"               
##  [43] "v_120"               "v_27"                "v_28"               
##  [46] "v_29"                "v_30"                "v_31"               
##  [49] "v_121"               "v_32"                "v_33"               
##  [52] "v_34"                "v_35"                "v_36"               
##  [55] "v_122"               "v_37"                "v_38"               
##  [58] "v_39"                "v_40"                "v_41"               
##  [61] "v_123"               "v_124"               "v_42"               
##  [64] "v_43"                "v_44"                "v_45"               
##  [67] "v_46"                "v_125"               "v_72"               
##  [70] "v_73"                "v_74"                "v_75"               
##  [73] "v_76"                "v_77"                "v_79"               
##  [76] "v_81"                "v_83"                "v_126"              
##  [79] "v_127"               "v_128"               "v_129"              
##  [82] "v_130"               "v_131"               "v_132"              
##  [85] "v_133"               "v_134"               "v_135"              
##  [88] "v_136"               "v_137"               "v_138"              
##  [91] "v_139"               "v_140"               "v_141"              
##  [94] "v_142"               "v_143"               "v_144"              
##  [97] "v_145"               "v_146"               "v_147"              
## [100] "v_148"               "v_149"               "v_150"              
## [103] "v_151"               "v_152"               "v_153"              
## [106] "v_154"               "v_155"               "v_156"              
## [109] "v_157"               "v_158"               "v_159"              
## [112] "v_160"               "v_161"               "v_162"              
## [115] "v_163"               "v_164"               "s_CAMA_1_1_1"       
## [118] "s_CAMA_1_1_2"        "s_CAMA_1_1_3"        "s_CAMA_1_1_4"       
## [121] "s_CAMA_1_1_5"        "s_CAMA_1_1_6"        "s_CAMA_1_1_7"       
## [124] "s_CAMA_1_1_8"        "s_CAMA_1_2_1"        "s_CAMA_1_2_2"       
## [127] "s_CAMA_1_2_3"        "s_CAMA_1_2_4"        "v_401"              
## [130] "v_91"                "v_92"                "v_93"               
## [133] "v_94"                "v_95"                "v_96"               
## [136] "v_98"                "v_100"               "v_102"              
## [139] "v_235"               "v_236"               "v_237"              
## [142] "v_238"               "v_239"               "v_240"              
## [145] "v_241"               "v_242"               "v_243"              
## [148] "v_244"               "v_245"               "v_246"              
## [151] "v_247"               "v_248"               "v_249"              
## [154] "v_250"               "v_251"               "v_252"              
## [157] "v_253"               "v_254"               "v_255"              
## [160] "v_256"               "v_257"               "v_258"              
## [163] "v_259"               "s_METI_1_Res_exp_1"  "s_METI_1_Res_int_1" 
## [166] "s_METI_1_Res_ben_1"  "s_METI_1_Res_ben_2"  "s_METI_1_Res_ben_3" 
## [169] "s_METI_1_Res_int_2"  "s_METI_1_Res_exp_2"  "s_METI_1_Res_exp_3" 
## [172] "s_METI_1_Res_exp_4"  "s_METI_1_Res_exp_5"  "s_METI_1_Res_ben_4" 
## [175] "s_METI_1_Res_int_3"  "s_METI_1_Res_exp_6"  "s_METI_1_Res_int_4" 
## [178] "s_METI_1_Auth_exp_1" "s_METI_1_Auth_int_1" "s_METI_1_Auth_ben_1"
## [181] "s_METI_1_Auth_ben_2" "s_METI_1_Auth_ben_3" "s_METI_1_Auth_int_2"
## [184] "s_METI_1_Auth_exp_2" "s_METI_1_Auth_exp_3" "s_METI_1_Auth_exp_4"
## [187] "s_METI_1_Auth_exp_5" "s_METI_1_Auth_ben_4" "s_METI_1_Auth_int_3"
## [190] "s_METI_1_Auth_exp_6" "s_METI_1_Auth_int_4" "v_103"              
## [193] "v_104"               "v_105"               "v_106"              
## [196] "v_107"               "v_108"               "v_110"              
## [199] "v_112"               "v_114"               "v_274"              
## [202] "v_275"               "v_276"               "v_277"              
## [205] "v_278"               "v_279"               "v_280"              
## [208] "v_281"               "v_282"               "v_283"              
## [211] "v_284"               "v_285"               "v_286"              
## [214] "v_287"               "v_288"               "v_289"              
## [217] "v_290"               "v_291"               "v_292"              
## [220] "v_293"               "v_294"               "v_295"              
## [223] "v_296"               "v_297"               "v_298"              
## [226] "s_CAMA_2_1_1"        "s_CAMA_2_1_2"        "s_CAMA_2_1_3"       
## [229] "s_CAMA_2_1_4"        "s_CAMA_2_1_5"        "s_CAMA_2_1_6"       
## [232] "s_CAMA_2_1_7"        "s_CAMA_2_1_8"        "s_CAMA_2_2_1"       
## [235] "s_CAMA_2_2_2"        "s_CAMA_2_2_3"        "s_CAMA_2_2_4"       
## [238] "v_402"               "s_METI_2_Res_exp_1"  "s_METI_2_Res_int_1" 
## [241] "s_METI_2_Res_ben_1"  "s_METI_2_Res_ben_2"  "s_METI_2_Res_ben_3" 
## [244] "s_METI_2_Res_int_2"  "s_METI_2_Res_exp_2"  "s_METI_2_Res_exp_3" 
## [247] "s_METI_2_Res_exp_4"  "s_METI_2_Res_exp_5"  "s_METI_2_Res_ben_4" 
## [250] "s_METI_2_Res_int_3"  "s_METI_2_Res_exp_6"  "s_METI_2_Res_int_4" 
## [253] "s_METI_2_Auth_exp_1" "s_METI_2_Auth_int_1" "s_METI_2_Auth_ben_1"
## [256] "s_METI_2_Auth_ben_2" "s_METI_2_Auth_ben_3" "s_METI_2_Auth_int_2"
## [259] "s_METI_2_Auth_exp_2" "s_METI_2_Auth_exp_3" "s_METI_2_Auth_exp_4"
## [262] "s_METI_2_Auth_exp_5" "s_METI_2_Auth_ben_4" "s_METI_2_Auth_int_3"
## [265] "s_METI_2_Auth_exp_6" "s_METI_2_Auth_int_4" "s_awareness"        
## [268] "browser"             "referer"             "device_type"        
## [271] "quota"               "quota_assignment"    "quota_rejected_id"  
## [274] "page_history"        "hflip"               "vflip"              
## [277] "output_mode"         "javascript"          "flash"              
## [280] "session_id"          "language"            "cleaned"            
## [283] "ats"                 "datetime"            "date_of_last_access"
## [286] "date_of_first_mail"  "rts6018385"          "rts6018739"         
## [289] "rts6018818"          "rts6019080"          "rts6019089"         
## [292] "rts6021451"          "rts6021455"          "rts6023513"         
## [295] "rts6023515"          "rts6023627"          "rts6023655"         
## [298] "rts6023657"          "rts6023660"          "rts6023667"         
## [301] "rts6023676"          "rts6023679"          "rts6033975"         
## [304] "METI_text"           "summary1"            "summary2"           
## [307] "version"             "causality"           "disclaimer"         
## [310] "CAMA"                "dropout"             "accessibility_1"    
## [313] "accessibility_2"     "understanding_1"     "understanding_2"    
## [316] "empowerment_1"       "empowerment_2"       "credibility_1"      
## [319] "credibility_2"       "relevance_1"         "relevance_2"        
## [322] "curiosity_1"         "curiosity_2"         "boredom_1"          
## [325] "boredom_2"           "confusion_1"         "confusion_2"        
## [328] "frustration_1"       "frustration_2"       "s_relationship_1"   
## [331] "s_relationship_2"    "s_relationship_3"    "s_relationship_4"   
## [334] "s_relationship_5"    "s_relationship_6"    "s_relationship_7"   
## [337] "s_relationship_8"    "s_extent_1"          "s_extent_2"         
## [340] "s_extent_3"          "s_extent_4"          "s_extent_5"         
## [343] "s_extent_6"          "s_diff_1_1"          "s_diff_1_2"         
## [346] "s_diff_1_3"          "s_diff_1_4"          "s_diff_1_5"         
## [349] "s_diff_1_6"          "s_diff_2_1"          "s_diff_2_2"         
## [352] "s_diff_2_3"          "s_diff_2_4"          "s_diff_2_5"         
## [355] "s_diff_2_6"          "s_funding_1_1"       "s_funding_1_2"      
## [358] "s_funding_1_3"       "s_funding_1_4"       "s_funding_1_5"      
## [361] "s_funding_1_6"       "s_funding_2_1"       "s_funding_2_2"      
## [364] "s_funding_2_3"       "s_funding_2_4"       "s_funding_2_5"      
## [367] "s_funding_2_6"       "s_coi_1_1"           "s_coi_1_2"          
## [370] "s_coi_1_3"           "s_coi_1_4"           "s_coi_1_5"          
## [373] "s_coi_1_6"           "s_coi_1_7"           "s_coi_2_1"          
## [376] "s_coi_2_2"           "s_coi_2_3"           "s_coi_2_4"          
## [379] "s_coi_2_5"           "s_coi_2_6"           "s_coi_2_7"          
## [382] "s_causality_1_1"     "s_causality_1_2"     "s_causality_1_3"    
## [385] "s_causality_1_4"     "s_causality_1_5"     "s_causality_1_6"    
## [388] "s_causality_2_1"     "s_causality_2_2"     "s_causality_2_3"    
## [391] "s_causality_2_4"     "s_causality_2_5"     "s_causality_2_6"    
## [394] "s_CAMA_1_3"          "s_CAMA_2_3"          "s_CAMA_1_1"         
## [397] "s_CAMA_1_2"          "s_CAMA_1_4"          "s_CAMA_1_5"         
## [400] "s_CAMA_1_6"          "s_CAMA_1_7"          "s_CAMA_1_8"         
## [403] "s_CAMA_2_1"          "s_CAMA_2_2"          "s_CAMA_2_4"         
## [406] "s_CAMA_3"            "s_METI_1_exp_1"      "s_METI_1_int_1"     
## [409] "s_METI_1_ben_1"      "s_METI_1_ben_2"      "s_METI_1_ben_3"     
## [412] "s_METI_1_int_2"      "s_METI_1_exp_2"      "s_METI_1_exp_3"     
## [415] "s_METI_1_exp_4"      "s_METI_1_exp_5"      "s_METI_1_ben_4"     
## [418] "s_METI_1_int_3"      "s_METI_1_exp_6"      "s_METI_1_int_4"     
## [421] "s_METI_2_exp_1"      "s_METI_2_int_1"      "s_METI_2_ben_1"     
## [424] "s_METI_2_ben_2"      "s_METI_2_ben_3"      "s_METI_2_int_2"     
## [427] "s_METI_2_exp_2"      "s_METI_2_exp_3"      "s_METI_2_exp_4"     
## [430] "s_METI_2_exp_5"      "s_METI_2_ben_4"      "s_METI_2_int_3"     
## [433] "s_METI_2_exp_6"      "s_METI_2_int_4"      "s_METI_exp_1"       
## [436] "s_METI_int_1"        "s_METI_ben_1"        "s_METI_ben_2"       
## [439] "s_METI_ben_3"        "s_METI_int_2"        "s_METI_exp_2"       
## [442] "s_METI_exp_3"        "s_METI_exp_4"        "s_METI_exp_5"       
## [445] "s_METI_ben_4"        "s_METI_int_3"        "s_METI_exp_6"       
## [448] "s_METI_int_4"
```

```
data_wide <- data[,!names(data) %in% c("external_lfdn","tester","lastpage",
                                       "quality","p_0001","c_0002","browser",
                                       "referer","device_type",
                                       "quota_assignment","quota_rejected_id",
                                       "page_history","hflip","vflip",
                                       "output_mode","javascript","flash",
                                       "session_id","language","cleaned","ats",
                                       "datetime","date_of_last_access",
                                       "day_of_first_mail","rts6018385",
                                       "rts6018739","rts6018818","rts6019080",
                                       "rts6019089","rts6021451","rts6021455",
                                       "rts6023513","rts6023515","rts6023627",
                                       "rts6023655","rts6023657","rts6023660",
                                       "rts6023667","rts6023676","rts6023679",
                                       "rts6033975")]
names(data_wide)
```

```
##   [1] "id"                  "dispcode"            "duration"           
##   [4] "condition"           "text_order"          "METI_target"        
##   [7] "s_sex"               "s_age"               "s_school"           
##  [10] "s_german"            "s_psychology"        "s_interest"         
##  [13] "s_contact"           "s_field"             "v_10"               
##  [16] "v_11"                "v_47"                "v_48"               
##  [19] "v_49"                "v_12"                "v_14"               
##  [22] "v_16"                "v_71"                "v_17"               
##  [25] "v_18"                "v_19"                "v_20"               
##  [28] "v_21"                "v_115"               "v_116"              
##  [31] "v_117"               "v_22"                "v_23"               
##  [34] "v_24"                "v_25"                "v_26"               
##  [37] "v_120"               "v_27"                "v_28"               
##  [40] "v_29"                "v_30"                "v_31"               
##  [43] "v_121"               "v_32"                "v_33"               
##  [46] "v_34"                "v_35"                "v_36"               
##  [49] "v_122"               "v_37"                "v_38"               
##  [52] "v_39"                "v_40"                "v_41"               
##  [55] "v_123"               "v_124"               "v_42"               
##  [58] "v_43"                "v_44"                "v_45"               
##  [61] "v_46"                "v_125"               "v_72"               
##  [64] "v_73"                "v_74"                "v_75"               
##  [67] "v_76"                "v_77"                "v_79"               
##  [70] "v_81"                "v_83"                "v_126"              
##  [73] "v_127"               "v_128"               "v_129"              
##  [76] "v_130"               "v_131"               "v_132"              
##  [79] "v_133"               "v_134"               "v_135"              
##  [82] "v_136"               "v_137"               "v_138"              
##  [85] "v_139"               "v_140"               "v_141"              
##  [88] "v_142"               "v_143"               "v_144"              
##  [91] "v_145"               "v_146"               "v_147"              
##  [94] "v_148"               "v_149"               "v_150"              
##  [97] "v_151"               "v_152"               "v_153"              
## [100] "v_154"               "v_155"               "v_156"              
## [103] "v_157"               "v_158"               "v_159"              
## [106] "v_160"               "v_161"               "v_162"              
## [109] "v_163"               "v_164"               "s_CAMA_1_1_1"       
## [112] "s_CAMA_1_1_2"        "s_CAMA_1_1_3"        "s_CAMA_1_1_4"       
## [115] "s_CAMA_1_1_5"        "s_CAMA_1_1_6"        "s_CAMA_1_1_7"       
## [118] "s_CAMA_1_1_8"        "s_CAMA_1_2_1"        "s_CAMA_1_2_2"       
## [121] "s_CAMA_1_2_3"        "s_CAMA_1_2_4"        "v_401"              
## [124] "v_91"                "v_92"                "v_93"               
## [127] "v_94"                "v_95"                "v_96"               
## [130] "v_98"                "v_100"               "v_102"              
## [133] "v_235"               "v_236"               "v_237"              
## [136] "v_238"               "v_239"               "v_240"              
## [139] "v_241"               "v_242"               "v_243"              
## [142] "v_244"               "v_245"               "v_246"              
## [145] "v_247"               "v_248"               "v_249"              
## [148] "v_250"               "v_251"               "v_252"              
## [151] "v_253"               "v_254"               "v_255"              
## [154] "v_256"               "v_257"               "v_258"              
## [157] "v_259"               "s_METI_1_Res_exp_1"  "s_METI_1_Res_int_1" 
## [160] "s_METI_1_Res_ben_1"  "s_METI_1_Res_ben_2"  "s_METI_1_Res_ben_3" 
## [163] "s_METI_1_Res_int_2"  "s_METI_1_Res_exp_2"  "s_METI_1_Res_exp_3" 
## [166] "s_METI_1_Res_exp_4"  "s_METI_1_Res_exp_5"  "s_METI_1_Res_ben_4" 
## [169] "s_METI_1_Res_int_3"  "s_METI_1_Res_exp_6"  "s_METI_1_Res_int_4" 
## [172] "s_METI_1_Auth_exp_1" "s_METI_1_Auth_int_1" "s_METI_1_Auth_ben_1"
## [175] "s_METI_1_Auth_ben_2" "s_METI_1_Auth_ben_3" "s_METI_1_Auth_int_2"
## [178] "s_METI_1_Auth_exp_2" "s_METI_1_Auth_exp_3" "s_METI_1_Auth_exp_4"
## [181] "s_METI_1_Auth_exp_5" "s_METI_1_Auth_ben_4" "s_METI_1_Auth_int_3"
## [184] "s_METI_1_Auth_exp_6" "s_METI_1_Auth_int_4" "v_103"              
## [187] "v_104"               "v_105"               "v_106"              
## [190] "v_107"               "v_108"               "v_110"              
## [193] "v_112"               "v_114"               "v_274"              
## [196] "v_275"               "v_276"               "v_277"              
## [199] "v_278"               "v_279"               "v_280"              
## [202] "v_281"               "v_282"               "v_283"              
## [205] "v_284"               "v_285"               "v_286"              
## [208] "v_287"               "v_288"               "v_289"              
## [211] "v_290"               "v_291"               "v_292"              
## [214] "v_293"               "v_294"               "v_295"              
## [217] "v_296"               "v_297"               "v_298"              
## [220] "s_CAMA_2_1_1"        "s_CAMA_2_1_2"        "s_CAMA_2_1_3"       
## [223] "s_CAMA_2_1_4"        "s_CAMA_2_1_5"        "s_CAMA_2_1_6"       
## [226] "s_CAMA_2_1_7"        "s_CAMA_2_1_8"        "s_CAMA_2_2_1"       
## [229] "s_CAMA_2_2_2"        "s_CAMA_2_2_3"        "s_CAMA_2_2_4"       
## [232] "v_402"               "s_METI_2_Res_exp_1"  "s_METI_2_Res_int_1" 
## [235] "s_METI_2_Res_ben_1"  "s_METI_2_Res_ben_2"  "s_METI_2_Res_ben_3" 
## [238] "s_METI_2_Res_int_2"  "s_METI_2_Res_exp_2"  "s_METI_2_Res_exp_3" 
## [241] "s_METI_2_Res_exp_4"  "s_METI_2_Res_exp_5"  "s_METI_2_Res_ben_4" 
## [244] "s_METI_2_Res_int_3"  "s_METI_2_Res_exp_6"  "s_METI_2_Res_int_4" 
## [247] "s_METI_2_Auth_exp_1" "s_METI_2_Auth_int_1" "s_METI_2_Auth_ben_1"
## [250] "s_METI_2_Auth_ben_2" "s_METI_2_Auth_ben_3" "s_METI_2_Auth_int_2"
## [253] "s_METI_2_Auth_exp_2" "s_METI_2_Auth_exp_3" "s_METI_2_Auth_exp_4"
## [256] "s_METI_2_Auth_exp_5" "s_METI_2_Auth_ben_4" "s_METI_2_Auth_int_3"
## [259] "s_METI_2_Auth_exp_6" "s_METI_2_Auth_int_4" "s_awareness"        
## [262] "quota"               "date_of_first_mail"  "METI_text"          
## [265] "summary1"            "summary2"            "version"            
## [268] "causality"           "disclaimer"          "CAMA"               
## [271] "dropout"             "accessibility_1"     "accessibility_2"    
## [274] "understanding_1"     "understanding_2"     "empowerment_1"      
## [277] "empowerment_2"       "credibility_1"       "credibility_2"      
## [280] "relevance_1"         "relevance_2"         "curiosity_1"        
## [283] "curiosity_2"         "boredom_1"           "boredom_2"          
## [286] "confusion_1"         "confusion_2"         "frustration_1"      
## [289] "frustration_2"       "s_relationship_1"    "s_relationship_2"   
## [292] "s_relationship_3"    "s_relationship_4"    "s_relationship_5"   
## [295] "s_relationship_6"    "s_relationship_7"    "s_relationship_8"   
## [298] "s_extent_1"          "s_extent_2"          "s_extent_3"         
## [301] "s_extent_4"          "s_extent_5"          "s_extent_6"         
## [304] "s_diff_1_1"          "s_diff_1_2"          "s_diff_1_3"         
## [307] "s_diff_1_4"          "s_diff_1_5"          "s_diff_1_6"         
## [310] "s_diff_2_1"          "s_diff_2_2"          "s_diff_2_3"         
## [313] "s_diff_2_4"          "s_diff_2_5"          "s_diff_2_6"         
## [316] "s_funding_1_1"       "s_funding_1_2"       "s_funding_1_3"      
## [319] "s_funding_1_4"       "s_funding_1_5"       "s_funding_1_6"      
## [322] "s_funding_2_1"       "s_funding_2_2"       "s_funding_2_3"      
## [325] "s_funding_2_4"       "s_funding_2_5"       "s_funding_2_6"      
## [328] "s_coi_1_1"           "s_coi_1_2"           "s_coi_1_3"          
## [331] "s_coi_1_4"           "s_coi_1_5"           "s_coi_1_6"          
## [334] "s_coi_1_7"           "s_coi_2_1"           "s_coi_2_2"          
## [337] "s_coi_2_3"           "s_coi_2_4"           "s_coi_2_5"          
## [340] "s_coi_2_6"           "s_coi_2_7"           "s_causality_1_1"    
## [343] "s_causality_1_2"     "s_causality_1_3"     "s_causality_1_4"    
## [346] "s_causality_1_5"     "s_causality_1_6"     "s_causality_2_1"    
## [349] "s_causality_2_2"     "s_causality_2_3"     "s_causality_2_4"    
## [352] "s_causality_2_5"     "s_causality_2_6"     "s_CAMA_1_3"         
## [355] "s_CAMA_2_3"          "s_CAMA_1_1"          "s_CAMA_1_2"         
## [358] "s_CAMA_1_4"          "s_CAMA_1_5"          "s_CAMA_1_6"         
## [361] "s_CAMA_1_7"          "s_CAMA_1_8"          "s_CAMA_2_1"         
## [364] "s_CAMA_2_2"          "s_CAMA_2_4"          "s_CAMA_3"           
## [367] "s_METI_1_exp_1"      "s_METI_1_int_1"      "s_METI_1_ben_1"     
## [370] "s_METI_1_ben_2"      "s_METI_1_ben_3"      "s_METI_1_int_2"     
## [373] "s_METI_1_exp_2"      "s_METI_1_exp_3"      "s_METI_1_exp_4"     
## [376] "s_METI_1_exp_5"      "s_METI_1_ben_4"      "s_METI_1_int_3"     
## [379] "s_METI_1_exp_6"      "s_METI_1_int_4"      "s_METI_2_exp_1"     
## [382] "s_METI_2_int_1"      "s_METI_2_ben_1"      "s_METI_2_ben_2"     
## [385] "s_METI_2_ben_3"      "s_METI_2_int_2"      "s_METI_2_exp_2"     
## [388] "s_METI_2_exp_3"      "s_METI_2_exp_4"      "s_METI_2_exp_5"     
## [391] "s_METI_2_ben_4"      "s_METI_2_int_3"      "s_METI_2_exp_6"     
## [394] "s_METI_2_int_4"      "s_METI_exp_1"        "s_METI_int_1"       
## [397] "s_METI_ben_1"        "s_METI_ben_2"        "s_METI_ben_3"       
## [400] "s_METI_int_2"        "s_METI_exp_2"        "s_METI_exp_3"       
## [403] "s_METI_exp_4"        "s_METI_exp_5"        "s_METI_ben_4"       
## [406] "s_METI_int_3"        "s_METI_exp_6"        "s_METI_int_4"
```

```
str(data_wide)
```

```
## 'data.frame':    3080 obs. of  408 variables:
##  $ id                 : Factor w/ 6705 levels "1","2","3","4",..: 4692 193 4223 5452 5207 3121 4700 3926 6230 6074 ...
##  $ dispcode           : int  31 22 22 31 22 31 31 31 31 31 ...
##  $ duration           : int  779 68 19 1043 36 546 746 938 568 1094 ...
##  $ condition          : Factor w/ 6 levels "1","2","3","4",..: 3 2 NA 4 5 3 5 6 2 1 ...
##  $ text_order         : Factor w/ 2 levels "Barth","Faerber": 1 2 NA 2 2 1 2 1 1 2 ...
##  $ METI_target        : Factor w/ 2 levels "Study Authors",..: 2 2 NA 1 2 2 1 2 1 2 ...
##  $ s_sex              : Factor w/ 2 levels "female","male": 2 2 2 1 2 2 1 2 1 1 ...
##  $ s_age              : int  31 42 25 31 47 54 45 43 51 57 ...
##  $ s_school           : Factor w/ 3 levels "Haupt","Real",..: 2 2 1 1 1 1 2 3 2 3 ...
##  $ s_german           : int  1 1 1 1 1 1 1 1 1 1 ...
##  $ s_psychology       : int  2 2 2 2 2 2 2 2 2 2 ...
##  $ s_interest         : int  5 6 5 5 5 5 4 7 7 8 ...
##  $ s_contact          : int  1 1 2 1 1 1 2 2 5 3 ...
##  $ s_field            : chr  NA NA NA NA ...
##  $ v_10               : int  4 NA NA NA NA 3 NA 5 NA NA ...
##  $ v_11               : int  6 NA NA NA NA 2 NA 7 6 NA ...
##  $ v_47               : int  3 NA NA NA NA 3 NA 7 6 NA ...
##  $ v_48               : int  6 NA NA NA NA 3 NA 7 NA NA ...
##  $ v_49               : int  6 NA NA NA NA 5 NA 7 NA NA ...
##  $ v_12               : int  2 NA NA NA NA 3 NA 4 2 NA ...
##  $ v_14               : int  1 NA NA NA NA 2 NA 1 4 NA ...
##  $ v_16               : int  3 NA NA NA NA 4 NA 1 2 NA ...
##  $ v_71               : int  3 NA NA NA NA 2 NA 1 3 NA ...
##  $ v_17               : int  1 NA NA NA NA 3 NA 1 2 NA ...
##  $ v_18               : int  1 NA NA NA NA 3 NA 1 1 NA ...
##  $ v_19               : int  2 NA NA NA NA 3 NA 1 3 NA ...
##  $ v_20               : int  2 NA NA NA NA 3 NA 1 2 NA ...
##  $ v_21               : int  1 NA NA NA NA 3 NA 1 1 NA ...
##  $ v_115              : int  1 NA NA NA NA 3 NA 1 NA NA ...
##  $ v_116              : int  1 NA NA NA NA 3 NA 1 1 NA ...
##  $ v_117              : int  1 NA NA NA NA 3 NA 1 1 NA ...
##  $ v_22               : int  3 NA NA NA NA 3 NA 1 3 NA ...
##  $ v_23               : int  1 NA NA NA NA 3 NA 1 1 NA ...
##  $ v_24               : int  1 NA NA NA NA 3 NA 3 2 NA ...
##  $ v_25               : int  1 NA NA NA NA 3 NA 1 1 NA ...
##  $ v_26               : int  1 NA NA NA NA 3 NA 1 3 NA ...
##  $ v_120              : int  1 NA NA NA NA 3 NA 1 2 NA ...
##  $ v_27               : num  1 NA NA NA NA 0 NA -1 -1 NA ...
##  $ v_28               : num  1 NA NA NA NA 0 NA -1 1 NA ...
##  $ v_29               : num  -1 NA NA NA NA 0 NA 1 -1 NA ...
##  $ v_30               : num  1 NA NA NA NA 0 NA -1 1 NA ...
##  $ v_31               : num  1 NA NA NA NA 0 NA 1 0 NA ...
##  $ v_121              : num  1 NA NA NA NA 0 NA 1 -1 NA ...
##  $ v_32               : num  1 NA NA NA NA 0 NA -1 0 NA ...
##  $ v_33               : num  1 NA NA NA NA 0 NA -1 1 NA ...
##  $ v_34               : num  1 NA NA NA NA 0 NA -1 0 NA ...
##  $ v_35               : num  1 NA NA NA NA 0 NA -1 -1 NA ...
##  $ v_36               : num  1 NA NA NA NA 0 NA -1 -1 NA ...
##  $ v_122              : num  1 NA NA NA NA 0 NA 1 -1 NA ...
##  $ v_37               : num  1 NA NA NA NA 0 NA -1 1 NA ...
##  $ v_38               : num  NA NA NA NA NA 0 NA 0 1 NA ...
##  $ v_39               : num  1 NA NA NA NA 0 NA 0 NA NA ...
##  $ v_40               : num  1 NA NA NA NA 0 NA -1 -1 NA ...
##  $ v_41               : num  -1 NA NA NA NA 0 NA -1 1 NA ...
##  $ v_123              : num  1 NA NA NA NA 0 NA 1 1 NA ...
##  $ v_124              : num  1 NA NA NA NA 0 NA -1 1 NA ...
##  $ v_42               : num  0 NA NA NA NA 0 NA 1 0 NA ...
##  $ v_43               : num  -1 NA NA NA NA 0 NA -1 1 NA ...
##  $ v_44               : num  0 NA NA NA NA 0 NA -1 1 NA ...
##  $ v_45               : num  -1 NA NA NA NA 0 NA -1 -1 NA ...
##  $ v_46               : num  0 NA NA NA NA 0 NA 1 -1 NA ...
##  $ v_125              : num  0 NA NA NA NA 0 NA -1 -1 NA ...
##  $ v_72               : int  NA 6 NA 4 NA NA 4 NA NA 8 ...
##  $ v_73               : int  NA 7 NA 5 NA NA 3 NA NA 8 ...
##  $ v_74               : int  NA 7 NA 5 NA NA 3 NA NA 8 ...
##  $ v_75               : int  NA 7 NA 6 NA NA 4 NA NA 8 ...
##  $ v_76               : int  NA 7 NA 8 NA NA 6 NA NA 8 ...
##  $ v_77               : int  NA 4 NA 4 NA NA 3 NA NA 5 ...
##  $ v_79               : int  NA 1 NA 3 NA NA 2 NA NA 1 ...
##  $ v_81               : int  NA 1 NA 1 NA NA 4 NA NA 1 ...
##  $ v_83               : int  NA 1 NA 2 NA NA 2 NA NA 1 ...
##  $ v_126              : int  NA NA NA 1 NA NA 3 NA NA 1 ...
##  $ v_127              : int  NA NA NA 1 NA NA 3 NA NA 1 ...
##  $ v_128              : int  NA NA NA 3 NA NA 3 NA NA 2 ...
##  $ v_129              : int  NA NA NA 3 NA NA 3 NA NA 2 ...
##  $ v_130              : int  NA NA NA 2 NA NA 3 NA NA 1 ...
##  $ v_131              : int  NA NA NA 1 NA NA 1 NA NA 1 ...
##  $ v_132              : int  NA NA NA 1 NA NA 1 NA NA 1 ...
##  $ v_133              : int  NA NA NA 2 NA NA 3 NA NA 1 ...
##  $ v_134              : int  NA NA NA 1 NA NA 3 NA NA 1 ...
##  $ v_135              : int  NA NA NA 2 NA NA 3 NA NA 1 ...
##  $ v_136              : int  NA NA NA 1 NA NA 1 NA NA 1 ...
##  $ v_137              : int  NA NA NA 2 NA NA 2 NA NA 1 ...
##  $ v_138              : int  NA NA NA 1 NA NA 3 NA NA 1 ...
##  $ v_139              : int  NA NA NA 1 NA NA 3 NA NA 1 ...
##  $ v_140              : logi  NA NA NA NA NA NA ...
##  $ v_141              : logi  NA NA NA NA NA NA ...
##  $ v_142              : logi  NA NA NA NA NA NA ...
##  $ v_143              : logi  NA NA NA NA NA NA ...
##  $ v_144              : logi  NA NA NA NA NA NA ...
##  $ v_145              : logi  NA NA NA NA NA NA ...
##  $ v_146              : num  NA NA NA -1 NA NA -1 NA NA -1 ...
##  $ v_147              : num  NA NA NA -1 NA NA -1 NA NA -1 ...
##  $ v_148              : num  NA NA NA 1 NA NA -1 NA NA 0 ...
##  $ v_149              : num  NA NA NA -1 NA NA 0 NA NA 0 ...
##  $ v_150              : num  NA NA NA 0 NA NA NA NA NA 0 ...
##  $ v_151              : num  NA NA NA -1 NA NA 1 NA NA 1 ...
##  $ v_152              : num  NA NA NA -1 NA NA 0 NA NA -1 ...
##  $ v_153              : num  NA NA NA -1 NA NA 1 NA NA -1 ...
##   [list output truncated]
```

```
View(data_wide)

data2_wide <- data2[,!names(data2) %in% c("external_lfdn","tester","lastpage",
                                       "quality","p_0001","c_0002","browser",
                                       "referer","device_type",
                                       "quota_assignment","quota_rejected_id",
                                       "page_history","hflip","vflip",
                                       "output_mode","javascript","flash",
                                       "session_id","language","cleaned","ats",
                                       "datetime","date_of_last_access",
                                       "day_of_first_mail","rts6018385",
                                       "rts6018739","rts6018818","rts6019080",
                                       "rts6019089","rts6021451","rts6021455",
                                       "rts6023513","rts6023515","rts6023627",
                                       "rts6023655","rts6023657","rts6023660",
                                       "rts6023667","rts6023676","rts6023679",
                                       "rts6033975")]
names(data2_wide)
```

```
##   [1] "id"                  "dispcode"            "duration"           
##   [4] "condition"           "text_order"          "METI_target"        
##   [7] "s_sex"               "s_age"               "s_school"           
##  [10] "s_german"            "s_psychology"        "s_interest"         
##  [13] "s_contact"           "s_field"             "v_10"               
##  [16] "v_11"                "v_47"                "v_48"               
##  [19] "v_49"                "v_12"                "v_14"               
##  [22] "v_16"                "v_71"                "v_17"               
##  [25] "v_18"                "v_19"                "v_20"               
##  [28] "v_21"                "v_115"               "v_116"              
##  [31] "v_117"               "v_22"                "v_23"               
##  [34] "v_24"                "v_25"                "v_26"               
##  [37] "v_120"               "v_27"                "v_28"               
##  [40] "v_29"                "v_30"                "v_31"               
##  [43] "v_121"               "v_32"                "v_33"               
##  [46] "v_34"                "v_35"                "v_36"               
##  [49] "v_122"               "v_37"                "v_38"               
##  [52] "v_39"                "v_40"                "v_41"               
##  [55] "v_123"               "v_124"               "v_42"               
##  [58] "v_43"                "v_44"                "v_45"               
##  [61] "v_46"                "v_125"               "v_72"               
##  [64] "v_73"                "v_74"                "v_75"               
##  [67] "v_76"                "v_77"                "v_79"               
##  [70] "v_81"                "v_83"                "v_126"              
##  [73] "v_127"               "v_128"               "v_129"              
##  [76] "v_130"               "v_131"               "v_132"              
##  [79] "v_133"               "v_134"               "v_135"              
##  [82] "v_136"               "v_137"               "v_138"              
##  [85] "v_139"               "v_140"               "v_141"              
##  [88] "v_142"               "v_143"               "v_144"              
##  [91] "v_145"               "v_146"               "v_147"              
##  [94] "v_148"               "v_149"               "v_150"              
##  [97] "v_151"               "v_152"               "v_153"              
## [100] "v_154"               "v_155"               "v_156"              
## [103] "v_157"               "v_158"               "v_159"              
## [106] "v_160"               "v_161"               "v_162"              
## [109] "v_163"               "v_164"               "s_CAMA_1_1_1"       
## [112] "s_CAMA_1_1_2"        "s_CAMA_1_1_3"        "s_CAMA_1_1_4"       
## [115] "s_CAMA_1_1_5"        "s_CAMA_1_1_6"        "s_CAMA_1_1_7"       
## [118] "s_CAMA_1_1_8"        "s_CAMA_1_2_1"        "s_CAMA_1_2_2"       
## [121] "s_CAMA_1_2_3"        "s_CAMA_1_2_4"        "v_401"              
## [124] "v_91"                "v_92"                "v_93"               
## [127] "v_94"                "v_95"                "v_96"               
## [130] "v_98"                "v_100"               "v_102"              
## [133] "v_235"               "v_236"               "v_237"              
## [136] "v_238"               "v_239"               "v_240"              
## [139] "v_241"               "v_242"               "v_243"              
## [142] "v_244"               "v_245"               "v_246"              
## [145] "v_247"               "v_248"               "v_249"              
## [148] "v_250"               "v_251"               "v_252"              
## [151] "v_253"               "v_254"               "v_255"              
## [154] "v_256"               "v_257"               "v_258"              
## [157] "v_259"               "s_METI_1_Res_exp_1"  "s_METI_1_Res_int_1" 
## [160] "s_METI_1_Res_ben_1"  "s_METI_1_Res_ben_2"  "s_METI_1_Res_ben_3" 
## [163] "s_METI_1_Res_int_2"  "s_METI_1_Res_exp_2"  "s_METI_1_Res_exp_3" 
## [166] "s_METI_1_Res_exp_4"  "s_METI_1_Res_exp_5"  "s_METI_1_Res_ben_4" 
## [169] "s_METI_1_Res_int_3"  "s_METI_1_Res_exp_6"  "s_METI_1_Res_int_4" 
## [172] "s_METI_1_Auth_exp_1" "s_METI_1_Auth_int_1" "s_METI_1_Auth_ben_1"
## [175] "s_METI_1_Auth_ben_2" "s_METI_1_Auth_ben_3" "s_METI_1_Auth_int_2"
## [178] "s_METI_1_Auth_exp_2" "s_METI_1_Auth_exp_3" "s_METI_1_Auth_exp_4"
## [181] "s_METI_1_Auth_exp_5" "s_METI_1_Auth_ben_4" "s_METI_1_Auth_int_3"
## [184] "s_METI_1_Auth_exp_6" "s_METI_1_Auth_int_4" "v_103"              
## [187] "v_104"               "v_105"               "v_106"              
## [190] "v_107"               "v_108"               "v_110"              
## [193] "v_112"               "v_114"               "v_274"              
## [196] "v_275"               "v_276"               "v_277"              
## [199] "v_278"               "v_279"               "v_280"              
## [202] "v_281"               "v_282"               "v_283"              
## [205] "v_284"               "v_285"               "v_286"              
## [208] "v_287"               "v_288"               "v_289"              
## [211] "v_290"               "v_291"               "v_292"              
## [214] "v_293"               "v_294"               "v_295"              
## [217] "v_296"               "v_297"               "v_298"              
## [220] "s_CAMA_2_1_1"        "s_CAMA_2_1_2"        "s_CAMA_2_1_3"       
## [223] "s_CAMA_2_1_4"        "s_CAMA_2_1_5"        "s_CAMA_2_1_6"       
## [226] "s_CAMA_2_1_7"        "s_CAMA_2_1_8"        "s_CAMA_2_2_1"       
## [229] "s_CAMA_2_2_2"        "s_CAMA_2_2_3"        "s_CAMA_2_2_4"       
## [232] "v_402"               "s_METI_2_Res_exp_1"  "s_METI_2_Res_int_1" 
## [235] "s_METI_2_Res_ben_1"  "s_METI_2_Res_ben_2"  "s_METI_2_Res_ben_3" 
## [238] "s_METI_2_Res_int_2"  "s_METI_2_Res_exp_2"  "s_METI_2_Res_exp_3" 
## [241] "s_METI_2_Res_exp_4"  "s_METI_2_Res_exp_5"  "s_METI_2_Res_ben_4" 
## [244] "s_METI_2_Res_int_3"  "s_METI_2_Res_exp_6"  "s_METI_2_Res_int_4" 
## [247] "s_METI_2_Auth_exp_1" "s_METI_2_Auth_int_1" "s_METI_2_Auth_ben_1"
## [250] "s_METI_2_Auth_ben_2" "s_METI_2_Auth_ben_3" "s_METI_2_Auth_int_2"
## [253] "s_METI_2_Auth_exp_2" "s_METI_2_Auth_exp_3" "s_METI_2_Auth_exp_4"
## [256] "s_METI_2_Auth_exp_5" "s_METI_2_Auth_ben_4" "s_METI_2_Auth_int_3"
## [259] "s_METI_2_Auth_exp_6" "s_METI_2_Auth_int_4" "s_awareness"        
## [262] "quota"               "date_of_first_mail"  "METI_text"          
## [265] "summary1"            "summary2"            "version"            
## [268] "causality"           "disclaimer"          "CAMA"               
## [271] "dropout"             "accessibility_1"     "accessibility_2"    
## [274] "understanding_1"     "understanding_2"     "empowerment_1"      
## [277] "empowerment_2"       "credibility_1"       "credibility_2"      
## [280] "relevance_1"         "relevance_2"         "curiosity_1"        
## [283] "curiosity_2"         "boredom_1"           "boredom_2"          
## [286] "confusion_1"         "confusion_2"         "frustration_1"      
## [289] "frustration_2"       "s_relationship_1"    "s_relationship_2"   
## [292] "s_relationship_3"    "s_relationship_4"    "s_relationship_5"   
## [295] "s_relationship_6"    "s_relationship_7"    "s_relationship_8"   
## [298] "s_extent_1"          "s_extent_2"          "s_extent_3"         
## [301] "s_extent_4"          "s_extent_5"          "s_extent_6"         
## [304] "s_diff_1_1"          "s_diff_1_2"          "s_diff_1_3"         
## [307] "s_diff_1_4"          "s_diff_1_5"          "s_diff_1_6"         
## [310] "s_diff_2_1"          "s_diff_2_2"          "s_diff_2_3"         
## [313] "s_diff_2_4"          "s_diff_2_5"          "s_diff_2_6"         
## [316] "s_funding_1_1"       "s_funding_1_2"       "s_funding_1_3"      
## [319] "s_funding_1_4"       "s_funding_1_5"       "s_funding_1_6"      
## [322] "s_funding_2_1"       "s_funding_2_2"       "s_funding_2_3"      
## [325] "s_funding_2_4"       "s_funding_2_5"       "s_funding_2_6"      
## [328] "s_coi_1_1"           "s_coi_1_2"           "s_coi_1_3"          
## [331] "s_coi_1_4"           "s_coi_1_5"           "s_coi_1_6"          
## [334] "s_coi_1_7"           "s_coi_2_1"           "s_coi_2_2"          
## [337] "s_coi_2_3"           "s_coi_2_4"           "s_coi_2_5"          
## [340] "s_coi_2_6"           "s_coi_2_7"           "s_causality_1_1"    
## [343] "s_causality_1_2"     "s_causality_1_3"     "s_causality_1_4"    
## [346] "s_causality_1_5"     "s_causality_1_6"     "s_causality_2_1"    
## [349] "s_causality_2_2"     "s_causality_2_3"     "s_causality_2_4"    
## [352] "s_causality_2_5"     "s_causality_2_6"     "s_CAMA_1_3"         
## [355] "s_CAMA_2_3"          "s_CAMA_1_1"          "s_CAMA_1_2"         
## [358] "s_CAMA_1_4"          "s_CAMA_1_5"          "s_CAMA_1_6"         
## [361] "s_CAMA_1_7"          "s_CAMA_1_8"          "s_CAMA_2_1"         
## [364] "s_CAMA_2_2"          "s_CAMA_2_4"          "s_CAMA_3"           
## [367] "s_METI_1_exp_1"      "s_METI_1_int_1"      "s_METI_1_ben_1"     
## [370] "s_METI_1_ben_2"      "s_METI_1_ben_3"      "s_METI_1_int_2"     
## [373] "s_METI_1_exp_2"      "s_METI_1_exp_3"      "s_METI_1_exp_4"     
## [376] "s_METI_1_exp_5"      "s_METI_1_ben_4"      "s_METI_1_int_3"     
## [379] "s_METI_1_exp_6"      "s_METI_1_int_4"      "s_METI_2_exp_1"     
## [382] "s_METI_2_int_1"      "s_METI_2_ben_1"      "s_METI_2_ben_2"     
## [385] "s_METI_2_ben_3"      "s_METI_2_int_2"      "s_METI_2_exp_2"     
## [388] "s_METI_2_exp_3"      "s_METI_2_exp_4"      "s_METI_2_exp_5"     
## [391] "s_METI_2_ben_4"      "s_METI_2_int_3"      "s_METI_2_exp_6"     
## [394] "s_METI_2_int_4"      "s_METI_exp_1"        "s_METI_int_1"       
## [397] "s_METI_ben_1"        "s_METI_ben_2"        "s_METI_ben_3"       
## [400] "s_METI_int_2"        "s_METI_exp_2"        "s_METI_exp_3"       
## [403] "s_METI_exp_4"        "s_METI_exp_5"        "s_METI_ben_4"       
## [406] "s_METI_int_3"        "s_METI_exp_6"        "s_METI_int_4"       
## [409] "duration_minutes"
```

```
str(data2_wide)
```

```
## 'data.frame':    2041 obs. of  409 variables:
##  $ id                 : Factor w/ 6705 levels "1","2","3","4",..: 4692 5452 3121 4700 3926 6230 6074 2675 160 402 ...
##  $ dispcode           : int  31 31 31 31 31 31 31 31 31 31 ...
##  $ duration           : int  779 1043 546 746 938 568 1094 1246 662 1298 ...
##  $ condition          : Factor w/ 6 levels "1","2","3","4",..: 3 4 3 5 6 2 1 6 4 4 ...
##  $ text_order         : Factor w/ 2 levels "Barth","Faerber": 1 2 1 2 1 1 2 1 1 2 ...
##  $ METI_target        : Factor w/ 2 levels "Study Authors",..: 2 1 2 1 2 1 2 1 1 1 ...
##  $ s_sex              : Factor w/ 2 levels "female","male": 2 1 2 1 2 1 1 1 2 2 ...
##  $ s_age              : int  31 31 54 45 43 51 57 26 50 35 ...
##  $ s_school           : Factor w/ 3 levels "Haupt","Real",..: 2 1 1 2 3 2 3 2 1 3 ...
##  $ s_german           : int  1 1 1 1 1 1 1 1 1 1 ...
##  $ s_psychology       : int  2 2 2 2 2 2 2 2 2 2 ...
##  $ s_interest         : int  5 5 5 4 7 7 8 7 5 5 ...
##  $ s_contact          : int  1 1 1 2 2 5 3 1 5 1 ...
##  $ s_field            : chr  NA NA NA NA ...
##  $ v_10               : int  4 NA 3 NA 5 NA NA 5 7 NA ...
##  $ v_11               : int  6 NA 2 NA 7 6 NA 7 6 NA ...
##  $ v_47               : int  3 NA 3 NA 7 6 NA 6 5 NA ...
##  $ v_48               : int  6 NA 3 NA 7 NA NA 7 5 NA ...
##  $ v_49               : int  6 NA 5 NA 7 NA NA 7 7 NA ...
##  $ v_12               : int  2 NA 3 NA 4 2 NA 3 4 NA ...
##  $ v_14               : int  1 NA 2 NA 1 4 NA 1 3 NA ...
##  $ v_16               : int  3 NA 4 NA 1 2 NA 1 3 NA ...
##  $ v_71               : int  3 NA 2 NA 1 3 NA 1 4 NA ...
##  $ v_17               : int  1 NA 3 NA 1 2 NA 1 3 NA ...
##  $ v_18               : int  1 NA 3 NA 1 1 NA 1 1 NA ...
##  $ v_19               : int  2 NA 3 NA 1 3 NA 3 3 NA ...
##  $ v_20               : int  2 NA 3 NA 1 2 NA 2 1 NA ...
##  $ v_21               : int  1 NA 3 NA 1 1 NA 1 1 NA ...
##  $ v_115              : int  1 NA 3 NA 1 NA NA 1 1 NA ...
##  $ v_116              : int  1 NA 3 NA 1 1 NA 1 1 NA ...
##  $ v_117              : int  1 NA 3 NA 1 1 NA 1 1 NA ...
##  $ v_22               : int  3 NA 3 NA 1 3 NA 2 1 NA ...
##  $ v_23               : int  1 NA 3 NA 1 1 NA 1 1 NA ...
##  $ v_24               : int  1 NA 3 NA 3 2 NA 1 1 NA ...
##  $ v_25               : int  1 NA 3 NA 1 1 NA 1 1 NA ...
##  $ v_26               : int  1 NA 3 NA 1 3 NA 3 1 NA ...
##  $ v_120              : int  1 NA 3 NA 1 2 NA 1 1 NA ...
##  $ v_27               : num  1 NA 0 NA -1 -1 NA 1 -1 NA ...
##  $ v_28               : num  1 NA 0 NA -1 1 NA -1 0 NA ...
##  $ v_29               : num  -1 NA 0 NA 1 -1 NA 1 1 NA ...
##  $ v_30               : num  1 NA 0 NA -1 1 NA -1 -1 NA ...
##  $ v_31               : num  1 NA 0 NA 1 0 NA 1 1 NA ...
##  $ v_121              : num  1 NA 0 NA 1 -1 NA -1 1 NA ...
##  $ v_32               : num  1 NA 0 NA -1 0 NA 0 -1 NA ...
##  $ v_33               : num  1 NA 0 NA -1 1 NA -1 -1 NA ...
##  $ v_34               : num  1 NA 0 NA -1 0 NA 0 -1 NA ...
##  $ v_35               : num  1 NA 0 NA -1 -1 NA -1 -1 NA ...
##  $ v_36               : num  1 NA 0 NA -1 -1 NA 0 -1 NA ...
##  $ v_122              : num  1 NA 0 NA 1 -1 NA 0 1 NA ...
##  $ v_37               : num  1 NA 0 NA -1 1 NA 1 -1 NA ...
##  $ v_38               : num  NA NA 0 NA 0 1 NA 0 0 NA ...
##  $ v_39               : num  1 NA 0 NA 0 NA NA -1 0 NA ...
##  $ v_40               : num  1 NA 0 NA -1 -1 NA -1 0 NA ...
##  $ v_41               : num  -1 NA 0 NA -1 1 NA -1 1 NA ...
##  $ v_123              : num  1 NA 0 NA 1 1 NA -1 0 NA ...
##  $ v_124              : num  1 NA 0 NA -1 1 NA 1 -1 NA ...
##  $ v_42               : num  0 NA 0 NA 1 0 NA 0 -1 NA ...
##  $ v_43               : num  -1 NA 0 NA -1 1 NA 0 0 NA ...
##  $ v_44               : num  0 NA 0 NA -1 1 NA 0 -1 NA ...
##  $ v_45               : num  -1 NA 0 NA -1 -1 NA -1 -1 NA ...
##  $ v_46               : num  0 NA 0 NA 1 -1 NA -1 0 NA ...
##  $ v_125              : num  0 NA 0 NA -1 -1 NA -1 0 NA ...
##  $ v_72               : int  NA 4 NA 4 NA NA 8 NA NA 3 ...
##  $ v_73               : int  NA 5 NA 3 NA NA 8 NA NA 5 ...
##  $ v_74               : int  NA 5 NA 3 NA NA 8 NA NA 3 ...
##  $ v_75               : int  NA 6 NA 4 NA NA 8 NA NA 8 ...
##  $ v_76               : int  NA 8 NA 6 NA NA 8 NA NA 4 ...
##  $ v_77               : int  NA 4 NA 3 NA NA 5 NA NA 2 ...
##  $ v_79               : int  NA 3 NA 2 NA NA 1 NA NA 2 ...
##  $ v_81               : int  NA 1 NA 4 NA NA 1 NA NA 2 ...
##  $ v_83               : int  NA 2 NA 2 NA NA 1 NA NA 1 ...
##  $ v_126              : int  NA 1 NA 3 NA NA 1 NA NA 1 ...
##  $ v_127              : int  NA 1 NA 3 NA NA 1 NA NA 2 ...
##  $ v_128              : int  NA 3 NA 3 NA NA 2 NA NA 2 ...
##  $ v_129              : int  NA 3 NA 3 NA NA 2 NA NA 2 ...
##  $ v_130              : int  NA 2 NA 3 NA NA 1 NA NA 1 ...
##  $ v_131              : int  NA 1 NA 1 NA NA 1 NA NA 1 ...
##  $ v_132              : int  NA 1 NA 1 NA NA 1 NA NA 2 ...
##  $ v_133              : int  NA 2 NA 3 NA NA 1 NA NA 2 ...
##  $ v_134              : int  NA 1 NA 3 NA NA 1 NA NA 2 ...
##  $ v_135              : int  NA 2 NA 3 NA NA 1 NA NA 2 ...
##  $ v_136              : int  NA 1 NA 1 NA NA 1 NA NA 2 ...
##  $ v_137              : int  NA 2 NA 2 NA NA 1 NA NA 2 ...
##  $ v_138              : int  NA 1 NA 3 NA NA 1 NA NA 2 ...
##  $ v_139              : int  NA 1 NA 3 NA NA 1 NA NA 1 ...
##  $ v_140              : logi  NA NA NA NA NA NA ...
##  $ v_141              : logi  NA NA NA NA NA NA ...
##  $ v_142              : logi  NA NA NA NA NA NA ...
##  $ v_143              : logi  NA NA NA NA NA NA ...
##  $ v_144              : logi  NA NA NA NA NA NA ...
##  $ v_145              : logi  NA NA NA NA NA NA ...
##  $ v_146              : num  NA -1 NA -1 NA NA -1 NA NA 1 ...
##  $ v_147              : num  NA -1 NA -1 NA NA -1 NA NA 1 ...
##  $ v_148              : num  NA 1 NA -1 NA NA 0 NA NA 1 ...
##  $ v_149              : num  NA -1 NA 0 NA NA 0 NA NA 1 ...
##  $ v_150              : num  NA 0 NA NA NA NA 0 NA NA 1 ...
##  $ v_151              : num  NA -1 NA 1 NA NA 1 NA NA 1 ...
##  $ v_152              : num  NA -1 NA 0 NA NA -1 NA NA 1 ...
##  $ v_153              : num  NA -1 NA 1 NA NA -1 NA NA 1 ...
##   [list output truncated]
```

```
View(data2_wide)

#Wide Dataset including only complete cases
```

## METI Scale Generation

```
psych::alpha(data2_wide[,c("s_METI_exp_1","s_METI_exp_2","s_METI_exp_3", "s_METI_exp_4", "s_METI_exp_5","s_METI_exp_6")])
```

```
## 
## Reliability analysis   
## Call: psych::alpha(x = data2_wide[, c("s_METI_exp_1", "s_METI_exp_2", 
##     "s_METI_exp_3", "s_METI_exp_4", "s_METI_exp_5", "s_METI_exp_6")])
## 
##   raw_alpha std.alpha G6(smc) average_r S/N    ase mean  sd median_r
##       0.94      0.94    0.93      0.72  16 0.0021  5.5 1.2     0.73
## 
##     95% confidence boundaries 
##          lower alpha upper
## Feldt     0.94  0.94  0.94
## Duhachek  0.94  0.94  0.94
## 
##  Reliability if an item is dropped:
##              raw_alpha std.alpha G6(smc) average_r S/N alpha se   var.r med.r
## s_METI_exp_1      0.93      0.93    0.92      0.73  13   0.0024 0.00042  0.73
## s_METI_exp_2      0.93      0.93    0.91      0.72  13   0.0025 0.00057  0.72
## s_METI_exp_3      0.93      0.93    0.92      0.73  14   0.0024 0.00024  0.73
## s_METI_exp_4      0.93      0.93    0.91      0.72  13   0.0026 0.00062  0.73
## s_METI_exp_5      0.93      0.93    0.91      0.72  13   0.0026 0.00076  0.72
## s_METI_exp_6      0.93      0.93    0.91      0.72  13   0.0025 0.00082  0.73
## 
##  Item statistics 
##                 n raw.r std.r r.cor r.drop mean  sd
## s_METI_exp_1 2030  0.87  0.87  0.83   0.80  5.5 1.4
## s_METI_exp_2 2034  0.88  0.88  0.85   0.82  5.5 1.4
## s_METI_exp_3 2034  0.86  0.86  0.82   0.79  5.3 1.4
## s_METI_exp_4 2035  0.89  0.89  0.86   0.83  5.4 1.4
## s_METI_exp_5 2029  0.89  0.89  0.86   0.83  5.5 1.4
## s_METI_exp_6 2034  0.88  0.88  0.86   0.83  5.5 1.4
## 
## Non missing response frequency for each item
##                 1    2    3    4    5    6    7 miss
## s_METI_exp_1 0.02 0.02 0.03 0.18 0.18 0.28 0.29 0.01
## s_METI_exp_2 0.02 0.02 0.04 0.18 0.18 0.29 0.29 0.00
## s_METI_exp_3 0.01 0.03 0.05 0.21 0.20 0.26 0.25 0.00
## s_METI_exp_4 0.01 0.02 0.04 0.19 0.18 0.28 0.28 0.00
## s_METI_exp_5 0.01 0.02 0.04 0.18 0.18 0.29 0.27 0.01
## s_METI_exp_6 0.01 0.02 0.04 0.18 0.18 0.29 0.29 0.00
```

```
psych::alpha(data2_wide[,c("s_METI_int_1","s_METI_int_2","s_METI_int_3", "s_METI_int_4")])
```

```
## 
## Reliability analysis   
## Call: psych::alpha(x = data2_wide[, c("s_METI_int_1", "s_METI_int_2", 
##     "s_METI_int_3", "s_METI_int_4")])
## 
##   raw_alpha std.alpha G6(smc) average_r S/N    ase mean  sd median_r
##       0.91      0.91    0.88      0.71 9.6 0.0034  5.4 1.2     0.71
## 
##     95% confidence boundaries 
##          lower alpha upper
## Feldt      0.9  0.91  0.91
## Duhachek   0.9  0.91  0.91
## 
##  Reliability if an item is dropped:
##              raw_alpha std.alpha G6(smc) average_r S/N alpha se   var.r med.r
## s_METI_int_1      0.88      0.89    0.84      0.72 7.7   0.0044 4.5e-04  0.71
## s_METI_int_2      0.88      0.88    0.83      0.70 7.1   0.0047 1.8e-03  0.71
## s_METI_int_3      0.88      0.88    0.83      0.71 7.4   0.0046 3.7e-05  0.71
## s_METI_int_4      0.87      0.87    0.82      0.69 6.8   0.0049 9.9e-04  0.71
## 
##  Item statistics 
##                 n raw.r std.r r.cor r.drop mean  sd
## s_METI_int_1 2033  0.87  0.87  0.81   0.77  5.3 1.4
## s_METI_int_2 2028  0.89  0.89  0.83   0.79  5.4 1.4
## s_METI_int_3 2029  0.88  0.88  0.83   0.78  5.5 1.4
## s_METI_int_4 2024  0.89  0.89  0.85   0.81  5.4 1.4
## 
## Non missing response frequency for each item
##                 1    2    3    4    5    6    7 miss
## s_METI_int_1 0.01 0.02 0.03 0.23 0.20 0.27 0.24 0.00
## s_METI_int_2 0.02 0.02 0.04 0.21 0.18 0.27 0.26 0.01
## s_METI_int_3 0.01 0.02 0.04 0.19 0.17 0.29 0.28 0.01
## s_METI_int_4 0.01 0.02 0.04 0.19 0.19 0.28 0.27 0.01
```

```
psych::alpha(data2_wide[,c("s_METI_ben_1","s_METI_ben_2","s_METI_ben_3", "s_METI_ben_4")])
```

```
## 
## Reliability analysis   
## Call: psych::alpha(x = data2_wide[, c("s_METI_ben_1", "s_METI_ben_2", 
##     "s_METI_ben_3", "s_METI_ben_4")])
## 
##   raw_alpha std.alpha G6(smc) average_r S/N    ase mean  sd median_r
##       0.91      0.91    0.88      0.71 9.6 0.0034  5.4 1.2     0.71
## 
##     95% confidence boundaries 
##          lower alpha upper
## Feldt      0.9  0.91  0.91
## Duhachek   0.9  0.91  0.91
## 
##  Reliability if an item is dropped:
##              raw_alpha std.alpha G6(smc) average_r S/N alpha se   var.r med.r
## s_METI_ben_1      0.87      0.87    0.82      0.70 7.0   0.0048 1.0e-04  0.70
## s_METI_ben_2      0.88      0.88    0.83      0.71 7.2   0.0047 1.6e-04  0.70
## s_METI_ben_3      0.88      0.88    0.83      0.72 7.6   0.0045 2.7e-05  0.72
## s_METI_ben_4      0.88      0.88    0.83      0.70 7.1   0.0047 1.5e-04  0.70
## 
##  Item statistics 
##                 n raw.r std.r r.cor r.drop mean  sd
## s_METI_ben_1 2027  0.89  0.89  0.84   0.80  5.3 1.4
## s_METI_ben_2 2031  0.88  0.88  0.83   0.79  5.3 1.4
## s_METI_ben_3 2032  0.88  0.88  0.81   0.77  5.5 1.4
## s_METI_ben_4 2023  0.89  0.89  0.83   0.79  5.3 1.4
## 
## Non missing response frequency for each item
##                 1    2    3    4    5    6    7 miss
## s_METI_ben_1 0.01 0.02 0.04 0.23 0.20 0.26 0.24 0.01
## s_METI_ben_2 0.02 0.02 0.04 0.22 0.20 0.26 0.25 0.00
## s_METI_ben_3 0.02 0.02 0.04 0.19 0.19 0.27 0.28 0.00
## s_METI_ben_4 0.02 0.02 0.04 0.23 0.20 0.26 0.24 0.01
```

```
data2_wide$s_METI_exp <- rowMeans(data2_wide[,c("s_METI_exp_1","s_METI_exp_2",                               "s_METI_exp_3","s_METI_exp_4",                               "s_METI_exp_5","s_METI_exp_6")])
data2_wide$s_METI_int <- rowMeans(data2_wide[,c("s_METI_int_1","s_METI_int_2",                               "s_METI_int_3","s_METI_int_4")])
data2_wide$s_METI_ben <- rowMeans(data2_wide[,c("s_METI_ben_1","s_METI_ben_2",
                       "s_METI_ben_3","s_METI_ben_4")])

describe(data2_wide$s_METI_exp)
```

```
##    vars    n mean   sd median trimmed  mad min max range  skew kurtosis   se
## X1    1 1997 5.45 1.22   5.67    5.54 1.48   1   7     6 -0.64     0.03 0.03
```

```
describe(data2_wide$s_METI_int)
```

```
##    vars    n mean   sd median trimmed  mad min max range  skew kurtosis   se
## X1    1 1999  5.4 1.23    5.5    5.48 1.48   1   7     6 -0.56    -0.04 0.03
```

```
describe(data2_wide$s_METI_ben)
```

```
##    vars    n mean   sd median trimmed  mad min max range  skew kurtosis   se
## X1    1 1996 5.35 1.23    5.5    5.42 1.48   1   7     6 -0.55     0.12 0.03
```

```
# METI Scale Reliabilities when targeting Summary Authors
data2_wide_summary_authors <- subset(data2_wide, METI_target == 
                                       "Summary Authors")

psych::alpha(data2_wide_summary_authors[,c("s_METI_exp_1","s_METI_exp_2",
                                           "s_METI_exp_3", "s_METI_exp_4",
                                           "s_METI_exp_5","s_METI_exp_6")])
```

```
## 
## Reliability analysis   
## Call: psych::alpha(x = data2_wide_summary_authors[, c("s_METI_exp_1", 
##     "s_METI_exp_2", "s_METI_exp_3", "s_METI_exp_4", "s_METI_exp_5", 
##     "s_METI_exp_6")])
## 
##   raw_alpha std.alpha G6(smc) average_r S/N    ase mean  sd median_r
##       0.94      0.94    0.93      0.73  16 0.0028  5.5 1.2     0.74
## 
##     95% confidence boundaries 
##          lower alpha upper
## Feldt     0.94  0.94  0.95
## Duhachek  0.94  0.94  0.95
## 
##  Reliability if an item is dropped:
##              raw_alpha std.alpha G6(smc) average_r S/N alpha se   var.r med.r
## s_METI_exp_1      0.93      0.94    0.92      0.74  14   0.0032 0.00074  0.75
## s_METI_exp_2      0.93      0.93    0.92      0.73  13   0.0035 0.00086  0.73
## s_METI_exp_3      0.93      0.94    0.92      0.74  14   0.0032 0.00067  0.75
## s_METI_exp_4      0.93      0.93    0.92      0.72  13   0.0035 0.00100  0.73
## s_METI_exp_5      0.93      0.93    0.91      0.72  13   0.0036 0.00128  0.72
## s_METI_exp_6      0.93      0.93    0.92      0.72  13   0.0035 0.00129  0.73
## 
##  Item statistics 
##                 n raw.r std.r r.cor r.drop mean  sd
## s_METI_exp_1 1009  0.86  0.86  0.82   0.79  5.5 1.4
## s_METI_exp_2 1012  0.88  0.88  0.86   0.83  5.5 1.4
## s_METI_exp_3 1012  0.86  0.86  0.82   0.79  5.3 1.4
## s_METI_exp_4 1010  0.89  0.89  0.87   0.84  5.4 1.4
## s_METI_exp_5 1008  0.90  0.90  0.87   0.85  5.5 1.4
## s_METI_exp_6 1009  0.89  0.89  0.87   0.84  5.5 1.4
## 
## Non missing response frequency for each item
##                 1    2    3    4    5    6    7 miss
## s_METI_exp_1 0.01 0.02 0.04 0.18 0.17 0.26 0.31 0.00
## s_METI_exp_2 0.02 0.01 0.04 0.17 0.18 0.28 0.29 0.00
## s_METI_exp_3 0.01 0.03 0.05 0.21 0.19 0.25 0.25 0.00
## s_METI_exp_4 0.01 0.02 0.04 0.19 0.18 0.27 0.28 0.00
## s_METI_exp_5 0.01 0.02 0.04 0.19 0.18 0.28 0.27 0.01
## s_METI_exp_6 0.01 0.02 0.04 0.18 0.19 0.27 0.29 0.00
```

```
psych::alpha(data2_wide_summary_authors[,c("s_METI_int_1","s_METI_int_2",
                                           "s_METI_int_3", "s_METI_int_4")])
```

```
## 
## Reliability analysis   
## Call: psych::alpha(x = data2_wide_summary_authors[, c("s_METI_int_1", 
##     "s_METI_int_2", "s_METI_int_3", "s_METI_int_4")])
## 
##   raw_alpha std.alpha G6(smc) average_r S/N    ase mean  sd median_r
##       0.91      0.91    0.89      0.72  10 0.0045  5.4 1.3     0.73
## 
##     95% confidence boundaries 
##          lower alpha upper
## Feldt      0.9  0.91  0.92
## Duhachek   0.9  0.91  0.92
## 
##  Reliability if an item is dropped:
##              raw_alpha std.alpha G6(smc) average_r S/N alpha se   var.r med.r
## s_METI_int_1      0.89      0.89    0.84      0.73 8.1   0.0060 7.0e-05  0.73
## s_METI_int_2      0.88      0.88    0.84      0.72 7.6   0.0063 1.0e-03  0.73
## s_METI_int_3      0.89      0.89    0.84      0.73 8.0   0.0060 2.1e-05  0.73
## s_METI_int_4      0.88      0.88    0.83      0.71 7.4   0.0064 7.4e-04  0.73
## 
##  Item statistics 
##                 n raw.r std.r r.cor r.drop mean  sd
## s_METI_int_1 1012  0.88  0.88  0.83   0.79  5.4 1.4
## s_METI_int_2 1008  0.89  0.89  0.84   0.81  5.4 1.4
## s_METI_int_3 1009  0.89  0.88  0.83   0.79  5.5 1.4
## s_METI_int_4 1005  0.90  0.90  0.85   0.81  5.5 1.4
## 
## Non missing response frequency for each item
##                 1    2    3    4    5    6    7 miss
## s_METI_int_1 0.01 0.02 0.03 0.23 0.18 0.26 0.26 0.00
## s_METI_int_2 0.02 0.02 0.05 0.21 0.17 0.24 0.29 0.01
## s_METI_int_3 0.01 0.02 0.04 0.19 0.16 0.28 0.30 0.00
## s_METI_int_4 0.01 0.02 0.04 0.18 0.19 0.26 0.29 0.01
```

```
psych::alpha(data2_wide_summary_authors[,c("s_METI_ben_1","s_METI_ben_2",
                                           "s_METI_ben_3", "s_METI_ben_4")])
```

```
## 
## Reliability analysis   
## Call: psych::alpha(x = data2_wide_summary_authors[, c("s_METI_ben_1", 
##     "s_METI_ben_2", "s_METI_ben_3", "s_METI_ben_4")])
## 
##   raw_alpha std.alpha G6(smc) average_r S/N    ase mean  sd median_r
##       0.91      0.91    0.89      0.72  10 0.0045  5.4 1.2     0.73
## 
##     95% confidence boundaries 
##          lower alpha upper
## Feldt      0.9  0.91  0.92
## Duhachek   0.9  0.91  0.92
## 
##  Reliability if an item is dropped:
##              raw_alpha std.alpha G6(smc) average_r S/N alpha se   var.r med.r
## s_METI_ben_1      0.88      0.88    0.84      0.72 7.7   0.0063 2.3e-04  0.73
## s_METI_ben_2      0.88      0.88    0.84      0.72 7.6   0.0064 2.2e-04  0.72
## s_METI_ben_3      0.89      0.89    0.84      0.73 8.1   0.0060 2.3e-05  0.73
## s_METI_ben_4      0.89      0.89    0.84      0.73 8.0   0.0060 7.7e-05  0.73
## 
##  Item statistics 
##                 n raw.r std.r r.cor r.drop mean  sd
## s_METI_ben_1 1008  0.89  0.89  0.85   0.81  5.4 1.4
## s_METI_ben_2 1010  0.90  0.90  0.85   0.81  5.3 1.4
## s_METI_ben_3 1011  0.88  0.88  0.83   0.79  5.5 1.4
## s_METI_ben_4 1005  0.89  0.89  0.83   0.79  5.3 1.4
## 
## Non missing response frequency for each item
##                 1    2    3    4    5    6    7 miss
## s_METI_ben_1 0.01 0.01 0.05 0.22 0.20 0.25 0.25 0.01
## s_METI_ben_2 0.01 0.02 0.05 0.21 0.19 0.26 0.26 0.00
## s_METI_ben_3 0.02 0.01 0.04 0.18 0.19 0.28 0.27 0.00
## s_METI_ben_4 0.02 0.02 0.04 0.23 0.19 0.25 0.25 0.01
```

```
# METI Scale Reliabilities when targeting Study Authors
data2_wide_study_authors <- subset(data2_wide, METI_target == "Study Authors")

psych::alpha(data2_wide_study_authors[,c("s_METI_exp_1","s_METI_exp_2",
                                           "s_METI_exp_3", "s_METI_exp_4",
                                           "s_METI_exp_5","s_METI_exp_6")])
```

```
## 
## Reliability analysis   
## Call: psych::alpha(x = data2_wide_study_authors[, c("s_METI_exp_1", 
##     "s_METI_exp_2", "s_METI_exp_3", "s_METI_exp_4", "s_METI_exp_5", 
##     "s_METI_exp_6")])
## 
##   raw_alpha std.alpha G6(smc) average_r S/N   ase mean  sd median_r
##       0.94      0.94    0.93      0.72  15 0.003  5.5 1.2     0.72
## 
##     95% confidence boundaries 
##          lower alpha upper
## Feldt     0.93  0.94  0.94
## Duhachek  0.93  0.94  0.94
## 
##  Reliability if an item is dropped:
##              raw_alpha std.alpha G6(smc) average_r S/N alpha se   var.r med.r
## s_METI_exp_1      0.93      0.93    0.91      0.72  13   0.0036 0.00032  0.72
## s_METI_exp_2      0.93      0.93    0.91      0.71  13   0.0037 0.00044  0.72
## s_METI_exp_3      0.93      0.93    0.91      0.72  13   0.0035 0.00035  0.72
## s_METI_exp_4      0.92      0.93    0.91      0.71  12   0.0037 0.00076  0.71
## s_METI_exp_5      0.93      0.93    0.91      0.71  12   0.0037 0.00074  0.71
## s_METI_exp_6      0.93      0.93    0.91      0.72  13   0.0037 0.00084  0.72
## 
##  Item statistics 
##                 n raw.r std.r r.cor r.drop mean  sd
## s_METI_exp_1 1021  0.87  0.87  0.84   0.81  5.5 1.4
## s_METI_exp_2 1022  0.88  0.88  0.85   0.82  5.5 1.4
## s_METI_exp_3 1022  0.86  0.86  0.82   0.79  5.3 1.4
## s_METI_exp_4 1025  0.88  0.88  0.85   0.83  5.4 1.4
## s_METI_exp_5 1021  0.88  0.88  0.85   0.82  5.5 1.4
## s_METI_exp_6 1025  0.87  0.87  0.84   0.82  5.5 1.3
## 
## Non missing response frequency for each item
##                 1    2    3    4    5    6    7 miss
## s_METI_exp_1 0.02 0.02 0.03 0.18 0.19 0.29 0.28 0.01
## s_METI_exp_2 0.02 0.02 0.03 0.18 0.17 0.29 0.29 0.00
## s_METI_exp_3 0.01 0.02 0.05 0.21 0.20 0.26 0.24 0.00
## s_METI_exp_4 0.01 0.02 0.03 0.19 0.19 0.29 0.27 0.00
## s_METI_exp_5 0.01 0.03 0.03 0.18 0.17 0.30 0.27 0.01
## s_METI_exp_6 0.01 0.02 0.03 0.18 0.18 0.30 0.29 0.00
```

```
psych::alpha(data2_wide_study_authors[,c("s_METI_int_1","s_METI_int_2",
                                           "s_METI_int_3", "s_METI_int_4")])
```

```
## 
## Reliability analysis   
## Call: psych::alpha(x = data2_wide_study_authors[, c("s_METI_int_1", 
##     "s_METI_int_2", "s_METI_int_3", "s_METI_int_4")])
## 
##   raw_alpha std.alpha G6(smc) average_r S/N    ase mean  sd median_r
##        0.9       0.9    0.87      0.69 8.9 0.0052  5.4 1.2     0.69
## 
##     95% confidence boundaries 
##          lower alpha upper
## Feldt     0.89   0.9  0.91
## Duhachek  0.89   0.9  0.91
## 
##  Reliability if an item is dropped:
##              raw_alpha std.alpha G6(smc) average_r S/N alpha se  var.r med.r
## s_METI_int_1      0.88      0.88    0.83      0.71 7.3   0.0066 0.0012  0.69
## s_METI_int_2      0.87      0.87    0.82      0.69 6.6   0.0071 0.0033  0.68
## s_METI_int_3      0.87      0.87    0.82      0.69 6.7   0.0070 0.0002  0.69
## s_METI_int_4      0.86      0.86    0.81      0.68 6.2   0.0075 0.0013  0.69
## 
##  Item statistics 
##                 n raw.r std.r r.cor r.drop mean  sd
## s_METI_int_1 1021  0.86  0.86  0.79   0.75  5.3 1.3
## s_METI_int_2 1020  0.88  0.88  0.82   0.78  5.3 1.4
## s_METI_int_3 1020  0.88  0.88  0.82   0.77  5.4 1.4
## s_METI_int_4 1019  0.89  0.89  0.84   0.80  5.4 1.4
## 
## Non missing response frequency for each item
##                 1    2    3    4    5    6    7 miss
## s_METI_int_1 0.01 0.02 0.03 0.22 0.22 0.28 0.22 0.01
## s_METI_int_2 0.02 0.02 0.03 0.21 0.20 0.29 0.23 0.01
## s_METI_int_3 0.01 0.02 0.04 0.19 0.18 0.30 0.26 0.01
## s_METI_int_4 0.02 0.01 0.04 0.20 0.19 0.30 0.25 0.01
```

```
psych::alpha(data2_wide_study_authors[,c("s_METI_ben_1","s_METI_ben_2",
                                           "s_METI_ben_3", "s_METI_ben_4")])
```

```
## 
## Reliability analysis   
## Call: psych::alpha(x = data2_wide_study_authors[, c("s_METI_ben_1", 
##     "s_METI_ben_2", "s_METI_ben_3", "s_METI_ben_4")])
## 
##   raw_alpha std.alpha G6(smc) average_r S/N    ase mean  sd median_r
##        0.9       0.9    0.87      0.69 8.9 0.0052  5.3 1.2     0.69
## 
##     95% confidence boundaries 
##          lower alpha upper
## Feldt     0.89   0.9  0.91
## Duhachek  0.89   0.9  0.91
## 
##  Reliability if an item is dropped:
##              raw_alpha std.alpha G6(smc) average_r S/N alpha se   var.r med.r
## s_METI_ben_1      0.86      0.86    0.81      0.68 6.4   0.0073 5.6e-04  0.69
## s_METI_ben_2      0.87      0.87    0.82      0.70 6.9   0.0068 1.3e-04  0.69
## s_METI_ben_3      0.88      0.88    0.82      0.70 7.0   0.0067 8.3e-05  0.70
## s_METI_ben_4      0.86      0.86    0.81      0.68 6.4   0.0073 5.7e-04  0.69
## 
##  Item statistics 
##                 n raw.r std.r r.cor r.drop mean  sd
## s_METI_ben_1 1019  0.88  0.88  0.83   0.79  5.3 1.4
## s_METI_ben_2 1021  0.87  0.87  0.80   0.76  5.3 1.4
## s_METI_ben_3 1021  0.87  0.87  0.80   0.76  5.5 1.4
## s_METI_ben_4 1018  0.88  0.88  0.83   0.79  5.3 1.4
## 
## Non missing response frequency for each item
##                 1    2    3    4    5    6    7 miss
## s_METI_ben_1 0.01 0.02 0.04 0.24 0.19 0.26 0.23 0.01
## s_METI_ben_2 0.02 0.01 0.03 0.23 0.20 0.27 0.24 0.01
## s_METI_ben_3 0.02 0.02 0.04 0.19 0.19 0.27 0.28 0.01
## s_METI_ben_4 0.02 0.02 0.04 0.22 0.21 0.27 0.23 0.01
```

### CFA METI

```
meti_mod1 <- "trust =~ s_METI_exp_1 + s_METI_exp_2 + s_METI_exp_3 + s_METI_exp_4
+ s_METI_exp_5 + s_METI_exp_6 + s_METI_int_1 + s_METI_int_2 + s_METI_int_3 +
s_METI_int_4 + s_METI_ben_1 + s_METI_ben_2 + s_METI_ben_3 + s_METI_ben_4"
meti_fit1 <- cfa(meti_mod1, data = data2_wide)
fit_1 <- fitmeasures(meti_fit1)[c("chisq","df","tli","cfi","rmsea","srmr")]

meti_mod2 <- "exp =~ s_METI_exp_1 + s_METI_exp_2 + s_METI_exp_3 + s_METI_exp_4
+ s_METI_exp_5 + s_METI_exp_6
intben =~ s_METI_int_1 + s_METI_int_2 + s_METI_int_3 +
s_METI_int_4 + s_METI_ben_1 + s_METI_ben_2 + s_METI_ben_3 + s_METI_ben_4"
meti_fit2 <- cfa(meti_mod2, data = data2_wide)
fit_2 <- fitmeasures(meti_fit2)[c("chisq","df","tli","cfi","rmsea","srmr")]

meti_mod3 <- "exp =~ s_METI_exp_1 + s_METI_exp_2 + s_METI_exp_3 + s_METI_exp_4
+ s_METI_exp_5 + s_METI_exp_6
int =~ s_METI_int_1 + s_METI_int_2 + s_METI_int_3 + s_METI_int_4
ben =~ s_METI_ben_1 + s_METI_ben_2 + s_METI_ben_3 + s_METI_ben_4"
meti_fit3 <- cfa(meti_mod3, data = data2_wide)
fit_3 <- fitmeasures(meti_fit3)[c("chisq","df","tli","cfi","rmsea","srmr")]

anova(meti_fit1,meti_fit2,meti_fit3)
```

```
## 
## Chi-Squared Difference Test
## 
##           Df   AIC   BIC  Chisq Chisq diff   RMSEA Df diff Pr(>Chisq)    
## meti_fit3 74 69189 69361 341.85                                          
## meti_fit2 76 69208 69369 364.96      23.11 0.07382       2  9.588e-06 ***
## meti_fit1 77 69437 69593 595.90     230.94 0.34454       1  < 2.2e-16 ***
## ---
## Signif. codes:  0 '***' 0.001 '**' 0.01 '*' 0.05 '.' 0.1 ' ' 1
```

## Create Overall Scores for Knowledge Items

### Relationship-Item

```
data2_wide$s_relationship <- rowSums(data2_wide[,c("s_relationship_1",
                                                   "s_relationship_2",
                                                   "s_relationship_3",
                                                   "s_relationship_4",
                                                   "s_relationship_5",
                                                   "s_relationship_6",
                                                   "s_relationship_7",
                                                   "s_relationship_8")])
describe(data2_wide$s_relationship)
```

```
##    vars    n mean   sd median trimmed  mad min max range skew kurtosis   se
## X1    1 1979 0.23 3.22      0    0.02 2.97  -8   8    16 0.45    -0.37 0.07
```

```
table(data2_wide$s_relationship)
```

```
## 
##  -8  -7  -6  -5  -4  -3  -2  -1   0   1   2   3   4   5   6   7   8 
##   4   1  20  20 247 110 251 163 425 112 192  67 141  30 135  17  44
```

### Extent of Evaluation-Item

```
data2_wide$s_extent <- rowSums(data2_wide[,c("s_extent_1","s_extent_2",                                   "s_extent_3","s_extent_4",                                   "s_extent_5","s_extent_6")])
describe(data2_wide$s_extent)
```

```
##    vars    n mean   sd median trimmed  mad min max range skew kurtosis   se
## X1    1 1999 0.52 2.28      0    0.41 2.97  -6   6    12 0.35    -0.28 0.05
```

```
table(data2_wide$s_extent)
```

```
## 
##  -6  -5  -4  -3  -2  -1   0   1   2   3   4   5   6 
##   4   2  45  52 345 180 533 179 281 118 168  35  57
```

### Differentiation-Item

```
data2_wide$s_diff_1 <- rowSums(data2_wide[,c("s_diff_1_1","s_diff_1_2",                                       "s_diff_1_3","s_diff_1_4",
                      "s_diff_1_5","s_diff_1_6")])
describe(data2_wide$s_diff_1)
```

```
##    vars   n mean   sd median trimmed  mad min max range skew kurtosis   se
## X1    1 997 0.07 1.83      0    0.05 1.48  -6   6    12 0.14     0.68 0.06
```

```
table(data2_wide$s_diff_1)
```

```
## 
##  -6  -4  -3  -2  -1   0   1   2   3   4   5   6 
##   3  33  22 145 103 360 117 146  22  33   7   6
```

```
data2_wide$s_diff_2 <- rowSums(data2_wide[,c("s_diff_2_1","s_diff_2_2",                                       "s_diff_2_3","s_diff_2_4",
                      "s_diff_2_5","s_diff_2_6")])
describe(data2_wide$s_diff_2)
```

```
##    vars   n mean   sd median trimmed  mad min max range skew kurtosis   se
## X1    1 981 0.52 2.07      0    0.46 2.97  -6   6    12 0.15     0.23 0.07
```

```
table(data2_wide$s_diff_2)
```

```
## 
##  -6  -5  -4  -3  -2  -1   0   1   2   3   4   5   6 
##   2   3  29  15 123  61 343  82 191  31  74  10  17
```

### Funding-Item

```
data2_wide$s_funding_1 <- rowSums(data2_wide[,c("s_funding_1_1","s_funding_1_2",
                      "s_funding_1_3","s_funding_1_4",                             "s_funding_1_5","s_funding_1_6")])
describe(data2_wide$s_funding_1)
```

```
##    vars    n mean   sd median trimmed  mad min max range skew kurtosis   se
## X1    1 1994 0.87 3.05      0    0.84 2.97  -6   6    12 0.26    -0.74 0.07
```

```
table(data2_wide$s_funding_1)
```

```
## 
##  -6  -5  -4  -3  -2  -1   0   1   2   3   4   5   6 
##  13  10 152  54 215 175 511 140 181  84 115  48 296
```

```
data2_wide$s_funding_2 <- rowSums(data2_wide[,c("s_funding_2_1","s_funding_2_2",
                      "s_funding_2_3","s_funding_2_4",
                      "s_funding_2_5","s_funding_2_6")])
describe(data2_wide$s_funding_2)
```

```
##    vars    n mean   sd median trimmed  mad min max range  skew kurtosis   se
## X1    1 2010 1.67 3.22      1    1.82 4.45  -6   6    12 -0.07    -1.03 0.07
```

```
table(data2_wide$s_funding_2)
```

```
## 
##  -6  -5  -4  -3  -2  -1   0   1   2   3   4   5   6 
##  12  12 119  46 157 103 474 118 206  59 177  48 479
```

### COI-Item

```
data2_wide$s_coi_1 <- rowSums(data2_wide[,c("s_coi_1_1","s_coi_1_2",
                      "s_coi_1_3","s_coi_1_4",
                      "s_coi_1_5","s_coi_1_6", "s_coi_1_7")])
describe(data2_wide$s_coi_1)
```

```
##    vars    n mean   sd median trimmed  mad min max range skew kurtosis   se
## X1    1 1955 0.72 3.33      0    0.66 2.97  -7   7    14 0.19     -0.5 0.08
```

```
table(data2_wide$s_coi_1)
```

```
## 
##  -7  -6  -5  -4  -3  -2  -1   0   1   2   3   4   5   6   7 
##   9   8 137  51 133 101 182 467 208  83 187  47 148  22 172
```

```
data2_wide$s_coi_2 <- rowSums(data2_wide[,c("s_coi_2_1","s_coi_2_2",
                      "s_coi_2_3","s_coi_2_4",                                     "s_coi_2_5","s_coi_2_6","s_coi_2_7")])
describe(data2_wide$s_coi_2)
```

```
##    vars    n mean   sd median trimmed  mad min max range  skew kurtosis   se
## X1    1 1993  1.3 3.56      1    1.36 2.97  -7   7    14 -0.01    -0.75 0.08
```

```
table(data2_wide$s_coi_2)
```

```
## 
##  -7  -6  -5  -4  -3  -2  -1   0   1   2   3   4   5   6   7 
##  18  14 114  37 123  72 182 407 201  69 207  33 235  22 259
```

### Causality-Item

```
data2_wide$s_causality_1 <- rowSums(data2_wide[,c("s_causality_1_1","s_causality_1_2",
                      "s_causality_1_3","s_causality_1_4",
                      "s_causality_1_5","s_causality_1_6")])
describe(data2_wide$s_causality_1)
```

```
##    vars    n  mean   sd median trimmed  mad min max range skew kurtosis   se
## X1    1 1989 -0.43 2.45      0   -0.52 2.97  -6   6    12  0.2    -0.56 0.06
```

```
table(data2_wide$s_causality_1)
```

```
## 
##  -6  -5  -4  -3  -2  -1   0   1   2   3   4   5   6 
##   8   7 297 112 295 173 493 116 266  68 121  14  19
```

```
data2_wide$s_causality_2 <- rowSums(data2_wide[,c("s_causality_2_1","s_causality_2_2",
                      "s_causality_2_3","s_causality_2_4",
                      "s_causality_2_5","s_causality_2_6")])
describe(data2_wide$s_causality_2)
```

```
##    vars    n  mean   sd median trimmed  mad min max range skew kurtosis   se
## X1    1 1995 -0.21 2.45      0   -0.29 2.97  -6   6    12 0.21    -0.31 0.05
```

```
table(data2_wide$s_causality_2)
```

```
## 
##  -6  -5  -4  -3  -2  -1   0   1   2   3   4   5   6 
##   8   7 247 100 267 173 541 149 263  59 131   9  41
```

### CAMA-Items

```
data2_wide$s_CAMA_1 <- rowSums(data2_wide[,c("s_CAMA_1_1","s_CAMA_1_2",
                      "s_CAMA_1_3","s_CAMA_1_4",
                      "s_CAMA_1_5","s_CAMA_1_6",
                      "s_CAMA_1_7","s_CAMA_1_8")])
describe(data2_wide$s_CAMA_1)
```

```
##    vars   n mean   sd median trimmed  mad min max range skew kurtosis   se
## X1    1 984  0.7 2.66      0    0.68 2.97  -7   8    15 0.16    -0.02 0.08
```

```
table(data2_wide$s_CAMA_1)
```

```
## 
##  -7  -6  -5  -4  -3  -2  -1   0   1   2   3   4   5   6   7   8 
##   1   5   5  69  38  66  46 318 100 110  61  85  25  41   7   7
```

```
data2_wide$s_CAMA_2 <- rowSums(data2_wide[,c("s_CAMA_2_1","s_CAMA_2_2",
                      "s_CAMA_2_3","s_CAMA_2_4")])
describe(data2_wide$s_CAMA_2)
```

```
##    vars    n mean   sd median trimmed  mad min max range skew kurtosis   se
## X1    1 1015 -0.4 1.62      0   -0.44 1.48  -4   4     8 0.18     0.05 0.05
```

```
table(data2_wide$s_CAMA_2)
```

```
## 
##  -4  -3  -2  -1   0   1   2   3   4 
##  32  36 239  99 397  67 112  14  19
```

```
describe(data2_wide$s_CAMA_3)
```

```
##    vars    n  mean   sd median trimmed  mad min max range skew kurtosis   se
## X1    1 1026 -0.14 0.75      0   -0.17 1.48  -1   1     2 0.23    -1.21 0.02
```

```
table(data2_wide$s_CAMA_3)
```

```
## 
##  -1   0   1 
## 369 428 229
```

```
data2_wide$s_CAMA <- rowSums(data2_wide[,c("s_CAMA_1","s_CAMA_2","s_CAMA_3")])
describe(data2_wide$s_CAMA)
```

```
##    vars   n mean   sd median trimmed  mad min max range skew kurtosis   se
## X1    1 975 0.17 3.73      0    0.17 2.97 -11  13    24 0.09     0.13 0.12
```

```
table(data2_wide$s_CAMA)
```

```
## 
## -11  -9  -8  -7  -6  -5  -4  -3  -2  -1   0   1   2   3   4   5   6   7   8   9 
##   1   4   1  50  26  43  25  49  68  93 230  89  50  67  49  55  23  26   8  12 
##  10  11  12  13 
##   1   3   1   1
```

## Melt Dataframe into Long Format

```
data2_long <- melt.data.table(setDT(data2_wide),measure.vars = 
                                list(c("summary1","summary2"),
                                     c("accessibility_1","accessibility_2"),
                                     c("understanding_1","understanding_2"),
                                     c("empowerment_1","empowerment_2"),
                                     c("credibility_1","credibility_2"),
                                     c("relevance_1","relevance_2"),
                                     c("curiosity_1","curiosity_2"),
                                     c("boredom_1","boredom_2"),
                                     c("frustration_1","frustration_2"),
                                     c("confusion_1","confusion_2"),
                                     c("s_funding_1","s_funding_2"),
                                     c("s_coi_1","s_coi_2"),
c("s_diff_1","s_diff_2"),                                     c("s_causality_1","s_causality_2")),
                              value.name = c("summary","accessibility",
                                             "understanding","empowerment",
                                             "credibility","relevance",
                                             "curiosity",
                                             "boredom","frustration",
                                             "confusion","s_funding",                                             "s_coi","s_diff","s_causality"),
                              variable.name = "Time_point")

View(data2_long)

data2_long <- dplyr::select(data2_long, -c(15:260,263))
View(data2_long)

data2_long$summary <- factor(data2_long$summary, levels = c("Barth","Faerber"))
```

## User Experience Scale Generation

```
psych::alpha(data2_long[,c("accessibility","understanding","empowerment")])
```

```
## 
## Reliability analysis   
## Call: psych::alpha(x = data2_long[, c("accessibility", "understanding", 
##     "empowerment")])
## 
##   raw_alpha std.alpha G6(smc) average_r S/N    ase mean  sd median_r
##       0.83      0.83    0.77      0.62   5 0.0046  5.3 1.5     0.66
## 
##     95% confidence boundaries 
##          lower alpha upper
## Feldt     0.82  0.83  0.84
## Duhachek  0.82  0.83  0.84
## 
##  Reliability if an item is dropped:
##               raw_alpha std.alpha G6(smc) average_r S/N alpha se var.r med.r
## accessibility      0.79      0.79    0.66      0.66 3.8   0.0065    NA  0.66
## understanding      0.72      0.72    0.56      0.56 2.5   0.0089    NA  0.56
## empowerment        0.79      0.79    0.66      0.66 3.8   0.0065    NA  0.66
## 
##  Item statistics 
##                  n raw.r std.r r.cor r.drop mean  sd
## accessibility 4045  0.86  0.85  0.73   0.67  5.5 1.8
## understanding 4038  0.89  0.89  0.82   0.74  5.6 1.7
## empowerment   4039  0.85  0.85  0.73   0.67  4.8 1.8
## 
## Non missing response frequency for each item
##                  1    2    3    4    5    6    7    8 miss
## accessibility 0.03 0.03 0.08 0.15 0.17 0.20 0.16 0.18 0.01
## understanding 0.02 0.03 0.07 0.14 0.19 0.22 0.17 0.15 0.01
## empowerment   0.06 0.06 0.12 0.19 0.22 0.19 0.09 0.08 0.01
```

### CFA User Experience

```
UEmodel <- "outcome =~ c(a)*accessibility + c(a)*understanding + c(a)*empowerment
accessibility ~~ c(b)*empowerment"

UEfit <- sem(UEmodel, data = data2_long, estimator = "MLR", missing = "ML",
             std.lv = T, fixed.x = F, group = "summary")
```

```
## Warning in lav_data_full(data = data, group = group, cluster = cluster, : lavaan WARNING: some cases are empty and will be ignored:
##   718 1417 1965 2985 3986
```

```
## Warning in lav_data_full(data = data, group = group, cluster = cluster, : lavaan WARNING: some cases are empty and will be ignored:
##   1975
```

```
## Warning in lavaanify(model = FLAT, constraints = constraints, varTable = DataOV, : lavaan WARNING: using a single label per parameter in a multiple group
##   setting implies imposing equality constraints across all the groups;
##   If this is not intended, either remove the label(s), or use a vector
##   of labels (one for each group);
##   See the Multiple groups section in the man page of model.syntax.
```

```
summary(UEfit, standardized = T)
```

```
## lavaan 0.6.16 ended normally after 36 iterations
## 
##   Estimator                                         ML
##   Optimization method                           NLMINB
##   Number of model parameters                        20
##   Number of equality constraints                     6
## 
##   Number of observations per group:               Used       Total
##     Barth                                         2036        2041
##     Faerber                                       2040        2041
##   Number of missing patterns per group:                           
##     Barth                                            6            
##     Faerber                                          7            
## 
## Model Test User Model:
##                                               Standard      Scaled
##   Test Statistic                                 3.534       3.167
##   Degrees of freedom                                 4           4
##   P-value (Chi-square)                           0.473       0.530
##   Scaling correction factor                                  1.116
##     Yuan-Bentler correction (Mplus variant)                       
##   Test statistic for each group:
##     Barth                                        1.326       1.188
##     Faerber                                      2.208       1.978
## 
## Parameter Estimates:
## 
##   Standard errors                             Sandwich
##   Information bread                           Observed
##   Observed information based on                Hessian
## 
## 
## Group 1 [Barth]:
## 
## Latent Variables:
##                    Estimate  Std.Err  z-value  P(>|z|)   Std.lv  Std.all
##   outcome =~                                                            
##     accessblty (a)    1.428    0.018   78.985    0.000    1.428    0.782
##     undrstndng (a)    1.428    0.018   78.985    0.000    1.428    0.834
##     empowermnt (a)    1.428    0.018   78.985    0.000    1.428    0.789
## 
## Covariances:
##                    Estimate  Std.Err  z-value  P(>|z|)   Std.lv  Std.all
##  .accessibility ~~                                                      
##    .empowermnt (b)   -0.204    0.039   -5.276    0.000   -0.204   -0.161
## 
## Intercepts:
##                    Estimate  Std.Err  z-value  P(>|z|)   Std.lv  Std.all
##    .accessibility     5.543    0.040  137.942    0.000    5.543    3.034
##    .understanding     5.646    0.037  150.746    0.000    5.646    3.295
##    .empowerment       4.839    0.040  121.599    0.000    4.839    2.674
##     outcome           0.000                               0.000    0.000
## 
## Variances:
##                    Estimate  Std.Err  z-value  P(>|z|)   Std.lv  Std.all
##    .accessibility     1.299    0.072   18.046    0.000    1.299    0.389
##    .understanding     0.896    0.057   15.604    0.000    0.896    0.305
##    .empowerment       1.236    0.071   17.419    0.000    1.236    0.377
##     outcome           1.000                               1.000    1.000
## 
## 
## Group 2 [Faerber]:
## 
## Latent Variables:
##                    Estimate  Std.Err  z-value  P(>|z|)   Std.lv  Std.all
##   outcome =~                                                            
##     accessblty (a)    1.428    0.018   78.985    0.000    1.428    0.787
##     undrstndng (a)    1.428    0.018   78.985    0.000    1.428    0.838
##     empowermnt (a)    1.428    0.018   78.985    0.000    1.428    0.786
## 
## Covariances:
##                    Estimate  Std.Err  z-value  P(>|z|)   Std.lv  Std.all
##  .accessibility ~~                                                      
##    .empowermnt (b)   -0.204    0.039   -5.276    0.000   -0.204   -0.162
## 
## Intercepts:
##                    Estimate  Std.Err  z-value  P(>|z|)   Std.lv  Std.all
##    .accessibility     5.528    0.041  135.085    0.000    5.528    3.045
##    .understanding     5.528    0.038  143.776    0.000    5.528    3.242
##    .empowerment       4.717    0.040  116.604    0.000    4.717    2.597
##     outcome           0.000                               0.000    0.000
## 
## Variances:
##                    Estimate  Std.Err  z-value  P(>|z|)   Std.lv  Std.all
##    .accessibility     1.256    0.074   17.074    0.000    1.256    0.381
##    .understanding     0.867    0.050   17.364    0.000    0.867    0.298
##    .empowerment       1.261    0.068   18.628    0.000    1.261    0.382
##     outcome           1.000                               1.000    1.000
```

```
modificationindices(UEfit)
```

```
##              lhs op           rhs block group level    mi    epc sepc.lv
## 8        outcome ~~       outcome     1     1     1 1.962 -0.073  -1.000
## 20       outcome ~~       outcome     2     2     1 1.962  0.073   1.000
## 31 accessibility ~~ understanding     1     1     1 0.337 -0.034  -0.034
## 32 understanding ~~   empowerment     1     1     1 0.425 -0.038  -0.038
## 33 accessibility ~~ understanding     2     2     1 2.234  0.086   0.086
## 34 understanding ~~   empowerment     2     2     1 0.072 -0.016  -0.016
##    sepc.all sepc.nox
## 8    -1.000   -1.000
## 20    1.000    1.000
## 31   -0.031   -0.031
## 32   -0.036   -0.036
## 33    0.083    0.083
## 34   -0.015   -0.015
```

```
fitmeasures(UEfit)
```

```
##                          npar                          fmin 
##                        14.000                         0.000 
##                         chisq                            df 
##                         3.534                         4.000 
##                        pvalue                  chisq.scaled 
##                         0.473                         3.167 
##                     df.scaled                 pvalue.scaled 
##                         4.000                         0.530 
##          chisq.scaling.factor                baseline.chisq 
##                         1.116                      4732.569 
##                   baseline.df               baseline.pvalue 
##                         6.000                         0.000 
##         baseline.chisq.scaled            baseline.df.scaled 
##                      2413.601                         6.000 
##        baseline.pvalue.scaled baseline.chisq.scaling.factor 
##                         0.000                         1.961 
##                           cfi                           tli 
##                         1.000                         1.000 
##                    cfi.scaled                    tli.scaled 
##                         1.000                         1.001 
##                    cfi.robust                    tli.robust 
##                         1.000                         1.000 
##                          nnfi                           rfi 
##                         1.000                         0.999 
##                           nfi                          pnfi 
##                         0.999                         0.666 
##                           ifi                           rni 
##                         1.000                         1.000 
##                   nnfi.scaled                    rfi.scaled 
##                         1.001                         0.998 
##                    nfi.scaled                   pnfi.scaled 
##                         0.999                         0.666 
##                    ifi.scaled                    rni.scaled 
##                         1.000                         1.000 
##                   nnfi.robust                    rni.robust 
##                         1.000                         1.000 
##                          logl             unrestricted.logl 
##                    -21827.855                    -21826.088 
##                           aic                           bic 
##                     43683.710                     43772.090 
##                        ntotal                          bic2 
##                      4076.000                     43727.604 
##             scaling.factor.h1             scaling.factor.h0 
##                         1.245                         0.898 
##                         rmsea                rmsea.ci.lower 
##                         0.000                         0.000 
##                rmsea.ci.upper                rmsea.ci.level 
##                         0.032                         0.900 
##                  rmsea.pvalue                rmsea.close.h0 
##                         0.999                         0.050 
##         rmsea.notclose.pvalue             rmsea.notclose.h0 
##                         0.000                         0.080 
##                  rmsea.scaled         rmsea.ci.lower.scaled 
##                         0.000                         0.000 
##         rmsea.ci.upper.scaled           rmsea.pvalue.scaled 
##                         0.029                         1.000 
##  rmsea.notclose.pvalue.scaled                  rmsea.robust 
##                         0.000                         0.000 
##         rmsea.ci.lower.robust         rmsea.ci.upper.robust 
##                         0.000                         0.032 
##           rmsea.pvalue.robust  rmsea.notclose.pvalue.robust 
##                         0.999                         0.000 
##                           rmr                    rmr_nomean 
##                         0.062                         0.076 
##                          srmr                  srmr_bentler 
##                         0.020                         0.020 
##           srmr_bentler_nomean                          crmr 
##                         0.024                         0.006 
##                   crmr_nomean                    srmr_mplus 
##                         0.009                         0.027 
##             srmr_mplus_nomean                         cn_05 
##                         0.019                     10943.881 
##                         cn_01                           gfi 
##                     15313.979                         1.000 
##                          agfi                          pgfi 
##                         1.000                         0.222 
##                           mfi                          ecvi 
##                         1.000                         0.008
```

# Descriptive Analyses

## Participants’ Gender

```
table(data2_wide$s_sex)
```

```
## 
## female   male 
##   1028   1013
```

```
prop.table(table(data2_wide$s_sex))
```

```
## 
##    female      male 
## 0.5036747 0.4963253
```

## Participants’ Age

```
describe(data2_wide$s_age)
```

```
##    vars    n  mean    sd median trimmed   mad min max range skew kurtosis   se
## X1    1 2040 45.22 15.23     45   45.01 17.79  18  90    72 0.12    -0.96 0.34
```

```
age.hist <- ggplot(data2_wide, aes(s_age)) + geom_histogram(colour = "black",
                                                            fill = "white")+
  labs(x = "Age", y = "Frequency")
age.hist
```

```
## `stat_bin()` using `bins = 30`. Pick better value with `binwidth`.
```

```
## Warning: Removed 1 rows containing non-finite values (`stat_bin()`).
```

```
data2_wide$age_group <- ifelse(data2_wide$s_age < 45, "low", "high")
data2_wide$age_group <- as.factor(data2_wide$age_group)
table(data2_wide$age_group)
```

```
## 
## high  low 
## 1024 1016
```

```
prop.table(table(data2_wide$age_group))
```

```
## 
##      high       low 
## 0.5019608 0.4980392
```

## Participant’s Educational Background

```
table(data2_wide$s_school)
```

```
## 
## Haupt  Real   Abi 
##   685   681   675
```

```
prop.table(table(data2_wide$s_school))
```

```
## 
##     Haupt      Real       Abi 
## 0.3356198 0.3336600 0.3307202
```

## Quota

```
table(data2_wide$quota)
```

```
## 
##   1   2   3   4   5   6   7   8   9  10  11  12 
## 169 171 174 168 171 172 168 167 164 170 172 175
```

## Awareness Check

```
table(data2_wide$s_awareness)
```

```
## 
## fail pass 
##  658 1383
```

```
prop.table(table(data2_wide$s_awareness))
```

```
## 
##     fail     pass 
## 0.322391 0.677609
```

```
table(data2_wide$s_awareness, data2_wide$condition)
```

```
##       
##          1   2   3   4   5   6
##   fail 113  94 116 135  90 110
##   pass 221 251 220 206 238 247
```

```
awareness_bar <- ggplot(data2_wide, aes(x = condition, fill = s_awareness))
awareness_bar <- awareness_bar + geom_bar() + theme_classic() + theme(
  panel.grid.major = element_blank(),
  panel.grid.minor = element_blank(),
  panel.background = element_blank(),
  axis.title = element_text(face = "bold"),
  text = element_text(face = "bold"),
  axis.text = element_text(face = "bold"),
  legend.title = element_text(face = "bold"))+
  labs(x = "Condition", y = "Number of Cases", fill = "Awareness Check") +
  scale_fill_brewer(palette = "Blues")
awareness_bar
```

```
CrossTable(data2_wide$condition, data2_wide$s_awareness,
           chisq = TRUE, expected = TRUE, sresid = TRUE, format = "SPSS")
```

```
## 
##    Cell Contents
## |-------------------------|
## |                   Count |
## |         Expected Values |
## | Chi-square contribution |
## |             Row Percent |
## |          Column Percent |
## |           Total Percent |
## |            Std Residual |
## |-------------------------|
## 
## Total Observations in Table:  2041 
## 
##                      | data2_wide$s_awareness 
## data2_wide$condition |     fail  |     pass  | Row Total | 
## ---------------------|-----------|-----------|-----------|
##                    1 |      113  |      221  |      334  | 
##                      |  107.679  |  226.321  |           | 
##                      |    0.263  |    0.125  |           | 
##                      |   33.832% |   66.168% |   16.365% | 
##                      |   17.173% |   15.980% |           | 
##                      |    5.537% |   10.828% |           | 
##                      |    0.513  |   -0.354  |           | 
## ---------------------|-----------|-----------|-----------|
##                    2 |       94  |      251  |      345  | 
##                      |  111.225  |  233.775  |           | 
##                      |    2.668  |    1.269  |           | 
##                      |   27.246% |   72.754% |   16.903% | 
##                      |   14.286% |   18.149% |           | 
##                      |    4.606% |   12.298% |           | 
##                      |   -1.633  |    1.127  |           | 
## ---------------------|-----------|-----------|-----------|
##                    3 |      116  |      220  |      336  | 
##                      |  108.323  |  227.677  |           | 
##                      |    0.544  |    0.259  |           | 
##                      |   34.524% |   65.476% |   16.463% | 
##                      |   17.629% |   15.907% |           | 
##                      |    5.683% |   10.779% |           | 
##                      |    0.738  |   -0.509  |           | 
## ---------------------|-----------|-----------|-----------|
##                    4 |      135  |      206  |      341  | 
##                      |  109.935  |  231.065  |           | 
##                      |    5.715  |    2.719  |           | 
##                      |   39.589% |   60.411% |   16.707% | 
##                      |   20.517% |   14.895% |           | 
##                      |    6.614% |   10.093% |           | 
##                      |    2.391  |   -1.649  |           | 
## ---------------------|-----------|-----------|-----------|
##                    5 |       90  |      238  |      328  | 
##                      |  105.744  |  222.256  |           | 
##                      |    2.344  |    1.115  |           | 
##                      |   27.439% |   72.561% |   16.071% | 
##                      |   13.678% |   17.209% |           | 
##                      |    4.410% |   11.661% |           | 
##                      |   -1.531  |    1.056  |           | 
## ---------------------|-----------|-----------|-----------|
##                    6 |      110  |      247  |      357  | 
##                      |  115.094  |  241.906  |           | 
##                      |    0.225  |    0.107  |           | 
##                      |   30.812% |   69.188% |   17.491% | 
##                      |   16.717% |   17.860% |           | 
##                      |    5.390% |   12.102% |           | 
##                      |   -0.475  |    0.327  |           | 
## ---------------------|-----------|-----------|-----------|
##         Column Total |      658  |     1383  |     2041  | 
##                      |   32.239% |   67.761% |           | 
## ---------------------|-----------|-----------|-----------|
## 
##  
## Statistics for All Table Factors
## 
## 
## Pearson's Chi-squared test 
## ------------------------------------------------------------
## Chi^2 =  17.35328     d.f. =  5     p =  0.003876309 
## 
## 
##  
##        Minimum expected frequency: 105.7442
```

```
fisher.test(data2_wide$condition, data2_wide$s_awareness, workspace = 2e8)
```

```
## 
##  Fisher's Exact Test for Count Data
## 
## data:  data2_wide$condition and data2_wide$s_awareness
## p-value = 0.004063
## alternative hypothesis: two.sided
```

```
CrossTable(data2_wide$s_sex, data2_wide$s_awareness,
           chisq = TRUE, expected = TRUE, sresid = TRUE, format = "SPSS")
```

```
## 
##    Cell Contents
## |-------------------------|
## |                   Count |
## |         Expected Values |
## | Chi-square contribution |
## |             Row Percent |
## |          Column Percent |
## |           Total Percent |
## |            Std Residual |
## |-------------------------|
## 
## Total Observations in Table:  2041 
## 
##                  | data2_wide$s_awareness 
## data2_wide$s_sex |     fail  |     pass  | Row Total | 
## -----------------|-----------|-----------|-----------|
##           female |      301  |      727  |     1028  | 
##                  |  331.418  |  696.582  |           | 
##                  |    2.792  |    1.328  |           | 
##                  |   29.280% |   70.720% |   50.367% | 
##                  |   45.745% |   52.567% |           | 
##                  |   14.748% |   35.620% |           | 
##                  |   -1.671  |    1.153  |           | 
## -----------------|-----------|-----------|-----------|
##             male |      357  |      656  |     1013  | 
##                  |  326.582  |  686.418  |           | 
##                  |    2.833  |    1.348  |           | 
##                  |   35.242% |   64.758% |   49.633% | 
##                  |   54.255% |   47.433% |           | 
##                  |   17.491% |   32.141% |           | 
##                  |    1.683  |   -1.161  |           | 
## -----------------|-----------|-----------|-----------|
##     Column Total |      658  |     1383  |     2041  | 
##                  |   32.239% |   67.761% |           | 
## -----------------|-----------|-----------|-----------|
## 
##  
## Statistics for All Table Factors
## 
## 
## Pearson's Chi-squared test 
## ------------------------------------------------------------
## Chi^2 =  8.30114     d.f. =  1     p =  0.003962019 
## 
## Pearson's Chi-squared test with Yates' continuity correction 
## ------------------------------------------------------------
## Chi^2 =  8.030481     d.f. =  1     p =  0.004599664 
## 
##  
##        Minimum expected frequency: 326.5821
```

```
fisher.test(data2_wide$s_sex, data2_wide$s_awareness, workspace = 2e8)
```

```
## 
##  Fisher's Exact Test for Count Data
## 
## data:  data2_wide$s_sex and data2_wide$s_awareness
## p-value = 0.004472
## alternative hypothesis: true odds ratio is not equal to 1
## 95 percent confidence interval:
##  0.6287428 0.9204853
## sample estimates:
## odds ratio 
##  0.7609049
```

```
data2_wide$s_age_1 <- ifelse(data2_wide$s_age < 45, 0, 1)
age_hist <- ggplot(data2_wide, aes(x = s_age, fill = s_awareness, color = s_awareness))
age_hist <- age_hist + geom_histogram(alpha = 0.1, position = "identity") + theme_classic() + theme(
  panel.grid.major = element_blank(),
  panel.grid.minor = element_blank(),
  panel.background = element_blank(),
  axis.title = element_text(face = "bold"),
  text = element_text(face = "bold"),
  axis.text = element_text(face = "bold"),
  legend.title = element_text(face = "bold"))+
  labs(x = "Age", y = "Number of Cases", fill = "Awareness Check", color = "Awareness Check") + 
  scale_fill_brewer(palette = "Dark2") + scale_color_brewer(palette = "Dark2")
age_hist
```

```
## `stat_bin()` using `bins = 30`. Pick better value with `binwidth`.
```

```
## Warning: Removed 1 rows containing non-finite values (`stat_bin()`).
```

```
CrossTable(data2_wide$s_age_1, data2_wide$s_awareness,
           chisq = TRUE, expected = TRUE, sresid = TRUE, format = "SPSS")
```

```
## 
##    Cell Contents
## |-------------------------|
## |                   Count |
## |         Expected Values |
## | Chi-square contribution |
## |             Row Percent |
## |          Column Percent |
## |           Total Percent |
## |            Std Residual |
## |-------------------------|
## 
## Total Observations in Table:  2040 
## 
##                    | data2_wide$s_awareness 
## data2_wide$s_age_1 |     fail  |     pass  | Row Total | 
## -------------------|-----------|-----------|-----------|
##                  0 |      411  |      605  |     1016  | 
##                    |  327.710  |  688.290  |           | 
##                    |   21.169  |   10.079  |           | 
##                    |   40.453% |   59.547% |   49.804% | 
##                    |   62.462% |   43.777% |           | 
##                    |   20.147% |   29.657% |           | 
##                    |    4.601  |   -3.175  |           | 
## -------------------|-----------|-----------|-----------|
##                  1 |      247  |      777  |     1024  | 
##                    |  330.290  |  693.710  |           | 
##                    |   21.004  |   10.000  |           | 
##                    |   24.121% |   75.879% |   50.196% | 
##                    |   37.538% |   56.223% |           | 
##                    |   12.108% |   38.088% |           | 
##                    |   -4.583  |    3.162  |           | 
## -------------------|-----------|-----------|-----------|
##       Column Total |      658  |     1382  |     2040  | 
##                    |   32.255% |   67.745% |           | 
## -------------------|-----------|-----------|-----------|
## 
##  
## Statistics for All Table Factors
## 
## 
## Pearson's Chi-squared test 
## ------------------------------------------------------------
## Chi^2 =  62.25162     d.f. =  1     p =  3.022599e-15 
## 
## Pearson's Chi-squared test with Yates' continuity correction 
## ------------------------------------------------------------
## Chi^2 =  61.50646     d.f. =  1     p =  4.412932e-15 
## 
##  
##        Minimum expected frequency: 327.7098
```

```
fisher.test(data2_wide$s_age_1, data2_wide$s_awareness, workspace = 2e8)
```

```
## 
##  Fisher's Exact Test for Count Data
## 
## data:  data2_wide$s_age_1 and data2_wide$s_awareness
## p-value = 2.97e-15
## alternative hypothesis: true odds ratio is not equal to 1
## 95 percent confidence interval:
##  1.759109 2.597653
## sample estimates:
## odds ratio 
##   2.136236
```

```
school_bar <- ggplot(data2_wide,aes(x = s_school, fill = s_awareness))
school_bar <- school_bar +  geom_bar() + theme_classic() + theme(
  panel.grid.major = element_blank(),
  panel.grid.minor = element_blank(),
  panel.background = element_blank(),
  axis.title = element_text(face = "bold"),
  text = element_text(face = "bold"),
  axis.text = element_text(face = "bold"),
  legend.title = element_text(face = "bold"))+
  labs(x = "Schooltype", y = "Number of Cases", fill = "Awareness Check") +
  scale_fill_brewer(palette = "Blues")
school_bar
```

```
CrossTable(data2_wide$s_school, data2_wide$s_awareness,
           chisq = TRUE, expected = TRUE, sresid = TRUE, format = "SPSS")
```

```
## 
##    Cell Contents
## |-------------------------|
## |                   Count |
## |         Expected Values |
## | Chi-square contribution |
## |             Row Percent |
## |          Column Percent |
## |           Total Percent |
## |            Std Residual |
## |-------------------------|
## 
## Total Observations in Table:  2041 
## 
##                     | data2_wide$s_awareness 
## data2_wide$s_school |     fail  |     pass  | Row Total | 
## --------------------|-----------|-----------|-----------|
##               Haupt |      270  |      415  |      685  | 
##                     |  220.838  |  464.162  |           | 
##                     |   10.944  |    5.207  |           | 
##                     |   39.416% |   60.584% |   33.562% | 
##                     |   41.033% |   30.007% |           | 
##                     |   13.229% |   20.333% |           | 
##                     |    3.308  |   -2.282  |           | 
## --------------------|-----------|-----------|-----------|
##                Real |      220  |      461  |      681  | 
##                     |  219.548  |  461.452  |           | 
##                     |    0.001  |    0.000  |           | 
##                     |   32.305% |   67.695% |   33.366% | 
##                     |   33.435% |   33.333% |           | 
##                     |   10.779% |   22.587% |           | 
##                     |    0.030  |   -0.021  |           | 
## --------------------|-----------|-----------|-----------|
##                 Abi |      168  |      507  |      675  | 
##                     |  217.614  |  457.386  |           | 
##                     |   11.312  |    5.382  |           | 
##                     |   24.889% |   75.111% |   33.072% | 
##                     |   25.532% |   36.659% |           | 
##                     |    8.231% |   24.841% |           | 
##                     |   -3.363  |    2.320  |           | 
## --------------------|-----------|-----------|-----------|
##        Column Total |      658  |     1383  |     2041  | 
##                     |   32.239% |   67.761% |           | 
## --------------------|-----------|-----------|-----------|
## 
##  
## Statistics for All Table Factors
## 
## 
## Pearson's Chi-squared test 
## ------------------------------------------------------------
## Chi^2 =  32.84601     d.f. =  2     p =  7.371908e-08 
## 
## 
##  
##        Minimum expected frequency: 217.6139
```

```
fisher.test(data2_wide$s_school, data2_wide$s_awareness, workspace = 2e8)
```

```
## 
##  Fisher's Exact Test for Count Data
## 
## data:  data2_wide$s_school and data2_wide$s_awareness
## p-value = 6.61e-08
## alternative hypothesis: two.sided
```

## Accessibility

```
describe(data2_wide$accessibility_1)
```

```
##    vars    n mean   sd median trimmed  mad min max range  skew kurtosis   se
## X1    1 2029 5.64 1.79      6    5.77 1.48   1   8     7 -0.45    -0.52 0.04
```

```
describe(data2_wide$accessibility_2)
```

```
##    vars    n mean   sd median trimmed  mad min max range skew kurtosis   se
## X1    1 2016 5.43 1.86      6    5.54 1.48   1   8     7 -0.4    -0.55 0.04
```

```
dep.access.test <- wilcox.test(data2_wide$accessibility_1,
                               data2_wide$accessibility_2,
                               paired = TRUE,
                               correct = TRUE)
dep.access.test
```

```
## 
##  Wilcoxon signed rank test with continuity correction
## 
## data:  data2_wide$accessibility_1 and data2_wide$accessibility_2
## V = 486685, p-value = 1.394e-09
## alternative hypothesis: true location shift is not equal to 0
```

```
data2_wide$accessibility <- rowMeans(data2_wide[,c("accessibility_1",
                                                   "accessibility_2")])
describe(data2_wide$accessibility)
```

```
##    vars    n mean   sd median trimmed  mad min max range  skew kurtosis   se
## X1    1 2005 5.54 1.64    5.5    5.61 1.48   1   8     7 -0.37    -0.43 0.04
```

```
access.hist <- ggplot(data2_wide, aes(accessibility)) + 
  geom_histogram(colour = "black", fill = "white") + labs(x = "Mean Accessibility",
                                                          y = "Frequency")
access.hist
```

```
## `stat_bin()` using `bins = 30`. Pick better value with `binwidth`.
```

```
## Warning: Removed 36 rows containing non-finite values (`stat_bin()`).
```

## Understanding

```
describe(data2_wide$understanding_1)
```

```
##    vars    n mean   sd median trimmed  mad min max range  skew kurtosis   se
## X1    1 2021 5.78 1.66      6    5.89 1.48   1   8     7 -0.47    -0.36 0.04
```

```
describe(data2_wide$understanding_2)
```

```
##    vars    n mean   sd median trimmed  mad min max range  skew kurtosis   se
## X1    1 2017 5.41 1.73      6    5.49 1.48   1   8     7 -0.42    -0.34 0.04
```

```
dep.understand.test <- wilcox.test(data2_wide$understanding_1,
                               data2_wide$understanding_2,
                               paired = TRUE,
                               correct = TRUE)
dep.understand.test
```

```
## 
##  Wilcoxon signed rank test with continuity correction
## 
## data:  data2_wide$understanding_1 and data2_wide$understanding_2
## V = 571930, p-value < 2.2e-16
## alternative hypothesis: true location shift is not equal to 0
```

```
data2_wide$understanding <- rowMeans(data2_wide[,c("understanding_1",
                                                   "understanding_2")])
describe(data2_wide$understanding)
```

```
##    vars    n mean  sd median trimmed  mad min max range skew kurtosis   se
## X1    1 1998  5.6 1.5    5.5    5.66 1.48   1   8     7 -0.4    -0.28 0.03
```

```
understand.hist <- ggplot(data2_wide, aes(understanding)) + 
  geom_histogram(colour = "black", fill = "white") + labs(x = "Mean Understanding",
                                                          y = "Frequency")
understand.hist
```

```
## `stat_bin()` using `bins = 30`. Pick better value with `binwidth`.
```

```
## Warning: Removed 43 rows containing non-finite values (`stat_bin()`).
```

## Empowerment

```
describe(data2_wide$empowerment_1)
```

```
##    vars    n mean  sd median trimmed  mad min max range  skew kurtosis   se
## X1    1 2018 4.85 1.8      5    4.89 1.48   1   8     7 -0.19    -0.48 0.04
```

```
describe(data2_wide$empowerment_2)
```

```
##    vars    n mean   sd median trimmed  mad min max range  skew kurtosis   se
## X1    1 2021 4.71 1.82      5    4.75 1.48   1   8     7 -0.18    -0.48 0.04
```

```
dep.emp.test <- wilcox.test(data2_wide$empowerment_1,
                            data2_wide$empowerment_2,
                            paired = TRUE,
                            correct = TRUE)
dep.emp.test
```

```
## 
##  Wilcoxon signed rank test with continuity correction
## 
## data:  data2_wide$empowerment_1 and data2_wide$empowerment_2
## V = 475021, p-value = 0.0001433
## alternative hypothesis: true location shift is not equal to 0
```

```
data2_wide$empowerment <- rowMeans(data2_wide[,c("empowerment_1",
                                                 "empowerment_2")])
describe(data2_wide$empowerment)
```

```
##    vars    n mean   sd median trimmed  mad min max range  skew kurtosis   se
## X1    1 2000 4.77 1.63      5    4.81 1.48   1   8     7 -0.18    -0.35 0.04
```

```
empower.hist <- ggplot(data2_wide, aes(empowerment)) + 
  geom_histogram(colour = "black", fill = "white") + labs(x = "Mean Empowerment",
                                                          y = "Frequency")
empower.hist
```

```
## `stat_bin()` using `bins = 30`. Pick better value with `binwidth`.
```

```
## Warning: Removed 41 rows containing non-finite values (`stat_bin()`).
```

## Credibility

```
data2_wide$credibility <- rowMeans(data2_wide[,c("credibility_1",
                                                 "credibility_2")])

describe(data2_wide$credibility_1)
```

```
##    vars    n mean   sd median trimmed  mad min max range  skew kurtosis   se
## X1    1 2020 5.91 1.52      6    5.97 1.48   1   8     7 -0.35    -0.48 0.03
```

```
describe(data2_wide$credibility_2)
```

```
##    vars    n mean   sd median trimmed  mad min max range  skew kurtosis   se
## X1    1 2018 5.77 1.58      6    5.85 1.48   1   8     7 -0.43    -0.17 0.04
```

```
dep.credible.test <- wilcox.test(data2_wide$credibility_1,
                               data2_wide$credibility_2,
                               paired = TRUE,
                               correct = TRUE)
dep.credible.test
```

```
## 
##  Wilcoxon signed rank test with continuity correction
## 
## data:  data2_wide$credibility_1 and data2_wide$credibility_2
## V = 391005, p-value = 0.0001058
## alternative hypothesis: true location shift is not equal to 0
```

```
describe(data2_wide$credibility)
```

```
##    vars    n mean   sd median trimmed  mad min max range  skew kurtosis   se
## X1    1 1997 5.84 1.37      6    5.87 1.48   1   8     7 -0.25    -0.47 0.03
```

```
credible.hist <- ggplot(data2_wide, aes(credibility)) + 
  geom_histogram(colour = "black", fill = "white") + labs(x = "Mean Credibility",
                                                          y = "Frequency")
credible.hist
```

```
## `stat_bin()` using `bins = 30`. Pick better value with `binwidth`.
```

```
## Warning: Removed 44 rows containing non-finite values (`stat_bin()`).
```

## Relevance

```
data2_wide$relevance <- rowMeans(data2_wide[,c("relevance_1","relevance_2")])
describe(data2_wide$relevance_1)
```

```
##    vars    n mean   sd median trimmed  mad min max range  skew kurtosis   se
## X1    1 2024 6.33 1.58      7    6.51 1.48   1   8     7 -0.78     0.06 0.04
```

```
describe(data2_wide$relevance_2)
```

```
##    vars    n mean   sd median trimmed  mad min max range skew kurtosis   se
## X1    1 2019 6.19 1.67      6    6.37 1.48   1   8     7 -0.8     0.15 0.04
```

```
dep.relevance.test <- wilcox.test(data2_wide$relevance_1,
                                 data2_wide$relevance_2,
                                 paired = TRUE,
                                 correct = TRUE)
dep.relevance.test
```

```
## 
##  Wilcoxon signed rank test with continuity correction
## 
## data:  data2_wide$relevance_1 and data2_wide$relevance_2
## V = 383964, p-value = 5.793e-05
## alternative hypothesis: true location shift is not equal to 0
```

```
describe(data2_wide$relevance)
```

```
##    vars    n mean   sd median trimmed  mad min max range  skew kurtosis   se
## X1    1 2003 6.26 1.42    6.5    6.39 1.48   1   8     7 -0.62    -0.14 0.03
```

```
relevance.hist <- ggplot(data2_wide, aes(relevance)) + 
  geom_histogram(colour = "black", fill = "white") + labs(x = "Mean Relevance",
                                                          y = "Frequency")
relevance.hist
```

```
## `stat_bin()` using `bins = 30`. Pick better value with `binwidth`.
```

```
## Warning: Removed 38 rows containing non-finite values (`stat_bin()`).
```

## Curiosity

```
data2_wide$curiosity <- rowMeans(data2_wide[,c("curiosity_1",
                                               "curiosity_2")])
describe(data2_wide$curiosity_1)
```

```
##    vars    n mean  sd median trimmed  mad min max range  skew kurtosis   se
## X1    1 2030 3.32 1.1      3    3.36 1.48   1   5     4 -0.29    -0.58 0.02
```

```
describe(data2_wide$curiosity_2)
```

```
##    vars    n mean   sd median trimmed  mad min max range  skew kurtosis   se
## X1    1 2025 3.16 1.15      3    3.18 1.48   1   5     4 -0.12    -0.77 0.03
```

```
dep.curiosity.test <- wilcox.test(data2_wide$curiosity_1,
                                  data2_wide$curiosity_2,
                                  paired = TRUE,
                                  correct = TRUE)
dep.curiosity.test
```

```
## 
##  Wilcoxon signed rank test with continuity correction
## 
## data:  data2_wide$curiosity_1 and data2_wide$curiosity_2
## V = 313831, p-value = 1.291e-12
## alternative hypothesis: true location shift is not equal to 0
```

```
describe(data2_wide$curiosity)
```

```
##    vars    n mean sd median trimmed  mad min max range  skew kurtosis   se
## X1    1 2014 3.24  1      3    3.25 1.48   1   5     4 -0.12    -0.54 0.02
```

```
curiosity.hist <- ggplot(data2_wide, aes(curiosity)) + 
  geom_histogram(colour = "black", fill = "white") + labs(x = "Mean Curiosity",
                                                          y = "Frequency")
curiosity.hist
```

```
## `stat_bin()` using `bins = 30`. Pick better value with `binwidth`.
```

```
## Warning: Removed 27 rows containing non-finite values (`stat_bin()`).
```

## Boredom

```
data2_wide$boredom <- rowMeans(data2_wide[,c("boredom_1",
                                             "boredom_2")])
describe(data2_wide$boredom_1)
```

```
##    vars    n mean   sd median trimmed  mad min max range skew kurtosis   se
## X1    1 2023 2.04 1.09      2    1.89 1.48   1   5     4 0.82    -0.08 0.02
```

```
describe(data2_wide$boredom_2)
```

```
##    vars    n mean   sd median trimmed  mad min max range skew kurtosis   se
## X1    1 2022 2.04 1.14      2    1.88 1.48   1   5     4 0.87    -0.11 0.03
```

```
dep.boredom.test <- wilcox.test(data2_wide$boredom_1,
                                  data2_wide$boredom_2,
                                  paired = TRUE,
                                  correct = TRUE)
dep.boredom.test
```

```
## 
##  Wilcoxon signed rank test with continuity correction
## 
## data:  data2_wide$boredom_1 and data2_wide$boredom_2
## V = 181847, p-value = 0.6904
## alternative hypothesis: true location shift is not equal to 0
```

```
describe(data2_wide$boredom)
```

```
##    vars    n mean   sd median trimmed  mad min max range skew kurtosis   se
## X1    1 2006 2.04 0.99      2    1.92 1.48   1   5     4 0.81     0.12 0.02
```

```
boredom.hist <- ggplot(data2_wide, aes(boredom)) + 
  geom_histogram(colour = "black", fill = "white") + labs(x = "Mean Boredom",
                                                          y = "Frequency")
boredom.hist
```

```
## `stat_bin()` using `bins = 30`. Pick better value with `binwidth`.
```

```
## Warning: Removed 35 rows containing non-finite values (`stat_bin()`).
```

## Confusion

```
data2_wide$confusion <- rowMeans(data2_wide[,c("confusion_1",
                                             "confusion_2")])
describe(data2_wide$confusion_1)
```

```
##    vars    n mean   sd median trimmed  mad min max range skew kurtosis   se
## X1    1 2025  2.1 1.05      2    1.97 1.48   1   5     4 0.67    -0.25 0.02
```

```
describe(data2_wide$confusion_2)
```

```
##    vars    n mean  sd median trimmed  mad min max range skew kurtosis   se
## X1    1 2024 2.15 1.1      2    2.02 1.48   1   5     4 0.69     -0.3 0.02
```

```
dep.confusion.test <- wilcox.test(data2_wide$confusion_1,
                                data2_wide$confusion_2,
                                paired = TRUE,
                                correct = TRUE)
dep.confusion.test
```

```
## 
##  Wilcoxon signed rank test with continuity correction
## 
## data:  data2_wide$confusion_1 and data2_wide$confusion_2
## V = 246237, p-value = 0.1284
## alternative hypothesis: true location shift is not equal to 0
```

```
describe(data2_wide$confusion)
```

```
##    vars    n mean   sd median trimmed  mad min max range skew kurtosis   se
## X1    1 2008 2.12 0.93      2    2.03 0.74   1   5     4 0.61    -0.17 0.02
```

```
confusion.hist <- ggplot(data2_wide, aes(confusion)) + 
  geom_histogram(colour = "black", fill = "white") + labs(x = "Mean Confusion",
                                                          y = "Frequency")
confusion.hist
```

```
## `stat_bin()` using `bins = 30`. Pick better value with `binwidth`.
```

```
## Warning: Removed 33 rows containing non-finite values (`stat_bin()`).
```

## Frustration

```
data2_wide$frustration <- rowMeans(data2_wide[,c("frustration_1",
                                               "frustration_2")])
describe(data2_wide$frustration_1)
```

```
##    vars    n mean sd median trimmed mad min max range skew kurtosis   se
## X1    1 2020 1.72  1      1    1.55   0   1   5     4 1.22     0.62 0.02
```

```
describe(data2_wide$frustration_2)
```

```
##    vars    n mean   sd median trimmed mad min max range skew kurtosis   se
## X1    1 2024  1.8 1.07      1    1.62   0   1   5     4  1.2     0.58 0.02
```

```
dep.frustration.test <- wilcox.test(data2_wide$frustration_1,
                                  data2_wide$frustration_2,
                                  paired = TRUE,
                                  correct = TRUE)
dep.frustration.test
```

```
## 
##  Wilcoxon signed rank test with continuity correction
## 
## data:  data2_wide$frustration_1 and data2_wide$frustration_2
## V = 122672, p-value = 0.001244
## alternative hypothesis: true location shift is not equal to 0
```

```
describe(data2_wide$frustration)
```

```
##    vars    n mean   sd median trimmed  mad min max range skew kurtosis   se
## X1    1 2004 1.76 0.91    1.5    1.62 0.74   1   5     4 1.11     0.55 0.02
```

```
frustration.hist <- ggplot(data2_wide, aes(frustration)) + 
  geom_histogram(colour = "black", fill = "white") + labs(x = "Mean Frustration",
                                                          y = "Frequency")
frustration.hist
```

```
## `stat_bin()` using `bins = 30`. Pick better value with `binwidth`.
```

```
## Warning: Removed 37 rows containing non-finite values (`stat_bin()`).
```

## Relationship Knowledge-Item

```
describe(data2_wide$s_relationship)
```

```
##    vars    n mean   sd median trimmed  mad min max range skew kurtosis   se
## X1    1 1979 0.23 3.22      0    0.02 2.97  -8   8    16 0.45    -0.37 0.07
```

```
table(data2_wide$s_relationship)
```

```
## 
##  -8  -7  -6  -5  -4  -3  -2  -1   0   1   2   3   4   5   6   7   8 
##   4   1  20  20 247 110 251 163 425 112 192  67 141  30 135  17  44
```

```
relationship.hist <- ggplot(data2_wide, aes(s_relationship)) + 
  geom_histogram(colour = "black", fill = "white") + labs(
    x = "Mean Relationship Knowledge Score", y = "Frequency") +
  scale_x_continuous(breaks = seq(-8,8,1))
relationship.hist
```

```
## `stat_bin()` using `bins = 30`. Pick better value with `binwidth`.
```

```
## Warning: Removed 62 rows containing non-finite values (`stat_bin()`).
```

## Extent of Evaluation Knowledge-Item

```
describe(data2_wide$s_extent)
```

```
##    vars    n mean   sd median trimmed  mad min max range skew kurtosis   se
## X1    1 1999 0.52 2.28      0    0.41 2.97  -6   6    12 0.35    -0.28 0.05
```

```
extent.hist <- ggplot(data2_wide, aes(s_extent)) + 
  geom_histogram(colour = "black", fill = "white") + labs(
    x = "Mean Extent Knowledge Score",
    y = "Frequency") +
  scale_x_continuous(breaks = seq(-6,6,1))
extent.hist
```

```
## `stat_bin()` using `bins = 30`. Pick better value with `binwidth`.
```

```
## Warning: Removed 42 rows containing non-finite values (`stat_bin()`).
```

## Differentiation Knowledge-Item

```
data2_wide$s_diff <- coalesce(data2_wide$s_diff_1,data2_wide$s_diff_2)
describe(data2_wide$s_diff_1)
```

```
##    vars   n mean   sd median trimmed  mad min max range skew kurtosis   se
## X1    1 997 0.07 1.83      0    0.05 1.48  -6   6    12 0.14     0.68 0.06
```

```
describe(data2_wide$s_diff_2)
```

```
##    vars   n mean   sd median trimmed  mad min max range skew kurtosis   se
## X1    1 981 0.52 2.07      0    0.46 2.97  -6   6    12 0.15     0.23 0.07
```

```
dep.diff.test <- wilcox.test(data2_wide$s_diff_1,
                                data2_wide$s_diff_2,
                                    paired = FALSE,
                                    correct = TRUE)
dep.diff.test
```

```
## 
##  Wilcoxon rank sum test with continuity correction
## 
## data:  data2_wide$s_diff_1 and data2_wide$s_diff_2
## W = 428088, p-value = 8.067e-07
## alternative hypothesis: true location shift is not equal to 0
```

```
describe(data2_wide$s_diff)
```

```
##    vars    n mean   sd median trimmed  mad min max range skew kurtosis   se
## X1    1 1978 0.29 1.97      0    0.23 1.48  -6   6    12 0.19     0.46 0.04
```

```
diff.hist <- ggplot(data2_wide, aes(s_diff)) + 
  geom_histogram(colour = "black", fill = "white") + labs(
    x = "Mean Differentiation Knowledge Score",
    y = "Frequency") +
  scale_x_continuous(breaks = seq(-6,6,1))
diff.hist
```

```
## `stat_bin()` using `bins = 30`. Pick better value with `binwidth`.
```

```
## Warning: Removed 63 rows containing non-finite values (`stat_bin()`).
```

## Funding Knowledge-Item

```
data2_wide$s_funding <- rowSums(data2_wide[,c("s_funding_1",
                                               "s_funding_2")])
describe(data2_wide$s_funding_1)
```

```
##    vars    n mean   sd median trimmed  mad min max range skew kurtosis   se
## X1    1 1994 0.87 3.05      0    0.84 2.97  -6   6    12 0.26    -0.74 0.07
```

```
describe(data2_wide$s_funding_2)
```

```
##    vars    n mean   sd median trimmed  mad min max range  skew kurtosis   se
## X1    1 2010 1.67 3.22      1    1.82 4.45  -6   6    12 -0.07    -1.03 0.07
```

```
dep.funding.test <- wilcox.test(data2_wide$s_funding_1,
                                data2_wide$s_funding_2,
                                    paired = TRUE,
                                    correct = TRUE)
dep.funding.test
```

```
## 
##  Wilcoxon signed rank test with continuity correction
## 
## data:  data2_wide$s_funding_1 and data2_wide$s_funding_2
## V = 321887, p-value < 2.2e-16
## alternative hypothesis: true location shift is not equal to 0
```

```
describe(data2_wide$s_funding)
```

```
##    vars    n mean   sd median trimmed  mad min max range skew kurtosis   se
## X1    1 1965 2.55 5.42      2    2.53 5.93 -12  12    24 0.07    -0.74 0.12
```

```
funding.hist <- ggplot(data2_wide, aes(s_funding)) + 
  geom_histogram(colour = "black", fill = "white") + labs(
    x = "Mean Funding Knowledge Score",
    y = "Frequency") +
  scale_x_continuous(breaks = seq(-12,12,1))
funding.hist
```

```
## `stat_bin()` using `bins = 30`. Pick better value with `binwidth`.
```

```
## Warning: Removed 76 rows containing non-finite values (`stat_bin()`).
```

## COI Knowledge-Item

```
data2_wide$s_coi <- rowSums(data2_wide[,c("s_coi_1",
                                               "s_coi_2")])
describe(data2_wide$s_coi_1)
```

```
##    vars    n mean   sd median trimmed  mad min max range skew kurtosis   se
## X1    1 1955 0.72 3.33      0    0.66 2.97  -7   7    14 0.19     -0.5 0.08
```

```
describe(data2_wide$s_coi_2)
```

```
##    vars    n mean   sd median trimmed  mad min max range  skew kurtosis   se
## X1    1 1993  1.3 3.56      1    1.36 2.97  -7   7    14 -0.01    -0.75 0.08
```

```
dep.coi.test <- wilcox.test(data2_wide$s_coi_1,
                                data2_wide$s_coi_2,
                                paired = TRUE,
                                correct = TRUE)
dep.coi.test
```

```
## 
##  Wilcoxon signed rank test with continuity correction
## 
## data:  data2_wide$s_coi_1 and data2_wide$s_coi_2
## V = 401472, p-value = 1.122e-12
## alternative hypothesis: true location shift is not equal to 0
```

```
describe(data2_wide$s_coi)
```

```
##    vars    n mean   sd median trimmed  mad min max range  skew kurtosis   se
## X1    1 1914 2.02 5.88      1    2.13 5.93 -13  14    27 -0.05    -0.59 0.13
```

```
coi.hist <- ggplot(data2_wide, aes(s_coi)) + 
  geom_histogram(colour = "black", fill = "white") + labs(
    x = "Mean COI Knowledge Score",
    y = "Frequency") +
  scale_x_continuous(breaks = seq(-14,14,1))
coi.hist
```

```
## `stat_bin()` using `bins = 30`. Pick better value with `binwidth`.
```

```
## Warning: Removed 127 rows containing non-finite values (`stat_bin()`).
```

## Causality Knowledge-Item

```
data2_wide$s_causality <- rowSums(data2_wide[,c("s_causality_1",
                                                 "s_causality_2")])
describe(data2_wide$s_causality_1)
```

```
##    vars    n  mean   sd median trimmed  mad min max range skew kurtosis   se
## X1    1 1989 -0.43 2.45      0   -0.52 2.97  -6   6    12  0.2    -0.56 0.06
```

```
describe(data2_wide$s_causality_2)
```

```
##    vars    n  mean   sd median trimmed  mad min max range skew kurtosis   se
## X1    1 1995 -0.21 2.45      0   -0.29 2.97  -6   6    12 0.21    -0.31 0.05
```

```
dep.causality.test <- wilcox.test(data2_wide$s_causality_1,
                            data2_wide$s_causality_2,
                            paired = TRUE,
                            correct = TRUE)
dep.causality.test
```

```
## 
##  Wilcoxon signed rank test with continuity correction
## 
## data:  data2_wide$s_causality_1 and data2_wide$s_causality_2
## V = 486523, p-value = 0.0008355
## alternative hypothesis: true location shift is not equal to 0
```

```
describe(data2_wide$s_causality)
```

```
##    vars    n  mean  sd median trimmed  mad min max range skew kurtosis   se
## X1    1 1949 -0.64 3.9      0   -0.67 2.97 -12  12    24  0.1    -0.15 0.09
```

```
causality.hist <- ggplot(data2_wide, aes(s_causality)) + 
  geom_histogram(colour = "black", fill = "white") + labs(
    x = "Mean Causality Knowledge Score",
    y = "Frequency") +
  scale_x_continuous(breaks = seq(-12,12,1))
causality.hist
```

```
## `stat_bin()` using `bins = 30`. Pick better value with `binwidth`.
```

```
## Warning: Removed 92 rows containing non-finite values (`stat_bin()`).
```

## CAMA Knowledge-Items

```
describe(data2_wide$s_CAMA_1)
```

```
##    vars   n mean   sd median trimmed  mad min max range skew kurtosis   se
## X1    1 984  0.7 2.66      0    0.68 2.97  -7   8    15 0.16    -0.02 0.08
```

```
describe(data2_wide$s_CAMA_2)
```

```
##    vars    n mean   sd median trimmed  mad min max range skew kurtosis   se
## X1    1 1015 -0.4 1.62      0   -0.44 1.48  -4   4     8 0.18     0.05 0.05
```

```
describe(data2_wide$s_CAMA_3)
```

```
##    vars    n  mean   sd median trimmed  mad min max range skew kurtosis   se
## X1    1 1026 -0.14 0.75      0   -0.17 1.48  -1   1     2 0.23    -1.21 0.02
```

```
describe(data2_wide$s_CAMA)
```

```
##    vars   n mean   sd median trimmed  mad min max range skew kurtosis   se
## X1    1 975 0.17 3.73      0    0.17 2.97 -11  13    24 0.09     0.13 0.12
```

```
CAMA1.hist <- ggplot(data2_wide, aes(s_CAMA_1)) + 
  geom_histogram(colour = "black", fill = "white") + labs(
    x = "Mean CAMA Knowledge Score",
    y = "Frequency") +
  scale_x_continuous(breaks = seq(-8,8,1))
CAMA1.hist
```

```
## `stat_bin()` using `bins = 30`. Pick better value with `binwidth`.
```

```
## Warning: Removed 1057 rows containing non-finite values (`stat_bin()`).
```

```
CAMA2.hist <- ggplot(data2_wide, aes(s_CAMA_2)) + 
  geom_histogram(colour = "black", fill = "white") + labs(
    x = "Mean CAMA Knowledge Score",
    y = "Frequency") +
  scale_x_continuous(breaks = seq(-4,4,1))
CAMA2.hist
```

```
## `stat_bin()` using `bins = 30`. Pick better value with `binwidth`.
```

```
## Warning: Removed 1026 rows containing non-finite values (`stat_bin()`).
```

```
CAMA3.hist <- ggplot(data2_wide, aes(s_CAMA_3)) + 
  geom_histogram(colour = "black", fill = "white") + labs(
    x = "Mean CAMA Knowledge Score",
    y = "Frequency") +
  scale_x_continuous(breaks = seq(-1,1,1))
CAMA3.hist
```

```
## `stat_bin()` using `bins = 30`. Pick better value with `binwidth`.
```

```
## Warning: Removed 1015 rows containing non-finite values (`stat_bin()`).
```

```
CAMA_oa.hist <- ggplot(data2_wide, aes(s_CAMA)) + 
  geom_histogram(colour = "black", fill = "white") + labs(
    x = "Mean CAMA Knowledge Score",
    y = "Frequency") +
  scale_x_continuous(breaks = seq(-13,13,1))
CAMA_oa.hist
```

```
## `stat_bin()` using `bins = 30`. Pick better value with `binwidth`.
```

```
## Warning: Removed 1066 rows containing non-finite values (`stat_bin()`).
```

# Hypotheses-Testing

## H1

### H1a

```
H1a <- subset(data2_wide, condition == 2|condition == 4|condition == 6)
View(H1a)
describeBy(H1a$s_relationship,H1a$disclaimer)
```

```
## 
##  Descriptive statistics by group 
## group: no disclaimer
##    vars   n mean   sd median trimmed  mad min max range skew kurtosis   se
## X1    1 346 0.06 2.91      0   -0.05 2.97  -8   8    16 0.28    -0.22 0.16
## ------------------------------------------------------------ 
## group: disclaimer
##    vars   n mean   sd median trimmed  mad min max range skew kurtosis   se
## X1    1 668 0.39 3.45      0    0.16 2.97  -8   8    16 0.48    -0.53 0.13
```

```
wilcox.test(s_relationship~disclaimer, data = H1a, exact = FALSE,
            conf.int = TRUE)
```

```
## 
##  Wilcoxon rank sum test with continuity correction
## 
## data:  s_relationship by disclaimer
## W = 112365, p-value = 0.4656
## alternative hypothesis: true location shift is not equal to 0
## 95 percent confidence interval:
##  -2.622830e-05  2.762469e-05
## sample estimates:
## difference in location 
##          -6.597433e-05
```

#### H1a post hoc

```
H1a_1 <- subset(data2_wide, condition ==2| condition == 6)
View(H1a_1)
describeBy(H1a_1$s_relationship, H1a_1$disclaimer)
```

```
## 
##  Descriptive statistics by group 
## group: no disclaimer
##    vars   n mean   sd median trimmed  mad min max range skew kurtosis   se
## X1    1 346 0.06 2.91      0   -0.05 2.97  -8   8    16 0.28    -0.22 0.16
## ------------------------------------------------------------ 
## group: disclaimer
##    vars   n mean   sd median trimmed  mad min max range skew kurtosis   se
## X1    1 336 0.24 3.44      0   -0.04 2.97  -6   8    14 0.57    -0.55 0.19
```

```
wilcox.test(s_relationship~disclaimer, data = H1a_1, exaxct = FALSE,
            conf.int = TRUE)
```

```
## 
##  Wilcoxon rank sum test with continuity correction
## 
## data:  s_relationship by disclaimer
## W = 58323, p-value = 0.9392
## alternative hypothesis: true location shift is not equal to 0
## 95 percent confidence interval:
##  -4.084067e-05  5.691002e-05
## sample estimates:
## difference in location 
##           2.295351e-05
```

```
H1a_2 <- subset(data2_wide, condition ==4| condition == 6)
View(H1a_2)
describeBy(H1a_2$s_relationship, H1a_2$disclaimer)
```

```
## 
##  Descriptive statistics by group 
## group: no disclaimer
##    vars   n mean   sd median trimmed  mad min max range skew kurtosis   se
## X1    1 346 0.06 2.91      0   -0.05 2.97  -8   8    16 0.28    -0.22 0.16
## ------------------------------------------------------------ 
## group: disclaimer
##    vars   n mean   sd median trimmed  mad min max range skew kurtosis   se
## X1    1 332 0.53 3.45      0    0.37 2.97  -8   8    16  0.4     -0.5 0.19
```

```
wilcox.test(s_relationship~disclaimer, data = H1a_2, exaxct = FALSE,
            conf.int = TRUE)
```

```
## 
##  Wilcoxon rank sum test with continuity correction
## 
## data:  s_relationship by disclaimer
## W = 54042, p-value = 0.1789
## alternative hypothesis: true location shift is not equal to 0
## 95 percent confidence interval:
##  -9.999601e-01  5.534757e-05
## sample estimates:
## difference in location 
##          -2.700176e-05
```

### H1b

```
H1b <- subset(data2_wide, condition == 1|condition == 2|condition == 3|
                condition == 4)
View(H1b)
describeBy(H1b$s_relationship,H1b$disclaimer)
```

```
## 
##  Descriptive statistics by group 
## group: no disclaimer
##    vars   n mean   sd median trimmed  mad min max range skew kurtosis   se
## X1    1 646 0.07 3.08      0   -0.08 2.97  -8   8    16 0.34    -0.37 0.12
## ------------------------------------------------------------ 
## group: disclaimer
##    vars   n mean   sd median trimmed  mad min max range skew kurtosis   se
## X1    1 668 0.39 3.45      0    0.16 2.97  -8   8    16 0.48    -0.53 0.13
```

```
wilcox.test(s_relationship~disclaimer, data = H1b, exact = FALSE,
            conf.int = TRUE)
```

```
## 
##  Wilcoxon rank sum test with continuity correction
## 
## data:  s_relationship by disclaimer
## W = 208967, p-value = 0.3191
## alternative hypothesis: true location shift is not equal to 0
## 95 percent confidence interval:
##  -3.794751e-05  3.878243e-05
## sample estimates:
## difference in location 
##          -6.124734e-06
```

#### H1b post hoc

```
H1b_1 <- subset(data2_wide, condition == 1|condition == 2)
View(H1b_1)
describeBy(H1b_1$s_relationship,H1b_1$disclaimer)
```

```
## 
##  Descriptive statistics by group 
## group: no disclaimer
##    vars   n mean   sd median trimmed  mad min max range skew kurtosis   se
## X1    1 322 0.22 3.11      0    0.12 2.97  -8   8    16 0.18    -0.53 0.17
## ------------------------------------------------------------ 
## group: disclaimer
##    vars   n mean   sd median trimmed  mad min max range skew kurtosis   se
## X1    1 336 0.24 3.44      0   -0.04 2.97  -6   8    14 0.57    -0.55 0.19
```

```
wilcox.test(s_relationship~disclaimer, data = H1b_1, exact = FALSE,
            conf.int = TRUE)
```

```
## 
##  Wilcoxon rank sum test with continuity correction
## 
## data:  s_relationship by disclaimer
## W = 55565, p-value = 0.5441
## alternative hypothesis: true location shift is not equal to 0
## 95 percent confidence interval:
##  -6.470564e-05  9.999129e-01
## sample estimates:
## difference in location 
##           1.755468e-05
```

```
H1b_2 <- subset(data2_wide, condition == 3|condition == 4)
View(H1b_2)
describeBy(H1b_2$s_relationship,H1b_2$disclaimer)
```

```
## 
##  Descriptive statistics by group 
## group: no disclaimer
##    vars   n  mean   sd median trimmed  mad min max range skew kurtosis   se
## X1    1 324 -0.08 3.06      0   -0.29 2.97  -6   8    14  0.5    -0.17 0.17
## ------------------------------------------------------------ 
## group: disclaimer
##    vars   n mean   sd median trimmed  mad min max range skew kurtosis   se
## X1    1 332 0.53 3.45      0    0.37 2.97  -8   8    16  0.4     -0.5 0.19
```

```
wilcox.test(s_relationship~disclaimer, data = H1b_2, exact = FALSE,
            conf.int = TRUE)
```

```
## 
##  Wilcoxon rank sum test with continuity correction
## 
## data:  s_relationship by disclaimer
## W = 48896, p-value = 0.04208
## alternative hypothesis: true location shift is not equal to 0
## 95 percent confidence interval:
##  -9.999832e-01 -7.580566e-05
## sample estimates:
## difference in location 
##          -2.674227e-05
```

```
H1b_3 <- subset(data2_wide, condition == 1|condition == 4)
View(H1b_3)
describeBy(H1b_3$s_relationship,H1b_3$disclaimer)
```

```
## 
##  Descriptive statistics by group 
## group: no disclaimer
##    vars   n mean   sd median trimmed  mad min max range skew kurtosis   se
## X1    1 322 0.22 3.11      0    0.12 2.97  -8   8    16 0.18    -0.53 0.17
## ------------------------------------------------------------ 
## group: disclaimer
##    vars   n mean   sd median trimmed  mad min max range skew kurtosis   se
## X1    1 332 0.53 3.45      0    0.37 2.97  -8   8    16  0.4     -0.5 0.19
```

```
wilcox.test(s_relationship~disclaimer, data = H1b_3, exact = FALSE,
            conf.int = TRUE)
```

```
## 
##  Wilcoxon rank sum test with continuity correction
## 
## data:  s_relationship by disclaimer
## W = 51756, p-value = 0.4789
## alternative hypothesis: true location shift is not equal to 0
## 95 percent confidence interval:
##  -9.999557e-01  3.933794e-05
## sample estimates:
## difference in location 
##          -8.397794e-06
```

```
H1b_4 <- subset(data2_wide, condition == 2|condition == 3)
View(H1b_4)
describeBy(H1b_4$s_relationship,H1b_4$disclaimer)
```

```
## 
##  Descriptive statistics by group 
## group: no disclaimer
##    vars   n  mean   sd median trimmed  mad min max range skew kurtosis   se
## X1    1 324 -0.08 3.06      0   -0.29 2.97  -6   8    14  0.5    -0.17 0.17
## ------------------------------------------------------------ 
## group: disclaimer
##    vars   n mean   sd median trimmed  mad min max range skew kurtosis   se
## X1    1 336 0.24 3.44      0   -0.04 2.97  -6   8    14 0.57    -0.55 0.19
```

```
wilcox.test(s_relationship~disclaimer, data = H1b_4, exact = FALSE,
            conf.int = TRUE)
```

```
## 
##  Wilcoxon rank sum test with continuity correction
## 
## data:  s_relationship by disclaimer
## W = 52751, p-value = 0.4892
## alternative hypothesis: true location shift is not equal to 0
## 95 percent confidence interval:
##  -9.999562e-01  5.642902e-05
## sample estimates:
## difference in location 
##          -3.622986e-06
```

### H1 Logistic Regression

```
# Exclude NAs for stepwise testing
sum(is.na(data2_wide$disclaimer))
```

```
## [1] 0
```

```
sum(is.na(data2_wide$s_awareness))
```

```
## [1] 0
```

```
sum(is.na(data2_wide$text_order))
```

```
## [1] 0
```

```
sum(is.na(data2_wide$s_age))
```

```
## [1] 1
```

```
data2_wide <- data2_wide %>% drop_na(s_age)
sum(is.na(data2_wide$s_sex))
```

```
## [1] 0
```

```
sum(is.na(data2_wide$s_school))
```

```
## [1] 0
```

```
sum(is.na(data2_wide$s_interest))
```

```
## [1] 0
```

```
data2_wide$H1_interaction <- interaction(data2_wide$disclaimer,
                                         data2_wide$version)
data2_wide$H1_interaction <- droplevels(data2_wide$H1_interaction)
table(data2_wide$H1_interaction)
```

```
## 
## no disclaimer.old guideline no disclaimer.new guideline 
##                         357                         670 
##    disclaimer.new guideline 
##                        1013
```

```
data2_wide_reg <- subset(data2_wide, condition != 5)
View(data2_wide_reg)

relationship_null <- clm(as.factor(s_relationship)~1, data = data2_wide_reg,
                         link = "logit")

relationship_model1 <- clm(as.factor(s_relationship)~ H1_interaction,
                           data = data2_wide_reg, link = "logit")
anova(relationship_null,relationship_model1)
```

```
## Likelihood ratio tests of cumulative link models:
##  
##                     formula:                                   link: threshold:
## relationship_null   as.factor(s_relationship) ~ 1              logit flexible  
## relationship_model1 as.factor(s_relationship) ~ H1_interaction logit flexible  
## 
##                     no.par    AIC  logLik LR.stat df Pr(>Chisq)
## relationship_null       16 8008.9 -3988.4                      
## relationship_model1     18 8011.7 -3987.9  1.1372  2     0.5663
```

```
relationship_model2 <- clm(as.factor(s_relationship)~ H1_interaction +
                             s_awareness, data = data2_wide_reg, link = "logit")
anova(relationship_null,relationship_model2)
```

```
## Likelihood ratio tests of cumulative link models:
##  
##                     formula:                                                
## relationship_null   as.factor(s_relationship) ~ 1                           
## relationship_model2 as.factor(s_relationship) ~ H1_interaction + s_awareness
##                     link: threshold:
## relationship_null   logit flexible  
## relationship_model2 logit flexible  
## 
##                     no.par    AIC  logLik LR.stat df Pr(>Chisq)    
## relationship_null       16 8008.9 -3988.4                          
## relationship_model2     19 7959.4 -3960.7  55.441  3  5.529e-12 ***
## ---
## Signif. codes:  0 '***' 0.001 '**' 0.01 '*' 0.05 '.' 0.1 ' ' 1
```

```
relationship_model3 <- clm(as.factor(s_relationship)~ 
                             H1_interaction*s_awareness, data = data2_wide_reg,
                           link = "logit")
anova(relationship_model2,relationship_model3)
```

```
## Likelihood ratio tests of cumulative link models:
##  
##                     formula:                                                
## relationship_model2 as.factor(s_relationship) ~ H1_interaction + s_awareness
## relationship_model3 as.factor(s_relationship) ~ H1_interaction * s_awareness
##                     link: threshold:
## relationship_model2 logit flexible  
## relationship_model3 logit flexible  
## 
##                     no.par    AIC  logLik LR.stat df Pr(>Chisq)
## relationship_model2     19 7959.4 -3960.7                      
## relationship_model3     21 7962.5 -3960.2  0.9571  2     0.6197
```

```
relationship_model4 <- clm(as.factor(s_relationship)~ 
                             H1_interaction*s_awareness + summary1, 
                           data = data2_wide_reg, link = "logit")
anova(relationship_model3,relationship_model4)
```

```
## Likelihood ratio tests of cumulative link models:
##  
##                     formula:                                                           
## relationship_model3 as.factor(s_relationship) ~ H1_interaction * s_awareness           
## relationship_model4 as.factor(s_relationship) ~ H1_interaction * s_awareness + summary1
##                     link: threshold:
## relationship_model3 logit flexible  
## relationship_model4 logit flexible  
## 
##                     no.par    AIC  logLik LR.stat df Pr(>Chisq)
## relationship_model3     21 7962.5 -3960.2                      
## relationship_model4     22 7963.6 -3959.8  0.8892  1     0.3457
```

```
relationship_model5 <- clm(as.factor(s_relationship)~ 
                             H1_interaction*s_awareness + summary1 
                           + s_age, data = data2_wide_reg,
                           link = "logit")
anova(relationship_model4,relationship_model5)
```

```
## Likelihood ratio tests of cumulative link models:
##  
##                     formula:                                                                   
## relationship_model4 as.factor(s_relationship) ~ H1_interaction * s_awareness + summary1        
## relationship_model5 as.factor(s_relationship) ~ H1_interaction * s_awareness + summary1 + s_age
##                     link: threshold:
## relationship_model4 logit flexible  
## relationship_model5 logit flexible  
## 
##                     no.par    AIC  logLik LR.stat df Pr(>Chisq)    
## relationship_model4     22 7963.6 -3959.8                          
## relationship_model5     23 7879.4 -3916.7  86.179  1  < 2.2e-16 ***
## ---
## Signif. codes:  0 '***' 0.001 '**' 0.01 '*' 0.05 '.' 0.1 ' ' 1
```

```
relationship_model6 <- clm(as.factor(s_relationship)~ 
                             H1_interaction*s_awareness + summary1 
                           + s_age + s_sex, data = data2_wide_reg,
                           link = "logit")
anova(relationship_model5,relationship_model6)
```

```
## Likelihood ratio tests of cumulative link models:
##  
##                     formula:                                                                           
## relationship_model5 as.factor(s_relationship) ~ H1_interaction * s_awareness + summary1 + s_age        
## relationship_model6 as.factor(s_relationship) ~ H1_interaction * s_awareness + summary1 + s_age + s_sex
##                     link: threshold:
## relationship_model5 logit flexible  
## relationship_model6 logit flexible  
## 
##                     no.par    AIC  logLik LR.stat df Pr(>Chisq)
## relationship_model5     23 7879.4 -3916.7                      
## relationship_model6     24 7881.1 -3916.6  0.2543  1     0.6141
```

```
relationship_model7 <- clm(as.factor(s_relationship)~ 
                             H1_interaction*s_awareness + summary1 +
                             s_age + s_sex + s_school, 
                           data = data2_wide_reg, link = "logit")
anova(relationship_model5,relationship_model7)
```

```
## Likelihood ratio tests of cumulative link models:
##  
##                     formula:                                                                                      
## relationship_model5 as.factor(s_relationship) ~ H1_interaction * s_awareness + summary1 + s_age                   
## relationship_model7 as.factor(s_relationship) ~ H1_interaction * s_awareness + summary1 + s_age + s_sex + s_school
##                     link: threshold:
## relationship_model5 logit flexible  
## relationship_model7 logit flexible  
## 
##                     no.par    AIC  logLik LR.stat df Pr(>Chisq)    
## relationship_model5     23 7879.4 -3916.7                          
## relationship_model7     26 7821.7 -3884.8  63.735  3   9.35e-14 ***
## ---
## Signif. codes:  0 '***' 0.001 '**' 0.01 '*' 0.05 '.' 0.1 ' ' 1
```

```
relationship_model8 <- clm(as.factor(s_relationship)~ 
                             H1_interaction*s_awareness + summary1 +
                             s_age + s_sex + s_school +
                             as.factor(s_interest), data = data2_wide_reg,
                           link = "logit")
anova(relationship_model7,relationship_model8)
```

```
## Likelihood ratio tests of cumulative link models:
##  
##                     formula:                                                                                                              
## relationship_model7 as.factor(s_relationship) ~ H1_interaction * s_awareness + summary1 + s_age + s_sex + s_school                        
## relationship_model8 as.factor(s_relationship) ~ H1_interaction * s_awareness + summary1 + s_age + s_sex + s_school + as.factor(s_interest)
##                     link: threshold:
## relationship_model7 logit flexible  
## relationship_model8 logit flexible  
## 
##                     no.par    AIC  logLik LR.stat df Pr(>Chisq)   
## relationship_model7     26 7821.7 -3884.8                         
## relationship_model8     30 7811.3 -3875.7  18.351  4   0.001054 **
## ---
## Signif. codes:  0 '***' 0.001 '**' 0.01 '*' 0.05 '.' 0.1 ' ' 1
```

```
summary(relationship_model8)
```

```
## formula: 
## as.factor(s_relationship) ~ H1_interaction * s_awareness + summary1 + s_age + s_sex + s_school + as.factor(s_interest)
## data:    data2_wide_reg
## 
##  link  threshold nobs logLik   AIC     niter max.grad cond.H 
##  logit flexible  1660 -3875.66 7811.31 9(2)  2.45e-12 1.3e+06
## 
## Coefficients:
##                                                            Estimate Std. Error
## H1_interactionno disclaimer.new guideline                  0.092030   0.200646
## H1_interactiondisclaimer.new guideline                     0.037610   0.200875
## s_awarenesspass                                            0.770701   0.200424
## summary1Faerber                                            0.084563   0.086509
## s_age                                                     -0.025094   0.002932
## s_sexmale                                                 -0.077937   0.087012
## s_schoolReal                                               0.309928   0.106545
## s_schoolAbi                                                0.873053   0.109980
## as.factor(s_interest)5                                     0.049578   0.132980
## as.factor(s_interest)6                                     0.072247   0.134347
## as.factor(s_interest)7                                    -0.029943   0.147005
## as.factor(s_interest)8                                    -0.442159   0.140765
## H1_interactionno disclaimer.new guideline:s_awarenesspass -0.163416   0.244856
## H1_interactiondisclaimer.new guideline:s_awarenesspass     0.078636   0.245433
##                                                           z value Pr(>|z|)    
## H1_interactionno disclaimer.new guideline                   0.459  0.64647    
## H1_interactiondisclaimer.new guideline                      0.187  0.85148    
## s_awarenesspass                                             3.845  0.00012 ***
## summary1Faerber                                             0.978  0.32832    
## s_age                                                      -8.560  < 2e-16 ***
## s_sexmale                                                  -0.896  0.37041    
## s_schoolReal                                                2.909  0.00363 ** 
## s_schoolAbi                                                 7.938 2.05e-15 ***
## as.factor(s_interest)5                                      0.373  0.70928    
## as.factor(s_interest)6                                      0.538  0.59074    
## as.factor(s_interest)7                                     -0.204  0.83860    
## as.factor(s_interest)8                                     -3.141  0.00168 ** 
## H1_interactionno disclaimer.new guideline:s_awarenesspass  -0.667  0.50452    
## H1_interactiondisclaimer.new guideline:s_awarenesspass      0.320  0.74867    
## ---
## Signif. codes:  0 '***' 0.001 '**' 0.01 '*' 0.05 '.' 0.1 ' ' 1
## 
## Threshold coefficients:
##       Estimate Std. Error z value
## -8|-7  -6.4870     0.5528 -11.734
## -7|-6  -6.2629     0.5056 -12.387
## -6|-5  -4.6803     0.3119 -15.008
## -5|-4  -4.0833     0.2807 -14.544
## -4|-3  -2.1374     0.2430  -8.797
## -3|-2  -1.7452     0.2402  -7.265
## -2|-1  -1.0550     0.2368  -4.455
## -1|0   -0.6615     0.2357  -2.807
## 0|1     0.2832     0.2352   1.204
## 1|2     0.5721     0.2355   2.429
## 2|3     1.1302     0.2372   4.766
## 3|4     1.3659     0.2383   5.731
## 4|5     1.9967     0.2432   8.211
## 5|6     2.2015     0.2454   8.970
## 6|7     3.4525     0.2726  12.665
## 7|8     3.8351     0.2887  13.284
## (53 Beobachtungen als fehlend gelöscht)
```

```
exp(coef(relationship_model8))
```

```
##                                                     -8|-7 
##                                               0.001523149 
##                                                     -7|-6 
##                                               0.001905707 
##                                                     -6|-5 
##                                               0.009275961 
##                                                     -5|-4 
##                                               0.016851824 
##                                                     -4|-3 
##                                               0.117966144 
##                                                     -3|-2 
##                                               0.174614003 
##                                                     -2|-1 
##                                               0.348181844 
##                                                      -1|0 
##                                               0.516085165 
##                                                       0|1 
##                                               1.327412450 
##                                                       1|2 
##                                               1.772012006 
##                                                       2|3 
##                                               3.096338894 
##                                                       3|4 
##                                               3.919213259 
##                                                       4|5 
##                                               7.364671907 
##                                                       5|6 
##                                               9.038596464 
##                                                       6|7 
##                                              31.579103646 
##                                                       7|8 
##                                              46.299122481 
##                 H1_interactionno disclaimer.new guideline 
##                                               1.096397874 
##                    H1_interactiondisclaimer.new guideline 
##                                               1.038326697 
##                                           s_awarenesspass 
##                                               2.161281741 
##                                           summary1Faerber 
##                                               1.088240988 
##                                                     s_age 
##                                               0.975217751 
##                                                 s_sexmale 
##                                               0.925022568 
##                                              s_schoolReal 
##                                               1.363327598 
##                                               s_schoolAbi 
##                                               2.394209013 
##                                    as.factor(s_interest)5 
##                                               1.050827415 
##                                    as.factor(s_interest)6 
##                                               1.074920924 
##                                    as.factor(s_interest)7 
##                                               0.970500604 
##                                    as.factor(s_interest)8 
##                                               0.642647248 
## H1_interactionno disclaimer.new guideline:s_awarenesspass 
##                                               0.849237482 
##    H1_interactiondisclaimer.new guideline:s_awarenesspass 
##                                               1.081809934
```

```
exp(confint(relationship_model8))
```

```
##                                                               2.5 %    97.5 %
## H1_interactionno disclaimer.new guideline                 0.7401323 1.6258461
## H1_interactiondisclaimer.new guideline                    0.7005461 1.5402705
## s_awarenesspass                                           1.4602271 3.2048311
## summary1Faerber                                           0.9185364 1.2893974
## s_age                                                     0.9696156 0.9808249
## s_sexmale                                                 0.7799476 1.0970176
## s_schoolReal                                              1.1065267 1.6802455
## s_schoolAbi                                               1.9307086 2.9715029
## as.factor(s_interest)5                                    0.8096677 1.3637648
## as.factor(s_interest)6                                    0.8260495 1.3988328
## as.factor(s_interest)7                                    0.7275112 1.2946699
## as.factor(s_interest)8                                    0.4875840 0.8467208
## H1_interactionno disclaimer.new guideline:s_awarenesspass 0.5252687 1.3719833
## H1_interactiondisclaimer.new guideline:s_awarenesspass    0.6684317 1.7498793
```

```
nagelkerke(fit = relationship_model8, null = relationship_null)
```

```
## $Models
##                                                                                                                                                            
## Model: "clm, as.factor(s_relationship) ~ H1_interaction * s_awareness + summary1 + s_age + s_sex + s_school + as.factor(s_interest), data2_wide_reg, logit"
## Null:  "clm, as.factor(s_relationship) ~ 1, data2_wide_reg, logit"                                                                                         
## 
## $Pseudo.R.squared.for.model.vs.null
##                              Pseudo.R.squared
## McFadden                            0.0282758
## Cox and Snell (ML)                  0.1270480
## Nagelkerke (Cragg and Uhler)        0.1280970
## 
## $Likelihood.ratio.test
##  Df.diff LogLik.diff  Chisq    p.value
##      -14     -112.78 225.55 3.1725e-40
## 
## $Number.of.observations
##            
## Model: 1660
## Null:  1660
## 
## $Messages
## [1] "Note: For models fit with REML, these statistics are based on refitting with ML"
## 
## $Warnings
## [1] "None"
```

```
H1test = emmeans(relationship_model8, ~ H1_interaction)
```

```
## NOTE: Results may be misleading due to involvement in interactions
```

```
pairs(H1test, adjust = "tukey")
```

```
##  contrast                                                  estimate    SE  df
##  no disclaimer.old guideline - no disclaimer.new guideline  -0.0103 0.122 Inf
##  no disclaimer.old guideline - disclaimer.new guideline     -0.0769 0.123 Inf
##  no disclaimer.new guideline - disclaimer.new guideline     -0.0666 0.102 Inf
##  z.ratio p.value
##   -0.084  0.9961
##   -0.627  0.8053
##   -0.652  0.7914
## 
## Results are averaged over the levels of: s_awareness, summary1, s_sex, s_school, s_interest 
## Note: contrasts are still on the as.factor scale 
## P value adjustment: tukey method for comparing a family of 3 estimates
```

```
cld(H1test, Letters = letters)
```

```
##  H1_interaction              emmean    SE  df asymp.LCL asymp.UCL .group
##  no disclaimer.old guideline  0.341 0.122 Inf     0.102     0.580  a    
##  no disclaimer.new guideline  0.351 0.101 Inf     0.153     0.549  a    
##  disclaimer.new guideline     0.418 0.101 Inf     0.220     0.616  a    
## 
## Results are averaged over the levels of: s_awareness, summary1, s_sex, s_school, s_interest 
## Results are given on the as.factor (not the response) scale. 
## Confidence level used: 0.95 
## Note: contrasts are still on the as.factor scale 
## P value adjustment: tukey method for comparing a family of 3 estimates 
## significance level used: alpha = 0.05 
## NOTE: If two or more means share the same grouping symbol,
##       then we cannot show them to be different.
##       But we also did not show them to be the same.
```

#### Logistic Regression by Group

```
data2_wide_reg1 <- subset(data2_wide_reg, condition == 2 | condition == 6)
View(data2_wide_reg1)

relationship_null_1 <- clm(as.factor(s_relationship)~1, data = data2_wide_reg1, link = "logit")

relationship_model8_1 <- clm(as.factor(s_relationship)~ 
                             H1_interaction*s_awareness + summary1 +
                             s_age + s_sex + s_school +
                             as.factor(s_interest), data = data2_wide_reg1, link = "logit")

summary(relationship_model8_1)
```

```
## formula: 
## as.factor(s_relationship) ~ H1_interaction * s_awareness + summary1 + s_age + s_sex + s_school + as.factor(s_interest)
## data:    data2_wide_reg1
## 
##  link  threshold nobs logLik   AIC     niter max.grad cond.H 
##  logit flexible  682  -1576.31 3208.62 8(1)  9.08e-08 1.0e+06
## 
## Coefficients:
##                                                         Estimate Std. Error
## H1_interactiondisclaimer.new guideline                 -0.020159   0.245631
## s_awarenesspass                                         0.783550   0.204978
## summary1Faerber                                         0.042646   0.135944
## s_age                                                  -0.028157   0.004545
## s_sexmale                                              -0.113214   0.137419
## s_schoolReal                                            0.173119   0.168318
## s_schoolAbi                                             0.831686   0.170260
## as.factor(s_interest)5                                  0.189489   0.208166
## as.factor(s_interest)6                                  0.311041   0.213707
## as.factor(s_interest)7                                 -0.015489   0.226762
## as.factor(s_interest)8                                 -0.362711   0.216145
## H1_interactiondisclaimer.new guideline:s_awarenesspass -0.047810   0.294064
##                                                        z value Pr(>|z|)    
## H1_interactiondisclaimer.new guideline                  -0.082 0.934592    
## s_awarenesspass                                          3.823 0.000132 ***
## summary1Faerber                                          0.314 0.753748    
## s_age                                                   -6.196 5.80e-10 ***
## s_sexmale                                               -0.824 0.410022    
## s_schoolReal                                             1.029 0.303706    
## s_schoolAbi                                              4.885 1.04e-06 ***
## as.factor(s_interest)5                                   0.910 0.362676    
## as.factor(s_interest)6                                   1.455 0.145544    
## as.factor(s_interest)7                                  -0.068 0.945544    
## as.factor(s_interest)8                                  -1.678 0.093329 .  
## H1_interactiondisclaimer.new guideline:s_awarenesspass  -0.163 0.870846    
## ---
## Signif. codes:  0 '***' 0.001 '**' 0.01 '*' 0.05 '.' 0.1 ' ' 1
## 
## Threshold coefficients:
##       Estimate Std. Error z value
## -8|-7  -7.1682     1.0459  -6.853
## -7|-6  -6.4740     0.7707  -8.400
## -6|-5  -5.3712     0.5105 -10.521
## -5|-4  -4.5115     0.4064 -11.100
## -4|-3  -2.2391     0.3170  -7.064
## -3|-2  -1.9020     0.3124  -6.088
## -2|-1  -1.2149     0.3053  -3.979
## -1|0   -0.7608     0.3024  -2.516
## 0|1     0.1479     0.3015   0.491
## 1|2     0.4680     0.3026   1.546
## 2|3     0.9964     0.3059   3.257
## 3|4     1.2663     0.3084   4.106
## 4|5     1.9165     0.3179   6.029
## 5|6     2.0589     0.3208   6.418
## 6|7     3.2745     0.3675   8.910
## 7|8     3.6994     0.3988   9.277
## (20 Beobachtungen als fehlend gelöscht)
```

```
exp(coef(relationship_model8_1))
```

```
##                                                  -8|-7 
##                                           7.706807e-04 
##                                                  -7|-6 
##                                           1.543060e-03 
##                                                  -6|-5 
##                                           4.648423e-03 
##                                                  -5|-4 
##                                           1.098187e-02 
##                                                  -4|-3 
##                                           1.065539e-01 
##                                                  -3|-2 
##                                           1.492630e-01 
##                                                  -2|-1 
##                                           2.967463e-01 
##                                                   -1|0 
##                                           4.672734e-01 
##                                                    0|1 
##                                           1.159442e+00 
##                                                    1|2 
##                                           1.596801e+00 
##                                                    2|3 
##                                           2.708518e+00 
##                                                    3|4 
##                                           3.547668e+00 
##                                                    4|5 
##                                           6.797181e+00 
##                                                    5|6 
##                                           7.837718e+00 
##                                                    6|7 
##                                           2.643053e+01 
##                                                    7|8 
##                                           4.042181e+01 
##                 H1_interactiondisclaimer.new guideline 
##                                           9.800432e-01 
##                                        s_awarenesspass 
##                                           2.189229e+00 
##                                        summary1Faerber 
##                                           1.043568e+00 
##                                                  s_age 
##                                           9.722360e-01 
##                                              s_sexmale 
##                                           8.929600e-01 
##                                           s_schoolReal 
##                                           1.189007e+00 
##                                            s_schoolAbi 
##                                           2.297188e+00 
##                                 as.factor(s_interest)5 
##                                           1.208631e+00 
##                                 as.factor(s_interest)6 
##                                           1.364845e+00 
##                                 as.factor(s_interest)7 
##                                           9.846307e-01 
##                                 as.factor(s_interest)8 
##                                           6.957877e-01 
## H1_interactiondisclaimer.new guideline:s_awarenesspass 
##                                           9.533150e-01
```

```
exp(confint(relationship_model8_1))
```

```
##                                                            2.5 %    97.5 %
## H1_interactiondisclaimer.new guideline                 0.6051809 1.5861761
## s_awarenesspass                                        1.4669237 3.2777771
## summary1Faerber                                        0.7994342 1.3623371
## s_age                                                  0.9635723 0.9808995
## s_sexmale                                              0.6820071 1.1689774
## s_schoolReal                                           0.8550335 1.6544106
## s_schoolAbi                                            1.6467673 3.2106536
## as.factor(s_interest)5                                 0.8034120 1.8176163
## as.factor(s_interest)6                                 0.8978204 2.0758112
## as.factor(s_interest)7                                 0.6311712 1.5360710
## as.factor(s_interest)8                                 0.4551834 1.0625466
## H1_interactiondisclaimer.new guideline:s_awarenesspass 0.5355740 1.6968891
```

```
nagelkerke(fit = relationship_model8_1, null = relationship_null_1)
```

```
## $Models
##                                                                                                                                                             
## Model: "clm, as.factor(s_relationship) ~ H1_interaction * s_awareness + summary1 + s_age + s_sex + s_school + as.factor(s_interest), data2_wide_reg1, logit"
## Null:  "clm, as.factor(s_relationship) ~ 1, data2_wide_reg1, logit"                                                                                         
## 
## $Pseudo.R.squared.for.model.vs.null
##                              Pseudo.R.squared
## McFadden                            0.0314367
## Cox and Snell (ML)                  0.1393230
## Nagelkerke (Cragg and Uhler)        0.1405110
## 
## $Likelihood.ratio.test
##  Df.diff LogLik.diff  Chisq    p.value
##      -12     -51.162 102.32 1.9488e-16
## 
## $Number.of.observations
##           
## Model: 682
## Null:  682
## 
## $Messages
## [1] "Note: For models fit with REML, these statistics are based on refitting with ML"
## 
## $Warnings
## [1] "None"
```

```
H1test_1 = emmeans(relationship_model8_1, ~ H1_interaction)
```

```
## NOTE: Results may be misleading due to involvement in interactions
```

```
pairs(H1test_1, adjust = "tukey")
```

```
##  contrast                                               estimate    SE  df
##  no disclaimer.old guideline - disclaimer.new guideline   0.0441 0.147 Inf
##  z.ratio p.value
##    0.299  0.7648
## 
## Results are averaged over the levels of: s_awareness, summary1, s_sex, s_school, s_interest 
## Note: contrasts are still on the as.factor scale
```

```
cld(H1test_1, Letters = letters)
```

```
##  H1_interaction              emmean    SE  df asymp.LCL asymp.UCL .group
##  disclaimer.new guideline     0.369 0.165 Inf    0.0460     0.691  a    
##  no disclaimer.old guideline  0.413 0.160 Inf    0.0991     0.726  a    
## 
## Results are averaged over the levels of: s_awareness, summary1, s_sex, s_school, s_interest 
## Results are given on the as.factor (not the response) scale. 
## Confidence level used: 0.95 
## Note: contrasts are still on the as.factor scale 
## significance level used: alpha = 0.05 
## NOTE: If two or more means share the same grouping symbol,
##       then we cannot show them to be different.
##       But we also did not show them to be the same.
```

```
data2_wide_reg2 <- subset(data2_wide_reg, condition == 4 | condition == 6)
View(data2_wide_reg2)

relationship_null_2 <- clm(as.factor(s_relationship)~1, data = data2_wide_reg2, link = "logit")

relationship_model8_2 <- clm(as.factor(s_relationship)~ 
                             H1_interaction*s_awareness + summary1 +
                             s_age + s_sex + s_school +
                             as.factor(s_interest), data = data2_wide_reg2, link = "logit")

summary(relationship_model8_2)
```

```
## formula: 
## as.factor(s_relationship) ~ H1_interaction * s_awareness + summary1 + s_age + s_sex + s_school + as.factor(s_interest)
## data:    data2_wide_reg2
## 
##  link  threshold nobs logLik   AIC     niter max.grad cond.H 
##  logit flexible  678  -1541.94 3139.88 10(3) 7.66e-13 8.9e+05
## 
## Coefficients:
##                                                         Estimate Std. Error
## H1_interactiondisclaimer.new guideline                  0.084544   0.226310
## s_awarenesspass                                         0.765877   0.205953
## summary1Faerber                                        -0.144396   0.136669
## s_age                                                  -0.024623   0.004634
## s_sexmale                                              -0.293743   0.136998
## s_schoolReal                                            0.316078   0.170422
## s_schoolAbi                                             0.827959   0.172419
## as.factor(s_interest)5                                  0.384884   0.206182
## as.factor(s_interest)6                                  0.168480   0.210609
## as.factor(s_interest)7                                  0.127651   0.229816
## as.factor(s_interest)8                                 -0.221542   0.215441
## H1_interactiondisclaimer.new guideline:s_awarenesspass  0.273848   0.284203
##                                                        z value Pr(>|z|)    
## H1_interactiondisclaimer.new guideline                   0.374   0.7087    
## s_awarenesspass                                          3.719   0.0002 ***
## summary1Faerber                                         -1.057   0.2907    
## s_age                                                   -5.313 1.08e-07 ***
## s_sexmale                                               -2.144   0.0320 *  
## s_schoolReal                                             1.855   0.0636 .  
## s_schoolAbi                                              4.802 1.57e-06 ***
## as.factor(s_interest)5                                   1.867   0.0619 .  
## as.factor(s_interest)6                                   0.800   0.4237    
## as.factor(s_interest)7                                   0.555   0.5786    
## as.factor(s_interest)8                                  -1.028   0.3038    
## H1_interactiondisclaimer.new guideline:s_awarenesspass   0.964   0.3353    
## ---
## Signif. codes:  0 '***' 0.001 '**' 0.01 '*' 0.05 '.' 0.1 ' ' 1
## 
## Threshold coefficients:
##       Estimate Std. Error z value
## -8|-7  -5.8942     0.6563  -8.981
## -7|-6  -5.6047     0.5894  -9.509
## -6|-5  -4.6779     0.4443 -10.528
## -5|-4  -4.1980     0.4000 -10.494
## -4|-3  -2.2286     0.3259  -6.838
## -3|-2  -1.8900     0.3209  -5.890
## -2|-1  -1.2040     0.3145  -3.828
## -1|0   -0.7364     0.3122  -2.359
## 0|1     0.3383     0.3112   1.087
## 1|2     0.6017     0.3119   1.929
## 2|3     1.1157     0.3143   3.550
## 3|4     1.3163     0.3158   4.168
## 4|5     1.9935     0.3242   6.149
## 5|6     2.1967     0.3280   6.697
## 6|7     3.6948     0.3900   9.475
## 7|8     3.7583     0.3947   9.523
## (20 Beobachtungen als fehlend gelöscht)
```

```
exp(coef(relationship_model8_2))
```

```
##                                                  -8|-7 
##                                            0.002755475 
##                                                  -7|-6 
##                                            0.003680616 
##                                                  -6|-5 
##                                            0.009298112 
##                                                  -5|-4 
##                                            0.015025709 
##                                                  -4|-3 
##                                            0.107679996 
##                                                  -3|-2 
##                                            0.151078243 
##                                                  -2|-1 
##                                            0.300002446 
##                                                   -1|0 
##                                            0.478824031 
##                                                    0|1 
##                                            1.402616547 
##                                                    1|2 
##                                            1.825187195 
##                                                    2|3 
##                                            3.051692428 
##                                                    3|4 
##                                            3.729733831 
##                                                    4|5 
##                                            7.340845989 
##                                                    5|6 
##                                            8.994906282 
##                                                    6|7 
##                                           40.236028259 
##                                                    7|8 
##                                           42.875244825 
##                 H1_interactiondisclaimer.new guideline 
##                                            1.088220611 
##                                        s_awarenesspass 
##                                            2.150879910 
##                                        summary1Faerber 
##                                            0.865544592 
##                                                  s_age 
##                                            0.975677234 
##                                              s_sexmale 
##                                            0.745467766 
##                                           s_schoolReal 
##                                            1.371737722 
##                                            s_schoolAbi 
##                                            2.288643582 
##                                 as.factor(s_interest)5 
##                                            1.469444424 
##                                 as.factor(s_interest)6 
##                                            1.183504958 
##                                 as.factor(s_interest)7 
##                                            1.136155948 
##                                 as.factor(s_interest)8 
##                                            0.801281937 
## H1_interactiondisclaimer.new guideline:s_awarenesspass 
##                                            1.315015242
```

```
exp(confint(relationship_model8_2))
```

```
##                                                            2.5 %    97.5 %
## H1_interactiondisclaimer.new guideline                 0.6982941 1.6964609
## s_awarenesspass                                        1.4382646 3.2260124
## summary1Faerber                                        0.6619509 1.1312632
## s_age                                                  0.9668233 0.9845555
## s_sexmale                                              0.5696816 0.9748339
## s_schoolReal                                           0.9825409 1.9168710
## s_schoolAbi                                            1.6338480 3.2125670
## as.factor(s_interest)5                                 0.9808999 2.2019295
## as.factor(s_interest)6                                 0.7832071 1.7889544
## as.factor(s_interest)7                                 0.7240196 1.7832371
## as.factor(s_interest)8                                 0.5250900 1.2223317
## H1_interactiondisclaimer.new guideline:s_awarenesspass 0.7533457 2.2961469
```

```
nagelkerke(fit = relationship_model8_2, null = relationship_null_2)
```

```
## $Models
##                                                                                                                                                             
## Model: "clm, as.factor(s_relationship) ~ H1_interaction * s_awareness + summary1 + s_age + s_sex + s_school + as.factor(s_interest), data2_wide_reg2, logit"
## Null:  "clm, as.factor(s_relationship) ~ 1, data2_wide_reg2, logit"                                                                                         
## 
## $Pseudo.R.squared.for.model.vs.null
##                              Pseudo.R.squared
## McFadden                            0.0343714
## Cox and Snell (ML)                  0.1494760
## Nagelkerke (Cragg and Uhler)        0.1508340
## 
## $Likelihood.ratio.test
##  Df.diff LogLik.diff  Chisq    p.value
##      -12     -54.885 109.77 6.6442e-18
## 
## $Number.of.observations
##           
## Model: 678
## Null:  678
## 
## $Messages
## [1] "Note: For models fit with REML, these statistics are based on refitting with ML"
## 
## $Warnings
## [1] "None"
```

```
H1test_2 = emmeans(relationship_model8_2, ~ H1_interaction)
```

```
## NOTE: Results may be misleading due to involvement in interactions
```

```
pairs(H1test_2, adjust = "tukey")
```

```
##  contrast                                               estimate    SE  df
##  no disclaimer.old guideline - disclaimer.new guideline   -0.221 0.142 Inf
##  z.ratio p.value
##   -1.558  0.1191
## 
## Results are averaged over the levels of: s_awareness, summary1, s_sex, s_school, s_interest 
## Note: contrasts are still on the as.factor scale
```

```
cld(H1test_2, Letters = letters)
```

```
##  H1_interaction              emmean    SE  df asymp.LCL asymp.UCL .group
##  no disclaimer.old guideline  0.222 0.137 Inf   -0.0456      0.49  a    
##  disclaimer.new guideline     0.444 0.136 Inf    0.1774      0.71  a    
## 
## Results are averaged over the levels of: s_awareness, summary1, s_sex, s_school, s_interest 
## Results are given on the as.factor (not the response) scale. 
## Confidence level used: 0.95 
## Note: contrasts are still on the as.factor scale 
## significance level used: alpha = 0.05 
## NOTE: If two or more means share the same grouping symbol,
##       then we cannot show them to be different.
##       But we also did not show them to be the same.
```

## H2

### H2a

```
H2a <- subset(data2_wide, condition == 2|condition == 4|condition == 6)
View(H2a)

describeBy(H2a$s_extent,H2a$disclaimer)
```

```
## 
##  Descriptive statistics by group 
## group: no disclaimer
##    vars   n mean   sd median trimmed  mad min max range skew kurtosis   se
## X1    1 349 0.52 2.23      0    0.42 2.97  -6   6    12 0.33    -0.23 0.12
## ------------------------------------------------------------ 
## group: disclaimer
##    vars   n mean  sd median trimmed  mad min max range skew kurtosis   se
## X1    1 676 0.62 2.4      0    0.54 2.97  -6   6    12 0.26     -0.5 0.09
```

```
wilcox.test(s_extent~disclaimer, data = H2a, exact = FALSE,
            conf.int = TRUE)
```

```
## 
##  Wilcoxon rank sum test with continuity correction
## 
## data:  s_extent by disclaimer
## W = 115603, p-value = 0.5948
## alternative hypothesis: true location shift is not equal to 0
## 95 percent confidence interval:
##  -7.469212e-05  8.124448e-05
## sample estimates:
## difference in location 
##          -4.862757e-05
```

#### H2a post hoc

```
H2a_1 <- subset(data2_wide, condition == 2|condition == 6)
View(H2a_1)

describeBy(H2a_1$s_extent,H2a_1$disclaimer)
```

```
## 
##  Descriptive statistics by group 
## group: no disclaimer
##    vars   n mean   sd median trimmed  mad min max range skew kurtosis   se
## X1    1 349 0.52 2.23      0    0.42 2.97  -6   6    12 0.33    -0.23 0.12
## ------------------------------------------------------------ 
## group: disclaimer
##    vars   n mean   sd median trimmed  mad min max range skew kurtosis   se
## X1    1 340 0.52 2.38      0    0.44 2.97  -6   6    12 0.22    -0.49 0.13
```

```
wilcox.test(s_extent~disclaimer, data = H2a_1, exact = FALSE,
            conf.int = TRUE)
```

```
## 
##  Wilcoxon rank sum test with continuity correction
## 
## data:  s_extent by disclaimer
## W = 59589, p-value = 0.9201
## alternative hypothesis: true location shift is not equal to 0
## 95 percent confidence interval:
##  -1.344322e-05  2.640857e-05
## sample estimates:
## difference in location 
##           7.842257e-05
```

```
H2a_2 <- subset(data2_wide, condition == 4|condition == 6)
View(H2a_2)

describeBy(H2a_2$s_extent,H2a_2$disclaimer)
```

```
## 
##  Descriptive statistics by group 
## group: no disclaimer
##    vars   n mean   sd median trimmed  mad min max range skew kurtosis   se
## X1    1 349 0.52 2.23      0    0.42 2.97  -6   6    12 0.33    -0.23 0.12
## ------------------------------------------------------------ 
## group: disclaimer
##    vars   n mean   sd median trimmed  mad min max range skew kurtosis   se
## X1    1 336 0.73 2.42      0    0.63 2.97  -4   6    10 0.31    -0.54 0.13
```

```
wilcox.test(s_extent~disclaimer, data = H2a_2, exact = FALSE,
            conf.int = TRUE)
```

```
## 
##  Wilcoxon rank sum test with continuity correction
## 
## data:  s_extent by disclaimer
## W = 56014, p-value = 0.3053
## alternative hypothesis: true location shift is not equal to 0
## 95 percent confidence interval:
##  -4.918730e-05  3.332259e-05
## sample estimates:
## difference in location 
##          -3.546894e-05
```

### H2b

```
H2b <- subset(data2_wide, condition == 1| condition == 2| condition == 3|
                condition == 4)
View(H2b)

describeBy(H2b$s_extent,H2b$disclaimer)
```

```
## 
##  Descriptive statistics by group 
## group: no disclaimer
##    vars   n mean   sd median trimmed  mad min max range skew kurtosis   se
## X1    1 654 0.41 2.15      0    0.29 2.97  -6   6    12 0.33    -0.19 0.08
## ------------------------------------------------------------ 
## group: disclaimer
##    vars   n mean  sd median trimmed  mad min max range skew kurtosis   se
## X1    1 676 0.62 2.4      0    0.54 2.97  -6   6    12 0.26     -0.5 0.09
```

```
wilcox.test(s_extent~disclaimer, data = H2b, exact = FALSE,
            conf.int = TRUE)
```

```
## 
##  Wilcoxon rank sum test with continuity correction
## 
## data:  s_extent by disclaimer
## W = 211224, p-value = 0.1547
## alternative hypothesis: true location shift is not equal to 0
## 95 percent confidence interval:
##  -5.113093e-05  3.383239e-05
## sample estimates:
## difference in location 
##          -9.912606e-05
```

#### H2b post hoc

```
H2b_1 <- subset(data2_wide, condition == 1| condition == 2)
View(H2b_1)
describeBy(H2b_1$s_extent,H2b_1$disclaimer)
```

```
## 
##  Descriptive statistics by group 
## group: no disclaimer
##    vars   n mean   sd median trimmed  mad min max range skew kurtosis   se
## X1    1 327 0.51 2.21      0    0.43 2.97  -4   6    10 0.25    -0.46 0.12
## ------------------------------------------------------------ 
## group: disclaimer
##    vars   n mean   sd median trimmed  mad min max range skew kurtosis   se
## X1    1 340 0.52 2.38      0    0.44 2.97  -6   6    12 0.22    -0.49 0.13
```

```
wilcox.test(s_extent~disclaimer, data = H2b_1, exact = FALSE,
            conf.int = TRUE)
```

```
## 
##  Wilcoxon rank sum test with continuity correction
## 
## data:  s_extent by disclaimer
## W = 55838, p-value = 0.9198
## alternative hypothesis: true location shift is not equal to 0
## 95 percent confidence interval:
##  -1.851467e-05  3.167506e-05
## sample estimates:
## difference in location 
##           4.181901e-05
```

```
H2b_2 <- subset(data2_wide, condition == 3| condition == 4)
View(H2b_2)
describeBy(H2b_2$s_extent,H2b_2$disclaimer)
```

```
## 
##  Descriptive statistics by group 
## group: no disclaimer
##    vars   n mean   sd median trimmed  mad min max range skew kurtosis   se
## X1    1 327  0.3 2.08      0    0.17 2.97  -6   6    12 0.41     0.14 0.11
## ------------------------------------------------------------ 
## group: disclaimer
##    vars   n mean   sd median trimmed  mad min max range skew kurtosis   se
## X1    1 336 0.73 2.42      0    0.63 2.97  -4   6    10 0.31    -0.54 0.13
```

```
wilcox.test(s_extent~disclaimer, data = H2b_2, exact = FALSE,
            conf.int = TRUE)
```

```
## 
##  Wilcoxon rank sum test with continuity correction
## 
## data:  s_extent by disclaimer
## W = 49672, p-value = 0.02996
## alternative hypothesis: true location shift is not equal to 0
## 95 percent confidence interval:
##  -9.999459e-01 -1.088538e-05
## sample estimates:
## difference in location 
##          -2.476233e-05
```

```
H2b_3 <- subset(data2_wide, condition == 1| condition == 4)
View(H2b_3)
describeBy(H2b_3$s_extent,H2b_3$disclaimer)
```

```
## 
##  Descriptive statistics by group 
## group: no disclaimer
##    vars   n mean   sd median trimmed  mad min max range skew kurtosis   se
## X1    1 327 0.51 2.21      0    0.43 2.97  -4   6    10 0.25    -0.46 0.12
## ------------------------------------------------------------ 
## group: disclaimer
##    vars   n mean   sd median trimmed  mad min max range skew kurtosis   se
## X1    1 336 0.73 2.42      0    0.63 2.97  -4   6    10 0.31    -0.54 0.13
```

```
wilcox.test(s_extent~disclaimer, data = H2b_3, exact = FALSE,
            conf.int = TRUE)
```

```
## 
##  Wilcoxon rank sum test with continuity correction
## 
## data:  s_extent by disclaimer
## W = 52652, p-value = 0.3482
## alternative hypothesis: true location shift is not equal to 0
## 95 percent confidence interval:
##  -2.863540e-05  3.467322e-05
## sample estimates:
## difference in location 
##          -3.262128e-05
```

```
H2b_4 <- subset(data2_wide, condition == 2| condition == 3)
View(H2b_4)
describeBy(H2b_4$s_extent,H2b_4$disclaimer)
```

```
## 
##  Descriptive statistics by group 
## group: no disclaimer
##    vars   n mean   sd median trimmed  mad min max range skew kurtosis   se
## X1    1 327  0.3 2.08      0    0.17 2.97  -6   6    12 0.41     0.14 0.11
## ------------------------------------------------------------ 
## group: disclaimer
##    vars   n mean   sd median trimmed  mad min max range skew kurtosis   se
## X1    1 340 0.52 2.38      0    0.44 2.97  -6   6    12 0.22    -0.49 0.13
```

```
wilcox.test(s_extent~disclaimer, data = H2b_4, exact = FALSE,
            conf.int = TRUE)
```

```
## 
##  Wilcoxon rank sum test with continuity correction
## 
## data:  s_extent by disclaimer
## W = 53062, p-value = 0.3021
## alternative hypothesis: true location shift is not equal to 0
## 95 percent confidence interval:
##  -5.449799e-05  3.884504e-05
## sample estimates:
## difference in location 
##          -6.112496e-05
```

### H2 Logistic Regression

```
data2_wide$H2_interaction <- data2_wide$H1_interaction
table(data2_wide$H2_interaction)
```

```
## 
## no disclaimer.old guideline no disclaimer.new guideline 
##                         357                         670 
##    disclaimer.new guideline 
##                        1013
```

```
data2_wide_reg <- subset(data2_wide, condition != 5)
View(data2_wide_reg)

extent_null <- clm(as.factor(s_extent) ~ 1, data = data2_wide_reg, link = "logit")

extent_model1 <- clm(as.factor(s_extent) ~ H2_interaction, data = data2_wide_reg,
                     link = "logit")
anova(extent_null,extent_model1)
```

```
## Likelihood ratio tests of cumulative link models:
##  
##               formula:                             link: threshold:
## extent_null   as.factor(s_extent) ~ 1              logit flexible  
## extent_model1 as.factor(s_extent) ~ H2_interaction logit flexible  
## 
##               no.par    AIC  logLik LR.stat df Pr(>Chisq)
## extent_null       12 7114.2 -3545.1                      
## extent_model1     14 7116.2 -3544.1  2.0399  2     0.3606
```

```
extent_model2 <- clm(as.factor(s_extent) ~ H2_interaction + s_awareness,
                     data = data2_wide_reg, link = "logit")
anova(extent_null,extent_model2)
```

```
## Likelihood ratio tests of cumulative link models:
##  
##               formula:                                           link:
## extent_null   as.factor(s_extent) ~ 1                            logit
## extent_model2 as.factor(s_extent) ~ H2_interaction + s_awareness logit
##               threshold:
## extent_null   flexible  
## extent_model2 flexible  
## 
##               no.par    AIC  logLik LR.stat df Pr(>Chisq)    
## extent_null       12 7114.2 -3545.1                          
## extent_model2     15 7028.5 -3499.3  91.681  3  < 2.2e-16 ***
## ---
## Signif. codes:  0 '***' 0.001 '**' 0.01 '*' 0.05 '.' 0.1 ' ' 1
```

```
extent_model3 <- clm(as.factor(s_extent) ~ H2_interaction*s_awareness,
                     data = data2_wide_reg, link = "logit")
anova(extent_model2,extent_model3)
```

```
## Likelihood ratio tests of cumulative link models:
##  
##               formula:                                           link:
## extent_model2 as.factor(s_extent) ~ H2_interaction + s_awareness logit
## extent_model3 as.factor(s_extent) ~ H2_interaction * s_awareness logit
##               threshold:
## extent_model2 flexible  
## extent_model3 flexible  
## 
##               no.par    AIC  logLik LR.stat df Pr(>Chisq)
## extent_model2     15 7028.5 -3499.3                      
## extent_model3     17 7028.8 -3497.4  3.7023  2     0.1571
```

```
extent_model4 <- clm(as.factor(s_extent) ~ H2_interaction*s_awareness + summary1,
                     data = data2_wide_reg, link = "logit")
anova(extent_model2,extent_model4)
```

```
## Likelihood ratio tests of cumulative link models:
##  
##               formula:                                                     
## extent_model2 as.factor(s_extent) ~ H2_interaction + s_awareness           
## extent_model4 as.factor(s_extent) ~ H2_interaction * s_awareness + summary1
##               link: threshold:
## extent_model2 logit flexible  
## extent_model4 logit flexible  
## 
##               no.par    AIC  logLik LR.stat df Pr(>Chisq)
## extent_model2     15 7028.5 -3499.3                      
## extent_model4     18 7030.6 -3497.3  3.9089  3     0.2715
```

```
extent_model5 <- clm(as.factor(s_extent) ~ H2_interaction*s_awareness + 
                       summary1 + s_age, data = data2_wide_reg, 
                     link = "logit")
anova(extent_model2,extent_model5)
```

```
## Likelihood ratio tests of cumulative link models:
##  
##               formula:                                                             
## extent_model2 as.factor(s_extent) ~ H2_interaction + s_awareness                   
## extent_model5 as.factor(s_extent) ~ H2_interaction * s_awareness + summary1 + s_age
##               link: threshold:
## extent_model2 logit flexible  
## extent_model5 logit flexible  
## 
##               no.par    AIC  logLik LR.stat df Pr(>Chisq)    
## extent_model2     15 7028.5 -3499.3                          
## extent_model5     19 7007.7 -3484.8  28.866  4  8.323e-06 ***
## ---
## Signif. codes:  0 '***' 0.001 '**' 0.01 '*' 0.05 '.' 0.1 ' ' 1
```

```
extent_model6 <- clm(as.factor(s_extent) ~ H2_interaction*s_awareness + 
                       summary1 + s_age + s_sex, 
                     data = data2_wide_reg, link = "logit")
anova(extent_model5,extent_model6)
```

```
## Likelihood ratio tests of cumulative link models:
##  
##               formula:                                                                     
## extent_model5 as.factor(s_extent) ~ H2_interaction * s_awareness + summary1 + s_age        
## extent_model6 as.factor(s_extent) ~ H2_interaction * s_awareness + summary1 + s_age + s_sex
##               link: threshold:
## extent_model5 logit flexible  
## extent_model6 logit flexible  
## 
##               no.par    AIC  logLik LR.stat df Pr(>Chisq)  
## extent_model5     19 7007.7 -3484.8                        
## extent_model6     20 7006.4 -3483.2  3.2613  1    0.07093 .
## ---
## Signif. codes:  0 '***' 0.001 '**' 0.01 '*' 0.05 '.' 0.1 ' ' 1
```

```
extent_model7 <- clm(as.factor(s_extent) ~ H2_interaction*s_awareness + 
                       summary1 + s_age + s_sex +
                       s_school, data = data2_wide_reg, 
                     link = "logit")
anova(extent_model5,extent_model7)
```

```
## Likelihood ratio tests of cumulative link models:
##  
##               formula:                                                                                
## extent_model5 as.factor(s_extent) ~ H2_interaction * s_awareness + summary1 + s_age                   
## extent_model7 as.factor(s_extent) ~ H2_interaction * s_awareness + summary1 + s_age + s_sex + s_school
##               link: threshold:
## extent_model5 logit flexible  
## extent_model7 logit flexible  
## 
##               no.par    AIC  logLik LR.stat df Pr(>Chisq)    
## extent_model5     19 7007.7 -3484.8                          
## extent_model7     22 6949.4 -3452.7  64.252  3  7.249e-14 ***
## ---
## Signif. codes:  0 '***' 0.001 '**' 0.01 '*' 0.05 '.' 0.1 ' ' 1
```

```
extent_model8 <- clm(as.factor(s_extent) ~ H2_interaction*s_awareness + 
                       summary1 + s_age + s_sex +
                       s_school + as.factor(s_interest), data = data2_wide_reg, 
                     link = "logit")
anova(extent_model7,extent_model8)
```

```
## Likelihood ratio tests of cumulative link models:
##  
##               formula:                                                                                                        
## extent_model7 as.factor(s_extent) ~ H2_interaction * s_awareness + summary1 + s_age + s_sex + s_school                        
## extent_model8 as.factor(s_extent) ~ H2_interaction * s_awareness + summary1 + s_age + s_sex + s_school + as.factor(s_interest)
##               link: threshold:
## extent_model7 logit flexible  
## extent_model8 logit flexible  
## 
##               no.par    AIC  logLik LR.stat df Pr(>Chisq)    
## extent_model7     22 6949.4 -3452.7                          
## extent_model8     26 6931.2 -3439.6  26.217  4  2.861e-05 ***
## ---
## Signif. codes:  0 '***' 0.001 '**' 0.01 '*' 0.05 '.' 0.1 ' ' 1
```

```
summary(extent_model8)
```

```
## formula: 
## as.factor(s_extent) ~ H2_interaction * s_awareness + summary1 + s_age + s_sex + s_school + as.factor(s_interest)
## data:    data2_wide_reg
## 
##  link  threshold nobs logLik   AIC     niter max.grad cond.H 
##  logit flexible  1679 -3439.60 6931.20 8(2)  5.73e-08 1.1e+06
## 
## Coefficients:
##                                                            Estimate Std. Error
## H2_interactionno disclaimer.new guideline                  0.182468   0.198635
## H2_interactiondisclaimer.new guideline                     0.036018   0.200742
## s_awarenesspass                                            0.966097   0.201849
## summary1Faerber                                           -0.023117   0.086318
## s_age                                                     -0.013140   0.002876
## s_sexmale                                                  0.119397   0.086817
## s_schoolReal                                               0.333643   0.106849
## s_schoolAbi                                                0.894282   0.110627
## as.factor(s_interest)5                                    -0.053340   0.133711
## as.factor(s_interest)6                                    -0.084149   0.134700
## as.factor(s_interest)7                                    -0.244367   0.146398
## as.factor(s_interest)8                                    -0.631426   0.142375
## H2_interactionno disclaimer.new guideline:s_awarenesspass -0.349353   0.244601
## H2_interactiondisclaimer.new guideline:s_awarenesspass     0.091748   0.246248
##                                                           z value Pr(>|z|)    
## H2_interactionno disclaimer.new guideline                   0.919  0.35830    
## H2_interactiondisclaimer.new guideline                      0.179  0.85760    
## s_awarenesspass                                             4.786 1.70e-06 ***
## summary1Faerber                                            -0.268  0.78884    
## s_age                                                      -4.568 4.92e-06 ***
## s_sexmale                                                   1.375  0.16905    
## s_schoolReal                                                3.123  0.00179 ** 
## s_schoolAbi                                                 8.084 6.28e-16 ***
## as.factor(s_interest)5                                     -0.399  0.68995    
## as.factor(s_interest)6                                     -0.625  0.53216    
## as.factor(s_interest)7                                     -1.669  0.09508 .  
## as.factor(s_interest)8                                     -4.435 9.21e-06 ***
## H2_interactionno disclaimer.new guideline:s_awarenesspass  -1.428  0.15322    
## H2_interactiondisclaimer.new guideline:s_awarenesspass      0.373  0.70946    
## ---
## Signif. codes:  0 '***' 0.001 '**' 0.01 '*' 0.05 '.' 0.1 ' ' 1
## 
## Threshold coefficients:
##       Estimate Std. Error z value
## -6|-5  -5.9015     0.5508 -10.715
## -5|-4  -5.6779     0.5034 -11.280
## -4|-3  -3.4992     0.2769 -12.636
## -3|-2  -2.7627     0.2548 -10.841
## -2|-1  -1.0233     0.2359  -4.338
## -1|0   -0.5311     0.2341  -2.268
## 0|1     0.6439     0.2349   2.742
## 1|2     1.0904     0.2363   4.615
## 2|3     1.9107     0.2397   7.971
## 3|4     2.3852     0.2429   9.818
## 4|5     3.6795     0.2631  13.984
## 5|6     4.1761     0.2793  14.953
## (34 Beobachtungen als fehlend gelöscht)
```

```
exp(coef(extent_model8))
```

```
##                                                     -6|-5 
##                                               0.002735333 
##                                                     -5|-4 
##                                               0.003420693 
##                                                     -4|-3 
##                                               0.030222339 
##                                                     -3|-2 
##                                               0.063122584 
##                                                     -2|-1 
##                                               0.359412666 
##                                                      -1|0 
##                                               0.587975715 
##                                                       0|1 
##                                               1.903977695 
##                                                       1|2 
##                                               2.975582789 
##                                                       2|3 
##                                               6.757910660 
##                                                       3|4 
##                                              10.860749671 
##                                                       4|5 
##                                              39.624729456 
##                                                       5|6 
##                                              65.110563255 
##                 H2_interactionno disclaimer.new guideline 
##                                               1.200175969 
##                    H2_interactiondisclaimer.new guideline 
##                                               1.036674672 
##                                           s_awarenesspass 
##                                               2.627668410 
##                                           summary1Faerber 
##                                               0.977148237 
##                                                     s_age 
##                                               0.986945602 
##                                                 s_sexmale 
##                                               1.126816965 
##                                              s_schoolReal 
##                                               1.396044342 
##                                               s_schoolAbi 
##                                               2.445580033 
##                                    as.factor(s_interest)5 
##                                               0.948057705 
##                                    as.factor(s_interest)6 
##                                               0.919294267 
##                                    as.factor(s_interest)7 
##                                               0.783199790 
##                                    as.factor(s_interest)8 
##                                               0.531833048 
## H2_interactionno disclaimer.new guideline:s_awarenesspass 
##                                               0.705144234 
##    H2_interactiondisclaimer.new guideline:s_awarenesspass 
##                                               1.096088417
```

```
exp(confint(extent_model8))
```

```
##                                                               2.5 %    97.5 %
## H2_interactionno disclaimer.new guideline                 0.8132562 1.7724428
## H2_interactiondisclaimer.new guideline                    0.6994857 1.5371276
## s_awarenesspass                                           1.7699782 3.9063695
## summary1Faerber                                           0.8250335 1.1572776
## s_age                                                     0.9813900 0.9925205
## s_sexmale                                                 0.9505479 1.3359513
## s_schoolReal                                              1.1324070 1.7216000
## s_schoolAbi                                               1.9696324 3.0391186
## as.factor(s_interest)5                                    0.7294092 1.2321070
## as.factor(s_interest)6                                    0.7058946 1.1970182
## as.factor(s_interest)7                                    0.5877056 1.0433855
## as.factor(s_interest)8                                    0.4021808 0.7028348
## H2_interactionno disclaimer.new guideline:s_awarenesspass 0.4363978 1.1387123
## H2_interactiondisclaimer.new guideline:s_awarenesspass    0.6762817 1.7760872
```

```
nagelkerke(fit = extent_model8, null = extent_null)
```

```
## $Models
##                                                                                                                                                      
## Model: "clm, as.factor(s_extent) ~ H2_interaction * s_awareness + summary1 + s_age + s_sex + s_school + as.factor(s_interest), data2_wide_reg, logit"
## Null:  "clm, as.factor(s_extent) ~ 1, data2_wide_reg, logit"                                                                                         
## 
## $Pseudo.R.squared.for.model.vs.null
##                              Pseudo.R.squared
## McFadden                            0.0297617
## Cox and Snell (ML)                  0.1181030
## Nagelkerke (Cragg and Uhler)        0.1198600
## 
## $Likelihood.ratio.test
##  Df.diff LogLik.diff  Chisq   p.value
##      -14     -105.51 211.02 3.061e-37
## 
## $Number.of.observations
##            
## Model: 1679
## Null:  1679
## 
## $Messages
## [1] "Note: For models fit with REML, these statistics are based on refitting with ML"
## 
## $Warnings
## [1] "None"
```

```
H2test = emmeans(extent_model8, ~ H2_interaction)
```

```
## NOTE: Results may be misleading due to involvement in interactions
```

```
pairs(H2test, adjust = "tukey")
```

```
##  contrast                                                  estimate    SE  df
##  no disclaimer.old guideline - no disclaimer.new guideline -0.00779 0.122 Inf
##  no disclaimer.old guideline - disclaimer.new guideline    -0.08189 0.123 Inf
##  no disclaimer.new guideline - disclaimer.new guideline    -0.07410 0.102 Inf
##  z.ratio p.value
##   -0.064  0.9978
##   -0.665  0.7837
##   -0.730  0.7459
## 
## Results are averaged over the levels of: s_awareness, summary1, s_sex, s_school, s_interest 
## Note: contrasts are still on the as.factor scale 
## P value adjustment: tukey method for comparing a family of 3 estimates
```

```
cld(H2test, Letters = letters)
```

```
##  H2_interaction              emmean    SE  df asymp.LCL asymp.UCL .group
##  no disclaimer.old guideline  0.599 0.131 Inf     0.343     0.856  a    
##  no disclaimer.new guideline  0.607 0.111 Inf     0.389     0.825  a    
##  disclaimer.new guideline     0.681 0.111 Inf     0.463     0.900  a    
## 
## Results are averaged over the levels of: s_awareness, summary1, s_sex, s_school, s_interest 
## Results are given on the as.factor (not the response) scale. 
## Confidence level used: 0.95 
## Note: contrasts are still on the as.factor scale 
## P value adjustment: tukey method for comparing a family of 3 estimates 
## significance level used: alpha = 0.05 
## NOTE: If two or more means share the same grouping symbol,
##       then we cannot show them to be different.
##       But we also did not show them to be the same.
```

#### Logistic Regression by Group

```
data2_wide_reg3 <- subset(data2_wide, condition == 2| condition == 6)
View(data2_wide_reg3)

extent_null_1 <- clm(as.factor(s_extent) ~ 1, data = data2_wide_reg3, link = "logit")

extent_model8_1 <- clm(as.factor(s_extent) ~ H2_interaction*s_awareness + 
                       summary1 + s_age + s_sex +
                       s_school + as.factor(s_interest), data = data2_wide_reg3, 
                     link = "logit")

summary(extent_model8_1)
```

```
## formula: 
## as.factor(s_extent) ~ H2_interaction * s_awareness + summary1 + s_age + s_sex + s_school + as.factor(s_interest)
## data:    data2_wide_reg3
## 
##  link  threshold nobs logLik   AIC     niter max.grad cond.H 
##  logit flexible  689  -1418.80 2883.60 7(0)  1.27e-12 6.0e+05
## 
## Coefficients:
##                                                         Estimate Std. Error
## H2_interactiondisclaimer.new guideline                 -0.090892   0.246319
## s_awarenesspass                                         1.000818   0.205758
## summary1Faerber                                         0.002191   0.136022
## s_age                                                  -0.016205   0.004450
## s_sexmale                                               0.217967   0.136897
## s_schoolReal                                            0.388165   0.170128
## s_schoolAbi                                             0.924417   0.171772
## as.factor(s_interest)5                                 -0.403292   0.214225
## as.factor(s_interest)6                                 -0.219034   0.214824
## as.factor(s_interest)7                                 -0.532376   0.231988
## as.factor(s_interest)8                                 -1.015751   0.221343
## H2_interactiondisclaimer.new guideline:s_awarenesspass  0.056550   0.293925
##                                                        z value Pr(>|z|)    
## H2_interactiondisclaimer.new guideline                  -0.369 0.712125    
## s_awarenesspass                                          4.864 1.15e-06 ***
## summary1Faerber                                          0.016 0.987147    
## s_age                                                   -3.642 0.000271 ***
## s_sexmale                                                1.592 0.111342    
## s_schoolReal                                             2.282 0.022513 *  
## s_schoolAbi                                              5.382 7.38e-08 ***
## as.factor(s_interest)5                                  -1.883 0.059760 .  
## as.factor(s_interest)6                                  -1.020 0.307918    
## as.factor(s_interest)7                                  -2.295 0.021742 *  
## as.factor(s_interest)8                                  -4.589 4.45e-06 ***
## H2_interactiondisclaimer.new guideline:s_awarenesspass   0.192 0.847432    
## ---
## Signif. codes:  0 '***' 0.001 '**' 0.01 '*' 0.05 '.' 0.1 ' ' 1
## 
## Threshold coefficients:
##       Estimate Std. Error z value
## -6|-4  -5.6172     0.6516  -8.620
## -4|-3  -3.8535     0.3873  -9.949
## -3|-2  -2.9814     0.3416  -8.728
## -2|-1  -1.3050     0.3084  -4.231
## -1|0   -0.7459     0.3040  -2.453
## 0|1     0.3914     0.3045   1.285
## 1|2     0.7784     0.3068   2.537
## 2|3     1.5571     0.3129   4.977
## 3|4     2.1805     0.3201   6.813
## 4|5     3.4604     0.3552   9.741
## 5|6     3.9278     0.3818  10.288
## (13 Beobachtungen als fehlend gelöscht)
```

```
exp(coef(extent_model8_1))
```

```
##                                                  -6|-4 
##                                            0.003634913 
##                                                  -4|-3 
##                                            0.021204999 
##                                                  -3|-2 
##                                            0.050720376 
##                                                  -2|-1 
##                                            0.271181252 
##                                                   -1|0 
##                                            0.474330351 
##                                                    0|1 
##                                            1.479098750 
##                                                    1|2 
##                                            2.177951598 
##                                                    2|3 
##                                            4.744824003 
##                                                    3|4 
##                                            8.850299130 
##                                                    4|5 
##                                           31.829325891 
##                                                    5|6 
##                                           50.794024402 
##                 H2_interactiondisclaimer.new guideline 
##                                            0.913115874 
##                                        s_awarenesspass 
##                                            2.720506151 
##                                        summary1Faerber 
##                                            1.002193681 
##                                                  s_age 
##                                            0.983925341 
##                                              s_sexmale 
##                                            1.243545409 
##                                           s_schoolReal 
##                                            1.474273412 
##                                            s_schoolAbi 
##                                            2.520399443 
##                                 as.factor(s_interest)5 
##                                            0.668117009 
##                                 as.factor(s_interest)6 
##                                            0.803294277 
##                                 as.factor(s_interest)7 
##                                            0.587208322 
##                                 as.factor(s_interest)8 
##                                            0.362130527 
## H2_interactiondisclaimer.new guideline:s_awarenesspass 
##                                            1.058179674
```

```
exp(confint(extent_model8_1))
```

```
##                                                            2.5 %    97.5 %
## H2_interactiondisclaimer.new guideline                 0.5629969 1.4795765
## s_awarenesspass                                        1.8197702 4.0786198
## summary1Faerber                                        0.7676102 1.3085061
## s_age                                                  0.9753589 0.9925287
## s_sexmale                                              0.9510733 1.6268243
## s_schoolReal                                           1.0566895 2.0591538
## s_schoolAbi                                            1.8018552 3.5339262
## as.factor(s_interest)5                                 0.4386213 1.0161700
## as.factor(s_interest)6                                 0.5268458 1.2234120
## as.factor(s_interest)7                                 0.3723557 0.9249113
## as.factor(s_interest)8                                 0.2342871 0.5581544
## H2_interactiondisclaimer.new guideline:s_awarenesspass 0.5947637 1.8833968
```

```
nagelkerke(fit = extent_model8_1, null = extent_null_1)
```

```
## $Models
##                                                                                                                                                       
## Model: "clm, as.factor(s_extent) ~ H2_interaction * s_awareness + summary1 + s_age + s_sex + s_school + as.factor(s_interest), data2_wide_reg3, logit"
## Null:  "clm, as.factor(s_extent) ~ 1, data2_wide_reg3, logit"                                                                                         
## 
## $Pseudo.R.squared.for.model.vs.null
##                              Pseudo.R.squared
## McFadden                            0.0359274
## Cox and Snell (ML)                  0.1422810
## Nagelkerke (Cragg and Uhler)        0.1442950
## 
## $Likelihood.ratio.test
##  Df.diff LogLik.diff  Chisq    p.value
##      -12     -52.873 105.75 4.1364e-17
## 
## $Number.of.observations
##           
## Model: 689
## Null:  689
## 
## $Messages
## [1] "Note: For models fit with REML, these statistics are based on refitting with ML"
## 
## $Warnings
## [1] "None"
```

```
H2test_1 = emmeans(extent_model8_1, ~ H2_interaction)
```

```
## NOTE: Results may be misleading due to involvement in interactions
```

```
pairs(H2test_1, adjust = "tukey")
```

```
##  contrast                                               estimate    SE  df
##  no disclaimer.old guideline - disclaimer.new guideline   0.0626 0.148 Inf
##  z.ratio p.value
##    0.424  0.6715
## 
## Results are averaged over the levels of: s_awareness, summary1, s_sex, s_school, s_interest 
## Note: contrasts are still on the as.factor scale
```

```
cld(H2test_1, Letters = letters)
```

```
##  H2_interaction               emmean    SE  df asymp.LCL asymp.UCL .group
##  disclaimer.new guideline    0.00715 0.132 Inf    -0.251     0.266  a    
##  no disclaimer.old guideline 0.06977 0.125 Inf    -0.176     0.316  a    
## 
## Results are averaged over the levels of: s_awareness, summary1, s_sex, s_school, s_interest 
## Results are given on the as.factor (not the response) scale. 
## Confidence level used: 0.95 
## Note: contrasts are still on the as.factor scale 
## significance level used: alpha = 0.05 
## NOTE: If two or more means share the same grouping symbol,
##       then we cannot show them to be different.
##       But we also did not show them to be the same.
```

```
data2_wide_reg4 <- subset(data2_wide, condition == 4| condition == 6)
View(data2_wide_reg4)

extent_null_2 <- clm(as.factor(s_extent) ~ 1, data = data2_wide_reg4, link = "logit")

extent_model8_2 <- clm(as.factor(s_extent) ~ H2_interaction*s_awareness + 
                       summary1 + s_age + s_sex +
                       s_school + as.factor(s_interest), data = data2_wide_reg4, 
                     link = "logit")

summary(extent_model8_2)
```

```
## formula: 
## as.factor(s_extent) ~ H2_interaction * s_awareness + summary1 + s_age + s_sex + s_school + as.factor(s_interest)
## data:    data2_wide_reg4
## 
##  link  threshold nobs logLik   AIC     niter max.grad cond.H 
##  logit flexible  685  -1403.90 2853.80 7(0)  7.90e-09 7.1e+05
## 
## Coefficients:
##                                                         Estimate Std. Error
## H2_interactiondisclaimer.new guideline                  0.127107   0.223031
## s_awarenesspass                                         0.938388   0.205758
## summary1Faerber                                        -0.162547   0.136466
## s_age                                                  -0.008477   0.004532
## s_sexmale                                              -0.019523   0.136157
## s_schoolReal                                            0.121688   0.171059
## s_schoolAbi                                             0.801180   0.169996
## as.factor(s_interest)5                                 -0.062240   0.208630
## as.factor(s_interest)6                                  0.081036   0.211852
## as.factor(s_interest)7                                 -0.314890   0.228084
## as.factor(s_interest)8                                 -0.876385   0.220413
## H2_interactiondisclaimer.new guideline:s_awarenesspass  0.179769   0.281711
##                                                        z value Pr(>|z|)    
## H2_interactiondisclaimer.new guideline                   0.570   0.5687    
## s_awarenesspass                                          4.561 5.10e-06 ***
## summary1Faerber                                         -1.191   0.2336    
## s_age                                                   -1.870   0.0614 .  
## s_sexmale                                               -0.143   0.8860    
## s_schoolReal                                             0.711   0.4768    
## s_schoolAbi                                              4.713 2.44e-06 ***
## as.factor(s_interest)5                                  -0.298   0.7655    
## as.factor(s_interest)6                                   0.383   0.7021    
## as.factor(s_interest)7                                  -1.381   0.1674    
## as.factor(s_interest)8                                  -3.976 7.01e-05 ***
## H2_interactiondisclaimer.new guideline:s_awarenesspass   0.638   0.5234    
## ---
## Signif. codes:  0 '***' 0.001 '**' 0.01 '*' 0.05 '.' 0.1 ' ' 1
## 
## Threshold coefficients:
##       Estimate Std. Error z value
## -6|-4  -6.4502     1.0459  -6.167
## -4|-3  -3.5783     0.3906  -9.162
## -3|-2  -2.7572     0.3471  -7.945
## -2|-1  -1.1149     0.3123  -3.570
## -1|0   -0.6131     0.3079  -1.992
## 0|1     0.6156     0.3089   1.993
## 1|2     1.0569     0.3119   3.389
## 2|3     1.8305     0.3178   5.760
## 3|4     2.3326     0.3234   7.212
## 4|5     3.5177     0.3526   9.976
## 5|6     3.9199     0.3710  10.565
## (13 Beobachtungen als fehlend gelöscht)
```

```
exp(coef(extent_model8_2))
```

```
##                                                  -6|-4 
##                                            0.001580161 
##                                                  -4|-3 
##                                            0.027922329 
##                                                  -3|-2 
##                                            0.063466214 
##                                                  -2|-1 
##                                            0.327932501 
##                                                   -1|0 
##                                            0.541656975 
##                                                    0|1 
##                                            1.850831433 
##                                                    1|2 
##                                            2.877462036 
##                                                    2|3 
##                                            6.236700238 
##                                                    3|4 
##                                           10.304665335 
##                                                    4|5 
##                                           33.707327868 
##                                                    5|6 
##                                           50.397042812 
##                 H2_interactiondisclaimer.new guideline 
##                                            1.135538128 
##                                        s_awarenesspass 
##                                            2.555859088 
##                                        summary1Faerber 
##                                            0.849976130 
##                                                  s_age 
##                                            0.991558983 
##                                              s_sexmale 
##                                            0.980666181 
##                                           s_schoolReal 
##                                            1.129401904 
##                                            s_schoolAbi 
##                                            2.228168254 
##                                 as.factor(s_interest)5 
##                                            0.939657635 
##                                 as.factor(s_interest)6 
##                                            1.084409468 
##                                 as.factor(s_interest)7 
##                                            0.729868975 
##                                 as.factor(s_interest)8 
##                                            0.416285213 
## H2_interactiondisclaimer.new guideline:s_awarenesspass 
##                                            1.196941256
```

```
exp(confint(extent_model8_2))
```

```
##                                                            2.5 %    97.5 %
## H2_interactiondisclaimer.new guideline                 0.7334265 1.7590529
## s_awarenesspass                                        1.7093048 3.8310222
## summary1Faerber                                        0.6502987 1.1104647
## s_age                                                  0.9827759 1.0003991
## s_sexmale                                              0.7509191 1.2807341
## s_schoolReal                                           0.8076441 1.5796077
## s_schoolAbi                                            1.5980104 3.1124011
## as.factor(s_interest)5                                 0.6241000 1.4144816
## as.factor(s_interest)6                                 0.7158071 1.6429696
## as.factor(s_interest)7                                 0.4665580 1.1413396
## as.factor(s_interest)8                                 0.2699372 0.6407366
## H2_interactiondisclaimer.new guideline:s_awarenesspass 0.6889916 2.0795712
```

```
nagelkerke(fit = extent_model8_2, null = extent_null_2)
```

```
## $Models
##                                                                                                                                                       
## Model: "clm, as.factor(s_extent) ~ H2_interaction * s_awareness + summary1 + s_age + s_sex + s_school + as.factor(s_interest), data2_wide_reg4, logit"
## Null:  "clm, as.factor(s_extent) ~ 1, data2_wide_reg4, logit"                                                                                         
## 
## $Pseudo.R.squared.for.model.vs.null
##                              Pseudo.R.squared
## McFadden                            0.0378383
## Cox and Snell (ML)                  0.1488760
## Nagelkerke (Cragg and Uhler)        0.1510090
## 
## $Likelihood.ratio.test
##  Df.diff LogLik.diff  Chisq    p.value
##      -12      -55.21 110.42 4.9409e-18
## 
## $Number.of.observations
##           
## Model: 685
## Null:  685
## 
## $Messages
## [1] "Note: For models fit with REML, these statistics are based on refitting with ML"
## 
## $Warnings
## [1] "None"
```

```
H2test_2 = emmeans(extent_model8_2, ~ H2_interaction)
```

```
## NOTE: Results may be misleading due to involvement in interactions
```

```
pairs(H2test_2, adjust = "tukey")
```

```
##  contrast                                               estimate   SE  df
##  no disclaimer.old guideline - disclaimer.new guideline   -0.217 0.14 Inf
##  z.ratio p.value
##   -1.546  0.1222
## 
## Results are averaged over the levels of: s_awareness, summary1, s_sex, s_school, s_interest 
## Note: contrasts are still on the as.factor scale
```

```
cld(H2test_2, Letters = letters)
```

```
##  H2_interaction              emmean    SE  df asymp.LCL asymp.UCL .group
##  no disclaimer.old guideline  0.176 0.145 Inf    -0.108     0.460  a    
##  disclaimer.new guideline     0.393 0.144 Inf     0.111     0.674  a    
## 
## Results are averaged over the levels of: s_awareness, summary1, s_sex, s_school, s_interest 
## Results are given on the as.factor (not the response) scale. 
## Confidence level used: 0.95 
## Note: contrasts are still on the as.factor scale 
## significance level used: alpha = 0.05 
## NOTE: If two or more means share the same grouping symbol,
##       then we cannot show them to be different.
##       But we also did not show them to be the same.
```

## H3

### H3a

```
H3a <- subset(data2_wide, condition == 2|condition == 4|condition == 6)
View(H3a)

describeBy(H3a$s_diff,H3a$disclaimer)
```

```
## 
##  Descriptive statistics by group 
## group: no disclaimer
##    vars   n mean   sd median trimmed  mad min max range skew kurtosis  se
## X1    1 345 0.37 1.93      0    0.37 1.48  -6   6    12 -0.1     0.46 0.1
## ------------------------------------------------------------ 
## group: disclaimer
##    vars   n mean   sd median trimmed  mad min max range skew kurtosis   se
## X1    1 660 0.27 1.94      0    0.18 1.48  -6   6    12 0.39     0.61 0.08
```

```
wilcox.test(s_diff~disclaimer, data = H3a, exact = FALSE,
            conf.int = TRUE)
```

```
## 
##  Wilcoxon rank sum test with continuity correction
## 
## data:  s_diff by disclaimer
## W = 120378, p-value = 0.1249
## alternative hypothesis: true location shift is not equal to 0
## 95 percent confidence interval:
##  -7.590539e-05  2.125303e-05
## sample estimates:
## difference in location 
##           5.661916e-06
```

#### H3a post hoc

```
H3a_1 <- subset(data2_wide, condition == 2|condition == 6)
View(H3a_1)
describeBy(H3a_1$s_diff,H3a_1$disclaimer)
```

```
## 
##  Descriptive statistics by group 
## group: no disclaimer
##    vars   n mean   sd median trimmed  mad min max range skew kurtosis  se
## X1    1 345 0.37 1.93      0    0.37 1.48  -6   6    12 -0.1     0.46 0.1
## ------------------------------------------------------------ 
## group: disclaimer
##    vars   n mean   sd median trimmed  mad min max range skew kurtosis   se
## X1    1 330 0.12 1.94      0    0.03 1.48  -4   6    10 0.34     0.13 0.11
```

```
wilcox.test(s_diff~disclaimer, data = H3a_1, exact = FALSE,
            conf.int = TRUE)
```

```
## 
##  Wilcoxon rank sum test with continuity correction
## 
## data:  s_diff by disclaimer
## W = 62522, p-value = 0.02371
## alternative hypothesis: true location shift is not equal to 0
## 95 percent confidence interval:
##  0.0000133882 0.9999363469
## sample estimates:
## difference in location 
##            6.73205e-06
```

```
H3a_2 <- subset(data2_wide, condition == 4|condition == 6)
View(H3a_2)
describeBy(H3a_2$s_diff,H3a_2$disclaimer)
```

```
## 
##  Descriptive statistics by group 
## group: no disclaimer
##    vars   n mean   sd median trimmed  mad min max range skew kurtosis  se
## X1    1 345 0.37 1.93      0    0.37 1.48  -6   6    12 -0.1     0.46 0.1
## ------------------------------------------------------------ 
## group: disclaimer
##    vars   n mean   sd median trimmed  mad min max range skew kurtosis   se
## X1    1 330 0.42 1.94      0    0.32 1.48  -6   6    12 0.44     1.04 0.11
```

```
wilcox.test(s_diff~disclaimer, data = H3a_2, exact = FALSE,
            conf.int = TRUE)
```

```
## 
##  Wilcoxon rank sum test with continuity correction
## 
## data:  s_diff by disclaimer
## W = 57857, p-value = 0.7051
## alternative hypothesis: true location shift is not equal to 0
## 95 percent confidence interval:
##  -4.302679e-05  3.720242e-05
## sample estimates:
## difference in location 
##           2.559738e-05
```

### H3b

```
H3b <- subset(data2_wide, condition == 1| condition == 2| condition == 3|
                condition == 4)
View(H3b)

describeBy(H3b$s_diff,H3b$disclaimer)
```

```
## 
##  Descriptive statistics by group 
## group: no disclaimer
##    vars   n mean   sd median trimmed  mad min max range skew kurtosis   se
## X1    1 652 0.27 2.02      0    0.22 1.48  -6   6    12 0.12     0.33 0.08
## ------------------------------------------------------------ 
## group: disclaimer
##    vars   n mean   sd median trimmed  mad min max range skew kurtosis   se
## X1    1 660 0.27 1.94      0    0.18 1.48  -6   6    12 0.39     0.61 0.08
```

```
wilcox.test(s_diff~disclaimer, data = H3b, exact = FALSE,
            conf.int = TRUE)
```

```
## 
##  Wilcoxon rank sum test with continuity correction
## 
## data:  s_diff by disclaimer
## W = 217553, p-value = 0.72
## alternative hypothesis: true location shift is not equal to 0
## 95 percent confidence interval:
##  -7.205325e-06  3.457267e-05
## sample estimates:
## difference in location 
##           3.252272e-05
```

#### H3b post hoc

```
H3b_1 <- subset(data2_wide, condition == 1| condition == 2)
View(H3b_1)
describeBy(H3b_1$s_diff,H3b_1$disclaimer)
```

```
## 
##  Descriptive statistics by group 
## group: no disclaimer
##    vars   n mean   sd median trimmed  mad min max range skew kurtosis   se
## X1    1 321 0.19 2.04      0    0.17 1.48  -6   6    12 0.02     0.16 0.11
## ------------------------------------------------------------ 
## group: disclaimer
##    vars   n mean   sd median trimmed  mad min max range skew kurtosis   se
## X1    1 330 0.12 1.94      0    0.03 1.48  -4   6    10 0.34     0.13 0.11
```

```
wilcox.test(s_diff~disclaimer, data = H3b_1, exact = FALSE,
            conf.int = TRUE)
```

```
## 
##  Wilcoxon rank sum test with continuity correction
## 
## data:  s_diff by disclaimer
## W = 54841, p-value = 0.4232
## alternative hypothesis: true location shift is not equal to 0
## 95 percent confidence interval:
##  -1.356082e-05  2.575015e-05
## sample estimates:
## difference in location 
##           3.936834e-05
```

```
H3b_2 <- subset(data2_wide, condition == 3| condition == 4)
View(H3b_2)
describeBy(H3b_2$s_diff,H3b_2$disclaimer)
```

```
## 
##  Descriptive statistics by group 
## group: no disclaimer
##    vars   n mean   sd median trimmed  mad min max range skew kurtosis   se
## X1    1 331 0.35 2.01      0    0.27 1.48  -6   6    12 0.21     0.47 0.11
## ------------------------------------------------------------ 
## group: disclaimer
##    vars   n mean   sd median trimmed  mad min max range skew kurtosis   se
## X1    1 330 0.42 1.94      0    0.32 1.48  -6   6    12 0.44     1.04 0.11
```

```
wilcox.test(s_diff~disclaimer, data = H3b_2, exact = FALSE,
            conf.int = TRUE)
```

```
## 
##  Wilcoxon rank sum test with continuity correction
## 
## data:  s_diff by disclaimer
## W = 53775, p-value = 0.7238
## alternative hypothesis: true location shift is not equal to 0
## 95 percent confidence interval:
##  -3.910076e-05  3.441754e-05
## sample estimates:
## difference in location 
##          -5.465709e-05
```

```
H3b_3 <- subset(data2_wide, condition == 2| condition == 3)
View(H3b_3)
describeBy(H3b_3$s_diff,H3b_3$disclaimer)
```

```
## 
##  Descriptive statistics by group 
## group: no disclaimer
##    vars   n mean   sd median trimmed  mad min max range skew kurtosis   se
## X1    1 331 0.35 2.01      0    0.27 1.48  -6   6    12 0.21     0.47 0.11
## ------------------------------------------------------------ 
## group: disclaimer
##    vars   n mean   sd median trimmed  mad min max range skew kurtosis   se
## X1    1 330 0.12 1.94      0    0.03 1.48  -4   6    10 0.34     0.13 0.11
```

```
wilcox.test(s_diff~disclaimer, data = H3b_3, exact = FALSE,
            conf.int = TRUE)
```

```
## 
##  Wilcoxon rank sum test with continuity correction
## 
## data:  s_diff by disclaimer
## W = 58499, p-value = 0.1045
## alternative hypothesis: true location shift is not equal to 0
## 95 percent confidence interval:
##  -1.456446e-05  6.645169e-05
## sample estimates:
## difference in location 
##           4.662093e-05
```

```
H3b_4 <- subset(data2_wide, condition == 1| condition == 4)
View(H3b_4)
describeBy(H3b_4$s_diff,H3b_4$disclaimer)
```

```
## 
##  Descriptive statistics by group 
## group: no disclaimer
##    vars   n mean   sd median trimmed  mad min max range skew kurtosis   se
## X1    1 321 0.19 2.04      0    0.17 1.48  -6   6    12 0.02     0.16 0.11
## ------------------------------------------------------------ 
## group: disclaimer
##    vars   n mean   sd median trimmed  mad min max range skew kurtosis   se
## X1    1 330 0.42 1.94      0    0.32 1.48  -6   6    12 0.44     1.04 0.11
```

```
wilcox.test(s_diff~disclaimer, data = H3b_4, exact = FALSE,
            conf.int = TRUE)
```

```
## 
##  Wilcoxon rank sum test with continuity correction
## 
## data:  s_diff by disclaimer
## W = 50439, p-value = 0.2779
## alternative hypothesis: true location shift is not equal to 0
## 95 percent confidence interval:
##  -1.565384e-05  5.297947e-05
## sample estimates:
## difference in location 
##           -1.49841e-07
```

### H3 Logistic Regression

```
data2_wide$H3_interaction <- data2_wide$H2_interaction
table(data2_wide$H3_interaction)
```

```
## 
## no disclaimer.old guideline no disclaimer.new guideline 
##                         357                         670 
##    disclaimer.new guideline 
##                        1013
```

```
data2_wide_reg <- subset(data2_wide, condition != 5)
View(data2_wide_reg)

diff_null <- clm(as.factor(s_diff) ~ 1, data = data2_wide_reg, link = "logit")

diff_model1 <- clm(as.factor(s_diff) ~ H3_interaction, data = data2_wide_reg,
                     link = "logit")
anova(diff_null,diff_model1)
```

```
## Likelihood ratio tests of cumulative link models:
##  
##             formula:                           link: threshold:
## diff_null   as.factor(s_diff) ~ 1              logit flexible  
## diff_model1 as.factor(s_diff) ~ H3_interaction logit flexible  
## 
##             no.par    AIC  logLik LR.stat df Pr(>Chisq)
## diff_null       12 6444.3 -3210.2                      
## diff_model1     14 6445.9 -3209.0   2.381  2     0.3041
```

```
diff_model2 <- clm(as.factor(s_diff) ~ H3_interaction + s_awareness,
                     data = data2_wide_reg, link = "logit")
anova(diff_null,diff_model2)
```

```
## Likelihood ratio tests of cumulative link models:
##  
##             formula:                                         link: threshold:
## diff_null   as.factor(s_diff) ~ 1                            logit flexible  
## diff_model2 as.factor(s_diff) ~ H3_interaction + s_awareness logit flexible  
## 
##             no.par    AIC  logLik LR.stat df Pr(>Chisq)    
## diff_null       12 6444.3 -3210.2                          
## diff_model2     15 6427.8 -3198.9  22.545  3  5.023e-05 ***
## ---
## Signif. codes:  0 '***' 0.001 '**' 0.01 '*' 0.05 '.' 0.1 ' ' 1
```

```
diff_model3 <- clm(as.factor(s_diff) ~ H3_interaction*s_awareness,
                     data = data2_wide_reg, link = "logit")
anova(diff_model2,diff_model3)
```

```
## Likelihood ratio tests of cumulative link models:
##  
##             formula:                                         link: threshold:
## diff_model2 as.factor(s_diff) ~ H3_interaction + s_awareness logit flexible  
## diff_model3 as.factor(s_diff) ~ H3_interaction * s_awareness logit flexible  
## 
##             no.par    AIC  logLik LR.stat df Pr(>Chisq)
## diff_model2     15 6427.8 -3198.9                      
## diff_model3     17 6430.8 -3198.4  0.9476  2     0.6226
```

```
diff_model4 <- clm(as.factor(s_diff) ~ H3_interaction*s_awareness + text_order, data = data2_wide_reg, link = "logit")
anova(diff_model2,diff_model4)
```

```
## Likelihood ratio tests of cumulative link models:
##  
##             formula:                                                      link:
## diff_model2 as.factor(s_diff) ~ H3_interaction + s_awareness              logit
## diff_model4 as.factor(s_diff) ~ H3_interaction * s_awareness + text_order logit
##             threshold:
## diff_model2 flexible  
## diff_model4 flexible  
## 
##             no.par    AIC  logLik LR.stat df Pr(>Chisq)    
## diff_model2     15 6427.8 -3198.9                          
## diff_model4     18 6411.5 -3187.7  22.271  3   5.73e-05 ***
## ---
## Signif. codes:  0 '***' 0.001 '**' 0.01 '*' 0.05 '.' 0.1 ' ' 1
```

```
diff_model5 <- clm(as.factor(s_diff) ~ H3_interaction*s_awareness + text_order + s_age, data = data2_wide_reg, 
                     link = "logit")
anova(diff_model4,diff_model5)
```

```
## Likelihood ratio tests of cumulative link models:
##  
##             formula:                                                             
## diff_model4 as.factor(s_diff) ~ H3_interaction * s_awareness + text_order        
## diff_model5 as.factor(s_diff) ~ H3_interaction * s_awareness + text_order + s_age
##             link: threshold:
## diff_model4 logit flexible  
## diff_model5 logit flexible  
## 
##             no.par    AIC  logLik LR.stat df Pr(>Chisq)  
## diff_model4     18 6411.5 -3187.7                        
## diff_model5     19 6407.8 -3184.9  5.7455  1    0.01653 *
## ---
## Signif. codes:  0 '***' 0.001 '**' 0.01 '*' 0.05 '.' 0.1 ' ' 1
```

```
diff_model6 <- clm(as.factor(s_diff) ~ H3_interaction*s_awareness + text_order + s_age + s_sex, 
                   data = data2_wide_reg, 
                     link = "logit")
anova(diff_model5,diff_model6)
```

```
## Likelihood ratio tests of cumulative link models:
##  
##             formula:                                                                     
## diff_model5 as.factor(s_diff) ~ H3_interaction * s_awareness + text_order + s_age        
## diff_model6 as.factor(s_diff) ~ H3_interaction * s_awareness + text_order + s_age + s_sex
##             link: threshold:
## diff_model5 logit flexible  
## diff_model6 logit flexible  
## 
##             no.par    AIC  logLik LR.stat df Pr(>Chisq)
## diff_model5     19 6407.8 -3184.9                      
## diff_model6     20 6407.1 -3183.6  2.6343  1     0.1046
```

```
diff_model7 <- clm(as.factor(s_diff) ~ H3_interaction*s_awareness + text_order + s_age + s_sex +
                       s_school, data = data2_wide_reg, 
                     link = "logit")
anova(diff_model5,diff_model7)
```

```
## Likelihood ratio tests of cumulative link models:
##  
##             formula:                                                                                
## diff_model5 as.factor(s_diff) ~ H3_interaction * s_awareness + text_order + s_age                   
## diff_model7 as.factor(s_diff) ~ H3_interaction * s_awareness + text_order + s_age + s_sex + s_school
##             link: threshold:
## diff_model5 logit flexible  
## diff_model7 logit flexible  
## 
##             no.par    AIC  logLik LR.stat df Pr(>Chisq)
## diff_model5     19 6407.8 -3184.9                      
## diff_model7     22 6409.2 -3182.6  4.5365  3     0.2091
```

```
diff_model8 <- clm(as.factor(s_diff) ~ H3_interaction*s_awareness  + text_order + s_age + s_sex +
                       s_school + as.factor(s_interest), data = data2_wide_reg, 
                     link = "logit")
anova(diff_model5,diff_model8)
```

```
## Likelihood ratio tests of cumulative link models:
##  
##             formula:                                                                                                        
## diff_model5 as.factor(s_diff) ~ H3_interaction * s_awareness + text_order + s_age                                           
## diff_model8 as.factor(s_diff) ~ H3_interaction * s_awareness + text_order + s_age + s_sex + s_school + as.factor(s_interest)
##             link: threshold:
## diff_model5 logit flexible  
## diff_model8 logit flexible  
## 
##             no.par    AIC  logLik LR.stat df Pr(>Chisq)
## diff_model5     19 6407.8 -3184.9                      
## diff_model8     26 6413.1 -3180.6  8.6496  7     0.2788
```

```
summary(diff_model8)
```

```
## formula: 
## as.factor(s_diff) ~ H3_interaction * s_awareness + text_order + s_age + s_sex + s_school + as.factor(s_interest)
## data:    data2_wide_reg
## 
##  link  threshold nobs logLik   AIC     niter max.grad cond.H 
##  logit flexible  1657 -3180.55 6413.10 7(0)  1.10e-09 1.0e+06
## 
## Coefficients:
##                                                            Estimate Std. Error
## H3_interactionno disclaimer.new guideline                 -0.004203   0.205288
## H3_interactiondisclaimer.new guideline                    -0.095444   0.203037
## s_awarenesspass                                            0.540993   0.205231
## text_orderFaerber                                          0.410684   0.088321
## s_age                                                     -0.006246   0.002890
## s_sexmale                                                 -0.143024   0.088332
## s_schoolReal                                               0.026193   0.108341
## s_schoolAbi                                                0.134485   0.110133
## as.factor(s_interest)5                                     0.175235   0.135269
## as.factor(s_interest)6                                     0.100804   0.137713
## as.factor(s_interest)7                                     0.120144   0.148907
## as.factor(s_interest)8                                    -0.074686   0.142485
## H3_interactionno disclaimer.new guideline:s_awarenesspass -0.207895   0.251160
## H3_interactiondisclaimer.new guideline:s_awarenesspass    -0.120352   0.248928
##                                                           z value Pr(>|z|)    
## H3_interactionno disclaimer.new guideline                  -0.020  0.98367    
## H3_interactiondisclaimer.new guideline                     -0.470  0.63830    
## s_awarenesspass                                             2.636  0.00839 ** 
## text_orderFaerber                                           4.650 3.32e-06 ***
## s_age                                                      -2.162  0.03066 *  
## s_sexmale                                                  -1.619  0.10541    
## s_schoolReal                                                0.242  0.80897    
## s_schoolAbi                                                 1.221  0.22205    
## as.factor(s_interest)5                                      1.295  0.19516    
## as.factor(s_interest)6                                      0.732  0.46418    
## as.factor(s_interest)7                                      0.807  0.41976    
## as.factor(s_interest)8                                     -0.524  0.60016    
## H3_interactionno disclaimer.new guideline:s_awarenesspass  -0.828  0.40782    
## H3_interactiondisclaimer.new guideline:s_awarenesspass     -0.483  0.62875    
## ---
## Signif. codes:  0 '***' 0.001 '**' 0.01 '*' 0.05 '.' 0.1 ' ' 1
## 
## Threshold coefficients:
##       Estimate Std. Error z value
## -6|-5  -5.8680     0.5520 -10.630
## -5|-4  -5.3066     0.4445 -11.939
## -4|-3  -3.1082     0.2669 -11.648
## -3|-2  -2.7127     0.2571 -10.550
## -2|-1  -1.2796     0.2412  -5.306
## -1|0   -0.8168     0.2393  -3.413
## 0|1     0.7005     0.2387   2.935
## 1|2     1.2036     0.2399   5.017
## 2|3     2.4419     0.2482   9.840
## 3|4     2.7840     0.2526  11.020
## 4|5     4.1303     0.2919  14.152
## 5|6     4.6722     0.3253  14.364
## (56 Beobachtungen als fehlend gelöscht)
```

```
exp(coef(diff_model8))
```

```
##                                                     -6|-5 
##                                              2.828484e-03 
##                                                     -5|-4 
##                                              4.958672e-03 
##                                                     -4|-3 
##                                              4.468047e-02 
##                                                     -3|-2 
##                                              6.635460e-02 
##                                                     -2|-1 
##                                              2.781520e-01 
##                                                      -1|0 
##                                              4.418229e-01 
##                                                       0|1 
##                                              2.014721e+00 
##                                                       1|2 
##                                              3.332141e+00 
##                                                       2|3 
##                                              1.149435e+01 
##                                                       3|4 
##                                              1.618335e+01 
##                                                       4|5 
##                                              6.219571e+01 
##                                                       5|6 
##                                              1.069331e+02 
##                 H3_interactionno disclaimer.new guideline 
##                                              9.958063e-01 
##                    H3_interactiondisclaimer.new guideline 
##                                              9.089693e-01 
##                                           s_awarenesspass 
##                                              1.717712e+00 
##                                         text_orderFaerber 
##                                              1.507849e+00 
##                                                     s_age 
##                                              9.937735e-01 
##                                                 s_sexmale 
##                                              8.667330e-01 
##                                              s_schoolReal 
##                                              1.026539e+00 
##                                               s_schoolAbi 
##                                              1.143947e+00 
##                                    as.factor(s_interest)5 
##                                              1.191526e+00 
##                                    as.factor(s_interest)6 
##                                              1.106059e+00 
##                                    as.factor(s_interest)7 
##                                              1.127659e+00 
##                                    as.factor(s_interest)8 
##                                              9.280352e-01 
## H3_interactionno disclaimer.new guideline:s_awarenesspass 
##                                              8.122921e-01 
##    H3_interactiondisclaimer.new guideline:s_awarenesspass 
##                                              8.866080e-01
```

```
exp(confint((diff_model8)))
```

```
##                                                               2.5 %    97.5 %
## H3_interactionno disclaimer.new guideline                 0.6660817 1.4899877
## H3_interactiondisclaimer.new guideline                    0.6106643 1.3540269
## s_awarenesspass                                           1.1494258 2.5706104
## text_orderFaerber                                         1.2684280 1.7932661
## s_age                                                     0.9881554 0.9994142
## s_sexmale                                                 0.7288709 1.0305012
## s_schoolReal                                              0.8301181 1.2694337
## s_schoolAbi                                               0.9219047 1.4197396
## as.factor(s_interest)5                                    0.9140985 1.5535360
## as.factor(s_interest)6                                    0.8444120 1.4489208
## as.factor(s_interest)7                                    0.8422136 1.5100043
## as.factor(s_interest)8                                    0.7017732 1.2269163
## H3_interactionno disclaimer.new guideline:s_awarenesspass 0.4962519 1.3286122
## H3_interactiondisclaimer.new guideline:s_awarenesspass    0.5440616 1.4439228
```

```
nagelkerke(fit = diff_model8, null = diff_null)
```

```
## $Models
##                                                                                                                                                      
## Model: "clm, as.factor(s_diff) ~ H3_interaction * s_awareness + text_order + s_age + s_sex + s_school + as.factor(s_interest), data2_wide_reg, logit"
## Null:  "clm, as.factor(s_diff) ~ 1, data2_wide_reg, logit"                                                                                           
## 
## $Pseudo.R.squared.for.model.vs.null
##                              Pseudo.R.squared
## McFadden                           0.00922245
## Cox and Snell (ML)                 0.03510290
## Nagelkerke (Cragg and Uhler)       0.03584720
## 
## $Likelihood.ratio.test
##  Df.diff LogLik.diff  Chisq    p.value
##      -14     -29.605 59.211 1.6126e-07
## 
## $Number.of.observations
##            
## Model: 1657
## Null:  1657
## 
## $Messages
## [1] "Note: For models fit with REML, these statistics are based on refitting with ML"
## 
## $Warnings
## [1] "None"
```

```
H3test = emmeans(diff_model8, ~ H3_interaction)
```

```
## NOTE: Results may be misleading due to involvement in interactions
```

```
pairs(H3test, adjust = "tukey")
```

```
##  contrast                                                  estimate    SE  df
##  no disclaimer.old guideline - no disclaimer.new guideline   0.1082 0.126 Inf
##  no disclaimer.old guideline - disclaimer.new guideline      0.1556 0.125 Inf
##  no disclaimer.new guideline - disclaimer.new guideline      0.0475 0.103 Inf
##  z.ratio p.value
##    0.861  0.6649
##    1.248  0.4251
##    0.459  0.8904
## 
## Results are averaged over the levels of: s_awareness, text_order, s_sex, s_school, s_interest 
## Note: contrasts are still on the as.factor scale 
## P value adjustment: tukey method for comparing a family of 3 estimates
```

```
cld(H3test, Letters = letters)
```

```
##  H3_interaction              emmean    SE  df asymp.LCL asymp.UCL .group
##  disclaimer.new guideline     0.347 0.109 Inf     0.134     0.560  a    
##  no disclaimer.new guideline  0.394 0.110 Inf     0.179     0.610  a    
##  no disclaimer.old guideline  0.502 0.130 Inf     0.247     0.758  a    
## 
## Results are averaged over the levels of: s_awareness, text_order, s_sex, s_school, s_interest 
## Results are given on the as.factor (not the response) scale. 
## Confidence level used: 0.95 
## Note: contrasts are still on the as.factor scale 
## P value adjustment: tukey method for comparing a family of 3 estimates 
## significance level used: alpha = 0.05 
## NOTE: If two or more means share the same grouping symbol,
##       then we cannot show them to be different.
##       But we also did not show them to be the same.
```

#### Logistic Regression by Group

```
data2_wide_reg5 <- subset(data2_wide, condition == 2 | condition == 6)
View(data2_wide_reg5)

diff_null_1 <- clm(as.factor(s_diff) ~ 1, data = data2_wide_reg5, link = "logit")

diff_model8_1 <- clm(as.factor(s_diff) ~ H3_interaction*s_awareness  + text_order + s_age + s_sex +
                       s_school + as.factor(s_interest), data = data2_wide_reg5, 
                     link = "logit")

summary(diff_model8_1)
```

```
## formula: 
## as.factor(s_diff) ~ H3_interaction * s_awareness + text_order + s_age + s_sex + s_school + as.factor(s_interest)
## data:    data2_wide_reg5
## 
##  link  threshold nobs logLik   AIC     niter max.grad cond.H 
##  logit flexible  675  -1297.88 2643.77 7(0)  6.50e-08 9.0e+05
## 
## Coefficients:
##                                                         Estimate Std. Error
## H3_interactiondisclaimer.new guideline                 -0.162461   0.248468
## s_awarenesspass                                         0.511903   0.208378
## text_orderFaerber                                       0.427983   0.139018
## s_age                                                  -0.004515   0.004535
## s_sexmale                                              -0.178657   0.139614
## s_schoolReal                                            0.237719   0.171886
## s_schoolAbi                                             0.469633   0.173103
## as.factor(s_interest)5                                  0.097727   0.212803
## as.factor(s_interest)6                                 -0.265737   0.219007
## as.factor(s_interest)7                                  0.126662   0.233023
## as.factor(s_interest)8                                 -0.145705   0.222113
## H3_interactiondisclaimer.new guideline:s_awarenesspass -0.250421   0.297342
##                                                        z value Pr(>|z|)   
## H3_interactiondisclaimer.new guideline                  -0.654  0.51321   
## s_awarenesspass                                          2.457  0.01403 * 
## text_orderFaerber                                        3.079  0.00208 **
## s_age                                                   -0.996  0.31941   
## s_sexmale                                               -1.280  0.20067   
## s_schoolReal                                             1.383  0.16666   
## s_schoolAbi                                              2.713  0.00667 **
## as.factor(s_interest)5                                   0.459  0.64606   
## as.factor(s_interest)6                                  -1.213  0.22499   
## as.factor(s_interest)7                                   0.544  0.58674   
## as.factor(s_interest)8                                  -0.656  0.51183   
## H3_interactiondisclaimer.new guideline:s_awarenesspass  -0.842  0.39968   
## ---
## Signif. codes:  0 '***' 0.001 '**' 0.01 '*' 0.05 '.' 0.1 ' ' 1
## 
## Threshold coefficients:
##       Estimate Std. Error z value
## -6|-5  -6.2882     1.0444  -6.021
## -5|-4  -5.5936     0.7686  -7.278
## -4|-3  -3.0738     0.3640  -8.445
## -3|-2  -2.5910     0.3423  -7.568
## -2|-1  -1.1443     0.3136  -3.648
## -1|0   -0.6486     0.3103  -2.090
## 0|1     0.7799     0.3108   2.509
## 1|2     1.3353     0.3141   4.251
## 2|3     2.6480     0.3318   7.982
## 3|4     3.0441     0.3420   8.900
## 4|5     4.5942     0.4407  10.424
## 5|6     5.2972     0.5425   9.765
## (27 Beobachtungen als fehlend gelöscht)
```

```
exp(coef(diff_model8_1))
```

```
##                                                  -6|-5 
##                                           1.858041e-03 
##                                                  -5|-4 
##                                           3.721608e-03 
##                                                  -4|-3 
##                                           4.624488e-02 
##                                                  -3|-2 
##                                           7.494329e-02 
##                                                  -2|-1 
##                                           3.184457e-01 
##                                                   -1|0 
##                                           5.227530e-01 
##                                                    0|1 
##                                           2.181147e+00 
##                                                    1|2 
##                                           3.801060e+00 
##                                                    2|3 
##                                           1.412593e+01 
##                                                    3|4 
##                                           2.099076e+01 
##                                                    4|5 
##                                           9.891049e+01 
##                                                    5|6 
##                                           1.997747e+02 
##                 H3_interactiondisclaimer.new guideline 
##                                           8.500493e-01 
##                                        s_awarenesspass 
##                                           1.668464e+00 
##                                      text_orderFaerber 
##                                           1.534160e+00 
##                                                  s_age 
##                                           9.954951e-01 
##                                              s_sexmale 
##                                           8.363931e-01 
##                                           s_schoolReal 
##                                           1.268353e+00 
##                                            s_schoolAbi 
##                                           1.599407e+00 
##                                 as.factor(s_interest)5 
##                                           1.102662e+00 
##                                 as.factor(s_interest)6 
##                                           7.666406e-01 
##                                 as.factor(s_interest)7 
##                                           1.135033e+00 
##                                 as.factor(s_interest)8 
##                                           8.644129e-01 
## H3_interactiondisclaimer.new guideline:s_awarenesspass 
##                                           7.784728e-01
```

```
exp(confint((diff_model8_1)))
```

```
##                                                            2.5 %   97.5 %
## H3_interactiondisclaimer.new guideline                 0.5222945 1.384156
## s_awarenesspass                                        1.1098621 2.513249
## text_orderFaerber                                      1.1688067 2.015979
## s_age                                                  0.9866743 1.004379
## s_sexmale                                              0.6359648 1.099494
## s_schoolReal                                           0.9056480 1.777042
## s_schoolAbi                                            1.1398880 2.247363
## as.factor(s_interest)5                                 0.7266725 1.674175
## as.factor(s_interest)6                                 0.4988425 1.177568
## as.factor(s_interest)7                                 0.7188252 1.792827
## as.factor(s_interest)8                                 0.5588914 1.335544
## H3_interactiondisclaimer.new guideline:s_awarenesspass 0.4342797 1.393749
```

```
nagelkerke(fit = diff_model8_1, null = diff_null_1)
```

```
## $Models
##                                                                                                                                                       
## Model: "clm, as.factor(s_diff) ~ H3_interaction * s_awareness + text_order + s_age + s_sex + s_school + as.factor(s_interest), data2_wide_reg5, logit"
## Null:  "clm, as.factor(s_diff) ~ 1, data2_wide_reg5, logit"                                                                                           
## 
## $Pseudo.R.squared.for.model.vs.null
##                              Pseudo.R.squared
## McFadden                            0.0137674
## Cox and Snell (ML)                  0.0522672
## Nagelkerke (Cragg and Uhler)        0.0533478
## 
## $Likelihood.ratio.test
##  Df.diff LogLik.diff  Chisq    p.value
##      -12     -18.118 36.236 0.00029689
## 
## $Number.of.observations
##           
## Model: 675
## Null:  675
## 
## $Messages
## [1] "Note: For models fit with REML, these statistics are based on refitting with ML"
## 
## $Warnings
## [1] "None"
```

```
H3test_1 = emmeans(diff_model8_1, ~ H3_interaction)
```

```
## NOTE: Results may be misleading due to involvement in interactions
```

```
pairs(H3test_1, adjust = "tukey")
```

```
##  contrast                                               estimate   SE  df
##  no disclaimer.old guideline - disclaimer.new guideline    0.288 0.15 Inf
##  z.ratio p.value
##    1.914  0.0556
## 
## Results are averaged over the levels of: s_awareness, text_order, s_sex, s_school, s_interest 
## Note: contrasts are still on the as.factor scale
```

```
cld(H3test_1, Letters = letters)
```

```
##  H3_interaction              emmean    SE  df asymp.LCL asymp.UCL .group
##  disclaimer.new guideline     0.221 0.187 Inf    -0.146     0.589  a    
##  no disclaimer.old guideline  0.509 0.184 Inf     0.148     0.870  a    
## 
## Results are averaged over the levels of: s_awareness, text_order, s_sex, s_school, s_interest 
## Results are given on the as.factor (not the response) scale. 
## Confidence level used: 0.95 
## Note: contrasts are still on the as.factor scale 
## significance level used: alpha = 0.05 
## NOTE: If two or more means share the same grouping symbol,
##       then we cannot show them to be different.
##       But we also did not show them to be the same.
```

```
data2_wide_reg6 <- subset(data2_wide, condition == 4 | condition == 6)
View(data2_wide_reg6)

diff_null_2 <- clm(as.factor(s_diff) ~ 1, data = data2_wide_reg6, link = "logit")

diff_model8_2 <- clm(as.factor(s_diff) ~ H3_interaction*s_awareness  + text_order + s_age + s_sex +
                       s_school + as.factor(s_interest), data = data2_wide_reg6, 
                     link = "logit")

summary(diff_model8_2)
```

```
## formula: 
## as.factor(s_diff) ~ H3_interaction * s_awareness + text_order + s_age + s_sex + s_school + as.factor(s_interest)
## data:    data2_wide_reg6
## 
##  link  threshold nobs logLik   AIC     niter max.grad cond.H 
##  logit flexible  675  -1265.62 2579.23 7(0)  5.63e-12 7.2e+05
## 
## Coefficients:
##                                                         Estimate Std. Error
## H3_interactiondisclaimer.new guideline                 -0.018587   0.227338
## s_awarenesspass                                         0.587635   0.211885
## text_orderFaerber                                       0.448846   0.139860
## s_age                                                  -0.003001   0.004543
## s_sexmale                                              -0.092249   0.139324
## s_schoolReal                                            0.154994   0.176163
## s_schoolAbi                                             0.271576   0.171963
## as.factor(s_interest)5                                  0.136431   0.213510
## as.factor(s_interest)6                                 -0.153997   0.221819
## as.factor(s_interest)7                                 -0.263025   0.234743
## as.factor(s_interest)8                                 -0.404837   0.220637
## H3_interactiondisclaimer.new guideline:s_awarenesspass  0.065429   0.288117
##                                                        z value Pr(>|z|)   
## H3_interactiondisclaimer.new guideline                  -0.082  0.93484   
## s_awarenesspass                                          2.773  0.00555 **
## text_orderFaerber                                        3.209  0.00133 **
## s_age                                                   -0.661  0.50888   
## s_sexmale                                               -0.662  0.50789   
## s_schoolReal                                             0.880  0.37895   
## s_schoolAbi                                              1.579  0.11427   
## as.factor(s_interest)5                                   0.639  0.52283   
## as.factor(s_interest)6                                  -0.694  0.48753   
## as.factor(s_interest)7                                  -1.120  0.26251   
## as.factor(s_interest)8                                  -1.835  0.06653 . 
## H3_interactiondisclaimer.new guideline:s_awarenesspass   0.227  0.82035   
## ---
## Signif. codes:  0 '***' 0.001 '**' 0.01 '*' 0.05 '.' 0.1 ' ' 1
## 
## Threshold coefficients:
##       Estimate Std. Error z value
## -6|-5  -5.4581     0.7708  -7.081
## -5|-4  -4.7623     0.5865  -8.119
## -4|-3  -3.0741     0.3768  -8.158
## -3|-2  -2.6679     0.3561  -7.491
## -2|-1  -1.2626     0.3236  -3.902
## -1|0   -0.7669     0.3200  -2.397
## 0|1     0.8411     0.3199   2.629
## 1|2     1.4047     0.3229   4.350
## 2|3     2.7948     0.3407   8.203
## 3|4     3.0315     0.3463   8.754
## 4|5     4.2642     0.4040  10.555
## 5|6     4.7466     0.4482  10.591
## (23 Beobachtungen als fehlend gelöscht)
```

```
exp(coef(diff_model8_2))
```

```
##                                                  -6|-5 
##                                           4.261512e-03 
##                                                  -5|-4 
##                                           8.546240e-03 
##                                                  -4|-3 
##                                           4.623109e-02 
##                                                  -3|-2 
##                                           6.939741e-02 
##                                                  -2|-1 
##                                           2.829156e-01 
##                                                   -1|0 
##                                           4.644447e-01 
##                                                    0|1 
##                                           2.319009e+00 
##                                                    1|2 
##                                           4.074329e+00 
##                                                    2|3 
##                                           1.635989e+01 
##                                                    3|4 
##                                           2.072832e+01 
##                                                    4|5 
##                                           7.110448e+01 
##                                                    5|6 
##                                           1.151946e+02 
##                 H3_interactiondisclaimer.new guideline 
##                                           9.815845e-01 
##                                        s_awarenesspass 
##                                           1.799726e+00 
##                                      text_orderFaerber 
##                                           1.566504e+00 
##                                                  s_age 
##                                           9.970034e-01 
##                                              s_sexmale 
##                                           9.118778e-01 
##                                           s_schoolReal 
##                                           1.167651e+00 
##                                            s_schoolAbi 
##                                           1.312031e+00 
##                                 as.factor(s_interest)5 
##                                           1.146176e+00 
##                                 as.factor(s_interest)6 
##                                           8.572742e-01 
##                                 as.factor(s_interest)7 
##                                           7.687225e-01 
##                                 as.factor(s_interest)8 
##                                           6.670855e-01 
## H3_interactiondisclaimer.new guideline:s_awarenesspass 
##                                           1.067616e+00
```

```
exp(confint((diff_model8_2)))
```

```
##                                                            2.5 %   97.5 %
## H3_interactiondisclaimer.new guideline                 0.6286427 1.533369
## s_awarenesspass                                        1.1892117 2.730148
## text_orderFaerber                                      1.1915187 2.061965
## s_age                                                  0.9881505 1.005915
## s_sexmale                                              0.6938016 1.198123
## s_schoolReal                                           0.8267911 1.649756
## s_schoolAbi                                            0.9369872 1.839099
## as.factor(s_interest)5                                 0.7542635 1.742525
## as.factor(s_interest)6                                 0.5546588 1.323845
## as.factor(s_interest)7                                 0.4849169 1.217616
## as.factor(s_interest)8                                 0.4324511 1.027397
## H3_interactiondisclaimer.new guideline:s_awarenesspass 0.6067614 1.877996
```

```
nagelkerke(fit = diff_model8_2, null = diff_null_1)
```

```
## $Models
##                                                                                                                                                       
## Model: "clm, as.factor(s_diff) ~ H3_interaction * s_awareness + text_order + s_age + s_sex + s_school + as.factor(s_interest), data2_wide_reg6, logit"
## Null:  "clm, as.factor(s_diff) ~ 1, data2_wide_reg5, logit"                                                                                           
## 
## $Pseudo.R.squared.for.model.vs.null
##                              Pseudo.R.squared
## McFadden                            0.0382867
## Cox and Snell (ML)                  0.1386810
## Nagelkerke (Cragg and Uhler)        0.1415480
## 
## $Likelihood.ratio.test
##  Df.diff LogLik.diff  Chisq    p.value
##      -12     -50.385 100.77 3.9323e-16
## 
## $Number.of.observations
##           
## Model: 675
## Null:  675
## 
## $Messages
## [1] "Note: For models fit with REML, these statistics are based on refitting with ML"
## 
## $Warnings
## [1] "None"
```

```
H3test_2 = emmeans(diff_model8_2, ~ H3_interaction)
```

```
## NOTE: Results may be misleading due to involvement in interactions
```

```
pairs(H3test_2, adjust = "tukey")
```

```
##  contrast                                               estimate    SE  df
##  no disclaimer.old guideline - disclaimer.new guideline  -0.0141 0.144 Inf
##  z.ratio p.value
##   -0.098  0.9218
## 
## Results are averaged over the levels of: s_awareness, text_order, s_sex, s_school, s_interest 
## Note: contrasts are still on the as.factor scale
```

```
cld(H3test_2, Letters = letters)
```

```
##  H3_interaction              emmean    SE  df asymp.LCL asymp.UCL .group
##  no disclaimer.old guideline  0.416 0.156 Inf     0.110     0.722  a    
##  disclaimer.new guideline     0.430 0.153 Inf     0.129     0.731  a    
## 
## Results are averaged over the levels of: s_awareness, text_order, s_sex, s_school, s_interest 
## Results are given on the as.factor (not the response) scale. 
## Confidence level used: 0.95 
## Note: contrasts are still on the as.factor scale 
## significance level used: alpha = 0.05 
## NOTE: If two or more means share the same grouping symbol,
##       then we cannot show them to be different.
##       But we also did not show them to be the same.
```

## H4

### H4a

```
H4a <- subset(data2_wide, condition == 3| condition == 4| condition == 6) 
View(H4a)

describeBy(H4a$s_causality, H4a$causality)
```

```
## 
##  Descriptive statistics by group 
## group: no causality statement
##    vars   n  mean   sd median trimmed  mad min max range skew kurtosis   se
## X1    1 342 -0.43 3.91      0   -0.46 4.45 -10  10    20 0.12    -0.23 0.21
## ------------------------------------------------------------ 
## group: causality statement
##    vars   n  mean   sd median trimmed  mad min max range skew kurtosis   se
## X1    1 645 -0.49 3.95      0   -0.54 2.97 -10  12    22  0.1    -0.19 0.16
```

```
wilcox.test(s_causality~causality, data = H4a, exact = FALSE,
            confint = TRUE)
```

```
## 
##  Wilcoxon rank sum test with continuity correction
## 
## data:  s_causality by causality
## W = 110914, p-value = 0.8841
## alternative hypothesis: true location shift is not equal to 0
```

#### H4a post hoc

```
H4a_1 <- subset(data2_wide, condition == 3| condition == 6) 
View(H4a_1)
describeBy(H4a_1$s_causality, H4a_1$causality)
```

```
## 
##  Descriptive statistics by group 
## group: no causality statement
##    vars   n  mean   sd median trimmed  mad min max range skew kurtosis   se
## X1    1 342 -0.43 3.91      0   -0.46 4.45 -10  10    20 0.12    -0.23 0.21
## ------------------------------------------------------------ 
## group: causality statement
##    vars   n mean  sd median trimmed  mad min max range skew kurtosis   se
## X1    1 320 -0.6 3.9      0   -0.67 2.97  -9  10    19 0.16    -0.21 0.22
```

```
wilcox.test(s_causality~causality, data = H4a_1, exact = FALSE,
            confint = TRUE)
```

```
## 
##  Wilcoxon rank sum test with continuity correction
## 
## data:  s_causality by causality
## W = 56246, p-value = 0.5331
## alternative hypothesis: true location shift is not equal to 0
```

```
H4a_2 <- subset(data2_wide, condition == 4| condition == 6) 
View(H4a_2)
describeBy(H4a_2$s_causality, H4a_2$causality)
```

```
## 
##  Descriptive statistics by group 
## group: no causality statement
##    vars   n  mean   sd median trimmed  mad min max range skew kurtosis   se
## X1    1 342 -0.43 3.91      0   -0.46 4.45 -10  10    20 0.12    -0.23 0.21
## ------------------------------------------------------------ 
## group: causality statement
##    vars   n  mean   sd median trimmed  mad min max range skew kurtosis   se
## X1    1 325 -0.39 4.01      0   -0.41 2.97 -10  12    22 0.04    -0.19 0.22
```

```
wilcox.test(s_causality~causality, data = H4a_2, exact = FALSE,
            confint = TRUE)
```

```
## 
##  Wilcoxon rank sum test with continuity correction
## 
## data:  s_causality by causality
## W = 54668, p-value = 0.7139
## alternative hypothesis: true location shift is not equal to 0
```

### H4b

```
H4b <- subset(data2_wide, condition == 1| condition == 2|
                condition == 3| condition == 4)
View(H4b)

describeBy(H4b$s_causality, H4b$causality)
```

```
## 
##  Descriptive statistics by group 
## group: no causality statement
##    vars   n  mean   sd median trimmed  mad min max range skew kurtosis   se
## X1    1 652 -0.65 3.84      0   -0.69 2.97 -10  10    20 0.11    -0.23 0.15
## ------------------------------------------------------------ 
## group: causality statement
##    vars   n  mean   sd median trimmed  mad min max range skew kurtosis   se
## X1    1 645 -0.49 3.95      0   -0.54 2.97 -10  12    22  0.1    -0.19 0.16
```

```
wilcox.test(s_causality~causality, data = H4b, exact = FALSE,
            confint = TRUE)
```

```
## 
##  Wilcoxon rank sum test with continuity correction
## 
## data:  s_causality by causality
## W = 205540, p-value = 0.481
## alternative hypothesis: true location shift is not equal to 0
```

#### H4b post hoc

```
H4b_1 <- subset(data2_wide, condition == 1|condition == 3)
View(H4b_1)
describeBy(H4b_1$s_causality, H4b_1$causality)
```

```
## 
##  Descriptive statistics by group 
## group: no causality statement
##    vars   n  mean   sd median trimmed  mad min max range skew kurtosis   se
## X1    1 323 -0.28 3.79      0   -0.33 2.97  -8  10    18 0.15    -0.21 0.21
## ------------------------------------------------------------ 
## group: causality statement
##    vars   n mean  sd median trimmed  mad min max range skew kurtosis   se
## X1    1 320 -0.6 3.9      0   -0.67 2.97  -9  10    19 0.16    -0.21 0.22
```

```
wilcox.test(s_causality~causality, data = H4b_1, exact = FALSE,
            confint = TRUE)
```

```
## 
##  Wilcoxon rank sum test with continuity correction
## 
## data:  s_causality by causality
## W = 54201, p-value = 0.2822
## alternative hypothesis: true location shift is not equal to 0
```

```
H4b_2 <- subset(data2_wide, condition == 2|condition == 4)
View(H4b_2)
describeBy(H4b_2$s_causality, H4b_2$causality)
```

```
## 
##  Descriptive statistics by group 
## group: no causality statement
##    vars   n  mean   sd median trimmed  mad min max range skew kurtosis   se
## X1    1 329 -1.02 3.87     -1   -1.05 4.45 -10  10    20 0.09     -0.3 0.21
## ------------------------------------------------------------ 
## group: causality statement
##    vars   n  mean   sd median trimmed  mad min max range skew kurtosis   se
## X1    1 325 -0.39 4.01      0   -0.41 2.97 -10  12    22 0.04    -0.19 0.22
```

```
wilcox.test(s_causality~causality, data = H4b_2, exact = FALSE,
            confint = TRUE)
```

```
## 
##  Wilcoxon rank sum test with continuity correction
## 
## data:  s_causality by causality
## W = 48566, p-value = 0.0416
## alternative hypothesis: true location shift is not equal to 0
```

```
H4b_3 <- subset(data2_wide, condition == 1|condition == 4)
View(H4b_3)
describeBy(H4b_3$s_causality, H4b_3$causality)
```

```
## 
##  Descriptive statistics by group 
## group: no causality statement
##    vars   n  mean   sd median trimmed  mad min max range skew kurtosis   se
## X1    1 323 -0.28 3.79      0   -0.33 2.97  -8  10    18 0.15    -0.21 0.21
## ------------------------------------------------------------ 
## group: causality statement
##    vars   n  mean   sd median trimmed  mad min max range skew kurtosis   se
## X1    1 325 -0.39 4.01      0   -0.41 2.97 -10  12    22 0.04    -0.19 0.22
```

```
wilcox.test(s_causality~causality, data = H4b_3, exact = FALSE,
            confint = TRUE)
```

```
## 
##  Wilcoxon rank sum test with continuity correction
## 
## data:  s_causality by causality
## W = 52827, p-value = 0.8862
## alternative hypothesis: true location shift is not equal to 0
```

```
H4b_4 <- subset(data2_wide, condition == 2|condition == 3)
View(H4b_4)
describeBy(H4b_4$s_causality, H4b_4$causality)
```

```
## 
##  Descriptive statistics by group 
## group: no causality statement
##    vars   n  mean   sd median trimmed  mad min max range skew kurtosis   se
## X1    1 329 -1.02 3.87     -1   -1.05 4.45 -10  10    20 0.09     -0.3 0.21
## ------------------------------------------------------------ 
## group: causality statement
##    vars   n mean  sd median trimmed  mad min max range skew kurtosis   se
## X1    1 320 -0.6 3.9      0   -0.67 2.97  -9  10    19 0.16    -0.21 0.22
```

```
wilcox.test(s_causality~causality, data = H4b_4, exact = FALSE,
            confint = TRUE)
```

```
## 
##  Wilcoxon rank sum test with continuity correction
## 
## data:  s_causality by causality
## W = 49946, p-value = 0.2572
## alternative hypothesis: true location shift is not equal to 0
```

### H4 Mixed Model

```
set.seed(288659)

data2_long$id <- as.numeric(data2_long$id)

data2_long$H4_interaction <- interaction(data2_long$causality, 
                                         data2_long$version)
data2_long$H4_interaction <- droplevels(data2_long$H4_interaction)
table(data2_long$H4_interaction)
```

```
## 
## no causality statement.old guideline no causality statement.new guideline 
##                                  714                                 1358 
##    causality statement.new guideline 
##                                 2010
```

```
data2_long_reg <- subset(data2_long, condition != 5)
View(data2_long_reg)

causality_null <- clm(as.factor(s_causality) ~ 1, data = data2_long_reg,
                       link = "logit")

causality_model1 <- clmm(as.factor(s_causality) ~ 1 + (1|id),
                                   data = data2_long_reg)
anova(causality_null,causality_model1)
```

```
## Likelihood ratio tests of cumulative link models:
##  
##                  formula:                              link: threshold:
## causality_null   as.factor(s_causality) ~ 1            logit flexible  
## causality_model1 as.factor(s_causality) ~ 1 + (1 | id) logit flexible  
## 
##                  no.par   AIC  logLik LR.stat df Pr(>Chisq)    
## causality_null       12 14339 -7157.5                          
## causality_model1     13 14253 -7113.4   88.32  1  < 2.2e-16 ***
## ---
## Signif. codes:  0 '***' 0.001 '**' 0.01 '*' 0.05 '.' 0.1 ' ' 1
```

```
causality_model2 <- clmm(as.factor(s_causality) ~ H4_interaction + (1|id),
                         data = data2_long_reg)
anova(causality_model1,causality_model2)
```

```
## Likelihood ratio tests of cumulative link models:
##  
##                  formula:                                           link:
## causality_model1 as.factor(s_causality) ~ 1 + (1 | id)              logit
## causality_model2 as.factor(s_causality) ~ H4_interaction + (1 | id) logit
##                  threshold:
## causality_model1 flexible  
## causality_model2 flexible  
## 
##                  no.par   AIC  logLik LR.stat df Pr(>Chisq)
## causality_model1     13 14253 -7113.4                      
## causality_model2     15 14256 -7112.8  1.1257  2     0.5696
```

```
causality_model3 <- clmm(as.factor(s_causality) ~ H4_interaction + s_awareness +
                           (1|id), data = data2_long_reg)
anova(causality_model2,causality_model3)
```

```
## Likelihood ratio tests of cumulative link models:
##  
##                  formula:                                                        
## causality_model2 as.factor(s_causality) ~ H4_interaction + (1 | id)              
## causality_model3 as.factor(s_causality) ~ H4_interaction + s_awareness + (1 | id)
##                  link: threshold:
## causality_model2 logit flexible  
## causality_model3 logit flexible  
## 
##                  no.par   AIC  logLik LR.stat df Pr(>Chisq)    
## causality_model2     15 14256 -7112.8                          
## causality_model3     16 14229 -7098.3  28.983  1  7.302e-08 ***
## ---
## Signif. codes:  0 '***' 0.001 '**' 0.01 '*' 0.05 '.' 0.1 ' ' 1
```

```
causality_model4 <- clmm(as.factor(s_causality) ~ H4_interaction*s_awareness +
                           (1|id), data = data2_long_reg)
```

```
## Warning in update.uC(rho): Non finite negative log-likelihood
##   at iteration 119
```

```
anova(causality_model3,causality_model4)
```

```
## Likelihood ratio tests of cumulative link models:
##  
##                  formula:                                                        
## causality_model3 as.factor(s_causality) ~ H4_interaction + s_awareness + (1 | id)
## causality_model4 as.factor(s_causality) ~ H4_interaction * s_awareness + (1 | id)
##                  link: threshold:
## causality_model3 logit flexible  
## causality_model4 logit flexible  
## 
##                  no.par   AIC  logLik LR.stat df Pr(>Chisq)  
## causality_model3     16 14229 -7098.3                        
## causality_model4     18 14226 -7095.2  6.1135  2    0.04704 *
## ---
## Signif. codes:  0 '***' 0.001 '**' 0.01 '*' 0.05 '.' 0.1 ' ' 1
```

```
causality_model5 <- clmm(as.factor(s_causality) ~ H4_interaction*s_awareness +
                           summary + (1|id), data = data2_long_reg)
anova(causality_model3,causality_model5)
```

```
## Likelihood ratio tests of cumulative link models:
##  
##                  formula:                                                                  
## causality_model3 as.factor(s_causality) ~ H4_interaction + s_awareness + (1 | id)          
## causality_model5 as.factor(s_causality) ~ H4_interaction * s_awareness + summary + (1 | id)
##                  link: threshold:
## causality_model3 logit flexible  
## causality_model5 logit flexible  
## 
##                  no.par   AIC  logLik LR.stat df Pr(>Chisq)   
## causality_model3     16 14229 -7098.3                         
## causality_model5     19 14221 -7091.3  14.036  3   0.002857 **
## ---
## Signif. codes:  0 '***' 0.001 '**' 0.01 '*' 0.05 '.' 0.1 ' ' 1
```

```
causality_model6 <- clmm(as.factor(s_causality) ~ H4_interaction*s_awareness +
                           summary + text_order + (1|id), data = data2_long_reg)
```

```
## Warning in update.uC(rho): Non finite negative log-likelihood
##   at iteration 131
```

```
anova(causality_model5,causality_model6)
```

```
## Likelihood ratio tests of cumulative link models:
##  
##                  formula:                                                                               
## causality_model5 as.factor(s_causality) ~ H4_interaction * s_awareness + summary + (1 | id)             
## causality_model6 as.factor(s_causality) ~ H4_interaction * s_awareness + summary + text_order + (1 | id)
##                  link: threshold:
## causality_model5 logit flexible  
## causality_model6 logit flexible  
## 
##                  no.par   AIC  logLik LR.stat df Pr(>Chisq)  
## causality_model5     19 14221 -7091.3                        
## causality_model6     20 14219 -7089.4  3.6952  1    0.05457 .
## ---
## Signif. codes:  0 '***' 0.001 '**' 0.01 '*' 0.05 '.' 0.1 ' ' 1
```

```
causality_model7 <- clmm(as.factor(s_causality) ~ H4_interaction*s_awareness +
                           summary + text_order + s_age + (1|id),
                         data = data2_long_reg)
anova(causality_model5,causality_model7)
```

```
## Likelihood ratio tests of cumulative link models:
##  
##                  formula:                                                                                       
## causality_model5 as.factor(s_causality) ~ H4_interaction * s_awareness + summary + (1 | id)                     
## causality_model7 as.factor(s_causality) ~ H4_interaction * s_awareness + summary + text_order + s_age + (1 | id)
##                  link: threshold:
## causality_model5 logit flexible  
## causality_model7 logit flexible  
## 
##                  no.par   AIC  logLik LR.stat df Pr(>Chisq)    
## causality_model5     19 14221 -7091.3                          
## causality_model7     21 14136 -7047.0    88.5  2  < 2.2e-16 ***
## ---
## Signif. codes:  0 '***' 0.001 '**' 0.01 '*' 0.05 '.' 0.1 ' ' 1
```

```
causality_model8 <- clmm(as.factor(s_causality) ~ H4_interaction*s_awareness +
                           summary + text_order + s_age + s_sex + (1|id),
                         data = data2_long_reg)
anova(causality_model7,causality_model8)
```

```
## Likelihood ratio tests of cumulative link models:
##  
##                  formula:                                                                                               
## causality_model7 as.factor(s_causality) ~ H4_interaction * s_awareness + summary + text_order + s_age + (1 | id)        
## causality_model8 as.factor(s_causality) ~ H4_interaction * s_awareness + summary + text_order + s_age + s_sex + (1 | id)
##                  link: threshold:
## causality_model7 logit flexible  
## causality_model8 logit flexible  
## 
##                  no.par   AIC  logLik LR.stat df Pr(>Chisq)  
## causality_model7     21 14136 -7047.0                        
## causality_model8     22 14133 -7044.4    5.18  1    0.02285 *
## ---
## Signif. codes:  0 '***' 0.001 '**' 0.01 '*' 0.05 '.' 0.1 ' ' 1
```

```
causality_model9 <- clmm(as.factor(s_causality) ~ H4_interaction*s_awareness +
                           summary + text_order + s_age + s_sex + s_school +
                           (1|id), data = data2_long_reg)
anova(causality_model7,causality_model9)
```

```
## Likelihood ratio tests of cumulative link models:
##  
##                  formula:                                                                                                          
## causality_model7 as.factor(s_causality) ~ H4_interaction * s_awareness + summary + text_order + s_age + (1 | id)                   
## causality_model9 as.factor(s_causality) ~ H4_interaction * s_awareness + summary + text_order + s_age + s_sex + s_school + (1 | id)
##                  link: threshold:
## causality_model7 logit flexible  
## causality_model9 logit flexible  
## 
##                  no.par   AIC  logLik LR.stat df Pr(>Chisq)    
## causality_model7     21 14136 -7047.0                          
## causality_model9     24 14096 -7023.9  46.309  3  4.876e-10 ***
## ---
## Signif. codes:  0 '***' 0.001 '**' 0.01 '*' 0.05 '.' 0.1 ' ' 1
```

```
causality_model10 <- clmm(as.factor(s_causality) ~ H4_interaction*s_awareness +
                           summary + text_order + s_age + s_sex + s_school +
                           as.factor(s_interest) + (1|id), data = data2_long_reg)
anova(causality_model9,causality_model10)
```

```
## Likelihood ratio tests of cumulative link models:
##  
##                   formula:                                                                                                                                  
## causality_model9  as.factor(s_causality) ~ H4_interaction * s_awareness + summary + text_order + s_age + s_sex + s_school + (1 | id)                        
## causality_model10 as.factor(s_causality) ~ H4_interaction * s_awareness + summary + text_order + s_age + s_sex + s_school + as.factor(s_interest) + (1 | id)
##                   link: threshold:
## causality_model9  logit flexible  
## causality_model10 logit flexible  
## 
##                   no.par   AIC  logLik LR.stat df Pr(>Chisq)    
## causality_model9      24 14096 -7023.9                          
## causality_model10     28 14072 -7008.1  31.653  4  2.252e-06 ***
## ---
## Signif. codes:  0 '***' 0.001 '**' 0.01 '*' 0.05 '.' 0.1 ' ' 1
```

```
summary(causality_model10)
```

```
## Cumulative Link Mixed Model fitted with the Laplace approximation
## 
## formula: as.factor(s_causality) ~ H4_interaction * s_awareness + summary +  
##     text_order + s_age + s_sex + s_school + as.factor(s_interest) +  
##     (1 | id)
## data:    data2_long_reg
## 
##  link  threshold nobs logLik   AIC      niter       max.grad cond.H 
##  logit flexible  3346 -7008.06 14072.11 4864(14592) 3.12e-02 1.0e+06
## 
## Random effects:
##  Groups Name        Variance Std.Dev.
##  id     (Intercept) 0.6998   0.8365  
## Number of groups:  id 1707 
## 
## Coefficients:
##                                                                     Estimate
## H4_interactionno causality statement.new guideline                  0.128746
## H4_interactioncausality statement.new guideline                    -0.014037
## s_awarenesspass                                                     0.560054
## summaryFaerber                                                     -0.167427
## text_orderFaerber                                                   0.157517
## s_age                                                              -0.022849
## s_sexmale                                                           0.137656
## s_schoolReal                                                        0.200122
## s_schoolAbi                                                         0.635815
## as.factor(s_interest)5                                              0.026437
## as.factor(s_interest)6                                             -0.100110
## as.factor(s_interest)7                                             -0.210662
## as.factor(s_interest)8                                             -0.573066
## H4_interactionno causality statement.new guideline:s_awarenesspass -0.320219
## H4_interactioncausality statement.new guideline:s_awarenesspass     0.118785
##                                                                    Std. Error
## H4_interactionno causality statement.new guideline                   0.180151
## H4_interactioncausality statement.new guideline                      0.174423
## s_awarenesspass                                                      0.177426
## summaryFaerber                                                       0.062329
## text_orderFaerber                                                    0.074599
## s_age                                                                0.002525
## s_sexmale                                                            0.075034
## s_schoolReal                                                         0.092225
## s_schoolAbi                                                          0.094534
## as.factor(s_interest)5                                               0.114523
## as.factor(s_interest)6                                               0.116301
## as.factor(s_interest)7                                               0.127171
## as.factor(s_interest)8                                               0.123157
## H4_interactionno causality statement.new guideline:s_awarenesspass   0.217091
## H4_interactioncausality statement.new guideline:s_awarenesspass      0.214019
##                                                                    z value
## H4_interactionno causality statement.new guideline                   0.715
## H4_interactioncausality statement.new guideline                     -0.080
## s_awarenesspass                                                      3.157
## summaryFaerber                                                      -2.686
## text_orderFaerber                                                    2.112
## s_age                                                               -9.049
## s_sexmale                                                            1.835
## s_schoolReal                                                         2.170
## s_schoolAbi                                                          6.726
## as.factor(s_interest)5                                               0.231
## as.factor(s_interest)6                                              -0.861
## as.factor(s_interest)7                                              -1.657
## as.factor(s_interest)8                                              -4.653
## H4_interactionno causality statement.new guideline:s_awarenesspass  -1.475
## H4_interactioncausality statement.new guideline:s_awarenesspass      0.555
##                                                                    Pr(>|z|)    
## H4_interactionno causality statement.new guideline                  0.47482    
## H4_interactioncausality statement.new guideline                     0.93586    
## s_awarenesspass                                                     0.00160 ** 
## summaryFaerber                                                      0.00723 ** 
## text_orderFaerber                                                   0.03473 *  
## s_age                                                               < 2e-16 ***
## s_sexmale                                                           0.06657 .  
## s_schoolReal                                                        0.03001 *  
## s_schoolAbi                                                        1.75e-11 ***
## as.factor(s_interest)5                                              0.81744    
## as.factor(s_interest)6                                              0.38935    
## as.factor(s_interest)7                                              0.09761 .  
## as.factor(s_interest)8                                             3.27e-06 ***
## H4_interactionno causality statement.new guideline:s_awarenesspass  0.14020    
## H4_interactioncausality statement.new guideline:s_awarenesspass     0.57888    
## ---
## Signif. codes:  0 '***' 0.001 '**' 0.01 '*' 0.05 '.' 0.1 ' ' 1
## 
## Threshold coefficients:
##       Estimate Std. Error z value
## -6|-5  -6.7316     0.3751 -17.945
## -5|-4  -6.0785     0.3120 -19.485
## -4|-3  -2.6165     0.2193 -11.931
## -3|-2  -2.1636     0.2161 -10.012
## -2|-1  -1.3085     0.2118  -6.177
## -1|0   -0.8773     0.2105  -4.167
## 0|1     0.3906     0.2100   1.860
## 1|2     0.7958     0.2110   3.772
## 2|3     1.8228     0.2156   8.456
## 3|4     2.2169     0.2184  10.151
## 4|5     3.8264     0.2441  15.674
## 5|6     4.2208     0.2574  16.397
## (80 Beobachtungen als fehlend gelöscht)
```

```
exp(coef(causality_model10))
```

```
##                                                              -6|-5 
##                                                        0.001192662 
##                                                              -5|-4 
##                                                        0.002291605 
##                                                              -4|-3 
##                                                        0.073059762 
##                                                              -3|-2 
##                                                        0.114905968 
##                                                              -2|-1 
##                                                        0.270213031 
##                                                               -1|0 
##                                                        0.415898556 
##                                                                0|1 
##                                                        1.477834658 
##                                                                1|2 
##                                                        2.216168478 
##                                                                2|3 
##                                                        6.188854547 
##                                                                3|4 
##                                                        9.178383300 
##                                                                4|5 
##                                                       45.895289519 
##                                                                5|6 
##                                                       68.089209945 
##                 H4_interactionno causality statement.new guideline 
##                                                        1.137401433 
##                    H4_interactioncausality statement.new guideline 
##                                                        0.986061512 
##                                                    s_awarenesspass 
##                                                        1.750766673 
##                                                     summaryFaerber 
##                                                        0.845838072 
##                                                  text_orderFaerber 
##                                                        1.170600718 
##                                                              s_age 
##                                                        0.977410071 
##                                                          s_sexmale 
##                                                        1.147580946 
##                                                       s_schoolReal 
##                                                        1.221552276 
##                                                        s_schoolAbi 
##                                                        1.888560451 
##                                             as.factor(s_interest)5 
##                                                        1.026789415 
##                                             as.factor(s_interest)6 
##                                                        0.904737468 
##                                             as.factor(s_interest)7 
##                                                        0.810047938 
##                                             as.factor(s_interest)8 
##                                                        0.563794369 
## H4_interactionno causality statement.new guideline:s_awarenesspass 
##                                                        0.725990156 
##    H4_interactioncausality statement.new guideline:s_awarenesspass 
##                                                        1.126128325
```

```
exp(confint(causality_model10))
```

```
##                                                                           2.5 %
## -6|-5                                                               0.000571748
## -5|-4                                                               0.001243362
## -4|-3                                                               0.047535094
## -3|-2                                                               0.075229893
## -2|-1                                                               0.178399335
## -1|0                                                                0.275294354
## 0|1                                                                 0.979161888
## 1|2                                                                 1.465559546
## 2|3                                                                 4.056332112
## 3|4                                                                 5.982364104
## 4|5                                                                28.442310254
## 5|6                                                                41.112481197
## H4_interactionno causality statement.new guideline                  0.799041199
## H4_interactioncausality statement.new guideline                     0.700542974
## s_awarenesspass                                                     1.236524653
## summaryFaerber                                                      0.748570411
## text_orderFaerber                                                   1.011369247
## s_age                                                               0.972584762
## s_sexmale                                                           0.990636499
## s_schoolReal                                                        1.019553274
## s_schoolAbi                                                         1.569146432
## as.factor(s_interest)5                                              0.820349456
## as.factor(s_interest)6                                              0.720322608
## as.factor(s_interest)7                                              0.631339351
## as.factor(s_interest)8                                              0.442883331
## H4_interactionno causality statement.new guideline:s_awarenesspass  0.474398106
## H4_interactioncausality statement.new guideline:s_awarenesspass     0.740311909
##                                                                          97.5 %
## -6|-5                                                              2.487884e-03
## -5|-4                                                              4.223590e-03
## -4|-3                                                              1.122903e-01
## -3|-2                                                              1.755071e-01
## -2|-1                                                              4.092789e-01
## -1|0                                                               6.283151e-01
## 0|1                                                                2.230474e+00
## 1|2                                                                3.351213e+00
## 2|3                                                                9.442501e+00
## 3|4                                                                1.408184e+01
## 4|5                                                                7.405789e+01
## 5|6                                                                1.127672e+02
## H4_interactionno causality statement.new guideline                 1.619043e+00
## H4_interactioncausality statement.new guideline                    1.387948e+00
## s_awarenesspass                                                    2.478870e+00
## summaryFaerber                                                     9.557445e-01
## text_orderFaerber                                                  1.354902e+00
## s_age                                                              9.822593e-01
## s_sexmale                                                          1.329390e+00
## s_schoolReal                                                       1.463572e+00
## s_schoolAbi                                                        2.272994e+00
## as.factor(s_interest)5                                             1.285180e+00
## as.factor(s_interest)6                                             1.136366e+00
## as.factor(s_interest)7                                             1.039342e+00
## as.factor(s_interest)8                                             7.177152e-01
## H4_interactionno causality statement.new guideline:s_awarenesspass 1.111011e+00
## H4_interactioncausality statement.new guideline:s_awarenesspass    1.713014e+00
```

```
nagelkerke(fit = causality_model10, null = causality_null)
```

```
## $Models
##                                                                                                                                                                          
## Model: "clmm, as.factor(s_causality) ~ H4_interaction * s_awareness + summary + text_order + s_age + s_sex + s_school + as.factor(s_interest) + (1 | id), data2_long_reg"
## Null:  "clm, as.factor(s_causality) ~ 1, data2_long_reg, logit"                                                                                                          
## 
## $Pseudo.R.squared.for.model.vs.null
##                              Pseudo.R.squared
## McFadden                            0.0208820
## Cox and Snell (ML)                  0.0854639
## Nagelkerke (Cragg and Uhler)        0.0866657
## 
## $Likelihood.ratio.test
##  Df.diff LogLik.diff  Chisq    p.value
##      -16     -149.46 298.93 4.2561e-54
## 
## $Number.of.observations
##            
## Model: 3346
## Null:  3346
## 
## $Messages
## [1] "Note: For models fit with REML, these statistics are based on refitting with ML"
## 
## $Warnings
## [1] "None"
```

```
H4test = emmeans(causality_model10, ~ H4_interaction)
```

```
## NOTE: Results may be misleading due to involvement in interactions
```

```
pairs(H4test, adjust = "tukey")
```

```
##  contrast                                                                   
##  no causality statement.old guideline - no causality statement.new guideline
##  no causality statement.old guideline - causality statement.new guideline   
##  no causality statement.new guideline - causality statement.new guideline   
##  estimate    SE  df z.ratio p.value
##    0.0314 0.109 Inf   0.289  0.9551
##   -0.0454 0.107 Inf  -0.424  0.9056
##   -0.0767 0.088 Inf  -0.871  0.6583
## 
## Results are averaged over the levels of: s_awareness, summary, text_order, s_sex, s_school, s_interest 
## Note: contrasts are still on the as.factor scale 
## P value adjustment: tukey method for comparing a family of 3 estimates
```

```
cld(H4test, Letters = letters)
```

```
##  H4_interaction                         emmean     SE  df asymp.LCL asymp.UCL
##  no causality statement.new guideline -0.07559 0.0783 Inf    -0.229    0.0778
##  no causality statement.old guideline -0.04423 0.0989 Inf    -0.238    0.1496
##  causality statement.new guideline     0.00113 0.0756 Inf    -0.147    0.1493
##  .group
##   a    
##   a    
##   a    
## 
## Results are averaged over the levels of: s_awareness, summary, text_order, s_sex, s_school, s_interest 
## Results are given on the as.factor (not the response) scale. 
## Confidence level used: 0.95 
## Note: contrasts are still on the as.factor scale
[truncated: 673,309 more chars]
