# Supplementary material for: Equivalent user experience and improved community augmented meta-analyses knowledge for a new version of a Plain Language Summary guideline
Source: PLoS One. 2024 May 9;19(5):e0300675. doi: 10.1371/journal.pone.0300675 (PMC11081257; doi:10.1371/journal.pone.0300675)
Supplement: S5 File — https://doi.org/10.23668/psycharchives.14219. (ZIP) [file pone.0300675.s005.zip › S5_File.html]

S5. Shortened R Markdown


# S5. Shortened R Markdown

#### 2024-01-23

# Preparation

## Load Dataframe

```
data <- read.csv2("data_project_917982_2022_11_15.csv",
                  na.strings = c("-66","-77","-99"),
                  encoding = "UFT-8")
View(data)
names(data)
```

```
##   [1] "lfdn"                "external_lfdn"       "tester"             
##   [4] "dispcode"            "lastpage"            "quality"            
##   [7] "duration"            "c_0001"              "p_0001"             
##  [10] "c_0002"              "c_0003"              "c_0004"             
##  [13] "v_1"                 "v_2"                 "v_3"                
##  [16] "v_4"                 "v_5"                 "v_7"                
##  [19] "v_8"                 "v_9"                 "v_10"               
##  [22] "v_11"                "v_47"                "v_48"               
##  [25] "v_49"                "v_12"                "v_14"               
##  [28] "v_16"                "v_71"                "v_17"               
##  [31] "v_18"                "v_19"                "v_20"               
##  [34] "v_21"                "v_115"               "v_116"              
##  [37] "v_117"               "v_22"                "v_23"               
##  [40] "v_24"                "v_25"                "v_26"               
##  [43] "v_120"               "v_27"                "v_28"               
##  [46] "v_29"                "v_30"                "v_31"               
##  [49] "v_121"               "v_32"                "v_33"               
##  [52] "v_34"                "v_35"                "v_36"               
##  [55] "v_122"               "v_37"                "v_38"               
##  [58] "v_39"                "v_40"                "v_41"               
##  [61] "v_123"               "v_124"               "v_42"               
##  [64] "v_43"                "v_44"                "v_45"               
##  [67] "v_46"                "v_125"               "v_72"               
##  [70] "v_73"                "v_74"                "v_75"               
##  [73] "v_76"                "v_77"                "v_79"               
##  [76] "v_81"                "v_83"                "v_126"              
##  [79] "v_127"               "v_128"               "v_129"              
##  [82] "v_130"               "v_131"               "v_132"              
##  [85] "v_133"               "v_134"               "v_135"              
##  [88] "v_136"               "v_137"               "v_138"              
##  [91] "v_139"               "v_140"               "v_141"              
##  [94] "v_142"               "v_143"               "v_144"              
##  [97] "v_145"               "v_146"               "v_147"              
## [100] "v_148"               "v_149"               "v_150"              
## [103] "v_151"               "v_152"               "v_153"              
## [106] "v_154"               "v_155"               "v_156"              
## [109] "v_157"               "v_158"               "v_159"              
## [112] "v_160"               "v_161"               "v_162"              
## [115] "v_163"               "v_164"               "v_50"               
## [118] "v_51"                "v_52"                "v_53"               
## [121] "v_54"                "v_165"               "v_166"              
## [124] "v_167"               "v_55"                "v_56"               
## [127] "v_57"                "v_58"                "v_401"              
## [130] "v_91"                "v_92"                "v_93"               
## [133] "v_94"                "v_95"                "v_96"               
## [136] "v_98"                "v_100"               "v_102"              
## [139] "v_235"               "v_236"               "v_237"              
## [142] "v_238"               "v_239"               "v_240"              
## [145] "v_241"               "v_242"               "v_243"              
## [148] "v_244"               "v_245"               "v_246"              
## [151] "v_247"               "v_248"               "v_249"              
## [154] "v_250"               "v_251"               "v_252"              
## [157] "v_253"               "v_254"               "v_255"              
## [160] "v_256"               "v_257"               "v_258"              
## [163] "v_259"               "v_313"               "v_314"              
## [166] "v_315"               "v_316"               "v_317"              
## [169] "v_323"               "v_324"               "v_325"              
## [172] "v_326"               "v_327"               "v_328"              
## [175] "v_329"               "v_330"               "v_331"              
## [178] "v_332"               "v_333"               "v_334"              
## [181] "v_335"               "v_336"               "v_337"              
## [184] "v_338"               "v_339"               "v_340"              
## [187] "v_341"               "v_342"               "v_343"              
## [190] "v_344"               "v_345"               "v_103"              
## [193] "v_104"               "v_105"               "v_106"              
## [196] "v_107"               "v_108"               "v_110"              
## [199] "v_112"               "v_114"               "v_274"              
## [202] "v_275"               "v_276"               "v_277"              
## [205] "v_278"               "v_279"               "v_280"              
## [208] "v_281"               "v_282"               "v_283"              
## [211] "v_284"               "v_285"               "v_286"              
## [214] "v_287"               "v_288"               "v_289"              
## [217] "v_290"               "v_291"               "v_292"              
## [220] "v_293"               "v_294"               "v_295"              
## [223] "v_296"               "v_297"               "v_298"              
## [226] "v_299"               "v_300"               "v_301"              
## [229] "v_302"               "v_303"               "v_304"              
## [232] "v_305"               "v_306"               "v_307"              
## [235] "v_308"               "v_309"               "v_310"              
## [238] "v_402"               "v_360"               "v_361"              
## [241] "v_362"               "v_363"               "v_364"              
## [244] "v_365"               "v_366"               "v_367"              
## [247] "v_368"               "v_369"               "v_370"              
## [250] "v_371"               "v_372"               "v_373"              
## [253] "v_374"               "v_375"               "v_376"              
## [256] "v_377"               "v_378"               "v_379"              
## [259] "v_380"               "v_381"               "v_382"              
## [262] "v_383"               "v_384"               "v_385"              
## [265] "v_386"               "v_387"               "v_388"              
## [268] "browser"             "referer"             "device_type"        
## [271] "quota"               "quota_assignment"    "quota_rejected_id"  
## [274] "page_history"        "hflip"               "vflip"              
## [277] "output_mode"         "javascript"          "flash"              
## [280] "session_id"          "language"            "cleaned"            
## [283] "ats"                 "datetime"            "date_of_last_access"
## [286] "date_of_first_mail"  "rts6018385"          "rts6018739"         
## [289] "rts6018818"          "rts6019080"          "rts6019089"         
## [292] "rts6021451"          "rts6021455"          "rts6023513"         
## [295] "rts6023515"          "rts6023627"          "rts6023655"         
## [298] "rts6023657"          "rts6023660"          "rts6023667"         
## [301] "rts6023676"          "rts6023679"          "rts6033975"
```

```
str(data)
```

```
## 'data.frame':    6706 obs. of  303 variables:
##  $ lfdn               : int  94 95 98 99 100 101 107 109 93 103 ...
##  $ external_lfdn      : int  0 0 0 0 0 0 0 0 0 0 ...
##  $ tester             : int  0 0 0 0 0 0 0 0 0 0 ...
##  $ dispcode           : int  37 37 37 37 37 37 37 37 31 22 ...
##  $ lastpage           : int  6018729 6018729 6018729 6018729 6018729 6018729 6018729 6018729 6018381 6023627 ...
##  $ quality            : logi  NA NA NA NA NA NA ...
##  $ duration           : int  23 57 19 25 23 37 30 38 779 68 ...
##  $ c_0001             : int  NA NA NA NA NA NA NA NA 3 2 ...
##  $ p_0001             : num  2.26e+14 2.26e+14 2.26e+14 2.26e+14 2.26e+14 ...
##  $ c_0002             : int  NA NA NA NA NA NA NA NA 3 2 ...
##  $ c_0003             : int  NA NA NA NA NA NA NA NA 1 2 ...
##  $ c_0004             : int  NA NA NA NA NA NA NA NA 2 2 ...
##  $ v_1                : int  2 1 2 1 2 1 1 2 2 2 ...
##  $ v_2                : int  48 19 55 55 33 44 64 43 31 42 ...
##  $ v_3                : int  1 1 2 2 1 1 3 3 2 2 ...
##  $ v_4                : int  1 1 1 1 1 1 1 1 1 1 ...
##  $ v_5                : int  2 2 2 2 2 2 2 2 2 2 ...
##  $ v_7                : int  1 3 1 2 1 1 3 2 5 6 ...
##  $ v_8                : int  NA NA NA NA NA NA NA NA 1 1 ...
##  $ v_9                : chr  NA NA NA NA ...
##  $ v_10               : int  NA NA NA NA NA NA NA NA 4 NA ...
##  $ v_11               : int  NA NA NA NA NA NA NA NA 6 NA ...
##  $ v_47               : int  NA NA NA NA NA NA NA NA 3 NA ...
##  $ v_48               : int  NA NA NA NA NA NA NA NA 6 NA ...
##  $ v_49               : int  NA NA NA NA NA NA NA NA 6 NA ...
##  $ v_12               : int  NA NA NA NA NA NA NA NA 2 NA ...
##  $ v_14               : int  NA NA NA NA NA NA NA NA 1 NA ...
##  $ v_16               : int  NA NA NA NA NA NA NA NA 3 NA ...
##  $ v_71               : int  NA NA NA NA NA NA NA NA 3 NA ...
##  $ v_17               : int  NA NA NA NA NA NA NA NA 1 NA ...
##  $ v_18               : int  NA NA NA NA NA NA NA NA 1 NA ...
##  $ v_19               : int  NA NA NA NA NA NA NA NA 2 NA ...
##  $ v_20               : int  NA NA NA NA NA NA NA NA 2 NA ...
##  $ v_21               : int  NA NA NA NA NA NA NA NA 1 NA ...
##  $ v_115              : int  NA NA NA NA NA NA NA NA 1 NA ...
##  $ v_116              : int  NA NA NA NA NA NA NA NA 1 NA ...
##  $ v_117              : int  NA NA NA NA NA NA NA NA 1 NA ...
##  $ v_22               : int  NA NA NA NA NA NA NA NA 3 NA ...
##  $ v_23               : int  NA NA NA NA NA NA NA NA 1 NA ...
##  $ v_24               : int  NA NA NA NA NA NA NA NA 1 NA ...
##  $ v_25               : int  NA NA NA NA NA NA NA NA 1 NA ...
##  $ v_26               : int  NA NA NA NA NA NA NA NA 1 NA ...
##  $ v_120              : int  NA NA NA NA NA NA NA NA 1 NA ...
##  $ v_27               : int  NA NA NA NA NA NA NA NA 2 NA ...
##  $ v_28               : int  NA NA NA NA NA NA NA NA 2 NA ...
##  $ v_29               : int  NA NA NA NA NA NA NA NA 2 NA ...
##  $ v_30               : int  NA NA NA NA NA NA NA NA 2 NA ...
##  $ v_31               : int  NA NA NA NA NA NA NA NA 1 NA ...
##  $ v_121              : int  NA NA NA NA NA NA NA NA 1 NA ...
##  $ v_32               : int  NA NA NA NA NA NA NA NA 2 NA ...
##  $ v_33               : int  NA NA NA NA NA NA NA NA 2 NA ...
##  $ v_34               : int  NA NA NA NA NA NA NA NA 2 NA ...
##  $ v_35               : int  NA NA NA NA NA NA NA NA 2 NA ...
##  $ v_36               : int  NA NA NA NA NA NA NA NA 2 NA ...
##  $ v_122              : int  NA NA NA NA NA NA NA NA 1 NA ...
##  $ v_37               : int  NA NA NA NA NA NA NA NA 2 NA ...
##  $ v_38               : int  NA NA NA NA NA NA NA NA 0 NA ...
##  $ v_39               : int  NA NA NA NA NA NA NA NA 2 NA ...
##  $ v_40               : int  NA NA NA NA NA NA NA NA 2 NA ...
##  $ v_41               : int  NA NA NA NA NA NA NA NA 1 NA ...
##  $ v_123              : int  NA NA NA NA NA NA NA NA 1 NA ...
##  $ v_124              : int  NA NA NA NA NA NA NA NA 2 NA ...
##  $ v_42               : int  NA NA NA NA NA NA NA NA 3 NA ...
##  $ v_43               : int  NA NA NA NA NA NA NA NA 1 NA ...
##  $ v_44               : int  NA NA NA NA NA NA NA NA 3 NA ...
##  $ v_45               : int  NA NA NA NA NA NA NA NA 1 NA ...
##  $ v_46               : int  NA NA NA NA NA NA NA NA 3 NA ...
##  $ v_125              : int  NA NA NA NA NA NA NA NA 3 NA ...
##  $ v_72               : int  NA NA NA NA NA NA NA NA NA 6 ...
##  $ v_73               : int  NA NA NA NA NA NA NA NA NA 7 ...
##  $ v_74               : int  NA NA NA NA NA NA NA NA NA 7 ...
##  $ v_75               : int  NA NA NA NA NA NA NA NA NA 7 ...
##  $ v_76               : int  NA NA NA NA NA NA NA NA NA 7 ...
##  $ v_77               : int  NA NA NA NA NA NA NA NA NA 4 ...
##  $ v_79               : int  NA NA NA NA NA NA NA NA NA 1 ...
##  $ v_81               : int  NA NA NA NA NA NA NA NA NA 1 ...
##  $ v_83               : int  NA NA NA NA NA NA NA NA NA 1 ...
##  $ v_126              : int  NA NA NA NA NA NA NA NA NA NA ...
##  $ v_127              : int  NA NA NA NA NA NA NA NA NA NA ...
##  $ v_128              : int  NA NA NA NA NA NA NA NA NA NA ...
##  $ v_129              : int  NA NA NA NA NA NA NA NA NA NA ...
##  $ v_130              : int  NA NA NA NA NA NA NA NA NA NA ...
##  $ v_131              : int  NA NA NA NA NA NA NA NA NA NA ...
##  $ v_132              : int  NA NA NA NA NA NA NA NA NA NA ...
##  $ v_133              : int  NA NA NA NA NA NA NA NA NA NA ...
##  $ v_134              : int  NA NA NA NA NA NA NA NA NA NA ...
##  $ v_135              : int  NA NA NA NA NA NA NA NA NA NA ...
##  $ v_136              : int  NA NA NA NA NA NA NA NA NA NA ...
##  $ v_137              : int  NA NA NA NA NA NA NA NA NA NA ...
##  $ v_138              : int  NA NA NA NA NA NA NA NA NA NA ...
##  $ v_139              : int  NA NA NA NA NA NA NA NA NA NA ...
##  $ v_140              : int  NA NA NA NA NA NA NA NA NA NA ...
##  $ v_141              : int  NA NA NA NA NA NA NA NA NA NA ...
##  $ v_142              : int  NA NA NA NA NA NA NA NA NA NA ...
##  $ v_143              : int  NA NA NA NA NA NA NA NA NA NA ...
##  $ v_144              : int  NA NA NA NA NA NA NA NA NA NA ...
##  $ v_145              : int  NA NA NA NA NA NA NA NA NA NA ...
##  $ v_146              : int  NA NA NA NA NA NA NA NA NA NA ...
##  $ v_147              : int  NA NA NA NA NA NA NA NA NA NA ...
##   [list output truncated]
```

```
nrow(data)
```

```
## [1] 6706
```

## Check Dispcodes

```
table(data$dispcode)
```

```
## 
##   20   22   31   32   36   37 
##   45 1039 1754  288 1474 2106
```

```
# 20 = Not started yet --> 45
# 22 = Interrupted --> 1039
# 37,38,39,40 = Screenout --> 2106
# 35,36,41 = Quota full --> 1474
# 31,32,33,34 = Finished --> 2042

# Check for multiple participations
x <- table(data$p_0001[data$dispcode==31|
                         data$dispcode==32])

code <- dimnames(x)[[1]]
code <- code[x>1]
code
```

```
## [1] "225951288721641"
```

```
#Case 225951288721641 participated twice. Exclude second participation.

data$lfdn[data$p_0001 == "225951288721641"]
```

```
## [1]   NA 1732 3192
```

```
data$datetime[data$p_0001 == "225951288721641"]
```

```
## [1] NA                    "2022-10-26 07:45:35" "2022-10-27 17:38:04"
```

```
data <- data[!data$lfdn == 3192,]
```

## Recode Data

```
data$lfdn <- rank(rnorm(nrow(data)))
names(data)[1] <- "id"

data$id <- factor(data$id,levels = c(1:6705))

str(data)
```

```
## 'data.frame':    6705 obs. of  303 variables:
##  $ id                 : Factor w/ 6705 levels "1","2","3","4",..: 4207 77 2660 3420 1541 2601 2041 2234 3600 4018 ...
##  $ external_lfdn      : int  0 0 0 0 0 0 0 0 0 0 ...
##  $ tester             : int  0 0 0 0 0 0 0 0 0 0 ...
##  $ dispcode           : int  37 37 37 37 37 37 37 37 31 22 ...
##  $ lastpage           : int  6018729 6018729 6018729 6018729 6018729 6018729 6018729 6018729 6018381 6023627 ...
##  $ quality            : logi  NA NA NA NA NA NA ...
##  $ duration           : int  23 57 19 25 23 37 30 38 779 68 ...
##  $ c_0001             : int  NA NA NA NA NA NA NA NA 3 2 ...
##  $ p_0001             : num  2.26e+14 2.26e+14 2.26e+14 2.26e+14 2.26e+14 ...
##  $ c_0002             : int  NA NA NA NA NA NA NA NA 3 2 ...
##  $ c_0003             : int  NA NA NA NA NA NA NA NA 1 2 ...
##  $ c_0004             : int  NA NA NA NA NA NA NA NA 2 2 ...
##  $ v_1                : int  2 1 2 1 2 1 1 2 2 2 ...
##  $ v_2                : int  48 19 55 55 33 44 64 43 31 42 ...
##  $ v_3                : int  1 1 2 2 1 1 3 3 2 2 ...
##  $ v_4                : int  1 1 1 1 1 1 1 1 1 1 ...
##  $ v_5                : int  2 2 2 2 2 2 2 2 2 2 ...
##  $ v_7                : int  1 3 1 2 1 1 3 2 5 6 ...
##  $ v_8                : int  NA NA NA NA NA NA NA NA 1 1 ...
##  $ v_9                : chr  NA NA NA NA ...
##  $ v_10               : int  NA NA NA NA NA NA NA NA 4 NA ...
##  $ v_11               : int  NA NA NA NA NA NA NA NA 6 NA ...
##  $ v_47               : int  NA NA NA NA NA NA NA NA 3 NA ...
##  $ v_48               : int  NA NA NA NA NA NA NA NA 6 NA ...
##  $ v_49               : int  NA NA NA NA NA NA NA NA 6 NA ...
##  $ v_12               : int  NA NA NA NA NA NA NA NA 2 NA ...
##  $ v_14               : int  NA NA NA NA NA NA NA NA 1 NA ...
##  $ v_16               : int  NA NA NA NA NA NA NA NA 3 NA ...
##  $ v_71               : int  NA NA NA NA NA NA NA NA 3 NA ...
##  $ v_17               : int  NA NA NA NA NA NA NA NA 1 NA ...
##  $ v_18               : int  NA NA NA NA NA NA NA NA 1 NA ...
##  $ v_19               : int  NA NA NA NA NA NA NA NA 2 NA ...
##  $ v_20               : int  NA NA NA NA NA NA NA NA 2 NA ...
##  $ v_21               : int  NA NA NA NA NA NA NA NA 1 NA ...
##  $ v_115              : int  NA NA NA NA NA NA NA NA 1 NA ...
##  $ v_116              : int  NA NA NA NA NA NA NA NA 1 NA ...
##  $ v_117              : int  NA NA NA NA NA NA NA NA 1 NA ...
##  $ v_22               : int  NA NA NA NA NA NA NA NA 3 NA ...
##  $ v_23               : int  NA NA NA NA NA NA NA NA 1 NA ...
##  $ v_24               : int  NA NA NA NA NA NA NA NA 1 NA ...
##  $ v_25               : int  NA NA NA NA NA NA NA NA 1 NA ...
##  $ v_26               : int  NA NA NA NA NA NA NA NA 1 NA ...
##  $ v_120              : int  NA NA NA NA NA NA NA NA 1 NA ...
##  $ v_27               : int  NA NA NA NA NA NA NA NA 2 NA ...
##  $ v_28               : int  NA NA NA NA NA NA NA NA 2 NA ...
##  $ v_29               : int  NA NA NA NA NA NA NA NA 2 NA ...
##  $ v_30               : int  NA NA NA NA NA NA NA NA 2 NA ...
##  $ v_31               : int  NA NA NA NA NA NA NA NA 1 NA ...
##  $ v_121              : int  NA NA NA NA NA NA NA NA 1 NA ...
##  $ v_32               : int  NA NA NA NA NA NA NA NA 2 NA ...
##  $ v_33               : int  NA NA NA NA NA NA NA NA 2 NA ...
##  $ v_34               : int  NA NA NA NA NA NA NA NA 2 NA ...
##  $ v_35               : int  NA NA NA NA NA NA NA NA 2 NA ...
##  $ v_36               : int  NA NA NA NA NA NA NA NA 2 NA ...
##  $ v_122              : int  NA NA NA NA NA NA NA NA 1 NA ...
##  $ v_37               : int  NA NA NA NA NA NA NA NA 2 NA ...
##  $ v_38               : int  NA NA NA NA NA NA NA NA 0 NA ...
##  $ v_39               : int  NA NA NA NA NA NA NA NA 2 NA ...
##  $ v_40               : int  NA NA NA NA NA NA NA NA 2 NA ...
##  $ v_41               : int  NA NA NA NA NA NA NA NA 1 NA ...
##  $ v_123              : int  NA NA NA NA NA NA NA NA 1 NA ...
##  $ v_124              : int  NA NA NA NA NA NA NA NA 2 NA ...
##  $ v_42               : int  NA NA NA NA NA NA NA NA 3 NA ...
##  $ v_43               : int  NA NA NA NA NA NA NA NA 1 NA ...
##  $ v_44               : int  NA NA NA NA NA NA NA NA 3 NA ...
##  $ v_45               : int  NA NA NA NA NA NA NA NA 1 NA ...
##  $ v_46               : int  NA NA NA NA NA NA NA NA 3 NA ...
##  $ v_125              : int  NA NA NA NA NA NA NA NA 3 NA ...
##  $ v_72               : int  NA NA NA NA NA NA NA NA NA 6 ...
##  $ v_73               : int  NA NA NA NA NA NA NA NA NA 7 ...
##  $ v_74               : int  NA NA NA NA NA NA NA NA NA 7 ...
##  $ v_75               : int  NA NA NA NA NA NA NA NA NA 7 ...
##  $ v_76               : int  NA NA NA NA NA NA NA NA NA 7 ...
##  $ v_77               : int  NA NA NA NA NA NA NA NA NA 4 ...
##  $ v_79               : int  NA NA NA NA NA NA NA NA NA 1 ...
##  $ v_81               : int  NA NA NA NA NA NA NA NA NA 1 ...
##  $ v_83               : int  NA NA NA NA NA NA NA NA NA 1 ...
##  $ v_126              : int  NA NA NA NA NA NA NA NA NA NA ...
##  $ v_127              : int  NA NA NA NA NA NA NA NA NA NA ...
##  $ v_128              : int  NA NA NA NA NA NA NA NA NA NA ...
##  $ v_129              : int  NA NA NA NA NA NA NA NA NA NA ...
##  $ v_130              : int  NA NA NA NA NA NA NA NA NA NA ...
##  $ v_131              : int  NA NA NA NA NA NA NA NA NA NA ...
##  $ v_132              : int  NA NA NA NA NA NA NA NA NA NA ...
##  $ v_133              : int  NA NA NA NA NA NA NA NA NA NA ...
##  $ v_134              : int  NA NA NA NA NA NA NA NA NA NA ...
##  $ v_135              : int  NA NA NA NA NA NA NA NA NA NA ...
##  $ v_136              : int  NA NA NA NA NA NA NA NA NA NA ...
##  $ v_137              : int  NA NA NA NA NA NA NA NA NA NA ...
##  $ v_138              : int  NA NA NA NA NA NA NA NA NA NA ...
##  $ v_139              : int  NA NA NA NA NA NA NA NA NA NA ...
##  $ v_140              : int  NA NA NA NA NA NA NA NA NA NA ...
##  $ v_141              : int  NA NA NA NA NA NA NA NA NA NA ...
##  $ v_142              : int  NA NA NA NA NA NA NA NA NA NA ...
##  $ v_143              : int  NA NA NA NA NA NA NA NA NA NA ...
##  $ v_144              : int  NA NA NA NA NA NA NA NA NA NA ...
##  $ v_145              : int  NA NA NA NA NA NA NA NA NA NA ...
##  $ v_146              : int  NA NA NA NA NA NA NA NA NA NA ...
##  $ v_147              : int  NA NA NA NA NA NA NA NA NA NA ...
##   [list output truncated]
```

```
data <- rename(data, condition = c_0001, text_order = c_0003,
               METI_target = c_0004)

data$condition <- factor(data$condition)

# Text presented first, 1 = Barth et al., 2 = Faerber et al.
data$text_order <- factor(data$text_order, levels = c(1,2), labels = 
                            c("Barth", "Faerber"))

data$METI_text <- ifelse(data$text_order == "Barth","Faerber","Barth")
data$METI_text <- factor(data$METI_text, levels = c("Barth","Faerber"))

data$summary1 <- data$text_order
data$summary2 <- data$METI_text

data$METI_target <- factor (data$METI_target, levels = c(1,2), labels =
                              c("Study Authors","Summary Authors"))

data <- rename(data, s_sex = v_1, s_age = v_2,
               s_school = v_3, s_german = v_4,
               s_psychology = v_5, s_interest = v_7,
               s_contact = v_8, s_field = v_9)

data$s_sex <- factor(data$s_sex, levels = c (1,2), labels = c("female","male"))
data$s_school <- factor(data$s_school, levels = c(1,2,3), 
                        labels = c("Haupt","Real","Abi"))

data$quota[data$quota == 0] <- NA
data$quota <- factor(data$quota)
```

### Create Factor for Experimental Condition

```
data$version <- case_when(data$condition == 1 ~1,
                          data$condition == 2 ~1,
                          data$condition == 3 ~1,
                          data$condition == 4 ~1,
                          data$condition == 5 ~1,
                          data$condition == 6 ~0)

data$version <- factor(data$version, levels = c(0,1),
                       labels = c("old guideline","new guideline"))
summary(data$version)
```

```
## old guideline new guideline          NA's 
##           498          2492          3715
```

```
data$causality <- case_when(data$condition == 1 ~0,
                            data$condition == 2 ~0,
                            data$condition == 3 ~1,
                            data$condition == 4 ~1,
                            data$condition == 5 ~1,
                            data$condition == 6 ~0)
data$causality <- factor(data$causality, levels = c(0,1),
                         labels = c("no causality statement",
                                    "causality statement"))
summary(data$causality)
```

```
## no causality statement    causality statement                   NA's 
##                   1496                   1494                   3715
```

```
data$disclaimer <- case_when(data$condition == 1 ~0,
                             data$condition == 2 ~1,
                             data$condition == 3 ~0,
                             data$condition == 4 ~1,
                             data$condition == 5 ~1,
                             data$condition == 6 ~0)
data$disclaimer <- factor(data$disclaimer, levels = c(0,1),
                         labels = c("no disclaimer",
                                    "disclaimer"))
summary(data$disclaimer)
```

```
## no disclaimer    disclaimer          NA's 
##          1495          1495          3715
```

```
data$CAMA <- case_when(data$condition == 1 ~0,
                             data$condition == 2 ~0,
                             data$condition == 3 ~0,
                             data$condition == 4 ~0,
                             data$condition == 5 ~1,
                             data$condition == 6 ~0)
data$CAMA <- factor(data$CAMA, levels = c(0,1),
                          labels = c("no CAMA PLS",
                                     "CAMA PLS"))
summary(data$disclaimer)
```

```
## no disclaimer    disclaimer          NA's 
##          1495          1495          3715
```

### Drop Unneeded Dispcodes

```
data <- data[data$dispcode == 22|data$dispcode == 31|data$dispcode == 32,]
length(unique(data$p_0001
              [data$dispcode==22|data$dispcode==31|data$dispcode==32]))
```

```
## [1] 3001
```

## Dropout Analyses

### By Condition

```
data$dropout <- data$dispcode == 22
data$dropout <- factor(data$dropout, c("FALSE","TRUE"),
                       labels = c("No Dropout", "Dropout"))

table(data$dropout, data$condition)
```

```
##             
##                1   2   3   4   5   6
##   No Dropout 334 345 336 341 328 357
##   Dropout    165 154 162 156 170 141
```

```
table(data$dropout,data$condition)[1,]/colSums(table(
  data$dropout,data$condition))*100
```

```
##        1        2        3        4        5        6 
## 66.93387 69.13828 67.46988 68.61167 65.86345 71.68675
```

```
chisq.test(data$dropout, data$condition)
```

```
## 
##  Pearson's Chi-squared test
## 
## data:  data$dropout and data$condition
## X-squared = 4.775, df = 5, p-value = 0.444
```

### By Quota

```
table(data$dropout, data$quota)
```

```
##             
##                1   2   3   4   5   6   7   8   9  10  11  12
##   No Dropout 169 171 174 168 171 172 168 167 164 170 172 175
##   Dropout     88 112 131  93 124 143  33  44  36  63  90  74
```

```
table(data$dropout, data$quota)[1,]/colSums(table(
  data$dropout, data$quota))*100
```

```
##        1        2        3        4        5        6        7        8 
## 65.75875 60.42403 57.04918 64.36782 57.96610 54.60317 83.58209 79.14692 
##        9       10       11       12 
## 82.00000 72.96137 65.64885 70.28112
```

```
chisq.test(data$dropout,data$quota)
```

```
## 
##  Pearson's Chi-squared test
## 
## data:  data$dropout and data$quota
## X-squared = 116.16, df = 11, p-value < 2.2e-16
```

### By Participants’ Gender

```
table(data$dropout, data$s_sex)
```

```
##             
##              female male
##   No Dropout   1028 1013
##   Dropout       587  446
```

```
table(data$dropout, data$s_sex)[1,]/colSums(table(data$dropout, data$s_sex))*100
```

```
##   female     male 
## 63.65325 69.43112
```

```
chisq.test(data$dropout,data$s_sex)
```

```
## 
##  Pearson's Chi-squared test with Yates' continuity correction
## 
## data:  data$dropout and data$s_sex
## X-squared = 11.211, df = 1, p-value = 0.0008129
```

### By Participants’ Age

```
# Set single age value of 744 as NA
data$s_age[data$s_age == 744] <- NA

dropout_age <- glm(data$dropout ~ data$s_age, data = data, family = "binomial",
                   na.action = na.omit)
summary(dropout_age)
```

```
## 
## Call:
## glm(formula = data$dropout ~ data$s_age, family = "binomial", 
##     data = data, na.action = na.omit)
## 
## Coefficients:
##              Estimate Std. Error z value Pr(>|z|)    
## (Intercept) -1.992992   0.130678  -15.25   <2e-16 ***
## data$s_age   0.027108   0.002514   10.78   <2e-16 ***
## ---
## Signif. codes:  0 '***' 0.001 '**' 0.01 '*' 0.05 '.' 0.1 ' ' 1
## 
## (Dispersion parameter for binomial family taken to be 1)
## 
##     Null deviance: 3932.6  on 3076  degrees of freedom
## Residual deviance: 3810.8  on 3075  degrees of freedom
##   (3 Beobachtungen als fehlend gelöscht)
## AIC: 3814.8
## 
## Number of Fisher Scoring iterations: 4
```

```
exp(dropout_age$coefficients)
```

```
## (Intercept)  data$s_age 
##    0.136287    1.027478
```

### By Participants’ Educational Background

```
table(data$dropout, data$s_school)
```

```
##             
##              Haupt Real Abi
##   No Dropout   685  681 675
##   Dropout      387  373 277
```

```
table(data$dropout, data$s_school)[1,]/colSums(
  table(data$dropout, data$s_school))*100
```

```
##    Haupt     Real      Abi 
## 63.89925 64.61101 70.90336
```

```
chisq.test(data$dropout,data$s_school)
```

```
## 
##  Pearson's Chi-squared test
## 
## data:  data$dropout and data$s_school
## X-squared = 13.142, df = 2, p-value = 0.001401
```

```
dropout_edu <- glm(data$dropout ~ data$s_school, data = data, family = "binomial",
                   na.action = na.omit)
summary(dropout_edu)
```

```
## 
## Call:
## glm(formula = data$dropout ~ data$s_school, family = "binomial", 
##     data = data, na.action = na.omit)
## 
## Coefficients:
##                   Estimate Std. Error z value Pr(>|z|)    
## (Intercept)       -0.57099    0.06359  -8.979  < 2e-16 ***
## data$s_schoolReal -0.03099    0.09052  -0.342 0.732076    
## data$s_schoolAbi  -0.31970    0.09558  -3.345 0.000823 ***
## ---
## Signif. codes:  0 '***' 0.001 '**' 0.01 '*' 0.05 '.' 0.1 ' ' 1
## 
## (Dispersion parameter for binomial family taken to be 1)
## 
##     Null deviance: 3933.5  on 3077  degrees of freedom
## Residual deviance: 3920.1  on 3075  degrees of freedom
##   (2 Beobachtungen als fehlend gelöscht)
## AIC: 3926.1
## 
## Number of Fisher Scoring iterations: 4
```

```
exp(dropout_edu$coefficients)
```

```
##       (Intercept) data$s_schoolReal  data$s_schoolAbi 
##         0.5649635         0.9694855         0.7263662
```

### Dropout Regression Analyses

```
dropout_logistic_1 <- glm(formula =  dropout ~ condition + quota, data = data, 
                        family = "binomial", na.action = na.omit)
summary(dropout_logistic_1)
```

```
## 
## Call:
## glm(formula = dropout ~ condition + quota, family = "binomial", 
##     data = data, na.action = na.omit)
## 
## Coefficients:
##              Estimate Std. Error z value Pr(>|z|)    
## (Intercept) -0.653151   0.163055  -4.006 6.18e-05 ***
## condition2  -0.140337   0.138830  -1.011 0.312085    
## condition3  -0.048597   0.138044  -0.352 0.724807    
## condition4  -0.122653   0.138728  -0.884 0.376629    
## condition5   0.007591   0.137135   0.055 0.955854    
## condition6  -0.257050   0.140745  -1.826 0.067798 .  
## quota2       0.220186   0.185468   1.187 0.235151    
## quota3       0.352223   0.180902   1.947 0.051530 .  
## quota4       0.136894   0.188245   0.727 0.467096    
## quota5       0.342825   0.182117   1.882 0.059776 .  
## quota6       0.487074   0.178462   2.729 0.006347 ** 
## quota7      -0.947738   0.238435  -3.975 7.04e-05 ***
## quota8      -0.825807   0.230417  -3.584 0.000338 ***
## quota9      -0.882218   0.236521  -3.730 0.000191 ***
## quota10     -0.334860   0.204088  -1.641 0.100846    
## quota11      0.035250   0.190172   0.185 0.852947    
## quota12     -0.162174   0.195749  -0.828 0.407400    
## ---
## Signif. codes:  0 '***' 0.001 '**' 0.01 '*' 0.05 '.' 0.1 ' ' 1
## 
## (Dispersion parameter for binomial family taken to be 1)
## 
##     Null deviance: 3734.5  on 2988  degrees of freedom
## Residual deviance: 3609.0  on 2972  degrees of freedom
##   (91 Beobachtungen als fehlend gelöscht)
## AIC: 3643
## 
## Number of Fisher Scoring iterations: 4
```

```
exp(dropout_logistic_1$coefficients)
```

```
## (Intercept)  condition2  condition3  condition4  condition5  condition6 
##   0.5204033   0.8690651   0.9525646   0.8845708   1.0076203   0.7733299 
##      quota2      quota3      quota4      quota5      quota6      quota7 
##   1.2463090   1.4222263   1.1467070   1.4089220   1.6275464   0.3876166 
##      quota8      quota9     quota10     quota11     quota12 
##   0.4378814   0.4138641   0.7154382   1.0358787   0.8502934
```

```
dropout_logistic_2 <- glm(formula =  dropout ~ s_sex + s_school + s_age,
                          data = data, family = "binomial", na.action = na.omit)
summary(dropout_logistic_2)
```

```
## 
## Call:
## glm(formula = dropout ~ s_sex + s_school + s_age, family = "binomial", 
##     data = data, na.action = na.omit)
## 
## Coefficients:
##               Estimate Std. Error z value Pr(>|z|)    
## (Intercept)  -1.828571   0.147292 -12.415  < 2e-16 ***
## s_sexmale    -0.346953   0.079185  -4.382 1.18e-05 ***
## s_schoolReal  0.031970   0.092998   0.344   0.7310    
## s_schoolAbi  -0.222797   0.098232  -2.268   0.0233 *  
## s_age         0.028037   0.002556  10.971  < 2e-16 ***
## ---
## Signif. codes:  0 '***' 0.001 '**' 0.01 '*' 0.05 '.' 0.1 ' ' 1
## 
## (Dispersion parameter for binomial family taken to be 1)
## 
##     Null deviance: 3919.6  on 3070  degrees of freedom
## Residual deviance: 3769.0  on 3066  degrees of freedom
##   (9 Beobachtungen als fehlend gelöscht)
## AIC: 3779
## 
## Number of Fisher Scoring iterations: 4
```

```
exp(dropout_logistic_2$coefficients)
```

```
##  (Intercept)    s_sexmale s_schoolReal  s_schoolAbi        s_age 
##    0.1606429    0.7068385    1.0324869    0.8002773    1.0284335
```

## Merging Variables

### Check for NAs in Demographic Variables

```
sum(is.na(data$s_sex))
```

```
## [1] 6
```

```
sum(is.na(data$s_age))
```

```
## [1] 3
```

```
sum(is.na(data$s_school))
```

```
## [1] 2
```

```
sum(is.na(data$s_german))
```

```
## [1] 0
```

```
sum(is.na(data$s_psychology))
```

```
## [1] 0
```

```
sum(is.na(data$s_interest))
```

```
## [1] 0
```

```
sum(is.na(data$s_contact))
```

```
## [1] 20
```

```
sum(is.na(data$s_field))
```

```
## [1] 2466
```

### Merge T1 and T2 Variables

```
data$v_10[data$v_10 == 0] <- NA
data$v_72[data$v_72 == 0] <- NA
data$v_91[data$v_91 == 0] <- NA
data$v_103[data$v_103 == 0] <- NA
data$v_11[data$v_11 == 0] <- NA
data$v_73[data$v_73 == 0] <- NA
data$v_92[data$v_92 == 0] <- NA
data$v_104[data$v_104 == 0] <- NA
data$v_47[data$v_47 == 0] <- NA
data$v_74[data$v_74 == 0] <- NA
data$v_93[data$v_93 == 0] <- NA
data$v_105[data$v_105 == 0] <- NA
data$v_48[data$v_48 == 0] <- NA
data$v_75[data$v_75 == 0] <- NA
data$v_94[data$v_94 == 0] <- NA
data$v_106[data$v_106 == 0] <- NA
data$v_49[data$v_49 == 0] <- NA
data$v_76[data$v_76 == 0] <- NA
data$v_95[data$v_95 == 0] <- NA
data$v_107[data$v_107 == 0] <- NA
data$v_12[data$v_12 == 0] <- NA
data$v_77[data$v_77 == 0] <- NA
data$v_96[data$v_96 == 0] <- NA
data$v_108[data$v_108 == 0] <- NA
data$v_14[data$v_14 == 0] <- NA
data$v_79[data$v_79 == 0] <- NA
data$v_98[data$v_98 == 0] <- NA
data$v_110[data$v_110 == 0] <- NA
data$v_16[data$v_16 == 0] <- NA
data$v_81[data$v_81 == 0] <- NA
data$v_100[data$v_100 == 0] <- NA
data$v_112[data$v_112 == 0] <- NA
data$v_71[data$v_71 == 0] <- NA
data$v_83[data$v_83 == 0] <- NA
data$v_102[data$v_102 == 0] <- NA
data$v_114[data$v_114 == 0] <- NA

data$accessibility_1 <- coalesce(data$v_10, data$v_72)
table(data$accessibility_1)
```

```
## 
##   1   2   3   4   5   6   7   8 
##  52  82 192 353 425 527 409 471
```

```
data$accessibility_2 <- coalesce(data$v_91, data$v_103)
table(data$accessibility_2)
```

```
## 
##   1   2   3   4   5   6   7   8 
##  67  74 177 321 353 393 321 331
```

```
data$understanding_1 <- coalesce(data$v_11, data$v_73)
table(data$understanding_1)
```

```
## 
##   1   2   3   4   5   6   7   8 
##  30  55 151 342 446 576 458 445
```

```
data$understanding_2 <- coalesce(data$v_92, data$v_104)
table(data$understanding_2)
```

```
## 
##   1   2   3   4   5   6   7   8 
##  51  76 160 304 410 459 327 251
```

```
data$empowerment_1 <- coalesce(data$v_47, data$v_74)
table(data$empowerment_1)
```

```
## 
##   1   2   3   4   5   6   7   8 
## 125 137 315 468 566 469 227 190
```

```
data$empowerment_2 <- coalesce(data$v_93, data$v_105)
table(data$empowerment_2)
```

```
## 
##   1   2   3   4   5   6   7   8 
## 131 127 232 410 449 367 182 144
```

```
data$credibility_1 <- coalesce(data$v_48, data$v_75)
table(data$credibility_1)
```

```
## 
##   1   2   3   4   5   6   7   8 
##  12  29  87 344 469 601 487 470
```

```
data$credibility_2 <- coalesce(data$v_94, data$v_106)
table(data$credibility_2)
```

```
## 
##   1   2   3   4   5   6   7   8 
##  26  25  91 296 423 465 372 341
```

```
data$relevance_1 <- coalesce(data$v_49, data$v_76)
table(data$relevance_1)
```

```
## 
##   1   2   3   4   5   6   7   8 
##  17  30  62 231 327 559 466 808
```

```
data$relevance_2 <- coalesce(data$v_95, data$v_107)
table(data$relevance_2)
```

```
## 
##   1   2   3   4   5   6   7   8 
##  32  26  77 204 298 411 397 594
```

```
data$curiosity_1 <- coalesce(data$v_12, data$v_77)
table(data$curiosity_1)
```

```
## 
##   1   2   3   4   5 
## 162 414 773 816 345
```

```
data$curiosity_2 <- coalesce(data$v_96, data$v_108)
table(data$curiosity_2)
```

```
## 
##   1   2   3   4   5 
## 168 420 642 542 273
```

```
data$boredom_1 <- coalesce(data$v_14, data$v_79)
table(data$boredom_1)
```

```
## 
##    1    2    3    4    5 
## 1045  671  542  168   76
```

```
data$boredom_2 <- coalesce(data$v_98, data$v_110)
table(data$boredom_2)
```

```
## 
##   1   2   3   4   5 
## 881 507 424 150  81
```

```
data$confusion_1 <- coalesce(data$v_16, data$v_81)
table(data$confusion_1)
```

```
## 
##   1   2   3   4   5 
## 939 751 598 162  54
```

```
data$confusion_2 <- coalesce(data$v_100, data$v_112)
table(data$confusion_2)
```

```
## 
##   1   2   3   4   5 
## 733 582 493 169  68
```

```
data$frustration_1 <- coalesce(data$v_71, data$v_83)
table(data$frustration_1)
```

```
## 
##    1    2    3    4    5 
## 1486  451  419  107   36
```

```
data$frustration_2 <- coalesce(data$v_102, data$v_114)
table(data$frustration_2)
```

```
## 
##    1    2    3    4    5 
## 1136  392  355  104   58
```

### Merge and Recode Relationship-Item

```
data$v_17[data$v_17 == 0] <- NA
data$v_126[data$v_126 == 0] <- NA
data$v_18[data$v_18 == 0] <- NA
data$v_127[data$v_127 == 0] <- NA
data$v_19[data$v_19 == 0] <- NA
data$v_128[data$v_128 == 0] <- NA
data$v_20[data$v_20 == 0] <- NA
data$v_129[data$v_129 == 0] <- NA
data$v_21[data$v_21 == 0] <- NA
data$v_130[data$v_130 == 0] <- NA
data$v_115[data$v_115 == 0] <- NA
data$v_131[data$v_131 == 0] <- NA
data$v_116[data$v_116 == 0] <- NA
data$v_132[data$v_132 == 0] <- NA
data$v_117[data$v_117 == 0] <- NA
data$v_133[data$v_133 == 0] <- NA

data$s_relationship_1 <- coalesce(data$v_17, data$v_126)
data$s_relationship_2 <- coalesce(data$v_18, data$v_127)
data$s_relationship_3 <- coalesce(data$v_19, data$v_128)
data$s_relationship_4 <- coalesce(data$v_20, data$v_129)
data$s_relationship_5 <- coalesce(data$v_21, data$v_130)
data$s_relationship_6 <- coalesce(data$v_115, data$v_131)
data$s_relationship_7 <- coalesce(data$v_116, data$v_132)
data$s_relationship_8 <- coalesce(data$v_117, data$v_133)

data$s_relationship_1 <- mapvalues(data$s_relationship_1, c(1,2,3), c(1,-1,0))
table(data$s_relationship_1)
```

```
## 
##   -1    0    1 
##  233  345 1690
```

```
data$s_relationship_2 <- mapvalues(data$s_relationship_2, c(1,2,3), c(-1,1,0))
table(data$s_relationship_2)
```

```
## 
##   -1    0    1 
## 1175  406  683
```

```
data$s_relationship_3 <- mapvalues(data$s_relationship_3, c(1,2,3), c(-1,1,0))
table(data$s_relationship_3)
```

```
## 
##  -1   0   1 
## 671 772 822
```

```
data$s_relationship_4 <- mapvalues(data$s_relationship_4, c(1,2,3), c(-1,1,0))
table(data$s_relationship_4)
```

```
## 
##  -1   0   1 
## 730 736 801
```

```
data$s_relationship_5 <- mapvalues(data$s_relationship_5, c(1,2,3), c(1,-1,0))
table(data$s_relationship_5)
```

```
## 
##   -1    0    1 
##  256  375 1638
```

```
data$s_relationship_6 <- mapvalues(data$s_relationship_6, c(1,2,3), c(-1,1,0))
table(data$s_relationship_6)
```

```
## 
##   -1    0    1 
## 1321  426  518
```

```
data$s_relationship_7 <- mapvalues(data$s_relationship_7, c(1,2,3), c(-1,1,0))
table(data$s_relationship_7)
```

```
## 
##   -1    0    1 
## 1349  448  467
```

```
data$s_relationship_8 <- mapvalues(data$s_relationship_8, c(1,2,3), c(-1,1,0))
table(data$s_relationship_8)
```

```
## 
##   -1    0    1 
## 1156  464  642
```

### Merge and Recode Extent of Evaluation-Item

```
data$v_22[data$v_22 == 0] <- NA
data$v_134[data$v_134 == 0] <- NA
data$v_23[data$v_23 == 0] <- NA
data$v_135[data$v_135 == 0] <- NA
data$v_24[data$v_24 == 0] <- NA
data$v_136[data$v_136 == 0] <- NA
data$v_25[data$v_25 == 0] <- NA
data$v_137[data$v_137 == 0] <- NA
data$v_26[data$v_26 == 0] <- NA
data$v_138[data$v_138 == 0] <- NA
data$v_120[data$v_120 == 0] <- NA
data$v_139[data$v_139 == 0] <- NA

data$s_extent_1 <- coalesce(data$v_22, data$v_134)
data$s_extent_2 <- coalesce(data$v_23, data$v_135)
data$s_extent_3 <- coalesce(data$v_24, data$v_136)
data$s_extent_4 <- coalesce(data$v_25, data$v_137)
data$s_extent_5 <- coalesce(data$v_26, data$v_138)
data$s_extent_6 <- coalesce(data$v_120, data$v_139)

data$s_extent_1 <- mapvalues(data$s_extent_1, c(1,2,3), c(-1,1,0))
table(data$s_extent_1)
```

```
## 
##  -1   0   1 
## 663 773 832
```

```
data$s_extent_2 <- mapvalues(data$s_extent_2, c(1,2,3), c(-1,1,0))
table(data$s_extent_2)
```

```
## 
##  -1   0   1 
## 931 585 750
```

```
data$s_extent_3 <- mapvalues(data$s_extent_3, c(1,2,3), c(-1,1,0))
table(data$s_extent_3)
```

```
## 
##   -1    0    1 
## 1035  740  488
```

```
data$s_extent_4 <- mapvalues(data$s_extent_4, c(1,2,3), c(-1,1,0))
table(data$s_extent_4)
```

```
## 
##  -1   0   1 
## 968 763 533
```

```
data$s_extent_5 <- mapvalues(data$s_extent_5, c(1,2,3), c(1,-1,0))
table(data$s_extent_5)
```

```
## 
##   -1    0    1 
##  317  535 1411
```

```
data$s_extent_6 <- mapvalues(data$s_extent_6, c(1,2,3), c(1,-1,0))
table(data$s_extent_6)
```

```
## 
##   -1    0    1 
##  380  497 1393
```

### Merge and Recode Differentiation-Item

```
# Caution: Due to an error (wrong answers provided during experiment), all values for Faerber et al. are NA. Only answers for Barth et al. can be considered for analysis

data$v_27[data$v_27 == 0] <- NA
data$v_28[data$v_28 == 0] <- NA
data$v_29[data$v_29 == 0] <- NA
data$v_30[data$v_30 == 0] <- NA
data$v_31[data$v_31 == 0] <- NA
data$v_121[data$v_121 == 0] <- NA

data$v_140 <- NA
data$v_141 <- NA
data$v_142 <- NA
data$v_143 <- NA
data$v_144 <- NA
data$v_145 <- NA

data$v_235[data$v_235 == 0] <- NA
data$v_236[data$v_236 == 0] <- NA
data$v_237[data$v_237 == 0] <- NA
data$v_238[data$v_238 == 0] <- NA
data$v_239[data$v_239 == 0] <- NA
data$v_240[data$v_240 == 0] <- NA

data$v_274 <- NA
data$v_275 <- NA
data$v_276 <- NA
data$v_277 <- NA
data$v_278 <- NA
data$v_279 <- NA

#Values only need to be mapped for the items from Barth et al.
data$v_27 <- mapvalues(data$v_27, c(1,2,3),c(-1,1,0))
table(data$v_27)
```

```
## 
##  -1   0   1 
## 529 305 305
```

```
data$v_28 <- mapvalues(data$v_28, c(1,2,3),c(-1,1,0))
table(data$v_28)
```

```
## 
##  -1   0   1 
## 561 297 284
```

```
data$v_29 <- mapvalues(data$v_29, c(1,2,3),c(1,-1,0))
table(data$v_29)
```

```
## 
##  -1   0   1 
## 371 321 447
```

```
data$v_30 <- mapvalues(data$v_30, c(1,2,3),c(-1,1,0))
table(data$v_30)
```

```
## 
##  -1   0   1 
## 373 298 467
```

```
data$v_31 <- mapvalues(data$v_31, c(1,2,3),c(1,-1,0))
table(data$v_31)
```

```
## 
##  -1   0   1 
## 262 404 474
```

```
data$v_121 <- mapvalues(data$v_121, c(1,2,3),c(1,-1,0))
table(data$v_121)
```

```
## 
##  -1   0   1 
## 317 343 483
```

```
data$v_235 <- mapvalues(data$v_235, c(1,2,3),c(-1,1,0))
table(data$v_235)
```

```
## 
##  -1   0   1 
## 386 229 397
```

```
data$v_236 <- mapvalues(data$v_236, c(1,2,3),c(-1,1,0))
table(data$v_236)
```

```
## 
##  -1   0   1 
## 415 255 344
```

```
data$v_237 <- mapvalues(data$v_237, c(1,2,3),c(1,-1,0))
table(data$v_237)
```

```
## 
##  -1   0   1 
## 376 255 377
```

```
data$v_238 <- mapvalues(data$v_238, c(1,2,3),c(-1,1,0))
table(data$v_238)
```

```
## 
##  -1   0   1 
## 282 252 481
```

```
data$v_239 <- mapvalues(data$v_239, c(1,2,3),c(1,-1,0))
table(data$v_239)
```

```
## 
##  -1   0   1 
## 259 250 500
```

```
data$v_240 <- mapvalues(data$v_240, c(1,2,3),c(1,-1,0))
table(data$v_240)
```

```
## 
##  -1   0   1 
## 311 255 446
```

```
#Merge for T1
data$s_diff_1_1 <- coalesce(data$v_27, data$v_140)
table(data$s_diff_1_1)
```

```
## 
##  -1   0   1 
## 529 305 305
```

```
data$s_diff_1_2 <- coalesce(data$v_28, data$v_141)
table(data$s_diff_1_2)
```

```
## 
##  -1   0   1 
## 561 297 284
```

```
data$s_diff_1_3 <- coalesce(data$v_29, data$v_142)
table(data$s_diff_1_3)
```

```
## 
##  -1   0   1 
## 371 321 447
```

```
data$s_diff_1_4 <- coalesce(data$v_30, data$v_143)
table(data$s_diff_1_4)
```

```
## 
##  -1   0   1 
## 373 298 467
```

```
data$s_diff_1_5 <- coalesce(data$v_31, data$v_144)
table(data$s_diff_1_5)
```

```
## 
##  -1   0   1 
## 262 404 474
```

```
data$s_diff_1_6 <- coalesce(data$v_121, data$v_145)
table(data$s_diff_1_6)
```

```
## 
##  -1   0   1 
## 317 343 483
```

```
#Merge for T2
data$s_diff_2_1 <- coalesce(data$v_235, data$v_274)
table(data$s_diff_2_1)
```

```
## 
##  -1   0   1 
## 386 229 397
```

```
data$s_diff_2_2 <- coalesce(data$v_236, data$v_275)
table(data$s_diff_2_2)
```

```
## 
##  -1   0   1 
## 415 255 344
```

```
data$s_diff_2_3 <- coalesce(data$v_237, data$v_276)
table(data$s_diff_2_3)
```

```
## 
##  -1   0   1 
## 376 255 377
```

```
data$s_diff_2_4 <- coalesce(data$v_238, data$v_277)
table(data$s_diff_2_4)
```

```
## 
##  -1   0   1 
## 282 252 481
```

```
data$s_diff_2_5 <- coalesce(data$v_239, data$v_278)
table(data$s_diff_2_5)
```

```
## 
##  -1   0   1 
## 259 250 500
```

```
data$s_diff_2_6 <- coalesce(data$v_240, data$v_279)
table(data$s_diff_2_6)
```

```
## 
##  -1   0   1 
## 311 255 446
```

### Merge and Recode Funding-Item

```
data$v_32[data$v_32 == 0] <- NA
data$v_33[data$v_33 == 0] <- NA
data$v_34[data$v_34 == 0] <- NA
data$v_35[data$v_35 == 0] <- NA
data$v_36[data$v_36 == 0] <- NA
data$v_122[data$v_122 == 0] <- NA

data$v_146[data$v_146 == 0] <- NA
data$v_147[data$v_147 == 0] <- NA
data$v_148[data$v_148 == 0] <- NA
data$v_149[data$v_149 == 0] <- NA
data$v_150[data$v_150 == 0] <- NA
data$v_151[data$v_151 == 0] <- NA

data$v_241[data$v_241 == 0] <- NA
data$v_242[data$v_242 == 0] <- NA
data$v_243[data$v_243 == 0] <- NA
data$v_244[data$v_244 == 0] <- NA
data$v_245[data$v_245 == 0] <- NA
data$v_246[data$v_246 == 0] <- NA

data$v_280[data$v_280 == 0] <- NA
data$v_281[data$v_281 == 0] <- NA
data$v_282[data$v_282 == 0] <- NA
data$v_283[data$v_283 == 0] <- NA
data$v_284[data$v_284 == 0] <- NA
data$v_285[data$v_285 == 0] <- NA

data$v_32 <- mapvalues(data$v_32, c(1,2,3), c(-1,1,0))
table(data$v_32)
```

```
## 
##  -1   0   1 
## 363 418 357
```

```
data$v_33 <- mapvalues(data$v_33, c(1,2,3), c(-1,1,0))
table(data$v_33)
```

```
## 
##  -1   0   1 
## 335 406 401
```

```
data$v_34 <- mapvalues(data$v_34, c(1,2,3), c(-1,1,0))
table(data$v_34)
```

```
## 
##  -1   0   1 
## 283 333 524
```

```
data$v_35 <- mapvalues(data$v_35, c(1,2,3), c(-1,1,0))
table(data$v_35)
```

```
## 
##  -1   0   1 
## 316 452 374
```

```
data$v_36 <- mapvalues(data$v_36, c(1,2,3), c(-1,1,0))
table(data$v_36)
```

```
## 
##  -1   0   1 
## 341 434 367
```

```
data$v_122 <- mapvalues(data$v_122, c(1,2,3), c(1,-1,0))
table(data$v_122)
```

```
## 
##  -1   0   1 
## 240 400 501
```

```
data$v_146 <- mapvalues(data$v_146, c(1,2,3), c(-1,1,0))
table(data$v_122)
```

```
## 
##  -1   0   1 
## 240 400 501
```

```
data$v_147 <- mapvalues(data$v_147, c(1,2,3), c(-1,1,0))
table(data$v_147)
```

```
## 
##  -1   0   1 
## 311 396 420
```

```
data$v_148 <- mapvalues(data$v_148, c(1,2,3), c(-1,1,0))
table(data$v_148)
```

```
## 
##  -1   0   1 
## 200 443 480
```

```
data$v_149 <- mapvalues(data$v_149, c(1,2,3), c(1,-1,0))
table(data$v_149)
```

```
## 
##  -1   0   1 
## 241 324 559
```

```
data$v_150 <- mapvalues(data$v_150, c(1,2,3), c(-1,1,0))
table(data$v_150)
```

```
## 
##  -1   0   1 
## 226 437 460
```

```
data$v_151 <- mapvalues(data$v_151, c(1,2,3), c(-1,1,0))
table(data$v_151)
```

```
## 
##  -1   0   1 
## 249 405 465
```

```
data$v_241 <- mapvalues(data$v_241, c(1,2,3), c(-1,1,0))
table(data$v_241)
```

```
## 
##  -1   0   1 
## 223 257 533
```

```
data$v_242 <- mapvalues(data$v_242, c(1,2,3), c(-1,1,0))
table(data$v_242)
```

```
## 
##  -1   0   1 
## 227 277 508
```

```
data$v_243 <- mapvalues(data$v_243, c(1,2,3), c(-1,1,0))
table(data$v_243)
```

```
## 
##  -1   0   1 
## 193 234 590
```

```
data$v_244 <- mapvalues(data$v_244, c(1,2,3), c(-1,1,0))
table(data$v_244)
```

```
## 
##  -1   0   1 
## 310 274 431
```

```
data$v_245 <- mapvalues(data$v_245, c(1,2,3), c(-1,1,0))
table(data$v_245)
```

```
## 
##  -1   0   1 
## 307 257 450
```

```
data$v_246 <- mapvalues(data$v_246, c(1,2,3), c(1,-1,0))
table(data$v_246)
```

```
## 
##  -1   0   1 
## 192 240 585
```

```
data$v_280 <- mapvalues(data$v_280, c(1,2,3), c(-1,1,0))
table(data$v_280)
```

```
## 
##  -1   0   1 
## 263 276 487
```

```
data$v_281 <- mapvalues(data$v_281, c(1,2,3), c(-1,1,0))
table(data$v_281)
```

```
## 
##  -1   0   1 
## 239 279 507
```

```
data$v_282 <- mapvalues(data$v_282, c(1,2,3), c(-1,1,0))
table(data$v_282)
```

```
## 
##  -1   0   1 
## 212 307 506
```

```
data$v_283 <- mapvalues(data$v_283, c(1,2,3), c(1,-1,0))
table(data$v_283)
```

```
## 
##  -1   0   1 
## 197 259 572
```

```
data$v_284 <- mapvalues(data$v_284, c(1,2,3), c(-1,1,0))
table(data$v_284)
```

```
## 
##  -1   0   1 
## 230 296 501
```

```
data$v_285 <- mapvalues(data$v_285, c(1,2,3), c(-1,1,0))
table(data$v_285)
```

```
## 
##  -1   0   1 
## 185 311 527
```

```
# Merge for T1
data$s_funding_1_1 <- coalesce(data$v_32, data$v_146)
table(data$s_funding_1_1)
```

```
## 
##  -1   0   1 
## 716 797 749
```

```
data$s_funding_1_2 <- coalesce(data$v_33, data$v_147)
table(data$s_funding_1_2)
```

```
## 
##  -1   0   1 
## 646 802 821
```

```
data$s_funding_1_3 <- coalesce(data$v_34, data$v_148)
table(data$s_funding_1_3)
```

```
## 
##   -1    0    1 
##  483  776 1004
```

```
data$s_funding_1_4 <- coalesce(data$v_35, data$v_149)
table(data$s_funding_1_4)
```

```
## 
##  -1   0   1 
## 557 776 933
```

```
data$s_funding_1_5 <- coalesce(data$v_36, data$v_150)
table(data$s_funding_1_5)
```

```
## 
##  -1   0   1 
## 567 871 827
```

```
data$s_funding_1_6 <- coalesce(data$v_122, data$v_151)
table(data$s_funding_1_6)
```

```
## 
##  -1   0   1 
## 489 805 966
```

```
# Merge for T2
data$s_funding_2_1 <- coalesce(data$v_241, data$v_280)
table(data$s_funding_2_1)
```

```
## 
##   -1    0    1 
##  486  533 1020
```

```
data$s_funding_2_2 <- coalesce(data$v_242, data$v_281)
table(data$s_funding_2_2)
```

```
## 
##   -1    0    1 
##  466  556 1015
```

```
data$s_funding_2_3 <- coalesce(data$v_243, data$v_282)
table(data$s_funding_2_3)
```

```
## 
##   -1    0    1 
##  405  541 1096
```

```
data$s_funding_2_4 <- coalesce(data$v_244, data$v_283)
table(data$s_funding_2_4)
```

```
## 
##   -1    0    1 
##  507  533 1003
```

```
data$s_funding_2_5 <- coalesce(data$v_245, data$v_284)
table(data$s_funding_2_5)
```

```
## 
##  -1   0   1 
## 537 553 951
```

```
data$s_funding_2_6 <- coalesce(data$v_246, data$v_285)
table(data$s_funding_2_6)
```

```
## 
##   -1    0    1 
##  377  551 1112
```

### Merge and Recode COI-Item

```
data$v_37[data$v_37 == 0] <- NA
data$v_38[data$v_38 == 0] <- NA
data$v_39[data$v_39 == 0] <- NA
data$v_40[data$v_40 == 0] <- NA
data$v_41[data$v_41 == 0] <- NA
data$v_123[data$v_123 == 0] <- NA
data$v_124[data$v_124 == 0] <- NA

data$v_152[data$v_152 == 0] <- NA
data$v_153[data$v_153 == 0] <- NA
data$v_154[data$v_154 == 0] <- NA
data$v_155[data$v_155 == 0] <- NA
data$v_156[data$v_156 == 0] <- NA
data$v_157[data$v_157 == 0] <- NA
data$v_158[data$v_158 == 0] <- NA

data$v_247[data$v_247 == 0] <- NA
data$v_248[data$v_248 == 0] <- NA
data$v_249[data$v_249 == 0] <- NA
data$v_250[data$v_250 == 0] <- NA
data$v_251[data$v_251 == 0] <- NA
data$v_252[data$v_252 == 0] <- NA
data$v_253[data$v_253 == 0] <- NA

data$v_286[data$v_286 == 0] <- NA
data$v_287[data$v_287 == 0] <- NA
data$v_288[data$v_288 == 0] <- NA
data$v_289[data$v_289 == 0] <- NA
data$v_290[data$v_290 == 0] <- NA
data$v_291[data$v_291 == 0] <- NA
data$v_292[data$v_292 == 0] <- NA

data$v_37 <- mapvalues(data$v_37,c(1,2,3),c(-1,1,0))
table(data$v_37)
```

```
## 
##  -1   0   1 
## 360 388 393
```

```
data$v_38 <- mapvalues(data$v_38,c(1,2,3),c(-1,1,0))
table(data$v_38)
```

```
## 
##  -1   0   1 
## 356 360 417
```

```
data$v_39 <- mapvalues(data$v_39,c(1,2,3),c(-1,1,0))
table(data$v_39)
```

```
## 
##  -1   0   1 
## 328 386 414
```

```
data$v_40 <- mapvalues(data$v_40,c(1,2,3),c(-1,1,0))
table(data$v_40)
```

```
## 
##  -1   0   1 
## 385 389 367
```

```
data$v_41 <- mapvalues(data$v_41,c(1,2,3),c(-1,1,0))
table(data$v_41)
```

```
## 
##  -1   0   1 
## 309 388 437
```

```
data$v_123 <- mapvalues(data$v_123,c(1,2,3),c(1,-1,0))
table(data$v_42)
```

```
## 
##   0   1   2   3 
##   9 542 339 257
```

```
data$v_124 <- mapvalues(data$v_124,c(1,2,3),c(-1,1,0))
table(data$v_124)
```

```
## 
##  -1   0   1 
## 345 395 397
```

```
data$v_152 <- mapvalues(data$v_152,c(1,2,3),c(-1,1,0))
table(data$v_152)
```

```
## 
##  -1   0   1 
## 306 371 446
```

```
data$v_153 <- mapvalues(data$v_153,c(1,2,3),c(-1,1,0))
table(data$v_153)
```

```
## 
##  -1   0   1 
## 270 380 469
```

```
data$v_154 <- mapvalues(data$v_154,c(1,2,3),c(-1,1,0))
table(data$v_154)
```

```
## 
##  -1   0   1 
## 302 366 452
```

```
data$v_155 <- mapvalues(data$v_155,c(1,2,3),c(-1,1,0))
table(data$v_155)
```

```
## 
##  -1   0   1 
## 317 379 428
```

```
data$v_156 <- mapvalues(data$v_156,c(1,2,3),c(-1,1,0))
table(data$v_156)
```

```
## 
##  -1   0   1 
## 265 375 482
```

```
data$v_157 <- mapvalues(data$v_157,c(1,2,3),c(-1,1,0))
table(data$v_157)
```

```
## 
##  -1   0   1 
## 276 378 467
```

```
data$v_158 <- mapvalues(data$v_158,c(1,2,3),c(1,-1,0))
table(data$v_158)
```

```
## 
##  -1   0   1 
## 293 377 453
```

```
data$v_247 <- mapvalues(data$v_247,c(1,2,3),c(-1,1,0))
table(data$v_247)
```

```
## 
##  -1   0   1 
## 316 265 436
```

```
data$v_248 <- mapvalues(data$v_248,c(1,2,3),c(-1,1,0))
table(data$v_248)
```

```
## 
##  -1   0   1 
## 276 287 451
```

```
data$v_249 <- mapvalues(data$v_249,c(1,2,3),c(-1,1,0))
table(data$v_249)
```

```
## 
##  -1   0   1 
## 269 293 452
```

```
data$v_250 <- mapvalues(data$v_250,c(1,2,3),c(-1,1,0))
table(data$v_250)
```

```
## 
##  -1   0   1 
## 312 301 403
```

```
data$v_251 <- mapvalues(data$v_251,c(1,2,3),c(-1,1,0))
table(data$v_251)
```

```
## 
##  -1   0   1 
## 261 300 455
```

```
data$v_252 <- mapvalues(data$v_252,c(1,2,3),c(1,-1,0))
table(data$v_252)
```

```
## 
##  -1   0   1 
## 375 301 336
```

```
data$v_253 <- mapvalues(data$v_253,c(1,2,3),c(-1,1,0))
table(data$v_253)
```

```
## 
##  -1   0   1 
## 255 307 453
```

```
data$v_286 <- mapvalues(data$v_286,c(1,2,3),c(-1,1,0))
table(data$v_286)
```

```
## 
##  -1   0   1 
## 246 270 511
```

```
data$v_287 <- mapvalues(data$v_287,c(1,2,3),c(-1,1,0))
table(data$v_287)
```

```
## 
##  -1   0   1 
## 260 284 482
```

```
data$v_288 <- mapvalues(data$v_288,c(1,2,3),c(-1,1,0))
table(data$v_288)
```

```
## 
##  -1   0   1 
## 247 260 518
```

```
data$v_289 <- mapvalues(data$v_289,c(1,2,3),c(-1,1,0))
table(data$v_289)
```

```
## 
##  -1   0   1 
## 238 283 505
```

```
data$v_290 <- mapvalues(data$v_290,c(1,2,3),c(-1,1,0))
table(data$v_290)
```

```
## 
##  -1   0   1 
## 241 272 513
```

```
data$v_291 <- mapvalues(data$v_291,c(1,2,3),c(-1,1,0))
table(data$v_291)
```

```
## 
##  -1   0   1 
## 231 282 513
```

```
data$v_292 <- mapvalues(data$v_292,c(1,2,3),c(1,-1,0))
table(data$v_292)
```

```
## 
##  -1   0   1 
## 316 266 443
```

```
# Merge for T1
data$s_coi_1_1 <- coalesce(data$v_37, data$v_152)
table(data$s_coi_1_1)
```

```
## 
##  -1   0   1 
## 666 759 839
```

```
data$s_coi_1_2 <- coalesce(data$v_38, data$v_153)
table(data$s_coi_1_2)
```

```
## 
##  -1   0   1 
## 626 740 886
```

```
data$s_coi_1_3 <- coalesce(data$v_39, data$v_154)
table(data$s_coi_1_3)
```

```
## 
##  -1   0   1 
## 630 752 866
```

```
data$s_coi_1_4 <- coalesce(data$v_40, data$v_155)
table(data$s_coi_1_4)
```

```
## 
##  -1   0   1 
## 702 768 795
```

```
data$s_coi_1_5 <- coalesce(data$v_41, data$v_156)
table(data$s_coi_1_5)
```

```
## 
##  -1   0   1 
## 574 763 919
```

```
data$s_coi_1_6 <- coalesce(data$v_123, data$v_157)
table(data$s_coi_1_6)
```

```
## 
##  -1   0   1 
## 644 765 854
```

```
data$s_coi_1_7 <- coalesce(data$v_124, data$v_158)
table(data$s_coi_1_7)
```

```
## 
##  -1   0   1 
## 638 772 850
```

```
# Merge for T2
data$s_coi_2_1 <- coalesce(data$v_247, data$v_286)
table(data$s_coi_2_1)
```

```
## 
##  -1   0   1 
## 562 535 947
```

```
data$s_coi_2_2 <- coalesce(data$v_248, data$v_287)
table(data$s_coi_2_2)
```

```
## 
##  -1   0   1 
## 536 571 933
```

```
data$s_coi_2_3 <- coalesce(data$v_249, data$v_288)
table(data$s_coi_2_3)
```

```
## 
##  -1   0   1 
## 516 553 970
```

```
data$s_coi_2_4 <- coalesce(data$v_250, data$v_289)
table(data$s_coi_2_4)
```

```
## 
##  -1   0   1 
## 550 584 908
```

```
data$s_coi_2_5 <- coalesce(data$v_251, data$v_290)
table(data$s_coi_2_5)
```

```
## 
##  -1   0   1 
## 502 572 968
```

```
data$s_coi_2_6 <- coalesce(data$v_252, data$v_291)
table(data$s_coi_2_6)
```

```
## 
##  -1   0   1 
## 606 583 849
```

```
data$s_coi_2_7 <- coalesce(data$v_253, data$v_292)
table(data$s_coi_2_7)
```

```
## 
##  -1   0   1 
## 571 573 896
```

### Merge and Recode Causality-Item

```
data$v_42[data$v_42 == 0] <- NA
data$v_43[data$v_43 == 0] <- NA
data$v_44[data$v_44 == 0] <- NA
data$v_45[data$v_45 == 0] <- NA
data$v_46[data$v_46 == 0] <- NA
data$v_125[data$v_125 == 0] <- NA

data$v_159[data$v_159 == 0] <- NA
data$v_160[data$v_160 == 0] <- NA
data$v_161[data$v_161 == 0] <- NA
data$v_162[data$v_162 == 0] <- NA
data$v_163[data$v_163 == 0] <- NA
data$v_164[data$v_164 == 0] <- NA

data$v_254[data$v_254 == 0] <- NA
data$v_255[data$v_255 == 0] <- NA
data$v_256[data$v_256 == 0] <- NA
data$v_257[data$v_257 == 0] <- NA
data$v_258[data$v_258 == 0] <- NA
data$v_259[data$v_259 == 0] <- NA

data$v_293[data$v_293 == 0] <- NA
data$v_294[data$v_294 == 0] <- NA
data$v_295[data$v_295 == 0] <- NA
data$v_296[data$v_296 == 0] <- NA
data$v_297[data$v_297 == 0] <- NA
data$v_298[data$v_298 == 0] <- NA

data$v_42 <- mapvalues(data$v_42, c(1,2,3), c(1,-1,0))
table(data$v_42)
```

```
## 
##  -1   0   1 
## 339 257 542
```

```
data$v_43 <- mapvalues(data$v_43, c(1,2,3), c(-1,1,0))
table(data$v_43)
```

```
## 
##  -1   0   1 
## 512 273 358
```

```
data$v_44 <- mapvalues(data$v_44, c(1,2,3), c(-1,1,0))
table(data$v_44)
```

```
## 
##  -1   0   1 
## 406 373 361
```

```
data$v_45 <- mapvalues(data$v_45, c(1,2,3), c(-1,1,0))
table(data$v_45)
```

```
## 
##  -1   0   1 
## 423 382 337
```

```
data$v_46 <- mapvalues(data$v_46, c(1,2,3), c(-1,1,0))
table(data$v_46)
```

```
## 
##  -1   0   1 
## 476 313 353
```

```
data$v_125 <- mapvalues(data$v_125, c(1,2,3), c(-1,1,0))
table(data$v_125)
```

```
## 
##  -1   0   1 
## 505 342 294
```

```
data$v_159 <- mapvalues(data$v_159, c(1,2,3), c(1,-1,0))
table(data$v_159)
```

```
## 
##  -1   0   1 
## 115 186 824
```

```
data$v_160 <- mapvalues(data$v_160, c(1,2,3), c(-1,1,0))
table(data$v_160)
```

```
## 
##  -1   0   1 
## 555 266 301
```

```
data$v_161 <- mapvalues(data$v_161, c(1,2,3), c(-1,1,0))
table(data$v_161)
```

```
## 
##  -1   0   1 
## 642 224 257
```

```
data$v_162 <- mapvalues(data$v_162, c(1,2,3), c(-1,1,0))
table(data$v_162)
```

```
## 
##  -1   0   1 
## 686 217 219
```

```
data$v_163 <- mapvalues(data$v_163, c(1,2,3), c(-1,1,0))
table(data$v_163)
```

```
## 
##  -1   0   1 
## 455 314 352
```

```
data$v_164 <- mapvalues(data$v_164, c(1,2,3), c(-1,1,0))
table(data$v_164)
```

```
## 
##  -1   0   1 
## 438 314 371
```

```
data$v_254 <- mapvalues(data$v_254, c(1,2,3), c(1,-1,0))
table(data$v_254)
```

```
## 
##  -1   0   1 
## 305 234 475
```

```
data$v_255 <- mapvalues(data$v_255, c(1,2,3), c(-1,1,0))
table(data$v_255)
```

```
## 
##  -1   0   1 
## 416 255 341
```

```
data$v_256 <- mapvalues(data$v_256, c(1,2,3), c(-1,1,0))
table(data$v_256)
```

```
## 
##  -1   0   1 
## 347 330 339
```

```
data$v_257 <- mapvalues(data$v_257, c(1,2,3), c(-1,1,0))
table(data$v_257)
```

```
## 
##  -1   0   1 
## 341 344 323
```

```
data$v_258 <- mapvalues(data$v_258, c(1,2,3), c(-1,1,0))
table(data$v_258)
```

```
## 
##  -1   0   1 
## 392 295 328
```

```
data$v_259 <- mapvalues(data$v_259, c(1,2,3), c(-1,1,0))
table(data$v_259)
```

```
## 
##  -1   0   1 
## 392 293 330
```

```
data$v_293 <- mapvalues(data$v_293, c(1,2,3), c(1,-1,0))
table(data$v_293)
```

```
## 
##  -1   0   1 
## 124 204 698
```

```
data$v_294 <- mapvalues(data$v_294, c(1,2,3), c(-1,1,0))
table(data$v_294)
```

```
## 
##  -1   0   1 
## 436 295 292
```

```
data$v_295 <- mapvalues(data$v_295, c(1,2,3), c(-1,1,0))
table(data$v_295)
```

```
## 
##  -1   0   1 
## 535 231 261
```

```
data$v_296 <- mapvalues(data$v_296, c(1,2,3), c(-1,1,0))
table(data$v_296)
```

```
## 
##  -1   0   1 
## 597 240 185
```

```
data$v_297 <- mapvalues(data$v_297, c(1,2,3), c(-1,1,0))
table(data$v_297)
```

```
## 
##  -1   0   1 
## 384 311 331
```

```
data$v_298 <- mapvalues(data$v_298, c(1,2,3), c(-1,1,0))
table(data$v_298)
```

```
## 
##  -1   0   1 
## 383 312 328
```

```
# Merge for T1
data$s_causality_1_1 <- coalesce(data$v_42, data$v_159)
table(data$s_causality_1_1)
```

```
## 
##   -1    0    1 
##  454  443 1366
```

```
data$s_causality_1_2 <- coalesce(data$v_43, data$v_160)
table(data$s_causality_1_2)
```

```
## 
##   -1    0    1 
## 1067  539  659
```

```
data$s_causality_1_3 <- coalesce(data$v_44, data$v_161)
table(data$s_causality_1_3)
```

```
## 
##   -1    0    1 
## 1048  597  618
```

```
data$s_causality_1_4 <- coalesce(data$v_45, data$v_162)
table(data$s_causality_1_4)
```

```
## 
##   -1    0    1 
## 1109  599  556
```

```
data$s_causality_1_5 <- coalesce(data$v_46, data$v_163)
table(data$s_causality_1_5)
```

```
## 
##  -1   0   1 
## 931 627 705
```

```
data$s_causality_1_6 <- coalesce(data$v_125, data$v_164)
table(data$s_causality_1_6)
```

```
## 
##  -1   0   1 
## 943 656 665
```

```
# Merge for T2
data$s_causality_2_1 <- coalesce(data$v_254, data$v_293)
table(data$s_causality_2_1)
```

```
## 
##   -1    0    1 
##  429  438 1173
```

```
data$s_causality_2_2 <- coalesce(data$v_255, data$v_294)
table(data$s_causality_2_2)
```

```
## 
##  -1   0   1 
## 852 550 633
```

```
data$s_causality_2_3 <- coalesce(data$v_256, data$v_295)
table(data$s_causality_2_3)
```

```
## 
##  -1   0   1 
## 882 561 600
```

```
data$s_causality_2_4 <- coalesce(data$v_257, data$v_296)
table(data$s_causality_2_4)
```

```
## 
##  -1   0   1 
## 938 584 508
```

```
data$s_causality_2_5 <- coalesce(data$v_258, data$v_297)
table(data$s_causality_2_5)
```

```
## 
##  -1   0   1 
## 776 606 659
```

```
data$s_causality_2_6 <- coalesce(data$v_259, data$v_298)
table(data$s_causality_2_6)
```

```
## 
##  -1   0   1 
## 775 605 658
```

### Merge and Recode CAMA-Items

```
data$v_50[data$v_50 == 0] <- NA
data$v_51[data$v_51 == 0] <- NA
data$v_52[data$v_52 == 0] <- NA
data$v_53[data$v_53 == 0] <- NA
data$v_54[data$v_54 == 0] <- NA
data$v_165[data$v_165 == 0] <- NA
data$v_166[data$v_166 == 0] <- NA
data$v_167[data$v_167 == 0] <- NA

data$v_55[data$v_55 == 0] <- NA
data$v_56[data$v_56 == 0] <- NA
data$v_57[data$v_57 == 0] <- NA
data$v_58[data$v_58 == 0] <- NA

data$v_401[data$v_401 == 0] <- NA

data$v_299[data$v_299 == 0] <- NA
data$v_300[data$v_300 == 0] <- NA
data$v_301[data$v_301 == 0] <- NA
data$v_302[data$v_302 == 0] <- NA
data$v_303[data$v_303 == 0] <- NA
data$v_304[data$v_304 == 0] <- NA
data$v_305[data$v_305 == 0] <- NA
data$v_306[data$v_306 == 0] <- NA

data$v_307[data$v_307 == 0] <- NA
data$v_308[data$v_308 == 0] <- NA
data$v_309[data$v_309 == 0] <- NA
data$v_310[data$v_310 == 0] <- NA

data$v_402[data$v_402 == 0] <- NA

# Caution: For v_401 and v_402, coding is dependent on condition. Items is correct in condition 5, incorrect in conditions 4 and 6.

data$v_50 <- mapvalues(data$v_50, c(1,2,3), c(1,-1,0))
table(data$v_50)
```

```
## 
##  -1   0   1 
##  65 179 284
```

```
data$v_51 <- mapvalues(data$v_51, c(1,2,3), c(-1,1,0))
table(data$v_51)
```

```
## 
##  -1   0   1 
## 129 188 211
```

```
data$v_52 <- mapvalues(data$v_52, c(1,2,3), c(-1,1,0))
table(data$v_52)
```

```
## 
##  -1   0   1 
## 203 217 111
```

```
data$v_53 <- mapvalues(data$v_53, c(1,2,3), c(-1,1,0))
table(data$v_53)
```

```
## 
##  -1   0   1 
## 221 204 100
```

```
data$v_54 <- mapvalues(data$v_54, c(1,2,3), c(1,-1,0))
table(data$v_54)
```

```
## 
##  -1   0   1 
##  81 246 202
```

```
data$v_165 <- mapvalues(data$v_165, c(1,2,3), c(-1,1,0))
table(data$v_165)
```

```
## 
##  -1   0   1 
## 130 297 102
```

```
data$v_166 <- mapvalues(data$v_166, c(1,2,3), c(-1,1,0))
table(data$v_166)
```

```
## 
##  -1   0   1 
##  99 225 206
```

```
data$v_167 <- mapvalues(data$v_167, c(1,2,3), c(-1,1,0))
table(data$v_167)
```

```
## 
##  -1   0   1 
##  98 282 150
```

```
data$v_55 <- mapvalues(data$v_55, c(1,2,3), c(-1,1,0))
table(data$v_55)
```

```
## 
##  -1   0   1 
## 256 191  83
```

```
data$v_56 <- mapvalues(data$v_56, c(1,2,3), c(1,-1,0))
table(data$v_56)
```

```
## 
##  -1   0   1 
## 120 243 166
```

```
data$v_57 <- mapvalues(data$v_57, c(1,2,3), c(-1,1,0))
table(data$v_57)
```

```
## 
##  -1   0   1 
## 155 238 138
```

```
data$v_58 <- mapvalues(data$v_58, c(1,2,3), c(-1,1,0))
table(data$v_58)
```

```
## 
##  -1   0   1 
## 171 226 133
```

```
data$v_401_n <- NA
data$v_401_n <- ifelse(data$condition == 4 & data$v_401 == 1, -1, data$v_401_n)
data$v_401_n <- ifelse(data$condition == 4 & data$v_401 == 2, 1, data$v_401_n)
data$v_401_n <- ifelse(data$condition == 4 & data$v_401 == 3, 0, data$v_401_n)
data$v_401_n <- ifelse(data$condition == 5 & data$v_401 == 1, 1, data$v_401_n)
data$v_401_n <- ifelse(data$condition == 5 & data$v_401 == 2, -1, data$v_401_n)
data$v_401_n <- ifelse(data$condition == 5 & data$v_401 == 3, 0, data$v_401_n)
data$v_401_n <- ifelse(data$condition == 6 & data$v_401 == 1, -1, data$v_401_n)
data$v_401_n <- ifelse(data$condition == 6 & data$v_401 == 2, 1, data$v_401_n)
data$v_401_n <- ifelse(data$condition == 6 & data$v_401 == 3, 0, data$v_401_n)
table(data$v_401_n)
```

```
## 
##  -1   0   1 
## 176 193 163
```

```
data$v_299 <- mapvalues(data$v_299, c(1,2,3), c(1,-1,0))
table(data$v_299)
```

```
## 
##  -1   0   1 
##  88 177 263
```

```
data$v_300 <- mapvalues(data$v_300, c(1,2,3), c(-1,1,0))
table(data$v_300)
```

```
## 
##  -1   0   1 
## 116 209 202
```

```
data$v_301 <- mapvalues(data$v_301, c(1,2,3), c(-1,1,0))
table(data$v_301)
```

```
## 
##  -1   0   1 
## 181 229 118
```

```
data$v_302 <- mapvalues(data$v_302, c(1,2,3), c(-1,1,0))
table(data$v_302)
```

```
## 
##  -1   0   1 
## 185 217 126
```

```
data$v_303 <- mapvalues(data$v_303, c(1,2,3), c(1,-1,0))
table(data$v_303)
```

```
## 
##  -1   0   1 
##  85 238 203
```

```
data$v_304 <- mapvalues(data$v_304, c(1,2,3), c(-1,1,0))
table(data$v_304)
```

```
## 
##  -1   0   1 
## 134 266 122
```

```
data$v_305 <- mapvalues(data$v_305, c(1,2,3), c(-1,1,0))
table(data$v_305)
```

```
## 
##  -1   0   1 
## 108 208 210
```

```
data$v_306 <- mapvalues(data$v_306, c(1,2,3), c(-1,1,0))
table(data$v_306)
```

```
## 
##  -1   0   1 
## 113 256 159
```

```
data$v_307 <- mapvalues(data$v_307, c(1,2,3), c(-1,1,0))
table(data$v_307)
```

```
## 
##  -1   0   1 
## 211 170 147
```

```
data$v_308 <- mapvalues(data$v_308, c(1,2,3), c(1,-1,0))
table(data$v_308)
```

```
## 
##  -1   0   1 
## 143 219 166
```

```
data$v_309 <- mapvalues(data$v_309, c(1,2,3), c(-1,1,0))
table(data$v_309)
```

```
## 
##  -1   0   1 
## 170 213 145
```

```
data$v_310 <- mapvalues(data$v_310, c(1,2,3), c(-1,1,0))
table(data$v_310)
```

```
## 
##  -1   0   1 
## 193 201 135
```

```
data$v_402_n <- NA
data$v_402_n <- ifelse(data$condition == 4 & data$v_402 == 1, -1, data$v_402_n)
data$v_402_n <- ifelse(data$condition == 4 & data$v_402 == 2, 1, data$v_402_n)
data$v_402_n <- ifelse(data$condition == 4 & data$v_402 == 3, 0, data$v_402_n)
data$v_402_n <- ifelse(data$condition == 5 & data$v_402 == 1, 1, data$v_402_n)
data$v_402_n <- ifelse(data$condition == 5 & data$v_402 == 2, -1, data$v_402_n)
data$v_402_n <- ifelse(data$condition == 5 & data$v_402 == 3, 0, data$v_402_n)
data$v_402_n <- ifelse(data$condition == 6 & data$v_402 == 1, -1, data$v_402_n)
data$v_402_n <- ifelse(data$condition == 6 & data$v_402 == 2, 1, data$v_402_n)
data$v_402_n <- ifelse(data$condition == 6 & data$v_402 == 3, 0, data$v_402_n)
table(data$v_402_n)
```

```
## 
##  -1   0   1 
## 141 217 172
```

```
data <- rename(data, s_CAMA_1_1_1 = v_50, s_CAMA_1_1_2 = v_51, s_CAMA_1_1_3 =
                 v_52, s_CAMA_1_1_4 = v_53, s_CAMA_1_1_5 = v_54, s_CAMA_1_1_6 =
                 v_165, s_CAMA_1_1_7 = v_166, s_CAMA_1_1_8 = v_167, 
               s_CAMA_1_2_1 = v_55, s_CAMA_1_2_2 = v_56, s_CAMA_1_2_3 = v_57,
               s_CAMA_1_2_4 = v_58, s_CAMA_1_3 = v_401_n, s_CAMA_2_1_1 = v_299,
               s_CAMA_2_1_2 = v_300, s_CAMA_2_1_3 = v_301, s_CAMA_2_1_4 = v_302,
               s_CAMA_2_1_5 = v_303, s_CAMA_2_1_6 = v_304, s_CAMA_2_1_7 = v_305,
               s_CAMA_2_1_8 = v_306, s_CAMA_2_2_1 = v_307, s_CAMA_2_2_2 = v_308,
               s_CAMA_2_2_3 = v_309, s_CAMA_2_2_4 = v_310, s_CAMA_2_3 = v_402_n)

data$s_CAMA_1_1 <- coalesce(data$s_CAMA_1_1_1, data$s_CAMA_2_1_1)
table(data$s_CAMA_1_1)
```

```
## 
##  -1   0   1 
## 153 356 547
```

```
data$s_CAMA_1_2 <- coalesce(data$s_CAMA_1_1_2, data$s_CAMA_2_1_2)
table(data$s_CAMA_1_2)
```

```
## 
##  -1   0   1 
## 245 397 413
```

```
data$s_CAMA_1_3 <- coalesce(data$s_CAMA_1_1_3, data$s_CAMA_2_1_3)
table(data$s_CAMA_1_3)
```

```
## 
##  -1   0   1 
## 384 446 229
```

```
data$s_CAMA_1_4 <- coalesce(data$s_CAMA_1_1_4, data$s_CAMA_2_1_4)
table(data$s_CAMA_1_4)
```

```
## 
##  -1   0   1 
## 406 421 226
```

```
data$s_CAMA_1_5 <- coalesce(data$s_CAMA_1_1_5, data$s_CAMA_2_1_5)
table(data$s_CAMA_1_5)
```

```
## 
##  -1   0   1 
## 166 484 405
```

```
data$s_CAMA_1_6 <- coalesce(data$s_CAMA_1_1_6, data$s_CAMA_2_1_6)
table(data$s_CAMA_1_6)
```

```
## 
##  -1   0   1 
## 264 563 224
```

```
data$s_CAMA_1_7 <- coalesce(data$s_CAMA_1_1_7, data$s_CAMA_2_1_7)
table(data$s_CAMA_1_7)
```

```
## 
##  -1   0   1 
## 207 433 416
```

```
data$s_CAMA_1_8 <- coalesce(data$s_CAMA_1_1_8, data$s_CAMA_2_1_8)
table(data$s_CAMA_1_8)
```

```
## 
##  -1   0   1 
## 211 538 309
```

```
data$s_CAMA_2_1 <- coalesce(data$s_CAMA_1_2_1,data$s_CAMA_2_2_1)
table(data$s_CAMA_2_1)
```

```
## 
##  -1   0   1 
## 467 361 230
```

```
data$s_CAMA_2_2 <- coalesce(data$s_CAMA_1_2_2,data$s_CAMA_2_2_2)
table(data$s_CAMA_2_2)
```

```
## 
##  -1   0   1 
## 263 462 332
```

```
data$s_CAMA_2_3 <- coalesce(data$s_CAMA_1_2_3,data$s_CAMA_2_2_3)
table(data$s_CAMA_2_3)
```

```
## 
##  -1   0   1 
## 325 451 283
```

```
data$s_CAMA_2_4 <- coalesce(data$s_CAMA_1_2_1,data$s_CAMA_2_2_4)
table(data$s_CAMA_2_4)
```

```
## 
##  -1   0   1 
## 449 392 218
```

```
data$s_CAMA_3 <- coalesce(data$s_CAMA_1_3, data$s_CAMA_2_3)
table(data$s_CAMA_3)
```

```
## 
##  -1   0   1 
## 385 446 231
```

### Merge and Recode METI

```
data$v_313[data$v_313 == 0] <- NA
data$v_314[data$v_314 == 0] <- NA
data$v_315[data$v_315 == 0] <- NA
data$v_316[data$v_316 == 0] <- NA
data$v_317[data$v_317 == 0] <- NA
data$v_323[data$v_323 == 0] <- NA
data$v_324[data$v_324 == 0] <- NA
data$v_325[data$v_325 == 0] <- NA
data$v_326[data$v_326 == 0] <- NA
data$v_327[data$v_327 == 0] <- NA
data$v_328[data$v_328 == 0] <- NA
data$v_329[data$v_329 == 0] <- NA
data$v_330[data$v_330 == 0] <- NA
data$v_331[data$v_331 == 0] <- NA

data$v_360[data$v_360 == 0] <- NA
data$v_361[data$v_361 == 0] <- NA
data$v_362[data$v_362 == 0] <- NA
data$v_363[data$v_363 == 0] <- NA
data$v_364[data$v_364 == 0] <- NA
data$v_365[data$v_365 == 0] <- NA
data$v_366[data$v_366 == 0] <- NA
data$v_367[data$v_367 == 0] <- NA
data$v_368[data$v_368 == 0] <- NA
data$v_369[data$v_369 == 0] <- NA
data$v_370[data$v_370 == 0] <- NA
data$v_371[data$v_371 == 0] <- NA
data$v_372[data$v_372 == 0] <- NA
data$v_373[data$v_373 == 0] <- NA

data$v_332[data$v_332 == 0] <- NA
data$v_333[data$v_333 == 0] <- NA
data$v_334[data$v_334 == 0] <- NA
data$v_335[data$v_335 == 0] <- NA
data$v_336[data$v_336 == 0] <- NA
data$v_337[data$v_337 == 0] <- NA
data$v_338[data$v_338 == 0] <- NA
data$v_339[data$v_339 == 0] <- NA
data$v_340[data$v_340 == 0] <- NA
data$v_341[data$v_341 == 0] <- NA
data$v_342[data$v_342 == 0] <- NA
data$v_343[data$v_343 == 0] <- NA
data$v_344[data$v_344 == 0] <- NA
data$v_345[data$v_345 == 0] <- NA

data$v_374[data$v_374 == 0] <- NA
data$v_375[data$v_375 == 0] <- NA
data$v_376[data$v_376 == 0] <- NA
data$v_377[data$v_377 == 0] <- NA
data$v_378[data$v_378 == 0] <- NA
data$v_379[data$v_379 == 0] <- NA
data$v_380[data$v_380 == 0] <- NA
data$v_381[data$v_381 == 0] <- NA
data$v_382[data$v_382 == 0] <- NA
data$v_383[data$v_383 == 0] <- NA
data$v_384[data$v_384 == 0] <- NA
data$v_385[data$v_385 == 0] <- NA
data$v_386[data$v_386 == 0] <- NA
data$v_387[data$v_387 == 0] <- NA

data <- rename(data, s_METI_1_Res_exp_1 = v_313, s_METI_1_Res_int_1 = v_314,
               s_METI_1_Res_ben_1 = v_315, s_METI_1_Res_ben_2 = v_316,
               s_METI_1_Res_ben_3 = v_317, s_METI_1_Res_int_2 = v_323, 
               s_METI_1_Res_exp_2 = v_324, s_METI_1_Res_exp_3 = v_325,
               s_METI_1_Res_exp_4 = v_326, s_METI_1_Res_exp_5 = v_327,
               s_METI_1_Res_ben_4 = v_328, s_METI_1_Res_int_3 = v_329,
               s_METI_1_Res_exp_6 = v_330, s_METI_1_Res_int_4 = v_331)

data <- rename(data, s_METI_2_Res_exp_1 = v_360, s_METI_2_Res_int_1 = v_361,
               s_METI_2_Res_ben_1 = v_362, s_METI_2_Res_ben_2 = v_363,
               s_METI_2_Res_ben_3 = v_364, s_METI_2_Res_int_2 = v_365, 
               s_METI_2_Res_exp_2 = v_366, s_METI_2_Res_exp_3 = v_367,
               s_METI_2_Res_exp_4 = v_368, s_METI_2_Res_exp_5 = v_369,
               s_METI_2_Res_ben_4 = v_370, s_METI_2_Res_int_3 = v_371,
               s_METI_2_Res_exp_6 = v_372, s_METI_2_Res_int_4 = v_373)

data <- rename(data, s_METI_1_Auth_exp_1 = v_332, s_METI_1_Auth_int_1 = v_333,
               s_METI_1_Auth_ben_1 = v_334, s_METI_1_Auth_ben_2 = v_335,
               s_METI_1_Auth_ben_3 = v_336, s_METI_1_Auth_int_2 = v_337, 
               s_METI_1_Auth_exp_2 = v_338, s_METI_1_Auth_exp_3 = v_339,
               s_METI_1_Auth_exp_4 = v_340, s_METI_1_Auth_exp_5 = v_341,
               s_METI_1_Auth_ben_4 = v_342, s_METI_1_Auth_int_3 = v_343,
               s_METI_1_Auth_exp_6 = v_344, s_METI_1_Auth_int_4 = v_345)

data <- rename(data, s_METI_2_Auth_exp_1 = v_374, s_METI_2_Auth_int_1 = v_375,
               s_METI_2_Auth_ben_1 = v_376, s_METI_2_Auth_ben_2 = v_377,
               s_METI_2_Auth_ben_3 = v_378, s_METI_2_Auth_int_2 = v_379, 
               s_METI_2_Auth_exp_2 = v_380, s_METI_2_Auth_exp_3 = v_381,
               s_METI_2_Auth_exp_4 = v_382, s_METI_2_Auth_exp_5 = v_383,
               s_METI_2_Auth_ben_4 = v_384, s_METI_2_Auth_int_3 = v_385,
               s_METI_2_Auth_exp_6 = v_386, s_METI_2_Auth_int_4 = v_387)

data$s_METI_1_exp_1 <- coalesce(data$s_METI_1_Res_exp_1,
                                data$s_METI_1_Auth_exp_1)
data$s_METI_1_int_1 <- coalesce(data$s_METI_1_Res_int_1,
                                data$s_METI_1_Auth_int_1)
data$s_METI_1_ben_1 <- coalesce(data$s_METI_1_Res_ben_1,
                                data$s_METI_1_Auth_ben_1)
data$s_METI_1_ben_2 <- coalesce(data$s_METI_1_Res_ben_2,
                                data$s_METI_1_Auth_ben_2)
data$s_METI_1_ben_3 <- coalesce(data$s_METI_1_Res_ben_3,
                                data$s_METI_1_Auth_ben_3)
data$s_METI_1_int_2 <- coalesce(data$s_METI_1_Res_int_2,
                                data$s_METI_1_Auth_int_2)
data$s_METI_1_exp_2 <- coalesce(data$s_METI_1_Res_exp_2,
                                data$s_METI_1_Auth_exp_2)
data$s_METI_1_exp_3 <- coalesce(data$s_METI_1_Res_exp_3,
                                data$s_METI_1_Auth_exp_3)
data$s_METI_1_exp_4 <- coalesce(data$s_METI_1_Res_exp_4,
                                data$s_METI_1_Auth_exp_4)
data$s_METI_1_exp_5 <- coalesce(data$s_METI_1_Res_exp_5,
                                data$s_METI_1_Auth_exp_5)
data$s_METI_1_ben_4 <- coalesce(data$s_METI_1_Res_ben_4,
                                data$s_METI_1_Auth_ben_4)
data$s_METI_1_int_3 <- coalesce(data$s_METI_1_Res_int_3,
                                data$s_METI_1_Auth_int_3)
data$s_METI_1_exp_6 <- coalesce(data$s_METI_1_Res_exp_6,
                                data$s_METI_1_Auth_exp_6)
data$s_METI_1_int_4 <- coalesce(data$s_METI_1_Res_int_4,
                                data$s_METI_1_Auth_int_4)

data$s_METI_2_exp_1 <- coalesce(data$s_METI_2_Res_exp_1,
                                data$s_METI_2_Auth_exp_1)
data$s_METI_2_int_1 <- coalesce(data$s_METI_2_Res_int_1,
                                data$s_METI_2_Auth_int_1)
data$s_METI_2_ben_1 <- coalesce(data$s_METI_2_Res_ben_1,
                                data$s_METI_2_Auth_ben_1)
data$s_METI_2_ben_2 <- coalesce(data$s_METI_2_Res_ben_2,
                                data$s_METI_2_Auth_ben_2)
data$s_METI_2_ben_3 <- coalesce(data$s_METI_2_Res_ben_3,
                                data$s_METI_2_Auth_ben_3)
data$s_METI_2_int_2 <- coalesce(data$s_METI_2_Res_int_2,
                                data$s_METI_2_Auth_int_2)
data$s_METI_2_exp_2 <- coalesce(data$s_METI_2_Res_exp_2,
                                data$s_METI_2_Auth_exp_2)
data$s_METI_2_exp_3 <- coalesce(data$s_METI_2_Res_exp_3,
                                data$s_METI_2_Auth_exp_3)
data$s_METI_2_exp_4 <- coalesce(data$s_METI_2_Res_exp_4,
                                data$s_METI_2_Auth_exp_4)
data$s_METI_2_exp_5 <- coalesce(data$s_METI_2_Res_exp_5,
                                data$s_METI_2_Auth_exp_5)
data$s_METI_2_ben_4 <- coalesce(data$s_METI_2_Res_ben_4,
                                data$s_METI_2_Auth_ben_4)
data$s_METI_2_int_3 <- coalesce(data$s_METI_2_Res_int_3,
                                data$s_METI_2_Auth_int_3)
data$s_METI_2_exp_6 <- coalesce(data$s_METI_2_Res_exp_6,
                                data$s_METI_2_Auth_exp_6)
data$s_METI_2_int_4 <- coalesce(data$s_METI_2_Res_int_4,
                                data$s_METI_2_Auth_int_4)

data$s_METI_exp_1 <- coalesce(data$s_METI_1_exp_1,data$s_METI_2_exp_1)
table(data$s_METI_exp_1)
```

```
## 
##   1   2   3   4   5   6   7 
##  33  42  68 366 362 561 598
```

```
data$s_METI_int_1 <- coalesce(data$s_METI_1_int_1,data$s_METI_2_int_1)
table(data$s_METI_int_1)
```

```
## 
##   1   2   3   4   5   6   7 
##  27  42  62 464 400 548 490
```

```
data$s_METI_ben_1 <- coalesce(data$s_METI_1_ben_1,data$s_METI_2_ben_1)
table(data$s_METI_ben_1)
```

```
## 
##   1   2   3   4   5   6   7 
##  25  34  89 468 399 521 491
```

```
data$s_METI_ben_2 <- coalesce(data$s_METI_1_ben_2,data$s_METI_2_ben_2)
table(data$s_METI_ben_2)
```

```
## 
##   1   2   3   4   5   6   7 
##  37  31  88 444 397 530 504
```

```
data$s_METI_ben_3 <- coalesce(data$s_METI_1_ben_3,data$s_METI_2_ben_3)
table(data$s_METI_ben_3)
```

```
## 
##   1   2   3   4   5   6   7 
##  35  33  76 377 386 558 567
```

```
data$s_METI_int_2 <- coalesce(data$s_METI_1_int_2,data$s_METI_2_int_2)
table(data$s_METI_int_2)
```

```
## 
##   1   2   3   4   5   6   7 
##  37  36  84 428 375 545 523
```

```
data$s_METI_exp_2 <- coalesce(data$s_METI_1_exp_2,data$s_METI_2_exp_2)
table(data$s_METI_exp_2)
```

```
## 
##   1   2   3   4   5   6   7 
##  33  33  72 356 364 587 589
```

```
data$s_METI_exp_3 <- coalesce(data$s_METI_1_exp_3,data$s_METI_2_exp_3)
table(data$s_METI_exp_3)
```

```
## 
##   1   2   3   4   5   6   7 
##  24  51 102 427 398 525 507
```

```
data$s_METI_exp_4 <- coalesce(data$s_METI_1_exp_4,data$s_METI_2_exp_4)
table(data$s_METI_exp_4)
```

```
## 
##   1   2   3   4   5   6   7 
##  27  45  78 385 375 565 560
```

```
data$s_METI_exp_5 <- coalesce(data$s_METI_1_exp_5,data$s_METI_2_exp_5)
table(data$s_METI_exp_5)
```

```
## 
##   1   2   3   4   5   6   7 
##  28  46  72 375 359 593 556
```

```
data$s_METI_ben_4 <- coalesce(data$s_METI_1_ben_4,data$s_METI_2_ben_4)
table(data$s_METI_ben_4)
```

```
## 
##   1   2   3   4   5   6   7 
##  33  36  83 462 402 528 479
```

```
data$s_METI_int_3 <- coalesce(data$s_METI_1_int_3,data$s_METI_2_int_3)
table(data$s_METI_int_3)
```

```
## 
##   1   2   3   4   5   6   7 
##  25  44  83 385 343 581 568
```

```
data$s_METI_exp_6 <- coalesce(data$s_METI_1_exp_6,data$s_METI_2_exp_6)
table(data$s_METI_exp_6)
```

```
## 
##   1   2   3   4   5   6   7 
##  24  33  74 364 370 583 586
```

```
data$s_METI_int_4 <- coalesce(data$s_METI_1_int_4,data$s_METI_2_int_4)
table(data$s_METI_int_4)
```

```
## 
##   1   2   3   4   5   6   7 
##  30  36  86 382 386 561 543
```

### Recode Awareness Check

```
data <- plyr::rename(data, c("v_388" = "s_awareness"))
data$s_awareness <- mapvalues(data$s_awareness, c(0,1,2,3,4,5,6,7,8,9),
                              c(1,0,0,0,0,0,0,0,0,0))
data$s_awareness <- factor(data$s_awareness, c(0,1),
                           labels = c("fail","pass"))
table(data$s_awareness)
```

```
## 
## fail pass 
##  658 1383
```

```
prop.table(table(data$s_awareness))
```

```
## 
##     fail     pass 
## 0.322391 0.677609
```

## Study Duration Analyses

```
data2 <- data[!data$dispcode == 22,]
length(unique(data$p_0001[data$dispcode == 31| data$dispcode == 32]))
```

```
## [1] 2041
```

```
View(data2)

data2$duration_minutes <- data2$duration/60
data2$duration_minutes[data2$duration_minutes <= 0] <- NA

psych::describe(data2$duration_minutes)
```

```
##    vars    n mean    sd median trimmed  mad  min    max range skew kurtosis
## X1    1 1753 21.9 11.91  18.45   19.93 8.06 8.02 104.83 96.82 2.05     6.21
##      se
## X1 0.28
```

```
hist.duration <- ggplot (data2, aes(duration_minutes)) + 
  theme(legend.position = "none") + geom_histogram(aes(y = after_stat(density)),
                                                   colour = "black",
                                                   fill = "white") +
  labs(x = "Duration in Minutes", y = "Density")

hist.duration + stat_function(fun = dnorm,
                              args = list(mean = mean(data2$duration_minutes,
                                                      na.rm = TRUE),
                                          sd = sd(data2$duration_minutes,
                                                  na.rm = TRUE)),
                              colour = "blue", size = 1)
```

```
## Warning: Using `size` aesthetic for lines was deprecated in ggplot2 3.4.0.
## ℹ Please use `linewidth` instead.
## This warning is displayed once every 8 hours.
## Call `lifecycle::last_lifecycle_warnings()` to see where this warning was
## generated.
```

```
## `stat_bin()` using `bins = 30`. Pick better value with `binwidth`.
```

```
## Warning: Removed 288 rows containing non-finite values (`stat_bin()`).
```

## Clean Wide-Format Dataset

```
names(data)
```

```
##   [1] "id"                  "external_lfdn"       "tester"             
##   [4] "dispcode"            "lastpage"            "quality"            
##   [7] "duration"            "condition"           "p_0001"             
##  [10] "c_0002"              "text_order"          "METI_target"        
##  [13] "s_sex"               "s_age"               "s_school"           
##  [16] "s_german"            "s_psychology"        "s_interest"         
##  [19] "s_contact"           "s_field"             "v_10"               
##  [22] "v_11"                "v_47"                "v_48"               
##  [25] "v_49"                "v_12"                "v_14"               
##  [28] "v_16"                "v_71"                "v_17"               
##  [31] "v_18"                "v_19"                "v_20"               
##  [34] "v_21"                "v_115"               "v_116"              
##  [37] "v_117"               "v_22"                "v_23"               
##  [40] "v_24"                "v_25"                "v_26"               
##  [43] "v_120"               "v_27"                "v_28"               
##  [46] "v_29"                "v_30"                "v_31"               
##  [49] "v_121"               "v_32"                "v_33"               
##  [52] "v_34"                "v_35"                "v_36"               
##  [55] "v_122"               "v_37"                "v_38"               
##  [58] "v_39"                "v_40"                "v_41"               
##  [61] "v_123"               "v_124"               "v_42"               
##  [64] "v_43"                "v_44"                "v_45"               
##  [67] "v_46"                "v_125"               "v_72"               
##  [70] "v_73"                "v_74"                "v_75"               
##  [73] "v_76"                "v_77"                "v_79"               
##  [76] "v_81"                "v_83"                "v_126"              
##  [79] "v_127"               "v_128"               "v_129"              
##  [82] "v_130"               "v_131"               "v_132"              
##  [85] "v_133"               "v_134"               "v_135"              
##  [88] "v_136"               "v_137"               "v_138"              
##  [91] "v_139"               "v_140"               "v_141"              
##  [94] "v_142"               "v_143"               "v_144"              
##  [97] "v_145"               "v_146"               "v_147"              
## [100] "v_148"               "v_149"               "v_150"              
## [103] "v_151"               "v_152"               "v_153"              
## [106] "v_154"               "v_155"               "v_156"              
## [109] "v_157"               "v_158"               "v_159"              
## [112] "v_160"               "v_161"               "v_162"              
## [115] "v_163"               "v_164"               "s_CAMA_1_1_1"       
## [118] "s_CAMA_1_1_2"        "s_CAMA_1_1_3"        "s_CAMA_1_1_4"       
## [121] "s_CAMA_1_1_5"        "s_CAMA_1_1_6"        "s_CAMA_1_1_7"       
## [124] "s_CAMA_1_1_8"        "s_CAMA_1_2_1"        "s_CAMA_1_2_2"       
## [127] "s_CAMA_1_2_3"        "s_CAMA_1_2_4"        "v_401"              
## [130] "v_91"                "v_92"                "v_93"               
## [133] "v_94"                "v_95"                "v_96"               
## [136] "v_98"                "v_100"               "v_102"              
## [139] "v_235"               "v_236"               "v_237"              
## [142] "v_238"               "v_239"               "v_240"              
## [145] "v_241"               "v_242"               "v_243"              
## [148] "v_244"               "v_245"               "v_246"              
## [151] "v_247"               "v_248"               "v_249"              
## [154] "v_250"               "v_251"               "v_252"              
## [157] "v_253"               "v_254"               "v_255"              
## [160] "v_256"               "v_257"               "v_258"              
## [163] "v_259"               "s_METI_1_Res_exp_1"  "s_METI_1_Res_int_1" 
## [166] "s_METI_1_Res_ben_1"  "s_METI_1_Res_ben_2"  "s_METI_1_Res_ben_3" 
## [169] "s_METI_1_Res_int_2"  "s_METI_1_Res_exp_2"  "s_METI_1_Res_exp_3" 
## [172] "s_METI_1_Res_exp_4"  "s_METI_1_Res_exp_5"  "s_METI_1_Res_ben_4" 
## [175] "s_METI_1_Res_int_3"  "s_METI_1_Res_exp_6"  "s_METI_1_Res_int_4" 
## [178] "s_METI_1_Auth_exp_1" "s_METI_1_Auth_int_1" "s_METI_1_Auth_ben_1"
## [181] "s_METI_1_Auth_ben_2" "s_METI_1_Auth_ben_3" "s_METI_1_Auth_int_2"
## [184] "s_METI_1_Auth_exp_2" "s_METI_1_Auth_exp_3" "s_METI_1_Auth_exp_4"
## [187] "s_METI_1_Auth_exp_5" "s_METI_1_Auth_ben_4" "s_METI_1_Auth_int_3"
## [190] "s_METI_1_Auth_exp_6" "s_METI_1_Auth_int_4" "v_103"              
## [193] "v_104"               "v_105"               "v_106"              
## [196] "v_107"               "v_108"               "v_110"              
## [199] "v_112"               "v_114"               "v_274"              
## [202] "v_275"               "v_276"               "v_277"              
## [205] "v_278"               "v_279"               "v_280"              
## [208] "v_281"               "v_282"               "v_283"              
## [211] "v_284"               "v_285"               "v_286"              
## [214] "v_287"               "v_288"               "v_289"              
## [217] "v_290"               "v_291"               "v_292"              
## [220] "v_293"               "v_294"               "v_295"              
## [223] "v_296"               "v_297"               "v_298"              
## [226] "s_CAMA_2_1_1"        "s_CAMA_2_1_2"        "s_CAMA_2_1_3"       
## [229] "s_CAMA_2_1_4"        "s_CAMA_2_1_5"        "s_CAMA_2_1_6"       
## [232] "s_CAMA_2_1_7"        "s_CAMA_2_1_8"        "s_CAMA_2_2_1"       
## [235] "s_CAMA_2_2_2"        "s_CAMA_2_2_3"        "s_CAMA_2_2_4"       
## [238] "v_402"               "s_METI_2_Res_exp_1"  "s_METI_2_Res_int_1" 
## [241] "s_METI_2_Res_ben_1"  "s_METI_2_Res_ben_2"  "s_METI_2_Res_ben_3" 
## [244] "s_METI_2_Res_int_2"  "s_METI_2_Res_exp_2"  "s_METI_2_Res_exp_3" 
## [247] "s_METI_2_Res_exp_4"  "s_METI_2_Res_exp_5"  "s_METI_2_Res_ben_4" 
## [250] "s_METI_2_Res_int_3"  "s_METI_2_Res_exp_6"  "s_METI_2_Res_int_4" 
## [253] "s_METI_2_Auth_exp_1" "s_METI_2_Auth_int_1" "s_METI_2_Auth_ben_1"
## [256] "s_METI_2_Auth_ben_2" "s_METI_2_Auth_ben_3" "s_METI_2_Auth_int_2"
## [259] "s_METI_2_Auth_exp_2" "s_METI_2_Auth_exp_3" "s_METI_2_Auth_exp_4"
## [262] "s_METI_2_Auth_exp_5" "s_METI_2_Auth_ben_4" "s_METI_2_Auth_int_3"
## [265] "s_METI_2_Auth_exp_6" "s_METI_2_Auth_int_4" "s_awareness"        
## [268] "browser"             "referer"             "device_type"        
## [271] "quota"               "quota_assignment"    "quota_rejected_id"  
## [274] "page_history"        "hflip"               "vflip"              
## [277] "output_mode"         "javascript"          "flash"              
## [280] "session_id"          "language"            "cleaned"            
## [283] "ats"                 "datetime"            "date_of_last_access"
## [286] "date_of_first_mail"  "rts6018385"          "rts6018739"         
## [289] "rts6018818"          "rts6019080"          "rts6019089"         
## [292] "rts6021451"          "rts6021455"          "rts6023513"         
## [295] "rts6023515"          "rts6023627"          "rts6023655"         
## [298] "rts6023657"          "rts6023660"          "rts6023667"         
## [301] "rts6023676"          "rts6023679"          "rts6033975"         
## [304] "METI_text"           "summary1"            "summary2"           
## [307] "version"             "causality"           "disclaimer"         
## [310] "CAMA"                "dropout"             "accessibility_1"    
## [313] "accessibility_2"     "understanding_1"     "understanding_2"    
## [316] "empowerment_1"       "empowerment_2"       "credibility_1"      
## [319] "credibility_2"       "relevance_1"         "relevance_2"        
## [322] "curiosity_1"         "curiosity_2"         "boredom_1"          
## [325] "boredom_2"           "confusion_1"         "confusion_2"        
## [328] "frustration_1"       "frustration_2"       "s_relationship_1"   
## [331] "s_relationship_2"    "s_relationship_3"    "s_relationship_4"   
## [334] "s_relationship_5"    "s_relationship_6"    "s_relationship_7"   
## [337] "s_relationship_8"    "s_extent_1"          "s_extent_2"         
## [340] "s_extent_3"          "s_extent_4"          "s_extent_5"         
## [343] "s_extent_6"          "s_diff_1_1"          "s_diff_1_2"         
## [346] "s_diff_1_3"          "s_diff_1_4"          "s_diff_1_5"         
## [349] "s_diff_1_6"          "s_diff_2_1"          "s_diff_2_2"         
## [352] "s_diff_2_3"          "s_diff_2_4"          "s_diff_2_5"         
## [355] "s_diff_2_6"          "s_funding_1_1"       "s_funding_1_2"      
## [358] "s_funding_1_3"       "s_funding_1_4"       "s_funding_1_5"      
## [361] "s_funding_1_6"       "s_funding_2_1"       "s_funding_2_2"      
## [364] "s_funding_2_3"       "s_funding_2_4"       "s_funding_2_5"      
## [367] "s_funding_2_6"       "s_coi_1_1"           "s_coi_1_2"          
## [370] "s_coi_1_3"           "s_coi_1_4"           "s_coi_1_5"          
## [373] "s_coi_1_6"           "s_coi_1_7"           "s_coi_2_1"          
## [376] "s_coi_2_2"           "s_coi_2_3"           "s_coi_2_4"          
## [379] "s_coi_2_5"           "s_coi_2_6"           "s_coi_2_7"          
## [382] "s_causality_1_1"     "s_causality_1_2"     "s_causality_1_3"    
## [385] "s_causality_1_4"     "s_causality_1_5"     "s_causality_1_6"    
## [388] "s_causality_2_1"     "s_causality_2_2"     "s_causality_2_3"    
## [391] "s_causality_2_4"     "s_causality_2_5"     "s_causality_2_6"    
## [394] "s_CAMA_1_3"          "s_CAMA_2_3"          "s_CAMA_1_1"         
## [397] "s_CAMA_1_2"          "s_CAMA_1_4"          "s_CAMA_1_5"         
## [400] "s_CAMA_1_6"          "s_CAMA_1_7"          "s_CAMA_1_8"         
## [403] "s_CAMA_2_1"          "s_CAMA_2_2"          "s_CAMA_2_4"         
## [406] "s_CAMA_3"            "s_METI_1_exp_1"      "s_METI_1_int_1"     
## [409] "s_METI_1_ben_1"      "s_METI_1_ben_2"      "s_METI_1_ben_3"     
## [412] "s_METI_1_int_2"      "s_METI_1_exp_2"      "s_METI_1_exp_3"     
## [415] "s_METI_1_exp_4"      "s_METI_1_exp_5"      "s_METI_1_ben_4"     
## [418] "s_METI_1_int_3"      "s_METI_1_exp_6"      "s_METI_1_int_4"     
## [421] "s_METI_2_exp_1"      "s_METI_2_int_1"      "s_METI_2_ben_1"     
## [424] "s_METI_2_ben_2"      "s_METI_2_ben_3"      "s_METI_2_int_2"     
## [427] "s_METI_2_exp_2"      "s_METI_2_exp_3"      "s_METI_2_exp_4"     
## [430] "s_METI_2_exp_5"      "s_METI_2_ben_4"      "s_METI_2_int_3"     
## [433] "s_METI_2_exp_6"      "s_METI_2_int_4"      "s_METI_exp_1"       
## [436] "s_METI_int_1"        "s_METI_ben_1"        "s_METI_ben_2"       
## [439] "s_METI_ben_3"        "s_METI_int_2"        "s_METI_exp_2"       
## [442] "s_METI_exp_3"        "s_METI_exp_4"        "s_METI_exp_5"       
## [445] "s_METI_ben_4"        "s_METI_int_3"        "s_METI_exp_6"       
## [448] "s_METI_int_4"
```

```
data_wide <- data[,!names(data) %in% c("external_lfdn","tester","lastpage",
                                       "quality","p_0001","c_0002","browser",
                                       "referer","device_type",
                                       "quota_assignment","quota_rejected_id",
                                       "page_history","hflip","vflip",
                                       "output_mode","javascript","flash",
                                       "session_id","language","cleaned","ats",
                                       "datetime","date_of_last_access",
                                       "day_of_first_mail","rts6018385",
                                       "rts6018739","rts6018818","rts6019080",
                                       "rts6019089","rts6021451","rts6021455",
                                       "rts6023513","rts6023515","rts6023627",
                                       "rts6023655","rts6023657","rts6023660",
                                       "rts6023667","rts6023676","rts6023679",
                                       "rts6033975")]
names(data_wide)
```

```
##   [1] "id"                  "dispcode"            "duration"           
##   [4] "condition"           "text_order"          "METI_target"        
##   [7] "s_sex"               "s_age"               "s_school"           
##  [10] "s_german"            "s_psychology"        "s_interest"         
##  [13] "s_contact"           "s_field"             "v_10"               
##  [16] "v_11"                "v_47"                "v_48"               
##  [19] "v_49"                "v_12"                "v_14"               
##  [22] "v_16"                "v_71"                "v_17"               
##  [25] "v_18"                "v_19"                "v_20"               
##  [28] "v_21"                "v_115"               "v_116"              
##  [31] "v_117"               "v_22"                "v_23"               
##  [34] "v_24"                "v_25"                "v_26"               
##  [37] "v_120"               "v_27"                "v_28"               
##  [40] "v_29"                "v_30"                "v_31"               
##  [43] "v_121"               "v_32"                "v_33"               
##  [46] "v_34"                "v_35"                "v_36"               
##  [49] "v_122"               "v_37"                "v_38"               
##  [52] "v_39"                "v_40"                "v_41"               
##  [55] "v_123"               "v_124"               "v_42"               
##  [58] "v_43"                "v_44"                "v_45"               
##  [61] "v_46"                "v_125"               "v_72"               
##  [64] "v_73"                "v_74"                "v_75"               
##  [67] "v_76"                "v_77"                "v_79"               
##  [70] "v_81"                "v_83"                "v_126"              
##  [73] "v_127"               "v_128"               "v_129"              
##  [76] "v_130"               "v_131"               "v_132"              
##  [79] "v_133"               "v_134"               "v_135"              
##  [82] "v_136"               "v_137"               "v_138"              
##  [85] "v_139"               "v_140"               "v_141"              
##  [88] "v_142"               "v_143"               "v_144"              
##  [91] "v_145"               "v_146"               "v_147"              
##  [94] "v_148"               "v_149"               "v_150"              
##  [97] "v_151"               "v_152"               "v_153"              
## [100] "v_154"               "v_155"               "v_156"              
## [103] "v_157"               "v_158"               "v_159"              
## [106] "v_160"               "v_161"               "v_162"              
## [109] "v_163"               "v_164"               "s_CAMA_1_1_1"       
## [112] "s_CAMA_1_1_2"        "s_CAMA_1_1_3"        "s_CAMA_1_1_4"       
## [115] "s_CAMA_1_1_5"        "s_CAMA_1_1_6"        "s_CAMA_1_1_7"       
## [118] "s_CAMA_1_1_8"        "s_CAMA_1_2_1"        "s_CAMA_1_2_2"       
## [121] "s_CAMA_1_2_3"        "s_CAMA_1_2_4"        "v_401"              
## [124] "v_91"                "v_92"                "v_93"               
## [127] "v_94"                "v_95"                "v_96"               
## [130] "v_98"                "v_100"               "v_102"              
## [133] "v_235"               "v_236"               "v_237"              
## [136] "v_238"               "v_239"               "v_240"              
## [139] "v_241"               "v_242"               "v_243"              
## [142] "v_244"               "v_245"               "v_246"              
## [145] "v_247"               "v_248"               "v_249"              
## [148] "v_250"               "v_251"               "v_252"              
## [151] "v_253"               "v_254"               "v_255"              
## [154] "v_256"               "v_257"               "v_258"              
## [157] "v_259"               "s_METI_1_Res_exp_1"  "s_METI_1_Res_int_1" 
## [160] "s_METI_1_Res_ben_1"  "s_METI_1_Res_ben_2"  "s_METI_1_Res_ben_3" 
## [163] "s_METI_1_Res_int_2"  "s_METI_1_Res_exp_2"  "s_METI_1_Res_exp_3" 
## [166] "s_METI_1_Res_exp_4"  "s_METI_1_Res_exp_5"  "s_METI_1_Res_ben_4" 
## [169] "s_METI_1_Res_int_3"  "s_METI_1_Res_exp_6"  "s_METI_1_Res_int_4" 
## [172] "s_METI_1_Auth_exp_1" "s_METI_1_Auth_int_1" "s_METI_1_Auth_ben_1"
## [175] "s_METI_1_Auth_ben_2" "s_METI_1_Auth_ben_3" "s_METI_1_Auth_int_2"
## [178] "s_METI_1_Auth_exp_2" "s_METI_1_Auth_exp_3" "s_METI_1_Auth_exp_4"
## [181] "s_METI_1_Auth_exp_5" "s_METI_1_Auth_ben_4" "s_METI_1_Auth_int_3"
## [184] "s_METI_1_Auth_exp_6" "s_METI_1_Auth_int_4" "v_103"              
## [187] "v_104"               "v_105"               "v_106"              
## [190] "v_107"               "v_108"               "v_110"              
## [193] "v_112"               "v_114"               "v_274"              
## [196] "v_275"               "v_276"               "v_277"              
## [199] "v_278"               "v_279"               "v_280"              
## [202] "v_281"               "v_282"               "v_283"              
## [205] "v_284"               "v_285"               "v_286"              
## [208] "v_287"               "v_288"               "v_289"              
## [211] "v_290"               "v_291"               "v_292"              
## [214] "v_293"               "v_294"               "v_295"              
## [217] "v_296"               "v_297"               "v_298"              
## [220] "s_CAMA_2_1_1"        "s_CAMA_2_1_2"        "s_CAMA_2_1_3"       
## [223] "s_CAMA_2_1_4"        "s_CAMA_2_1_5"        "s_CAMA_2_1_6"       
## [226] "s_CAMA_2_1_7"        "s_CAMA_2_1_8"        "s_CAMA_2_2_1"       
## [229] "s_CAMA_2_2_2"        "s_CAMA_2_2_3"        "s_CAMA_2_2_4"       
## [232] "v_402"               "s_METI_2_Res_exp_1"  "s_METI_2_Res_int_1" 
## [235] "s_METI_2_Res_ben_1"  "s_METI_2_Res_ben_2"  "s_METI_2_Res_ben_3" 
## [238] "s_METI_2_Res_int_2"  "s_METI_2_Res_exp_2"  "s_METI_2_Res_exp_3" 
## [241] "s_METI_2_Res_exp_4"  "s_METI_2_Res_exp_5"  "s_METI_2_Res_ben_4" 
## [244] "s_METI_2_Res_int_3"  "s_METI_2_Res_exp_6"  "s_METI_2_Res_int_4" 
## [247] "s_METI_2_Auth_exp_1" "s_METI_2_Auth_int_1" "s_METI_2_Auth_ben_1"
## [250] "s_METI_2_Auth_ben_2" "s_METI_2_Auth_ben_3" "s_METI_2_Auth_int_2"
## [253] "s_METI_2_Auth_exp_2" "s_METI_2_Auth_exp_3" "s_METI_2_Auth_exp_4"
## [256] "s_METI_2_Auth_exp_5" "s_METI_2_Auth_ben_4" "s_METI_2_Auth_int_3"
## [259] "s_METI_2_Auth_exp_6" "s_METI_2_Auth_int_4" "s_awareness"        
## [262] "quota"               "date_of_first_mail"  "METI_text"          
## [265] "summary1"            "summary2"            "version"            
## [268] "causality"           "disclaimer"          "CAMA"               
## [271] "dropout"             "accessibility_1"     "accessibility_2"    
## [274] "understanding_1"     "understanding_2"     "empowerment_1"      
## [277] "empowerment_2"       "credibility_1"       "credibility_2"      
## [280] "relevance_1"         "relevance_2"         "curiosity_1"        
## [283] "curiosity_2"         "boredom_1"           "boredom_2"          
## [286] "confusion_1"         "confusion_2"         "frustration_1"      
## [289] "frustration_2"       "s_relationship_1"    "s_relationship_2"   
## [292] "s_relationship_3"    "s_relationship_4"    "s_relationship_5"   
## [295] "s_relationship_6"    "s_relationship_7"    "s_relationship_8"   
## [298] "s_extent_1"          "s_extent_2"          "s_extent_3"         
## [301] "s_extent_4"          "s_extent_5"          "s_extent_6"         
## [304] "s_diff_1_1"          "s_diff_1_2"          "s_diff_1_3"         
## [307] "s_diff_1_4"          "s_diff_1_5"          "s_diff_1_6"         
## [310] "s_diff_2_1"          "s_diff_2_2"          "s_diff_2_3"         
## [313] "s_diff_2_4"          "s_diff_2_5"          "s_diff_2_6"         
## [316] "s_funding_1_1"       "s_funding_1_2"       "s_funding_1_3"      
## [319] "s_funding_1_4"       "s_funding_1_5"       "s_funding_1_6"      
## [322] "s_funding_2_1"       "s_funding_2_2"       "s_funding_2_3"      
## [325] "s_funding_2_4"       "s_funding_2_5"       "s_funding_2_6"      
## [328] "s_coi_1_1"           "s_coi_1_2"           "s_coi_1_3"          
## [331] "s_coi_1_4"           "s_coi_1_5"           "s_coi_1_6"          
## [334] "s_coi_1_7"           "s_coi_2_1"           "s_coi_2_2"          
## [337] "s_coi_2_3"           "s_coi_2_4"           "s_coi_2_5"          
## [340] "s_coi_2_6"           "s_coi_2_7"           "s_causality_1_1"    
## [343] "s_causality_1_2"     "s_causality_1_3"     "s_causality_1_4"    
## [346] "s_causality_1_5"     "s_causality_1_6"     "s_causality_2_1"    
## [349] "s_causality_2_2"     "s_causality_2_3"     "s_causality_2_4"    
## [352] "s_causality_2_5"     "s_causality_2_6"     "s_CAMA_1_3"         
## [355] "s_CAMA_2_3"          "s_CAMA_1_1"          "s_CAMA_1_2"         
## [358] "s_CAMA_1_4"          "s_CAMA_1_5"          "s_CAMA_1_6"         
## [361] "s_CAMA_1_7"          "s_CAMA_1_8"          "s_CAMA_2_1"         
## [364] "s_CAMA_2_2"          "s_CAMA_2_4"          "s_CAMA_3"           
## [367] "s_METI_1_exp_1"      "s_METI_1_int_1"      "s_METI_1_ben_1"     
## [370] "s_METI_1_ben_2"      "s_METI_1_ben_3"      "s_METI_1_int_2"     
## [373] "s_METI_1_exp_2"      "s_METI_1_exp_3"      "s_METI_1_exp_4"     
## [376] "s_METI_1_exp_5"      "s_METI_1_ben_4"      "s_METI_1_int_3"     
## [379] "s_METI_1_exp_6"      "s_METI_1_int_4"      "s_METI_2_exp_1"     
## [382] "s_METI_2_int_1"      "s_METI_2_ben_1"      "s_METI_2_ben_2"     
## [385] "s_METI_2_ben_3"      "s_METI_2_int_2"      "s_METI_2_exp_2"     
## [388] "s_METI_2_exp_3"      "s_METI_2_exp_4"      "s_METI_2_exp_5"     
## [391] "s_METI_2_ben_4"      "s_METI_2_int_3"      "s_METI_2_exp_6"     
## [394] "s_METI_2_int_4"      "s_METI_exp_1"        "s_METI_int_1"       
## [397] "s_METI_ben_1"        "s_METI_ben_2"        "s_METI_ben_3"       
## [400] "s_METI_int_2"        "s_METI_exp_2"        "s_METI_exp_3"       
## [403] "s_METI_exp_4"        "s_METI_exp_5"        "s_METI_ben_4"       
## [406] "s_METI_int_3"        "s_METI_exp_6"        "s_METI_int_4"
```

```
str(data_wide)
```

```
## 'data.frame':    3080 obs. of  408 variables:
##  $ id                 : Factor w/ 6705 levels "1","2","3","4",..: 3600 4018 1355 2457 4593 1215 3269 2531 5304 2352 ...
##  $ dispcode           : int  31 22 22 31 22 31 31 31 31 31 ...
##  $ duration           : int  779 68 19 1043 36 546 746 938 568 1094 ...
##  $ condition          : Factor w/ 6 levels "1","2","3","4",..: 3 2 NA 4 5 3 5 6 2 1 ...
##  $ text_order         : Factor w/ 2 levels "Barth","Faerber": 1 2 NA 2 2 1 2 1 1 2 ...
##  $ METI_target        : Factor w/ 2 levels "Study Authors",..: 2 2 NA 1 2 2 1 2 1 2 ...
##  $ s_sex              : Factor w/ 2 levels "female","male": 2 2 2 1 2 2 1 2 1 1 ...
##  $ s_age              : int  31 42 25 31 47 54 45 43 51 57 ...
##  $ s_school           : Factor w/ 3 levels "Haupt","Real",..: 2 2 1 1 1 1 2 3 2 3 ...
##  $ s_german           : int  1 1 1 1 1 1 1 1 1 1 ...
##  $ s_psychology       : int  2 2 2 2 2 2 2 2 2 2 ...
##  $ s_interest         : int  5 6 5 5 5 5 4 7 7 8 ...
##  $ s_contact          : int  1 1 2 1 1 1 2 2 5 3 ...
##  $ s_field            : chr  NA NA NA NA ...
##  $ v_10               : int  4 NA NA NA NA 3 NA 5 NA NA ...
##  $ v_11               : int  6 NA NA NA NA 2 NA 7 6 NA ...
##  $ v_47               : int  3 NA NA NA NA 3 NA 7 6 NA ...
##  $ v_48               : int  6 NA NA NA NA 3 NA 7 NA NA ...
##  $ v_49               : int  6 NA NA NA NA 5 NA 7 NA NA ...
##  $ v_12               : int  2 NA NA NA NA 3 NA 4 2 NA ...
##  $ v_14               : int  1 NA NA NA NA 2 NA 1 4 NA ...
##  $ v_16               : int  3 NA NA NA NA 4 NA 1 2 NA ...
##  $ v_71               : int  3 NA NA NA NA 2 NA 1 3 NA ...
##  $ v_17               : int  1 NA NA NA NA 3 NA 1 2 NA ...
##  $ v_18               : int  1 NA NA NA NA 3 NA 1 1 NA ...
##  $ v_19               : int  2 NA NA NA NA 3 NA 1 3 NA ...
##  $ v_20               : int  2 NA NA NA NA 3 NA 1 2 NA ...
##  $ v_21               : int  1 NA NA NA NA 3 NA 1 1 NA ...
##  $ v_115              : int  1 NA NA NA NA 3 NA 1 NA NA ...
##  $ v_116              : int  1 NA NA NA NA 3 NA 1 1 NA ...
##  $ v_117              : int  1 NA NA NA NA 3 NA 1 1 NA ...
##  $ v_22               : int  3 NA NA NA NA 3 NA 1 3 NA ...
##  $ v_23               : int  1 NA NA NA NA 3 NA 1 1 NA ...
##  $ v_24               : int  1 NA NA NA NA 3 NA 3 2 NA ...
##  $ v_25               : int  1 NA NA NA NA 3 NA 1 1 NA ...
##  $ v_26               : int  1 NA NA NA NA 3 NA 1 3 NA ...
##  $ v_120              : int  1 NA NA NA NA 3 NA 1 2 NA ...
##  $ v_27               : num  1 NA NA NA NA 0 NA -1 -1 NA ...
##  $ v_28               : num  1 NA NA NA NA 0 NA -1 1 NA ...
##  $ v_29               : num  -1 NA NA NA NA 0 NA 1 -1 NA ...
##  $ v_30               : num  1 NA NA NA NA 0 NA -1 1 NA ...
##  $ v_31               : num  1 NA NA NA NA 0 NA 1 0 NA ...
##  $ v_121              : num  1 NA NA NA NA 0 NA 1 -1 NA ...
##  $ v_32               : num  1 NA NA NA NA 0 NA -1 0 NA ...
##  $ v_33               : num  1 NA NA NA NA 0 NA -1 1 NA ...
##  $ v_34               : num  1 NA NA NA NA 0 NA -1 0 NA ...
##  $ v_35               : num  1 NA NA NA NA 0 NA -1 -1 NA ...
##  $ v_36               : num  1 NA NA NA NA 0 NA -1 -1 NA ...
##  $ v_122              : num  1 NA NA NA NA 0 NA 1 -1 NA ...
##  $ v_37               : num  1 NA NA NA NA 0 NA -1 1 NA ...
##  $ v_38               : num  NA NA NA NA NA 0 NA 0 1 NA ...
##  $ v_39               : num  1 NA NA NA NA 0 NA 0 NA NA ...
##  $ v_40               : num  1 NA NA NA NA 0 NA -1 -1 NA ...
##  $ v_41               : num  -1 NA NA NA NA 0 NA -1 1 NA ...
##  $ v_123              : num  1 NA NA NA NA 0 NA 1 1 NA ...
##  $ v_124              : num  1 NA NA NA NA 0 NA -1 1 NA ...
##  $ v_42               : num  0 NA NA NA NA 0 NA 1 0 NA ...
##  $ v_43               : num  -1 NA NA NA NA 0 NA -1 1 NA ...
##  $ v_44               : num  0 NA NA NA NA 0 NA -1 1 NA ...
##  $ v_45               : num  -1 NA NA NA NA 0 NA -1 -1 NA ...
##  $ v_46               : num  0 NA NA NA NA 0 NA 1 -1 NA ...
##  $ v_125              : num  0 NA NA NA NA 0 NA -1 -1 NA ...
##  $ v_72               : int  NA 6 NA 4 NA NA 4 NA NA 8 ...
##  $ v_73               : int  NA 7 NA 5 NA NA 3 NA NA 8 ...
##  $ v_74               : int  NA 7 NA 5 NA NA 3 NA NA 8 ...
##  $ v_75               : int  NA 7 NA 6 NA NA 4 NA NA 8 ...
##  $ v_76               : int  NA 7 NA 8 NA NA 6 NA NA 8 ...
##  $ v_77               : int  NA 4 NA 4 NA NA 3 NA NA 5 ...
##  $ v_79               : int  NA 1 NA 3 NA NA 2 NA NA 1 ...
##  $ v_81               : int  NA 1 NA 1 NA NA 4 NA NA 1 ...
##  $ v_83               : int  NA 1 NA 2 NA NA 2 NA NA 1 ...
##  $ v_126              : int  NA NA NA 1 NA NA 3 NA NA 1 ...
##  $ v_127              : int  NA NA NA 1 NA NA 3 NA NA 1 ...
##  $ v_128              : int  NA NA NA 3 NA NA 3 NA NA 2 ...
##  $ v_129              : int  NA NA NA 3 NA NA 3 NA NA 2 ...
##  $ v_130              : int  NA NA NA 2 NA NA 3 NA NA 1 ...
##  $ v_131              : int  NA NA NA 1 NA NA 1 NA NA 1 ...
##  $ v_132              : int  NA NA NA 1 NA NA 1 NA NA 1 ...
##  $ v_133              : int  NA NA NA 2 NA NA 3 NA NA 1 ...
##  $ v_134              : int  NA NA NA 1 NA NA 3 NA NA 1 ...
##  $ v_135              : int  NA NA NA 2 NA NA 3 NA NA 1 ...
##  $ v_136              : int  NA NA NA 1 NA NA 1 NA NA 1 ...
##  $ v_137              : int  NA NA NA 2 NA NA 2 NA NA 1 ...
##  $ v_138              : int  NA NA NA 1 NA NA 3 NA NA 1 ...
##  $ v_139              : int  NA NA NA 1 NA NA 3 NA NA 1 ...
##  $ v_140              : logi  NA NA NA NA NA NA ...
##  $ v_141              : logi  NA NA NA NA NA NA ...
##  $ v_142              : logi  NA NA NA NA NA NA ...
##  $ v_143              : logi  NA NA NA NA NA NA ...
##  $ v_144              : logi  NA NA NA NA NA NA ...
##  $ v_145              : logi  NA NA NA NA NA NA ...
##  $ v_146              : num  NA NA NA -1 NA NA -1 NA NA -1 ...
##  $ v_147              : num  NA NA NA -1 NA NA -1 NA NA -1 ...
##  $ v_148              : num  NA NA NA 1 NA NA -1 NA NA 0 ...
##  $ v_149              : num  NA NA NA -1 NA NA 0 NA NA 0 ...
##  $ v_150              : num  NA NA NA 0 NA NA NA NA NA 0 ...
##  $ v_151              : num  NA NA NA -1 NA NA 1 NA NA 1 ...
##  $ v_152              : num  NA NA NA -1 NA NA 0 NA NA -1 ...
##  $ v_153              : num  NA NA NA -1 NA NA 1 NA NA -1 ...
##   [list output truncated]
```

```
View(data_wide)

data2_wide <- data2[,!names(data2) %in% c("external_lfdn","tester","lastpage",
                                       "quality","p_0001","c_0002","browser",
                                       "referer","device_type",
                                       "quota_assignment","quota_rejected_id",
                                       "page_history","hflip","vflip",
                                       "output_mode","javascript","flash",
                                       "session_id","language","cleaned","ats",
                                       "datetime","date_of_last_access",
                                       "day_of_first_mail","rts6018385",
                                       "rts6018739","rts6018818","rts6019080",
                                       "rts6019089","rts6021451","rts6021455",
                                       "rts6023513","rts6023515","rts6023627",
                                       "rts6023655","rts6023657","rts6023660",
                                       "rts6023667","rts6023676","rts6023679",
                                       "rts6033975")]
names(data2_wide)
```

```
##   [1] "id"                  "dispcode"            "duration"           
##   [4] "condition"           "text_order"          "METI_target"        
##   [7] "s_sex"               "s_age"               "s_school"           
##  [10] "s_german"            "s_psychology"        "s_interest"         
##  [13] "s_contact"           "s_field"             "v_10"               
##  [16] "v_11"                "v_47"                "v_48"               
##  [19] "v_49"                "v_12"                "v_14"               
##  [22] "v_16"                "v_71"                "v_17"               
##  [25] "v_18"                "v_19"                "v_20"               
##  [28] "v_21"                "v_115"               "v_116"              
##  [31] "v_117"               "v_22"                "v_23"               
##  [34] "v_24"                "v_25"                "v_26"               
##  [37] "v_120"               "v_27"                "v_28"               
##  [40] "v_29"                "v_30"                "v_31"               
##  [43] "v_121"               "v_32"                "v_33"               
##  [46] "v_34"                "v_35"                "v_36"               
##  [49] "v_122"               "v_37"                "v_38"               
##  [52] "v_39"                "v_40"                "v_41"               
##  [55] "v_123"               "v_124"               "v_42"               
##  [58] "v_43"                "v_44"                "v_45"               
##  [61] "v_46"                "v_125"               "v_72"               
##  [64] "v_73"                "v_74"                "v_75"               
##  [67] "v_76"                "v_77"                "v_79"               
##  [70] "v_81"                "v_83"                "v_126"              
##  [73] "v_127"               "v_128"               "v_129"              
##  [76] "v_130"               "v_131"               "v_132"              
##  [79] "v_133"               "v_134"               "v_135"              
##  [82] "v_136"               "v_137"               "v_138"              
##  [85] "v_139"               "v_140"               "v_141"              
##  [88] "v_142"               "v_143"               "v_144"              
##  [91] "v_145"               "v_146"               "v_147"              
##  [94] "v_148"               "v_149"               "v_150"              
##  [97] "v_151"               "v_152"               "v_153"              
## [100] "v_154"               "v_155"               "v_156"              
## [103] "v_157"               "v_158"               "v_159"              
## [106] "v_160"               "v_161"               "v_162"              
## [109] "v_163"               "v_164"               "s_CAMA_1_1_1"       
## [112] "s_CAMA_1_1_2"        "s_CAMA_1_1_3"        "s_CAMA_1_1_4"       
## [115] "s_CAMA_1_1_5"        "s_CAMA_1_1_6"        "s_CAMA_1_1_7"       
## [118] "s_CAMA_1_1_8"        "s_CAMA_1_2_1"        "s_CAMA_1_2_2"       
## [121] "s_CAMA_1_2_3"        "s_CAMA_1_2_4"        "v_401"              
## [124] "v_91"                "v_92"                "v_93"               
## [127] "v_94"                "v_95"                "v_96"               
## [130] "v_98"                "v_100"               "v_102"              
## [133] "v_235"               "v_236"               "v_237"              
## [136] "v_238"               "v_239"               "v_240"              
## [139] "v_241"               "v_242"               "v_243"              
## [142] "v_244"               "v_245"               "v_246"              
## [145] "v_247"               "v_248"               "v_249"              
## [148] "v_250"               "v_251"               "v_252"              
## [151] "v_253"               "v_254"               "v_255"              
## [154] "v_256"               "v_257"               "v_258"              
## [157] "v_259"               "s_METI_1_Res_exp_1"  "s_METI_1_Res_int_1" 
## [160] "s_METI_1_Res_ben_1"  "s_METI_1_Res_ben_2"  "s_METI_1_Res_ben_3" 
## [163] "s_METI_1_Res_int_2"  "s_METI_1_Res_exp_2"  "s_METI_1_Res_exp_3" 
## [166] "s_METI_1_Res_exp_4"  "s_METI_1_Res_exp_5"  "s_METI_1_Res_ben_4" 
## [169] "s_METI_1_Res_int_3"  "s_METI_1_Res_exp_6"  "s_METI_1_Res_int_4" 
## [172] "s_METI_1_Auth_exp_1" "s_METI_1_Auth_int_1" "s_METI_1_Auth_ben_1"
## [175] "s_METI_1_Auth_ben_2" "s_METI_1_Auth_ben_3" "s_METI_1_Auth_int_2"
## [178] "s_METI_1_Auth_exp_2" "s_METI_1_Auth_exp_3" "s_METI_1_Auth_exp_4"
## [181] "s_METI_1_Auth_exp_5" "s_METI_1_Auth_ben_4" "s_METI_1_Auth_int_3"
## [184] "s_METI_1_Auth_exp_6" "s_METI_1_Auth_int_4" "v_103"              
## [187] "v_104"               "v_105"               "v_106"              
## [190] "v_107"               "v_108"               "v_110"              
## [193] "v_112"               "v_114"               "v_274"              
## [196] "v_275"               "v_276"               "v_277"              
## [199] "v_278"               "v_279"               "v_280"              
## [202] "v_281"               "v_282"               "v_283"              
## [205] "v_284"               "v_285"               "v_286"              
## [208] "v_287"               "v_288"               "v_289"              
## [211] "v_290"               "v_291"               "v_292"              
## [214] "v_293"               "v_294"               "v_295"              
## [217] "v_296"               "v_297"               "v_298"              
## [220] "s_CAMA_2_1_1"        "s_CAMA_2_1_2"        "s_CAMA_2_1_3"       
## [223] "s_CAMA_2_1_4"        "s_CAMA_2_1_5"        "s_CAMA_2_1_6"       
## [226] "s_CAMA_2_1_7"        "s_CAMA_2_1_8"        "s_CAMA_2_2_1"       
## [229] "s_CAMA_2_2_2"        "s_CAMA_2_2_3"        "s_CAMA_2_2_4"       
## [232] "v_402"               "s_METI_2_Res_exp_1"  "s_METI_2_Res_int_1" 
## [235] "s_METI_2_Res_ben_1"  "s_METI_2_Res_ben_2"  "s_METI_2_Res_ben_3" 
## [238] "s_METI_2_Res_int_2"  "s_METI_2_Res_exp_2"  "s_METI_2_Res_exp_3" 
## [241] "s_METI_2_Res_exp_4"  "s_METI_2_Res_exp_5"  "s_METI_2_Res_ben_4" 
## [244] "s_METI_2_Res_int_3"  "s_METI_2_Res_exp_6"  "s_METI_2_Res_int_4" 
## [247] "s_METI_2_Auth_exp_1" "s_METI_2_Auth_int_1" "s_METI_2_Auth_ben_1"
## [250] "s_METI_2_Auth_ben_2" "s_METI_2_Auth_ben_3" "s_METI_2_Auth_int_2"
## [253] "s_METI_2_Auth_exp_2" "s_METI_2_Auth_exp_3" "s_METI_2_Auth_exp_4"
## [256] "s_METI_2_Auth_exp_5" "s_METI_2_Auth_ben_4" "s_METI_2_Auth_int_3"
## [259] "s_METI_2_Auth_exp_6" "s_METI_2_Auth_int_4" "s_awareness"        
## [262] "quota"               "date_of_first_mail"  "METI_text"          
## [265] "summary1"            "summary2"            "version"            
## [268] "causality"           "disclaimer"          "CAMA"               
## [271] "dropout"             "accessibility_1"     "accessibility_2"    
## [274] "understanding_1"     "understanding_2"     "empowerment_1"      
## [277] "empowerment_2"       "credibility_1"       "credibility_2"      
## [280] "relevance_1"         "relevance_2"         "curiosity_1"        
## [283] "curiosity_2"         "boredom_1"           "boredom_2"          
## [286] "confusion_1"         "confusion_2"         "frustration_1"      
## [289] "frustration_2"       "s_relationship_1"    "s_relationship_2"   
## [292] "s_relationship_3"    "s_relationship_4"    "s_relationship_5"   
## [295] "s_relationship_6"    "s_relationship_7"    "s_relationship_8"   
## [298] "s_extent_1"          "s_extent_2"          "s_extent_3"         
## [301] "s_extent_4"          "s_extent_5"          "s_extent_6"         
## [304] "s_diff_1_1"          "s_diff_1_2"          "s_diff_1_3"         
## [307] "s_diff_1_4"          "s_diff_1_5"          "s_diff_1_6"         
## [310] "s_diff_2_1"          "s_diff_2_2"          "s_diff_2_3"         
## [313] "s_diff_2_4"          "s_diff_2_5"          "s_diff_2_6"         
## [316] "s_funding_1_1"       "s_funding_1_2"       "s_funding_1_3"      
## [319] "s_funding_1_4"       "s_funding_1_5"       "s_funding_1_6"      
## [322] "s_funding_2_1"       "s_funding_2_2"       "s_funding_2_3"      
## [325] "s_funding_2_4"       "s_funding_2_5"       "s_funding_2_6"      
## [328] "s_coi_1_1"           "s_coi_1_2"           "s_coi_1_3"          
## [331] "s_coi_1_4"           "s_coi_1_5"           "s_coi_1_6"          
## [334] "s_coi_1_7"           "s_coi_2_1"           "s_coi_2_2"          
## [337] "s_coi_2_3"           "s_coi_2_4"           "s_coi_2_5"          
## [340] "s_coi_2_6"           "s_coi_2_7"           "s_causality_1_1"    
## [343] "s_causality_1_2"     "s_causality_1_3"     "s_causality_1_4"    
## [346] "s_causality_1_5"     "s_causality_1_6"     "s_causality_2_1"    
## [349] "s_causality_2_2"     "s_causality_2_3"     "s_causality_2_4"    
## [352] "s_causality_2_5"     "s_causality_2_6"     "s_CAMA_1_3"         
## [355] "s_CAMA_2_3"          "s_CAMA_1_1"          "s_CAMA_1_2"         
## [358] "s_CAMA_1_4"          "s_CAMA_1_5"          "s_CAMA_1_6"         
## [361] "s_CAMA_1_7"          "s_CAMA_1_8"          "s_CAMA_2_1"         
## [364] "s_CAMA_2_2"          "s_CAMA_2_4"          "s_CAMA_3"           
## [367] "s_METI_1_exp_1"      "s_METI_1_int_1"      "s_METI_1_ben_1"     
## [370] "s_METI_1_ben_2"      "s_METI_1_ben_3"      "s_METI_1_int_2"     
## [373] "s_METI_1_exp_2"      "s_METI_1_exp_3"      "s_METI_1_exp_4"     
## [376] "s_METI_1_exp_5"      "s_METI_1_ben_4"      "s_METI_1_int_3"     
## [379] "s_METI_1_exp_6"      "s_METI_1_int_4"      "s_METI_2_exp_1"     
## [382] "s_METI_2_int_1"      "s_METI_2_ben_1"      "s_METI_2_ben_2"     
## [385] "s_METI_2_ben_3"      "s_METI_2_int_2"      "s_METI_2_exp_2"     
## [388] "s_METI_2_exp_3"      "s_METI_2_exp_4"      "s_METI_2_exp_5"     
## [391] "s_METI_2_ben_4"      "s_METI_2_int_3"      "s_METI_2_exp_6"     
## [394] "s_METI_2_int_4"      "s_METI_exp_1"        "s_METI_int_1"       
## [397] "s_METI_ben_1"        "s_METI_ben_2"        "s_METI_ben_3"       
## [400] "s_METI_int_2"        "s_METI_exp_2"        "s_METI_exp_3"       
## [403] "s_METI_exp_4"        "s_METI_exp_5"        "s_METI_ben_4"       
## [406] "s_METI_int_3"        "s_METI_exp_6"        "s_METI_int_4"       
## [409] "duration_minutes"
```

```
str(data2_wide)
```

```
## 'data.frame':    2041 obs. of  409 variables:
##  $ id                 : Factor w/ 6705 levels "1","2","3","4",..: 3600 2457 1215 3269 2531 5304 2352 170 1052 1611 ...
##  $ dispcode           : int  31 31 31 31 31 31 31 31 31 31 ...
##  $ duration           : int  779 1043 546 746 938 568 1094 1246 662 1298 ...
##  $ condition          : Factor w/ 6 levels "1","2","3","4",..: 3 4 3 5 6 2 1 6 4 4 ...
##  $ text_order         : Factor w/ 2 levels "Barth","Faerber": 1 2 1 2 1 1 2 1 1 2 ...
##  $ METI_target        : Factor w/ 2 levels "Study Authors",..: 2 1 2 1 2 1 2 1 1 1 ...
##  $ s_sex              : Factor w/ 2 levels "female","male": 2 1 2 1 2 1 1 1 2 2 ...
##  $ s_age              : int  31 31 54 45 43 51 57 26 50 35 ...
##  $ s_school           : Factor w/ 3 levels "Haupt","Real",..: 2 1 1 2 3 2 3 2 1 3 ...
##  $ s_german           : int  1 1 1 1 1 1 1 1 1 1 ...
##  $ s_psychology       : int  2 2 2 2 2 2 2 2 2 2 ...
##  $ s_interest         : int  5 5 5 4 7 7 8 7 5 5 ...
##  $ s_contact          : int  1 1 1 2 2 5 3 1 5 1 ...
##  $ s_field            : chr  NA NA NA NA ...
##  $ v_10               : int  4 NA 3 NA 5 NA NA 5 7 NA ...
##  $ v_11               : int  6 NA 2 NA 7 6 NA 7 6 NA ...
##  $ v_47               : int  3 NA 3 NA 7 6 NA 6 5 NA ...
##  $ v_48               : int  6 NA 3 NA 7 NA NA 7 5 NA ...
##  $ v_49               : int  6 NA 5 NA 7 NA NA 7 7 NA ...
##  $ v_12               : int  2 NA 3 NA 4 2 NA 3 4 NA ...
##  $ v_14               : int  1 NA 2 NA 1 4 NA 1 3 NA ...
##  $ v_16               : int  3 NA 4 NA 1 2 NA 1 3 NA ...
##  $ v_71               : int  3 NA 2 NA 1 3 NA 1 4 NA ...
##  $ v_17               : int  1 NA 3 NA 1 2 NA 1 3 NA ...
##  $ v_18               : int  1 NA 3 NA 1 1 NA 1 1 NA ...
##  $ v_19               : int  2 NA 3 NA 1 3 NA 3 3 NA ...
##  $ v_20               : int  2 NA 3 NA 1 2 NA 2 1 NA ...
##  $ v_21               : int  1 NA 3 NA 1 1 NA 1 1 NA ...
##  $ v_115              : int  1 NA 3 NA 1 NA NA 1 1 NA ...
##  $ v_116              : int  1 NA 3 NA 1 1 NA 1 1 NA ...
##  $ v_117              : int  1 NA 3 NA 1 1 NA 1 1 NA ...
##  $ v_22               : int  3 NA 3 NA 1 3 NA 2 1 NA ...
##  $ v_23               : int  1 NA 3 NA 1 1 NA 1 1 NA ...
##  $ v_24               : int  1 NA 3 NA 3 2 NA 1 1 NA ...
##  $ v_25               : int  1 NA 3 NA 1 1 NA 1 1 NA ...
##  $ v_26               : int  1 NA 3 NA 1 3 NA 3 1 NA ...
##  $ v_120              : int  1 NA 3 NA 1 2 NA 1 1 NA ...
##  $ v_27               : num  1 NA 0 NA -1 -1 NA 1 -1 NA ...
##  $ v_28               : num  1 NA 0 NA -1 1 NA -1 0 NA ...
##  $ v_29               : num  -1 NA 0 NA 1 -1 NA 1 1 NA ...
##  $ v_30               : num  1 NA 0 NA -1 1 NA -1 -1 NA ...
##  $ v_31               : num  1 NA 0 NA 1 0 NA 1 1 NA ...
##  $ v_121              : num  1 NA 0 NA 1 -1 NA -1 1 NA ...
##  $ v_32               : num  1 NA 0 NA -1 0 NA 0 -1 NA ...
##  $ v_33               : num  1 NA 0 NA -1 1 NA -1 -1 NA ...
##  $ v_34               : num  1 NA 0 NA -1 0 NA 0 -1 NA ...
##  $ v_35               : num  1 NA 0 NA -1 -1 NA -1 -1 NA ...
##  $ v_36               : num  1 NA 0 NA -1 -1 NA 0 -1 NA ...
##  $ v_122              : num  1 NA 0 NA 1 -1 NA 0 1 NA ...
##  $ v_37               : num  1 NA 0 NA -1 1 NA 1 -1 NA ...
##  $ v_38               : num  NA NA 0 NA 0 1 NA 0 0 NA ...
##  $ v_39               : num  1 NA 0 NA 0 NA NA -1 0 NA ...
##  $ v_40               : num  1 NA 0 NA -1 -1 NA -1 0 NA ...
##  $ v_41               : num  -1 NA 0 NA -1 1 NA -1 1 NA ...
##  $ v_123              : num  1 NA 0 NA 1 1 NA -1 0 NA ...
##  $ v_124              : num  1 NA 0 NA -1 1 NA 1 -1 NA ...
##  $ v_42               : num  0 NA 0 NA 1 0 NA 0 -1 NA ...
##  $ v_43               : num  -1 NA 0 NA -1 1 NA 0 0 NA ...
##  $ v_44               : num  0 NA 0 NA -1 1 NA 0 -1 NA ...
##  $ v_45               : num  -1 NA 0 NA -1 -1 NA -1 -1 NA ...
##  $ v_46               : num  0 NA 0 NA 1 -1 NA -1 0 NA ...
##  $ v_125              : num  0 NA 0 NA -1 -1 NA -1 0 NA ...
##  $ v_72               : int  NA 4 NA 4 NA NA 8 NA NA 3 ...
##  $ v_73               : int  NA 5 NA 3 NA NA 8 NA NA 5 ...
##  $ v_74               : int  NA 5 NA 3 NA NA 8 NA NA 3 ...
##  $ v_75               : int  NA 6 NA 4 NA NA 8 NA NA 8 ...
##  $ v_76               : int  NA 8 NA 6 NA NA 8 NA NA 4 ...
##  $ v_77               : int  NA 4 NA 3 NA NA 5 NA NA 2 ...
##  $ v_79               : int  NA 3 NA 2 NA NA 1 NA NA 2 ...
##  $ v_81               : int  NA 1 NA 4 NA NA 1 NA NA 2 ...
##  $ v_83               : int  NA 2 NA 2 NA NA 1 NA NA 1 ...
##  $ v_126              : int  NA 1 NA 3 NA NA 1 NA NA 1 ...
##  $ v_127              : int  NA 1 NA 3 NA NA 1 NA NA 2 ...
##  $ v_128              : int  NA 3 NA 3 NA NA 2 NA NA 2 ...
##  $ v_129              : int  NA 3 NA 3 NA NA 2 NA NA 2 ...
##  $ v_130              : int  NA 2 NA 3 NA NA 1 NA NA 1 ...
##  $ v_131              : int  NA 1 NA 1 NA NA 1 NA NA 1 ...
##  $ v_132              : int  NA 1 NA 1 NA NA 1 NA NA 2 ...
##  $ v_133              : int  NA 2 NA 3 NA NA 1 NA NA 2 ...
##  $ v_134              : int  NA 1 NA 3 NA NA 1 NA NA 2 ...
##  $ v_135              : int  NA 2 NA 3 NA NA 1 NA NA 2 ...
##  $ v_136              : int  NA 1 NA 1 NA NA 1 NA NA 2 ...
##  $ v_137              : int  NA 2 NA 2 NA NA 1 NA NA 2 ...
##  $ v_138              : int  NA 1 NA 3 NA NA 1 NA NA 2 ...
##  $ v_139              : int  NA 1 NA 3 NA NA 1 NA NA 1 ...
##  $ v_140              : logi  NA NA NA NA NA NA ...
##  $ v_141              : logi  NA NA NA NA NA NA ...
##  $ v_142              : logi  NA NA NA NA NA NA ...
##  $ v_143              : logi  NA NA NA NA NA NA ...
##  $ v_144              : logi  NA NA NA NA NA NA ...
##  $ v_145              : logi  NA NA NA NA NA NA ...
##  $ v_146              : num  NA -1 NA -1 NA NA -1 NA NA 1 ...
##  $ v_147              : num  NA -1 NA -1 NA NA -1 NA NA 1 ...
##  $ v_148              : num  NA 1 NA -1 NA NA 0 NA NA 1 ...
##  $ v_149              : num  NA -1 NA 0 NA NA 0 NA NA 1 ...
##  $ v_150              : num  NA 0 NA NA NA NA 0 NA NA 1 ...
##  $ v_151              : num  NA -1 NA 1 NA NA 1 NA NA 1 ...
##  $ v_152              : num  NA -1 NA 0 NA NA -1 NA NA 1 ...
##  $ v_153              : num  NA -1 NA 1 NA NA -1 NA NA 1 ...
##   [list output truncated]
```

```
View(data2_wide)

#Wide Dataset including only complete cases
```

## METI Scale Generation

```
psych::alpha(data2_wide[,c("s_METI_exp_1","s_METI_exp_2","s_METI_exp_3", "s_METI_exp_4", "s_METI_exp_5","s_METI_exp_6")])
```

```
## 
## Reliability analysis   
## Call: psych::alpha(x = data2_wide[, c("s_METI_exp_1", "s_METI_exp_2", 
##     "s_METI_exp_3", "s_METI_exp_4", "s_METI_exp_5", "s_METI_exp_6")])
## 
##   raw_alpha std.alpha G6(smc) average_r S/N    ase mean  sd median_r
##       0.94      0.94    0.93      0.72  16 0.0021  5.5 1.2     0.73
## 
##     95% confidence boundaries 
##          lower alpha upper
## Feldt     0.94  0.94  0.94
## Duhachek  0.94  0.94  0.94
## 
##  Reliability if an item is dropped:
##              raw_alpha std.alpha G6(smc) average_r S/N alpha se   var.r med.r
## s_METI_exp_1      0.93      0.93    0.92      0.73  13   0.0024 0.00042  0.73
## s_METI_exp_2      0.93      0.93    0.91      0.72  13   0.0025 0.00057  0.72
## s_METI_exp_3      0.93      0.93    0.92      0.73  14   0.0024 0.00024  0.73
## s_METI_exp_4      0.93      0.93    0.91      0.72  13   0.0026 0.00062  0.73
## s_METI_exp_5      0.93      0.93    0.91      0.72  13   0.0026 0.00076  0.72
## s_METI_exp_6      0.93      0.93    0.91      0.72  13   0.0025 0.00082  0.73
## 
##  Item statistics 
##                 n raw.r std.r r.cor r.drop mean  sd
## s_METI_exp_1 2030  0.87  0.87  0.83   0.80  5.5 1.4
## s_METI_exp_2 2034  0.88  0.88  0.85   0.82  5.5 1.4
## s_METI_exp_3 2034  0.86  0.86  0.82   0.79  5.3 1.4
## s_METI_exp_4 2035  0.89  0.89  0.86   0.83  5.4 1.4
## s_METI_exp_5 2029  0.89  0.89  0.86   0.83  5.5 1.4
## s_METI_exp_6 2034  0.88  0.88  0.86   0.83  5.5 1.4
## 
## Non missing response frequency for each item
##                 1    2    3    4    5    6    7 miss
## s_METI_exp_1 0.02 0.02 0.03 0.18 0.18 0.28 0.29 0.01
## s_METI_exp_2 0.02 0.02 0.04 0.18 0.18 0.29 0.29 0.00
## s_METI_exp_3 0.01 0.03 0.05 0.21 0.20 0.26 0.25 0.00
## s_METI_exp_4 0.01 0.02 0.04 0.19 0.18 0.28 0.28 0.00
## s_METI_exp_5 0.01 0.02 0.04 0.18 0.18 0.29 0.27 0.01
## s_METI_exp_6 0.01 0.02 0.04 0.18 0.18 0.29 0.29 0.00
```

```
psych::alpha(data2_wide[,c("s_METI_int_1","s_METI_int_2","s_METI_int_3", "s_METI_int_4")])
```

```
## 
## Reliability analysis   
## Call: psych::alpha(x = data2_wide[, c("s_METI_int_1", "s_METI_int_2", 
##     "s_METI_int_3", "s_METI_int_4")])
## 
##   raw_alpha std.alpha G6(smc) average_r S/N    ase mean  sd median_r
##       0.91      0.91    0.88      0.71 9.6 0.0034  5.4 1.2     0.71
## 
##     95% confidence boundaries 
##          lower alpha upper
## Feldt      0.9  0.91  0.91
## Duhachek   0.9  0.91  0.91
## 
##  Reliability if an item is dropped:
##              raw_alpha std.alpha G6(smc) average_r S/N alpha se   var.r med.r
## s_METI_int_1      0.88      0.89    0.84      0.72 7.7   0.0044 4.5e-04  0.71
## s_METI_int_2      0.88      0.88    0.83      0.70 7.1   0.0047 1.8e-03  0.71
## s_METI_int_3      0.88      0.88    0.83      0.71 7.4   0.0046 3.7e-05  0.71
## s_METI_int_4      0.87      0.87    0.82      0.69 6.8   0.0049 9.9e-04  0.71
## 
##  Item statistics 
##                 n raw.r std.r r.cor r.drop mean  sd
## s_METI_int_1 2033  0.87  0.87  0.81   0.77  5.3 1.4
## s_METI_int_2 2028  0.89  0.89  0.83   0.79  5.4 1.4
## s_METI_int_3 2029  0.88  0.88  0.83   0.78  5.5 1.4
## s_METI_int_4 2024  0.89  0.89  0.85   0.81  5.4 1.4
## 
## Non missing response frequency for each item
##                 1    2    3    4    5    6    7 miss
## s_METI_int_1 0.01 0.02 0.03 0.23 0.20 0.27 0.24 0.00
## s_METI_int_2 0.02 0.02 0.04 0.21 0.18 0.27 0.26 0.01
## s_METI_int_3 0.01 0.02 0.04 0.19 0.17 0.29 0.28 0.01
## s_METI_int_4 0.01 0.02 0.04 0.19 0.19 0.28 0.27 0.01
```

```
psych::alpha(data2_wide[,c("s_METI_ben_1","s_METI_ben_2","s_METI_ben_3", "s_METI_ben_4")])
```

```
## 
## Reliability analysis   
## Call: psych::alpha(x = data2_wide[, c("s_METI_ben_1", "s_METI_ben_2", 
##     "s_METI_ben_3", "s_METI_ben_4")])
## 
##   raw_alpha std.alpha G6(smc) average_r S/N    ase mean  sd median_r
##       0.91      0.91    0.88      0.71 9.6 0.0034  5.4 1.2     0.71
## 
##     95% confidence boundaries 
##          lower alpha upper
## Feldt      0.9  0.91  0.91
## Duhachek   0.9  0.91  0.91
## 
##  Reliability if an item is dropped:
##              raw_alpha std.alpha G6(smc) average_r S/N alpha se   var.r med.r
## s_METI_ben_1      0.87      0.87    0.82      0.70 7.0   0.0048 1.0e-04  0.70
## s_METI_ben_2      0.88      0.88    0.83      0.71 7.2   0.0047 1.6e-04  0.70
## s_METI_ben_3      0.88      0.88    0.83      0.72 7.6   0.0045 2.7e-05  0.72
## s_METI_ben_4      0.88      0.88    0.83      0.70 7.1   0.0047 1.5e-04  0.70
## 
##  Item statistics 
##                 n raw.r std.r r.cor r.drop mean  sd
## s_METI_ben_1 2027  0.89  0.89  0.84   0.80  5.3 1.4
## s_METI_ben_2 2031  0.88  0.88  0.83   0.79  5.3 1.4
## s_METI_ben_3 2032  0.88  0.88  0.81   0.77  5.5 1.4
## s_METI_ben_4 2023  0.89  0.89  0.83   0.79  5.3 1.4
## 
## Non missing response frequency for each item
##                 1    2    3    4    5    6    7 miss
## s_METI_ben_1 0.01 0.02 0.04 0.23 0.20 0.26 0.24 0.01
## s_METI_ben_2 0.02 0.02 0.04 0.22 0.20 0.26 0.25 0.00
## s_METI_ben_3 0.02 0.02 0.04 0.19 0.19 0.27 0.28 0.00
## s_METI_ben_4 0.02 0.02 0.04 0.23 0.20 0.26 0.24 0.01
```

```
data2_wide$s_METI_exp <- rowMeans(data2_wide[,c("s_METI_exp_1","s_METI_exp_2",                               "s_METI_exp_3","s_METI_exp_4",                               "s_METI_exp_5","s_METI_exp_6")])
data2_wide$s_METI_int <- rowMeans(data2_wide[,c("s_METI_int_1","s_METI_int_2",                               "s_METI_int_3","s_METI_int_4")])
data2_wide$s_METI_ben <- rowMeans(data2_wide[,c("s_METI_ben_1","s_METI_ben_2",
                       "s_METI_ben_3","s_METI_ben_4")])

describe(data2_wide$s_METI_exp)
```

```
##    vars    n mean   sd median trimmed  mad min max range  skew kurtosis   se
## X1    1 1997 5.45 1.22   5.67    5.54 1.48   1   7     6 -0.64     0.03 0.03
```

```
describe(data2_wide$s_METI_int)
```

```
##    vars    n mean   sd median trimmed  mad min max range  skew kurtosis   se
## X1    1 1999  5.4 1.23    5.5    5.48 1.48   1   7     6 -0.56    -0.04 0.03
```

```
describe(data2_wide$s_METI_ben)
```

```
##    vars    n mean   sd median trimmed  mad min max range  skew kurtosis   se
## X1    1 1996 5.35 1.23    5.5    5.42 1.48   1   7     6 -0.55     0.12 0.03
```

```
# METI Scale Reliabilities when targeting Summary Authors
data2_wide_summary_authors <- subset(data2_wide, METI_target == 
                                       "Summary Authors")

psych::alpha(data2_wide_summary_authors[,c("s_METI_exp_1","s_METI_exp_2",
                                           "s_METI_exp_3", "s_METI_exp_4",
                                           "s_METI_exp_5","s_METI_exp_6")])
```

```
## 
## Reliability analysis   
## Call: psych::alpha(x = data2_wide_summary_authors[, c("s_METI_exp_1", 
##     "s_METI_exp_2", "s_METI_exp_3", "s_METI_exp_4", "s_METI_exp_5", 
##     "s_METI_exp_6")])
## 
##   raw_alpha std.alpha G6(smc) average_r S/N    ase mean  sd median_r
##       0.94      0.94    0.93      0.73  16 0.0028  5.5 1.2     0.74
## 
##     95% confidence boundaries 
##          lower alpha upper
## Feldt     0.94  0.94  0.95
## Duhachek  0.94  0.94  0.95
## 
##  Reliability if an item is dropped:
##              raw_alpha std.alpha G6(smc) average_r S/N alpha se   var.r med.r
## s_METI_exp_1      0.93      0.94    0.92      0.74  14   0.0032 0.00074  0.75
## s_METI_exp_2      0.93      0.93    0.92      0.73  13   0.0035 0.00086  0.73
## s_METI_exp_3      0.93      0.94    0.92      0.74  14   0.0032 0.00067  0.75
## s_METI_exp_4      0.93      0.93    0.92      0.72  13   0.0035 0.00100  0.73
## s_METI_exp_5      0.93      0.93    0.91      0.72  13   0.0036 0.00128  0.72
## s_METI_exp_6      0.93      0.93    0.92      0.72  13   0.0035 0.00129  0.73
## 
##  Item statistics 
##                 n raw.r std.r r.cor r.drop mean  sd
## s_METI_exp_1 1009  0.86  0.86  0.82   0.79  5.5 1.4
## s_METI_exp_2 1012  0.88  0.88  0.86   0.83  5.5 1.4
## s_METI_exp_3 1012  0.86  0.86  0.82   0.79  5.3 1.4
## s_METI_exp_4 1010  0.89  0.89  0.87   0.84  5.4 1.4
## s_METI_exp_5 1008  0.90  0.90  0.87   0.85  5.5 1.4
## s_METI_exp_6 1009  0.89  0.89  0.87   0.84  5.5 1.4
## 
## Non missing response frequency for each item
##                 1    2    3    4    5    6    7 miss
## s_METI_exp_1 0.01 0.02 0.04 0.18 0.17 0.26 0.31 0.00
## s_METI_exp_2 0.02 0.01 0.04 0.17 0.18 0.28 0.29 0.00
## s_METI_exp_3 0.01 0.03 0.05 0.21 0.19 0.25 0.25 0.00
## s_METI_exp_4 0.01 0.02 0.04 0.19 0.18 0.27 0.28 0.00
## s_METI_exp_5 0.01 0.02 0.04 0.19 0.18 0.28 0.27 0.01
## s_METI_exp_6 0.01 0.02 0.04 0.18 0.19 0.27 0.29 0.00
```

```
psych::alpha(data2_wide_summary_authors[,c("s_METI_int_1","s_METI_int_2",
                                           "s_METI_int_3", "s_METI_int_4")])
```

```
## 
## Reliability analysis   
## Call: psych::alpha(x = data2_wide_summary_authors[, c("s_METI_int_1", 
##     "s_METI_int_2", "s_METI_int_3", "s_METI_int_4")])
## 
##   raw_alpha std.alpha G6(smc) average_r S/N    ase mean  sd median_r
##       0.91      0.91    0.89      0.72  10 0.0045  5.4 1.3     0.73
## 
##     95% confidence boundaries 
##          lower alpha upper
## Feldt      0.9  0.91  0.92
## Duhachek   0.9  0.91  0.92
## 
##  Reliability if an item is dropped:
##              raw_alpha std.alpha G6(smc) average_r S/N alpha se   var.r med.r
## s_METI_int_1      0.89      0.89    0.84      0.73 8.1   0.0060 7.0e-05  0.73
## s_METI_int_2      0.88      0.88    0.84      0.72 7.6   0.0063 1.0e-03  0.73
## s_METI_int_3      0.89      0.89    0.84      0.73 8.0   0.0060 2.1e-05  0.73
## s_METI_int_4      0.88      0.88    0.83      0.71 7.4   0.0064 7.4e-04  0.73
## 
##  Item statistics 
##                 n raw.r std.r r.cor r.drop mean  sd
## s_METI_int_1 1012  0.88  0.88  0.83   0.79  5.4 1.4
## s_METI_int_2 1008  0.89  0.89  0.84   0.81  5.4 1.4
## s_METI_int_3 1009  0.89  0.88  0.83   0.79  5.5 1.4
## s_METI_int_4 1005  0.90  0.90  0.85   0.81  5.5 1.4
## 
## Non missing response frequency for each item
##                 1    2    3    4    5    6    7 miss
## s_METI_int_1 0.01 0.02 0.03 0.23 0.18 0.26 0.26 0.00
## s_METI_int_2 0.02 0.02 0.05 0.21 0.17 0.24 0.29 0.01
## s_METI_int_3 0.01 0.02 0.04 0.19 0.16 0.28 0.30 0.00
## s_METI_int_4 0.01 0.02 0.04 0.18 0.19 0.26 0.29 0.01
```

```
psych::alpha(data2_wide_summary_authors[,c("s_METI_ben_1","s_METI_ben_2",
                                           "s_METI_ben_3", "s_METI_ben_4")])
```

```
## 
## Reliability analysis   
## Call: psych::alpha(x = data2_wide_summary_authors[, c("s_METI_ben_1", 
##     "s_METI_ben_2", "s_METI_ben_3", "s_METI_ben_4")])
## 
##   raw_alpha std.alpha G6(smc) average_r S/N    ase mean  sd median_r
##       0.91      0.91    0.89      0.72  10 0.0045  5.4 1.2     0.73
## 
##     95% confidence boundaries 
##          lower alpha upper
## Feldt      0.9  0.91  0.92
## Duhachek   0.9  0.91  0.92
## 
##  Reliability if an item is dropped:
##              raw_alpha std.alpha G6(smc) average_r S/N alpha se   var.r med.r
## s_METI_ben_1      0.88      0.88    0.84      0.72 7.7   0.0063 2.3e-04  0.73
## s_METI_ben_2      0.88      0.88    0.84      0.72 7.6   0.0064 2.2e-04  0.72
## s_METI_ben_3      0.89      0.89    0.84      0.73 8.1   0.0060 2.3e-05  0.73
## s_METI_ben_4      0.89      0.89    0.84      0.73 8.0   0.0060 7.7e-05  0.73
## 
##  Item statistics 
##                 n raw.r std.r r.cor r.drop mean  sd
## s_METI_ben_1 1008  0.89  0.89  0.85   0.81  5.4 1.4
## s_METI_ben_2 1010  0.90  0.90  0.85   0.81  5.3 1.4
## s_METI_ben_3 1011  0.88  0.88  0.83   0.79  5.5 1.4
## s_METI_ben_4 1005  0.89  0.89  0.83   0.79  5.3 1.4
## 
## Non missing response frequency for each item
##                 1    2    3    4    5    6    7 miss
## s_METI_ben_1 0.01 0.01 0.05 0.22 0.20 0.25 0.25 0.01
## s_METI_ben_2 0.01 0.02 0.05 0.21 0.19 0.26 0.26 0.00
## s_METI_ben_3 0.02 0.01 0.04 0.18 0.19 0.28 0.27 0.00
## s_METI_ben_4 0.02 0.02 0.04 0.23 0.19 0.25 0.25 0.01
```

```
# METI Scale Reliabilities when targeting Study Authors
data2_wide_study_authors <- subset(data2_wide, METI_target == "Study Authors")

psych::alpha(data2_wide_study_authors[,c("s_METI_exp_1","s_METI_exp_2",
                                           "s_METI_exp_3", "s_METI_exp_4",
                                           "s_METI_exp_5","s_METI_exp_6")])
```

```
## 
## Reliability analysis   
## Call: psych::alpha(x = data2_wide_study_authors[, c("s_METI_exp_1", 
##     "s_METI_exp_2", "s_METI_exp_3", "s_METI_exp_4", "s_METI_exp_5", 
##     "s_METI_exp_6")])
## 
##   raw_alpha std.alpha G6(smc) average_r S/N   ase mean  sd median_r
##       0.94      0.94    0.93      0.72  15 0.003  5.5 1.2     0.72
## 
##     95% confidence boundaries 
##          lower alpha upper
## Feldt     0.93  0.94  0.94
## Duhachek  0.93  0.94  0.94
## 
##  Reliability if an item is dropped:
##              raw_alpha std.alpha G6(smc) average_r S/N alpha se   var.r med.r
## s_METI_exp_1      0.93      0.93    0.91      0.72  13   0.0036 0.00032  0.72
## s_METI_exp_2      0.93      0.93    0.91      0.71  13   0.0037 0.00044  0.72
## s_METI_exp_3      0.93      0.93    0.91      0.72  13   0.0035 0.00035  0.72
## s_METI_exp_4      0.92      0.93    0.91      0.71  12   0.0037 0.00076  0.71
## s_METI_exp_5      0.93      0.93    0.91      0.71  12   0.0037 0.00074  0.71
## s_METI_exp_6      0.93      0.93    0.91      0.72  13   0.0037 0.00084  0.72
## 
##  Item statistics 
##                 n raw.r std.r r.cor r.drop mean  sd
## s_METI_exp_1 1021  0.87  0.87  0.84   0.81  5.5 1.4
## s_METI_exp_2 1022  0.88  0.88  0.85   0.82  5.5 1.4
## s_METI_exp_3 1022  0.86  0.86  0.82   0.79  5.3 1.4
## s_METI_exp_4 1025  0.88  0.88  0.85   0.83  5.4 1.4
## s_METI_exp_5 1021  0.88  0.88  0.85   0.82  5.5 1.4
## s_METI_exp_6 1025  0.87  0.87  0.84   0.82  5.5 1.3
## 
## Non missing response frequency for each item
##                 1    2    3    4    5    6    7 miss
## s_METI_exp_1 0.02 0.02 0.03 0.18 0.19 0.29 0.28 0.01
## s_METI_exp_2 0.02 0.02 0.03 0.18 0.17 0.29 0.29 0.00
## s_METI_exp_3 0.01 0.02 0.05 0.21 0.20 0.26 0.24 0.00
## s_METI_exp_4 0.01 0.02 0.03 0.19 0.19 0.29 0.27 0.00
## s_METI_exp_5 0.01 0.03 0.03 0.18 0.17 0.30 0.27 0.01
## s_METI_exp_6 0.01 0.02 0.03 0.18 0.18 0.30 0.29 0.00
```

```
psych::alpha(data2_wide_study_authors[,c("s_METI_int_1","s_METI_int_2",
                                           "s_METI_int_3", "s_METI_int_4")])
```

```
## 
## Reliability analysis   
## Call: psych::alpha(x = data2_wide_study_authors[, c("s_METI_int_1", 
##     "s_METI_int_2", "s_METI_int_3", "s_METI_int_4")])
## 
##   raw_alpha std.alpha G6(smc) average_r S/N    ase mean  sd median_r
##        0.9       0.9    0.87      0.69 8.9 0.0052  5.4 1.2     0.69
## 
##     95% confidence boundaries 
##          lower alpha upper
## Feldt     0.89   0.9  0.91
## Duhachek  0.89   0.9  0.91
## 
##  Reliability if an item is dropped:
##              raw_alpha std.alpha G6(smc) average_r S/N alpha se  var.r med.r
## s_METI_int_1      0.88      0.88    0.83      0.71 7.3   0.0066 0.0012  0.69
## s_METI_int_2      0.87      0.87    0.82      0.69 6.6   0.0071 0.0033  0.68
## s_METI_int_3      0.87      0.87    0.82      0.69 6.7   0.0070 0.0002  0.69
## s_METI_int_4      0.86      0.86    0.81      0.68 6.2   0.0075 0.0013  0.69
## 
##  Item statistics 
##                 n raw.r std.r r.cor r.drop mean  sd
## s_METI_int_1 1021  0.86  0.86  0.79   0.75  5.3 1.3
## s_METI_int_2 1020  0.88  0.88  0.82   0.78  5.3 1.4
## s_METI_int_3 1020  0.88  0.88  0.82   0.77  5.4 1.4
## s_METI_int_4 1019  0.89  0.89  0.84   0.80  5.4 1.4
## 
## Non missing response frequency for each item
##                 1    2    3    4    5    6    7 miss
## s_METI_int_1 0.01 0.02 0.03 0.22 0.22 0.28 0.22 0.01
## s_METI_int_2 0.02 0.02 0.03 0.21 0.20 0.29 0.23 0.01
## s_METI_int_3 0.01 0.02 0.04 0.19 0.18 0.30 0.26 0.01
## s_METI_int_4 0.02 0.01 0.04 0.20 0.19 0.30 0.25 0.01
```

```
psych::alpha(data2_wide_study_authors[,c("s_METI_ben_1","s_METI_ben_2",
                                           "s_METI_ben_3", "s_METI_ben_4")])
```

```
## 
## Reliability analysis   
## Call: psych::alpha(x = data2_wide_study_authors[, c("s_METI_ben_1", 
##     "s_METI_ben_2", "s_METI_ben_3", "s_METI_ben_4")])
## 
##   raw_alpha std.alpha G6(smc) average_r S/N    ase mean  sd median_r
##        0.9       0.9    0.87      0.69 8.9 0.0052  5.3 1.2     0.69
## 
##     95% confidence boundaries 
##          lower alpha upper
## Feldt     0.89   0.9  0.91
## Duhachek  0.89   0.9  0.91
## 
##  Reliability if an item is dropped:
##              raw_alpha std.alpha G6(smc) average_r S/N alpha se   var.r med.r
## s_METI_ben_1      0.86      0.86    0.81      0.68 6.4   0.0073 5.6e-04  0.69
## s_METI_ben_2      0.87      0.87    0.82      0.70 6.9   0.0068 1.3e-04  0.69
## s_METI_ben_3      0.88      0.88    0.82      0.70 7.0   0.0067 8.3e-05  0.70
## s_METI_ben_4      0.86      0.86    0.81      0.68 6.4   0.0073 5.7e-04  0.69
## 
##  Item statistics 
##                 n raw.r std.r r.cor r.drop mean  sd
## s_METI_ben_1 1019  0.88  0.88  0.83   0.79  5.3 1.4
## s_METI_ben_2 1021  0.87  0.87  0.80   0.76  5.3 1.4
## s_METI_ben_3 1021  0.87  0.87  0.80   0.76  5.5 1.4
## s_METI_ben_4 1018  0.88  0.88  0.83   0.79  5.3 1.4
## 
## Non missing response frequency for each item
##                 1    2    3    4    5    6    7 miss
## s_METI_ben_1 0.01 0.02 0.04 0.24 0.19 0.26 0.23 0.01
## s_METI_ben_2 0.02 0.01 0.03 0.23 0.20 0.27 0.24 0.01
## s_METI_ben_3 0.02 0.02 0.04 0.19 0.19 0.27 0.28 0.01
## s_METI_ben_4 0.02 0.02 0.04 0.22 0.21 0.27 0.23 0.01
```

### CFA METI

```
meti_mod1 <- "trust =~ s_METI_exp_1 + s_METI_exp_2 + s_METI_exp_3 + s_METI_exp_4
+ s_METI_exp_5 + s_METI_exp_6 + s_METI_int_1 + s_METI_int_2 + s_METI_int_3 +
s_METI_int_4 + s_METI_ben_1 + s_METI_ben_2 + s_METI_ben_3 + s_METI_ben_4"
meti_fit1 <- cfa(meti_mod1, data = data2_wide)
fit_1 <- fitmeasures(meti_fit1)[c("chisq","df","tli","cfi","rmsea","srmr")]

meti_mod2 <- "exp =~ s_METI_exp_1 + s_METI_exp_2 + s_METI_exp_3 + s_METI_exp_4
+ s_METI_exp_5 + s_METI_exp_6
intben =~ s_METI_int_1 + s_METI_int_2 + s_METI_int_3 +
s_METI_int_4 + s_METI_ben_1 + s_METI_ben_2 + s_METI_ben_3 + s_METI_ben_4"
meti_fit2 <- cfa(meti_mod2, data = data2_wide)
fit_2 <- fitmeasures(meti_fit2)[c("chisq","df","tli","cfi","rmsea","srmr")]

meti_mod3 <- "exp =~ s_METI_exp_1 + s_METI_exp_2 + s_METI_exp_3 + s_METI_exp_4
+ s_METI_exp_5 + s_METI_exp_6
int =~ s_METI_int_1 + s_METI_int_2 + s_METI_int_3 + s_METI_int_4
ben =~ s_METI_ben_1 + s_METI_ben_2 + s_METI_ben_3 + s_METI_ben_4"
meti_fit3 <- cfa(meti_mod3, data = data2_wide)
fit_3 <- fitmeasures(meti_fit3)[c("chisq","df","tli","cfi","rmsea","srmr")]

anova(meti_fit1,meti_fit2,meti_fit3)
```

```
## 
## Chi-Squared Difference Test
## 
##           Df   AIC   BIC  Chisq Chisq diff   RMSEA Df diff Pr(>Chisq)    
## meti_fit3 74 69189 69361 341.85                                          
## meti_fit2 76 69208 69369 364.96      23.11 0.07382       2  9.588e-06 ***
## meti_fit1 77 69437 69593 595.90     230.94 0.34454       1  < 2.2e-16 ***
## ---
## Signif. codes:  0 '***' 0.001 '**' 0.01 '*' 0.05 '.' 0.1 ' ' 1
```

## Create Overall Scores for Knowledge Items

### Relationship-Item

```
data2_wide$s_relationship <- rowSums(data2_wide[,c("s_relationship_1",
                                                   "s_relationship_2",
                                                   "s_relationship_3",
                                                   "s_relationship_4",
                                                   "s_relationship_5",
                                                   "s_relationship_6",
                                                   "s_relationship_7",
                                                   "s_relationship_8")])
describe(data2_wide$s_relationship)
```

```
##    vars    n mean   sd median trimmed  mad min max range skew kurtosis   se
## X1    1 1979 0.23 3.22      0    0.02 2.97  -8   8    16 0.45    -0.37 0.07
```

```
table(data2_wide$s_relationship)
```

```
## 
##  -8  -7  -6  -5  -4  -3  -2  -1   0   1   2   3   4   5   6   7   8 
##   4   1  20  20 247 110 251 163 425 112 192  67 141  30 135  17  44
```

### Extent of Evaluation-Item

```
data2_wide$s_extent <- rowSums(data2_wide[,c("s_extent_1","s_extent_2",                                   "s_extent_3","s_extent_4",                                   "s_extent_5","s_extent_6")])
describe(data2_wide$s_extent)
```

```
##    vars    n mean   sd median trimmed  mad min max range skew kurtosis   se
## X1    1 1999 0.52 2.28      0    0.41 2.97  -6   6    12 0.35    -0.28 0.05
```

```
table(data2_wide$s_extent)
```

```
## 
##  -6  -5  -4  -3  -2  -1   0   1   2   3   4   5   6 
##   4   2  45  52 345 180 533 179 281 118 168  35  57
```

### Differentiation-Item

```
data2_wide$s_diff_1 <- rowSums(data2_wide[,c("s_diff_1_1","s_diff_1_2",                                       "s_diff_1_3","s_diff_1_4",
                      "s_diff_1_5","s_diff_1_6")])
describe(data2_wide$s_diff_1)
```

```
##    vars   n mean   sd median trimmed  mad min max range skew kurtosis   se
## X1    1 997 0.07 1.83      0    0.05 1.48  -6   6    12 0.14     0.68 0.06
```

```
table(data2_wide$s_diff_1)
```

```
## 
##  -6  -4  -3  -2  -1   0   1   2   3   4   5   6 
##   3  33  22 145 103 360 117 146  22  33   7   6
```

```
data2_wide$s_diff_2 <- rowSums(data2_wide[,c("s_diff_2_1","s_diff_2_2",                                       "s_diff_2_3","s_diff_2_4",
                      "s_diff_2_5","s_diff_2_6")])
describe(data2_wide$s_diff_2)
```

```
##    vars   n mean   sd median trimmed  mad min max range skew kurtosis   se
## X1    1 981 0.52 2.07      0    0.46 2.97  -6   6    12 0.15     0.23 0.07
```

```
table(data2_wide$s_diff_2)
```

```
## 
##  -6  -5  -4  -3  -2  -1   0   1   2   3   4   5   6 
##   2   3  29  15 123  61 343  82 191  31  74  10  17
```

### Funding-Item

```
data2_wide$s_funding_1 <- rowSums(data2_wide[,c("s_funding_1_1","s_funding_1_2",
                      "s_funding_1_3","s_funding_1_4",                             "s_funding_1_5","s_funding_1_6")])
describe(data2_wide$s_funding_1)
```

```
##    vars    n mean   sd median trimmed  mad min max range skew kurtosis   se
## X1    1 1994 0.87 3.05      0    0.84 2.97  -6   6    12 0.26    -0.74 0.07
```

```
table(data2_wide$s_funding_1)
```

```
## 
##  -6  -5  -4  -3  -2  -1   0   1   2   3   4   5   6 
##  13  10 152  54 215 175 511 140 181  84 115  48 296
```

```
data2_wide$s_funding_2 <- rowSums(data2_wide[,c("s_funding_2_1","s_funding_2_2",
                      "s_funding_2_3","s_funding_2_4",
                      "s_funding_2_5","s_funding_2_6")])
describe(data2_wide$s_funding_2)
```

```
##    vars    n mean   sd median trimmed  mad min max range  skew kurtosis   se
## X1    1 2010 1.67 3.22      1    1.82 4.45  -6   6    12 -0.07    -1.03 0.07
```

```
table(data2_wide$s_funding_2)
```

```
## 
##  -6  -5  -4  -3  -2  -1   0   1   2   3   4   5   6 
##  12  12 119  46 157 103 474 118 206  59 177  48 479
```

### COI-Item

```
data2_wide$s_coi_1 <- rowSums(data2_wide[,c("s_coi_1_1","s_coi_1_2",
                      "s_coi_1_3","s_coi_1_4",
                      "s_coi_1_5","s_coi_1_6", "s_coi_1_7")])
describe(data2_wide$s_coi_1)
```

```
##    vars    n mean   sd median trimmed  mad min max range skew kurtosis   se
## X1    1 1955 0.72 3.33      0    0.66 2.97  -7   7    14 0.19     -0.5 0.08
```

```
table(data2_wide$s_coi_1)
```

```
## 
##  -7  -6  -5  -4  -3  -2  -1   0   1   2   3   4   5   6   7 
##   9   8 137  51 133 101 182 467 208  83 187  47 148  22 172
```

```
data2_wide$s_coi_2 <- rowSums(data2_wide[,c("s_coi_2_1","s_coi_2_2",
                      "s_coi_2_3","s_coi_2_4",                                     "s_coi_2_5","s_coi_2_6","s_coi_2_7")])
describe(data2_wide$s_coi_2)
```

```
##    vars    n mean   sd median trimmed  mad min max range  skew kurtosis   se
## X1    1 1993  1.3 3.56      1    1.36 2.97  -7   7    14 -0.01    -0.75 0.08
```

```
table(data2_wide$s_coi_2)
```

```
## 
##  -7  -6  -5  -4  -3  -2  -1   0   1   2   3   4   5   6   7 
##  18  14 114  37 123  72 182 407 201  69 207  33 235  22 259
```

### Causality-Item

```
data2_wide$s_causality_1 <- rowSums(data2_wide[,c("s_causality_1_1","s_causality_1_2",
                      "s_causality_1_3","s_causality_1_4",
                      "s_causality_1_5","s_causality_1_6")])
describe(data2_wide$s_causality_1)
```

```
##    vars    n  mean   sd median trimmed  mad min max range skew kurtosis   se
## X1    1 1989 -0.43 2.45      0   -0.52 2.97  -6   6    12  0.2    -0.56 0.06
```

```
table(data2_wide$s_causality_1)
```

```
## 
##  -6  -5  -4  -3  -2  -1   0   1   2   3   4   5   6 
##   8   7 297 112 295 173 493 116 266  68 121  14  19
```

```
data2_wide$s_causality_2 <- rowSums(data2_wide[,c("s_causality_2_1","s_causality_2_2",
                      "s_causality_2_3","s_causality_2_4",
                      "s_causality_2_5","s_causality_2_6")])
describe(data2_wide$s_causality_2)
```

```
##    vars    n  mean   sd median trimmed  mad min max range skew kurtosis   se
## X1    1 1995 -0.21 2.45      0   -0.29 2.97  -6   6    12 0.21    -0.31 0.05
```

```
table(data2_wide$s_causality_2)
```

```
## 
##  -6  -5  -4  -3  -2  -1   0   1   2   3   4   5   6 
##   8   7 247 100 267 173 541 149 263  59 131   9  41
```

### CAMA-Items

```
data2_wide$s_CAMA_1 <- rowSums(data2_wide[,c("s_CAMA_1_1","s_CAMA_1_2",
                      "s_CAMA_1_3","s_CAMA_1_4",
                      "s_CAMA_1_5","s_CAMA_1_6",
                      "s_CAMA_1_7","s_CAMA_1_8")])
describe(data2_wide$s_CAMA_1)
```

```
##    vars   n mean   sd median trimmed  mad min max range skew kurtosis   se
## X1    1 984  0.7 2.66      0    0.68 2.97  -7   8    15 0.16    -0.02 0.08
```

```
table(data2_wide$s_CAMA_1)
```

```
## 
##  -7  -6  -5  -4  -3  -2  -1   0   1   2   3   4   5   6   7   8 
##   1   5   5  69  38  66  46 318 100 110  61  85  25  41   7   7
```

```
data2_wide$s_CAMA_2 <- rowSums(data2_wide[,c("s_CAMA_2_1","s_CAMA_2_2",
                      "s_CAMA_2_3","s_CAMA_2_4")])
describe(data2_wide$s_CAMA_2)
```

```
##    vars    n mean   sd median trimmed  mad min max range skew kurtosis   se
## X1    1 1015 -0.4 1.62      0   -0.44 1.48  -4   4     8 0.18     0.05 0.05
```

```
table(data2_wide$s_CAMA_2)
```

```
## 
##  -4  -3  -2  -1   0   1   2   3   4 
##  32  36 239  99 397  67 112  14  19
```

```
describe(data2_wide$s_CAMA_3)
```

```
##    vars    n  mean   sd median trimmed  mad min max range skew kurtosis   se
## X1    1 1026 -0.14 0.75      0   -0.17 1.48  -1   1     2 0.23    -1.21 0.02
```

```
table(data2_wide$s_CAMA_3)
```

```
## 
##  -1   0   1 
## 369 428 229
```

```
data2_wide$s_CAMA <- rowSums(data2_wide[,c("s_CAMA_1","s_CAMA_2","s_CAMA_3")])
describe(data2_wide$s_CAMA)
```

```
##    vars   n mean   sd median trimmed  mad min max range skew kurtosis   se
## X1    1 975 0.17 3.73      0    0.17 2.97 -11  13    24 0.09     0.13 0.12
```

```
table(data2_wide$s_CAMA)
```

```
## 
## -11  -9  -8  -7  -6  -5  -4  -3  -2  -1   0   1   2   3   4   5   6   7   8   9 
##   1   4   1  50  26  43  25  49  68  93 230  89  50  67  49  55  23  26   8  12 
##  10  11  12  13 
##   1   3   1   1
```

## Melt Dataframe into Long Format

```
data2_long <- melt.data.table(setDT(data2_wide),measure.vars = 
                                list(c("summary1","summary2"),
                                     c("accessibility_1","accessibility_2"),
                                     c("understanding_1","understanding_2"),
                                     c("empowerment_1","empowerment_2"),
                                     c("credibility_1","credibility_2"),
                                     c("relevance_1","relevance_2"),
                                     c("curiosity_1","curiosity_2"),
                                     c("boredom_1","boredom_2"),
                                     c("frustration_1","frustration_2"),
                                     c("confusion_1","confusion_2"),
                                     c("s_funding_1","s_funding_2"),
                                     c("s_coi_1","s_coi_2"),
c("s_diff_1","s_diff_2"),                                     c("s_causality_1","s_causality_2")),
                              value.name = c("summary","accessibility",
                                             "understanding","empowerment",
                                             "credibility","relevance",
                                             "curiosity",
                                             "boredom","frustration",
                                             "confusion","s_funding",                                             "s_coi","s_diff","s_causality"),
                              variable.name = "Time_point")

View(data2_long)

data2_long <- dplyr::select(data2_long, -c(15:260,263))
View(data2_long)

data2_long$summary <- factor(data2_long$summary, levels = c("Barth","Faerber"))
```

## User Experience Scale Generation

```
psych::alpha(data2_long[,c("accessibility","understanding","empowerment")])
```

```
## 
## Reliability analysis   
## Call: psych::alpha(x = data2_long[, c("accessibility", "understanding", 
##     "empowerment")])
## 
##   raw_alpha std.alpha G6(smc) average_r S/N    ase mean  sd median_r
##       0.83      0.83    0.77      0.62   5 0.0046  5.3 1.5     0.66
## 
##     95% confidence boundaries 
##          lower alpha upper
## Feldt     0.82  0.83  0.84
## Duhachek  0.82  0.83  0.84
## 
##  Reliability if an item is dropped:
##               raw_alpha std.alpha G6(smc) average_r S/N alpha se var.r med.r
## accessibility      0.79      0.79    0.66      0.66 3.8   0.0065    NA  0.66
## understanding      0.72      0.72    0.56      0.56 2.5   0.0089    NA  0.56
## empowerment        0.79      0.79    0.66      0.66 3.8   0.0065    NA  0.66
## 
##  Item statistics 
##                  n raw.r std.r r.cor r.drop mean  sd
## accessibility 4045  0.86  0.85  0.73   0.67  5.5 1.8
## understanding 4038  0.89  0.89  0.82   0.74  5.6 1.7
## empowerment   4039  0.85  0.85  0.73   0.67  4.8 1.8
## 
## Non missing response frequency for each item
##                  1    2    3    4    5    6    7    8 miss
## accessibility 0.03 0.03 0.08 0.15 0.17 0.20 0.16 0.18 0.01
## understanding 0.02 0.03 0.07 0.14 0.19 0.22 0.17 0.15 0.01
## empowerment   0.06 0.06 0.12 0.19 0.22 0.19 0.09 0.08 0.01
```

### CFA User Experience

```
UEmodel <- "outcome =~ c(a)*accessibility + c(a)*understanding + c(a)*empowerment
accessibility ~~ c(b)*empowerment"

UEfit <- sem(UEmodel, data = data2_long, estimator = "MLR", missing = "ML",
             std.lv = T, fixed.x = F, group = "summary")
```

```
## Warning in lav_data_full(data = data, group = group, cluster = cluster, : lavaan WARNING: some cases are empty and will be ignored:
##   718 1417 1965 2985 3986
```

```
## Warning in lav_data_full(data = data, group = group, cluster = cluster, : lavaan WARNING: some cases are empty and will be ignored:
##   1975
```

```
## Warning in lavaanify(model = FLAT, constraints = constraints, varTable = DataOV, : lavaan WARNING: using a single label per parameter in a multiple group
##   setting implies imposing equality constraints across all the groups;
##   If this is not intended, either remove the label(s), or use a vector
##   of labels (one for each group);
##   See the Multiple groups section in the man page of model.syntax.
```

```
summary(UEfit, standardized = T)
```

```
## lavaan 0.6.16 ended normally after 36 iterations
## 
##   Estimator                                         ML
##   Optimization method                           NLMINB
##   Number of model parameters                        20
##   Number of equality constraints                     6
## 
##   Number of observations per group:               Used       Total
##     Barth                                         2036        2041
##     Faerber                                       2040        2041
##   Number of missing patterns per group:                           
##     Barth                                            6            
##     Faerber                                          7            
## 
## Model Test User Model:
##                                               Standard      Scaled
##   Test Statistic                                 3.534       3.167
##   Degrees of freedom                                 4           4
##   P-value (Chi-square)                           0.473       0.530
##   Scaling correction factor                                  1.116
##     Yuan-Bentler correction (Mplus variant)                       
##   Test statistic for each group:
##     Barth                                        1.326       1.188
##     Faerber                                      2.208       1.978
## 
## Parameter Estimates:
## 
##   Standard errors                             Sandwich
##   Information bread                           Observed
##   Observed information based on                Hessian
## 
## 
## Group 1 [Barth]:
## 
## Latent Variables:
##                    Estimate  Std.Err  z-value  P(>|z|)   Std.lv  Std.all
##   outcome =~                                                            
##     accessblty (a)    1.428    0.018   78.985    0.000    1.428    0.782
##     undrstndng (a)    1.428    0.018   78.985    0.000    1.428    0.834
##     empowermnt (a)    1.428    0.018   78.985    0.000    1.428    0.789
## 
## Covariances:
##                    Estimate  Std.Err  z-value  P(>|z|)   Std.lv  Std.all
##  .accessibility ~~                                                      
##    .empowermnt (b)   -0.204    0.039   -5.276    0.000   -0.204   -0.161
## 
## Intercepts:
##                    Estimate  Std.Err  z-value  P(>|z|)   Std.lv  Std.all
##    .accessibility     5.543    0.040  137.942    0.000    5.543    3.034
##    .understanding     5.646    0.037  150.746    0.000    5.646    3.295
##    .empowerment       4.839    0.040  121.599    0.000    4.839    2.674
##     outcome           0.000                               0.000    0.000
## 
## Variances:
##                    Estimate  Std.Err  z-value  P(>|z|)   Std.lv  Std.all
##    .accessibility     1.299    0.072   18.046    0.000    1.299    0.389
##    .understanding     0.896    0.057   15.604    0.000    0.896    0.305
##    .empowerment       1.236    0.071   17.419    0.000    1.236    0.377
##     outcome           1.000                               1.000    1.000
## 
## 
## Group 2 [Faerber]:
## 
## Latent Variables:
##                    Estimate  Std.Err  z-value  P(>|z|)   Std.lv  Std.all
##   outcome =~                                                            
##     accessblty (a)    1.428    0.018   78.985    0.000    1.428    0.787
##     undrstndng (a)    1.428    0.018   78.985    0.000    1.428    0.838
##     empowermnt (a)    1.428    0.018   78.985    0.000    1.428    0.786
## 
## Covariances:
##                    Estimate  Std.Err  z-value  P(>|z|)   Std.lv  Std.all
##  .accessibility ~~                                                      
##    .empowermnt (b)   -0.204    0.039   -5.276    0.000   -0.204   -0.162
## 
## Intercepts:
##                    Estimate  Std.Err  z-value  P(>|z|)   Std.lv  Std.all
##    .accessibility     5.528    0.041  135.085    0.000    5.528    3.045
##    .understanding     5.528    0.038  143.776    0.000    5.528    3.242
##    .empowerment       4.717    0.040  116.604    0.000    4.717    2.597
##     outcome           0.000                               0.000    0.000
## 
## Variances:
##                    Estimate  Std.Err  z-value  P(>|z|)   Std.lv  Std.all
##    .accessibility     1.256    0.074   17.074    0.000    1.256    0.381
##    .understanding     0.867    0.050   17.364    0.000    0.867    0.298
##    .empowerment       1.261    0.068   18.628    0.000    1.261    0.382
##     outcome           1.000                               1.000    1.000
```

```
modificationindices(UEfit)
```

```
##              lhs op           rhs block group level    mi    epc sepc.lv
## 8        outcome ~~       outcome     1     1     1 1.962 -0.073  -1.000
## 20       outcome ~~       outcome     2     2     1 1.962  0.073   1.000
## 31 accessibility ~~ understanding     1     1     1 0.337 -0.034  -0.034
## 32 understanding ~~   empowerment     1     1     1 0.425 -0.038  -0.038
## 33 accessibility ~~ understanding     2     2     1 2.234  0.086   0.086
## 34 understanding ~~   empowerment     2     2     1 0.072 -0.016  -0.016
##    sepc.all sepc.nox
## 8    -1.000   -1.000
## 20    1.000    1.000
## 31   -0.031   -0.031
## 32   -0.036   -0.036
## 33    0.083    0.083
## 34   -0.015   -0.015
```

```
fitmeasures(UEfit)
```

```
##                          npar                          fmin 
##                        14.000                         0.000 
##                         chisq                            df 
##                         3.534                         4.000 
##                        pvalue                  chisq.scaled 
##                         0.473                         3.167 
##                     df.scaled                 pvalue.scaled 
##                         4.000                         0.530 
##          chisq.scaling.factor                baseline.chisq 
##                         1.116                      4732.569 
##                   baseline.df               baseline.pvalue 
##                         6.000                         0.000 
##         baseline.chisq.scaled            baseline.df.scaled 
##                      2413.601                         6.000 
##        baseline.pvalue.scaled baseline.chisq.scaling.factor 
##                         0.000                         1.961 
##                           cfi                           tli 
##                         1.000                         1.000 
##                    cfi.scaled                    tli.scaled 
##                         1.000                         1.001 
##                    cfi.robust                    tli.robust 
##                         1.000                         1.000 
##                          nnfi                           rfi 
##                         1.000                         0.999 
##                           nfi                          pnfi 
##                         0.999                         0.666 
##                           ifi                           rni 
##                         1.000                         1.000 
##                   nnfi.scaled                    rfi.scaled 
##                         1.001                         0.998 
##                    nfi.scaled                   pnfi.scaled 
##                         0.999                         0.666 
##                    ifi.scaled                    rni.scaled 
##                         1.000                         1.000 
##                   nnfi.robust                    rni.robust 
##                         1.000                         1.000 
##                          logl             unrestricted.logl 
##                    -21827.855                    -21826.088 
##                           aic                           bic 
##                     43683.710                     43772.090 
##                        ntotal                          bic2 
##                      4076.000                     43727.604 
##             scaling.factor.h1             scaling.factor.h0 
##                         1.245                         0.898 
##                         rmsea                rmsea.ci.lower 
##                         0.000                         0.000 
##                rmsea.ci.upper                rmsea.ci.level 
##                         0.032                         0.900 
##                  rmsea.pvalue                rmsea.close.h0 
##                         0.999                         0.050 
##         rmsea.notclose.pvalue             rmsea.notclose.h0 
##                         0.000                         0.080 
##                  rmsea.scaled         rmsea.ci.lower.scaled 
##                         0.000                         0.000 
##         rmsea.ci.upper.scaled           rmsea.pvalue.scaled 
##                         0.029                         1.000 
##  rmsea.notclose.pvalue.scaled                  rmsea.robust 
##                         0.000                         0.000 
##         rmsea.ci.lower.robust         rmsea.ci.upper.robust 
##                         0.000                         0.032 
##           rmsea.pvalue.robust  rmsea.notclose.pvalue.robust 
##                         0.999                         0.000 
##                           rmr                    rmr_nomean 
##                         0.062                         0.076 
##                          srmr                  srmr_bentler 
##                         0.020                         0.020 
##           srmr_bentler_nomean                          crmr 
##                         0.024                         0.006 
##                   crmr_nomean                    srmr_mplus 
##                         0.009                         0.027 
##             srmr_mplus_nomean                         cn_05 
##                         0.019                     10943.881 
##                         cn_01                           gfi 
##                     15313.979                         1.000 
##                          agfi                          pgfi 
##                         1.000                         0.222 
##                           mfi                          ecvi 
##                         1.000                         0.008
```

# Descriptive Analyses

## Participants’ Gender

```
table(data2_wide$s_sex)
```

```
## 
## female   male 
##   1028   1013
```

```
prop.table(table(data2_wide$s_sex))
```

```
## 
##    female      male 
## 0.5036747 0.4963253
```

## Participants’ Age

```
describe(data2_wide$s_age)
```

```
##    vars    n  mean    sd median trimmed   mad min max range skew kurtosis   se
## X1    1 2040 45.22 15.23     45   45.01 17.79  18  90    72 0.12    -0.96 0.34
```

```
age.hist <- ggplot(data2_wide, aes(s_age)) + geom_histogram(colour = "black",
                                                            fill = "white")+
  labs(x = "Age", y = "Frequency")
age.hist
```

```
## `stat_bin()` using `bins = 30`. Pick better value with `binwidth`.
```

```
## Warning: Removed 1 rows containing non-finite values (`stat_bin()`).
```

```
data2_wide$age_group <- ifelse(data2_wide$s_age < 45, "low", "high")
data2_wide$age_group <- as.factor(data2_wide$age_group)
table(data2_wide$age_group)
```

```
## 
## high  low 
## 1024 1016
```

```
prop.table(table(data2_wide$age_group))
```

```
## 
##      high       low 
## 0.5019608 0.4980392
```

## Participant’s Educational Background

```
table(data2_wide$s_school)
```

```
## 
## Haupt  Real   Abi 
##   685   681   675
```

```
prop.table(table(data2_wide$s_school))
```

```
## 
##     Haupt      Real       Abi 
## 0.3356198 0.3336600 0.3307202
```

## Quota

```
table(data2_wide$quota)
```

```
## 
##   1   2   3   4   5   6   7   8   9  10  11  12 
## 169 171 174 168 171 172 168 167 164 170 172 175
```

## Awareness Check

```
table(data2_wide$s_awareness)
```

```
## 
## fail pass 
##  658 1383
```

```
prop.table(table(data2_wide$s_awareness))
```

```
## 
##     fail     pass 
## 0.322391 0.677609
```

```
table(data2_wide$s_awareness, data2_wide$condition)
```

```
##       
##          1   2   3   4   5   6
##   fail 113  94 116 135  90 110
##   pass 221 251 220 206 238 247
```

```
awareness_bar <- ggplot(data2_wide, aes(x = condition, fill = s_awareness))
awareness_bar <- awareness_bar + geom_bar() + theme_classic() + theme(
  panel.grid.major = element_blank(),
  panel.grid.minor = element_blank(),
  panel.background = element_blank(),
  axis.title = element_text(face = "bold"),
  text = element_text(face = "bold"),
  axis.text = element_text(face = "bold"),
  legend.title = element_text(face = "bold"))+
  labs(x = "Condition", y = "Number of Cases", fill = "Awareness Check") +
  scale_fill_brewer(palette = "Blues")
awareness_bar
```

```
CrossTable(data2_wide$condition, data2_wide$s_awareness,
           chisq = TRUE, expected = TRUE, sresid = TRUE, format = "SPSS")
```

```
## 
##    Cell Contents
## |-------------------------|
## |                   Count |
## |         Expected Values |
## | Chi-square contribution |
## |             Row Percent |
## |          Column Percent |
## |           Total Percent |
## |            Std Residual |
## |-------------------------|
## 
## Total Observations in Table:  2041 
## 
##                      | data2_wide$s_awareness 
## data2_wide$condition |     fail  |     pass  | Row Total | 
## ---------------------|-----------|-----------|-----------|
##                    1 |      113  |      221  |      334  | 
##                      |  107.679  |  226.321  |           | 
##                      |    0.263  |    0.125  |           | 
##                      |   33.832% |   66.168% |   16.365% | 
##                      |   17.173% |   15.980% |           | 
##                      |    5.537% |   10.828% |           | 
##                      |    0.513  |   -0.354  |           | 
## ---------------------|-----------|-----------|-----------|
##                    2 |       94  |      251  |      345  | 
##                      |  111.225  |  233.775  |           | 
##                      |    2.668  |    1.269  |           | 
##                      |   27.246% |   72.754% |   16.903% | 
##                      |   14.286% |   18.149% |           | 
##                      |    4.606% |   12.298% |           | 
##                      |   -1.633  |    1.127  |           | 
## ---------------------|-----------|-----------|-----------|
##                    3 |      116  |      220  |      336  | 
##                      |  108.323  |  227.677  |           | 
##                      |    0.544  |    0.259  |           | 
##                      |   34.524% |   65.476% |   16.463% | 
##                      |   17.629% |   15.907% |           | 
##                      |    5.683% |   10.779% |           | 
##                      |    0.738  |   -0.509  |           | 
## ---------------------|-----------|-----------|-----------|
##                    4 |      135  |      206  |      341  | 
##                      |  109.935  |  231.065  |           | 
##                      |    5.715  |    2.719  |           | 
##                      |   39.589% |   60.411% |   16.707% | 
##                      |   20.517% |   14.895% |           | 
##                      |    6.614% |   10.093% |           | 
##                      |    2.391  |   -1.649  |           | 
## ---------------------|-----------|-----------|-----------|
##                    5 |       90  |      238  |      328  | 
##                      |  105.744  |  222.256  |           | 
##                      |    2.344  |    1.115  |           | 
##                      |   27.439% |   72.561% |   16.071% | 
##                      |   13.678% |   17.209% |           | 
##                      |    4.410% |   11.661% |           | 
##                      |   -1.531  |    1.056  |           | 
## ---------------------|-----------|-----------|-----------|
##                    6 |      110  |      247  |      357  | 
##                      |  115.094  |  241.906  |           | 
##                      |    0.225  |    0.107  |           | 
##                      |   30.812% |   69.188% |   17.491% | 
##                      |   16.717% |   17.860% |           | 
##                      |    5.390% |   12.102% |           | 
##                      |   -0.475  |    0.327  |           | 
## ---------------------|-----------|-----------|-----------|
##         Column Total |      658  |     1383  |     2041  | 
##                      |   32.239% |   67.761% |           | 
## ---------------------|-----------|-----------|-----------|
## 
##  
## Statistics for All Table Factors
## 
## 
## Pearson's Chi-squared test 
## ------------------------------------------------------------
## Chi^2 =  17.35328     d.f. =  5     p =  0.003876309 
## 
## 
##  
##        Minimum expected frequency: 105.7442
```

```
fisher.test(data2_wide$condition, data2_wide$s_awareness, workspace = 2e8)
```

```
## 
##  Fisher's Exact Test for Count Data
## 
## data:  data2_wide$condition and data2_wide$s_awareness
## p-value = 0.004063
## alternative hypothesis: two.sided
```

```
CrossTable(data2_wide$s_sex, data2_wide$s_awareness,
           chisq = TRUE, expected = TRUE, sresid = TRUE, format = "SPSS")
```

```
## 
##    Cell Contents
## |-------------------------|
## |                   Count |
## |         Expected Values |
## | Chi-square contribution |
## |             Row Percent |
## |          Column Percent |
## |           Total Percent |
## |            Std Residual |
## |-------------------------|
## 
## Total Observations in Table:  2041 
## 
##                  | data2_wide$s_awareness 
## data2_wide$s_sex |     fail  |     pass  | Row Total | 
## -----------------|-----------|-----------|-----------|
##           female |      301  |      727  |     1028  | 
##                  |  331.418  |  696.582  |           | 
##                  |    2.792  |    1.328  |           | 
##                  |   29.280% |   70.720% |   50.367% | 
##                  |   45.745% |   52.567% |           | 
##                  |   14.748% |   35.620% |           | 
##                  |   -1.671  |    1.153  |           | 
## -----------------|-----------|-----------|-----------|
##             male |      357  |      656  |     1013  | 
##                  |  326.582  |  686.418  |           | 
##                  |    2.833  |    1.348  |           | 
##                  |   35.242% |   64.758% |   49.633% | 
##                  |   54.255% |   47.433% |           | 
##                  |   17.491% |   32.141% |           | 
##                  |    1.683  |   -1.161  |           | 
## -----------------|-----------|-----------|-----------|
##     Column Total |      658  |     1383  |     2041  | 
##                  |   32.239% |   67.761% |           | 
## -----------------|-----------|-----------|-----------|
## 
##  
## Statistics for All Table Factors
## 
## 
## Pearson's Chi-squared test 
## ------------------------------------------------------------
## Chi^2 =  8.30114     d.f. =  1     p =  0.003962019 
## 
## Pearson's Chi-squared test with Yates' continuity correction 
## ------------------------------------------------------------
## Chi^2 =  8.030481     d.f. =  1     p =  0.004599664 
## 
##  
##        Minimum expected frequency: 326.5821
```

```
fisher.test(data2_wide$s_sex, data2_wide$s_awareness, workspace = 2e8)
```

```
## 
##  Fisher's Exact Test for Count Data
## 
## data:  data2_wide$s_sex and data2_wide$s_awareness
## p-value = 0.004472
## alternative hypothesis: true odds ratio is not equal to 1
## 95 percent confidence interval:
##  0.6287428 0.9204853
## sample estimates:
## odds ratio 
##  0.7609049
```

```
data2_wide$s_age_1 <- ifelse(data2_wide$s_age < 45, 0, 1)
age_hist <- ggplot(data2_wide, aes(x = s_age, fill = s_awareness, color = s_awareness))
age_hist <- age_hist + geom_histogram(alpha = 0.1, position = "identity") + theme_classic() + theme(
  panel.grid.major = element_blank(),
  panel.grid.minor = element_blank(),
  panel.background = element_blank(),
  axis.title = element_text(face = "bold"),
  text = element_text(face = "bold"),
  axis.text = element_text(face = "bold"),
  legend.title = element_text(face = "bold"))+
  labs(x = "Age", y = "Number of Cases", fill = "Awareness Check", color = "Awareness Check") + 
  scale_fill_brewer(palette = "Dark2") + scale_color_brewer(palette = "Dark2")
age_hist
```

```
## `stat_bin()` using `bins = 30`. Pick better value with `binwidth`.
```

```
## Warning: Removed 1 rows containing non-finite values (`stat_bin()`).
```

```
CrossTable(data2_wide$s_age_1, data2_wide$s_awareness,
           chisq = TRUE, expected = TRUE, sresid = TRUE, format = "SPSS")
```

```
## 
##    Cell Contents
## |-------------------------|
## |                   Count |
## |         Expected Values |
## | Chi-square contribution |
## |             Row Percent |
## |          Column Percent |
## |           Total Percent |
## |            Std Residual |
## |-------------------------|
## 
## Total Observations in Table:  2040 
## 
##                    | data2_wide$s_awareness 
## data2_wide$s_age_1 |     fail  |     pass  | Row Total | 
## -------------------|-----------|-----------|-----------|
##                  0 |      411  |      605  |     1016  | 
##                    |  327.710  |  688.290  |           | 
##                    |   21.169  |   10.079  |           | 
##                    |   40.453% |   59.547% |   49.804% | 
##                    |   62.462% |   43.777% |           | 
##                    |   20.147% |   29.657% |           | 
##                    |    4.601  |   -3.175  |           | 
## -------------------|-----------|-----------|-----------|
##                  1 |      247  |      777  |     1024  | 
##                    |  330.290  |  693.710  |           | 
##                    |   21.004  |   10.000  |           | 
##                    |   24.121% |   75.879% |   50.196% | 
##                    |   37.538% |   56.223% |           | 
##                    |   12.108% |   38.088% |           | 
##                    |   -4.583  |    3.162  |           | 
## -------------------|-----------|-----------|-----------|
##       Column Total |      658  |     1382  |     2040  | 
##                    |   32.255% |   67.745% |           | 
## -------------------|-----------|-----------|-----------|
## 
##  
## Statistics for All Table Factors
## 
## 
## Pearson's Chi-squared test 
## ------------------------------------------------------------
## Chi^2 =  62.25162     d.f. =  1     p =  3.022599e-15 
## 
## Pearson's Chi-squared test with Yates' continuity correction 
## ------------------------------------------------------------
## Chi^2 =  61.50646     d.f. =  1     p =  4.412932e-15 
## 
##  
##        Minimum expected frequency: 327.7098
```

```
fisher.test(data2_wide$s_age_1, data2_wide$s_awareness, workspace = 2e8)
```

```
## 
##  Fisher's Exact Test for Count Data
## 
## data:  data2_wide$s_age_1 and data2_wide$s_awareness
## p-value = 2.97e-15
## alternative hypothesis: true odds ratio is not equal to 1
## 95 percent confidence interval:
##  1.759109 2.597653
## sample estimates:
## odds ratio 
##   2.136236
```

```
school_bar <- ggplot(data2_wide,aes(x = s_school, fill = s_awareness))
school_bar <- school_bar +  geom_bar() + theme_classic() + theme(
  panel.grid.major = element_blank(),
  panel.grid.minor = element_blank(),
  panel.background = element_blank(),
  axis.title = element_text(face = "bold"),
  text = element_text(face = "bold"),
  axis.text = element_text(face = "bold"),
  legend.title = element_text(face = "bold"))+
  labs(x = "Schooltype", y = "Number of Cases", fill = "Awareness Check") +
  scale_fill_brewer(palette = "Blues")
school_bar
```

```
CrossTable(data2_wide$s_school, data2_wide$s_awareness,
           chisq = TRUE, expected = TRUE, sresid = TRUE, format = "SPSS")
```

```
## 
##    Cell Contents
## |-------------------------|
## |                   Count |
## |         Expected Values |
## | Chi-square contribution |
## |             Row Percent |
## |          Column Percent |
## |           Total Percent |
## |            Std Residual |
## |-------------------------|
## 
## Total Observations in Table:  2041 
## 
##                     | data2_wide$s_awareness 
## data2_wide$s_school |     fail  |     pass  | Row Total | 
## --------------------|-----------|-----------|-----------|
##               Haupt |      270  |      415  |      685  | 
##                     |  220.838  |  464.162  |           | 
##                     |   10.944  |    5.207  |           | 
##                     |   39.416% |   60.584% |   33.562% | 
##                     |   41.033% |   30.007% |           | 
##                     |   13.229% |   20.333% |           | 
##                     |    3.308  |   -2.282  |           | 
## --------------------|-----------|-----------|-----------|
##                Real |      220  |      461  |      681  | 
##                     |  219.548  |  461.452  |           | 
##                     |    0.001  |    0.000  |           | 
##                     |   32.305% |   67.695% |   33.366% | 
##                     |   33.435% |   33.333% |           | 
##                     |   10.779% |   22.587% |           | 
##                     |    0.030  |   -0.021  |           | 
## --------------------|-----------|-----------|-----------|
##                 Abi |      168  |      507  |      675  | 
##                     |  217.614  |  457.386  |           | 
##                     |   11.312  |    5.382  |           | 
##                     |   24.889% |   75.111% |   33.072% | 
##                     |   25.532% |   36.659% |           | 
##                     |    8.231% |   24.841% |           | 
##                     |   -3.363  |    2.320  |           | 
## --------------------|-----------|-----------|-----------|
##        Column Total |      658  |     1383  |     2041  | 
##                     |   32.239% |   67.761% |           | 
## --------------------|-----------|-----------|-----------|
## 
##  
## Statistics for All Table Factors
## 
## 
## Pearson's Chi-squared test 
## ------------------------------------------------------------
## Chi^2 =  32.84601     d.f. =  2     p =  7.371908e-08 
## 
## 
##  
##        Minimum expected frequency: 217.6139
```

```
fisher.test(data2_wide$s_school, data2_wide$s_awareness, workspace = 2e8)
```

```
## 
##  Fisher's Exact Test for Count Data
## 
## data:  data2_wide$s_school and data2_wide$s_awareness
## p-value = 6.61e-08
## alternative hypothesis: two.sided
```

## Accessibility

```
describe(data2_wide$accessibility_1)
```

```
##    vars    n mean   sd median trimmed  mad min max range  skew kurtosis   se
## X1    1 2029 5.64 1.79      6    5.77 1.48   1   8     7 -0.45    -0.52 0.04
```

```
describe(data2_wide$accessibility_2)
```

```
##    vars    n mean   sd median trimmed  mad min max range skew kurtosis   se
## X1    1 2016 5.43 1.86      6    5.54 1.48   1   8     7 -0.4    -0.55 0.04
```

```
dep.access.test <- wilcox.test(data2_wide$accessibility_1,
                               data2_wide$accessibility_2,
                               paired = TRUE,
                               correct = TRUE)
dep.access.test
```

```
## 
##  Wilcoxon signed rank test with continuity correction
## 
## data:  data2_wide$accessibility_1 and data2_wide$accessibility_2
## V = 486685, p-value = 1.394e-09
## alternative hypothesis: true location shift is not equal to 0
```

```
data2_wide$accessibility <- rowMeans(data2_wide[,c("accessibility_1",
                                                   "accessibility_2")])
describe(data2_wide$accessibility)
```

```
##    vars    n mean   sd median trimmed  mad min max range  skew kurtosis   se
## X1    1 2005 5.54 1.64    5.5    5.61 1.48   1   8     7 -0.37    -0.43 0.04
```

```
access.hist <- ggplot(data2_wide, aes(accessibility)) + 
  geom_histogram(colour = "black", fill = "white") + labs(x = "Mean Accessibility",
                                                          y = "Frequency")
access.hist
```

```
## `stat_bin()` using `bins = 30`. Pick better value with `binwidth`.
```

```
## Warning: Removed 36 rows containing non-finite values (`stat_bin()`).
```

## Understanding

```
describe(data2_wide$understanding_1)
```

```
##    vars    n mean   sd median trimmed  mad min max range  skew kurtosis   se
## X1    1 2021 5.78 1.66      6    5.89 1.48   1   8     7 -0.47    -0.36 0.04
```

```
describe(data2_wide$understanding_2)
```

```
##    vars    n mean   sd median trimmed  mad min max range  skew kurtosis   se
## X1    1 2017 5.41 1.73      6    5.49 1.48   1   8     7 -0.42    -0.34 0.04
```

```
dep.understand.test <- wilcox.test(data2_wide$understanding_1,
                               data2_wide$understanding_2,
                               paired = TRUE,
                               correct = TRUE)
dep.understand.test
```

```
## 
##  Wilcoxon signed rank test with continuity correction
## 
## data:  data2_wide$understanding_1 and data2_wide$understanding_2
## V = 571930, p-value < 2.2e-16
## alternative hypothesis: true location shift is not equal to 0
```

```
data2_wide$understanding <- rowMeans(data2_wide[,c("understanding_1",
                                                   "understanding_2")])
describe(data2_wide$understanding)
```

```
##    vars    n mean  sd median trimmed  mad min max range skew kurtosis   se
## X1    1 1998  5.6 1.5    5.5    5.66 1.48   1   8     7 -0.4    -0.28 0.03
```

```
understand.hist <- ggplot(data2_wide, aes(understanding)) + 
  geom_histogram(colour = "black", fill = "white") + labs(x = "Mean Understanding",
                                                          y = "Frequency")
understand.hist
```

```
## `stat_bin()` using `bins = 30`. Pick better value with `binwidth`.
```

```
## Warning: Removed 43 rows containing non-finite values (`stat_bin()`).
```

## Empowerment

```
describe(data2_wide$empowerment_1)
```

```
##    vars    n mean  sd median trimmed  mad min max range  skew kurtosis   se
## X1    1 2018 4.85 1.8      5    4.89 1.48   1   8     7 -0.19    -0.48 0.04
```

```
describe(data2_wide$empowerment_2)
```

```
##    vars    n mean   sd median trimmed  mad min max range  skew kurtosis   se
## X1    1 2021 4.71 1.82      5    4.75 1.48   1   8     7 -0.18    -0.48 0.04
```

```
dep.emp.test <- wilcox.test(data2_wide$empowerment_1,
                            data2_wide$empowerment_2,
                            paired = TRUE,
                            correct = TRUE)
dep.emp.test
```

```
## 
##  Wilcoxon signed rank test with continuity correction
## 
## data:  data2_wide$empowerment_1 and data2_wide$empowerment_2
## V = 475021, p-value = 0.0001433
## alternative hypothesis: true location shift is not equal to 0
```

```
data2_wide$empowerment <- rowMeans(data2_wide[,c("empowerment_1",
                                                 "empowerment_2")])
describe(data2_wide$empowerment)
```

```
##    vars    n mean   sd median trimmed  mad min max range  skew kurtosis   se
## X1    1 2000 4.77 1.63      5    4.81 1.48   1   8     7 -0.18    -0.35 0.04
```

```
empower.hist <- ggplot(data2_wide, aes(empowerment)) + 
  geom_histogram(colour = "black", fill = "white") + labs(x = "Mean Empowerment",
                                                          y = "Frequency")
empower.hist
```

```
## `stat_bin()` using `bins = 30`. Pick better value with `binwidth`.
```

```
## Warning: Removed 41 rows containing non-finite values (`stat_bin()`).
```

## Credibility

```
data2_wide$credibility <- rowMeans(data2_wide[,c("credibility_1",
                                                 "credibility_2")])

describe(data2_wide$credibility_1)
```

```
##    vars    n mean   sd median trimmed  mad min max range  skew kurtosis   se
## X1    1 2020 5.91 1.52      6    5.97 1.48   1   8     7 -0.35    -0.48 0.03
```

```
describe(data2_wide$credibility_2)
```

```
##    vars    n mean   sd median trimmed  mad min max range  skew kurtosis   se
## X1    1 2018 5.77 1.58      6    5.85 1.48   1   8     7 -0.43    -0.17 0.04
```

```
dep.credible.test <- wilcox.test(data2_wide$credibility_1,
                               data2_wide$credibility_2,
                               paired = TRUE,
                               correct = TRUE)
dep.credible.test
```

```
## 
##  Wilcoxon signed rank test with continuity correction
## 
## data:  data2_wide$credibility_1 and data2_wide$credibility_2
## V = 391005, p-value = 0.0001058
## alternative hypothesis: true location shift is not equal to 0
```

```
describe(data2_wide$credibility)
```

```
##    vars    n mean   sd median trimmed  mad min max range  skew kurtosis   se
## X1    1 1997 5.84 1.37      6    5.87 1.48   1   8     7 -0.25    -0.47 0.03
```

```
credible.hist <- ggplot(data2_wide, aes(credibility)) + 
  geom_histogram(colour = "black", fill = "white") + labs(x = "Mean Credibility",
                                                          y = "Frequency")
credible.hist
```

```
## `stat_bin()` using `bins = 30`. Pick better value with `binwidth`.
```

```
## Warning: Removed 44 rows containing non-finite values (`stat_bin()`).
```

## Relevance

```
data2_wide$relevance <- rowMeans(data2_wide[,c("relevance_1","relevance_2")])
describe(data2_wide$relevance_1)
```

```
##    vars    n mean   sd median trimmed  mad min max range  skew kurtosis   se
## X1    1 2024 6.33 1.58      7    6.51 1.48   1   8     7 -0.78     0.06 0.04
```

```
describe(data2_wide$relevance_2)
```

```
##    vars    n mean   sd median trimmed  mad min max range skew kurtosis   se
## X1    1 2019 6.19 1.67      6    6.37 1.48   1   8     7 -0.8     0.15 0.04
```

```
dep.relevance.test <- wilcox.test(data2_wide$relevance_1,
                                 data2_wide$relevance_2,
                                 paired = TRUE,
                                 correct = TRUE)
dep.relevance.test
```

```
## 
##  Wilcoxon signed rank test with continuity correction
## 
## data:  data2_wide$relevance_1 and data2_wide$relevance_2
## V = 383964, p-value = 5.793e-05
## alternative hypothesis: true location shift is not equal to 0
```

```
describe(data2_wide$relevance)
```

```
##    vars    n mean   sd median trimmed  mad min max range  skew kurtosis   se
## X1    1 2003 6.26 1.42    6.5    6.39 1.48   1   8     7 -0.62    -0.14 0.03
```

```
relevance.hist <- ggplot(data2_wide, aes(relevance)) + 
  geom_histogram(colour = "black", fill = "white") + labs(x = "Mean Relevance",
                                                          y = "Frequency")
relevance.hist
```

```
## `stat_bin()` using `bins = 30`. Pick better value with `binwidth`.
```

```
## Warning: Removed 38 rows containing non-finite values (`stat_bin()`).
```

## Relationship Knowledge-Item

```
describe(data2_wide$s_relationship)
```

```
##    vars    n mean   sd median trimmed  mad min max range skew kurtosis   se
## X1    1 1979 0.23 3.22      0    0.02 2.97  -8   8    16 0.45    -0.37 0.07
```

```
table(data2_wide$s_relationship)
```

```
## 
##  -8  -7  -6  -5  -4  -3  -2  -1   0   1   2   3   4   5   6   7   8 
##   4   1  20  20 247 110 251 163 425 112 192  67 141  30 135  17  44
```

```
relationship.hist <- ggplot(data2_wide, aes(s_relationship)) + 
  geom_histogram(colour = "black", fill = "white") + labs(
    x = "Mean Relationship Knowledge Score", y = "Frequency") +
  scale_x_continuous(breaks = seq(-8,8,1))
relationship.hist
```

```
## `stat_bin()` using `bins = 30`. Pick better value with `binwidth`.
```

```
## Warning: Removed 62 rows containing non-finite values (`stat_bin()`).
```

## Extent of Evaluation Knowledge-Item

```
describe(data2_wide$s_extent)
```

```
##    vars    n mean   sd median trimmed  mad min max range skew kurtosis   se
## X1    1 1999 0.52 2.28      0    0.41 2.97  -6   6    12 0.35    -0.28 0.05
```

```
extent.hist <- ggplot(data2_wide, aes(s_extent)) + 
  geom_histogram(colour = "black", fill = "white") + labs(
    x = "Mean Extent Knowledge Score",
    y = "Frequency") +
  scale_x_continuous(breaks = seq(-6,6,1))
extent.hist
```

```
## `stat_bin()` using `bins = 30`. Pick better value with `binwidth`.
```

```
## Warning: Removed 42 rows containing non-finite values (`stat_bin()`).
```

## Differentiation Knowledge-Item

```
data2_wide$s_diff <- coalesce(data2_wide$s_diff_1,data2_wide$s_diff_2)
describe(data2_wide$s_diff_1)
```

```
##    vars   n mean   sd median trimmed  mad min max range skew kurtosis   se
## X1    1 997 0.07 1.83      0    0.05 1.48  -6   6    12 0.14     0.68 0.06
```

```
describe(data2_wide$s_diff_2)
```

```
##    vars   n mean   sd median trimmed  mad min max range skew kurtosis   se
## X1    1 981 0.52 2.07      0    0.46 2.97  -6   6    12 0.15     0.23 0.07
```

```
dep.diff.test <- wilcox.test(data2_wide$s_diff_1,
                                data2_wide$s_diff_2,
                                    paired = FALSE,
                                    correct = TRUE)
dep.diff.test
```

```
## 
##  Wilcoxon rank sum test with continuity correction
## 
## data:  data2_wide$s_diff_1 and data2_wide$s_diff_2
## W = 428088, p-value = 8.067e-07
## alternative hypothesis: true location shift is not equal to 0
```

```
describe(data2_wide$s_diff)
```

```
##    vars    n mean   sd median trimmed  mad min max range skew kurtosis   se
## X1    1 1978 0.29 1.97      0    0.23 1.48  -6   6    12 0.19     0.46 0.04
```

```
diff.hist <- ggplot(data2_wide, aes(s_diff)) + 
  geom_histogram(colour = "black", fill = "white") + labs(
    x = "Mean Differentiation Knowledge Score",
    y = "Frequency") +
  scale_x_continuous(breaks = seq(-6,6,1))
diff.hist
```

```
## `stat_bin()` using `bins = 30`. Pick better value with `binwidth`.
```

```
## Warning: Removed 63 rows containing non-finite values (`stat_bin()`).
```

## Funding Knowledge-Item

```
data2_wide$s_funding <- rowSums(data2_wide[,c("s_funding_1",
                                               "s_funding_2")])
describe(data2_wide$s_funding_1)
```

```
##    vars    n mean   sd median trimmed  mad min max range skew kurtosis   se
## X1    1 1994 0.87 3.05      0    0.84 2.97  -6   6    12 0.26    -0.74 0.07
```

```
describe(data2_wide$s_funding_2)
```

```
##    vars    n mean   sd median trimmed  mad min max range  skew kurtosis   se
## X1    1 2010 1.67 3.22      1    1.82 4.45  -6   6    12 -0.07    -1.03 0.07
```

```
dep.funding.test <- wilcox.test(data2_wide$s_funding_1,
                                data2_wide$s_funding_2,
                                    paired = TRUE,
                                    correct = TRUE)
dep.funding.test
```

```
## 
##  Wilcoxon signed rank test with continuity correction
## 
## data:  data2_wide$s_funding_1 and data2_wide$s_funding_2
## V = 321887, p-value < 2.2e-16
## alternative hypothesis: true location shift is not equal to 0
```

```
describe(data2_wide$s_funding)
```

```
##    vars    n mean   sd median trimmed  mad min max range skew kurtosis   se
## X1    1 1965 2.55 5.42      2    2.53 5.93 -12  12    24 0.07    -0.74 0.12
```

```
funding.hist <- ggplot(data2_wide, aes(s_funding)) + 
  geom_histogram(colour = "black", fill = "white") + labs(
    x = "Mean Funding Knowledge Score",
    y = "Frequency") +
  scale_x_continuous(breaks = seq(-12,12,1))
funding.hist
```

```
## `stat_bin()` using `bins = 30`. Pick better value with `binwidth`.
```

```
## Warning: Removed 76 rows containing non-finite values (`stat_bin()`).
```

## COI Knowledge-Item

```
data2_wide$s_coi <- rowSums(data2_wide[,c("s_coi_1",
                                               "s_coi_2")])
describe(data2_wide$s_coi_1)
```

```
##    vars    n mean   sd median trimmed  mad min max range skew kurtosis   se
## X1    1 1955 0.72 3.33      0    0.66 2.97  -7   7    14 0.19     -0.5 0.08
```

```
describe(data2_wide$s_coi_2)
```

```
##    vars    n mean   sd median trimmed  mad min max range  skew kurtosis   se
## X1    1 1993  1.3 3.56      1    1.36 2.97  -7   7    14 -0.01    -0.75 0.08
```

```
dep.coi.test <- wilcox.test(data2_wide$s_coi_1,
                                data2_wide$s_coi_2,
                                paired = TRUE,
                                correct = TRUE)
dep.coi.test
```

```
## 
##  Wilcoxon signed rank test with continuity correction
## 
## data:  data2_wide$s_coi_1 and data2_wide$s_coi_2
## V = 401472, p-value = 1.122e-12
## alternative hypothesis: true location shift is not equal to 0
```

```
describe(data2_wide$s_coi)
```

```
##    vars    n mean   sd median trimmed  mad min max range  skew kurtosis   se
## X1    1 1914 2.02 5.88      1    2.13 5.93 -13  14    27 -0.05    -0.59 0.13
```

```
coi.hist <- ggplot(data2_wide, aes(s_coi)) + 
  geom_histogram(colour = "black", fill = "white") + labs(
    x = "Mean COI Knowledge Score",
    y = "Frequency") +
  scale_x_continuous(breaks = seq(-14,14,1))
coi.hist
```

```
## `stat_bin()` using `bins = 30`. Pick better value with `binwidth`.
```

```
## Warning: Removed 127 rows containing non-finite values (`stat_bin()`).
```

## Causality Knowledge-Item

```
data2_wide$s_causality <- rowSums(data2_wide[,c("s_causality_1",
                                                 "s_causality_2")])
describe(data2_wide$s_causality_1)
```

```
##    vars    n  mean   sd median trimmed  mad min max range skew kurtosis   se
## X1    1 1989 -0.43 2.45      0   -0.52 2.97  -6   6    12  0.2    -0.56 0.06
```

```
describe(data2_wide$s_causality_2)
```

```
##    vars    n  mean   sd median trimmed  mad min max range skew kurtosis   se
## X1    1 1995 -0.21 2.45      0   -0.29 2.97  -6   6    12 0.21    -0.31 0.05
```

```
dep.causality.test <- wilcox.test(data2_wide$s_causality_1,
                            data2_wide$s_causality_2,
                            paired = TRUE,
                            correct = TRUE)
dep.causality.test
```

```
## 
##  Wilcoxon signed rank test with continuity correction
## 
## data:  data2_wide$s_causality_1 and data2_wide$s_causality_2
## V = 486523, p-value = 0.0008355
## alternative hypothesis: true location shift is not equal to 0
```

```
describe(data2_wide$s_causality)
```

```
##    vars    n  mean  sd median trimmed  mad min max range skew kurtosis   se
## X1    1 1949 -0.64 3.9      0   -0.67 2.97 -12  12    24  0.1    -0.15 0.09
```

```
causality.hist <- ggplot(data2_wide, aes(s_causality)) + 
  geom_histogram(colour = "black", fill = "white") + labs(
    x = "Mean Causality Knowledge Score",
    y = "Frequency") +
  scale_x_continuous(breaks = seq(-12,12,1))
causality.hist
```

```
## `stat_bin()` using `bins = 30`. Pick better value with `binwidth`.
```

```
## Warning: Removed 92 rows containing non-finite values (`stat_bin()`).
```

## CAMA Knowledge-Items

```
describe(data2_wide$s_CAMA_1)
```

```
##    vars   n mean   sd median trimmed  mad min max range skew kurtosis   se
## X1    1 984  0.7 2.66      0    0.68 2.97  -7   8    15 0.16    -0.02 0.08
```

```
describe(data2_wide$s_CAMA_2)
```

```
##    vars    n mean   sd median trimmed  mad min max range skew kurtosis   se
## X1    1 1015 -0.4 1.62      0   -0.44 1.48  -4   4     8 0.18     0.05 0.05
```

```
describe(data2_wide$s_CAMA_3)
```

```
##    vars    n  mean   sd median trimmed  mad min max range skew kurtosis   se
## X1    1 1026 -0.14 0.75      0   -0.17 1.48  -1   1     2 0.23    -1.21 0.02
```

```
describe(data2_wide$s_CAMA)
```

```
##    vars   n mean   sd median trimmed  mad min max range skew kurtosis   se
## X1    1 975 0.17 3.73      0    0.17 2.97 -11  13    24 0.09     0.13 0.12
```

```
CAMA1.hist <- ggplot(data2_wide, aes(s_CAMA_1)) + 
  geom_histogram(colour = "black", fill = "white") + labs(
    x = "Mean CAMA Knowledge Score",
    y = "Frequency") +
  scale_x_continuous(breaks = seq(-8,8,1))
CAMA1.hist
```

```
## `stat_bin()` using `bins = 30`. Pick better value with `binwidth`.
```

```
## Warning: Removed 1057 rows containing non-finite values (`stat_bin()`).
```

```
CAMA2.hist <- ggplot(data2_wide, aes(s_CAMA_2)) + 
  geom_histogram(colour = "black", fill = "white") + labs(
    x = "Mean CAMA Knowledge Score",
    y = "Frequency") +
  scale_x_continuous(breaks = seq(-4,4,1))
CAMA2.hist
```

```
## `stat_bin()` using `bins = 30`. Pick better value with `binwidth`.
```

```
## Warning: Removed 1026 rows containing non-finite values (`stat_bin()`).
```

```
CAMA3.hist <- ggplot(data2_wide, aes(s_CAMA_3)) + 
  geom_histogram(colour = "black", fill = "white") + labs(
    x = "Mean CAMA Knowledge Score",
    y = "Frequency") +
  scale_x_continuous(breaks = seq(-1,1,1))
CAMA3.hist
```

```
## `stat_bin()` using `bins = 30`. Pick better value with `binwidth`.
```

```
## Warning: Removed 1015 rows containing non-finite values (`stat_bin()`).
```

```
CAMA_oa.hist <- ggplot(data2_wide, aes(s_CAMA)) + 
  geom_histogram(colour = "black", fill = "white") + labs(
    x = "Mean CAMA Knowledge Score",
    y = "Frequency") +
  scale_x_continuous(breaks = seq(-13,13,1))
CAMA_oa.hist
```

```
## `stat_bin()` using `bins = 30`. Pick better value with `binwidth`.
```

```
## Warning: Removed 1066 rows containing non-finite values (`stat_bin()`).
```

# Hypotheses Testing (Awareness Check Pass)

```
data2_wide_pass <- subset(data2_wide, s_awareness == "pass")
length(unique(data2_wide_pass$id))
```

```
## [1] 1383
```

```
data2_long_pass <- subset(data2_long, s_awareness == "pass")
length(unique(data2_long_pass$id))
```

```
## [1] 1383
```

## H1

### H1a

```
H1a_pass <- subset(data2_wide_pass, condition == 2|condition == 4|condition == 6)
View(H1a_pass)

describeBy(H1a_pass$s_relationship,H1a_pass$disclaimer)
```

```
## 
##  Descriptive statistics by group 
## group: no disclaimer
##    vars   n mean   sd median trimmed  mad min max range skew kurtosis  se
## X1    1 240 0.51 3.02      0    0.45 2.97  -7   8    15 0.19    -0.46 0.2
## ------------------------------------------------------------ 
## group: disclaimer
##    vars   n mean   sd median trimmed  mad min max range skew kurtosis   se
## X1    1 450 0.91 3.64      0    0.74 2.97  -6   8    14 0.38    -0.94 0.17
```

```
wilcox.test(s_relationship~disclaimer, data = H1a_pass, exact = FALSE,
            conf.int = TRUE)
```

```
## 
##  Wilcoxon rank sum test with continuity correction
## 
## data:  s_relationship by disclaimer
## W = 52152, p-value = 0.4557
## alternative hypothesis: true location shift is not equal to 0
## 95 percent confidence interval:
##  -9.999659e-01  6.563517e-05
## sample estimates:
## difference in location 
##          -2.086247e-05
```

#### H1a post hoc

```
H1a_pass_1 <- subset(data2_wide_pass, condition ==2| condition == 6)
View(H1a_pass_1)
describeBy(H1a_pass_1$s_relationship, H1a_pass_1$disclaimer)
```

```
## 
##  Descriptive statistics by group 
## group: no disclaimer
##    vars   n mean   sd median trimmed  mad min max range skew kurtosis  se
## X1    1 240 0.51 3.02      0    0.45 2.97  -7   8    15 0.19    -0.46 0.2
## ------------------------------------------------------------ 
## group: disclaimer
##    vars   n mean   sd median trimmed  mad min max range skew kurtosis   se
## X1    1 246 0.61 3.66      0    0.38 2.97  -6   8    14 0.45    -0.86 0.23
```

```
wilcox.test(s_relationship~disclaimer, data = H1a_pass_1, exaxct = FALSE,
            conf.int = TRUE)
```

```
## 
##  Wilcoxon rank sum test with continuity correction
## 
## data:  s_relationship by disclaimer
## W = 30093, p-value = 0.71
## alternative hypothesis: true location shift is not equal to 0
## 95 percent confidence interval:
##  -6.923148e-05  9.999839e-01
## sample estimates:
## difference in location 
##           5.003085e-05
```

```
H1a_pass_2 <- subset(data2_wide_pass, condition ==4| condition == 6)
View(H1a_pass_2)
describeBy(H1a_pass_2$s_relationship, H1a_pass_2$disclaimer)
```

```
## 
##  Descriptive statistics by group 
## group: no disclaimer
##    vars   n mean   sd median trimmed  mad min max range skew kurtosis  se
## X1    1 240 0.51 3.02      0    0.45 2.97  -7   8    15 0.19    -0.46 0.2
## ------------------------------------------------------------ 
## group: disclaimer
##    vars   n mean   sd median trimmed  mad min max range skew kurtosis   se
## X1    1 204 1.27 3.59      0    1.16 2.97  -5   8    13  0.3    -1.03 0.25
```

```
wilcox.test(s_relationship~disclaimer, data = H1a_pass_2, exaxct = FALSE,
            conf.int = TRUE)
```

```
## 
##  Wilcoxon rank sum test with continuity correction
## 
## data:  s_relationship by disclaimer
## W = 22059, p-value = 0.07029
## alternative hypothesis: true location shift is not equal to 0
## 95 percent confidence interval:
##  -1.000003e+00  7.173675e-05
## sample estimates:
## difference in location 
##          -1.098109e-05
```

### H1b

```
H1b_pass <- subset(data2_wide_pass, condition == 1|condition == 2|condition == 3|
                condition == 4)
View(H1b_pass)

describeBy(H1b_pass$s_relationship,H1b_pass$disclaimer)
```

```
## 
##  Descriptive statistics by group 
## group: no disclaimer
##    vars   n mean   sd median trimmed  mad min max range skew kurtosis   se
## X1    1 424 0.45 3.21      0    0.32 2.97  -6   8    14 0.25    -0.63 0.16
## ------------------------------------------------------------ 
## group: disclaimer
##    vars   n mean   sd median trimmed  mad min max range skew kurtosis   se
## X1    1 450 0.91 3.64      0    0.74 2.97  -6   8    14 0.38    -0.94 0.17
```

```
wilcox.test(s_relationship~disclaimer, data = H1b_pass, exact = FALSE,
            conf.int = TRUE)
```

```
## 
##  Wilcoxon rank sum test with continuity correction
## 
## data:  s_relationship by disclaimer
## W = 90501, p-value = 0.1862
## alternative hypothesis: true location shift is not equal to 0
## 95 percent confidence interval:
##  -9.999266e-01  2.889107e-05
## sample estimates:
## difference in location 
##          -3.904304e-05
```

#### H1b post hoc

```
H1b_pass_1 <- subset(data2_wide_pass, condition == 1|condition == 2)
View(H1b_pass_1)
describeBy(H1b_pass_1$s_relationship,H1b_pass_1$disclaimer)
```

```
## 
##  Descriptive statistics by group 
## group: no disclaimer
##    vars   n mean   sd median trimmed  mad min max range skew kurtosis   se
## X1    1 213 0.62 3.21      0    0.56 2.97  -6   8    14 0.09    -0.68 0.22
## ------------------------------------------------------------ 
## group: disclaimer
##    vars   n mean   sd median trimmed  mad min max range skew kurtosis   se
## X1    1 246 0.61 3.66      0    0.38 2.97  -6   8    14 0.45    -0.86 0.23
```

```
wilcox.test(s_relationship~disclaimer, data = H1b_pass_1, exact = FALSE,
            conf.int = TRUE)
```

```
## 
##  Wilcoxon rank sum test with continuity correction
## 
## data:  s_relationship by disclaimer
## W = 27041, p-value = 0.5505
## alternative hypothesis: true location shift is not equal to 0
## 95 percent confidence interval:
##  -4.730705e-05  9.999690e-01
## sample estimates:
## difference in location 
##           5.618701e-05
```

```
H1b_pass_2 <- subset(data2_wide_pass, condition == 3|condition == 4)
View(H1b_pass_2)
describeBy(H1b_pass_2$s_relationship,H1b_pass_2$disclaimer)
```

```
## 
##  Descriptive statistics by group 
## group: no disclaimer
##    vars   n mean   sd median trimmed  mad min max range skew kurtosis   se
## X1    1 211 0.27 3.21      0    0.09 2.97  -6   8    14 0.41    -0.54 0.22
## ------------------------------------------------------------ 
## group: disclaimer
##    vars   n mean   sd median trimmed  mad min max range skew kurtosis   se
## X1    1 204 1.27 3.59      0    1.16 2.97  -5   8    13  0.3    -1.03 0.25
```

```
wilcox.test(s_relationship~disclaimer, data = H1b_pass_2, exact = FALSE,
            conf.int = TRUE)
```

```
## 
##  Wilcoxon rank sum test with continuity correction
## 
## data:  s_relationship by disclaimer
## W = 18358, p-value = 0.009108
## alternative hypothesis: true location shift is not equal to 0
## 95 percent confidence interval:
##  -1.999996e+00 -2.505375e-05
## sample estimates:
## difference in location 
##             -0.9999673
```

```
H1b_pass_3 <- subset(data2_wide_pass, condition == 1|condition == 4)
View(H1b_pass_3)
describeBy(H1b_pass_3$s_relationship,H1b_pass_3$disclaimer)
```

```
## 
##  Descriptive statistics by group 
## group: no disclaimer
##    vars   n mean   sd median trimmed  mad min max range skew kurtosis   se
## X1    1 213 0.62 3.21      0    0.56 2.97  -6   8    14 0.09    -0.68 0.22
## ------------------------------------------------------------ 
## group: disclaimer
##    vars   n mean   sd median trimmed  mad min max range skew kurtosis   se
## X1    1 204 1.27 3.59      0    1.16 2.97  -5   8    13  0.3    -1.03 0.25
```

```
wilcox.test(s_relationship~disclaimer, data = H1b_pass_3, exact = FALSE,
            conf.int = TRUE)
```

```
## 
##  Wilcoxon rank sum test with continuity correction
## 
## data:  s_relationship by disclaimer
## W = 19958, p-value = 0.1479
## alternative hypothesis: true location shift is not equal to 0
## 95 percent confidence interval:
##  -1.000021e+00  3.034113e-05
## sample estimates:
## difference in location 
##          -6.483228e-05
```

```
H1b_pass_4 <- subset(data2_wide_pass, condition == 2|condition == 3)
View(H1b_pass_4)
describeBy(H1b_pass_4$s_relationship,H1b_pass_4$disclaimer)
```

```
## 
##  Descriptive statistics by group 
## group: no disclaimer
##    vars   n mean   sd median trimmed  mad min max range skew kurtosis   se
## X1    1 211 0.27 3.21      0    0.09 2.97  -6   8    14 0.41    -0.54 0.22
## ------------------------------------------------------------ 
## group: disclaimer
##    vars   n mean   sd median trimmed  mad min max range skew kurtosis   se
## X1    1 246 0.61 3.66      0    0.38 2.97  -6   8    14 0.45    -0.86 0.23
```

```
wilcox.test(s_relationship~disclaimer, data = H1b_pass_4, exact = FALSE,
            conf.int = TRUE)
```

```
## 
##  Wilcoxon rank sum test with continuity correction
## 
## data:  s_relationship by disclaimer
## W = 25145, p-value = 0.5635
## alternative hypothesis: true location shift is not equal to 0
## 95 percent confidence interval:
##  -9.999563e-01  4.130311e-05
## sample estimates:
## difference in location 
##           -4.90988e-05
```

### H1 Logistic Regression

```
sum(is.na(data2_wide_pass$disclaimer))
```

```
## [1] 0
```

```
sum(is.na(data2_wide_pass$s_awareness))
```

```
## [1] 0
```

```
sum(is.na(data2_wide_pass$text_order))
```

```
## [1] 0
```

```
sum(is.na(data2_wide_pass$s_age))
```

```
## [1] 1
```

```
data2_wide_pass <- data2_wide_pass %>% drop_na(s_age)
sum(is.na(data2_wide_pass$s_sex))
```

```
## [1] 0
```

```
sum(is.na(data2_wide_pass$s_school))
```

```
## [1] 0
```

```
sum(is.na(data2_wide_pass$s_interest))
```

```
## [1] 0
```

```
data2_wide_pass$H1_interaction <- interaction(data2_wide_pass$disclaimer,
                                         data2_wide_pass$version)
data2_wide_pass$H1_interaction <- droplevels(data2_wide_pass$H1_interaction)

data2_wide_pass$H1_interaction <- factor(data2_wide_pass$H1_interaction, 
                                             levels = c(
                                               "no disclaimer.old guideline",
                                               "no disclaimer.new guideline",
                                               "disclaimer.new guideline"))
table(data2_wide_pass$H1_interaction)
```

```
## 
## no disclaimer.old guideline no disclaimer.new guideline 
##                         247                         441 
##    disclaimer.new guideline 
##                         694
```

```
data2_wide_pass_reg <- subset(data2_wide_pass, condition != 5)
View(data2_wide_pass_reg)

relationship_null_pass <- clm(as.factor(s_relationship)~1,
                              data = data2_wide_pass_reg, link = "logit")

relationship_model1_pass <- clm(as.factor(s_relationship)~ H1_interaction,
                           data = data2_wide_pass_reg, link = "logit")
anova(relationship_null_pass,relationship_model1_pass)
```

```
## Likelihood ratio tests of cumulative link models:
##  
##                          formula:                                   link:
## relationship_null_pass   as.factor(s_relationship) ~ 1              logit
## relationship_model1_pass as.factor(s_relationship) ~ H1_interaction logit
##                          threshold:
## relationship_null_pass   flexible  
## relationship_model1_pass flexible  
## 
##                          no.par    AIC  logLik LR.stat df Pr(>Chisq)
## relationship_null_pass       15 5463.4 -2716.7                      
## relationship_model1_pass     17 5465.6 -2715.8  1.8388  2     0.3988
```

```
relationship_model2_pass <- clm(as.factor(s_relationship)~ 
                             H1_interaction + summary1,
                             data = data2_wide_pass_reg,
                             link = "logit")
anova(relationship_null_pass,relationship_model2_pass)
```

```
## Likelihood ratio tests of cumulative link models:
##  
##                          formula:                                             
## relationship_null_pass   as.factor(s_relationship) ~ 1                        
## relationship_model2_pass as.factor(s_relationship) ~ H1_interaction + summary1
##                          link: threshold:
## relationship_null_pass   logit flexible  
## relationship_model2_pass logit flexible  
## 
##                          no.par    AIC  logLik LR.stat df Pr(>Chisq)
## relationship_null_pass       15 5463.4 -2716.7                      
## relationship_model2_pass     18 5467.4 -2715.7  2.0697  3     0.5581
```

```
relationship_model3_pass <- clm(as.factor(s_relationship)~ 
                             H1_interaction + summary1 
                             ,  data = data2_wide_pass_reg,
                             link = "logit")
anova(relationship_null_pass,relationship_model3_pass)
```

```
## Likelihood ratio tests of cumulative link models:
##  
##                          formula:                                             
## relationship_null_pass   as.factor(s_relationship) ~ 1                        
## relationship_model3_pass as.factor(s_relationship) ~ H1_interaction + summary1
##                          link: threshold:
## relationship_null_pass   logit flexible  
## relationship_model3_pass logit flexible  
## 
##                          no.par    AIC  logLik LR.stat df Pr(>Chisq)
## relationship_null_pass       15 5463.4 -2716.7                      
## relationship_model3_pass     18 5467.4 -2715.7  2.0697  3     0.5581
```

```
relationship_model4_pass <- clm(as.factor(s_relationship)~ 
                             H1_interaction + summary1 +
                             s_age, data = data2_wide_pass_reg,
                           link = "logit")
anova(relationship_null_pass,relationship_model4_pass)
```

```
## Likelihood ratio tests of cumulative link models:
##  
##                          formula:                                                     
## relationship_null_pass   as.factor(s_relationship) ~ 1                                
## relationship_model4_pass as.factor(s_relationship) ~ H1_interaction + summary1 + s_age
##                          link: threshold:
## relationship_null_pass   logit flexible  
## relationship_model4_pass logit flexible  
## 
##                          no.par    AIC  logLik LR.stat df Pr(>Chisq)    
## relationship_null_pass       15 5463.4 -2716.7                          
## relationship_model4_pass     19 5400.5 -2681.2  70.945  4  1.433e-14 ***
## ---
## Signif. codes:  0 '***' 0.001 '**' 0.01 '*' 0.05 '.' 0.1 ' ' 1
```

```
relationship_model5_pass <- clm(as.factor(s_relationship)~ 
                             H1_interaction + summary1 +
                             s_age + s_sex,
                             data = data2_wide_pass_reg,
                           link = "logit")
anova(relationship_model4_pass,relationship_model5_pass)
```

```
## Likelihood ratio tests of cumulative link models:
##  
##                          formula:                                                             
## relationship_model4_pass as.factor(s_relationship) ~ H1_interaction + summary1 + s_age        
## relationship_model5_pass as.factor(s_relationship) ~ H1_interaction + summary1 + s_age + s_sex
##                          link: threshold:
## relationship_model4_pass logit flexible  
## relationship_model5_pass logit flexible  
## 
##                          no.par    AIC  logLik LR.stat df Pr(>Chisq)  
## relationship_model4_pass     19 5400.5 -2681.2                        
## relationship_model5_pass     20 5399.3 -2679.6  3.1887  1    0.07415 .
## ---
## Signif. codes:  0 '***' 0.001 '**' 0.01 '*' 0.05 '.' 0.1 ' ' 1
```

```
relationship_model6_pass <- clm(as.factor(s_relationship)~ 
                             H1_interaction + summary1 +
                             s_age + s_sex + s_school, 
                           data = data2_wide_pass_reg, link = "logit")
anova(relationship_model4_pass,relationship_model6_pass)
```

```
## Likelihood ratio tests of cumulative link models:
##  
##                          formula:                                                                        
## relationship_model4_pass as.factor(s_relationship) ~ H1_interaction + summary1 + s_age                   
## relationship_model6_pass as.factor(s_relationship) ~ H1_interaction + summary1 + s_age + s_sex + s_school
##                          link: threshold:
## relationship_model4_pass logit flexible  
## relationship_model6_pass logit flexible  
## 
##                          no.par    AIC  logLik LR.stat df Pr(>Chisq)    
## relationship_model4_pass     19 5400.5 -2681.2                          
## relationship_model6_pass     22 5333.5 -2644.7  72.996  3  9.742e-16 ***
## ---
## Signif. codes:  0 '***' 0.001 '**' 0.01 '*' 0.05 '.' 0.1 ' ' 1
```

```
relationship_model7_pass <- clm(as.factor(s_relationship)~ 
                             H1_interaction + summary1 +
                             s_age + s_sex + s_school +
                             as.factor(s_interest), data = data2_wide_pass_reg,
                           link = "logit")
anova(relationship_model6_pass,relationship_model7_pass)
```

```
## Likelihood ratio tests of cumulative link models:
##  
##                          formula:                                                                                                
## relationship_model6_pass as.factor(s_relationship) ~ H1_interaction + summary1 + s_age + s_sex + s_school                        
## relationship_model7_pass as.factor(s_relationship) ~ H1_interaction + summary1 + s_age + s_sex + s_school + as.factor(s_interest)
##                          link: threshold:
## relationship_model6_pass logit flexible  
## relationship_model7_pass logit flexible  
## 
##                          no.par    AIC  logLik LR.stat df Pr(>Chisq)
## relationship_model6_pass     22 5333.5 -2644.7                      
## relationship_model7_pass     26 5338.7 -2643.4  2.7427  4     0.6018
```

```
summary(relationship_model7_pass)
```

```
## formula: 
## as.factor(s_relationship) ~ H1_interaction + summary1 + s_age + s_sex + s_school + as.factor(s_interest)
## data:    data2_wide_pass_reg
## 
##  link  threshold nobs logLik   AIC     niter max.grad cond.H 
##  logit flexible  1114 -2643.37 5338.74 8(1)  1.31e-12 1.2e+06
## 
## Coefficients:
##                                           Estimate Std. Error z value Pr(>|z|)
## H1_interactionno disclaimer.new guideline -0.06035    0.13830  -0.436  0.66260
## H1_interactiondisclaimer.new guideline     0.11472    0.13879   0.827  0.40846
## summary1Faerber                            0.02985    0.10508   0.284  0.77634
## s_age                                     -0.02585    0.00353  -7.322 2.44e-13
## s_sexmale                                 -0.19652    0.10678  -1.840  0.06572
## s_schoolReal                               0.45967    0.13360   3.441  0.00058
## s_schoolAbi                                1.07333    0.13350   8.040 9.00e-16
## as.factor(s_interest)5                     0.02395    0.16480   0.145  0.88447
## as.factor(s_interest)6                     0.13498    0.16600   0.813  0.41612
## as.factor(s_interest)7                    -0.04526    0.17902  -0.253  0.80041
## as.factor(s_interest)8                    -0.12919    0.17824  -0.725  0.46857
##                                              
## H1_interactionno disclaimer.new guideline    
## H1_interactiondisclaimer.new guideline       
## summary1Faerber                              
## s_age                                     ***
## s_sexmale                                 .  
## s_schoolReal                              ***
## s_schoolAbi                               ***
## as.factor(s_interest)5                       
## as.factor(s_interest)6                       
## as.factor(s_interest)7                       
## as.factor(s_interest)8                       
## ---
## Signif. codes:  0 '***' 0.001 '**' 0.01 '*' 0.05 '.' 0.1 ' ' 1
## 
## Threshold coefficients:
##       Estimate Std. Error z value
## -7|-6  -7.9237     1.0343  -7.661
## -6|-5  -5.3487     0.3831 -13.963
## -5|-4  -4.7245     0.3338 -14.156
## -4|-3  -2.8220     0.2746 -10.276
## -3|-2  -2.3623     0.2691  -8.778
## -2|-1  -1.6553     0.2631  -6.291
## -1|0   -1.2762     0.2610  -4.889
## 0|1    -0.4453     0.2586  -1.722
## 1|2    -0.2039     0.2583  -0.789
## 2|3     0.3402     0.2589   1.314
## 3|4     0.5520     0.2596   2.126
## 4|5     1.2115     0.2639   4.592
## 5|6     1.3670     0.2655   5.150
## 6|7     2.6058     0.2911   8.951
## 7|8     2.9495     0.3049   9.674
## (31 Beobachtungen als fehlend gelöscht)
```

```
exp(coef(relationship_model7_pass))
```

```
##                                     -7|-6 
##                              3.620686e-04 
##                                     -6|-5 
##                              4.754510e-03 
##                                     -5|-4 
##                              8.875414e-03 
##                                     -4|-3 
##                              5.948701e-02 
##                                     -3|-2 
##                              9.420078e-02 
##                                     -2|-1 
##                              1.910288e-01 
##                                      -1|0 
##                              2.790939e-01 
##                                       0|1 
##                              6.406466e-01 
##                                       1|2 
##                              8.155315e-01 
##                                       2|3 
##                              1.405292e+00 
##                                       3|4 
##                              1.736693e+00 
##                                       4|5 
##                              3.358526e+00 
##                                       5|6 
##                              3.923682e+00 
##                                       6|7 
##                              1.354198e+01 
##                                       7|8 
##                              1.909619e+01 
## H1_interactionno disclaimer.new guideline 
##                              9.414391e-01 
##    H1_interactiondisclaimer.new guideline 
##                              1.121562e+00 
##                           summary1Faerber 
##                              1.030301e+00 
##                                     s_age 
##                              9.744805e-01 
##                                 s_sexmale 
##                              8.215881e-01 
##                              s_schoolReal 
##                              1.583553e+00 
##                               s_schoolAbi 
##                              2.925090e+00 
##                    as.factor(s_interest)5 
##                              1.024235e+00 
##                    as.factor(s_interest)6 
##                              1.144519e+00 
##                    as.factor(s_interest)7 
##                              9.557482e-01 
##                    as.factor(s_interest)8 
##                              8.788072e-01
```

```
exp(confint(relationship_model7_pass))
```

```
##                                               2.5 %    97.5 %
## H1_interactionno disclaimer.new guideline 0.7179040 1.2347526
## H1_interactiondisclaimer.new guideline    0.8545192 1.4725154
## summary1Faerber                           0.8385192 1.2659907
## s_age                                     0.9677420 0.9812311
## s_sexmale                                 0.6663281 1.0127647
## s_schoolReal                              1.2190899 2.0584310
## s_schoolAbi                               2.2533018 3.8031841
## as.factor(s_interest)5                    0.7414271 1.4148866
## as.factor(s_interest)6                    0.8265856 1.5848085
## as.factor(s_interest)7                    0.6728448 1.3576500
## as.factor(s_interest)8                    0.6195866 1.2463580
```

```
nagelkerke(fit = relationship_model7_pass, null = relationship_null_pass)
```

```
## $Models
##                                                                                                                                                   
## Model: "clm, as.factor(s_relationship) ~ H1_interaction + summary1 + s_age + s_sex + s_school + as.factor(s_interest), data2_wide_pass_reg, logit"
## Null:  "clm, as.factor(s_relationship) ~ 1, data2_wide_pass_reg, logit"                                                                           
## 
## $Pseudo.R.squared.for.model.vs.null
##                              Pseudo.R.squared
## McFadden                            0.0269966
## Cox and Snell (ML)                  0.1233720
## Nagelkerke (Cragg and Uhler)        0.1243190
## 
## $Likelihood.ratio.test
##  Df.diff LogLik.diff  Chisq    p.value
##      -11     -73.342 146.68 7.0852e-26
## 
## $Number.of.observations
##            
## Model: 1114
## Null:  1114
## 
## $Messages
## [1] "Note: For models fit with REML, these statistics are based on refitting with ML"
## 
## $Warnings
## [1] "None"
```

```
H1test_pass = emmeans(relationship_model7_pass, ~ H1_interaction)
pairs(H1test_pass, adjust = "tukey")
```

```
##  contrast                                                  estimate    SE  df
##  no disclaimer.old guideline - no disclaimer.new guideline   0.0603 0.138 Inf
##  no disclaimer.old guideline - disclaimer.new guideline     -0.1147 0.139 Inf
##  no disclaimer.new guideline - disclaimer.new guideline     -0.1751 0.120 Inf
##  z.ratio p.value
##    0.436  0.9004
##   -0.827  0.6865
##   -1.461  0.3100
## 
## Results are averaged over the levels of: summary1, s_sex, s_school, s_interest 
## Note: contrasts are still on the as.factor scale 
## P value adjustment: tukey method for comparing a family of 3 estimates
```

```
cld(H1test_pass, Letters = letters)
```

```
##  H1_interaction              emmean    SE  df asymp.LCL asymp.UCL .group
##  no disclaimer.new guideline  0.328 0.117 Inf    0.0978     0.558  a    
##  no disclaimer.old guideline  0.388 0.137 Inf    0.1190     0.657  a    
##  disclaimer.new guideline     0.503 0.117 Inf    0.2731     0.733  a    
## 
## Results are averaged over the levels of: summary1, s_sex, s_school, s_interest 
## Results are given on the as.factor (not the response) scale. 
## Confidence level used: 0.95 
## Note: contrasts are still on the as.factor scale 
## P value adjustment: tukey method for comparing a family of 3 estimates 
## significance level used: alpha = 0.05 
## NOTE: If two or more means share the same grouping symbol,
##       then we cannot show them to be different.
##       But we also did not show them to be the same.
```

#### Logistic Regression by Group

```
data2_wide_pass_reg1 <- subset(data2_wide_pass_reg, condition == 2 | condition == 6)
View(data2_wide_pass_reg1)

relationship_null_pass1 <- clm(as.factor(s_relationship)~1, data = data2_wide_pass_reg1, link = "logit")

relationship_model8_pass1 <- clm(as.factor(s_relationship)~ 
                             H1_interaction + summary1 +
                             s_age + s_sex + s_school +
                             as.factor(s_interest), data = data2_wide_pass_reg1, link = "logit")

summary(relationship_model8_pass1)
```

```
## formula: 
## as.factor(s_relationship) ~ H1_interaction + summary1 + s_age + s_sex + s_school + as.factor(s_interest)
## data:    data2_wide_pass_reg1
## 
##  link  threshold nobs logLik   AIC     niter max.grad cond.H 
##  logit flexible  486  -1147.25 2344.50 8(1)  1.95e-12 8.8e+05
## 
## Coefficients:
##                                         Estimate Std. Error z value Pr(>|z|)
## H1_interactiondisclaimer.new guideline -0.065661   0.159779  -0.411   0.6811
## summary1Faerber                         0.011016   0.160189   0.069   0.9452
## s_age                                  -0.031365   0.005283  -5.937 2.90e-09
## s_sexmale                              -0.212001   0.164079  -1.292   0.1963
## s_schoolReal                            0.481969   0.204975   2.351   0.0187
## s_schoolAbi                             1.005286   0.199581   5.037 4.73e-07
## as.factor(s_interest)5                  0.089178   0.248484   0.359   0.7197
## as.factor(s_interest)6                  0.438808   0.258044   1.701   0.0890
## as.factor(s_interest)7                 -0.072975   0.274101  -0.266   0.7901
## as.factor(s_interest)8                 -0.158705   0.263502  -0.602   0.5470
##                                           
## H1_interactiondisclaimer.new guideline    
## summary1Faerber                           
## s_age                                  ***
## s_sexmale                                 
## s_schoolReal                           *  
## s_schoolAbi                            ***
## as.factor(s_interest)5                    
## as.factor(s_interest)6                 .  
## as.factor(s_interest)7                    
## as.factor(s_interest)8                    
## ---
## Signif. codes:  0 '***' 0.001 '**' 0.01 '*' 0.05 '.' 0.1 ' ' 1
## 
## Threshold coefficients:
##       Estimate Std. Error z value
## -7|-6  -7.4248     1.0679  -6.953
## -6|-5  -5.8077     0.5834  -9.955
## -5|-4  -5.0036     0.4805 -10.412
## -4|-3  -3.0118     0.3864  -7.795
## -3|-2  -2.6811     0.3794  -7.067
## -2|-1  -1.9338     0.3675  -5.262
## -1|0   -1.4740     0.3627  -4.064
## 0|1    -0.6758     0.3585  -1.885
## 1|2    -0.3784     0.3585  -1.056
## 2|3     0.1258     0.3601   0.349
## 3|4     0.3354     0.3611   0.929
## 4|5     0.9917     0.3672   2.700
## 5|6     1.1274     0.3694   3.052
## 6|7     2.3418     0.4092   5.722
## 7|8     2.7719     0.4377   6.333
## (12 Beobachtungen als fehlend gelöscht)
```

```
exp(coef(relationship_model8_pass1))
```

```
##                                  -7|-6                                  -6|-5 
##                           5.962604e-04                           3.004315e-03 
##                                  -5|-4                                  -4|-3 
##                           6.713648e-03                           4.920516e-02 
##                                  -3|-2                                  -2|-1 
##                           6.849028e-02                           1.445920e-01 
##                                   -1|0                                    0|1 
##                           2.290127e-01                           5.087602e-01 
##                                    1|2                                    2|3 
##                           6.849830e-01                           1.134024e+00 
##                                    3|4                                    4|5 
##                           1.398514e+00                           2.695847e+00 
##                                    5|6                                    6|7 
##                           3.087526e+00                           1.039951e+01 
##                                    7|8 H1_interactiondisclaimer.new guideline 
##                           1.598859e+01                           9.364482e-01 
##                        summary1Faerber                                  s_age 
##                           1.011076e+00                           9.691219e-01 
##                              s_sexmale                           s_schoolReal 
##                           8.089638e-01                           1.619259e+00 
##                            s_schoolAbi                 as.factor(s_interest)5 
##                           2.732688e+00                           1.093276e+00 
##                 as.factor(s_interest)6                 as.factor(s_interest)7 
##                           1.550858e+00                           9.296238e-01 
##                 as.factor(s_interest)8 
##                           8.532480e-01
```

```
exp(confint(relationship_model8_pass1))
```

```
##                                            2.5 %    97.5 %
## H1_interactiondisclaimer.new guideline 0.6845099 1.2808557
## summary1Faerber                        0.7385515 1.3842098
## s_age                                  0.9590856 0.9791665
## s_sexmale                              0.5862197 1.1156221
## s_schoolReal                           1.0843282 2.4227437
## s_schoolAbi                            1.8506928 4.0483615
## as.factor(s_interest)5                 0.6713715 1.7795245
## as.factor(s_interest)6                 0.9349611 2.5728632
## as.factor(s_interest)7                 0.5430250 1.5915346
## as.factor(s_interest)8                 0.5085288 1.4297325
```

```
nagelkerke(fit = relationship_model8_pass1, null = relationship_null_pass1)
```

```
## $Models
##                                                                                                                                                    
## Model: "clm, as.factor(s_relationship) ~ H1_interaction + summary1 + s_age + s_sex + s_school + as.factor(s_interest), data2_wide_pass_reg1, logit"
## Null:  "clm, as.factor(s_relationship) ~ 1, data2_wide_pass_reg1, logit"                                                                           
## 
## $Pseudo.R.squared.for.model.vs.null
##                              Pseudo.R.squared
## McFadden                            0.0322679
## Cox and Snell (ML)                  0.1456570
## Nagelkerke (Cragg and Uhler)        0.1467740
## 
## $Likelihood.ratio.test
##  Df.diff LogLik.diff  Chisq    p.value
##      -10     -38.254 76.508 2.4192e-12
## 
## $Number.of.observations
##           
## Model: 486
## Null:  486
## 
## $Messages
## [1] "Note: For models fit with REML, these statistics are based on refitting with ML"
## 
## $Warnings
## [1] "None"
```

```
H1test_pass1 = emmeans(relationship_model8_pass1, ~ H1_interaction)
pairs(H1test_pass1, adjust = "tukey")
```

```
##  contrast                                               estimate   SE  df
##  no disclaimer.old guideline - disclaimer.new guideline   0.0657 0.16 Inf
##  z.ratio p.value
##    0.411  0.6811
## 
## Results are averaged over the levels of: summary1, s_sex, s_school, s_interest 
## Note: contrasts are still on the as.factor scale
```

```
cld(H1test_pass1, Letters = letters)
```

```
##  H1_interaction              emmean    SE  df asymp.LCL asymp.UCL .group
##  disclaimer.new guideline     0.274 0.150 Inf   -0.0201     0.569  a    
##  no disclaimer.old guideline  0.340 0.149 Inf    0.0473     0.632  a    
## 
## Results are averaged over the levels of: summary1, s_sex, s_school, s_interest 
## Results are given on the as.factor (not the response) scale. 
## Confidence level used: 0.95 
## Note: contrasts are still on the as.factor scale 
## significance level used: alpha = 0.05 
## NOTE: If two or more means share the same grouping symbol,
##       then we cannot show them to be different.
##       But we also did not show them to be the same.
```

```
data2_wide_pass_reg2 <- subset(data2_wide_pass_reg, condition == 4 | condition == 6)
View(data2_wide_pass_reg2)

relationship_null_pass2 <- clm(as.factor(s_relationship)~1, data = data2_wide_pass_reg2, link = "logit")

relationship_model8_pass2 <- clm(as.factor(s_relationship)~ 
                             H1_interaction + summary1 +
                             s_age + s_sex + s_school +
                             as.factor(s_interest), data = data2_wide_pass_reg2, link = "logit")

summary(relationship_model8_pass2)
```

```
## formula: 
## as.factor(s_relationship) ~ H1_interaction + summary1 + s_age + s_sex + s_school + as.factor(s_interest)
## data:    data2_wide_pass_reg2
## 
##  link  threshold nobs logLik   AIC     niter max.grad cond.H 
##  logit flexible  444  -1031.59 2113.19 9(3)  3.60e-09 9.9e+05
## 
## Coefficients:
##                                         Estimate Std. Error z value Pr(>|z|)
## H1_interactiondisclaimer.new guideline  0.352494   0.169991   2.074   0.0381
## summary1Faerber                        -0.188076   0.167999  -1.120   0.2629
## s_age                                  -0.024956   0.005708  -4.372 1.23e-05
## s_sexmale                              -0.433739   0.171931  -2.523   0.0116
## s_schoolReal                            0.502953   0.220156   2.285   0.0223
## s_schoolAbi                             0.941977   0.214734   4.387 1.15e-05
## as.factor(s_interest)5                  0.471621   0.261108   1.806   0.0709
## as.factor(s_interest)6                  0.190710   0.266937   0.714   0.4750
## as.factor(s_interest)7                  0.181755   0.283032   0.642   0.5208
## as.factor(s_interest)8                  0.273741   0.280349   0.976   0.3289
##                                           
## H1_interactiondisclaimer.new guideline *  
## summary1Faerber                           
## s_age                                  ***
## s_sexmale                              *  
## s_schoolReal                           *  
## s_schoolAbi                            ***
## as.factor(s_interest)5                 .  
## as.factor(s_interest)6                    
## as.factor(s_interest)7                    
## as.factor(s_interest)8                    
## ---
## Signif. codes:  0 '***' 0.001 '**' 0.01 '*' 0.05 '.' 0.1 ' ' 1
## 
## Threshold coefficients:
##       Estimate Std. Error z value
## -7|-6 -6.88286    1.08026  -6.372
## -6|-5 -5.48822    0.64543  -8.503
## -5|-4 -4.78372    0.53975  -8.863
## -4|-3 -2.92696    0.43038  -6.801
## -3|-2 -2.47213    0.41996  -5.887
## -2|-1 -1.74864    0.41001  -4.265
## -1|0  -1.25725    0.40655  -3.093
## 0|1   -0.31747    0.40275  -0.788
## 1|2   -0.08492    0.40226  -0.211
## 2|3    0.37662    0.40244   0.936
## 3|4    0.55056    0.40290   1.366
## 4|5    1.25390    0.40822   3.072
## 5|6    1.45013    0.41116   3.527
## 6|7    2.87853    0.46015   6.256
## 7|8    2.94284    0.46417   6.340
## (9 Beobachtungen als fehlend gelöscht)
```

```
exp(coef(relationship_model8_pass2))
```

```
##                                  -7|-6                                  -6|-5 
##                            0.001025204                            0.004135188 
##                                  -5|-4                                  -4|-3 
##                            0.008364854                            0.053559424 
##                                  -3|-2                                  -2|-1 
##                            0.084404961                            0.174011274 
##                                   -1|0                                    0|1 
##                            0.284434790                            0.727991812 
##                                    1|2                                    2|3 
##                            0.918581584                            1.457353505 
##                                    3|4                                    4|5 
##                            1.734226224                            3.503976489 
##                                    5|6                                    6|7 
##                            4.263680510                           17.788192626 
##                                    7|8 H1_interactiondisclaimer.new guideline 
##                           18.969570287                            1.422611563 
##                        summary1Faerber                                  s_age 
##                            0.828551806                            0.975352831 
##                              s_sexmale                           s_schoolReal 
##                            0.648081388                            1.653597700 
##                            s_schoolAbi                 as.factor(s_interest)5 
##                            2.565046537                            1.602589467 
##                 as.factor(s_interest)6                 as.factor(s_interest)7 
##                            1.210108923                            1.199320176 
##                 as.factor(s_interest)8 
##                            1.314873638
```

```
exp(confint(relationship_model8_pass2))
```

```
##                                            2.5 %    97.5 %
## H1_interactiondisclaimer.new guideline 1.0200037 1.9866959
## summary1Faerber                        0.5957644 1.1513567
## s_age                                  0.9644536 0.9862922
## s_sexmale                              0.4622383 0.9072056
## s_schoolReal                           1.0750819 2.5496399
## s_schoolAbi                            1.6867743 3.9159907
## as.factor(s_interest)5                 0.9607293 2.6758901
## as.factor(s_interest)6                 0.7169060 2.0429677
## as.factor(s_interest)7                 0.6887823 2.0907663
## as.factor(s_interest)8                 0.7593988 2.2809793
```

```
nagelkerke(fit = relationship_model8_pass2, null = relationship_null_pass2)
```

```
## $Models
##                                                                                                                                                    
## Model: "clm, as.factor(s_relationship) ~ H1_interaction + summary1 + s_age + s_sex + s_school + as.factor(s_interest), data2_wide_pass_reg2, logit"
## Null:  "clm, as.factor(s_relationship) ~ 1, data2_wide_pass_reg2, logit"                                                                           
## 
## $Pseudo.R.squared.for.model.vs.null
##                              Pseudo.R.squared
## McFadden                            0.0309605
## Cox and Snell (ML)                  0.1379690
## Nagelkerke (Cragg and Uhler)        0.1391200
## 
## $Likelihood.ratio.test
##  Df.diff LogLik.diff  Chisq   p.value
##      -10     -32.959 65.918 2.704e-10
## 
## $Number.of.observations
##           
## Model: 444
## Null:  444
## 
## $Messages
## [1] "Note: For models fit with REML, these statistics are based on refitting with ML"
## 
## $Warnings
## [1] "None"
```

```
H1test_pass2 = emmeans(relationship_model8_pass2, ~ H1_interaction)
pairs(H1test_pass2, adjust = "tukey")
```

```
##  contrast                                               estimate   SE  df
##  no disclaimer.old guideline - disclaimer.new guideline   -0.352 0.17 Inf
##  z.ratio p.value
##   -2.074  0.0381
## 
## Results are averaged over the levels of: summary1, s_sex, s_school, s_interest 
## Note: contrasts are still on the as.factor scale
```

```
cld(H1test_pass2, Letters = letters)
```

```
##  H1_interaction              emmean    SE  df asymp.LCL asymp.UCL .group
##  no disclaimer.old guideline  0.295 0.155 Inf  -0.00851     0.598  a    
##  disclaimer.new guideline     0.647 0.166 Inf   0.32274     0.972   b   
## 
## Results are averaged over the levels of: summary1, s_sex, s_school, s_interest 
## Results are given on the as.factor (not the response) scale. 
## Confidence level used: 0.95 
## Note: contrasts are still on the as.factor scale 
## significance level used: alpha = 0.05 
## NOTE: If two or more means share the same grouping symbol,
##       then we cannot show them to be different.
##       But we also did not show them to be the same.
```

### H1 Graphical

```
describeBy(data2_wide_pass_reg$s_relationship,
           data2_wide_pass_reg$H1_interaction)
```

```
## 
##  Descriptive statistics by group 
## group: no disclaimer.old guideline
##    vars   n mean   sd median trimmed  mad min max range skew kurtosis  se
## X1    1 240 0.51 3.02      0    0.45 2.97  -7   8    15 0.19    -0.46 0.2
## ------------------------------------------------------------ 
## group: no disclaimer.new guideline
##    vars   n mean   sd median trimmed  mad min max range skew kurtosis   se
## X1    1 424 0.45 3.21      0    0.32 2.97  -6   8    14 0.25    -0.63 0.16
## ------------------------------------------------------------ 
## group: disclaimer.new guideline
##    vars   n mean   sd median trimmed  mad min max range skew kurtosis   se
## X1    1 450 0.91 3.64      0    0.74 2.97  -6   8    14 0.38    -0.94 0.17
```

```
H1_bar <- ggplot(data2_wide_pass_reg, aes(H1_interaction,
                                       s_relationship)) +
  stat_summary(fun = mean, geom = "bar", fill = "White",
               colour = "Black") + stat_summary(fun.data = 
                                                  mean_cl_normal,
                                                geom = "pointrange") +
  labs(x = "Condition", y = "Relationship Knowledge Score")
H1_bar
```

```
## Warning: Removed 31 rows containing non-finite values (`stat_summary()`).
## Removed 31 rows containing non-finite values (`stat_summary()`).
```

```
data2_wide_pass_reg$H1_interaction <- mapvalues(data2_wide_pass_reg$H1_interaction,
                                                c("no disclaimer.old guideline",
                                                  "no disclaimer.new guideline",
                                                  "disclaimer.new guideline"),
                                                c("old, no disclaimer",
                                                  "new, no disclaimer",
                                                  "new, disclaimer"))

H1_boxplot <- ggplot(data2_wide_pass_reg, aes(H1_interaction, s_relationship,
                                              fill = H1_interaction))
H1_boxplot <- H1_boxplot + geom_boxplot() +
  theme_classic() + theme(legend.position = "none",
  panel.grid.major = element_blank(),
  panel.grid.minor = element_blank(),
  panel.background = element_blank(),
  axis.title = element_text(face = "bold"),
  text = element_text(face = "bold"),
  axis.text = element_text(face = "bold"),
  legend.title = element_text(face = "bold"))+
  labs(x = "Condition", y = "Realtionship Knowledge Score") +
  scale_fill_brewer(palette = "Blues")
H1_boxplot
```

```
## Warning: Removed 31 rows containing non-finite values (`stat_boxplot()`).
```

```
ggsave("H1_boxplot.png", plot = H1_boxplot,
       scale = 1, dpi = 600)
```

```
## Saving 7 x 5 in image
```

```
## Warning: Removed 31 rows containing non-finite values (`stat_boxplot()`).
```

## H2

### H2a

```
H2a_pass <- subset(data2_wide_pass, condition == 2|condition == 4|condition == 6)
View(H2a_pass)

describeBy(H2a_pass$s_extent,H2a_pass$disclaimer)
```

```
## 
##  Descriptive statistics by group 
## group: no disclaimer
##    vars   n mean   sd median trimmed  mad min max range skew kurtosis   se
## X1    1 241 0.93 2.27      1    0.87 2.97  -4   6    10 0.22    -0.63 0.15
## ------------------------------------------------------------ 
## group: disclaimer
##    vars   n mean   sd median trimmed  mad min max range skew kurtosis   se
## X1    1 453 1.07 2.45      1    1.02 2.97  -6   6    12 0.12    -0.77 0.12
```

```
wilcox.test(s_extent~disclaimer, data = H2a_pass, exact = FALSE,
            conf.int = TRUE)
```

```
## 
##  Wilcoxon rank sum test with continuity correction
## 
## data:  s_extent by disclaimer
## W = 52872, p-value = 0.4913
## alternative hypothesis: true location shift is not equal to 0
## 95 percent confidence interval:
##  -1.072637e-05  8.141003e-05
## sample estimates:
## difference in location 
##          -4.522336e-05
```

#### H2a post hoc

```
H2a_pass_1 <- subset(data2_wide_pass, condition == 2|condition == 6)
View(H2a_pass_1)

describeBy(H2a_pass_1$s_extent,H2a_pass_1$disclaimer)
```

```
## 
##  Descriptive statistics by group 
## group: no disclaimer
##    vars   n mean   sd median trimmed  mad min max range skew kurtosis   se
## X1    1 241 0.93 2.27      1    0.87 2.97  -4   6    10 0.22    -0.63 0.15
## ------------------------------------------------------------ 
## group: disclaimer
##    vars   n mean   sd median trimmed  mad min max range skew kurtosis   se
## X1    1 249 0.87 2.43      0    0.81 2.97  -6   6    12 0.15    -0.69 0.15
```

```
wilcox.test(s_extent~disclaimer, data = H2a_pass_1, exact = FALSE,
            conf.int = TRUE)
```

```
## 
##  Wilcoxon rank sum test with continuity correction
## 
## data:  s_extent by disclaimer
## W = 30495, p-value = 0.7521
## alternative hypothesis: true location shift is not equal to 0
## 95 percent confidence interval:
##  -1.699256e-05  1.121775e-05
## sample estimates:
## difference in location 
##           2.572533e-05
```

```
H2a_pass_2 <- subset(data2_wide_pass, condition == 4|condition == 6)
View(H2a_pass_2)

describeBy(H2a_pass_2$s_extent,H2a_pass_2$disclaimer)
```

```
## 
##  Descriptive statistics by group 
## group: no disclaimer
##    vars   n mean   sd median trimmed  mad min max range skew kurtosis   se
## X1    1 241 0.93 2.27      1    0.87 2.97  -4   6    10 0.22    -0.63 0.15
## ------------------------------------------------------------ 
## group: disclaimer
##    vars   n mean   sd median trimmed  mad min max range skew kurtosis   se
## X1    1 204 1.31 2.47      1    1.27 2.97  -4   6    10 0.07    -0.89 0.17
```

```
wilcox.test(s_extent~disclaimer, data = H2a_pass_2, exact = FALSE,
            conf.int = TRUE)
```

```
## 
##  Wilcoxon rank sum test with continuity correction
## 
## data:  s_extent by disclaimer
## W = 22377, p-value = 0.09958
## alternative hypothesis: true location shift is not equal to 0
## 95 percent confidence interval:
##  -9.999351e-01  3.470513e-05
## sample estimates:
## difference in location 
##           -4.93409e-05
```

### H2b

```
H2b_pass <- subset(data2_wide_pass, condition == 1| condition == 2| condition == 3|
                condition == 4)
View(H2b_pass)

describeBy(H2b_pass$s_extent,H2b_pass$disclaimer)
```

```
## 
##  Descriptive statistics by group 
## group: no disclaimer
##    vars   n mean   sd median trimmed  mad min max range skew kurtosis   se
## X1    1 430 0.69 2.24      0    0.64 2.97  -6   6    12 0.15    -0.39 0.11
## ------------------------------------------------------------ 
## group: disclaimer
##    vars   n mean   sd median trimmed  mad min max range skew kurtosis   se
## X1    1 453 1.07 2.45      1    1.02 2.97  -6   6    12 0.12    -0.77 0.12
```

```
wilcox.test(s_extent~disclaimer, data = H2b_pass, exact = FALSE,
            conf.int = TRUE)
```

```
## 
##  Wilcoxon rank sum test with continuity correction
## 
## data:  s_extent by disclaimer
## W = 89460, p-value = 0.03434
## alternative hypothesis: true location shift is not equal to 0
## 95 percent confidence interval:
##  -9.999829e-01 -3.957081e-05
## sample estimates:
## difference in location 
##          -4.508197e-05
```

#### H2b post hoc

```
H2b_pass_1 <- subset(data2_wide, condition == 1| condition == 2)
View(H2b_pass_1)
describeBy(H2b_pass_1$s_extent,H2b_pass_1$disclaimer)
```

```
## 
##  Descriptive statistics by group 
## group: no disclaimer
##    vars   n mean   sd median trimmed  mad min max range skew kurtosis   se
## X1    1 327 0.51 2.21      0    0.43 2.97  -4   6    10 0.25    -0.46 0.12
## ------------------------------------------------------------ 
## group: disclaimer
##    vars   n mean   sd median trimmed  mad min max range skew kurtosis   se
## X1    1 340 0.52 2.38      0    0.44 2.97  -6   6    12 0.22    -0.49 0.13
```

```
wilcox.test(s_extent~disclaimer, data = H2b_pass_1, exact = FALSE,
            conf.int = TRUE)
```

```
## 
##  Wilcoxon rank sum test with continuity correction
## 
## data:  s_extent by disclaimer
## W = 55838, p-value = 0.9198
## alternative hypothesis: true location shift is not equal to 0
## 95 percent confidence interval:
##  -1.851467e-05  3.167506e-05
## sample estimates:
## difference in location 
##           4.181901e-05
```

```
H2b_pass_2 <- subset(data2_wide_pass, condition == 3| condition == 4)
View(H2b_pass_2)
describeBy(H2b_pass_2$s_extent,H2b_pass_2$disclaimer)
```

```
## 
##  Descriptive statistics by group 
## group: no disclaimer
##    vars   n mean   sd median trimmed  mad min max range skew kurtosis   se
## X1    1 213 0.53 2.16      0    0.47 2.97  -6   6    12 0.12    -0.09 0.15
## ------------------------------------------------------------ 
## group: disclaimer
##    vars   n mean   sd median trimmed  mad min max range skew kurtosis   se
## X1    1 204 1.31 2.47      1    1.27 2.97  -4   6    10 0.07    -0.89 0.17
```

```
wilcox.test(s_extent~disclaimer, data = H2b_pass_2, exact = FALSE,
            conf.int = TRUE)
```

```
## 
##  Wilcoxon rank sum test with continuity correction
## 
## data:  s_extent by disclaimer
## W = 17879, p-value = 0.001565
## alternative hypothesis: true location shift is not equal to 0
## 95 percent confidence interval:
##  -1.000023e+00 -4.802819e-05
## sample estimates:
## difference in location 
##             -0.9999337
```

```
H2b_pass_3 <- subset(data2_wide_pass, condition == 1| condition == 4)
View(H2b_pass_3)
describeBy(H2b_pass_3$s_extent,H2b_pass_3$disclaimer)
```

```
## 
##  Descriptive statistics by group 
## group: no disclaimer
##    vars   n mean   sd median trimmed  mad min max range skew kurtosis   se
## X1    1 217 0.85 2.31      1     0.8 2.97  -4   6    10 0.14    -0.68 0.16
## ------------------------------------------------------------ 
## group: disclaimer
##    vars   n mean   sd median trimmed  mad min max range skew kurtosis   se
## X1    1 204 1.31 2.47      1    1.27 2.97  -4   6    10 0.07    -0.89 0.17
```

```
wilcox.test(s_extent~disclaimer, data = H2b_pass_3, exact = FALSE,
            conf.int = TRUE)
```

```
## 
##  Wilcoxon rank sum test with continuity correction
## 
## data:  s_extent by disclaimer
## W = 19853, p-value = 0.06502
## alternative hypothesis: true location shift is not equal to 0
## 95 percent confidence interval:
##  -9.999654e-01  2.904643e-05
## sample estimates:
## difference in location 
##          -2.018815e-05
```

```
H2b_pass_4 <- subset(data2_wide_pass, condition == 2| condition == 3)
View(H2b_pass_4)
describeBy(H2b_pass_4$s_extent,H2b_pass_4$disclaimer)
```

```
## 
##  Descriptive statistics by group 
## group: no disclaimer
##    vars   n mean   sd median trimmed  mad min max range skew kurtosis   se
## X1    1 213 0.53 2.16      0    0.47 2.97  -6   6    12 0.12    -0.09 0.15
## ------------------------------------------------------------ 
## group: disclaimer
##    vars   n mean   sd median trimmed  mad min max range skew kurtosis   se
## X1    1 249 0.87 2.43      0    0.81 2.97  -6   6    12 0.15    -0.69 0.15
```

```
wilcox.test(s_extent~disclaimer, data = H2b_pass_4, exact = FALSE,
            conf.int = TRUE)
```

```
## 
##  Wilcoxon rank sum test with continuity correction
## 
## data:  s_extent by disclaimer
## W = 24692, p-value = 0.1965
## alternative hypothesis: true location shift is not equal to 0
## 95 percent confidence interval:
##  -9.999545e-01  2.824539e-05
## sample estimates:
## difference in location 
##          -7.797653e-05
```

### H2 Logistic Regression

```
data2_wide_pass$H2_interaction <- data2_wide_pass$H1_interaction
table(data2_wide_pass$H2_interaction)
```

```
## 
## no disclaimer.old guideline no disclaimer.new guideline 
##                         247                         441 
##    disclaimer.new guideline 
##                         694
```

```
data2_wide_pass_reg <- subset(data2_wide_pass, condition != 5)
View(data2_wide_pass_reg)

extent_null_pass <- clm(as.factor(s_extent) ~ 1, data = data2_wide_pass_reg,
                        link = "logit")

extent_model1_pass <- clm(as.factor(s_extent) ~ H2_interaction,
                          data = data2_wide_pass_reg, link = "logit")
anova(extent_null_pass,extent_model1_pass)
```

```
## Likelihood ratio tests of cumulative link models:
##  
##                    formula:                             link: threshold:
## extent_null_pass   as.factor(s_extent) ~ 1              logit flexible  
## extent_model1_pass as.factor(s_extent) ~ H2_interaction logit flexible  
## 
##                    no.par    AIC  logLik LR.stat df Pr(>Chisq)
## extent_null_pass       12 4901.9 -2438.9                      
## extent_model1_pass     14 4901.3 -2436.6  4.5981  2     0.1004
```

```
extent_model2_pass <- clm(as.factor(s_extent) ~ H2_interaction + summary1,
                     data = data2_wide_pass_reg, link = "logit")
anova(extent_model1_pass,extent_model2_pass)
```

```
## Likelihood ratio tests of cumulative link models:
##  
##                    formula:                                        link:
## extent_model1_pass as.factor(s_extent) ~ H2_interaction            logit
## extent_model2_pass as.factor(s_extent) ~ H2_interaction + summary1 logit
##                    threshold:
## extent_model1_pass flexible  
## extent_model2_pass flexible  
## 
##                    no.par    AIC  logLik LR.stat df Pr(>Chisq)
## extent_model1_pass     14 4901.3 -2436.6                      
## extent_model2_pass     15 4903.3 -2436.6   0.002  1     0.9641
```

```
extent_model3_pass <- clm(as.factor(s_extent) ~ H2_interaction + summary1 + 
                            s_age, data = data2_wide_pass_reg, 
                     link = "logit")
anova(extent_model1_pass,extent_model3_pass)
```

```
## Likelihood ratio tests of cumulative link models:
##  
##                    formula:                                               
## extent_model1_pass as.factor(s_extent) ~ H2_interaction                   
## extent_model3_pass as.factor(s_extent) ~ H2_interaction + summary1 + s_age
##                    link: threshold:
## extent_model1_pass logit flexible  
## extent_model3_pass logit flexible  
## 
##                    no.par    AIC  logLik LR.stat df Pr(>Chisq)    
## extent_model1_pass     14 4901.3 -2436.6                          
## extent_model3_pass     16 4889.2 -2428.6  16.109  2  0.0003177 ***
## ---
## Signif. codes:  0 '***' 0.001 '**' 0.01 '*' 0.05 '.' 0.1 ' ' 1
```

```
extent_model4_pass <- clm(as.factor(s_extent) ~ H2_interaction + 
                       summary1 + s_age + s_sex,
                       data = data2_wide_pass_reg, link = "logit")
anova(extent_model3_pass,extent_model4_pass)
```

```
## Likelihood ratio tests of cumulative link models:
##  
##                    formula:                                                       
## extent_model3_pass as.factor(s_extent) ~ H2_interaction + summary1 + s_age        
## extent_model4_pass as.factor(s_extent) ~ H2_interaction + summary1 + s_age + s_sex
##                    link: threshold:
## extent_model3_pass logit flexible  
## extent_model4_pass logit flexible  
## 
##                    no.par    AIC  logLik LR.stat df Pr(>Chisq)
## extent_model3_pass     16 4889.2 -2428.6                      
## extent_model4_pass     17 4890.0 -2428.0  1.1912  1     0.2751
```

```
extent_model5_pass <- clm(as.factor(s_extent) ~ H2_interaction + summary1 +
                            s_age + s_sex + s_school, 
                          data = data2_wide_pass_reg, link = "logit")
anova(extent_model3_pass,extent_model5_pass)
```

```
## Likelihood ratio tests of cumulative link models:
##  
##                    formula:                                                                  
## extent_model3_pass as.factor(s_extent) ~ H2_interaction + summary1 + s_age                   
## extent_model5_pass as.factor(s_extent) ~ H2_interaction + summary1 + s_age + s_sex + s_school
##                    link: threshold:
## extent_model3_pass logit flexible  
## extent_model5_pass logit flexible  
## 
##                    no.par    AIC  logLik LR.stat df Pr(>Chisq)    
## extent_model3_pass     16 4889.2 -2428.6                          
## extent_model5_pass     19 4827.2 -2394.6  67.932  3  1.183e-14 ***
## ---
## Signif. codes:  0 '***' 0.001 '**' 0.01 '*' 0.05 '.' 0.1 ' ' 1
```

```
extent_model6_pass <- clm(as.factor(s_extent) ~ H2_interaction + summary1 + 
                            s_age + s_sex + s_school + 
                            as.factor(s_interest), data = data2_wide_pass_reg, 
                     link = "logit")
anova(extent_model5_pass,extent_model6_pass)
```

```
## Likelihood ratio tests of cumulative link models:
##  
##                    formula:                                                                                          
## extent_model5_pass as.factor(s_extent) ~ H2_interaction + summary1 + s_age + s_sex + s_school                        
## extent_model6_pass as.factor(s_extent) ~ H2_interaction + summary1 + s_age + s_sex + s_school + as.factor(s_interest)
##                    link: threshold:
## extent_model5_pass logit flexible  
## extent_model6_pass logit flexible  
## 
##                    no.par    AIC  logLik LR.stat df Pr(>Chisq)  
## extent_model5_pass     19 4827.2 -2394.6                        
## extent_model6_pass     23 4824.1 -2389.1  11.137  4    0.02507 *
## ---
## Signif. codes:  0 '***' 0.001 '**' 0.01 '*' 0.05 '.' 0.1 ' ' 1
```

```
summary(extent_model6_pass)
```

```
## formula: 
## as.factor(s_extent) ~ H2_interaction + summary1 + s_age + s_sex + s_school + as.factor(s_interest)
## data:    data2_wide_pass_reg
## 
##  link  threshold nobs logLik   AIC     niter max.grad cond.H 
##  logit flexible  1124 -2389.05 4824.11 7(0)  7.79e-08 1.1e+06
## 
## Coefficients:
##                                            Estimate Std. Error z value Pr(>|z|)
## H2_interactionno disclaimer.new guideline -0.147638   0.140490  -1.051  0.29331
## H2_interactiondisclaimer.new guideline     0.117724   0.140369   0.839  0.40165
## summary1Faerber                            0.020479   0.104929   0.195  0.84526
## s_age                                     -0.011816   0.003469  -3.406  0.00066
## s_sexmale                                  0.078822   0.106339   0.741  0.45855
## s_schoolReal                               0.565252   0.134680   4.197  2.7e-05
## s_schoolAbi                                1.101721   0.133843   8.231  < 2e-16
## as.factor(s_interest)5                    -0.153299   0.166794  -0.919  0.35805
## as.factor(s_interest)6                    -0.142009   0.165998  -0.855  0.39228
## as.factor(s_interest)7                    -0.295302   0.181130  -1.630  0.10303
## as.factor(s_interest)8                    -0.561386   0.182576  -3.075  0.00211
##                                              
## H2_interactionno disclaimer.new guideline    
## H2_interactiondisclaimer.new guideline       
## summary1Faerber                              
## s_age                                     ***
## s_sexmale                                    
## s_schoolReal                              ***
## s_schoolAbi                               ***
## as.factor(s_interest)5                       
## as.factor(s_interest)6                       
## as.factor(s_interest)7                       
## as.factor(s_interest)8                    ** 
## ---
## Signif. codes:  0 '***' 0.001 '**' 0.01 '*' 0.05 '.' 0.1 ' ' 1
## 
## Threshold coefficients:
##       Estimate Std. Error z value
## -6|-5  -6.6085     0.7542  -8.762
## -5|-4  -6.2020     0.6342  -9.779
## -4|-3  -4.2889     0.3449 -12.436
## -3|-2  -3.3854     0.2997 -11.294
## -2|-1  -1.7076     0.2704  -6.315
## -1|0   -1.1692     0.2669  -4.381
## 0|1    -0.1881     0.2651  -0.710
## 1|2     0.2877     0.2657   1.083
## 2|3     1.0299     0.2677   3.848
## 3|4     1.5009     0.2702   5.554
## 4|5     2.8465     0.2905   9.799
## 5|6     3.4037     0.3093  11.004
## (21 Beobachtungen als fehlend gelöscht)
```

```
exp(coef(extent_model6_pass))
```

```
##                                     -6|-5 
##                               0.001348841 
##                                     -5|-4 
##                               0.002025423 
##                                     -4|-3 
##                               0.013720079 
##                                     -3|-2 
##                               0.033863684 
##                                     -2|-1 
##                               0.181304316 
##                                      -1|0 
##                               0.310600539 
##                                       0|1 
##                               0.828508464 
##                                       1|2 
##                               1.333313271 
##                                       2|3 
##                               2.800707691 
##                                       3|4 
##                               4.485916176 
##                                       4|5 
##                              17.226841649 
##                                       5|6 
##                              30.074089560 
## H2_interactionno disclaimer.new guideline 
##                               0.862743658 
##    H2_interactiondisclaimer.new guideline 
##                               1.124933886 
##                           summary1Faerber 
##                               1.020690062 
##                                     s_age 
##                               0.988253475 
##                                 s_sexmale 
##                               1.082011416 
##                              s_schoolReal 
##                               1.759891565 
##                               s_schoolAbi 
##                               3.009341248 
##                    as.factor(s_interest)5 
##                               0.857873532 
##                    as.factor(s_interest)6 
##                               0.867613364 
##                    as.factor(s_interest)7 
##                               0.744306662 
##                    as.factor(s_interest)8 
##                               0.570417685
```

```
exp(confint(extent_model6_pass))
```

```
##                                               2.5 %    97.5 %
## H2_interactionno disclaimer.new guideline 0.6550016 1.1362689
## H2_interactiondisclaimer.new guideline    0.8544239 1.4815155
## summary1Faerber                           0.8309360 1.2538099
## s_age                                     0.9815466 0.9949903
## s_sexmale                                 0.8784657 1.3328799
## s_schoolReal                              1.3520751 2.2926483
## s_schoolAbi                               2.3166961 3.9154156
## as.factor(s_interest)5                    0.6184745 1.1895039
## as.factor(s_interest)6                    0.6264859 1.2011560
## as.factor(s_interest)7                    0.5216627 1.0613352
## as.factor(s_interest)8                    0.3985866 0.8155419
```

```
nagelkerke(fit = extent_model6_pass, null = extent_null_pass)
```

```
## $Models
##                                                                                                                                             
## Model: "clm, as.factor(s_extent) ~ H2_interaction + summary1 + s_age + s_sex + s_school + as.factor(s_interest), data2_wide_pass_reg, logit"
## Null:  "clm, as.factor(s_extent) ~ 1, data2_wide_pass_reg, logit"                                                                           
## 
## $Pseudo.R.squared.for.model.vs.null
##                              Pseudo.R.squared
## McFadden                            0.0204548
## Cox and Snell (ML)                  0.0849428
## Nagelkerke (Cragg and Uhler)        0.0860651
## 
## $Likelihood.ratio.test
##  Df.diff LogLik.diff  Chisq    p.value
##      -11     -49.888 99.776 1.9778e-16
## 
## $Number.of.observations
##            
## Model: 1124
## Null:  1124
## 
## $Messages
## [1] "Note: For models fit with REML, these statistics are based on refitting with ML"
## 
## $Warnings
## [1] "None"
```

```
H2test_pass = emmeans(extent_model6_pass, ~ H2_interaction)
pairs(H2test_pass, adjust = "tukey")
```

```
##  contrast                                                  estimate    SE  df
##  no disclaimer.old guideline - no disclaimer.new guideline    0.148 0.140 Inf
##  no disclaimer.old guideline - disclaimer.new guideline      -0.118 0.140 Inf
##  no disclaimer.new guideline - disclaimer.new guideline      -0.265 0.119 Inf
##  z.ratio p.value
##    1.051  0.5447
##   -0.839  0.6789
##   -2.236  0.0653
## 
## Results are averaged over the levels of: summary1, s_sex, s_school, s_interest 
## Note: contrasts are still on the as.factor scale 
## P value adjustment: tukey method for comparing a family of 3 estimates
```

```
cld(H2test_pass, Letters = letters)
```

```
##  H2_interaction              emmean    SE  df asymp.LCL asymp.UCL .group
##  no disclaimer.new guideline  0.875 0.142 Inf     0.597      1.15  a    
##  no disclaimer.old guideline  1.023 0.161 Inf     0.708      1.34  a    
##  disclaimer.new guideline     1.141 0.141 Inf     0.863      1.42  a    
## 
## Results are averaged over the levels of: summary1, s_sex, s_school, s_interest 
## Results are given on the as.factor (not the response) scale. 
## Confidence level used: 0.95 
## Note: contrasts are still on the as.factor scale 
## P value adjustment: tukey method for comparing a family of 3 estimates 
## significance level used: alpha = 0.05 
## NOTE: If two or more means share the same grouping symbol,
##       then we cannot show them to be different.
##       But we also did not show them to be the same.
```

#### Logistic Regression by Group

```
data2_wide_pass_reg3 <- subset(data2_wide_pass, condition == 2| condition == 6)
View(data2_wide_pass_reg3)

extent_null_pass1 <- clm(as.factor(s_extent) ~ 1, data = data2_wide_pass_reg3, link = "logit")

extent_model8_pass1 <- clm(as.factor(s_extent) ~ H2_interaction + 
                       summary1 + s_age + s_sex +
                       s_school + as.factor(s_interest), data = data2_wide_pass_reg3, 
                     link = "logit")

summary(extent_model8_pass1)
```

```
## formula: 
## as.factor(s_extent) ~ H2_interaction + summary1 + s_age + s_sex + s_school + as.factor(s_interest)
## data:    data2_wide_pass_reg3
## 
##  link  threshold nobs logLik   AIC     niter max.grad cond.H 
##  logit flexible  490  -1034.81 2111.63 7(0)  3.06e-13 7.5e+05
## 
## Coefficients:
##                                         Estimate Std. Error z value Pr(>|z|)
## H2_interactiondisclaimer.new guideline -0.044804   0.159616  -0.281  0.77894
## summary1Faerber                         0.095749   0.160578   0.596  0.55099
## s_age                                  -0.014622   0.005158  -2.835  0.00459
## s_sexmale                               0.145365   0.162996   0.892  0.37248
## s_schoolReal                            0.624281   0.208019   3.001  0.00269
## s_schoolAbi                             1.223665   0.202689   6.037 1.57e-09
## as.factor(s_interest)5                 -0.686079   0.261132  -2.627  0.00861
## as.factor(s_interest)6                 -0.346777   0.261641  -1.325  0.18504
## as.factor(s_interest)7                 -0.610305   0.285482  -2.138  0.03253
## as.factor(s_interest)8                 -1.078512   0.273982  -3.936 8.27e-05
##                                           
## H2_interactiondisclaimer.new guideline    
## summary1Faerber                           
## s_age                                  ** 
## s_sexmale                                 
## s_schoolReal                           ** 
## s_schoolAbi                            ***
## as.factor(s_interest)5                 ** 
## as.factor(s_interest)6                    
## as.factor(s_interest)7                 *  
## as.factor(s_interest)8                 ***
## ---
## Signif. codes:  0 '***' 0.001 '**' 0.01 '*' 0.05 '.' 0.1 ' ' 1
## 
## Threshold coefficients:
##       Estimate Std. Error z value
## -6|-4 -6.86459    1.06688  -6.434
## -4|-3 -5.06113    0.55190  -9.170
## -3|-2 -3.67533    0.42583  -8.631
## -2|-1 -2.06712    0.38233  -5.407
## -1|0  -1.49388    0.37557  -3.978
## 0|1   -0.49938    0.37124  -1.345
## 1|2   -0.08956    0.37196  -0.241
## 2|3    0.64829    0.37515   1.728
## 3|4    1.23557    0.37937   3.257
## 4|5    2.53649    0.40768   6.222
## 5|6    3.03144    0.43258   7.008
## (8 Beobachtungen als fehlend gelöscht)
```

```
exp(coef(extent_model8_pass1))
```

```
##                                  -6|-4                                  -4|-3 
##                            0.001044105                            0.006338367 
##                                  -3|-2                                  -2|-1 
##                            0.025341049                            0.126550148 
##                                   -1|0                                    0|1 
##                            0.224499522                            0.606904850 
##                                    1|2                                    2|3 
##                            0.914330938                            1.912260879 
##                                    3|4                                    4|5 
##                            3.440345754                           12.635257566 
##                                    5|6 H2_interactiondisclaimer.new guideline 
##                           20.727031668                            0.956185263 
##                        summary1Faerber                                  s_age 
##                            1.100482296                            0.985484742 
##                              s_sexmale                           s_schoolReal 
##                            1.156461331                            1.866902863 
##                            s_schoolAbi                 as.factor(s_interest)5 
##                            3.399625174                            0.503546744 
##                 as.factor(s_interest)6                 as.factor(s_interest)7 
##                            0.706962760                            0.543185084 
##                 as.factor(s_interest)8 
##                            0.340101191
```

```
exp(confint(extent_model8_pass1))
```

```
##                                            2.5 %    97.5 %
## H2_interactiondisclaimer.new guideline 0.6991790 1.3074693
## summary1Faerber                        0.8033906 1.5080332
## s_age                                  0.9755443 0.9954825
## s_sexmale                              0.8402997 1.5923638
## s_schoolReal                           1.2430276 2.8106958
## s_schoolAbi                            2.2897012 5.0701958
## as.factor(s_interest)5                 0.3011995 0.8389337
## as.factor(s_interest)6                 0.4227658 1.1798470
## as.factor(s_interest)7                 0.3099438 0.9498413
## as.factor(s_interest)8                 0.1982278 0.5806786
```

```
nagelkerke(fit = extent_model8_pass1, null = extent_null_pass1)
```

```
## $Models
##                                                                                                                                              
## Model: "clm, as.factor(s_extent) ~ H2_interaction + summary1 + s_age + s_sex + s_school + as.factor(s_interest), data2_wide_pass_reg3, logit"
## Null:  "clm, as.factor(s_extent) ~ 1, data2_wide_pass_reg3, logit"                                                                           
## 
## $Pseudo.R.squared.for.model.vs.null
##                              Pseudo.R.squared
## McFadden                            0.0282131
## Cox and Snell (ML)                  0.1154040
## Nagelkerke (Cragg and Uhler)        0.1169180
## 
## $Likelihood.ratio.test
##  Df.diff LogLik.diff  Chisq    p.value
##      -10     -30.043 60.086 3.4913e-09
## 
## $Number.of.observations
##           
## Model: 490
## Null:  490
## 
## $Messages
## [1] "Note: For models fit with REML, these statistics are based on refitting with ML"
## 
## $Warnings
## [1] "None"
```

```
H2test_pass1 = emmeans(extent_model8_pass1, ~ H2_interaction)
pairs(H2test_pass1, adjust = "tukey")
```

```
##  contrast                                               estimate   SE  df
##  no disclaimer.old guideline - disclaimer.new guideline   0.0448 0.16 Inf
##  z.ratio p.value
##    0.281  0.7789
## 
## Results are averaged over the levels of: summary1, s_sex, s_school, s_interest 
## Note: contrasts are still on the as.factor scale
```

```
cld(H2test_pass1, Letters = letters)
```

```
##  H2_interaction              emmean    SE  df asymp.LCL asymp.UCL .group
##  disclaimer.new guideline     0.568 0.163 Inf     0.248     0.888  a    
##  no disclaimer.old guideline  0.613 0.166 Inf     0.288     0.938  a    
## 
## Results are averaged over the levels of: summary1, s_sex, s_school, s_interest 
## Results are given on the as.factor (not the response) scale. 
## Confidence level used: 0.95 
## Note: contrasts are still on the as.factor scale 
## significance level used: alpha = 0.05 
## NOTE: If two or more means share the same grouping symbol,
##       then we cannot show them to be different.
##       But we also did not show them to be the same.
```

```
data2_wide_pass_reg4 <- subset(data2_wide_pass, condition == 4| condition == 6)
View(data2_wide_pass_reg4)

extent_null_pass2 <- clm(as.factor(s_extent) ~ 1, data = data2_wide_pass_reg4, link = "logit")

extent_model8_pass2 <- clm(as.factor(s_extent) ~ H2_interaction + 
                       summary1 + s_age + s_sex +
                       s_school + as.factor(s_interest), data = data2_wide_pass_reg4, 
                     link = "logit")

summary(extent_model8_pass2)
```

```
## formula: 
## as.factor(s_extent) ~ H2_interaction + summary1 + s_age + s_sex + s_school + as.factor(s_interest)
## data:    data2_wide_pass_reg4
## 
##  link  threshold nobs logLik  AIC     niter max.grad cond.H 
##  logit flexible  445  -943.66 1927.33 6(0)  4.78e-08 6.5e+05
## 
## Coefficients:
##                                         Estimate Std. Error z value Pr(>|z|)
## H2_interactiondisclaimer.new guideline  0.278387   0.168382   1.653  0.09827
## summary1Faerber                        -0.108853   0.167853  -0.649  0.51666
## s_age                                  -0.003981   0.005547  -0.718  0.47291
## s_sexmale                              -0.011885   0.169055  -0.070  0.94395
## s_schoolReal                            0.474719   0.219729   2.160  0.03074
## s_schoolAbi                             1.073030   0.212220   5.056 4.28e-07
## as.factor(s_interest)5                 -0.124291   0.265590  -0.468  0.63980
## as.factor(s_interest)6                 -0.024801   0.267356  -0.093  0.92609
## as.factor(s_interest)7                 -0.349794   0.285345  -1.226  0.22025
## as.factor(s_interest)8                 -0.862544   0.291118  -2.963  0.00305
##                                           
## H2_interactiondisclaimer.new guideline .  
## summary1Faerber                           
## s_age                                     
## s_sexmale                                 
## s_schoolReal                           *  
## s_schoolAbi                            ***
## as.factor(s_interest)5                    
## as.factor(s_interest)6                    
## as.factor(s_interest)7                    
## as.factor(s_interest)8                 ** 
## ---
## Signif. codes:  0 '***' 0.001 '**' 0.01 '*' 0.05 '.' 0.1 ' ' 1
## 
## Threshold coefficients:
##       Estimate Std. Error z value
## -4|-3 -4.42779    0.60056  -7.373
## -3|-2 -3.16934    0.46954  -6.750
## -2|-1 -1.49384    0.41582  -3.593
## -1|0  -0.95047    0.40910  -2.323
## 0|1    0.03591    0.40670   0.088
## 1|2    0.47841    0.40851   1.171
## 2|3    1.20354    0.41197   2.921
## 3|4    1.68563    0.41575   4.054
## 4|5    2.97797    0.44392   6.708
## 5|6    3.41373    0.46278   7.377
## (8 Beobachtungen als fehlend gelöscht)
```

```
exp(coef(extent_model8_pass2))
```

```
##                                  -4|-3                                  -3|-2 
##                             0.01194086                             0.04203126 
##                                  -2|-1                                   -1|0 
##                             0.22450841                             0.38656013 
##                                    0|1                                    1|2 
##                             1.03656299                             1.61350067 
##                                    2|3                                    3|4 
##                             3.33189615                             5.39586770 
##                                    4|5                                    5|6 
##                            19.64792867                            30.37828672 
## H2_interactiondisclaimer.new guideline                        summary1Faerber 
##                             1.32099742                             0.89686220 
##                                  s_age                              s_sexmale 
##                             0.99602656                             0.98818499 
##                           s_schoolReal                            s_schoolAbi 
##                             1.60756289                             2.92422687 
##                 as.factor(s_interest)5                 as.factor(s_interest)6 
##                             0.88312240                             0.97550415 
##                 as.factor(s_interest)7                 as.factor(s_interest)8 
##                             0.70483297                             0.42208709
```

```
exp(confint(extent_model8_pass2))
```

```
##                                            2.5 %    97.5 %
## H2_interactiondisclaimer.new guideline 0.9499940 1.8387032
## summary1Faerber                        0.6452066 1.2461931
## s_age                                  0.9852479 1.0069212
## s_sexmale                              0.7093950 1.3766550
## s_schoolReal                           1.0456603 2.4757086
## s_schoolAbi                            1.9324547 4.4424361
## as.factor(s_interest)5                 0.5243658 1.4864294
## as.factor(s_interest)6                 0.5773738 1.6480811
## as.factor(s_interest)7                 0.4025247 1.2330482
## as.factor(s_interest)8                 0.2380862 0.7460035
```

```
nagelkerke(fit = extent_model8_pass2, null = extent_null_pass2)
```

```
## $Models
##                                                                                                                                              
## Model: "clm, as.factor(s_extent) ~ H2_interaction + summary1 + s_age + s_sex + s_school + as.factor(s_interest), data2_wide_pass_reg4, logit"
## Null:  "clm, as.factor(s_extent) ~ 1, data2_wide_pass_reg4, logit"                                                                           
## 
## $Pseudo.R.squared.for.model.vs.null
##                              Pseudo.R.squared
## McFadden                            0.0227249
## Cox and Snell (ML)                  0.0939145
## Nagelkerke (Cragg and Uhler)        0.0951552
## 
## $Likelihood.ratio.test
##  Df.diff LogLik.diff  Chisq    p.value
##      -10     -21.943 43.887 3.4497e-06
## 
## $Number.of.observations
##           
## Model: 445
## Null:  445
## 
## $Messages
## [1] "Note: For models fit with REML, these statistics are based on refitting with ML"
## 
## $Warnings
## [1] "None"
```

```
H2test_pass2 = emmeans(extent_model8_pass2, ~ H2_interaction)
pairs(H2test_pass2, adjust = "tukey")
```

```
##  contrast                                               estimate    SE  df
##  no disclaimer.old guideline - disclaimer.new guideline   -0.278 0.168 Inf
##  z.ratio p.value
##   -1.653  0.0983
## 
## Results are averaged over the levels of: summary1, s_sex, s_school, s_interest 
## Note: contrasts are still on the as.factor scale
```

```
cld(H2test_pass2, Letters = letters)
```

```
##  H2_interaction              emmean    SE  df asymp.LCL asymp.UCL .group
##  no disclaimer.old guideline 0.0166 0.131 Inf   -0.2403     0.273  a    
##  disclaimer.new guideline    0.2950 0.140 Inf    0.0203     0.570  a    
## 
## Results are averaged over the levels of: summary1, s_sex, s_school, s_interest 
## Results are given on the as.factor (not the response) scale. 
## Confidence level used: 0.95 
## Note: contrasts are still on the as.factor scale 
## significance level used: alpha = 0.05 
## NOTE: If two or more means share the same grouping symbol,
##       then we cannot show them to be different.
##       But we also did not show them to be the same.
```

### H2 Graphical

```
describeBy(data2_wide_pass_reg$s_extent,
           data2_wide_pass_reg$H2_interaction)
```

```
## 
##  Descriptive statistics by group 
## group: no disclaimer.old guideline
##    vars   n mean   sd median trimmed  mad min max range skew kurtosis   se
## X1    1 241 0.93 2.27      1    0.87 2.97  -4   6    10 0.22    -0.63 0.15
## ------------------------------------------------------------ 
## group: no disclaimer.new guideline
##    vars   n mean   sd median trimmed  mad min max range skew kurtosis   se
## X1    1 430 0.69 2.24      0    0.64 2.97  -6   6    12 0.15    -0.39 0.11
## ------------------------------------------------------------ 
## group: disclaimer.new guideline
##    vars   n mean   sd median trimmed  mad min max range skew kurtosis   se
## X1    1 453 1.07 2.45      1    1.02 2.97  -6   6    12 0.12    -0.77 0.12
```

```
H2_bar <- ggplot(data2_wide_pass_reg, aes(H2_interaction,
                                       s_extent)) +
  stat_summary(fun = mean, geom = "bar", fill = "White",
               colour = "Black") + stat_summary(fun.data = 
                                                  mean_cl_normal,
                                                geom = "pointrange") +
  labs(x = "Condition", y = "Extent of Evaluation Knowledge Score")
H2_bar
```

```
## Warning: Removed 21 rows containing non-finite values (`stat_summary()`).
## Removed 21 rows containing non-finite values (`stat_summary()`).
```

```
data2_wide_pass_reg$H2_interaction <- mapvalues(data2_wide_pass_reg$H2_interaction,
                                                c("no disclaimer.old guideline",
                                                  "no disclaimer.new guideline",
                                                  "disclaimer.new guideline"),
                                                c("old, no disclaimer",
                                                  "new, no disclaimer",
                                                  "new, disclaimer"))

H2_boxplot <- ggplot(data2_wide_pass_reg, aes(H2_interaction, s_extent,
                                              fill = H2_interaction))
H2_boxplot <- H2_boxplot + geom_boxplot() + theme_classic() + theme(
  legend.position = "none",
  panel.grid.major = element_blank(),
  panel.grid.minor = element_blank(),
  panel.background = element_blank(),
  axis.title = element_text(face = "bold"),
  axis.text = element_text(face = "bold"),
  legend.title = element_text(face = "bold"))+
  labs(x = "Condition", y = "Extent of Evaluation Knowledge Score") +
  scale_fill_brewer(palette = "Blues")
H2_boxplot
```

```
## Warning: Removed 21 rows containing non-finite values (`stat_boxplot()`).
```

## H3

### H3a

```
H3a_pass <- subset(data2_wide_pass, condition == 2|condition == 4|condition == 6)
View(H3a_pass)

describeBy(H3a_pass$s_diff,H3a_pass$disclaimer)
```

```
## 
##  Descriptive statistics by group 
## group: no disclaimer
##    vars   n mean sd median trimmed  mad min max range  skew kurtosis   se
## X1    1 239 0.58  2      0    0.56 1.48  -6   6    12 -0.07     0.24 0.13
## ------------------------------------------------------------ 
## group: disclaimer
##    vars   n mean  sd median trimmed  mad min max range skew kurtosis  se
## X1    1 438 0.43 2.1      0    0.32 2.97  -6   6    12 0.31     0.21 0.1
```

```
wilcox.test(s_diff~disclaimer, data = H3a_pass, exact = FALSE,
            conf.int = TRUE)
```

```
## 
##  Wilcoxon rank sum test with continuity correction
## 
## data:  s_diff by disclaimer
## W = 55496, p-value = 0.1862
## alternative hypothesis: true location shift is not equal to 0
## 95 percent confidence interval:
##  -5.555652e-05  3.627647e-05
## sample estimates:
## difference in location 
##           6.347909e-05
```

#### H3a post hoc

```
H3a_pass1 <- subset(data2_wide_pass, condition == 2|condition == 6)
View(H3a_pass1)
describeBy(H3a_pass1$s_diff,H3a_pass1$disclaimer)
```

```
## 
##  Descriptive statistics by group 
## group: no disclaimer
##    vars   n mean sd median trimmed  mad min max range  skew kurtosis   se
## X1    1 239 0.58  2      0    0.56 1.48  -6   6    12 -0.07     0.24 0.13
## ------------------------------------------------------------ 
## group: disclaimer
##    vars   n mean   sd median trimmed  mad min max range skew kurtosis   se
## X1    1 238 0.19 2.03      0     0.1 2.97  -4   6    10 0.31     0.08 0.13
```

```
wilcox.test(s_diff~disclaimer, data = H3a_pass1, exact = FALSE,
            conf.int = TRUE)
```

```
## 
##  Wilcoxon rank sum test with continuity correction
## 
## data:  s_diff by disclaimer
## W = 31996, p-value = 0.01615
## alternative hypothesis: true location shift is not equal to 0
## 95 percent confidence interval:
##  5.293119e-05 9.999779e-01
## sample estimates:
## difference in location 
##           6.013123e-05
```

```
H3a_pass2 <- subset(data2_wide_pass, condition == 4|condition == 6)
View(H3a_pass2)
describeBy(H3a_pass2$s_diff,H3a_pass2$disclaimer)
```

```
## 
##  Descriptive statistics by group 
## group: no disclaimer
##    vars   n mean sd median trimmed  mad min max range  skew kurtosis   se
## X1    1 239 0.58  2      0    0.56 1.48  -6   6    12 -0.07     0.24 0.13
## ------------------------------------------------------------ 
## group: disclaimer
##    vars   n mean   sd median trimmed  mad min max range skew kurtosis   se
## X1    1 200 0.72 2.14      0    0.61 2.97  -6   6    12 0.28      0.3 0.15
```

```
wilcox.test(s_diff~disclaimer, data = H3a_pass2, exact = FALSE,
            conf.int = TRUE)
```

```
## 
##  Wilcoxon rank sum test with continuity correction
## 
## data:  s_diff by disclaimer
## W = 23501, p-value = 0.7588
## alternative hypothesis: true location shift is not equal to 0
## 95 percent confidence interval:
##  -4.626346e-05  2.603510e-05
## sample estimates:
## difference in location 
##           -5.60431e-05
```

### H3b

```
H3b_pass <- subset(data2_wide_pass, condition == 1| condition == 2| condition == 3|
                condition == 4)
View(H3b_pass)

describeBy(H3b_pass$s_diff,H3b_pass$disclaimer)
```

```
## 
##  Descriptive statistics by group 
## group: no disclaimer
##    vars   n mean   sd median trimmed  mad min max range skew kurtosis  se
## X1    1 432 0.39 2.11      0    0.34 2.22  -6   6    12 0.07      0.3 0.1
## ------------------------------------------------------------ 
## group: disclaimer
##    vars   n mean  sd median trimmed  mad min max range skew kurtosis  se
## X1    1 438 0.43 2.1      0    0.32 2.97  -6   6    12 0.31     0.21 0.1
```

```
wilcox.test(s_diff~disclaimer, data = H3b_pass, exact = FALSE,
            conf.int = TRUE)
```

```
## 
##  Wilcoxon rank sum test with continuity correction
## 
## data:  s_diff by disclaimer
## W = 94509, p-value = 0.9783
## alternative hypothesis: true location shift is not equal to 0
## 95 percent confidence interval:
##  -7.612500e-05  4.959797e-05
## sample estimates:
## difference in location 
##          -3.882985e-05
```

#### H3b post hoc

```
H3b_pass1 <- subset(data2_wide_pass, condition == 1| condition == 2)
View(H3b_pass1)
describeBy(H3b_pass1$s_diff,H3b_pass1$disclaimer)
```

```
## 
##  Descriptive statistics by group 
## group: no disclaimer
##    vars   n mean   sd median trimmed  mad min max range  skew kurtosis   se
## X1    1 216 0.26 2.05      0    0.27 1.48  -6   6    12 -0.06     0.29 0.14
## ------------------------------------------------------------ 
## group: disclaimer
##    vars   n mean   sd median trimmed  mad min max range skew kurtosis   se
## X1    1 238 0.19 2.03      0     0.1 2.97  -4   6    10 0.31     0.08 0.13
```

```
wilcox.test(s_diff~disclaimer, data = H3b_pass1, exact = FALSE,
            conf.int = TRUE)
```

```
## 
##  Wilcoxon rank sum test with continuity correction
## 
## data:  s_diff by disclaimer
## W = 26715, p-value = 0.4599
## alternative hypothesis: true location shift is not equal to 0
## 95 percent confidence interval:
##  -4.963525e-06  8.116125e-06
## sample estimates:
## difference in location 
##           3.549421e-05
```

```
H3b_pass2 <- subset(data2_wide_pass, condition == 3| condition == 4)
View(H3b_pass2)
describeBy(H3b_pass2$s_diff,H3b_pass2$disclaimer)
```

```
## 
##  Descriptive statistics by group 
## group: no disclaimer
##    vars   n mean   sd median trimmed  mad min max range skew kurtosis   se
## X1    1 216 0.51 2.16      0    0.45 2.97  -6   6    12 0.17     0.22 0.15
## ------------------------------------------------------------ 
## group: disclaimer
##    vars   n mean   sd median trimmed  mad min max range skew kurtosis   se
## X1    1 200 0.72 2.14      0    0.61 2.97  -6   6    12 0.28      0.3 0.15
```

```
wilcox.test(s_diff~disclaimer, data = H3b_pass2, exact = FALSE,
            conf.int = TRUE)
```

```
## 
##  Wilcoxon rank sum test with continuity correction
## 
## data:  s_diff by disclaimer
## W = 20468, p-value = 0.3454
## alternative hypothesis: true location shift is not equal to 0
## 95 percent confidence interval:
##  -3.811281e-05  1.060134e-05
## sample estimates:
## difference in location 
##           -5.52108e-05
```

```
H3b_pass3 <- subset(data2_wide_pass, condition == 2| condition == 3)
View(H3b_pass3)
describeBy(H3b_pass3$s_diff,H3b_pass3$disclaimer)
```

```
## 
##  Descriptive statistics by group 
## group: no disclaimer
##    vars   n mean   sd median trimmed  mad min max range skew kurtosis   se
## X1    1 216 0.51 2.16      0    0.45 2.97  -6   6    12 0.17     0.22 0.15
## ------------------------------------------------------------ 
## group: disclaimer
##    vars   n mean   sd median trimmed  mad min max range skew kurtosis   se
## X1    1 238 0.19 2.03      0     0.1 2.97  -4   6    10 0.31     0.08 0.13
```

```
wilcox.test(s_diff~disclaimer, data = H3b_pass3, exact = FALSE,
            conf.int = TRUE)
```

```
## 
##  Wilcoxon rank sum test with continuity correction
## 
## data:  s_diff by disclaimer
## W = 27860, p-value = 0.1147
## alternative hypothesis: true location shift is not equal to 0
## 95 percent confidence interval:
##  -4.027259e-05  9.999986e-01
## sample estimates:
## difference in location 
##           5.850487e-05
```

```
H3b_pass4 <- subset(data2_wide_pass, condition == 1| condition == 4)
View(H3b_pass4)
describeBy(H3b_pass4$s_diff,H3b_pass4$disclaimer)
```

```
## 
##  Descriptive statistics by group 
## group: no disclaimer
##    vars   n mean   sd median trimmed  mad min max range  skew kurtosis   se
## X1    1 216 0.26 2.05      0    0.27 1.48  -6   6    12 -0.06     0.29 0.14
## ------------------------------------------------------------ 
## group: disclaimer
##    vars   n mean   sd median trimmed  mad min max range skew kurtosis   se
## X1    1 200 0.72 2.14      0    0.61 2.97  -6   6    12 0.28      0.3 0.15
```

```
wilcox.test(s_diff~disclaimer, data = H3b_pass4, exact = FALSE,
            conf.int = TRUE)
```

```
## 
##  Wilcoxon rank sum test with continuity correction
## 
## data:  s_diff by disclaimer
## W = 19467, p-value = 0.07539
## alternative hypothesis: true location shift is not equal to 0
## 95 percent confidence interval:
##  -0.9999553527  0.0000525234
## sample estimates:
## difference in location 
##          -4.526937e-05
```

### H3 Logistic Regression

```
data2_wide_pass$H3_interaction <- data2_wide_pass$H2_interaction
table(data2_wide_pass$H3_interaction)
```

```
## 
## no disclaimer.old guideline no disclaimer.new guideline 
##                         247                         441 
##    disclaimer.new guideline 
##                         694
```

```
data2_wide_pass_reg <- subset(data2_wide_pass, condition != 5)
View(data2_wide_pass_reg)

diff_null_pass <- clm(as.factor(s_diff) ~ 1, data = data2_wide_pass_reg, link = "logit")

diff_model1_pass <- clm(as.factor(s_diff) ~ H3_interaction, data = data2_wide_pass_reg,
                     link = "logit")
anova(diff_null_pass,diff_model1_pass)
```

```
## Likelihood ratio tests of cumulative link models:
##  
##                  formula:                           link: threshold:
## diff_null_pass   as.factor(s_diff) ~ 1              logit flexible  
## diff_model1_pass as.factor(s_diff) ~ H3_interaction logit flexible  
## 
##                  no.par    AIC  logLik LR.stat df Pr(>Chisq)
## diff_null_pass       12 4455.7 -2215.8                      
## diff_model1_pass     14 4457.4 -2214.7  2.2258  2     0.3286
```

```
diff_model2_pass <- clm(as.factor(s_diff) ~ H3_interaction + text_order, data = data2_wide_pass_reg, link = "logit")
anova(diff_null_pass,diff_model2_pass)
```

```
## Likelihood ratio tests of cumulative link models:
##  
##                  formula:                                        link:
## diff_null_pass   as.factor(s_diff) ~ 1                           logit
## diff_model2_pass as.factor(s_diff) ~ H3_interaction + text_order logit
##                  threshold:
## diff_null_pass   flexible  
## diff_model2_pass flexible  
## 
##                  no.par    AIC  logLik LR.stat df Pr(>Chisq)    
## diff_null_pass       12 4455.7 -2215.8                          
## diff_model2_pass     15 4436.4 -2203.2  25.314  3  1.328e-05 ***
## ---
## Signif. codes:  0 '***' 0.001 '**' 0.01 '*' 0.05 '.' 0.1 ' ' 1
```

```
diff_model3_pass <- clm(as.factor(s_diff) ~ H3_interaction + text_order + s_age, data = data2_wide_pass_reg, 
                     link = "logit")
anova(diff_model2_pass,diff_model3_pass)
```

```
## Likelihood ratio tests of cumulative link models:
##  
##                  formula:                                                link:
## diff_model2_pass as.factor(s_diff) ~ H3_interaction + text_order         logit
## diff_model3_pass as.factor(s_diff) ~ H3_interaction + text_order + s_age logit
##                  threshold:
## diff_model2_pass flexible  
## diff_model3_pass flexible  
## 
##                  no.par    AIC  logLik LR.stat df Pr(>Chisq)   
## diff_model2_pass     15 4436.4 -2203.2                         
## diff_model3_pass     16 4431.5 -2199.8  6.8496  1   0.008866 **
## ---
## Signif. codes:  0 '***' 0.001 '**' 0.01 '*' 0.05 '.' 0.1 ' ' 1
```

```
diff_model4_pass <- clm(as.factor(s_diff) ~ H3_interaction + text_order + s_age + s_sex, 
                   data = data2_wide_pass_reg, 
                     link = "logit")
anova(diff_model3_pass,diff_model4_pass)
```

```
## Likelihood ratio tests of cumulative link models:
##  
##                  formula:                                                       
## diff_model3_pass as.factor(s_diff) ~ H3_interaction + text_order + s_age        
## diff_model4_pass as.factor(s_diff) ~ H3_interaction + text_order + s_age + s_sex
##                  link: threshold:
## diff_model3_pass logit flexible  
## diff_model4_pass logit flexible  
## 
##                  no.par    AIC  logLik LR.stat df Pr(>Chisq)  
## diff_model3_pass     16 4431.5 -2199.8                        
## diff_model4_pass     17 4428.3 -2197.2  5.1723  1    0.02295 *
## ---
## Signif. codes:  0 '***' 0.001 '**' 0.01 '*' 0.05 '.' 0.1 ' ' 1
```

```
diff_model5_pass <- clm(as.factor(s_diff) ~ H3_interaction + text_order + s_age + s_sex +
                       s_school, data = data2_wide_pass_reg, 
                     link = "logit")
anova(diff_model4_pass,diff_model5_pass)
```

```
## Likelihood ratio tests of cumulative link models:
##  
##                  formula:                                                                  
## diff_model4_pass as.factor(s_diff) ~ H3_interaction + text_order + s_age + s_sex           
## diff_model5_pass as.factor(s_diff) ~ H3_interaction + text_order + s_age + s_sex + s_school
##                  link: threshold:
## diff_model4_pass logit flexible  
## diff_model5_pass logit flexible  
## 
##                  no.par    AIC  logLik LR.stat df Pr(>Chisq)
## diff_model4_pass     17 4428.3 -2197.2                      
## diff_model5_pass     19 4429.3 -2195.6    3.05  2     0.2176
```

```
diff_model6_pass <- clm(as.factor(s_diff) ~ H3_interaction  + text_order + s_age + s_sex +
                       s_school + as.factor(s_interest), data = data2_wide_pass_reg, 
                     link = "logit")
anova(diff_model4_pass,diff_model6_pass)
```

```
## Likelihood ratio tests of cumulative link models:
##  
##                  formula:                                                                                          
## diff_model4_pass as.factor(s_diff) ~ H3_interaction + text_order + s_age + s_sex                                   
## diff_model6_pass as.factor(s_diff) ~ H3_interaction + text_order + s_age + s_sex + s_school + as.factor(s_interest)
##                  link: threshold:
## diff_model4_pass logit flexible  
## diff_model6_pass logit flexible  
## 
##                  no.par    AIC  logLik LR.stat df Pr(>Chisq)
## diff_model4_pass     17 4428.3 -2197.2                      
## diff_model6_pass     23 4432.6 -2193.3  7.6897  6     0.2617
```

```
summary(diff_model6_pass)
```

```
## formula: 
## as.factor(s_diff) ~ H3_interaction + text_order + s_age + s_sex + s_school + as.factor(s_interest)
## data:    data2_wide_pass_reg
## 
##  link  threshold nobs logLik   AIC     niter max.grad cond.H 
##  logit flexible  1109 -2193.32 4432.64 8(2)  2.77e-09 8.9e+05
## 
## Coefficients:
##                                            Estimate Std. Error z value Pr(>|z|)
## H3_interactionno disclaimer.new guideline -0.194153   0.142134  -1.366   0.1719
## H3_interactiondisclaimer.new guideline    -0.194669   0.141730  -1.374   0.1696
## text_orderFaerber                          0.516386   0.107709   4.794 1.63e-06
## s_age                                     -0.007849   0.003501  -2.242   0.0250
## s_sexmale                                 -0.247799   0.108325  -2.288   0.0222
## s_schoolReal                               0.005230   0.136029   0.038   0.9693
## s_schoolAbi                                0.192445   0.132190   1.456   0.1454
## as.factor(s_interest)5                     0.124630   0.168940   0.738   0.4607
## as.factor(s_interest)6                     0.089459   0.170234   0.526   0.5992
## as.factor(s_interest)7                     0.103742   0.182736   0.568   0.5702
## as.factor(s_interest)8                    -0.200823   0.182186  -1.102   0.2703
##                                              
## H3_interactionno disclaimer.new guideline    
## H3_interactiondisclaimer.new guideline       
## text_orderFaerber                         ***
## s_age                                     *  
## s_sexmale                                 *  
## s_schoolReal                                 
## s_schoolAbi                                  
## as.factor(s_interest)5                       
## as.factor(s_interest)6                       
## as.factor(s_interest)7                       
## as.factor(s_interest)8                       
## ---
## Signif. codes:  0 '***' 0.001 '**' 0.01 '*' 0.05 '.' 0.1 ' ' 1
## 
## Threshold coefficients:
##        Estimate Std. Error z value
## -6|-5 -5.952747   0.564623 -10.543
## -5|-4 -5.728589   0.518428 -11.050
## -4|-3 -3.617525   0.306116 -11.817
## -3|-2 -3.210759   0.292886 -10.962
## -2|-1 -1.759454   0.271141  -6.489
## -1|0  -1.313645   0.268326  -4.896
## 0|1   -0.003668   0.264491  -0.014
## 1|2    0.502327   0.264802   1.897
## 2|3    1.780497   0.272814   6.526
## 3|4    2.053815   0.276476   7.429
## 4|5    3.304792   0.312878  10.563
## 5|6    3.841515   0.345440  11.121
## (36 Beobachtungen als fehlend gelöscht)
```

```
exp(coef(diff_model6_pass))
```

```
##                                     -6|-5 
##                               0.002598693 
##                                     -5|-4 
##                               0.003251662 
##                                     -4|-3 
##                               0.026849048 
##                                     -3|-2 
##                               0.040325981 
##                                     -2|-1 
##                               0.172138759 
##                                      -1|0 
##                               0.268838330 
##                                       0|1 
##                               0.996338650 
##                                       1|2 
##                               1.652562935 
##                                       2|3 
##                               5.932805631 
##                                       3|4 
##                               7.797591811 
##                                       4|5 
##                              27.242875959 
##                                       5|6 
##                              46.595999285 
## H3_interactionno disclaimer.new guideline 
##                               0.823531544 
##    H3_interactiondisclaimer.new guideline 
##                               0.823107147 
##                         text_orderFaerber 
##                               1.675959468 
##                                     s_age 
##                               0.992181668 
##                                 s_sexmale 
##                               0.780516664 
##                              s_schoolReal 
##                               1.005243926 
##                               s_schoolAbi 
##                               1.212209291 
##                    as.factor(s_interest)5 
##                               1.132729009 
##                    as.factor(s_interest)6 
##                               1.093582519 
##                    as.factor(s_interest)7 
##                               1.109314599 
##                    as.factor(s_interest)8 
##                               0.818056919
```

```
exp(confint(diff_model6_pass))
```

```
##                                               2.5 %    97.5 %
## H3_interactionno disclaimer.new guideline 0.6231962 1.0880869
## H3_interactiondisclaimer.new guideline    0.6233792 1.0866853
## text_orderFaerber                         1.3575010 2.0708138
## s_age                                     0.9853886 0.9990093
## s_sexmale                                 0.6310609 0.9649871
## s_schoolReal                              0.7699230 1.3124440
## s_schoolAbi                               0.9356472 1.5711167
## as.factor(s_interest)5                    0.8134840 1.5777806
## as.factor(s_interest)6                    0.7832952 1.5269502
## as.factor(s_interest)7                    0.7753767 1.5874767
## as.factor(s_interest)8                    0.5721892 1.1689666
```

```
nagelkerke(fit = diff_model6_pass, null = diff_null_pass)
```

```
## $Models
##                                                                                                                                             
## Model: "clm, as.factor(s_diff) ~ H3_interaction + text_order + s_age + s_sex + s_school + as.factor(s_interest), data2_wide_pass_reg, logit"
## Null:  "clm, as.factor(s_diff) ~ 1, data2_wide_pass_reg, logit"                                                                             
## 
## $Pseudo.R.squared.for.model.vs.null
##                              Pseudo.R.squared
## McFadden                            0.0101598
## Cox and Snell (ML)                  0.0397866
## Nagelkerke (Cragg and Uhler)        0.0405318
## 
## $Likelihood.ratio.test
##  Df.diff LogLik.diff  Chisq    p.value
##      -11     -22.513 45.025 4.8032e-06
## 
## $Number.of.observations
##            
## Model: 1109
## Null:  1109
## 
## $Messages
## [1] "Note: For models fit with REML, these statistics are based on refitting with ML"
## 
## $Warnings
## [1] "None"
```

```
H3test_pass = emmeans(diff_model6_pass, ~ H3_interaction)
pairs(H3test_pass, adjust = "tukey")
```

```
##  contrast                                                  estimate    SE  df
##  no disclaimer.old guideline - no disclaimer.new guideline 0.194153 0.142 Inf
##  no disclaimer.old guideline - disclaimer.new guideline    0.194669 0.142 Inf
##  no disclaimer.new guideline - disclaimer.new guideline    0.000515 0.121 Inf
##  z.ratio p.value
##    1.366  0.3589
##    1.374  0.3548
##    0.004  1.0000
## 
## Results are averaged over the levels of: text_order, s_sex, s_school, s_interest 
## Note: contrasts are still on the as.factor scale 
## P value adjustment: tukey method for comparing a family of 3 estimates
```

```
cld(H3test_pass, Letters = letters)
```

```
##  H3_interaction              emmean    SE  df asymp.LCL asymp.UCL .group
##  disclaimer.new guideline     0.502 0.124 Inf     0.258     0.746  a    
##  no disclaimer.new guideline  0.502 0.125 Inf     0.257     0.747  a    
##  no disclaimer.old guideline  0.696 0.146 Inf     0.411     0.982  a    
## 
## Results are averaged over the levels of: text_order, s_sex, s_school, s_interest 
## Results are given on the as.factor (not the response) scale. 
## Confidence level used: 0.95 
## Note: contrasts are still on the as.factor scale 
## P value adjustment: tukey method for comparing a family of 3 estimates 
## significance level used: alpha = 0.05 
## NOTE: If two or more means share the same grouping symbol,
##       then we cannot show them to be different.
##       But we also did not show them to be the same.
```

#### Logistic Regression by Group

```
data2_wide_pass_reg5 <- subset(data2_wide_pass, condition == 2 | condition == 6)
View(data2_wide_pass_reg5)

diff_null_pass1 <- clm(as.factor(s_diff) ~ 1, data = data2_wide_pass_reg5, link = "logit")

diff_model8_pass1 <- clm(as.factor(s_diff) ~ H3_interaction + text_order + s_age + s_sex +
                       s_school + as.factor(s_interest), data = data2_wide_pass_reg5, 
                     link = "logit")

summary(diff_model8_pass1)
```

```
## formula: 
## as.factor(s_diff) ~ H3_interaction + text_order + s_age + s_sex + s_school + as.factor(s_interest)
## data:    data2_wide_pass_reg5
## 
##  link  threshold nobs logLik  AIC     niter max.grad cond.H 
##  logit flexible  477  -932.58 1907.17 7(0)  3.80e-11 7.1e+05
## 
## Coefficients:
##                                         Estimate Std. Error z value Pr(>|z|)   
## H3_interactiondisclaimer.new guideline -0.388193   0.164305  -2.363  0.01815 * 
## text_orderFaerber                       0.486639   0.165159   2.946  0.00321 **
## s_age                                  -0.006172   0.005239  -1.178  0.23874   
## s_sexmale                              -0.345801   0.167879  -2.060  0.03942 * 
## s_schoolReal                            0.198953   0.211035   0.943  0.34581   
## s_schoolAbi                             0.539629   0.203704   2.649  0.00807 **
## as.factor(s_interest)5                 -0.094372   0.260464  -0.362  0.71711   
## as.factor(s_interest)6                 -0.313121   0.267101  -1.172  0.24108   
## as.factor(s_interest)7                  0.068689   0.284515   0.241  0.80923   
## as.factor(s_interest)8                 -0.532610   0.274367  -1.941  0.05223 . 
## ---
## Signif. codes:  0 '***' 0.001 '**' 0.01 '*' 0.05 '.' 0.1 ' ' 1
## 
## Threshold coefficients:
##       Estimate Std. Error z value
## -6|-4 -6.58513    1.06629  -6.176
## -4|-3 -3.77559    0.44636  -8.459
## -3|-2 -3.26554    0.41875  -7.798
## -2|-1 -1.77227    0.38316  -4.625
## -1|0  -1.32142    0.37890  -3.488
## 0|1   -0.05228    0.37387  -0.140
## 1|2    0.55506    0.37512   1.480
## 2|3    1.86651    0.38929   4.795
## 3|4    2.13559    0.39532   5.402
## 4|5    3.63270    0.48029   7.564
## 5|6    4.33950    0.57506   7.546
## (21 Beobachtungen als fehlend gelöscht)
```

```
exp(coef(diff_model8_pass1))
```

```
##                                  -6|-4                                  -4|-3 
##                            0.001380745                            0.022923453 
##                                  -3|-2                                  -2|-1 
##                            0.038176286                            0.169946912 
##                                   -1|0                                    0|1 
##                            0.266755290                            0.949067024 
##                                    1|2                                    2|3 
##                            1.742043392                            6.465663507 
##                                    3|4                                    4|5 
##                            8.462045053                           37.814938028 
##                                    5|6 H3_interactiondisclaimer.new guideline 
##                           76.669164452                            0.678281411 
##                      text_orderFaerber                                  s_age 
##                            1.626839914                            0.993846724 
##                              s_sexmale                           s_schoolReal 
##                            0.707653180                            1.220124945 
##                            s_schoolAbi                 as.factor(s_interest)5 
##                            1.715370987                            0.909944074 
##                 as.factor(s_interest)6                 as.factor(s_interest)7 
##                            0.731161557                            1.071103294 
##                 as.factor(s_interest)8 
##                            0.587070492
```

```
exp(confint((diff_model8_pass1)))
```

```
##                                            2.5 %    97.5 %
## H3_interactiondisclaimer.new guideline 0.4911004 0.9354306
## text_orderFaerber                      1.1778298 2.2510215
## s_age                                  0.9836779 1.0041017
## s_sexmale                              0.5088386 0.9829124
## s_schoolReal                           0.8067329 1.8458995
## s_schoolAbi                            1.1517100 2.5605504
## as.factor(s_interest)5                 0.5460993 1.5171487
## as.factor(s_interest)6                 0.4328520 1.2341965
## as.factor(s_interest)7                 0.6130843 1.8717818
## as.factor(s_interest)8                 0.3422971 1.0043133
```

```
nagelkerke(fit = diff_model8_pass1, null = diff_null_pass1)
```

```
## $Models
##                                                                                                                                              
## Model: "clm, as.factor(s_diff) ~ H3_interaction + text_order + s_age + s_sex + s_school + as.factor(s_interest), data2_wide_pass_reg5, logit"
## Null:  "clm, as.factor(s_diff) ~ 1, data2_wide_pass_reg5, logit"                                                                             
## 
## $Pseudo.R.squared.for.model.vs.null
##                              Pseudo.R.squared
## McFadden                            0.0168509
## Cox and Snell (ML)                  0.0648234
## Nagelkerke (Cragg and Uhler)        0.0660612
## 
## $Likelihood.ratio.test
##  Df.diff LogLik.diff  Chisq    p.value
##      -10     -15.984 31.968 0.00040531
## 
## $Number.of.observations
##           
## Model: 477
## Null:  477
## 
## $Messages
## [1] "Note: For models fit with REML, these statistics are based on refitting with ML"
## 
## $Warnings
## [1] "None"
```

```
H3test_pass1 = emmeans(diff_model8_pass1, ~ H3_interaction)
pairs(H3test_pass1, adjust = "tukey")
```

```
##  contrast                                               estimate    SE  df
##  no disclaimer.old guideline - disclaimer.new guideline    0.388 0.164 Inf
##  z.ratio p.value
##    2.363  0.0181
## 
## Results are averaged over the levels of: text_order, s_sex, s_school, s_interest 
## Note: contrasts are still on the as.factor scale
```

```
cld(H3test_pass1, Letters = letters)
```

```
##  H3_interaction              emmean    SE  df asymp.LCL asymp.UCL .group
##  disclaimer.new guideline    -0.153 0.170 Inf    -0.486     0.179  a    
##  no disclaimer.old guideline  0.235 0.169 Inf    -0.096     0.566   b   
## 
## Results are averaged over the levels of: text_order, s_sex, s_school, s_interest 
## Results are given on the as.factor (not the response) scale. 
## Confidence level used: 0.95 
## Note: contrasts are still on the as.factor scale 
## significance level used: alpha = 0.05 
## NOTE: If two or more means share the same grouping symbol,
##       then we cannot show them to be different.
##       But we also did not show them to be the same.
```

```
data2_wide_pass_reg6 <- subset(data2_wide_pass, condition == 4 | condition == 6)
View(data2_wide_pass_reg6)

diff_null_pass2 <- clm(as.factor(s_diff) ~ 1, data = data2_wide_pass_reg6, link = "logit")

diff_model8_pass2 <- clm(as.factor(s_diff) ~ H3_interaction + text_order + s_age + s_sex +
                       s_school + as.factor(s_interest), data = data2_wide_pass_reg6, 
                     link = "logit")

summary(diff_model8_pass2)
```

```
## formula: 
## as.factor(s_diff) ~ H3_interaction + text_order + s_age + s_sex + s_school + as.factor(s_interest)
## data:    data2_wide_pass_reg6
## 
##  link  threshold nobs logLik  AIC     niter max.grad cond.H 
##  logit flexible  439  -863.63 1769.26 6(0)  2.79e-07 7.4e+05
## 
## Coefficients:
##                                         Estimate Std. Error z value Pr(>|z|)
## H3_interactiondisclaimer.new guideline  0.040609   0.171091   0.237  0.81238
## text_orderFaerber                       0.688554   0.173445   3.970 7.19e-05
## s_age                                  -0.005643   0.005587  -1.010  0.31245
## s_sexmale                              -0.186012   0.172343  -1.079  0.28045
## s_schoolReal                            0.084964   0.224419   0.379  0.70499
## s_schoolAbi                             0.250525   0.211117   1.187  0.23536
## as.factor(s_interest)5                 -0.081958   0.273645  -0.300  0.76456
## as.factor(s_interest)6                 -0.275165   0.284954  -0.966  0.33422
## as.factor(s_interest)7                 -0.352366   0.289682  -1.216  0.22384
## as.factor(s_interest)8                 -0.768699   0.290873  -2.643  0.00822
##                                           
## H3_interactiondisclaimer.new guideline    
## text_orderFaerber                      ***
## s_age                                     
## s_sexmale                                 
## s_schoolReal                              
## s_schoolAbi                               
## as.factor(s_interest)5                    
## as.factor(s_interest)6                    
## as.factor(s_interest)7                    
## as.factor(s_interest)8                 ** 
## ---
## Signif. codes:  0 '***' 0.001 '**' 0.01 '*' 0.05 '.' 0.1 ' ' 1
## 
## Threshold coefficients:
##       Estimate Std. Error z value
## -6|-4 -5.62529    0.81663  -6.888
## -4|-3 -3.89968    0.50775  -7.680
## -3|-2 -3.33395    0.46892  -7.110
## -2|-1 -1.86714    0.42576  -4.385
## -1|0  -1.35600    0.41965  -3.231
## 0|1   -0.09764    0.41315  -0.236
## 1|2    0.47915    0.41409   1.157
## 2|3    1.84211    0.42647   4.319
## 3|4    2.04630    0.43012   4.757
## 4|5    3.28624    0.47948   6.854
## 5|6    3.81460    0.52408   7.279
## (14 Beobachtungen als fehlend gelöscht)
```

```
exp(coef(diff_model8_pass2))
```

```
##                                  -6|-4                                  -4|-3 
##                            0.003605502                            0.020248427 
##                                  -3|-2                                  -2|-1 
##                            0.035652068                            0.154564650 
##                                   -1|0                                    0|1 
##                            0.257689089                            0.906974807 
##                                    1|2                                    2|3 
##                            1.614697910                            6.309817279 
##                                    3|4                                    4|5 
##                            7.739192246                           26.742070059 
##                                    5|6 H3_interactiondisclaimer.new guideline 
##                           45.358523588                            1.041445037 
##                      text_orderFaerber                                  s_age 
##                            1.990835127                            0.994372655 
##                              s_sexmale                           s_schoolReal 
##                            0.830263764                            1.088678216 
##                            s_schoolAbi                 as.factor(s_interest)5 
##                            1.284699639                            0.921310933 
##                 as.factor(s_interest)6                 as.factor(s_interest)7 
##                            0.759446499                            0.703022865 
##                 as.factor(s_interest)8 
##                            0.463616005
```

```
exp(confint((diff_model8_pass2)))
```

```
##                                            2.5 %    97.5 %
## H3_interactiondisclaimer.new guideline 0.7446871 1.4567482
## text_orderFaerber                      1.4188033 2.8012153
## s_age                                  0.9835143 1.0053083
## s_sexmale                              0.5919237 1.1636173
## s_schoolReal                           0.7011513 1.6908710
## s_schoolAbi                            0.8496957 1.9449402
## as.factor(s_interest)5                 0.5387405 1.5762248
## as.factor(s_interest)6                 0.4339542 1.3272051
## as.factor(s_interest)7                 0.3980580 1.2402486
## as.factor(s_interest)8                 0.2616050 0.8189529
```

```
nagelkerke(fit = diff_model8_pass2, null = diff_null_pass1)
```

```
## $Models
##                                                                                                                                              
## Model: "clm, as.factor(s_diff) ~ H3_interaction + text_order + s_age + s_sex + s_school + as.factor(s_interest), data2_wide_pass_reg6, logit"
## Null:  "clm, as.factor(s_diff) ~ 1, data2_wide_pass_reg5, logit"                                                                             
## 
## $Pseudo.R.squared.for.model.vs.null
##                              Pseudo.R.squared
## McFadden                            0.0895446
## Cox and Snell (ML)                  0.3208860
## Nagelkerke (Cragg and Uhler)        0.3252050
## 
## $Likelihood.ratio.test
##  Df.diff LogLik.diff  Chisq    p.value
##      -10     -84.939 169.88 2.9397e-31
## 
## $Number.of.observations
##           
## Model: 439
## Null:  477
## 
## $Messages
## [1] "Note: For models fit with REML, these statistics are based on refitting with ML"
## 
## $Warnings
## [1] "WARNING: Fitted and null models have different numbers of observations"
```

```
H3test_pass2 = emmeans(diff_model8_pass2, ~ H3_interaction)
pairs(H3test_pass2, adjust = "tukey")
```

```
##  contrast                                               estimate    SE  df
##  no disclaimer.old guideline - disclaimer.new guideline  -0.0406 0.171 Inf
##  z.ratio p.value
##   -0.237  0.8124
## 
## Results are averaged over the levels of: text_order, s_sex, s_school, s_interest 
## Note: contrasts are still on the as.factor scale
```

```
cld(H3test_pass2, Letters = letters)
```

```
##  H3_interaction              emmean    SE  df asymp.LCL asymp.UCL .group
##  no disclaimer.old guideline  0.225 0.154 Inf   -0.0770     0.528  a    
##  disclaimer.new guideline     0.266 0.163 Inf   -0.0542     0.586  a    
## 
## Results are averaged over the levels of: text_order, s_sex, s_school, s_interest 
## Results are given on the as.factor (not the response) scale. 
## Confidence level used: 0.95 
## Note: contrasts are still on the as.factor scale 
## significance level used: alpha = 0.05 
## NOTE: If two or more means share the same grouping symbol,
##       then we cannot show them to be different.
##       But we also did not show them to be the same.
```

### H3 Graphical

```
describeBy(data2_wide_pass_reg$s_diff,
           data2_wide_pass_reg$H3_interaction)
```

```
## 
##  Descriptive statistics by group 
## group: no disclaimer.old guideline
##    vars   n mean sd median trimmed  mad min max range  skew kurtosis   se
## X1    1 239 0.58  2      0    0.56 1.48  -6   6    12 -0.07     0.24 0.13
## ------------------------------------------------------------ 
## group: no disclaimer.new guideline
##    vars   n mean   sd median trimmed  mad min max range skew kurtosis  se
## X1    1 432 0.39 2.11      0    0.34 2.22  -6   6    12 0.07      0.3 0.1
## ------------------------------------------------------------ 
## group: disclaimer.new guideline
##    vars   n mean  sd median trimmed  mad min max range skew kurtosis  se
## X1    1 438 0.43 2.1      0    0.32 2.97  -6   6    12 0.31     0.21 0.1
```

```
H3_bar <- ggplot(data2_wide_pass_reg, aes(H3_interaction, s_diff)) +
  stat_summary(fun = mean, geom = "bar", fill = "White",
               colour = "Black") + stat_summary(fun.data = 
                                                  mean_cl_normal,
                                                geom = "pointrange") +
  labs(x = "Condition", y = "Differentiation Knowledge Score")
H3_bar
```

```
## Warning: Removed 36 rows containing non-finite values (`stat_summary()`).
## Removed 36 rows containing non-finite values (`stat_summary()`).
```

```
data2_wide_pass_reg$H3_interaction <- mapvalues(data2_wide_pass_reg$H3_interaction,
                                                c("no disclaimer.old guideline",
                                                  "no disclaimer.new guideline",
                                                  "disclaimer.new guideline"),
                                                c("old, no disclaimer",
                                                  "new, no disclaimer",
                                                  "new, disclaimer"))

H3_boxplot <- ggplot(data2_wide_pass_reg, aes(H3_interaction, s_diff,
                                              fill = H3_interaction))
H3_boxplot <- H3_boxplot + geom_boxplot() + theme_classic() + theme(
  legend.position = "none",
  panel.grid.major = element_blank(),
  panel.grid.minor = element_blank(),
  panel.background = element_blank(),
  axis.title = element_text(face = "bold"),
  axis.text = element_text(face = "bold"),
  legend.title = element_text(face = "bold"))+
  labs(x = "Condition", y = "Differentiation Knowledge Score") +
  scale_fill_brewer(palette = "Blues")
H3_boxplot
```

```
## Warning: Removed 36 rows containing non-finite values (`stat_boxplot()`).
```

```
by(data2_wide_pass_reg$s_diff, data2_wide_pass_reg$H3_interaction, describe)
```

```
## data2_wide_pass_reg$H3_interaction: old, no disclaimer
##    vars   n mean sd median trimmed  mad min max range  skew kurtosis   se
## X1    1 239 0.58  2      0    0.56 1.48  -6   6    12 -0.07     0.24 0.13
## ------------------------------------------------------------ 
## data2_wide_pass_reg$H3_interaction: new, no disclaimer
##    vars   n mean   sd median trimmed  mad min max range skew kurtosis  se
## X1    1 432 0.39 2.11      0    0.34 2.22  -6   6    12 0.07      0.3 0.1
## ------------------------------------------------------------ 
## data2_wide_pass_reg$H3_interaction: new, disclaimer
##    vars   n mean  sd median trimmed  mad min max range skew kurtosis  se
## X1    1 438 0.43 2.1      0    0.32 2.97  -6   6    12 0.31     0.21 0.1
```

```
data2_wide_pass_reg_old <- subset(data2_wide_pass_reg, H3_interaction == 
                                    "old, no disclaimer")
quantile(data2_wide_pass_reg_old$s_diff, c(0.25, 0.75), na.rm = TRUE)
```

```
## 25% 75% 
##   0   2
```

## H4

### H4a

```
H4a_pass <- subset(data2_wide_pass, condition == 3| condition == 4| condition == 6) 
View(H4a_pass)

describeBy(H4a_pass$s_causality, H4a_pass$causality)
```

```
## 
##  Descriptive statistics by group 
## group: no causality statement
##    vars   n mean   sd median trimmed  mad min max range skew kurtosis   se
## X1    1 239    0 3.85      0   -0.06 2.97  -8  10    18 0.15    -0.27 0.25
## ------------------------------------------------------------ 
## group: causality statement
##    vars   n mean   sd median trimmed  mad min max range skew kurtosis  se
## X1    1 405 0.11 3.95      0    0.08 2.97  -9  12    21 0.11     -0.2 0.2
```

```
wilcox.test(s_causality~causality, data = H4a_pass, exact = FALSE,
            confint = TRUE)
```

```
## 
##  Wilcoxon rank sum test with continuity correction
## 
## data:  s_causality by causality
## W = 47508, p-value = 0.6952
## alternative hypothesis: true location shift is not equal to 0
```

```
# T-Test was computed to double-check results. Careful: 
# Requirements are not met.
t.test(s_causality~causality, data = H4a_pass, confint = TRUE)
```

```
## 
##  Welch Two Sample t-test
## 
## data:  s_causality by causality
## t = -0.32162, df = 508.95, p-value = 0.7479
## alternative hypothesis: true difference in means between group no causality statement and group causality statement is not equal to 0
## 95 percent confidence interval:
##  -0.7249870  0.5210096
## sample estimates:
## mean in group no causality statement    mean in group causality statement 
##                            0.0041841                            0.1061728
```

#### H4a post hoc

```
H4a_pass1 <- subset(data2_wide_pass, condition == 3| condition == 6) 
View(H4a_pass1)
describeBy(H4a_pass1$s_causality, H4a_pass1$causality)
```

```
## 
##  Descriptive statistics by group 
## group: no causality statement
##    vars   n mean   sd median trimmed  mad min max range skew kurtosis   se
## X1    1 239    0 3.85      0   -0.06 2.97  -8  10    18 0.15    -0.27 0.25
## ------------------------------------------------------------ 
## group: causality statement
##    vars   n mean   sd median trimmed  mad min max range skew kurtosis   se
## X1    1 209 0.08 3.92      0    0.05 2.97  -8  10    18 0.11    -0.23 0.27
```

```
wilcox.test(s_causality~causality, data = H4a_pass1, exact = FALSE,
            confint = TRUE)
```

```
## 
##  Wilcoxon rank sum test with continuity correction
## 
## data:  s_causality by causality
## W = 24716, p-value = 0.8491
## alternative hypothesis: true location shift is not equal to 0
```

```
H4a_pass2 <- subset(data2_wide_pass, condition == 4| condition == 6) 
View(H4a_pass2)
describeBy(H4a_pass2$s_causality, H4a_pass2$causality)
```

```
## 
##  Descriptive statistics by group 
## group: no causality statement
##    vars   n mean   sd median trimmed  mad min max range skew kurtosis   se
## X1    1 239    0 3.85      0   -0.06 2.97  -8  10    18 0.15    -0.27 0.25
## ------------------------------------------------------------ 
## group: causality statement
##    vars   n mean   sd median trimmed  mad min max range skew kurtosis   se
## X1    1 196 0.14 3.98      0    0.11 2.97  -9  12    21  0.1     -0.2 0.28
```

```
wilcox.test(s_causality~causality, data = H4a_pass2, exact = FALSE,
            confint = TRUE)
```

```
## 
##  Wilcoxon rank sum test with continuity correction
## 
## data:  s_causality by causality
## W = 22792, p-value = 0.6275
## alternative hypothesis: true location shift is not equal to 0
```

### H4b

```
H4b_pass <- subset(data2_wide_pass, condition == 1| condition == 2|
                condition == 3| condition == 4)
View(H4b_pass)

describeBy(H4b_pass$s_causality, H4b_pass$causality)
```

```
## 
##  Descriptive statistics by group 
## group: no causality statement
##    vars   n  mean   sd median trimmed  mad min max range skew kurtosis   se
## X1    1 457 -0.51 3.88      0   -0.57 2.97 -10  10    20 0.13    -0.24 0.18
## ------------------------------------------------------------ 
## group: causality statement
##    vars   n mean   sd median trimmed  mad min max range skew kurtosis  se
## X1    1 405 0.11 3.95      0    0.08 2.97  -9  12    21 0.11     -0.2 0.2
```

```
wilcox.test(s_causality~causality, data = H4b_pass, exact = FALSE,
            confint = TRUE)
```

```
## 
##  Wilcoxon rank sum test with continuity correction
## 
## data:  s_causality by causality
## W = 84435, p-value = 0.02563
## alternative hypothesis: true location shift is not equal to 0
```

```
# T-Test was computed to double-check results. Careful: 
# Requirements are not met.
t.test(s_causality~causality, data = H4b_pass, confint = TRUE)
```

```
## 
##  Welch Two Sample t-test
## 
## data:  s_causality by causality
## t = -2.3053, df = 844.03, p-value = 0.02139
## alternative hypothesis: true difference in means between group no causality statement and group causality statement is not equal to 0
## 95 percent confidence interval:
##  -1.14050223 -0.09153711
## sample estimates:
## mean in group no causality statement    mean in group causality statement 
##                           -0.5098468                            0.1061728
```

#### H4b post hoc

```
H4b_pass1 <- subset(data2_wide_pass, condition == 1|condition == 3)
View(H4b_pass1)
describeBy(H4b_pass1$s_causality, H4b_pass1$causality)
```

```
## 
##  Descriptive statistics by group 
## group: no causality statement
##    vars   n  mean   sd median trimmed  mad min max range skew kurtosis   se
## X1    1 215 -0.12 3.85      0   -0.19 4.45  -8  10    18 0.15    -0.39 0.26
## ------------------------------------------------------------ 
## group: causality statement
##    vars   n mean   sd median trimmed  mad min max range skew kurtosis   se
## X1    1 209 0.08 3.92      0    0.05 2.97  -8  10    18 0.11    -0.23 0.27
```

```
wilcox.test(s_causality~causality, data = H4b_pass1, exact = FALSE,
            confint = TRUE)
```

```
## 
##  Wilcoxon rank sum test with continuity correction
## 
## data:  s_causality by causality
## W = 21834, p-value = 0.6144
## alternative hypothesis: true location shift is not equal to 0
```

```
H4b_pass2 <- subset(data2_wide_pass, condition == 2|condition == 4)
View(H4b_pass2)
describeBy(H4b_pass2$s_causality, H4b_pass2$causality)
```

```
## 
##  Descriptive statistics by group 
## group: no causality statement
##    vars   n  mean   sd median trimmed  mad min max range skew kurtosis   se
## X1    1 242 -0.86 3.88     -1   -0.91 4.45 -10  10    20 0.12    -0.16 0.25
## ------------------------------------------------------------ 
## group: causality statement
##    vars   n mean   sd median trimmed  mad min max range skew kurtosis   se
## X1    1 196 0.14 3.98      0    0.11 2.97  -9  12    21  0.1     -0.2 0.28
```

```
wilcox.test(s_causality~causality, data = H4b_pass2, exact = FALSE,
            confint = TRUE)
```

```
## 
##  Wilcoxon rank sum test with continuity correction
## 
## data:  s_causality by causality
## W = 20336, p-value = 0.009955
## alternative hypothesis: true location shift is not equal to 0
```

```
H4b_pass3 <- subset(data2_wide_pass, condition == 1|condition == 4)
View(H4b_pass3)
describeBy(H4b_pass3$s_causality, H4b_pass3$causality)
```

```
## 
##  Descriptive statistics by group 
## group: no causality statement
##    vars   n  mean   sd median trimmed  mad min max range skew kurtosis   se
## X1    1 215 -0.12 3.85      0   -0.19 4.45  -8  10    18 0.15    -0.39 0.26
## ------------------------------------------------------------ 
## group: causality statement
##    vars   n mean   sd median trimmed  mad min max range skew kurtosis   se
## X1    1 196 0.14 3.98      0    0.11 2.97  -9  12    21  0.1     -0.2 0.28
```

```
wilcox.test(s_causality~causality, data = H4b_pass3, exact = FALSE,
            confint = TRUE)
```

```
## 
##  Wilcoxon rank sum test with continuity correction
## 
## data:  s_causality by causality
## W = 20235, p-value = 0.4859
## alternative hypothesis: true location shift is not equal to 0
```

```
H4b_pass4 <- subset(data2_wide_pass, condition == 2|condition == 3)
View(H4b_pass4)
describeBy(H4b_pass4$s_causality, H4b_pass4$causality)
```

```
## 
##  Descriptive statistics by group 
## group: no causality statement
##    vars   n  mean   sd median trimmed  mad min max range skew kurtosis   se
## X1    1 242 -0.86 3.88     -1   -0.91 4.45 -10  10    20 0.12    -0.16 0.25
## ------------------------------------------------------------ 
## group: causality statement
##    vars   n mean   sd median trimmed  mad min max range skew kurtosis   se
## X1    1 209 0.08 3.92      0    0.05 2.97  -8  10    18 0.11    -0.23 0.27
```

```
wilcox.test(s_causality~causality, data = H4b_pass4, exact = FALSE,
            confint = TRUE)
```

```
## 
##  Wilcoxon rank sum test with continuity correction
## 
## data:  s_causality by causality
## W = 22030, p-value = 0.01772
## alternative hypothesis: true location shift is not equal to 0
```

### H4 Mixed Model

```
sum(is.na(data2_long_pass$disclaimer))
```

```
## [1] 0
```

```
sum(is.na(data2_long_pass$s_awareness))
```

```
## [1] 0
```

```
sum(is.na(data2_long_pass$text_order))
```

```
## [1] 0
```

```
sum(is.na(data2_long_pass$s_age))
```

```
## [1] 2
```

```
data2_long_pass <- data2_long_pass %>% drop_na(s_age)
sum(is.na(data2_long_pass$s_sex))
```

```
## [1] 0
```

```
sum(is.na(data2_long_pass$s_school))
```

```
## [1] 0
```

```
sum(is.na(data2_long_pass$s_interest))
```

```
## [1] 0
```

```
data2_long_pass$id <- as.numeric(data2_long_pass$id)

data2_long_pass$H4_interaction <- interaction(data2_long_pass$causality, 
                                         data2_long_pass$version)
data2_long_pass$H4_interaction <- droplevels(data2_long_pass$H4_interaction)
table(data2_long_pass$H4_interaction)
```

```
## 
## no causality statement.old guideline no causality statement.new guideline 
##                                  494                                  944 
##    causality statement.new guideline 
##                                 1326
```

```
data2_long_pass_reg <- subset(data2_long_pass, condition != 5)
View(data2_long_pass_reg)

data2_long_pass_reg$H4_interaction <- relevel(data2_long_pass_reg$H4_interaction,
                                              ref = "no causality statement.old guideline")

set.seed(288659)

causality_null_pass <- clm(as.factor(s_causality) ~ 1,
                           data = data2_long_pass_reg,
                      link = "logit")

causality_model1_pass <- clmm(as.factor(s_causality) ~ 1 + (1|id),
                         data = data2_long_pass_reg)
```

```
## Warning in update.uC(rho): Non finite negative log-likelihood
##   at iteration 75
```

```
anova(causality_null_pass,causality_model1_pass)
```

```
## Likelihood ratio tests of cumulative link models:
##  
##                       formula:                              link: threshold:
## causality_null_pass   as.factor(s_causality) ~ 1            logit flexible  
## causality_model1_pass as.factor(s_causality) ~ 1 + (1 | id) logit flexible  
## 
##                       no.par    AIC  logLik LR.stat df Pr(>Chisq)    
## causality_null_pass       12 9757.2 -4866.6                          
## causality_model1_pass     13 9727.4 -4850.7  31.761  1  1.744e-08 ***
## ---
## Signif. codes:  0 '***' 0.001 '**' 0.01 '*' 0.05 '.' 0.1 ' ' 1
```

```
causality_model2_pass <- clmm(as.factor(s_causality) ~ H4_interaction + (1|id),
                         data = data2_long_pass_reg)
```

```
## Warning in update.uC(rho): Non finite negative log-likelihood
##   at iteration 85
```

```
anova(causality_model1_pass,causality_model2_pass)
```

```
## Likelihood ratio tests of cumulative link models:
##  
##                       formula:                                           link:
## causality_model1_pass as.factor(s_causality) ~ 1 + (1 | id)              logit
## causality_model2_pass as.factor(s_causality) ~ H4_interaction + (1 | id) logit
##                       threshold:
## causality_model1_pass flexible  
## causality_model2_pass flexible  
## 
##                       no.par    AIC  logLik LR.stat df Pr(>Chisq)  
## causality_model1_pass     13 9727.4 -4850.7                        
## causality_model2_pass     15 9725.0 -4847.5  6.3853  2    0.04106 *
## ---
## Signif. codes:  0 '***' 0.001 '**' 0.01 '*' 0.05 '.' 0.1 ' ' 1
```

```
causality_model3_pass <- clmm(as.factor(s_causality) ~ H4_interaction +
                           summary + (1|id), data = data2_long_pass_reg)
```

```
## Warning in update.uC(rho): Non finite negative log-likelihood
##   at iteration 90
```

```
anova(causality_model2_pass,causality_model3_pass)
```

```
## Likelihood ratio tests of cumulative link models:
##  
##                       formula:                                                    
## causality_model2_pass as.factor(s_causality) ~ H4_interaction + (1 | id)          
## causality_model3_pass as.factor(s_causality) ~ H4_interaction + summary + (1 | id)
##                       link: threshold:
## causality_model2_pass logit flexible  
## causality_model3_pass logit flexible  
## 
##                       no.par    AIC  logLik LR.stat df Pr(>Chisq)  
## causality_model2_pass     15 9725.0 -4847.5                        
## causality_model3_pass     16 9723.8 -4845.9  3.2427  1    0.07174 .
## ---
## Signif. codes:  0 '***' 0.001 '**' 0.01 '*' 0.05 '.' 0.1 ' ' 1
```

```
causality_model4_pass <- clmm(as.factor(s_causality) ~ H4_interaction + 
                                summary + text_order + (1|id), 
                              data = data2_long_pass_reg)
```

```
## Warning in update.uC(rho): Non finite negative log-likelihood
##   at iteration 95
```

```
anova(causality_model2_pass,causality_model4_pass)
```

```
## Likelihood ratio tests of cumulative link models:
##  
##                       formula:                                                                 
## causality_model2_pass as.factor(s_causality) ~ H4_interaction + (1 | id)                       
## causality_model4_pass as.factor(s_causality) ~ H4_interaction + summary + text_order + (1 | id)
##                       link: threshold:
## causality_model2_pass logit flexible  
## causality_model4_pass logit flexible  
## 
##                       no.par    AIC  logLik LR.stat df Pr(>Chisq)  
## causality_model2_pass     15 9725.0 -4847.5                        
## causality_model4_pass     17 9722.2 -4844.1   6.866  2    0.03229 *
## ---
## Signif. codes:  0 '***' 0.001 '**' 0.01 '*' 0.05 '.' 0.1 ' ' 1
```

```
causality_model5_pass <- clmm(as.factor(s_causality) ~ H4_interaction +
                           summary + text_order + s_age + (1|id),
                         data = data2_long_pass_reg)
anova(causality_model2_pass,causality_model5_pass)
```

```
## Likelihood ratio tests of cumulative link models:
##  
##                       formula:                                                                         
## causality_model2_pass as.factor(s_causality) ~ H4_interaction + (1 | id)                               
## causality_model5_pass as.factor(s_causality) ~ H4_interaction + summary + text_order + s_age + (1 | id)
##                       link: threshold:
## causality_model2_pass logit flexible  
## causality_model5_pass logit flexible  
## 
##                       no.par    AIC  logLik LR.stat df Pr(>Chisq)    
## causality_model2_pass     15 9725.0 -4847.5                          
## causality_model5_pass     18 9681.1 -4822.6  49.889  3  8.434e-11 ***
## ---
## Signif. codes:  0 '***' 0.001 '**' 0.01 '*' 0.05 '.' 0.1 ' ' 1
```

```
causality_model6_pass <- clmm(as.factor(s_causality) ~ H4_interaction + 
                                summary + text_order + s_age + s_sex + (1|id),
                         data = data2_long_pass_reg)
anova(causality_model5_pass,causality_model6_pass)
```

```
## Likelihood ratio tests of cumulative link models:
##  
##                       formula:                                                                                 
## causality_model5_pass as.factor(s_causality) ~ H4_interaction + summary + text_order + s_age + (1 | id)        
## causality_model6_pass as.factor(s_causality) ~ H4_interaction + summary + text_order + s_age + s_sex + (1 | id)
##                       link: threshold:
## causality_model5_pass logit flexible  
## causality_model6_pass logit flexible  
## 
##                       no.par    AIC  logLik LR.stat df Pr(>Chisq)
## causality_model5_pass     18 9681.1 -4822.6                      
## causality_model6_pass     19 9681.5 -4821.8  1.6077  1     0.2048
```

```
# Note: The number of model iterations for causality_model7_pass & 
# causality_model_8_pass can deviate slightly from their counterparts in
# the original, longer version of the Markdown. 
# Therefore, analysis results can vary from the third and forth digit onward. 
# The overall significance patterns of effects remain unchanged.

causality_model7_pass <- clmm(as.factor(s_causality) ~ H4_interaction +
                           summary + text_order + s_age + s_sex + s_school +
                           (1|id), data = data2_long_pass_reg)
anova(causality_model5_pass,causality_model7_pass)
```

```
## Likelihood ratio tests of cumulative link models:
##  
##                       formula:                                                                                            
## causality_model5_pass as.factor(s_causality) ~ H4_interaction + summary + text_order + s_age + (1 | id)                   
## causality_model7_pass as.factor(s_causality) ~ H4_interaction + summary + text_order + s_age + s_sex + s_school + (1 | id)
##                       link: threshold:
## causality_model5_pass logit flexible  
## causality_model7_pass logit flexible  
## 
##                       no.par    AIC  logLik LR.stat df Pr(>Chisq)    
## causality_model5_pass     18 9681.1 -4822.6                          
## causality_model7_pass     21 9641.4 -4799.7  45.778  3  6.324e-10 ***
## ---
## Signif. codes:  0 '***' 0.001 '**' 0.01 '*' 0.05 '.' 0.1 ' ' 1
```

```
causality_model8_pass <- clmm(as.factor(s_causality) ~ H4_interaction +
                            summary + text_order + s_age + s_sex + s_school +
                            as.factor(s_interest) + (1|id), 
                            data = data2_long_pass_reg)
anova(causality_model7_pass,causality_model8_pass)
```

```
## Likelihood ratio tests of cumulative link models:
##  
##                       formula:                                                                                                                    
## causality_model7_pass as.factor(s_causality) ~ H4_interaction + summary + text_order + s_age + s_sex + s_school + (1 | id)                        
## causality_model8_pass as.factor(s_causality) ~ H4_interaction + summary + text_order + s_age + s_sex + s_school + as.factor(s_interest) + (1 | id)
##                       link: threshold:
## causality_model7_pass logit flexible  
## causality_model8_pass logit flexible  
## 
##                       no.par    AIC  logLik LR.stat df Pr(>Chisq)  
## causality_model7_pass     21 9641.4 -4799.7                        
## causality_model8_pass     25 9639.8 -4794.9  9.5524  4    0.04868 *
## ---
## Signif. codes:  0 '***' 0.001 '**' 0.01 '*' 0.05 '.' 0.1 ' ' 1
```

```
# Model parameters can vary compared to the original markdown due to deviations 
# in the number of model iterations

summary(causality_model8_pass)
```

```
## Cumulative Link Mixed Model fitted with the Laplace approximation
## 
## formula: as.factor(s_causality) ~ H4_interaction + summary + text_order +  
##     s_age + s_sex + s_school + as.factor(s_interest) + (1 | id)
## data:    data2_long_pass_reg
## 
##  link  threshold nobs logLik   AIC     niter       max.grad cond.H 
##  logit flexible  2241 -4794.91 9639.81 4258(12411) 1.39e+03 8.4e+06
## 
## Random effects:
##  Groups Name        Variance Std.Dev.
##  id     (Intercept) 0.496    0.7043  
## Number of groups:  id 1140 
## 
## Coefficients:
##                                                      Estimate Std. Error
## H4_interactionno causality statement.new guideline -0.1767424  0.0001998
## H4_interactioncausality statement.new guideline     0.1066658  0.0863799
## summaryFaerber                                     -0.1252846  0.0001896
## text_orderFaerber                                   0.1860169  0.0789123
## s_age                                              -0.0179132  0.0001886
## s_sexmale                                           0.0986956  0.0789299
## s_schoolReal                                        0.2083232  0.0903357
## s_schoolAbi                                         0.6916899  0.0001998
## as.factor(s_interest)5                              0.0778646  0.1070096
## as.factor(s_interest)6                             -0.0244182  0.1047462
## as.factor(s_interest)7                             -0.1531524  0.1169174
## as.factor(s_interest)8                             -0.2994093  0.0001998
##                                                      z value Pr(>|z|)    
## H4_interactionno causality statement.new guideline  -884.514   <2e-16 ***
## H4_interactioncausality statement.new guideline        1.235   0.2169    
## summaryFaerber                                      -660.898   <2e-16 ***
## text_orderFaerber                                      2.357   0.0184 *  
## s_age                                                -94.993   <2e-16 ***
## s_sexmale                                              1.250   0.2111    
## s_schoolReal                                           2.306   0.0211 *  
## s_schoolAbi                                         3461.587   <2e-16 ***
## as.factor(s_interest)5                                 0.728   0.4668    
## as.factor(s_interest)6                                -0.233   0.8157    
## as.factor(s_interest)7                                -1.310   0.1902    
## as.factor(s_interest)8                             -1498.405   <2e-16 ***
## ---
## Signif. codes:  0 '***' 0.001 '**' 0.01 '*' 0.05 '.' 0.1 ' ' 1
## 
## Threshold coefficients:
##         Estimate Std. Error    z value
## -6|-5 -6.8001518  0.4056923    -16.762
## -5|-4 -5.9451852  0.2624667    -22.651
## -4|-3 -2.6763651  0.0001896 -14118.386
## -3|-2 -2.2462298  0.0001896 -11849.355
## -2|-1 -1.3935607  0.0435217    -32.020
## -1|0  -0.9610444  0.0493534    -19.473
## 0|1    0.1046717  0.0595704      1.757
## 1|2    0.4721950  0.0632637      7.464
## 2|3    1.4655003  0.0768068     19.080
## 3|4    1.7967658  0.0832537     21.582
## 4|5    3.4448469  0.1444983     23.840
## 5|6    3.7689982  0.1654350     22.782
## (49 Beobachtungen als fehlend gelöscht)
```

```
exp(coef(causality_model8_pass))
```

```
##                                              -6|-5 
##                                        0.001113606 
##                                              -5|-4 
##                                        0.002618417 
##                                              -4|-3 
##                                        0.068812828 
##                                              -3|-2 
##                                        0.105797349 
##                                              -2|-1 
##                                        0.248189992 
##                                               -1|0 
##                                        0.382493205 
##                                                0|1 
##                                        1.110345985 
##                                                1|2 
##                                        1.603510042 
##                                                2|3 
##                                        4.329708860 
##                                                3|4 
##                                        6.030113219 
##                                                4|5 
##                                       31.338486170 
##                                                5|6 
##                                       43.336628924 
## H4_interactionno causality statement.new guideline 
##                                        0.837995655 
##    H4_interactioncausality statement.new guideline 
##                                        1.112562377 
##                                     summaryFaerber 
##                                        0.882245790 
##                                  text_orderFaerber 
##                                        1.204442603 
##                                              s_age 
##                                        0.982246284 
##                                          s_sexmale 
##                                        1.103730219 
##                                       s_schoolReal 
##                                        1.231611114 
##                                        s_schoolAbi 
##                                        1.997087626 
##                             as.factor(s_interest)5 
##                                        1.080976261 
##                             as.factor(s_interest)6 
##                                        0.975877487 
##                             as.factor(s_interest)7 
##                                        0.857998979 
##                             as.factor(s_interest)8 
##                                        0.741255974
```

```
exp(confint(causality_model8_pass))
```

```
##                                                           2.5 %       97.5 %
## -6|-5                                               0.000502812  0.002466366
## -5|-4                                               0.001565405  0.004379767
## -4|-3                                               0.068787266  0.068838399
## -3|-2                                               0.105758048  0.105836664
## -2|-1                                               0.227896956  0.270290018
## -1|0                                                0.347227429  0.421340711
## 0|1                                                 0.987988610  1.247856699
## 1|2                                                 1.416516121  1.815188981
## 2|3                                                 3.724608720  5.033113604
## 3|4                                                 5.122238855  7.098900786
## 4|5                                                23.609177459 41.598260555
## 5|6                                                31.335499538 59.934050332
## H4_interactionno causality statement.new guideline  0.837667529  0.838323909
## H4_interactioncausality statement.new guideline     0.939285546  1.317804843
## summaryFaerber                                      0.881918057  0.882573645
## text_orderFaerber                                   1.031848331  1.405906218
## s_age                                               0.981883314  0.982609389
## s_sexmale                                           0.945535172  1.288392470
## s_schoolReal                                        1.031762453  1.470169738
## s_schoolAbi                                         1.996305645  1.997869914
## as.factor(s_interest)5                              0.876454587  1.333223300
## as.factor(s_interest)6                              0.794758457  1.198272080
## as.factor(s_interest)7                              0.682286093  1.078964169
## as.factor(s_interest)8                              0.740965727  0.741546334
```

```
nagelkerke(fit = causality_model8_pass, null = causality_null_pass)
```

```
## $Models
##                                                                                                                                                                 
## Model: "clmm, as.factor(s_causality) ~ H4_interaction + summary + text_order + s_age + s_sex + s_school + as.factor(s_interest) + (1 | id), data2_long_pass_reg"
## Null:  "clm, as.factor(s_causality) ~ 1, data2_long_pass_reg, logit"                                                                                            
## 
## $Pseudo.R.squared.for.model.vs.null
##                              Pseudo.R.squared
## McFadden                            0.0147296
## Cox and Snell (ML)                  0.0619706
## Nagelkerke (Cragg and Uhler)        0.0627865
## 
## $Likelihood.ratio.test
##  Df.diff LogLik.diff  Chisq    p.value
##      -13     -71.683 143.37 4.4487e-24
## 
## $Number.of.observations
##            
## Model: 2241
## Null:  2241
## 
## $Messages
## [1] "Note: For models fit with REML, these statistics are based on refitting with ML"
## 
## $Warnings
## [1] "None"
```

```
H4test_pass = emmeans(causality_model8_pass, ~ H4_interaction)
pairs(H4test_pass, adjust = "tukey")
```

```
##  contrast                                                                   
##  no causality statement.old guideline - no causality statement.new guideline
##  no causality statement.old guideline - causality statement.new guideline   
##  no causality statement.new guideline - causality statement.new guideline   
##  estimate        SE  df z.ratio p.value
##    0.1767 0.0001998 Inf 884.514  <.0001
##   -0.1067 0.0863799 Inf  -1.235  0.4326
##   -0.2834 0.0863799 Inf  -3.281  0.0030
## 
## Results are averaged over the levels of: summary, text_order, s_sex, s_school, s_interest 
## Note: contrasts are still on the as.factor scale 
## P value adjustment: tukey method for comparing a family of 3 estimates
```

```
cld(H4test_pass, Letters = letters)
```

```
##  H4_interaction                       emmean     SE  df asymp.LCL asymp.UCL
##  no causality statement.new guideline 0.0254 0.0726 Inf   -0.1169     0.168
##  no causality statement.old guideline 0.2022 0.0726 Inf    0.0599     0.344
##  causality statement.new guideline    0.3088 0.0900 Inf    0.1324     0.485
##  .group
##   a    
##    b   
##    b   
## 
## Results are averaged over the levels of: summary, text_order, s_sex, s_school, s_interest 
## Results are given on the as.factor (not the response) scale. 
## Confidence level used: 0.95 
## Note: contrasts are still on the as.factor scale 
## P value adjustment: tukey method for comparing a family of 3 estimates 
## significance level used: alpha = 0.05 
## NOTE: If two or more means share the same grouping symbol,
##       then we cannot show them to be different.
##       But we also did not show them to be the same.
```

```
# Changing reference group for H4_interaction to new_no causality" 
# for further testing. Once again, deviations in model iterations can cause 
# slightly different results compared to the original Markdown. 

data2_long_pass_reg$H4_interaction <- relevel(data2_long_pass_reg$H4_interaction,
                                              ref = "no causality statement.new guideline")

set.seed(288659)

causality_null_pass <- clm(as.factor(s_causality) ~ 1,
                           data = data2_long_pass_reg,
                      link = "logit")

causality_model8_pass <- clmm(as.factor(s_causality) ~ H4_interaction +
                            summary + text_order + s_age + s_sex + s_school +
                            as.factor(s_interest) + (1|id), 
                            data = data2_long_pass_reg)

summary(causality_model8_pass)
```

```
## Cumulative Link Mixed Model fitted with the Laplace approximation
## 
## formula: as.factor(s_causality) ~ H4_interaction + summary + text_order +  
##     s_age + s_sex + s_school + as.factor(s_interest) + (1 | id)
## data:    data2_long_pass_reg
## 
##  link  threshold nobs logLik   AIC     niter       max.grad cond.H 
##  logit flexible  2241 -4794.89 9639.77 3858(11292) 1.39e+03 8.4e+06
## 
## Random effects:
##  Groups Name        Variance Std.Dev.
##  id     (Intercept) 0.4961   0.7044  
## Number of groups:  id 1140 
## 
## Coefficients:
##                                                      Estimate Std. Error
## H4_interactionno causality statement.old guideline  0.1784537  0.1078954
## H4_interactioncausality statement.new guideline     0.2788842  0.0913016
## summaryFaerber                                     -0.1259872  0.0001896
## text_orderFaerber                                   0.1734859  0.0796439
## s_age                                              -0.0177471  0.0001886
## s_sexmale                                           0.0883798  0.0795865
## s_schoolReal                                        0.2067943  0.0906230
## s_schoolAbi                                         0.6943997  0.0001998
## as.factor(s_interest)5                              0.0918665  0.1083699
## as.factor(s_interest)6                             -0.0173139  0.1055820
## as.factor(s_interest)7                             -0.1572691  0.1185124
## as.factor(s_interest)8                             -0.3082968  0.0001998
##                                                      z value Pr(>|z|)    
## H4_interactionno causality statement.old guideline     1.654  0.09814 .  
## H4_interactioncausality statement.new guideline        3.055  0.00225 ** 
## summaryFaerber                                      -664.607  < 2e-16 ***
## text_orderFaerber                                      2.178  0.02939 *  
## s_age                                                -94.078  < 2e-16 ***
## s_sexmale                                              1.110  0.26679    
## s_schoolReal                                           2.282  0.02249 *  
## s_schoolAbi                                         3475.160  < 2e-16 ***
## as.factor(s_interest)5                                 0.848  0.39660    
## as.factor(s_interest)6                                -0.164  0.86974    
## as.factor(s_interest)7                                -1.327  0.18450    
## as.factor(s_interest)8                             -1542.888  < 2e-16 ***
## ---
## Signif. codes:  0 '***' 0.001 '**' 0.01 '*' 0.05 '.' 0.1 ' ' 1
## 
## Threshold coefficients:
##         Estimate Std. Error    z value
## -6|-5 -6.6247530  0.4062325    -16.308
## -5|-4 -5.7735176  0.2649810    -21.788
## -4|-3 -2.5009338  0.0393879    -63.495
## -3|-2 -2.0710706  0.0001998 -10364.765
## -2|-1 -1.2171552  0.0438892    -27.732
## -1|0  -0.7854131  0.0498319    -15.761
## 0|1    0.2811497  0.0602391      4.667
## 1|2    0.6473577  0.0639129     10.129
## 2|3    1.6422687  0.0774550     21.203
## 3|4    1.9721289  0.0838210     23.528
## 4|5    3.6194489  0.1448116     24.994
## 5|6    3.9433681  0.1656867     23.800
## (49 Beobachtungen als fehlend gelöscht)
```

```
exp(coef(causality_model8_pass))
```

```
##                                              -6|-5 
##                                        0.001327108 
##                                              -5|-4 
##                                        0.003108803 
##                                              -4|-3 
##                                        0.082008382 
##                                              -3|-2 
##                                        0.126050756 
##                                              -2|-1 
##                                        0.296071234 
##                                               -1|0 
##                                        0.455931338 
##                                                0|1 
##                                        1.324651929 
##                                                1|2 
##                                        1.910486034 
##                                                2|3 
##                                        5.166878189 
##                                                3|4 
##                                        7.185958480 
##                                                4|5 
##                                       37.316996650 
##                                                5|6 
##                                       51.592073783 
## H4_interactionno causality statement.old guideline 
##                                        1.195367547 
##    H4_interactioncausality statement.new guideline 
##                                        1.321654242 
##                                     summaryFaerber 
##                                        0.881626133 
##                                  text_orderFaerber 
##                                        1.189443906 
##                                              s_age 
##                                        0.982409426 
##                                          s_sexmale 
##                                        1.092402891 
##                                       s_schoolReal 
##                                        1.229729564 
##                                        s_schoolAbi 
##                                        2.002506687 
##                             as.factor(s_interest)5 
##                                        1.096218473 
##                             as.factor(s_interest)6 
##                                        0.982835138 
##                             as.factor(s_interest)7 
##                                        0.854474073 
##                             as.factor(s_interest)8 
##                                        0.734697224
```

```
exp(confint(causality_model8_pass))
```

```
##                                                           2.5 %       97.5 %
## -6|-5                                              5.985778e-04  0.002942335
## -5|-4                                              1.849443e-03  0.005225711
## -4|-3                                              7.591563e-02  0.088590121
## -3|-2                                              1.260014e-01  0.126100132
## -2|-1                                              2.716675e-01  0.322667156
## -1|0                                               4.135066e-01  0.502708728
## 0|1                                                1.177135e+00  1.490655898
## 1|2                                                1.685548e+00  2.165442363
## 2|3                                                4.439135e+00  6.013926005
## 3|4                                                6.097280e+00  8.469022221
## 4|5                                                2.809589e+01 49.564478505
## 5|6                                                3.728638e+01 71.386451629
## H4_interactionno causality statement.old guideline 9.675218e-01  1.476869642
## H4_interactioncausality statement.new guideline    1.105101e+00  1.580643411
## summaryFaerber                                     8.812986e-01  0.881953756
## text_orderFaerber                                  1.017539e+00  1.390391110
## s_age                                              9.820463e-01  0.982772722
## s_sexmale                                          9.346279e-01  1.276811995
## s_schoolReal                                       1.029606e+00  1.468750587
## s_schoolAbi                                        2.001723e+00  2.003291095
## as.factor(s_interest)5                             8.864464e-01  1.355631781
## as.factor(s_interest)6                             7.991147e-01  1.208793809
## as.factor(s_interest)7                             6.773622e-01  1.077895867
## as.factor(s_interest)8                             7.344095e-01  0.734985014
```

```
nagelkerke(fit = causality_model8_pass, null = causality_null_pass)
```

```
## $Models
##                                                                                                                                                                 
## Model: "clmm, as.factor(s_causality) ~ H4_interaction + summary + text_order + s_age + s_sex + s_school + as.factor(s_interest) + (1 | id), data2_long_pass_reg"
## Null:  "clm, as.factor(s_causality) ~ 1, data2_long_pass_reg, logit"                                                                                            
## 
## $Pseudo.R.squared.for.model.vs.null
##                              Pseudo.R.squared
## McFadden                            0.0147338
## Cox and Snell (ML)                  0.0619877
## Nagelkerke (Cragg and Uhler)        0.0628038
## 
## $Likelihood.ratio.test
##  Df.diff LogLik.diff  Chisq    p.value
##      -13     -71.703 143.41 4.3654e-24
## 
## $Number.of.observations
##            
## Model: 2241
## Null:  2241
## 
## $Messages
## [1] "Note: For models fit with REML, these statistics are based on refitting with ML"
## 
## $Warnings
## [1] "None"
```

```
H4test_pass = emmeans(causality_model8_pass, ~ H4_interaction)
pairs(H4test_pass, adjust = "tukey")
```

```
##  contrast                                                                   
##  no causality statement.new guideline - no causality statement.old guideline
##  no causality statement.new guideline - causality statement.new guideline   
##  no causality statement.old guideline - causality statement.new guideline   
##  estimate     SE  df z.ratio p.value
##    -0.178 0.1079 Inf  -1.654  0.2232
##    -0.279 0.0913 Inf  -3.055  0.0064
##    -0.100 0.1166 Inf  -0.861  0.6648
## 
## Results are averaged over the levels of: summary, text_order, s_sex, s_school, s_interest 
## Note: contrasts are still on the as.factor scale 
## P value adjustment: tukey method for comparing a family of 3 estimates
```

```
cld(H4test_pass, Letters = letters)
```

```
##  H4_interaction                       emmean     SE  df asymp.LCL asymp.UCL
##  no causality statement.new guideline  0.025 0.0782 Inf  -0.12822     0.178
##  no causality statement.old guideline  0.203 0.1079 Inf  -0.00808     0.415
##  causality statement.new guideline     0.304 0.0902 Inf   0.12717     0.481
##  .group
##   a    
##   ab   
##    b   
## 
## Results are averaged over the levels of: summary, text_order, s_sex, s_school, s_interest 
## Results are given on the as.factor (not the response) scale. 
## Confidence level used: 0.95 
## Note: contrasts are still on the as.factor scale 
## P value adjustment: tukey method for comparing a family of 3 estimates 
## significance level used: alpha = 0.05 
## NOTE: If two or more means share the same grouping symbol,
##       then we cannot show them to be different.
##       But we also did not show them to be the same.
```

#### Mixed Model by Groups

```
# Once again, deviations in model iterations compared to the original Markdown
# can cause slightly different results.

set.seed(288659)

data2_long_pass_reg1 <- subset(data2_long_pass, condition == 3 | condition == 6)
View(data2_long_pass_reg1)

causality_null_pass1 <- clm(as.factor(s_causality) ~ 1, data = data2_long_pass_reg1, link = "logit")

causality_model10_pass1 <- clmm(as.factor(s_causality) ~ H4_interaction +
                           summary + text_order + s_age + s_sex + s_school +
                           as.factor(s_interest) + (1|id), data = data2_long_pass_reg1)

summary(causality_model10_pass1)
```

```
## Cumulative Link Mixed Model fitted with the Laplace approximation
## 
## formula: as.factor(s_causality) ~ H4_interaction + summary + text_order +  
##     s_age + s_sex + s_school + as.factor(s_interest) + (1 | id)
## data:    data2_long_pass_reg1
## 
##  link  threshold nobs logLik   AIC     niter      max.grad cond.H 
##  logit flexible  913  -1946.70 3941.41 2742(7893) 5.83e+02 1.0e+07
## 
## Random effects:
##  Groups Name        Variance Std.Dev.
##  id     (Intercept) 0.4968   0.7048  
## Number of groups:  id 465 
## 
## Coefficients:
##                                                   Estimate Std. Error z value
## H4_interactioncausality statement.new guideline  0.1030131  0.0003087 333.680
## summaryFaerber                                  -0.0137720  0.0002929 -47.023
## text_orderFaerber                                0.1533856  0.1252755   1.224
## s_age                                           -0.0187952  0.0002916 -64.467
## s_sexmale                                        0.0692701  0.0003087 224.379
## s_schoolReal                                     0.0396559  0.0003087 128.453
## s_schoolAbi                                      0.7117213  0.1426882   4.988
## as.factor(s_interest)5                           0.1420950  0.1602276   0.887
## as.factor(s_interest)6                           0.0695439  0.1709242   0.407
## as.factor(s_interest)7                          -0.0276561  0.1895659  -0.146
## as.factor(s_interest)8                          -0.1519852  0.2047080  -0.742
##                                                 Pr(>|z|)    
## H4_interactioncausality statement.new guideline  < 2e-16 ***
## summaryFaerber                                   < 2e-16 ***
## text_orderFaerber                                  0.221    
## s_age                                            < 2e-16 ***
## s_sexmale                                        < 2e-16 ***
## s_schoolReal                                     < 2e-16 ***
## s_schoolAbi                                      6.1e-07 ***
## as.factor(s_interest)5                             0.375    
## as.factor(s_interest)6                             0.684    
## as.factor(s_interest)7                             0.884    
## as.factor(s_interest)8                             0.458    
## ---
## Signif. codes:  0 '***' 0.001 '**' 0.01 '*' 0.05 '.' 0.1 ' ' 1
## 
## Threshold coefficients:
##         Estimate Std. Error  z value
## -6|-5 -6.9056679  0.7116693   -9.703
## -5|-4 -5.8028048  0.4172264  -13.908
## -4|-3 -2.6876127  0.1282342  -20.959
## -3|-2 -2.2936112  0.1172157  -19.567
## -2|-1 -1.3970174  0.1001405  -13.951
## -1|0  -0.9333214  0.0939286   -9.936
## 0|1    0.1627729  0.0795859    2.045
## 1|2    0.4996759  0.0731177    6.834
## 2|3    1.4471730  0.0002929 4941.213
## 3|4    1.8132166  0.0002929 6191.050
## 4|5    3.6380499  0.2047215   17.771
## 5|6    3.9117980  0.2365570   16.536
## (21 Beobachtungen als fehlend gelöscht)
```

```
exp(coef(causality_model10_pass1))
```

```
##                                           -6|-5 
##                                     0.001002090 
##                                           -5|-4 
##                                     0.003019075 
##                                           -4|-3 
##                                     0.068043185 
##                                           -3|-2 
##                                     0.100901430 
##                                           -2|-1 
##                                     0.247333552 
##                                            -1|0 
##                                     0.393245397 
##                                             0|1 
##                                     1.176769375 
##                                             1|2 
##                                     1.648186979 
##                                             2|3 
##                                     4.251079634 
##                                             3|4 
##                                     6.130133711 
##                                             4|5 
##                                    38.017626594 
##                                             5|6 
##                                    49.988751993 
## H4_interactioncausality statement.new guideline 
##                                     1.108505925 
##                                  summaryFaerber 
##                                     0.986322408 
##                               text_orderFaerber 
##                                     1.165774459 
##                                           s_age 
##                                     0.981380279 
##                                       s_sexmale 
##                                     1.071725621 
##                                    s_schoolReal 
##                                     1.040452703 
##                                     s_schoolAbi 
##                                     2.037495337 
##                          as.factor(s_interest)5 
##                                     1.152686098 
##                          as.factor(s_interest)6 
##                                     1.072019121 
##                          as.factor(s_interest)7 
##                                     0.972722843 
##                          as.factor(s_interest)8 
##                                     0.859000973
```

```
exp(confint(causality_model10_pass1))
```

```
##                                                        2.5 %       97.5 %
## -6|-5                                           2.483891e-04  0.004042784
## -5|-4                                           1.332693e-03  0.006839394
## -4|-3                                           5.292142e-02  0.087485848
## -3|-2                                           8.019053e-02  0.126961355
## -2|-1                                           2.032560e-01  0.300969675
## -1|0                                            3.271233e-01  0.472732925
## 0|1                                             1.006810e+00  1.375418897
## 1|2                                             1.428133e+00  1.902148505
## 2|3                                             4.248640e+00  4.253520584
## 3|4                                             6.126616e+00  6.133653593
## 4|5                                             2.545222e+01 56.786395131
## 5|6                                             3.144232e+01 79.474905310
## H4_interactioncausality statement.new guideline 1.107835e+00  1.109176860
## summaryFaerber                                  9.857564e-01  0.986888754
## text_orderFaerber                               9.119684e-01  1.490216178
## s_age                                           9.808197e-01  0.981941227
## s_sexmale                                       1.071077e+00  1.072374296
## s_schoolReal                                    1.039823e+00  1.041082447
## s_schoolAbi                                     1.540424e+00  2.694964597
## as.factor(s_interest)5                          8.420252e-01  1.577963827
## as.factor(s_interest)6                          7.668520e-01  1.498626803
## as.factor(s_interest)7                          6.708575e-01  1.410418301
## as.factor(s_interest)8                          5.751034e-01  1.283043591
```

```
nagelkerke(fit = causality_model10_pass1, null = causality_null_pass1)
```

```
## $Models
##                                                                                                                                                                  
## Model: "clmm, as.factor(s_causality) ~ H4_interaction + summary + text_order + s_age + s_sex + s_school + as.factor(s_interest) + (1 | id), data2_long_pass_reg1"
## Null:  "clm, as.factor(s_causality) ~ 1, data2_long_pass_reg1, logit"                                                                                            
## 
## $Pseudo.R.squared.for.model.vs.null
##                              Pseudo.R.squared
## McFadden                            0.0149556
## Cox and Snell (ML)                  0.0626937
## Nagelkerke (Cragg and Uhler)        0.0635310
## 
## $Likelihood.ratio.test
##  Df.diff LogLik.diff  Chisq    p.value
##      -12     -29.556 59.112 3.2748e-08
## 
## $Number.of.observations
##           
## Model: 913
## Null:  913
## 
## $Messages
## [1] "Note: For models fit with REML, these statistics are based on refitting with ML"
## 
## $Warnings
## [1] "None"
```

```
H4test_pass1 = emmeans(causality_model10_pass1, ~ H4_interaction)
pairs(H4test_pass1, adjust = "tukey")
```

```
##  contrast                                                                
##  no causality statement.old guideline - causality statement.new guideline
##  estimate       SE  df  z.ratio p.value
##    -0.103 0.000309 Inf -333.680  <.0001
## 
## Results are averaged over the levels of: summary, text_order, s_sex, s_school, s_interest 
## Note: contrasts are still on the as.factor scale
```

```
cld(H4test_pass1, Letters = letters)
```

```
##  H4_interaction                       emmean    SE  df asymp.LCL asymp.UCL
##  no causality statement.old guideline  0.192 0.108 Inf   -0.0202     0.404
##  causality statement.new guideline     0.295 0.108 Inf    0.0828     0.507
##  .group
##   a    
##    b   
## 
## Results are averaged over the levels of: summary, text_order, s_sex, s_school, s_interest 
## Results are given on the as.factor (not the response) scale. 
## Confidence level used: 0.95 
## Note: contrasts are still on the as.factor scale 
## significance level used: alpha = 0.05 
## NOTE: If two or more means share the same grouping symbol,
##       then we cannot show them to be different.
##       But we also did not show them to be the same.
```

```
data2_long_pass_reg2 <- subset(data2_long_pass, condition == 4 | condition == 6)
View(data2_long_pass_reg2)

causality_null_pass2 <- clm(as.factor(s_causality) ~ 1, data = data2_long_pass_reg2, link = "logit")

causality_model10_pass2 <- clmm(as.factor(s_causality) ~ H4_interaction +
                           summary + text_order + s_age + s_sex + s_school +
                           as.factor(s_interest) + (1|id), data = data2_long_pass_reg2)

summary(causality_model10_pass2)
```

```
## Cumulative Link Mixed Model fitted with the Laplace approximation
## 
## formula: as.factor(s_causality) ~ H4_interaction + summary + text_order +  
##     s_age + s_sex + s_school + as.factor(s_interest) + (1 | id)
## data:    data2_long_pass_reg2
## 
##  link  threshold nobs logLik   AIC     niter      max.grad cond.H 
##  logit flexible  886  -1888.59 3825.17 2734(7833) 5.28e+02 1.8e+07
## 
## Random effects:
##  Groups Name        Variance Std.Dev.
##  id     (Intercept) 0.5044   0.7102  
## Number of groups:  id 451 
## 
## Coefficients:
##                                                   Estimate Std. Error  z value
## H4_interactioncausality statement.new guideline  0.1257058  0.0003288  382.266
## summaryFaerber                                  -0.1468639  0.0003076 -477.434
## text_orderFaerber                                0.1254237  0.0003288  381.409
## s_age                                           -0.0188970  0.0003052  -61.911
## s_sexmale                                        0.0199916  0.1205199    0.166
## s_schoolReal                                     0.0433673  0.1420353    0.305
## s_schoolAbi                                      0.6290552  0.0003288 1912.933
## as.factor(s_interest)5                           0.2701188  0.1624932    1.662
## as.factor(s_interest)6                           0.0849287  0.0003288  258.265
## as.factor(s_interest)7                          -0.0314668  0.1701220   -0.185
## as.factor(s_interest)8                          -0.1163818  0.1769264   -0.658
##                                                 Pr(>|z|)    
## H4_interactioncausality statement.new guideline   <2e-16 ***
## summaryFaerber                                    <2e-16 ***
## text_orderFaerber                                 <2e-16 ***
## s_age                                             <2e-16 ***
## s_sexmale                                         0.8683    
## s_schoolReal                                      0.7601    
## s_schoolAbi                                       <2e-16 ***
## as.factor(s_interest)5                            0.0964 .  
## as.factor(s_interest)6                            <2e-16 ***
## as.factor(s_interest)7                            0.8533    
## as.factor(s_interest)8                            0.5107    
## ---
## Signif. codes:  0 '***' 0.001 '**' 0.01 '*' 0.05 '.' 0.1 ' ' 1
## 
## Threshold coefficients:
##         Estimate Std. Error   z value
## -6|-5 -7.5479671  0.9737526    -7.751
## -5|-4 -5.7848992  0.3987859   -14.506
## -4|-3 -2.7182867  0.0640073   -42.468
## -3|-2 -2.2906987  0.0003076 -7446.848
## -2|-1 -1.4298641  0.0003076 -4648.335
## -1|0  -1.0263013  0.0450119   -22.801
## 0|1    0.0351914  0.0741094     0.475
## 1|2    0.3687718  0.0807433     4.567
## 2|3    1.3933938  0.1061048    13.132
## 3|4    1.6574412  0.1145196    14.473
## 4|5    3.2154967  0.2024812    15.880
## 5|6    3.3274795  0.2114865    15.734
## (20 Beobachtungen als fehlend gelöscht)
```

```
exp(coef(causality_model10_pass2))
```

```
##                                           -6|-5 
##                                    5.271807e-04 
##                                           -5|-4 
##                                    3.073620e-03 
##                                           -4|-3 
##                                    6.598771e-02 
##                                           -3|-2 
##                                    1.011957e-01 
##                                           -2|-1 
##                                    2.393414e-01 
##                                            -1|0 
##                                    3.583299e-01 
##                                             0|1 
##                                    1.035818e+00 
##                                             1|2 
##                                    1.445958e+00 
##                                             2|3 
##                                    4.028499e+00 
##                                             3|4 
##                                    5.245871e+00 
##                                             4|5 
##                                    2.491567e+01 
##                                             5|6 
##                                    2.786801e+01 
## H4_interactioncausality statement.new guideline 
##                                    1.133949e+00 
##                                  summaryFaerber 
##                                    8.634115e-01 
##                               text_orderFaerber 
##                                    1.133629e+00 
##                                           s_age 
##                                    9.812805e-01 
##                                       s_sexmale 
##                                    1.020193e+00 
##                                    s_schoolReal 
##                                    1.044321e+00 
##                                     s_schoolAbi 
##                                    1.875837e+00 
##                          as.factor(s_interest)5 
##                                    1.310120e+00 
##                          as.factor(s_interest)6 
##                                    1.088639e+00 
##                          as.factor(s_interest)7 
##                                    9.690231e-01 
##                          as.factor(s_interest)8 
##                                    8.901353e-01
```

```
exp(confint(causality_model10_pass2))
```

```
##                                                        2.5 %       97.5 %
## -6|-5                                           7.818074e-05  0.003554834
## -5|-4                                           1.406705e-03  0.006715794
## -4|-3                                           5.820763e-02  0.074807686
## -3|-2                                           1.011347e-01  0.101256761
## -2|-1                                           2.391972e-01  0.239485791
## -1|0                                            3.280717e-01  0.391378742
## 0|1                                             8.957800e-01  1.197748021
## 1|2                                             1.234317e+00  1.693886605
## 2|3                                             3.272100e+00  4.959750528
## 3|4                                             4.191200e+00  6.565938307
## 4|5                                             1.675407e+01 37.053119016
## 5|6                                             1.841146e+01 42.181654180
## H4_interactioncausality statement.new guideline 1.133218e+00  1.134679614
## summaryFaerber                                  8.628911e-01  0.863932190
## text_orderFaerber                               1.132898e+00  1.134359604
## s_age                                           9.806936e-01  0.981867670
## s_sexmale                                       8.055557e-01  1.292019127
## s_schoolReal                                    7.905575e-01  1.379541770
## s_schoolAbi                                     1.874629e+00  1.877046791
## as.factor(s_interest)5                          9.527887e-01  1.801464081
## as.factor(s_interest)6                          1.087938e+00  1.089341364
## as.factor(s_interest)7                          6.942662e-01  1.352515403
## as.factor(s_interest)8                          6.292975e-01  1.259087763
```

```
nagelkerke(fit = causality_model10_pass2, null = causality_null_pass2)
```

```
## $Models
##                                                                                                                                                                  
## Model: "clmm, as.factor(s_causality) ~ H4_interaction + summary + text_order + s_age + s_sex + s_school + as.factor(s_interest) + (1 | id), data2_long_pass_reg2"
## Null:  "clm, as.factor(s_causality) ~ 1, data2_long_pass_reg2, logit"                                                                                            
## 
## $Pseudo.R.squared.for.model.vs.null
##                              Pseudo.R.squared
## McFadden                            0.0138339
## Cox and Snell (ML)                  0.0580506
## Nagelkerke (Cragg and Uhler)        0.0588307
## 
## $Likelihood.ratio.test
##  Df.diff LogLik.diff  Chisq   p.value
##      -12     -26.493 52.986 4.143e-07
## 
## $Number.of.observations
##           
## Model: 886
## Null:  886
## 
## $Messages
## [1] "Note: For models fit with REML, these statistics are based on refitting with ML"
## 
## $Warnings
## [1] "None"
```

```
H4test_pass2 = emmeans(causality_model10_pass2, ~ H4_interaction)
pairs(H4test_pass2, adjust = "tukey")
```

```
##  contrast                                                                
##  no causality statement.old guideline - causality statement.new guideline
##  estimate       SE  df  z.ratio p.value
##    -0.126 0.000329 Inf -382.266  <.0001
## 
## Results are averaged over the levels of: summary, text_order, s_sex, s_school, s_interest 
## Note: contrasts are still on the as.factor scale
```

```
cld(H4test_pass2, Letters = letters)
```

```
##  H4_interaction                       emmean    SE  df asymp.LCL asymp.UCL
##  no causality statement.old guideline  0.259 0.121 Inf    0.0216     0.496
##  causality statement.new guideline     0.384 0.121 Inf    0.1473     0.621
##  .group
##   a    
##    b   
## 
## Results are averaged over the levels of: summary, text_order, s_sex, s_school, s_interest 
## Results are given on the as.factor (not the response) scale. 
## Confidence level used: 0.95 
## Note: contrasts are still on the as.factor scale 
## significance level used: alpha = 0.05 
## NOTE: If two or more means share the same grouping symbol,
##       then we cannot show them to be different.
##       But we also did not show them to be the same.
```

### H4 Graphical

```
describeBy(data2_long_pass_reg$s_causality,
           data2_long_pass_reg$H4_interaction)
```

```
## 
##  Descriptive statistics by group 
## group: no causality statement.new guideline
##    vars   n  mean  sd median trimmed  mad min max range skew kurtosis   se
## X1    1 928 -0.26 2.5      0   -0.36 2.97  -6   6    12 0.18    -0.61 0.08
## ------------------------------------------------------------ 
## group: no causality statement.old guideline
##    vars   n  mean   sd median trimmed  mad min max range skew kurtosis   se
## X1    1 486 -0.02 2.52      0   -0.07 2.97  -5   6    11 0.18    -0.57 0.11
## ------------------------------------------------------------ 
## group: causality statement.new guideline
##    vars   n mean   sd median trimmed  mad min max range skew kurtosis   se
## X1    1 827 0.06 2.55      0    0.02 2.97  -6   6    12 0.14    -0.56 0.09
```

```
H4_bar <- ggplot(data2_long_pass_reg, aes(H4_interaction,
[truncated: 97,894 more chars]
