# Supplementary material for: Equivalent user experience and improved community augmented meta-analyses knowledge for a new version of a Plain Language Summary guideline
Source: PLoS One. 2024 May 9;19(5):e0300675. doi: 10.1371/journal.pone.0300675 (PMC11081257; doi:10.1371/journal.pone.0300675)
Supplement: Attachment — Submitted filename: Response to Reviewers.docx [file pone.0300675.s006.docx]

Rebuttal Letter

- Please ensure that your manuscript meets PLOS ONE's style requirements, including those for file naming. The PLOS ONE style templates can be found at <https://journals.plos.org/plosone/s/file?id=wjVg/PLOSOne_formatting_sample_main_body.pdf> and <https://journals.plos.org/plosone/s/file?id=ba62/PLOSOne_formatting_sample_title_authors_affiliations.pdf>

The entire manuscript has been reviewed and revised in order to adequately comply with PLOS ONE’s formatting and file naming guidelines. In addition, the file names were revised and the files were reuploaded.

- At this time, please address the following queries:
  a) Please clarify the sources of funding (financial or material support) for your study. List the grants or organizations that supported your study, including funding received from your institution.
  b) State what role the funders took in the study. If the funders had no role in your study, please state: “The funders had no role in study design, data collection and analysis, decision to publish, or preparation of the manuscript.”
  c) If any authors received a salary from any of your funders, please state which authors and which funders.
  d) If you did not receive any funding for this study, please state: “The authors received no specific funding for this work.”
  Please include your amended statements within your cover letter; we will change the online submission form on your behalf.

  As requested, a new funding statement has been added to our cover letter that addresses the four queries above.
- When completing the data availability statement of the submission form, you indicated that you will make your data available on acceptance. We strongly recommend all authors decide on a data sharing plan before acceptance, as the process can be lengthy and hold up publication timelines. Please note that, though access restrictions are acceptable now, your entire data will need to be made freely accessible if your manuscript is accepted for publication. This policy applies to all data except where public deposition would breach compliance with the protocol approved by your research ethics board. If you are unable to adhere to our open data policy, please kindly revise your statement to explain your reasoning and we will seek the editor's input on an exemption. Please be assured that, once you have provided your new statement, the assessment of your exemption will not hold up the peer review process.

All authors agreed on the current data sharing plan before submitting our manuscript to PLOS ONE. Supplements and Materials shared via a “PsychArchives Review Link” , while currently anonymised and limited in terms of accessibility, have already been reviewed by the repository staff and are eligible for inclusion in the repository. In case of an acceptance, the data can be made openly accessible within 1-2 business days and receive a DOI that can be included in the final article instead of the review link. In light of the typical editorial process (i.e. typesetting and corrections), the data and all Supplemental Materials are going to be available upon publication.

As for data availability, our study protocol and the connected ethics vote requires us to exclude data that could lead to a personal identification of participants (i.e. age, gender, educational attainment level). Therefore, datasets including this information cannot be uploaded without causing a breach of our approved study protocol. The data availability statement has been modified to reflect this:

*Data is available, but as per the study protocol, any data that could be used to identify individual participants (age, sex, educational attainment level) had to be excluded from the final datasets.*

- 4. Please review your reference list to ensure that it is complete and correct. If you have cited papers that have been retracted, please include the rationale for doing so in the manuscript text, or remove these references and replace them with relevant current references. Any changes to the reference list should be mentioned in the rebuttal letter that accompanies your revised manuscript. If you need to cite a retracted article, indicate the article’s retracted status in the References list and also include a citation and full reference for the retraction notice.

The references have undergone an additional review to ensure correctness and

completeness. No retracted articles were cited, and all included references are, to the best of our knowledge, cited correctly. Six additional publications were cited in order to adequately respond to reviewer requests (see revised manuscript).

## Editorial Manager

- In the end of each Results section, you mention if a numbered hypothesis is supported. Instead of just listing the number, a short textual reminder of what that hypothesis is would be helpful for the reader.

We thank the editorial manager for this suggestion. To make the content of the
hypotheses clearer, short textual reminders have now been added for all
confirmatory hypotheses (H1-H6). This should help to make our results clearer for
the readers.

## Reviewer 1

This paper is generally scientifically sound but with two notable issues that require clarification. I also have a handful of comments about things that would be helpful to clarify or highlight to aid the reader in interpreting and perhaps applying these results.

We thank Reviewer 1 for his time, effort and comments on our manuscript. Taking his comments into consideration helped to further explore our study results and improve the quality of our manuscript.

- The first issue has to do with the nature of some of the comparisons in relation to the six conditions the authors used. In short, almost all of the significant results seem to be found in highly asymmetric comparisons, in which one group is 3x-5x the size of the group it's being compared to. For example, for H1a-H5a, all of the comparisons against the old guidelines are combinations of three conditions versus the one condition that saw the old guidelines. The CAMA analysis is the same thing in reverse: only one condition saw the CAMA statement, and it is compared against the five that did not. The authors should make clear whether this imbalance affects the interpretation of their results, whether it is incorporated into the underlying assumptions of their analytical approach, and perhaps whether these comparisons hold up when individual conditions are compared pairwise in post-hoc tests.

These are valid issues to point out, and we are happy to offer some clarification.
To begin with, we followed the reviewer’s suggestion to include additional post hoc
tests.These tests confirmed our initial results. They have been added under the
header “post hoc” to each confirmatory analysis step (H1-H6) in both the updated
complete (S4) and new shortened version (S5) of the R Markdown.
We would also like to emphasize that most of the results that compared the old
guideline to the new guidelines did not reach significance (H1a, H2a, H3a, H4a) and
produced only small effects (typically around R² = .10 or lower). These non-significant
results emerged both in the logistic regressions and the Wilcoxon tests. For the
complete dataset, our a priori power analysis ensured that we can reliably interpret
these results. While the power may be lower for the subset of participants who
passed the awareness check due to a smaller sample size, we would still argue that
these non-significant findings hold due to the small effect sizes.

As for H5, the reviewer’s comments allowed us to correct the above-mentioned error
in the analysis script, and the new analysis with more balanced groups still revealed
significant differences in CAMA knowledge for the new guideline with CAMA
elements compared to the old guideline, OR = 1.61, 95%CI (1.16, 2.24), *p* <. 01.

For the non-inferiority test in H6, we now included additional post hoc non-inferiority	tests for each of the five experimental conditions against condition 6 (i.e. the old
guideline).

We would also like to offer some additional clarification regarding the asymmetric
group sizes: These typically occur when the old guideline is compared to two
variations of the new guideline conditions (e.g. new guideline - no disclaimer and new
guideline - disclaimer). This is perhaps most noticeable in the cumulative link and
cumulative link mixed model logistic regression analyses. While unbalanced group
sizes can potentially bias model parameters in logistic regressions, this often only
starts to pose problems in rare event analysis, where the instances of an event
compared to all cases makes up about 5 % or less. For an in-depth discussion of the
topic, see:

[King G, Zeng L. Logistic Regression in Rare Events Data. Polit Anal.
2001;9(2):137–63.](https://www.zotero.org/google-docs/?V6qurM)

In our logistic regression models, old guideline datapoints make up the following percentage share of total cases:

- H1: 17.87 % (247 cases from 1382 cases total)
- H2: 17.87 % (247 cases from 1382 cases total)
- H3: 17.87% (247 cases from 1382 cases total)
  H4: 17.87% (494 cases from 2764 cases total)
- H5: 35.80% (247 cases from 690 cases total). Regarding H5, the reviewer’s comments pointed us towards a mistake in our R analyses script. In the original version of the script, all 5 other conditions were compared to the PLS based on the new guideline with CAMA elements. However, we originally only intended to compare condition 4 (new guideline PLS with all additional elements except CAMA) and condition 6 to the CAMA PLS (see Preregistration, Table 1). The reason behind this is that the CAMA PLS included both a disclaimer and a causality statement, and that we therefore only wanted to compare CAMA PLSs to a similar PLS format without CAMA elements (H4) as well as to the old guideline (H6). Correcting this led to more balanced group sizes (old guideline: 247; new guideline, no CAMA: 206; new guideline, CAMA = 237)
- H6: 17.82% (239 cases from 1341 cases total)

We would thus argue, that, while unbalanced, the case distribution in the present
study does not cause biased results in terms of rare event analysis (King & Zeng,
2001).

- The second issue can be resolved by simply reporting a brief analysis or some additional descriptive information: were the 33% of participants who failed the awareness check evenly distributed across demographic groups and conditions? With regard to conditions, while the individual conditions vary in size by up to ~45 participants depending on the measure, there are no obvious patterns based on the specific factors being manipulated. The authors should present descriptive data or an analysis to demonstrate whether the same is true for the demographic factors considered, and if not, consider whether this impacts the interpretation of some of the effects of demographics (which were consistent throughout).

We thank Reviewer 1 for pointing this out and have conducted additional chi²-Tests to determine whether the frequencies of awareness-check-pass results deviate significantly from chance in terms of experimental conditions and demographic variables. The analyses can be found in the complete Markdown (S4) and a new, shortened Markdown version (S5) in the section Descriptive Analyses - Awareness Check.

- For experimental condition, the Chi²-test reached significance, 𝜒² (5) = 17.32, *p* <.01. This is likely caused by a larger than expected number of participants failing the awareness check in condition 4, as indicated by a significant standardized residual of *z* = 2.391.
- The Chi²-test for participant’s sex reached significance, 𝜒² (1) = 8.30, *p* < .01. However, no significant standardized residuals were observed, indicating that there were not more or fewer cases of awareness check = pass or fail in either the male or female group.
- For participant’s age, a histogram depicting the awareness check results (i.e. pass/fail) has been added for each age group (see below), and a Chi² test has been conducted with the two age groups of 18-44 and <45. The test reached significance , 𝜒² (1) = 62.25, *p* <.001. Based on significant standardized residuals, more participants than expected failed the awareness check in the younger age group (*z* =4.601) and passed the awareness check in the older age group (*z* = 3.162). Furthermore, fewer participants than expected passed the awareness check in the younger age group (*z* = -3.175 and failed the awareness check in the older age group (*z* = -4.583). While these results may point towards issues with awareness for younger readers, a more fine-grained analysis of demographic effects is beyond our study’s aim of broadly testing the effects of different PLS features for a wide range of layreaders.

- For participants’ educational background, a Chi² test comparing the three educational levels reached significance, 𝜒² (1) = 8.30, *p* < .01. Significant standardized residuals revealed that more participants than expected failed the awareness check (*z* = 3.308) and fewer participants than expected passed the awareness check (*z* = -2.282) in the subgroup with a Hauptschule-degree (lower secondary track). In addition, fewer participants than expected failed the awareness-check (*z* = -3.363) and more participants than expected passed the awareness check (*z* = 2.320) in the Abitur-subgroup (higher educational track). All other residuals remained non-significant. Explanations could be differences in reading abilities or attention between the different subgroups. Implications for the creation of PLSs for participants with a lower level of education are discussed, however, a more fine-grained analysis of demographic influences was beyond the scope of the present study.

In order to keep our manuscript concise, we chose to refer to these results in the
main text by including the following passage:

[pp. 20-21, ll. 418-420] *Distributions of cases where the awareness check failed
across demographic variables such as age, gender or educational background are
available in S4 and S5 File.*

- First, was the goal to get a demographically representative sample, or a demographically balanced sample? Frankly I don't know the demographics of the German populace to say whether those are going to be the same or not, but based on the breakdown described by the authors, it sounds like they went for a balanced sample where each demographic group had roughly the same number of participants. However, they cite the fact that they use a representative sample as one of the strengths of this work on line 675. Is that truly representative of the proportions of these demographic groups in the German population as a whole? This is worth considering because it affects how this kind of work may be used in the future. There is an effect of age on almost every measure, for example, but if one age group is overrepresented in the general population, and it's the age group that showed overall worse performance, then perhaps this whole approach needs to be reconsidered.

Reviewing this comment helped us to realize that the nature of our sample may
require additional clarification. In total, we formed twelve different quota conditions
based on sex (male vs. female), educational attainment (Hauptschule, Mittlere Reife ,
Abitur) and age group (18-44, 45 and older), aiming for 365 participants in each
quota condition. Reviewer 1 pointed out correctly that this would be a balanced
demographic sample instead of a truly representative sample of the German general
population. As such, the following adjustments were made to the manuscript:

[p. 16, ll. 313-319] *To recruit approximately the same proportion of participants in
terms of age group (50.20 % 18 - 44 years, 49.80 % 45 years or older, M = 45.22,
SD = 15.23, range = 18 - 90 years), sex (50.37 % women, 49.63 % men), and
education level (33.56 % “Hauptschulabschluss”, 33.37 % “Mittlere Reife”, 33.07%
“Hochschulreife”), we applied quotas (see Preregistration for more details). It is
noteworthy that this resulted in the collection of a balanced demographic sample,
rather than a sample precisely representing the age, sex or educational distributions
in the German general population.*

[p. 42, ll. 760-763] *Second, we collected data from a large sample of the
German-speaking general population, which increases the generalizability of our
findings beyond a purely academic context. To achieve this, we aimed for a balanced
sample, with an approximately equal number of participants in each demographic
category.*

- Second, it was not clear why some measures or factors were important. For example, H1ab and H3ab could use some additional justification. Why is it important that lay readers understand the difference between the PLS and the meta-review? Why does it matter that they have different authors? What are the ramifications of these differences for the public's understanding of the underlying science? I have similar concerns about H5ab, and the entire CAMA question. It would be helpful if the authors explained why it is important to know whether the public is sensitive to these features, and what ramifications this sensitivity might have.

  The points raised by Reviewer 1 helped us to rework passages where our line of argument could be improved in order to further underline the relevance of laypeoples’ ability to differentiate between PLSs and meta-analyses, as well as between PLSs based on CAMA and traditional meta-analyses. The following passages have been added:

  [p. 5, ll. 86 -99] *Knowledge of this fact is relevant for multiple reasons: First, PLSs are frequently written by third-party authors not involved in the original studies. These third-party authors may emphasize different aspects of a main study’s results in their PLS, e.g. by highlighting them more prominently or mentioning them first. For example, authors may be more inclined to spotlight treatment outcomes for one particular approach of psychotherapy compared to other approaches, depending on their work approach or therapeutic background. And second, PLSs often aim to concisely convey the key points of a study. However, this may come with the potential risk of leaving out more specific information (e.g. the effectiveness of a therapy approach for a specific subgroup or higher-level interaction effects). Both issues may nudge readers’ process of informed decision-making in a certain direction. With this in mind, is there a way of supporting laypeople in grasping author differences between an original study and its subsequent PLS? One possible solution could be to explicitly state the difference between the two formats via a disclaimer.*

  [p.7, ll. 140-144] *Given the fact that source features are rarely considered, this may be challenging to comprehend. Readers may simply assume that both texts stem from researchers of the original meta-analysis. However, this assumption may pose risks, such as failing to grasp that a different set of authors may influence the level of priority given to particular results.*

[pp. 8-9, ll. 181-189] *This may offer benefits for laypeoples’ decision-making process.
Normally, a meta-analysis’ sample remains set after publication, cannot be
dynamically updated and may therefore become more inaccurate over time, as new
study results become available. Relying on CAMA benefits laypeople in that they are
more likely to receive up-to-date results and in that they can check effects for their
consistency. However, laypeople are likely unaware of the particular features of
CAMA in comparison to a more traditional form of meta-analyses. As such, it seems
worthwhile to examine how their awareness and knowledge of this particular form of
meta-analysis can be increased.*

- Finally, I think more should be said about the demographic effects, since these were highly consistent. Do you have any explanation for them? And, as discussed above, do they have ramifications for the usefulness of these guidelines for effective science communication with different populations?

In order to address this comment and to respond to an additional comment by
reviewer 2 concerning the use of both visual and textual elements in future PLSs, a	new section titled “Demographic Effects” has been added to the discussion section:

[pp. 39 -40, ll. 681-711] *Demographic effects
Generally speaking, two consistent effects of demographic variables on item scores
could be observed in the present study. First, a higher reader age was associated
with lower scores on knowledge items on the relationship between the PLS and the
corresponding meta-analysis (H1), the extent of evaluation (H2), the differentiation
between PLS authors and meta-analysis authors (H3) and the causality of effects
(H4, see Table 2). Reasons for this may include a poorer memory performance
associated with higher age (e.g. in item recognition tasks, see [37]), or a potential
greater familiarity of younger laypeople with short research summaries or info texts
prevalent in online contexts. And second, a higher educational attainment level
(Mittlere Reife or Abitur) generally predicted higher scores regarding knowledge on
the relationship between the PLS and the corresponding meta-analysis (H1), on the
extent of evaluation (H2), on the causality of effects (H4) and on CAMA (H5, see
Table 2). Given that both individual student characteristics such as socio-economic
status (SES) and science self-efficacy as well as school characteristics such as
school-level SES and parental involvement can affect scientific literacy [38], and that
these variables likely reach higher values in connection to secondary school types
such as “Realschule” or “Gymnasium/Gesamtschule” in Germany, a higher level of
scientific literacy may be the driving force behind these findings.
Based on these observations, it may be worthwhile to ask the following question:
How can one support older laypeople or laypeople with a comparatively lower level
of education in processing and understanding psychological PLSs? After all, both
traits seem to be predictive of lower scores on multiple knowledge items. One
approach could be to not only include textual information in PLSs, but to also rely on
elements such as infographics, or to develop PLS formats including both orally
narrated and visually presented information. Based on the cognitive theory of
multimedia learning [39], this approach is likely to have benefits for both laypeople’s’
encoding and retrieval of information, e.g. by reducing the information load that
requires processing. The present study was first and foremost focused on textual
elements in order to create an empirically validated guideline without focusing on
needs of different user groups. As such, we did not take multimedia learning
into account. Future research would benefit from drawing on multimedia principles to
make PLSs more effective and to address particular needs of specific demographic
groups.*

## Reviewer 2:

Dear Authors,

I find your project to be very interesting and believe that your innovative approach to scientific communication has the potential to yield insights while paving the way for fresh avenues of research and refined methods of scientific dissemination. Allow me to share a few comments and suggestions to enhance the impact of your work. I hope you will find them useful.

We would like to thank Reviewer 2 for the positive words about our research approach, his time and efforts and his insightful comments. Taking these into account allowed us to expand on the discussion section of our paper and create a stronger tie between our research findings and ongoing discussions in the field of PLS research and science communication.

- It appears that your project has invested considerable effort and resources into a complex research, yet it provides limited information regarding the practical applicability of PLS/the new guidelines. In your considerations for future research, it may be advantageous to incorporate methods that scrutinize the effectiveness of (the new) PLS using modern user experience (UX) tools such as eye tracking and memory tests. By streamlining your project, focusing on fewer hypotheses and research questions, and employing precise instruments, you could potentially uncover more influential and practical insights.

We agree with Reviewer 2 on the issue that UX tools may prove to be valuable for
future research in terms of understanding laypeoples’ reception of and interaction
with Plain Language Summaries. To underline this, we have added the following
passage to our discussion section:

[pp. 41, ll. 738-745] *Further PLS development would also benefit from a more
in-depth analysis of user experience and user behavior. For example, employing
eye-tracking technology [40] could help to identify laypeople’s gaze patterns, thereby
improving understanding about which PLS passages laypeople focus the most
attention on and which passages they may merely skim. Similarly, recognition and
recall memory tests could be used to further explore differences between future PLS
versions. This could even be combined with time-delayed follow-up tests (e.g. after 2,
4 or 6 weeks) to explore how well laypeople retain information included in PLS in
their long-term memory.*

- Exploring the combination of textual and visual elements as a means to convey the essence of meta-analyses might prove more valuable, even if it deviates (slightly) from the new PLS standards. As such, it could limit the information load.

We thank Reviewer 2 for bringing our attention to the question of the integration of
visual and textual elements in future PLS. This has been a topic that has been
repeatedly mentioned during conference presentations as well as instances of expert
feedback in the context of the development of our guideline. In order to first and
foremost arrive at an empirically validated guideline, we chose to specifically focus
on the impact of textual elements. The decision was made on for
practical and strategic reasons: First, there were institutional requirements that made
it necessary to focus on textual elements. And second, there were resource
limitations to consider: Running multiple empirical studies on psychological PLS while
simultaneously creating and testing graphical illustrations would have gone beyond
the capacity of our core project team. However, a new passage has been added to
the discussion section to adequately consider multimedia principles for future PLS
research (see also our reply to Reviewer 1 regarding demographic
effects).

[pp. 39-40, ll. 681-711]

- The challenge in communicating the results of meta-analyses lies in their abstract nature, demanding a high degree of abstract thinking and prior knowledge. It would be advisable to consider the characteristics of your target audience in future research. Understanding how meta-analytical findings impact professionals and policymakers could be pivotal, and including this in your discussion could enhance the (practical) relevance of your work.

This is a valid point, and we agree with the fact that including a short discussion of
the potential impact of PLSs on the decision-making process of practitioners and
policy-makers will have benefits for the discussion section. We therefore included the
following passage.

[pp. 41-42, ll. 745-753] *And, finally, examining how practitioners such as clinical
psychologists, counselors, child development specialists or recruiters as well as
policymakers draw on PLSs and which PLS aspects may prove beneficial when
transferring information into practical work remains a worthwhile question. Although
initial research on this issue exists for systematic reviews [41,42] and points towards
the benefits of said reviews for practitioners, further studies and more thorough
examinations have the potential to uncover how PLSs can be turned into a more
effective support-tool for decision-making. Investigating practitioners’ or
policymakers’ reactions to PLSs, e.g. in the context of a decision-making task, may
help to illuminate factors that facilitate or impede PLS use among these groups.*

- I appreciate your commitment to open science principles, even beyond the journal's requirements. However, the extensive code within your analysis script can be overwhelming for reviewers who have limited time to verify and reproduce your analyses. To address this, I recommend sharing code that facilitates a step-by-step reproduction of the analyses as presented in your manuscript, ensuring ease of identification and inspection of the most critical analyses.

We are grateful for the positive words regarding our open science approach
and agree that the complete R Markdown script can be daunting if one aims for
insights into our analytic process and our results. As such, we have created a
shortened version of the original Markdown as additional Supplemental Material (S5
File). The new Markdown only includes analyses for the subgroup that passed
the awareness check and leaves out additional exploratory analyses, cutting its
length to 3432 lines of code compared to the 7615 lines of the original Markdown.
